# The Materials Science behind Sustainable Metals and Alloys

**DOI:** 10.1021/acs.chemrev.2c00799

**Published:** 2023-02-27

**Authors:** Dierk Raabe

**Affiliations:** Max-Planck-Institut für Eisenforschung, Max-Planck-Str. 1, 40237 Düsseldorf, Germany

## Abstract

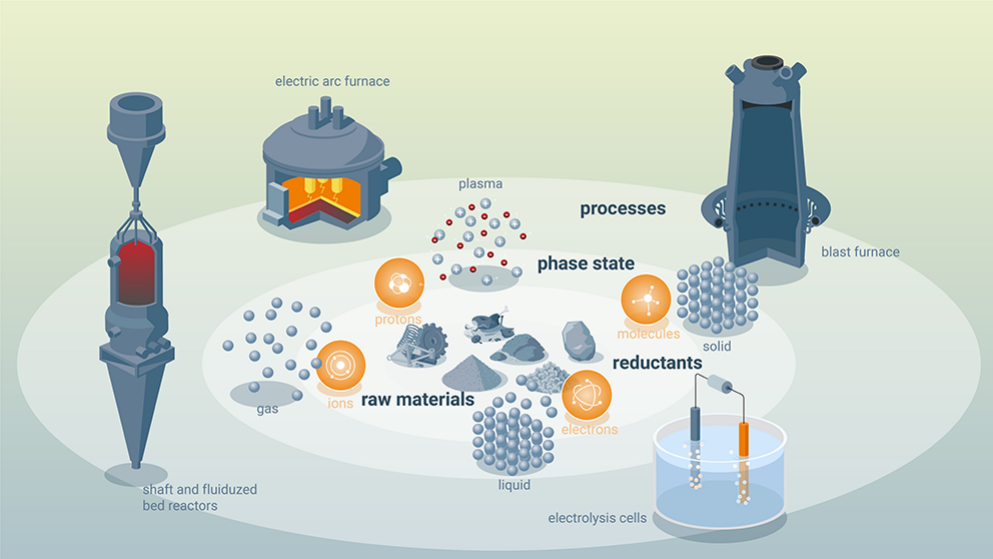

Production of metals stands for 40% of all industrial greenhouse gas emissions, 10% of the global energy consumption, 3.2 billion tonnes of minerals mined, and several billion tonnes of by-products every year. Therefore, metals must become more sustainable. A circular economy model does not work, because market demand exceeds the available scrap currently by about two-thirds. Even under optimal conditions, at least one-third of the metals will also in the future come from primary production, creating huge emissions. Although the influence of metals on global warming has been discussed with respect to mitigation strategies and socio-economic factors, the fundamental materials science to make the metallurgical sector more sustainable has been less addressed. This may be attributed to the fact that the field of sustainable metals describes a global challenge, but not yet a homogeneous research field. However, the sheer magnitude of this challenge and its huge environmental effects, caused by more than 2 billion tonnes of metals produced every year, make its sustainability an essential research topic not only from a technological point of view but also from a basic materials research perspective. Therefore, this paper aims to identify and discuss the most pressing scientific bottleneck questions and key mechanisms, considering metal synthesis from primary (minerals), secondary (scrap), and tertiary (re-mined) sources as well as the energy-intensive downstream processing. Focus is placed on materials science aspects, particularly on those that help reduce CO_2_ emissions, and less on process engineering or economy. The paper does not describe the devastating influence of metal-related greenhouse gas emissions on climate, but scientific approaches how to solve this problem, through research that can render metallurgy fossil-free. The content is considering only direct measures to metallurgical sustainability (production) and not indirect measures that materials leverage through their properties (strength, weight, longevity, functionality).

## 1 Introduction to Sustainable and CO_2_-Reduced Metals and Alloys

### 1.1 The Big Numbers in the Metal Sector

**Figure 1:**
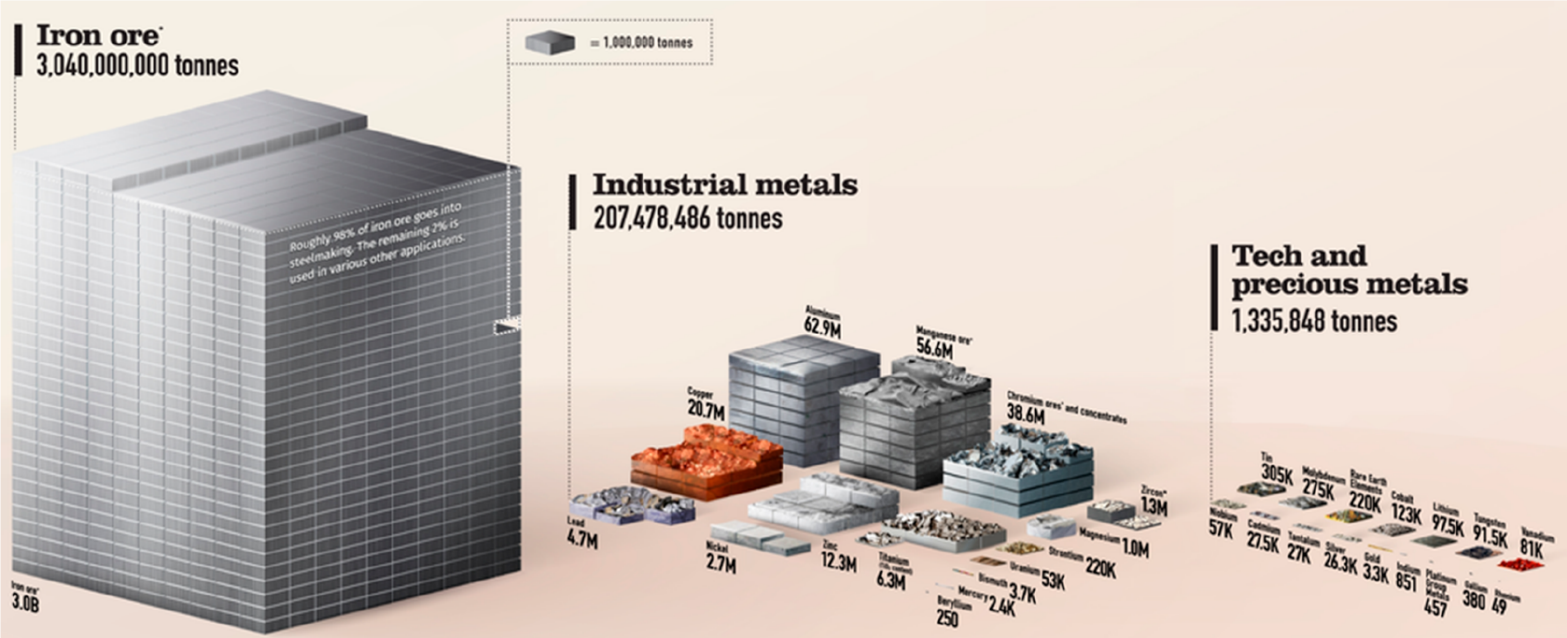
Minerals mined in the year 2019. Full details can be found in several overview works.^1,3,14,19−22^ Figure adapted in modified form with permission from ref (23) (https://www.visualcapitalist.com). Copyright 2019, VisualCaptialist.

**Figure 2:**
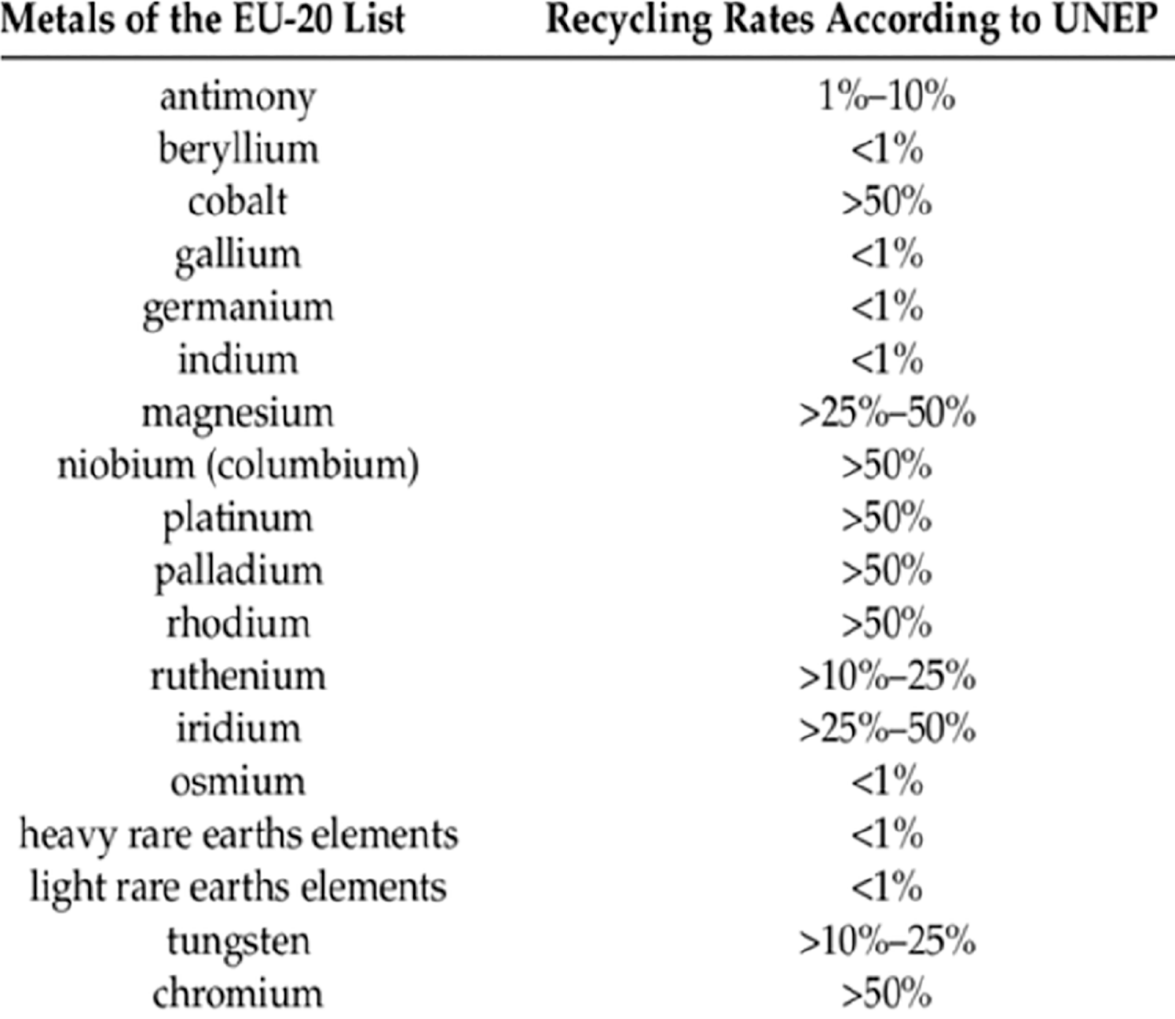
Recycling rates for a few selected strategic metals. Full references can be found in a number of overview papers.^1,3,14,19−22^ Numbers taken with permission from the UN Environment Programme UNEP.^24^ Copyright 2020, UNEP.

The production of currently 2 billion tonnes of metals and alloys per year accounts for around 40% of all industrial greenhouse gas emissions, consumes 10% of the global energy supplies, and requires 3.2 billion tonnes of minerals for primary synthesis,^1−3^Figure 1. In addition, huge amounts of residual and waste products, tailings and removed overburden are generated during mining, production and processing. These substances range from mineral gangue to dusts and processing residues which altogether are about an additional factor of 15–20 larger in volume than the total amount of metal produced itself.^4,5^ These numbers grow fast and will double by 2050.^6^ This creates a high driving force to render a large portion (50–70%) of the material production and manufacturing chain circular, with recycling playing a key role.^7−10^ Yet, recycling rates are often low, particularly for some of the strategically most critical metals with recovery rates being in part below 1%,^11^Figure 2. Several overviews have discussed most of these quantities (both metals and minerals) and the associated emission, mining, waste and recycling problems caused by the metallurgical sector.^1,7,12−18^ Therefore, many of the specific numbers and trends will not be repeated here in full breadth but the reader is referred to some main overviews for details.^1,3,14,19−22^

The sheer magnitude of these numbers explains the significant impact that metal production has on climate, habitats, human living, future metal availability, biodiversity and working conditions.^20,22,25−30^ The most pressing and urgent problem among all these factors is the greenhouse gas emissions.^2,14,31−33^ The vast majority of these emissions stems from the use of fossil reductants in primary metallurgical synthesis, mostly through carbothermic reduction. Further main sources are the consumed electrical power of fossil origin and the use of fossil energy carriers as feedstock for combustion, because most processes in metallurgy require heat. The triggering role of CO_2_ emissions on the greenhouse effect is the most hazardous and imminent of all these impacts, qualifying metal production as the biggest single reason for global warming.

Another challenge is that many of the supply critical metals that are urgently needed for electrification, digitalization, automation, green chemistry and renewable energy supply are scarce, have in part very low recycling rates <1, and are among the highest CO_2_-producing materials per kg of metal produced.^11,34^ The term “recycling rate” refers here to that fraction of material that is scrapped at the end of life of a product and is then reused to make new material, Figure 2.

**Figure 3:**
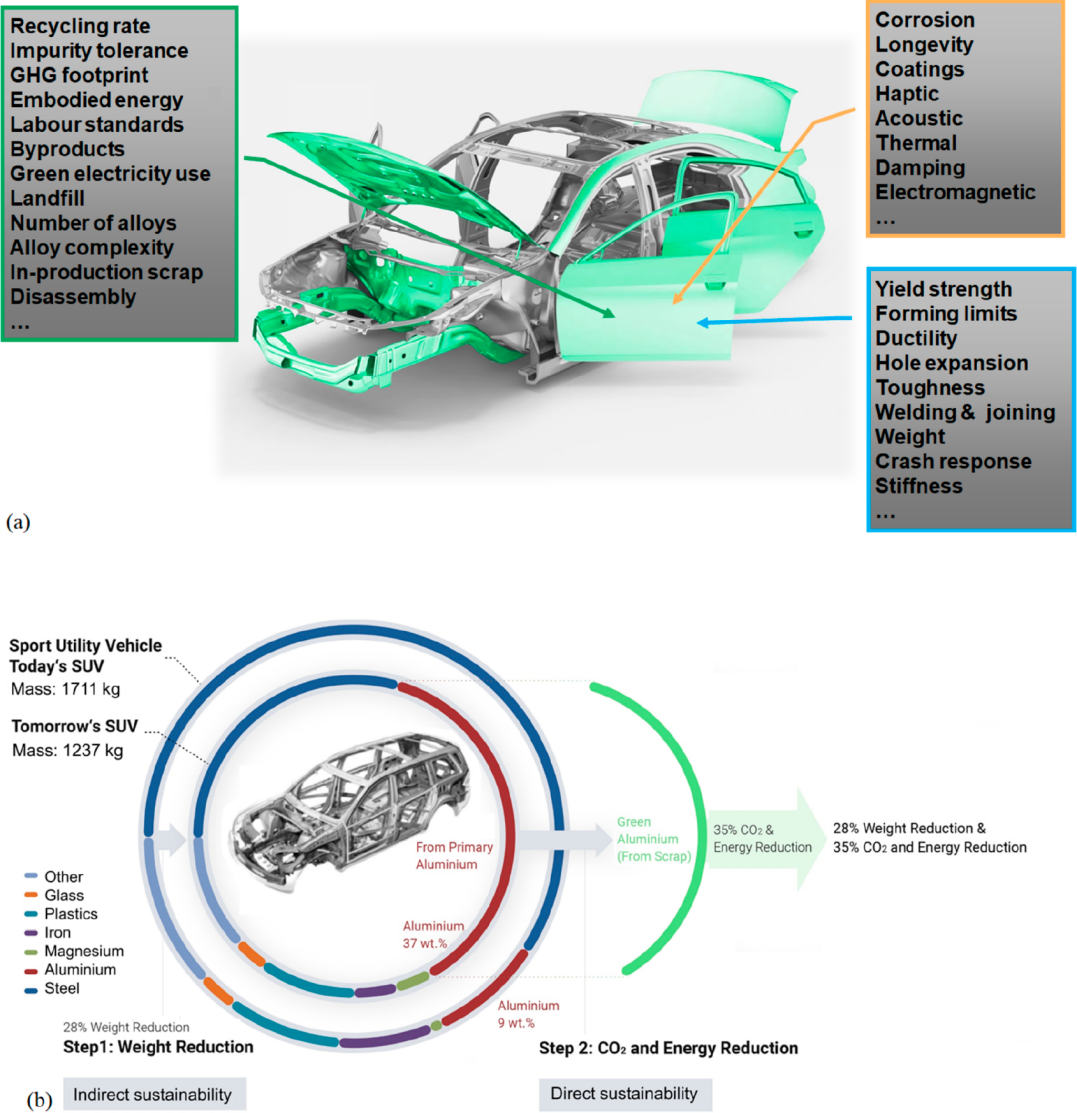
(a) Downstream original equipment manufacturers who are the customers of metal products have started to pick the sustainability trend up, demanding more sustainable and transparent production methods and lower CO_2_ footprint of the metals delivered to them. (b) Sustainable approaches for material development and processing must take into consideration both the indirect benefit of advanced materials, e.g. through weight reduction, and the direct ones, by producing the same or better materials as before, but with lower energy consumption and reduced CO_2_ emissions.^7^ The first step of the approach is referred to as “indirect sustainability” (sustainability gain through material properties) and the second one as “direct sustainability” (sustainability gain through less harmful material production).^2^

The highly necessary system change toward the use of more sustainable technologies in the metal sector has already started to act as a massive driver of market growth and innovation. However, the discussion of this topic should be free from naivety and based on scientific analysis and understanding. It is not just the growing population and increase in average global gross domestic product (GDP) and the associated per capita consumption values that propel the demand for metals, but also the growing massive investments in sustainable technologies themselves that accelerate the environmental burden. Downstream original equipment manufacturers who are the main customers of metal products have started to pick this trend for more sustainably produced metals up, particularly in products of high customer sensitivity and visibility. This means that the downstream markets start to demand from primary producers the implementation of more sustainable production methods and lower CO_2_ footprint of the metals delivered. These actions must not be based on waving-hand or “feel-good” pseudo-argumentation, but they must be substantiated by scientifically well-rooted and transparent life cycle documentation. This means that future metallurgical products will not only have to provide specific mechanical properties such as strength and ductility as well as functional properties such as optical appearance, magnetism or corrosion resistance, but they will also have to comply with certain bounds related to their carbon footprint, recycled metal content, embodied energy, etc.^8,14,21,35,36^ This trend will fundamentally change future relations among market participants, and it requires proper and transparent life cycle documentation along the entire manufacturing chains, starting with mining, metallurgy and recycling, Figure 3. This trend creates not only new market opportunities but also green-technology-related rebound effects.^37^ This means that on the one hand sustainable technologies (such as wind power plants etc.) enable improved sustainability of industry and society but on the other hand they boost the consumption of metals, especially rare and CO_2_-intense materials, and thus create further market and sustainability pressure^38,39^ (see details in section 2).

### 1.2 Abundant, Critical and Rare Metals

The often used terms “critical”, “rare” or “strategic” elements require a more detailed definition in the context of sustainable metallurgy. Regarding mineral abundance, a distinction must be made between reserves, resources and geopotentials.^14,20,21,40^ There are two types of mineral deposits, referred to as “identified resources” and “unexplored resources”. In order to profitably extract a mineral commodity now or in the future, there must be a sufficiently rich concentration of naturally occurring solid, liquid, or gaseous components in the Earth’s crust. A sufficiently promising mineral deposit that has been identified from geologic data in terms of its quality, richness, and location is referred to as an “identified resource” even though it may not have been exploited yet owing to market circumstances.^41−43^ “Undiscovered resources” refer to metal-bearing mineral deposits that are thought to exist but lack solid geological evidence to support them. Such assessments are usually made based on experience, knowledge, and theory-based hypotheses. Another pertinent distinction is the difference between an “ore” and a “mineral”. The term ore refers to reserves of certain mineral commodities, usually metallic oxides, sulfides and carbonates, but it is also used to refer to nonmetallic commodities. More specific, an ore is a type of mineral (mixture) from which a metal can be extracted economically. In most cases ores are naturally occurring blends of different minerals, yet for metallurgical extraction those minerals that are the desired and targeted ones must as a rule be first separated from undesired mineral by-products. This is for instance one of the main reasons for the high environmental burden associated with the production of metals such as nickel, cobalt or copper, whose naturally occurring minerals have very small metal content, in part below 1 wt % (see details in sections 7.4.10 and 7.4.12). A mineral reserve is the percentage of an identified resource from which a useable mineral or energy commodity may be economically and legally removed. A geopotential of a mineral resource is essentially the content that is available in the Earth’s crust, irrespective of its dispersion, local concentration or the capital needed to tap it. The latter quantity (such as graphs showing element reserves in the Earth’s crust) can be misleading because not the integral abundance of an element but its dispersion, enrichment, or respective agglomeration pattern is the decisive criterion for a commercially viable mining operation.

**Figure 4:**
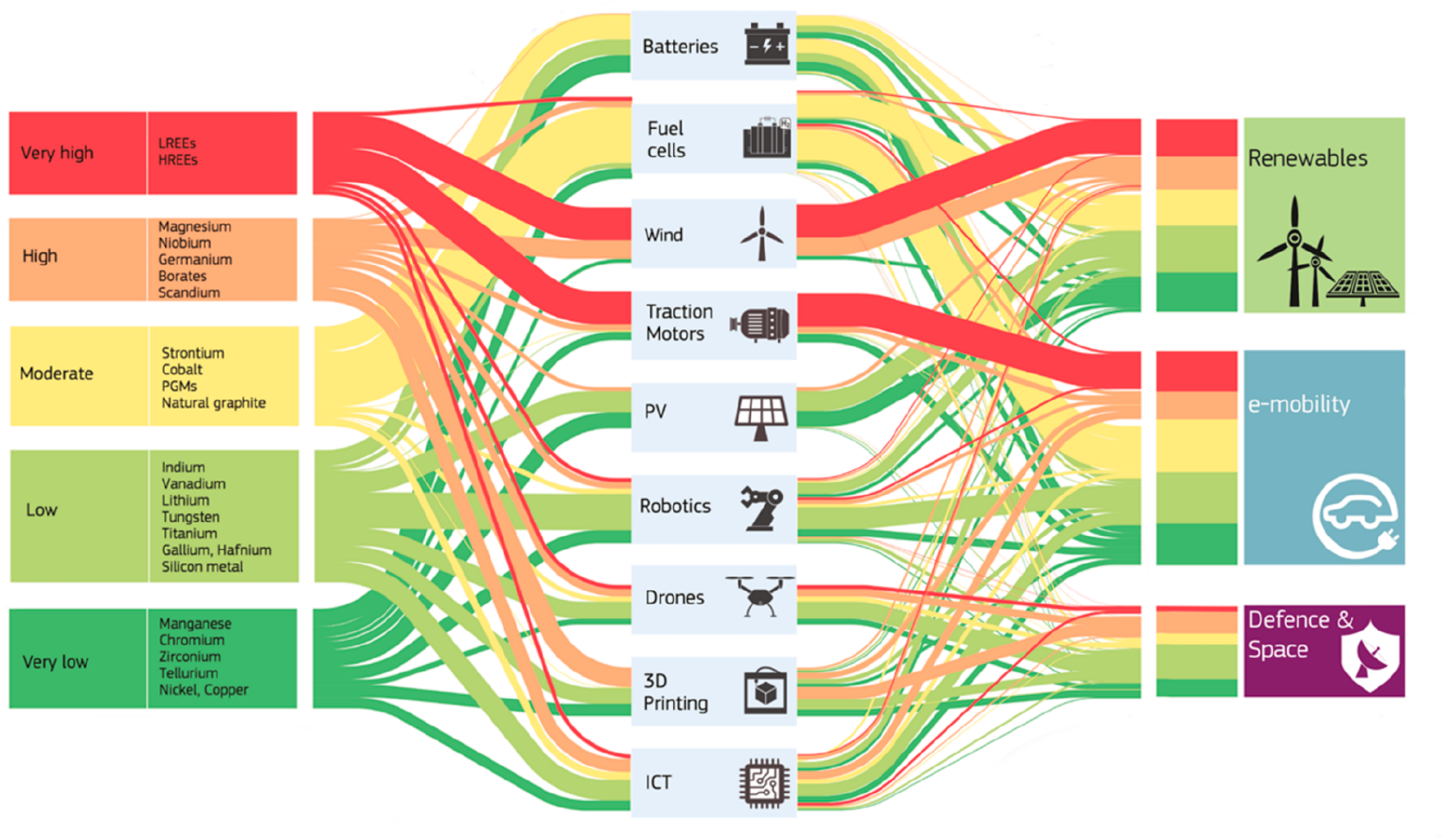
Overview in the form of a Sankey diagram of the main supply risks for some key metal groups (according to a EU 2020 criticality assessment) and other raw materials used in strategic value chains and strategically important industrial sectors in the EU.^44,45^ The figure has been reproduced with permission from the EU Open Data Portal (http://data.europa.eu/euodp). Copyright 2020, EU Open Data Portal. PV, photovoltaics; ICT, integrated circuit technology.

In this context the term “critical” or “strategic” metal is sometimes defined in the context of the element’s respective supply risk coupled with its (important) role for a region’s economy and security, but appears to be threatened by risk of supply disruption. This means that the terms “critical” and “strategic” refer to the uncertainty of obtaining sufficient amounts of a metal to meet certain future market demand projections. Factors responsible for such supply risks can be market volatility, excessive market growth, market speculation, geopolitical instability, natural disasters or political restrictions on mining and export. This can lead to price spikes and supply chain disruptions and limit the development of technologies that rely on these metals, Figure 4. Examples for such metals are lithium, nickel and cobalt for batteries, copper for electrification and dysprosium, europium, and neodymium for hard magnetic materials.

### 1.3 Greenhouse Gas Emissions Associated with Primary Metal Production

**Figure 5:**
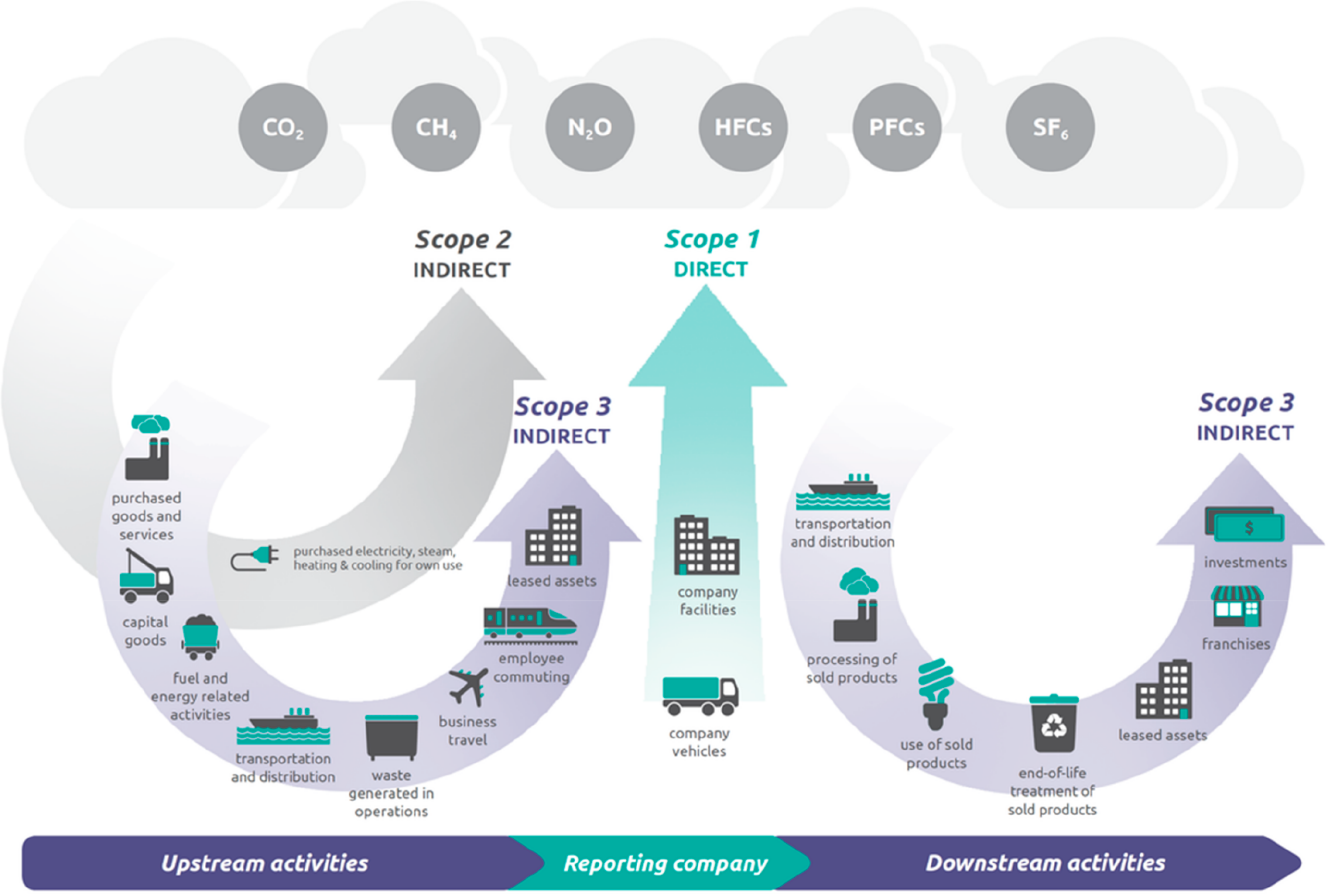
Scopes and emissions across the value chain. The image has been reproduced with permission from the World Economic Forum webpage (https://www.weforum.org/agenda/2022/09/scope-emissions-climate-greenhouse-business). Copyright 2021, World Economic Forum. HFCs, hydrofluorocarbons; PFCs, perfluorocarbons.

The term “greenhouse gas emissions” requires clarification, Figure 5. Usually 3 categories can be distinguished in corresponding life cycle assessments, Table 1. These categories help to identify, quantify and control emissions along manufacturing value chains. From a scientific perspective it must also be taken into account that there are very different types of greenhouse gases, with both a (a) different effect on the greenhouse effect and (b) different lifetime in the atmosphere, Table 2 (note that some of the lifetime values vary by more than a factor of 2 in the literature). Both aspects must always be taken into account in metallurgical sustainability.

**Table 1:** Categories of Greenhouse Gas Emissions

Scope	Control and responsibility for emission sources	Examples of emission sources
Scope 1 (direct emissions)	Emissions from sources controlled/owned by a company or organization; emissions released into the atmosphere through activities at the organization level; four types are distinguished: (I) stationary combustion, (II) mobile combustion, (III) fugitive emissions, (IV) process emissions	Scope 1 – type I: heating
		Scope 1 – type II: cars, trucks
		Scope 1 – type III: refrigeration, air conditioning
		Scope 1 – type IV: production of CO_2_ during steel production
Scope 2 (indirect emissions, owned)	Emissions released into the atmosphere, from the consumption of purchased energy supply, such as steam, electricity, heat and cooling	Electrical power purchased from a power plant/energy supplier
Scope 3 (indirect emissions, not owned)	Emissions released along the organization’s value chain, including upstream and downstream emissions, not included in scope 1 or 2, i.e. all other emissions linked to an organization’s operations	Employee commuting, business trips, emissions from disposed or incinerated waste, purchased goods and services

**Table 2:** Global Warming Potential and Atmospheric Lifetimes for Major Greenhouse Gases

Greenhouse gas	Chemical formula	Global warming potential, 100-year time horizon	Atmospheric lifetime (years)
Carbon dioxide	CO_2_	1	100
Methane	CH_4_	25	12
Nitrous oxide	N_2_O	298	114
Chlorofluorocarbon-12 (CFC-12)	CCl_2_F_2_	10,900	100
Hydrofluorocarbon-23 (HFC-23)	CHF_3_	14,800	270
Sulfur hexafluoride	SF_6_	22,800	3,200
Nitrogen trifluoride	NF_3_	17,200	740

Table 2 shows that the various forms of greenhouse gases can be divided into two groups, both of which are important when addressing metallurgical process emissions and mitigation strategies. The first one is the potential for global warming over a 100-year time horizon.^9,46^ The second factor is the gas’s atmospheric lifespan, which indicates how long each greenhouse gas stays in the atmosphere and contributes to global warming. Except for methane, which has a relatively short lifetime in the atmosphere of approximately 12 years, all of the major industrial greenhouse gases have a very long lifetime. This indicates that all of our current emissions will contribute to global warming over the next 100 years at the very least. This indicates that the ramifications of any metallurgical industry actions will outlast our generation. The second point to consider is that the different gases have varying degrees of impact on global warming. The reason for the special attention on CO_2_ and CH_4_ reduction in the field of sustainable metallurgy is obvious: these greenhouse gas emissions are by far the largest ones in comparison to other gas emissions, which have a considerably bigger influence on global warming but are emitted in much smaller quantities.

The largest amounts of these greenhouse gases, particularly the CO_2_, emitted in the metallurgical sector come from primary extraction and refining (i.e., synthesis), where oxidic (and to a lesser extent carbonatic and sulfidic) ores are exposed to reductants of fossil origin, mostly coke, or reduced via electrolysis, for instance Al, often using electrical energy of fossil origin.

**Figure 6:**
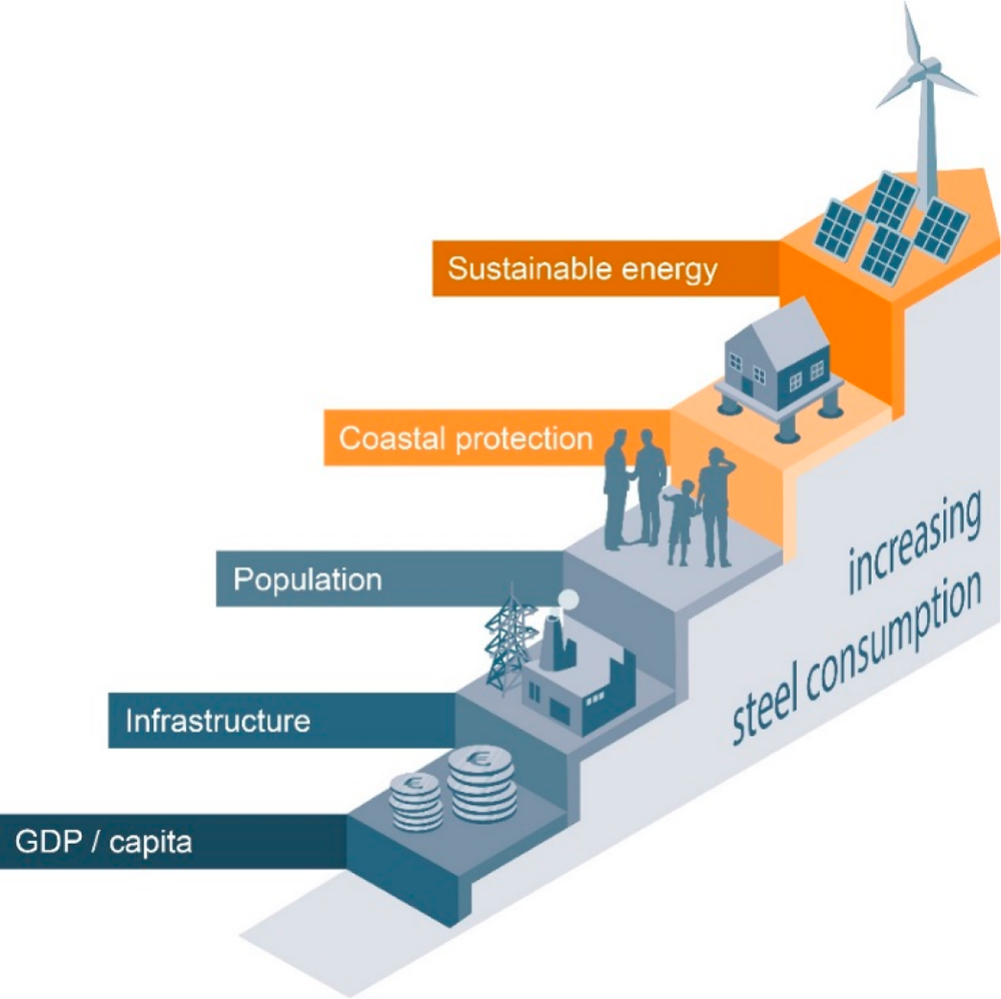
Main factors that fuel the global acceleration in the consumption of metals and their effects on the growing CO_2_ emissions. The figure uses trends for steel as an example material because it is the largest single industrial contributor to global warming through its massive CO_2_ emissions which primarily stem from the use of fossil reductants in blast furnaces, a route which stands for about 70% of the global steel production.^49^ GDP: gross domestic product, a macroeconomic indicator reflecting the monetary worth of all finished goods and services produced in a region over a given time period. The GDP scales in particular with the per capita (i.e., per person) consumption of metals.

**Figure 7:**
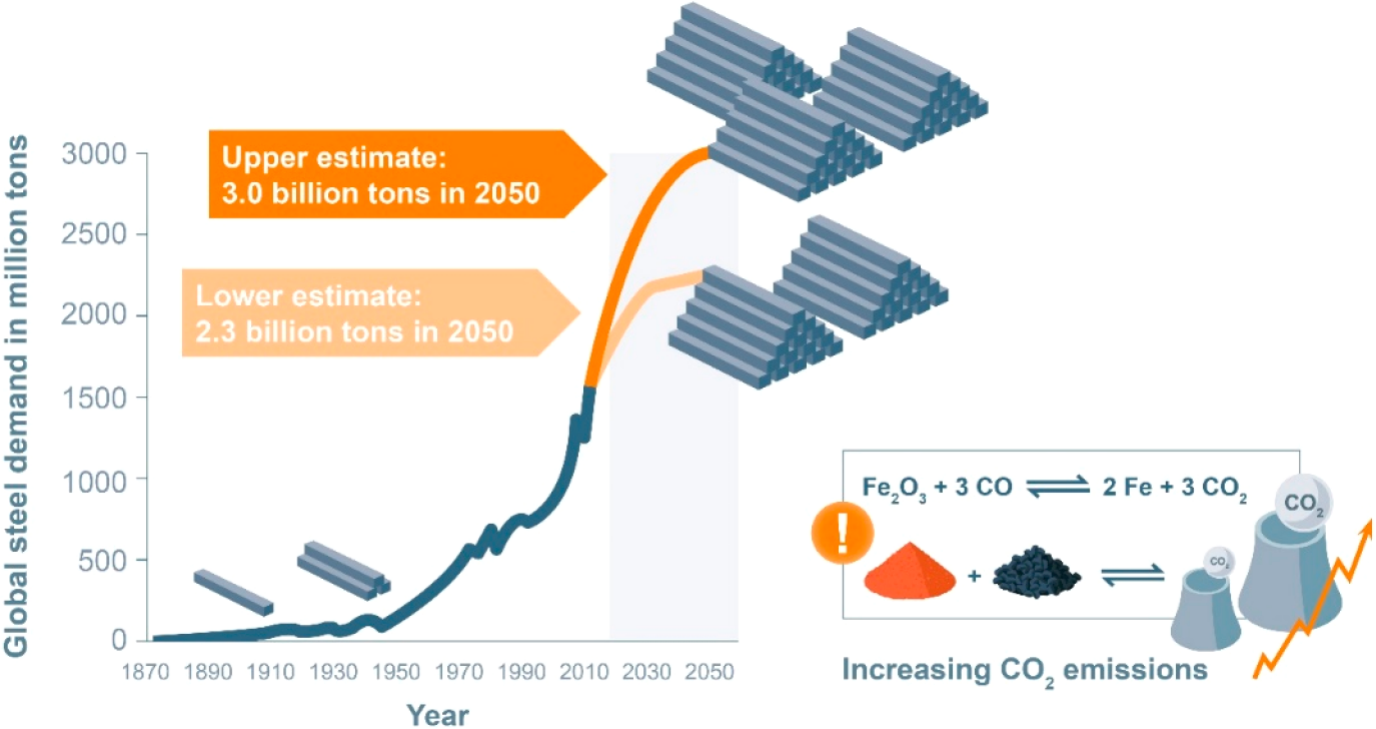
Market growth projections for steel (showing upper bound and lower bound estimates) using numbers from ref (50) and the net redox equation which explains the massive CO_2_ emissions associated with the carbon-based reduction of iron oxide ores.

**Figure 8:**
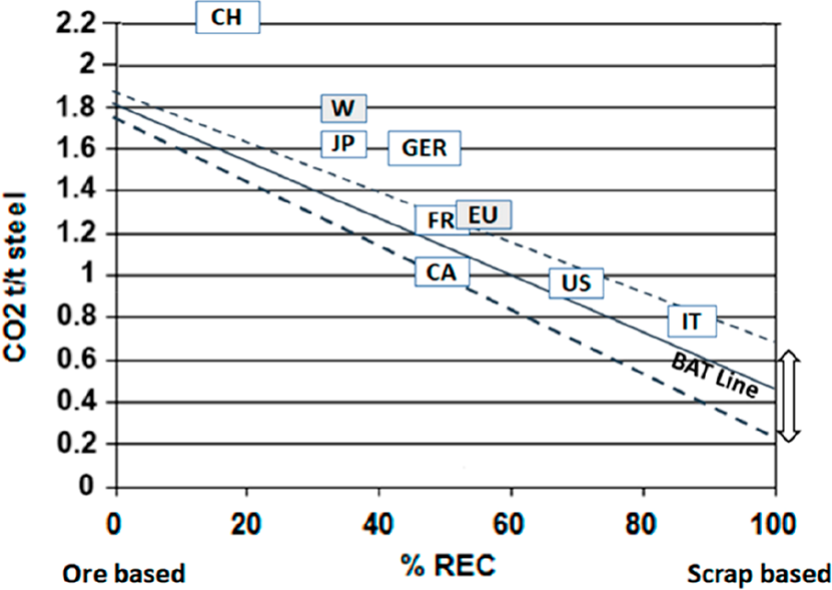
Dependence of CO_2_ emissions in steel making on the fraction of scrap used (referred to on the axis as % REC).^49^ BAT: Best Available Technology. The BAT reference line refers to the use of those existing techniques for the prevention of the CO_2_ emissions in steel production, which are developed at a scale that enables implementation under economically and technically viable conditions. JP, Japan; EU, European Union; GER, Germany; FR, France; CA, Canada; US, United States; IT, Italy; CH, China; W, World (global average, with about 35% of the steel coming from recycling). The figure is reproduced with permission from ref (49) under an open access Creative Commons CC BY license. Copyright 2020, MDPI.

As an example, Figure 6–Figure 8 and Table 3 show some statistics concerning steel, with its emissions accounting for around one-third of all CO_2_ emissions from industry (see details in section 7.4.1). The data show that in metallurgy size matters: the market for steels (like for most other metals) is growing and not shrinking; i.e., the accompanying environmental problems are not stagnant, but they will become larger, at a rapid pace, Figure 7. The data unmistakably demonstrate the enormous impact that research on synthesis processes that emit less CO_2_ would have in this industry. The quest to find ways to reduce greenhouse gas emissions through more environmentally friendly industrial processes qualifies metallurgy as one of the most rewarding academic study subjects, with very high potential leverage to combat global warming.^31,47−49^

**Table 3:** Carbon Footprint of Important Materials in Comparison: Magnitude of Emissions for Several Important Material Classes^a^

Material	Global CO_2_ emissions associated with production in 2017 (Gt CO_2_ per year)	Current global average specific CO_2_ intensity (tonne CO_2_ per tonne of metal produced)
**Steel**	3.7	2.00
**Aluminum**	1.0	14.40
**Nickel, cobalt**	0.01	20.00
**Petrochemicals**	1.5	1.70
**Cement**	2.2	0.86

a The data have been taken from Daehn et al.^31^.

### 1.4 Recycling, Downcycling and Upcycling of Metals and Alloys

**Figure 9:**
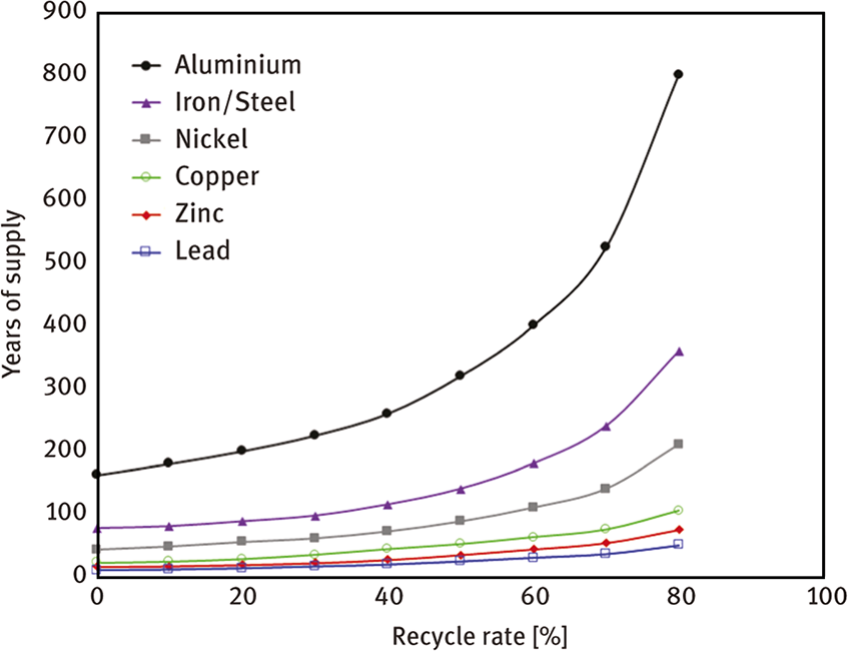
Effect of the recycling rate on the future supply of a few key metals.^69^ The figure is reproduced in modified form with permission from ref (69). Copyright 2002, Australian Institute of Mining and Metallurgy.

A first impulse to react to this (growing) emission scenario of the metallurgical sector could be to transform its linear parts (based on new mineral input, use of non-sustainable fossil feedstock as reductant for primary synthesis, and dumping of the waste products) into circular ones (making alloys from scrap instead, called secondary synthesis) (see details in section 6.3). This is exemplarily shown for the case of steel in Figure 6.^2,7,51−53^ Such a transition could reduce emissions, waste, by-products and energy consumption in part by up to 70% (steels) or even 90% (aluminum alloys).^7,54,55^ Also, drastically increasing the recycling rate would simply increase the availability of metals, as some of the metal supplies would become otherwise more and more limited, when only taken from mineral resources, Figure 9. A third option could be to develop re-integrative market elements, also referred to as re-mining, where old deposited industry waste is retuned back into the recycling stream and used as feedstock.^56−58^ The forth approach is to reuse and repair parts instead of scrapping them,^27,59−65^Figure 11.

The use of the term “recycling” in the metallurgical sector requires some refinement: in conventional recycling, products are re-introduced into the processing cycle from which they once left. In this respect, waste products are reprocessed and transformed into new raw materials, thus acquiring a new use and re-entering the cycle. However, it must be understood in that context that this is by no means a one-to-one reuse of the same amount of material but the recycling processes itself can have in part substantial losses and requires multiple types of resources to bring the material back in a state where it can serve in new products. This means that during recycling, which is an industry branch of its own, also material losses, energy consumption and greenhouse gas emissions apply, depending on the specific recycling processes that are used. All these aspects must be considered in corresponding life cycle assessments when comparing recycled materials with those stemming from primary synthesis, Figure 9. In most cases, however, all these emissions and losses are in secondary metallurgy indeed much smaller than those in primary synthesis.

**Figure 10:**
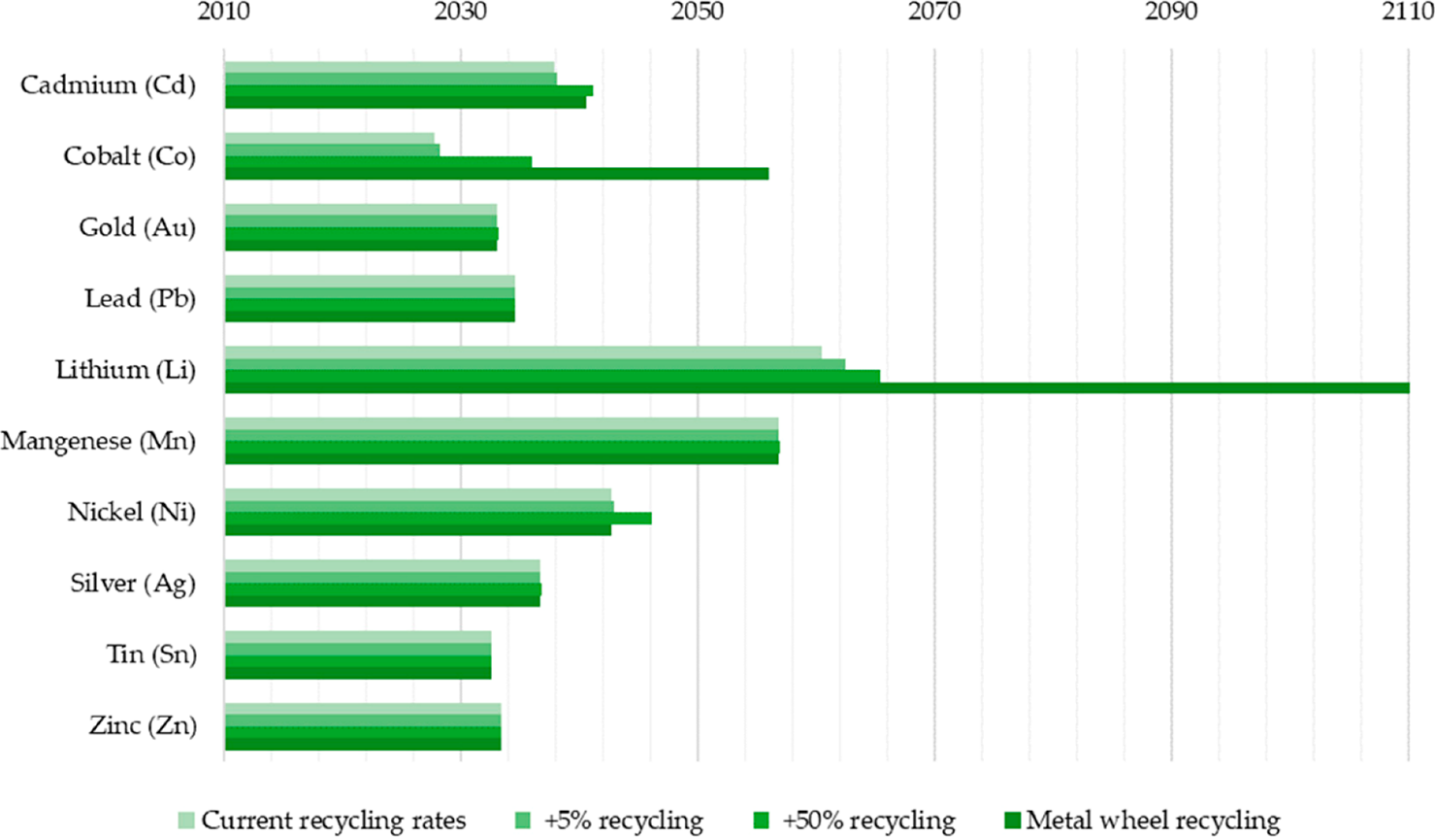
Influence of recycling rates for a few metals that are particularly critical for realizing sustainable energy supply and transport solutions in terms of the respective depletion horizons based on reserves for four different recycling scenarios according to Moreau et al.^66^ The term “metal wheel recycling” means that both the carrier or base elements of a metallic alloy are recovered but also the minor elements (doping or alloying elements), which often involves the use of staggered recycling approaches and different techniques of extractive metallurgy. The figure has been reproduced with permission from ref (66) under an open access Creative Commons CC BY 4.0 license. Copyright 2019, MDPI.

Figure 10 shows the effect of recycling rates for a few key metals that are particularly important for future sustainable energy supply and transport. The data show the respective depletion horizons based on reserves for four different recycling scenarios according to Moreau et al.^66^ The different recycling scenarios considered in their analysis are: (1) the current recycling rates for the metals considered remain unchanged until the year 2050; (2) 5% increase in recycling rates between now and 2050; (3) 50% increase in recycling rates (not very likely to happen); and (4) specialty metals are recycled at the current rates of their parent metals, which are assumed to remain the same until 2050 (as in scenario 1), according to the “wheel of metals” by product, according to the classification scheme suggested by Reuter et al.^21,67^

In contrast to downcycling, in standard recycled metallurgical products the quality of the product should not be diminished when using materials from recycling processes. The process of downcycling is the transformation of a product into one of lower quality. In this process, waste products can be used for the synthesis of less pure and less valuable products or they can be broken down into more basic chemical components, that are then mixed with new materials and imported back into the cycle in a completely new yet lower-value form. This process often involves considerable energy costs, and the often dispersed materials have to be transported over long distances. Despite all this, downcycling is no less important than upcycling, because recovery and reuse are still preferable to disposal. The downcycling process brings the metal back into a product of lower added value; i.e., the intrinsic high embodied values of the metal are diminished in downcycling. A typical example is the downcycling of titanium chips from machining of parts for aerospace applications into titanium oxide, a low-price product which is used, for instance, for wall-painting.

The stark opposite of downcycling is reprocessing in the form of upcycling: Here, the waste products undergo material upgrading and thus attain a higher value than before. The raw material undergoes little change and remains relatively true to its original form, saving energy and achieving a high degree of sustainability. The upcycling process brings the metal back into a product of higher added value; i.e., the intrinsic high embodied value of the metal is even further enhanced. For the metallurgical sector, the question arises whether there are even opportunities where metals can be brought together in such a way that they can be used in higher-value-added products. This research direction has been comparatively little pursued to date and holds great opportunities for basic research. A recent example in metallurgy is a newly developed solid-state electrolysis process for upcycling aluminum scrap.^68^ Details about the scientific aspects behind recycling via scrap melting are addressed in section 6.3.

### 1.5 Linear, Circular, Re-mining and Reuse Economy Models for Metals

**Figure 11:**
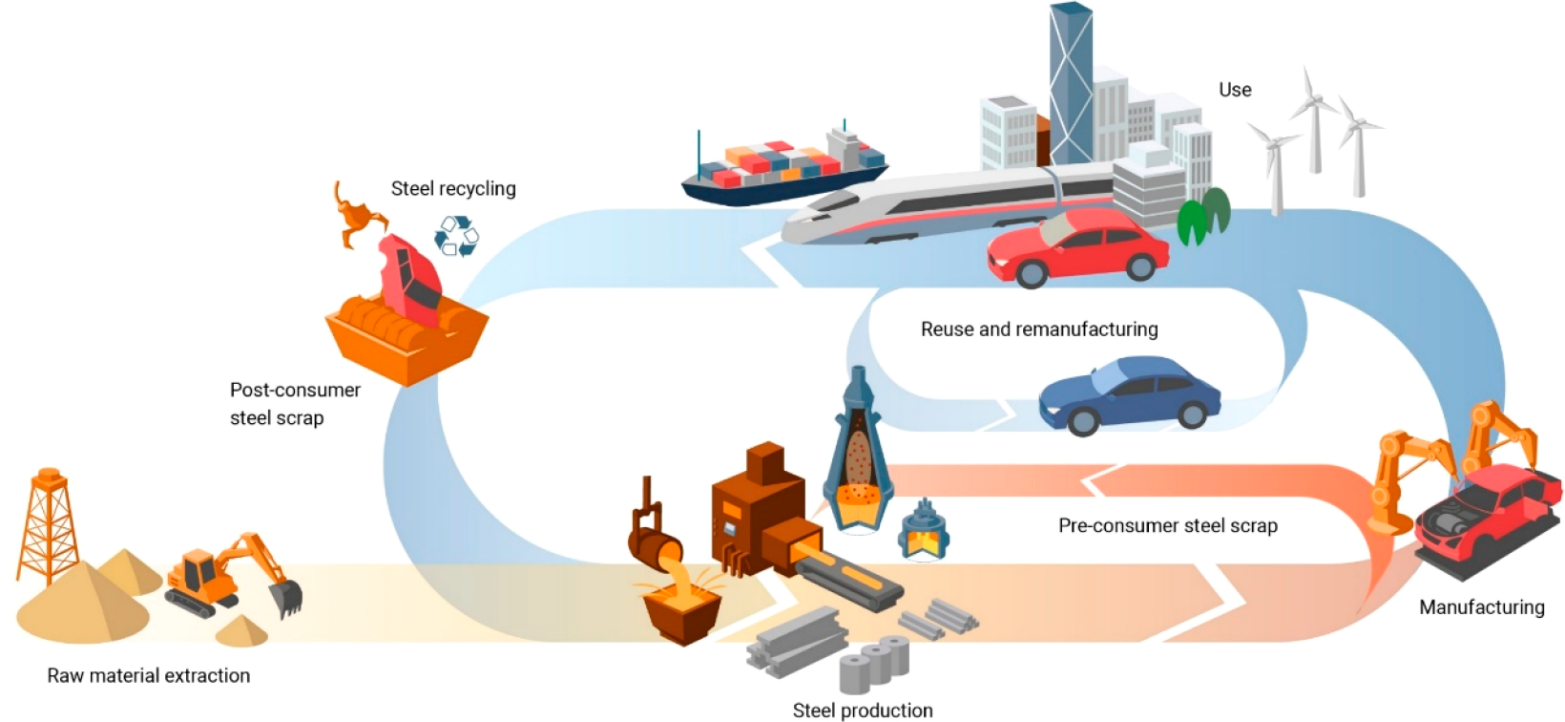
Some streams for old scrap (contaminated post-consumer scrap) and new scrap (runaround and in-production scrap with certified chemical composition), shown here for the case of steel.

Several classification schemes for approaches to reduce the need for primary synthesis of metals, hence reducing CO_2_ emissions, have been discussed in the literature. Examples are the so-called 3R, 4R and 6R models, Table 4 and Figure 11–Figure 13. 3R stands for reuse, reduce, and recycle; 4R refers to reduce, reuse, recycle, and recover; and 6R means reduce, reuse, recycle, recover, redesign, and remanufacture.^15,21,67^ These approaches especially address the more responsible use of critical and rare metals used, for instance, in catalysts or magnets, where the recycling rate today is in part below 1%,^3,11,70^Figure 14. The specific scientific challenges in recycling some of the important metals are discussed below in more detail.

**Table 4:** Linear, Circular, Re-integrative (Re-mining), and Reuse Economy Models for Metals^a^

Metallurgical economy model	Synthesis type	Feedstock	Total metal volume on the market	Waste volume on the market	Market volume
**Linear**	Primary	Ores, mined minerals, reductants (carbon-containing or carbon-free ones)	Growing	Growing	2/3 (Fe)
					2/3 (Al)
**Circular**	Secondary	Scrap	Constant	Constant or moderately growing	1/3 (Fe)
					1/3 (Al)
**Re-integrative (re-mining)**	Tertiary/re-mined	Deposited industry waste that is still rich in metallic element content (e.g., red mud)	Growing (from re-integration of waste as feedstock instead of from ores)	Shrinking	<1%
**Reuse, repair, reduce, redesign, remanufacture**	No	Part repair, reuse, redesign and reassignment	Constant	Constant	<1%

a Currently about two thirds of the metallurgical mass market, which also stand for the largest greenhouse gas emissions (i.e. steel and aluminium alloys), are linear, while only one third comes from scrap. Re-mining is today done only to a very small extent.

**Figure 12:**
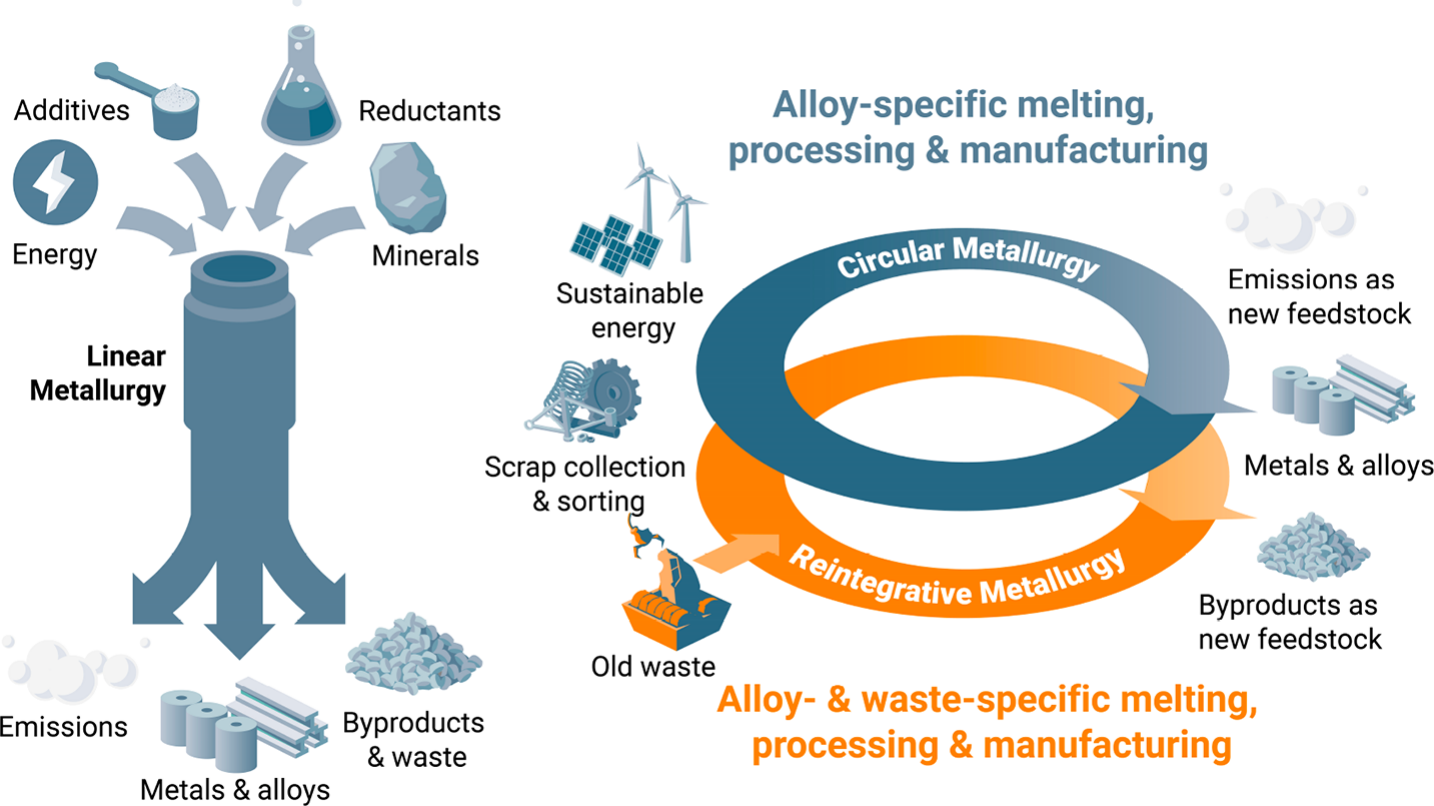
Schematic presentation of the main economic market models in the metallurgical sector: linear, circular and re-integrative aspects of the respective economy models. The latter term “re-integrative” refers to an approach where dumped waste material is “re-mined” and fed back into the manufacturing chain. An example is the extraction of metals from dumped red mud waste, a residue from bauxite refinement into alumina (see details in sections 6.2.6 and 6.4.2).

**Figure 13:**
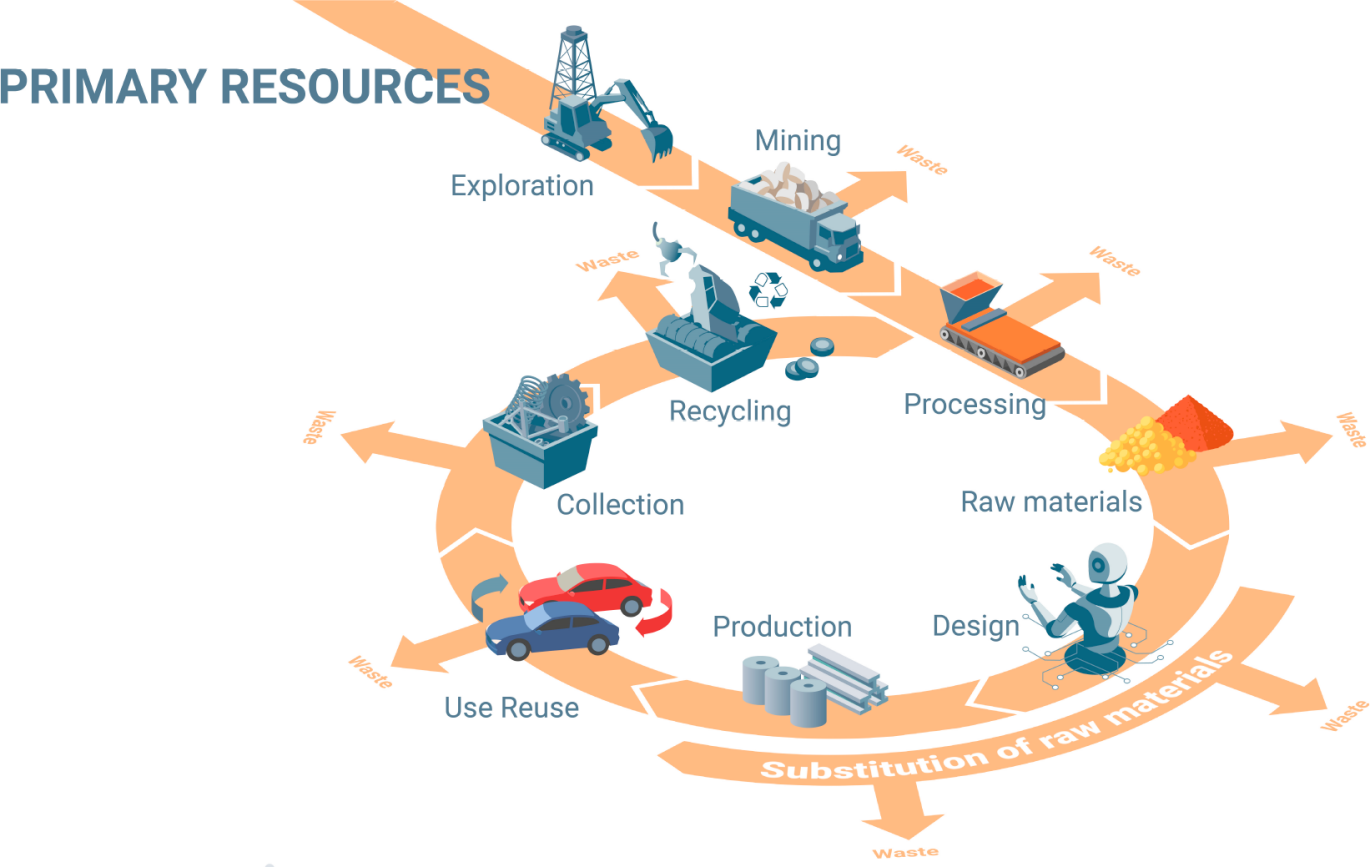
Schematic sketch of material losses on the way toward a more circular metallurgical economy.

**Figure 14:**
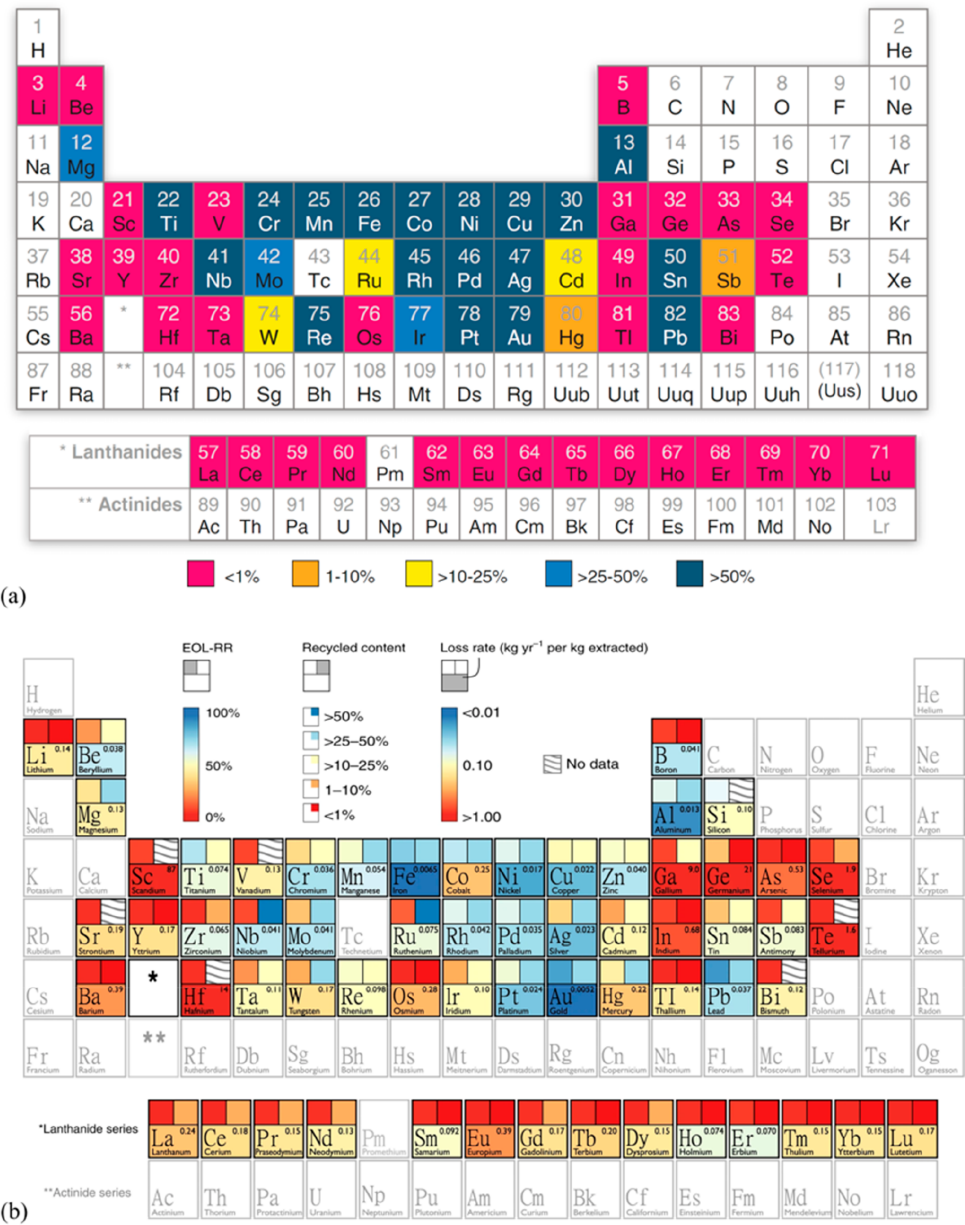
(a) End of life recycling rates^11^ and (b) metal loss rates for metals.^26^ The figures have been reproduced with permission. (a): Copyright 2011, Wiley-Blackwell. (b): Copyright 2022 Nature Publishing Group.

The short important message from these categories is as follows: (a) Primary synthesis principally causes by far the largest burden in terms of emissions, energy use, minerals and soil moved, and waste created; i.e., it is the most harmful of all synthesis approaches. Research in this field must be strictly directed at reducing the staggering CO_2_ emissions associated with the many different primary benefication and synthesis pathways. (b) Secondary synthesis via recycling of scrap metal is currently the most efficient, fastest to realize, and largest-scale option toward a more circular economy,^7,52,71−73^Figure 12. However, making new metal products preferably via recycling is limited (i) by the global availability of scrap (scrap is therefore sometimes also called the “oil” of the age of sustainability) and (ii) by mutual “poisoning” of alloys when mixed scraps are used.^73−77^ The latter scenario, i.e. the easier availability of mixed post-consumer scrap vs clean and well sorted in-production scrap, is actually the rule and biggest challenge in this field and not the exception, Figures 11−13. (c) Tertiary synthesis proceeds by the re-mining of already dumped waste materials that can serve as new feedstock for metal recovery (or as reductants etc.).^21,56−58,78−81^ This is a currently highly underdeveloped branch in metallurgy although it is the only approach that can potentially help to effectively reduce (rather than further grow) the huge existing waste burden. This means that re-mining is a negative-growth economy model for the mass balance in the metal sector that needs to be explored more. An example is emerging research on the recovery of both mass-produced and rare metals from red mud, which is a 4 billion ton dumped by-product when extracting Al_2_O_3_ from bauxite mineral mixtures^82^ (see details in sections 6.4.2 and 6.4.5).

**Figure 15:**
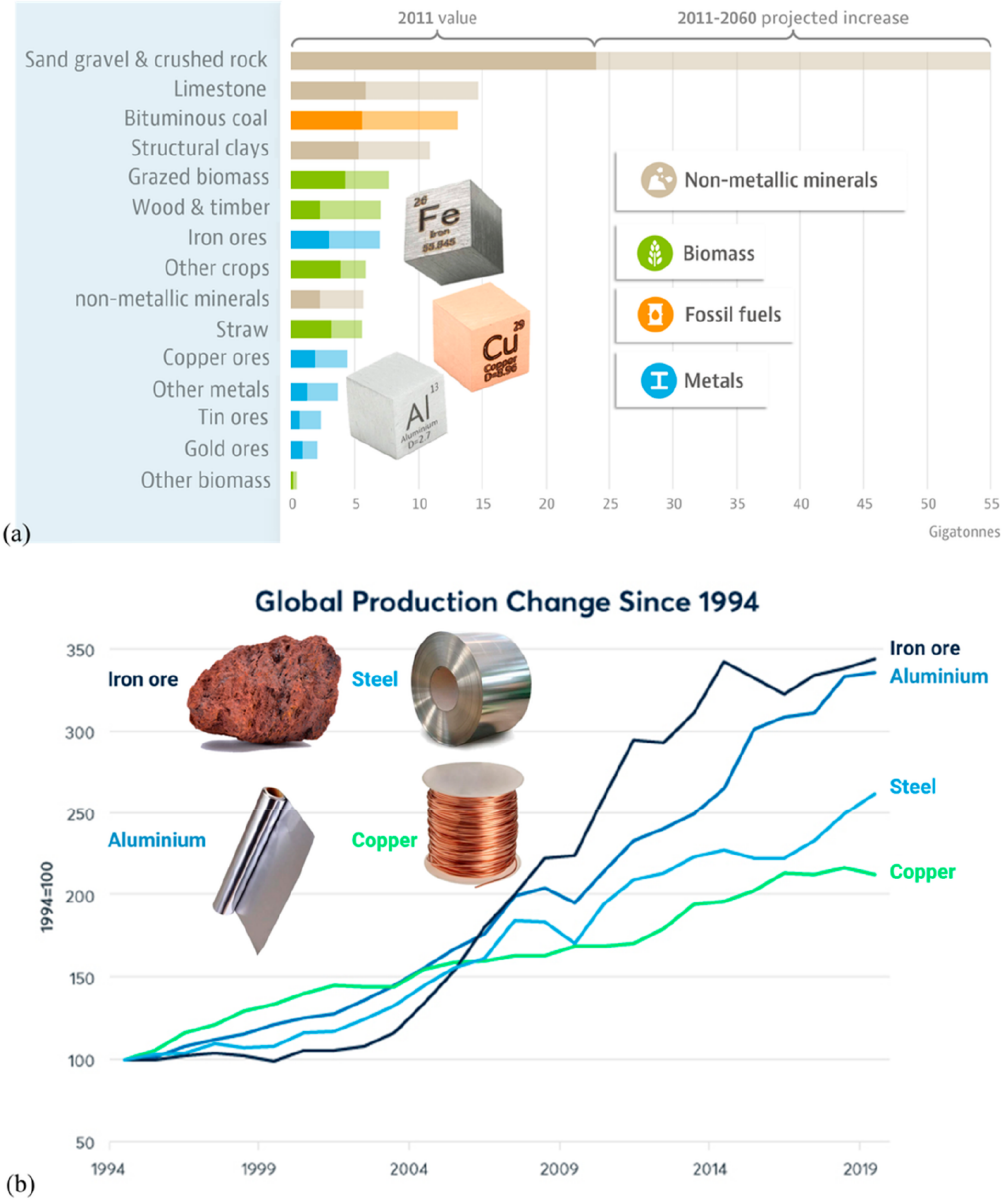
(a) An OECD forecast suggests that consumption of most raw materials will double by 2060. This means that the metallurgical (and generally the materials) sector cannot be exclusively built on circular economy principles, simply because there is not enough scrap and waste material available to fuel this strong market growth during the next decades (in which massive reduction in CO_2_ emissions is particularly critical). Therefore, sustainability measures must be identified, researched, and implemented which encompass all facets of the currently prevalent linear production, i.e. the primary metallurgical synthesis with its huge greenhouse gas emissions.^5^ (b) Changes in the amounts of metals produced over the last decades, with growth rates exceeding in part 300%. OECD: Organization for Economic Co-operation and Development. The images have been reproduced (part (a) has been modified) with permission from the OECD publication.^5^ Copyright 2019, OECD (https://www.oecd.org/termsandconditions).

For mass-produced alloys such as steel and aluminum, which contribute by far the largest fraction of greenhouse gas emissions, pollution, by-products, waste, and energy consumption (but also for most other metals), there is a fundamental limit to reuse and recycling, Table 3. This limit comes naturally from the massive growth in global market demand, Figure 6 and Figure 15, but also from rebound effects and dissipation.^4,38,39,50,69^ This means that establishing the metallurgical sector for these materials entirely on a circular approach is not possible, at least not until about 2060, as the markets for most metals, particularly for steel, aluminum, copper, nickel, cobalt, and lithium, grow much faster than the availability of scrap.^28,29,83,84^ The circular economy approach describes a concept for closing material loops, which means that the material recovered from waste must also serve as feedstock for new synthesis. In other words, every atom that is used in products and services must re-enter the production cycle when it is scrapped, minus the fraction lost due to dissipation.

The enormous market growth which stands against a fully circular metallurgical sector, at least during the coming decades, is particularly driven by the increase in global population and the average growth in per capita consumption of metals, Figure 6. Recent OECD estimates^5^ show that the demand for many metals will actually double by the year 2060, Figure 15. Besides this vehement pull from market growth, the second law of thermodynamics also teaches that any production, also a circular one, has entropic losses.

**Figure 16:**
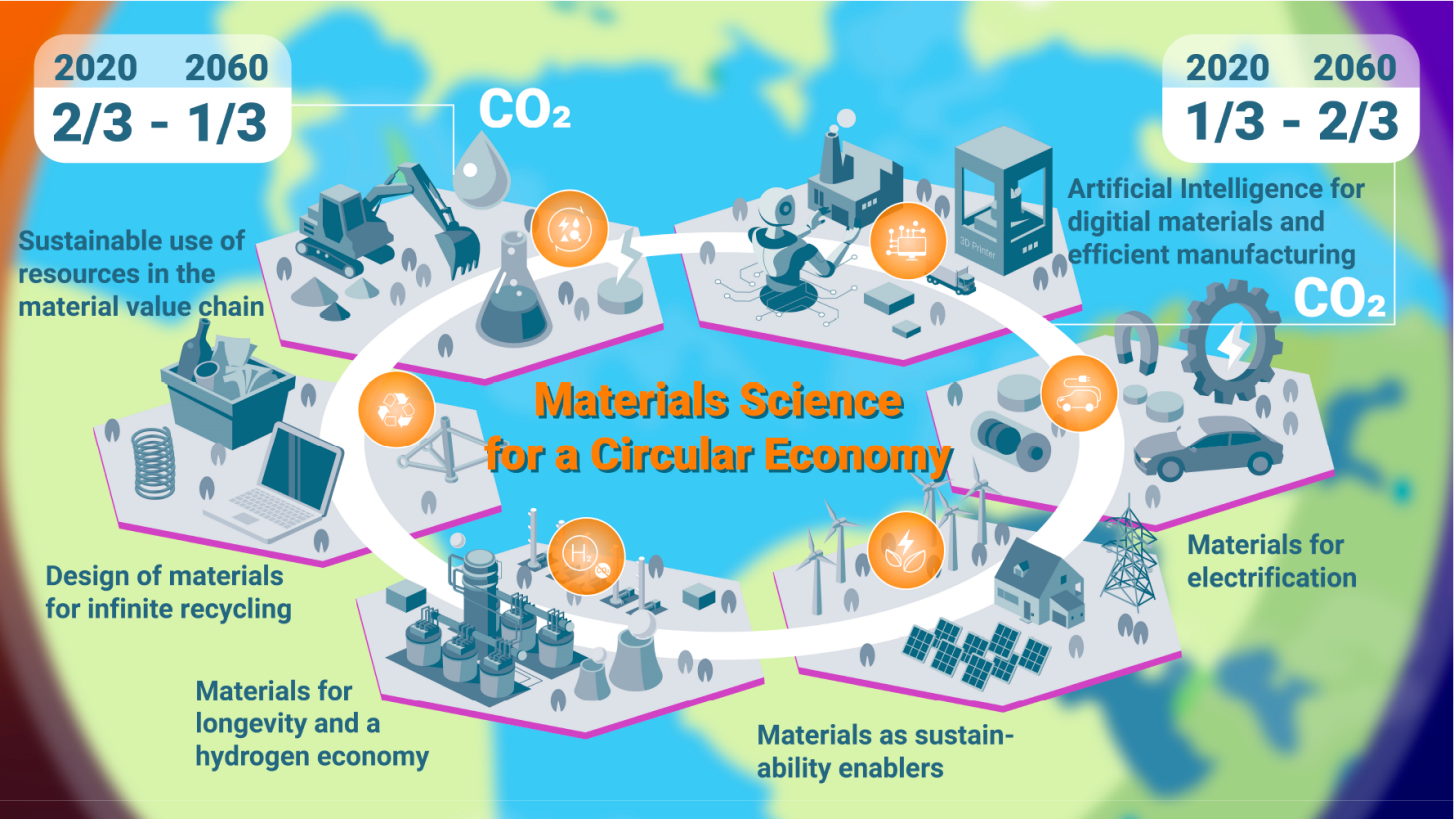
Even in a best-case scenario—by the year 2060—only two-thirds of the greenhouse-gas-intensive mass market for metals (iron, aluminum, nickel, titanium) will be circular in average and at least one-third will remain linear (i.e., based on mining, refining and primary reduction) for several decades to come. This means that—simply due to high demand growth—at least one-third of the metal mass markets will also in the future have to be provided by primary synthesis, which creates massive greenhouse gas emissions. Today the situation is actually opposite when viewed at a global average; i.e., only about one-third of the total metal mass market is served from circular production (through melting scrap) while two-thirds are linear, coming from newly mined feedstock. This huge fraction of primary synthesis is the biggest single source of global warming and must therefore be addressed by research with high priority. The figure also refers to a few other high-leverage measures for increasing the sustainability of the metallurgical sector.^2^

Therefore, during the next decades, where emission reduction is essential, even under optimal conditions, only about two-thirds of the metallurgical mass market can be circular and at least one-third will remain linear for several decades to come (until about 2060). This is a best-case scenario. The reality—particularly for those metals that stand for the largest market and emission volume—is actually opposite; i.e., only about one-third of the market is served from circular production (through melting scrap) while two-thirds is linear, coming from newly mined feedstock, to serve the growing market, Figure 16. Under less optimal conditions, the circular fraction can be even much smaller.

This discrepancy of metal demand growth and insufficient scrap supply means that massive amounts of minerals are needed as new feedstock, to feed primary synthesis.^5,19,37,42,85^ Yet, this step produces by far the largest emissions and energy demand, if no sustainable primary synthesis (refining and reduction) methods are identified, Figure 1. This means that under the current predominantly fossil-based production conditions we do not talk about a massive reduction in emissions and energy consumption in the metallurgical sector, as would be desirable to match the targets of the Paris Agreement, but only about the reduction of their further increase.

**Figure 17:**
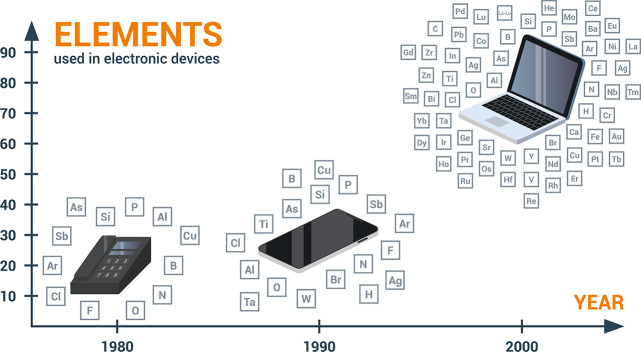
Change in the number of elements used in consumer electronics over the years. An analysis of the number of chemical elements over a wide range of such products was presented for instance by Christian et al.^91^ See details for example in section 7.6.4.

When reviewing the engineering and economic competition between circular, linear, reuse and re-integrative (based on re-mining) metallurgical production, it must be considered that recycling of metals and the use of waste material is not per se a clean technology, but it is also accompanied by multiple harmful and polluting effects which must be billed in.^3,8,55,73,86−88^ This means that research into sustainable metallurgy must target the development of such recycling techniques which are sustainable in themselves; otherwise, they might harm the environment in some cases even more than linear production methods (see details in section 6.3). A negative example is the use of highly scattered or coated thin film post-consumer packaging aluminum for secondary synthesis versus primary synthesis of aluminum by the use of sustainable (e.g., hydropower) electrolysis. Several authors^11,19^ have therefore introduced the notion of “high-quality recycling”, where these aspects are considered. One specific challenge behind that, particularly for the precious metal sector and the huge multi-metal recycling problems encountered in the recovery of elements from electrical and electronic waste (containing up to 60 elements, e.g. in a modern notebook computer or smartphones), is the mutual poisoning problem, Figure 17. This means that special efforts must be devoted to careful upstream sorting of scrap with respect to specifically detrimental (“poisoning”) elements before mixing them together. The reason is that some elements are mutually particularly detrimental for the downstream metallurgical separation of metals in multi-element waste streams (e.g., coming from cell phones).^89,90^ Another aspect related to the use of scrap is its dispersion. This refers to the problem that when scrap is too much scattered, the CO_2_ emissions associated with collecting and using it might be larger than the gain of finally using it as feedstock for secondary synthesis.

These aspects show that the entire metallurgical sector must be made more sustainable, considering all possible economic models, be they more circular or more linear in nature.

As briefly mentioned above, care should be taken in the metallurgical and mining sectors before declaring certain measures as being more or less sustainable, without consulting the results from corresponding evaluation methods. For the evaluation of the effectiveness of a sustainability measure, a number of assessment protocols have been developed. Examples are life cycle assessment, life cycle energy, global warming potential, acidification potential, materials intensity per unit of service, environmental impact assessment or ecological risk assessment, to name but a few of these tools, Table 5. Particularly the life cycle assessment method has gained global acceptance in this field, and it is widely used for the assessment of potential environmental impacts associated with a product, service, process, or related activity during its entire life cycle.^27,92^ It is therefore sometimes also referred to as “cradle-to-grave” assessment. A few useful software packages and workflows are available for conducting life cycle assessments, yet they are often equipped with limited data.

**Table 5:** Comparison of Some Life Cycle Assessment Tools, i.e. Life Cycle Energy, Global Warming Potential (GWP) and Acidification Potential (AP) for Various Metal Production Processes^a^^,^^b^

Metal	Process	Total energy (MJ/kg)	GWP^c^ (kg CO_2_e/kg)	AP^1^ (kg SO_2_e/kg)
Iron/steel	BF/BOF^d^	22	2.3	0.02
Aluminum	Electrolytic^e^	211	22.4	0.13
Copper	Pyrometallurgy^f^	33	3.3	0.04
	Hydrometallurgy^g^	64	6.2	0.05
Lead	BF^h^	20	2.1	0.02
	ISF^i^	32	3.2	0.02
Zinc	Electrolytic^j^	48	4.6	0.06
	ISF	36	3.3	0.03
Nickel	Pyrometallurgy^k^	114	11.4	0.13
	Hydrometallurgy^l^		16.1	0.07

a The table has been reproduced from the work of Norgate and Rankin.^69^.

b GWP: global warming potential. This quantity refers to the relative effect of different molecules to act as a greenhouse gas, considering also how long it remains active in the atmosphere. The GWPs currently used are those calculated over 100 years. Carbon dioxide is taken as the gas of reference and given a 100-year GWP of 1. AP: acidification potential. This quantity describes the extent to which different chemicals contribute to acid rain. In the context of metallurgy this considers in particular SO_2_, NO_*x*_, NO, N_2_O, and several other substances. ISF: imperial smelting furnace.

c Black coal-based electricity.

d Blast furnace (BF) and basic oxygen furnace (BOF) (iron ore, 64% Fe, 50% lump, 50% fines, open-cut mine).

e Bayer and Hall–Heroult processes (bauxite ore, 17.4% Al, open-cut mine).

f Matte smelting, converting and electro-refining (sulfide ore, 3.0% Cu, underground mine).

g Heap leaching, solvent extraction and electrowinning (sulfide ore, 2.0% Cu, underground mine).

h Blast furnace (ore 5.5% Pb, underground mine).

i Imperial smelting furnace (ore 5.5% Pb, underground mine).

j Roasting & electrolysis (ore 8.6% Zn, underground mine).

k Flash furnace smelting and Sherritt–Gordon refining (sulfide ore, 2.3% Ni, underground mine).

l Pressure acid leaching, solvent extraction and electrowinning (laterite ore, 1.0% Ni, underground mine).

### 1.6 Goals in Sustainable Metallurgy and Mission of This Paper

The analysis given in the preceding sections can be cast into a few more specific topical items. Along these lines, sustainable metallurgy can be defined as a holistic and systemic approach of producing metals in a way that balances engineering, economic, social, and environmental considerations. This approach can be grouped along a few main pillars, namely, environmental sustainability, economic viability, social fairness, resource efficiency, physical and chemical foundations of the required processes, and disruptive innovation strategies.^25,37,41,52,93−95^

Environmental sustainability refers to reducing the environmental impact of the entire metal production chain, with the most essential and urgent goals of reducing greenhouse gas emissions, minimizing water and energy use, and reducing waste production.

Economic viability includes producing metals in a way that is economically rewarding and profitable, while also ensuring that the underlying and downstream industries are themselves resilient and sustainable in the long term.

Social fairness refers to the sustainability of the consequences that the transition toward a more circular economy has on society, referring explicitly to the global society.^19,85,96^ This means that sustainable metallurgy includes the task of ensuring that the industry is socially responsible, by providing safe and healthy working conditions for employees, respecting the rights of local communities, and promoting fair labor practices. A socially responsible approach to sustainable metallurgy must ensure that sustainability gains in wealthy regions are not created by suffering in less wealthy regions of the globe. This means that it cannot work by exporting all the health risks and poor labor conditions associated with mining and production of the additional metals needed for a more sustainable technology infrastructure to low-wage regions. This would create a global imbalance where sustainability gains in rich parts of the world are bought at the costs of the suffering of poor parts of the world.

Resource efficiency means that the use of natural resources, such as water, energy, and raw materials, is minimized in the production and use of metals.

The last two pillars of sustainable metallurgy, namely, the scientific foundations of the processes involved and the many disruptive innovations needed to revolutionize this sector, are at the core of this paper. They refer to all basic and applied questions that help to render the entire metallurgical sector more sustainable, through recycling and closed-loop systems, less energy- and greenhouse-gas-intense primary production, waste minimization, re-mining, as well as the invention and maturation of new technologies, processes, and materials. All these items must be scalable to the huge dimensions and quantities in this field, characterized by the production of about 2 billion tonnes of metals every year. As a guideline through this paper, the later points can be grouped along a few main goals and research directions, where the focus is placed particularly on topics with high leverage on reducing CO_2_ emissions and energy consumption:

1. Sustainable primary production of metals and alloys. This includes sustainable synthesis from primary (minerals) and ternary resources (dumped industry waste that can be re-mined) as well as more efficient and energy-saving downstream production. In essence this encompasses all efforts to extract and process chemically bound metals from raw and waste materials with less greenhouse gas effects and at lower energy consumption. The huge amounts of waste and by-products from metal production must also be considered in this category.
2. Sustainable secondary production of metals and alloys by use of scrap. This includes better collection and sorting of scrap and its use for making recycled and even upcycled metallic alloys. It also includes research on improving recycling of intensely mixed scrap where element recovery is very challenging owing to their close integration in components. A related task is to change alloy design in a way to make materials compositionally more robust and thus better suited for recycling. This means that we must rethink alloys in a recycling-oriented way that they can better serve (a) as scrap-donator for a larger variety of new materials and (b) as scrap-acceptor from a larger variety of old materials. This means that alloys must become compositionally more streamlined and lean and that the chemical variety of metallic alloys should be reduced. This turns the entire field from chemistry-dominated alloy design to microstructure-dominated alloy design. Also, in general, alloys must become more impurity-tolerant.
3. Substitution of metallic alloys, i.e. replacing less sustainable metallic materials by more sustainable ones.
4. Increased longevity of metallic materials, to avoid the products made of them being scrapped in the first place.

Of course there are many more aspects to be considered in that context in each of these categories. Examples are discussions around the general reduction in the consumption of metals for capita and more profound changes in how we live and consume goods. However, these more societal facets are outside of the scope of this paper, which aims to take a scientific view at metallurgical measures for the fast and efficient reduction of greenhouse gas emissions in this sector and which are realistic and compatible with the expected global consumer behavior. Also, it has been shown that the growing market demand for metals scales with the increase in the gross domestic product and this is particularly driven by the growth of economies in highly populated and less wealthy regions of the globe who strive to escape from poverty. It seems hence not very realistic and not fair to expect that the populations in these regions abandon their right for economic prosperity. Furthermore, the hazardous influence of greenhouse gas emissions, energy demand, and waste and by-products from metal production on the planet’s future and its relationship to the global economy and societal boundary conditions have been addressed in detail in the literature.^4^ In contrast, the exploration and reflection of the scientific foundations of how to reduce all these effects by disruptive innovations in the metal sector have received much less attention.

Many of the currently discussed engineering and technological mitigation strategies to change this system appear often as linear extrapolations from well-established synthesis and processing concepts, and some of these concepts have a moderate effect on the improvement of the sustainability of metals, particularly on CO_2_ emissions. They are in part rather motivated by a gradual transition approach toward more sustainable technologies, where existing technologies are integrated rather than replaced. The reason is that synthesis and processing investments in the metallurgical sector are usually huge, sometimes of billion Euro dimension. This means that wrong investment decisions can substantially harm a company or even an entire industry sector. This is also one further motivation item for conducting more basic metallurgical research in sustainable metallurgy, as better understanding is the best guarantee for making pertinent and robust investment decisions with a long-term effect paired with economic viability. This means that developing more disruptive and innovative approaches in this field, at minimized investment risk, will profit from deeper understanding of the underlying governing scientific principles, allowing identification of key bottlenecks and fundamentally new approaches with high efficiency for a sustainable metallurgical system.

**Figure 18:**
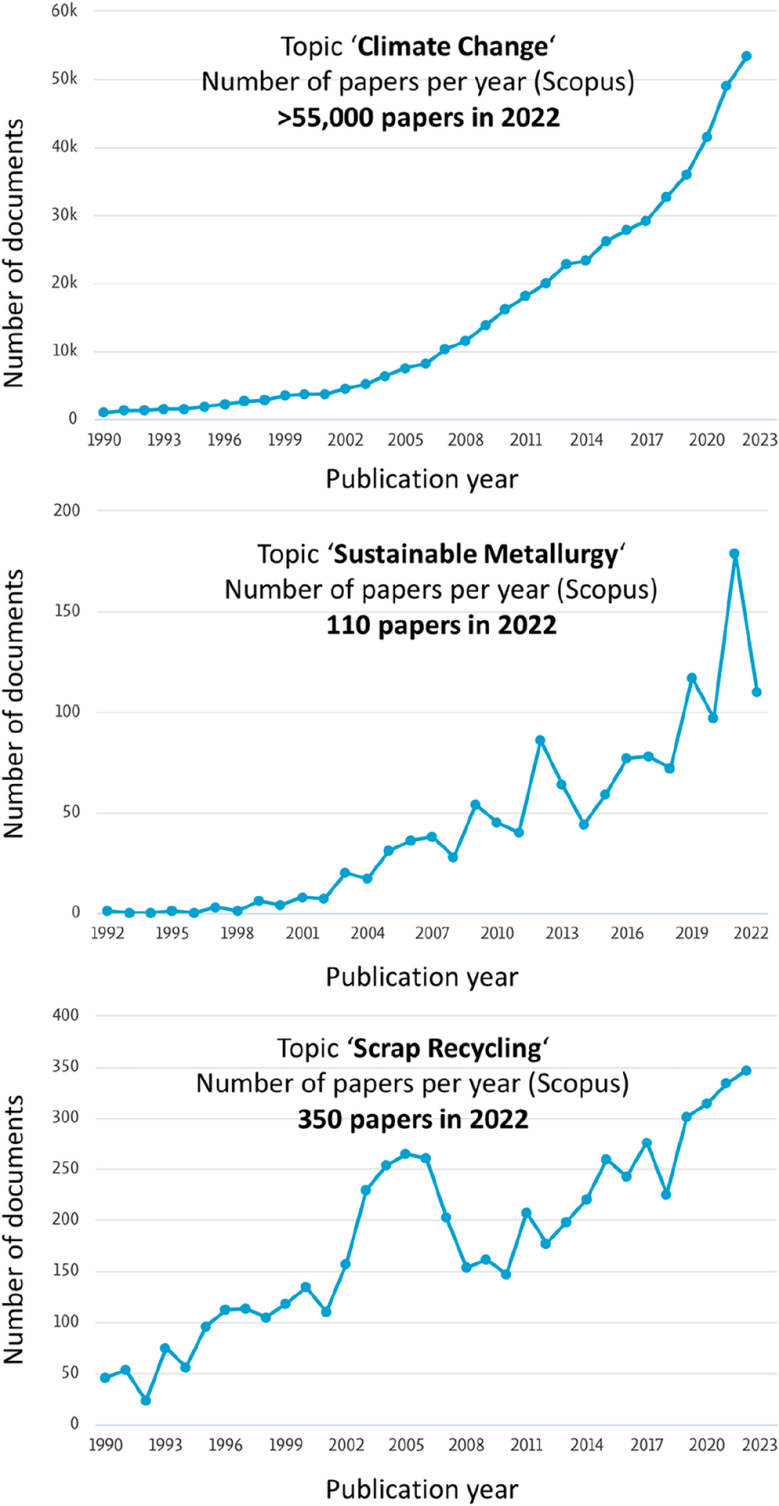
Publication records on climate change (propelled essentially by CO_2_ emissions) compared to records that deal with the science to make the metallurgical sector, as the largest contributor to global warming, more sustainable (data taken from the database “Scopus” between 1990 and 2022). The comparison reveals that more than 55 thousand scientific articles are published about the origin of climate change every year but only a few hundred papers deal with the urgently needed research about solutions to solve the problem and actually reduce CO_2_ emissions. This shows that there is a mismatch between basic research on the causes of climate change on the one hand and basic research about the mitigation of climate change on the other hand. Research on more sustainable materials could therefore become a key discipline in the future, owing to the huge leverage of material production on greenhouse gas emissions.

Yet, while the relationship between greenhouse gas emissions and global warming as well as the associated environmental crisis are meanwhile addressed by more than 55 thousand scientific publications per year, with a growing trend, only 200–500 papers per year address the basic science behind the quest to eliminate its biggest single cause, i.e. the greenhouse gas emissions from the metallurgical sector that are responsible for the climate change, Figure 18. This mismatch suggests to adjust the research focus in this field from a mere descriptive approach toward more solution-oriented thinking. This means that there is an urgent need to identify new approaches to actually reduce CO_2_ emissions rather than to only contemplate about their harmful effects. Yet, the fundamental materials research opportunities and challenges behind the question how to render metals more sustainable are surprisingly little addressed by the research community so far, Figure 18. This paper therefore tries to identify the most promising research topics in the metallurgical sciences, that can help to reduce emissions and not just describe their effects on the climate.

This disparity is probably not surprising, as the task to make metals and alloys more sustainable describes an urgent global challenge but it does not per se constitute a generic homogeneous research field. Instead, it consists of a wide array of scientifically quite different and often interdisciplinary research aspects, where quite different fundamental research questions emerge, some of which have little in common.

This becomes apparent when considering the quite different research challenges behind specific categories of metallurgical sustainability synthesis and recycling chains. Examples are categories such as mass-produced construction materials^17,97^ (e.g., used in buildings and vehicles) vs nanoscale integrated functional material systems^98^ (e.g., used in computer chips); the degree of dispersion of metallic elements in the scrap (i.e., large-volume scrap vs highly mixed nanoscrap, e.g. in microelectronic parts); the primary synthesis and recycling processes used (such as pyrometallurgy, hydrometallurgy, or electrometallurgy); the dilution vs richness of metals in minerals and their respective abundance and commercial accessibility; or differences in intrinsic properties among various metals and alloys (magnetic response, binding energies with oxygen, electrochemical nobility, mass density, etc.).

Also, alloys are usually “invisible” and “hide” inside products and technologies into which they are often very closely integrated (e.g., metals in mobile phones, computers, power plants, or household appliances). This makes it even more difficult, impossible, or even counterproductive from a sustainability standpoint to dismantle, retrieve, collect, separate, reuse, sort and recycle all of them. The reason for this is that the collection, sorting and recycling of metals, depending on their degree of dispersion in waste and scrap, can in some cases even generate more greenhouse gases and can have a higher energy consumption than if the same materials are produced via primary synthesis, i.e. from minerals. Such competing sustainability scenarios can be evaluated through life cycle assessments,^57,99−104^ which, however, are not the subject of this article. In other words, measures that may appear sustainable do not necessarily have to really be sustainable.

The fact that the production numbers in the metal sector and the environmental harm caused by it have such a huge magnitude qualify metallurgical sustainability research as an important and urgent research topic, with significant leverage on the future of an entire industry. Solving fundamental questions in this field requires inclusion of methods from metallurgy, mechanical engineering, physics, manufacturing and chemistry. The scientific challenges but also the research opportunities in this field are enormous. This makes the topic appealing to a new generation of researchers with a highly interdisciplinary approach to materials science. The reward is to conduct research that has high impact and significance for a sustainable society and industry.

As indicated in Figure 16 the sustainability of metallic materials can be grouped into two main categories, direct and indirect sustainability.^2^ The former refers to all measures that help reduce the environmental burden associated with the synthesis and manufacturing of metals and alloys, i.e. primarily the reduction of their carbon, energy, and waste footprint associated with production. The latter refers to all sustainability effects that metals enable through their properties, when used in products or processes. This means that direct sustainability addresses the sustainability of metal production while indirect sustainability addresses sustainability gains through the use of metallic materials, Figure 3.

**Figure 19:**
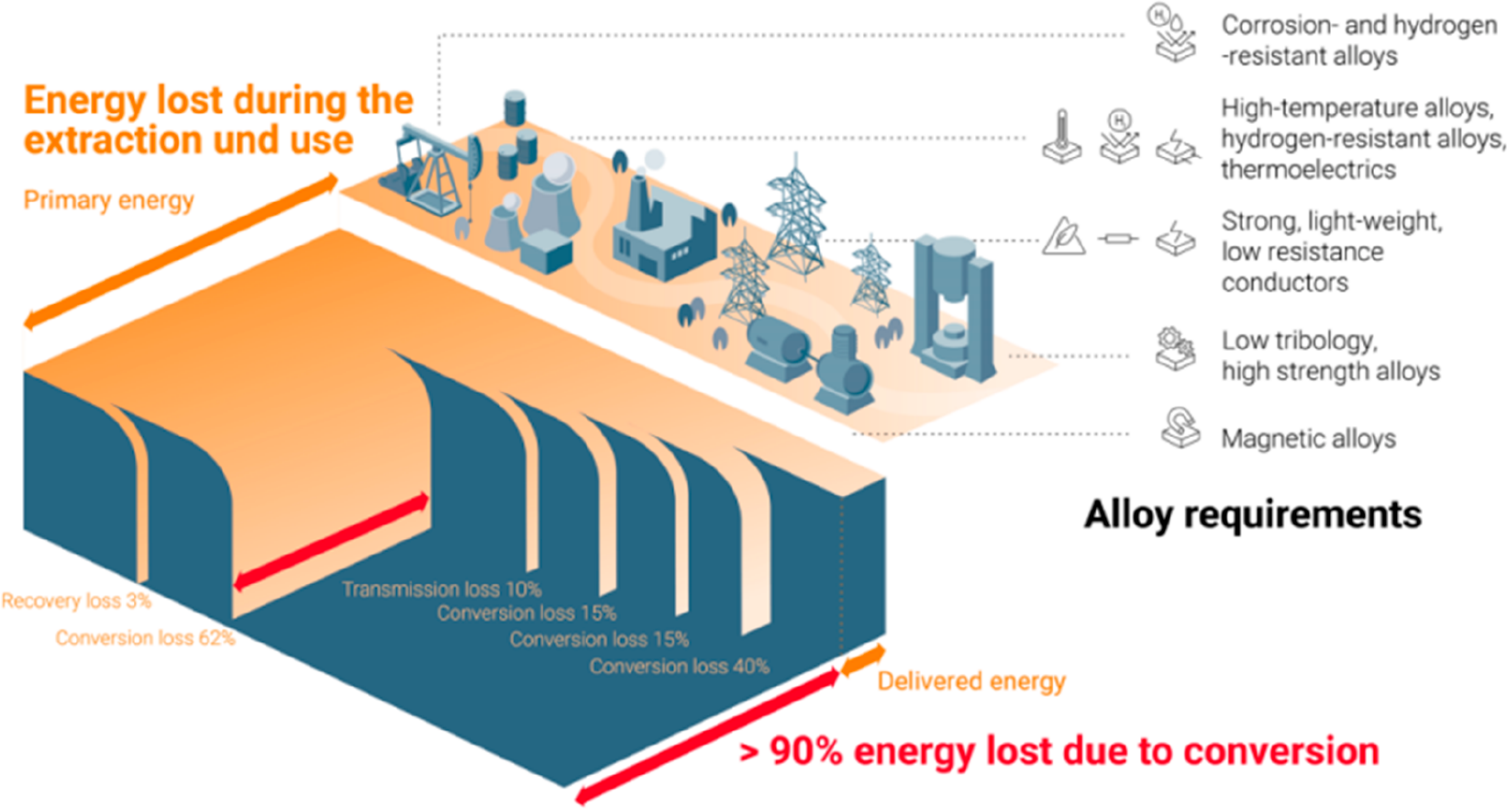
Example of indirect effects of metallic materials on sustainability. Global electricity is created today primarily from fossil energy carriers in thermal power plants. This power is then transmitted and distributed to the points of usage, where it is transformed to mechanical work and heating etc. More than 90% of the primary energy is lost during the conversion of the fossil fuel into usable forms of energy.

It should be underlined that this article is not about indirect sustainability of metallic materials,^2^ where metals serve through their properties, Figure 19. Such topics are frequently discussed in specific overviews and will therefore not be repeated here. Examples can be found in the wide body of literature and the many reviews on high strength steels; magnesium- and aluminum-alloys (to reduce vehicle weight); high-strength electrical conductors (to reduce resistive losses); soft and hard magnetic materials (for efficient electrical motors, transformers and magnetochaloric applications); thermoelectric materials (to harvest waste heat); creep resistant alloys (to increase Carnot efficiency); catalysts (to reduce barriers for chemical reactions); advanced corrosion protection (for higher product longevity); or battery cathodes (to serve in energy storage); etc. Also, research topics such as corrosion, tribology, abrasion, damage, failure, or fatigue are not covered here. They all have very high indirect impact on sustainability, due to their influence on product longevity and system efficiency, but they are regularly covered by expert reviews.

**Figure 20:**
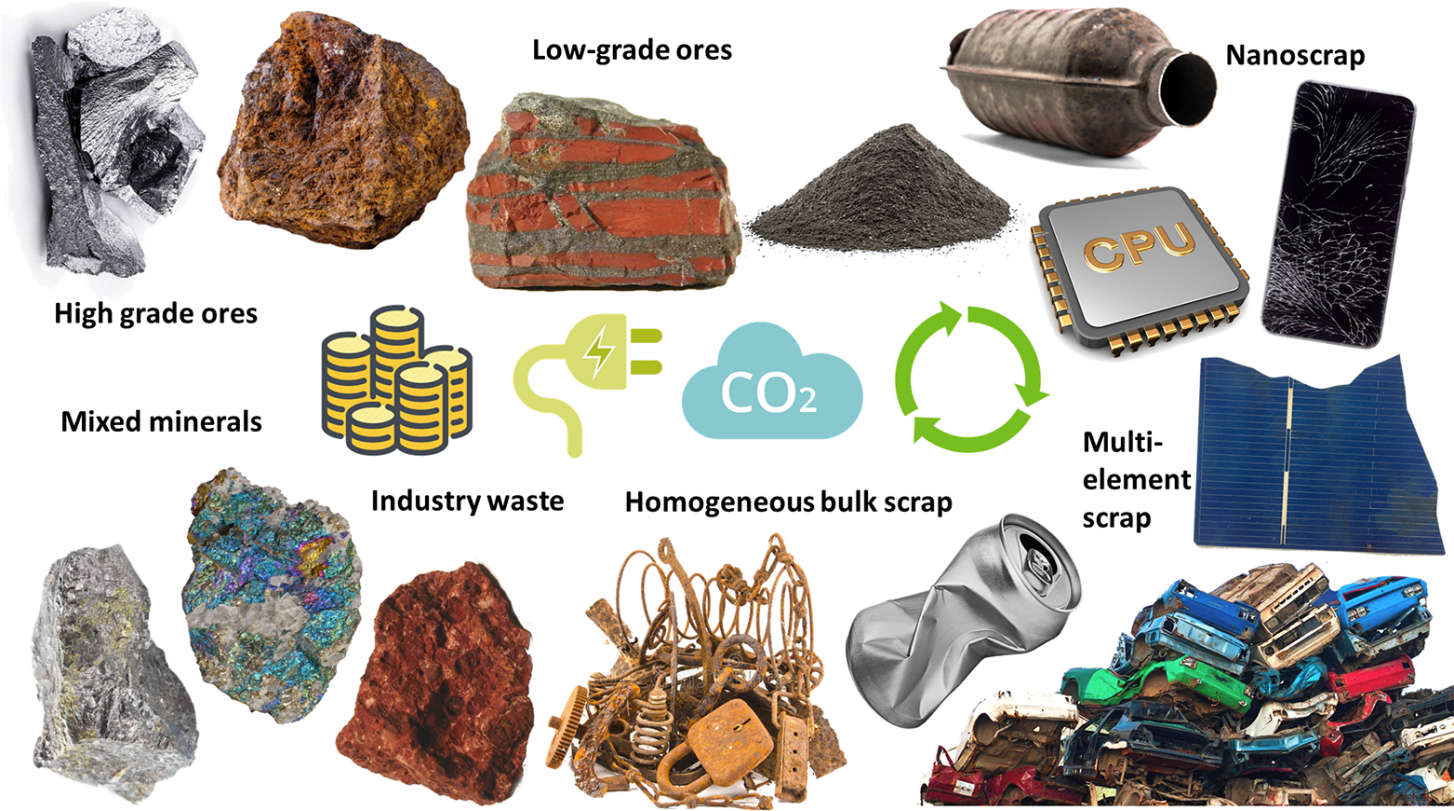
Figure summarizing some particularly important aspects of the direct sustainability of metallic materials that are addressed in this paper. Direct sustainability refers to all processes that deal with emission- and energy-reduced methods to produce metals and alloys. It includes all processes that particularly enable the CO_2_-reduced synthesis of metals by way of primary (from mineral raw materials), secondary (from scrap), and tertiary (from re-mined waste) synthesis. In this context the figure points to different types of ores (low grade, high grade): rich or dilute minerals for less abundant metals; bulk and compositionally rather homogeneous mass scrap (i.e., collected in-production); less well sorted, contaminated and post-consumer scrap (so-called old scrap); industry and post-consumer waste as new mineral-rich or rare-earth-rich feedstock or as alternative reductant (e.g., biomass); and multi-element recovery of metals from waste products that can contain more than 60 elements (e.g., notebook computers), a challenge referred to as nanoscrap recycling (see details in sections 3, 4.4, 4.8, 6.3.1, 6.3.7, 6.3.9, 6.3.10).

**Figure 21:**
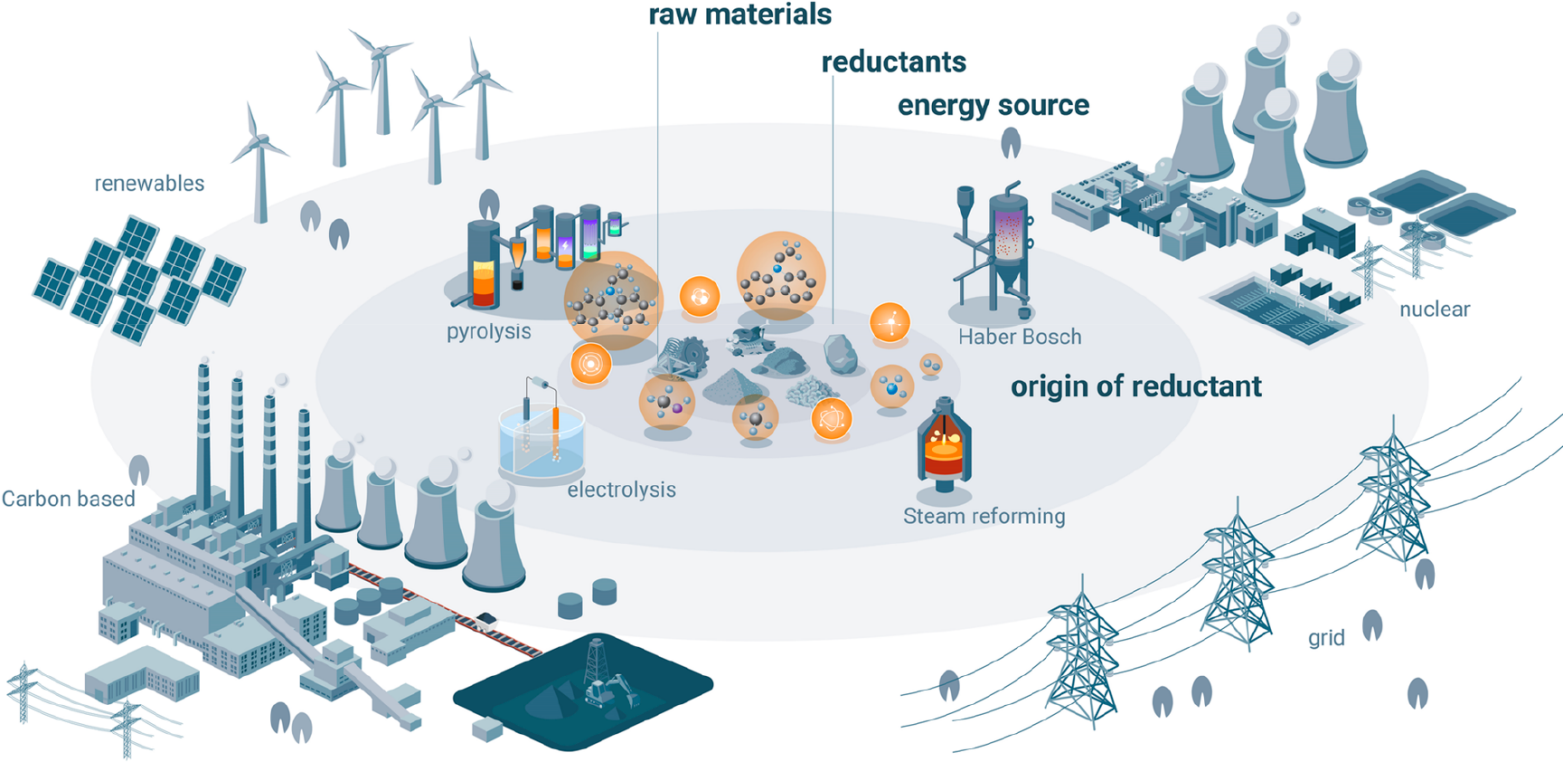
Overview of the many possible synthesis pathways for making more sustainable steel (as the most impactful material class when it comes to greenhouse gas emissions) and the associated industrial context from a sustainability point of view. For evaluating the sustainability of practically all of the technically feasible process combinations between the different technologies, raw materials, reductants, and aggregate states, the origin and degree of sustainability of the underlying electrical energy are essential. The entire electrification of the metallurgical sector will make the system only substantially more sustainable if the electrical energy which is needed for most of these process steps and also for the production of non-fossil reductants is renewable and fossil-free. Similar figures apply also for the other large metal groups, particularly for the case of aluminum production.^7^

This paper is instead concerned exclusively with research questions that act on direct sustainability, Figure 20.^2^ More specific, among the many sustainability criteria listed by the UN,^105^ this paper places focus on those basic metallurgical research topics that have the potential to particularly leverage lower greenhouse gas emissions, lower energy consumption, higher efficiency in the use of feedstock, lower by-product quantities and waste avoidance, and the direct use of sustainable electricity when producing metals. Figure 21 provide an example related to the latter point. The figure gives an overview of the many possible synthesis pathways for making more sustainable steel. For practically all possible combinations between the many different new (and mature) technologies, raw materials and (fossil-free) reductants, the origin and sustainability of the electrical energy is essential. The entire electrification of the metallurgical sector will make the system only more sustainable if the underlying electrical energy (which is needed for most of these novel process steps and for the production of sustainable reductants) is renewable and of fossil-free origin.

Naturally, such an endeavor of reviewing this large field of sustainable metallurgy has to set certain priorities. In the current paper, focus is therefore placed on those topics where the magnitude of the potential CO_2_ reduction could be very large.

The field of sustainable metallurgy is interdisciplinary and naturally overlaps with established disciplines such as extractive metallurgy, physical metallurgy, (electro-)chemistry and solid-state physics, as illuminated below in more detail. Yet, this paper does not try to recapitulate common textbook knowledge in this fields but instead aims to identify novel research opportunities with bottleneck character between these disciplines, regarding their specific leverage on the improvement of sustainability and particularly on CO_2_ reduction in metal production.

This means that this paper identifies, reviews and critically discusses some of the metallurgical key mechanisms and scientific bottleneck questions in the field of direct sustainability of metals and alloys. Emphasis is placed on the basic materials science and not on technological or economical aspects, fields about which several excellent overviews and books exist.^3,26^ The review tries instead to address the question what the fundamental materials science behind sustainable metals and alloys is, i.e. how the different scientific and engineering challenges in direct sustainability can be translated into basic research. Effects with highest leverage on sustainability will be primarily addressed, namely, measures for decarbonization, the use of hydrogen and its vectors for reduction, and electrified synthesis and recycling, with varying relevance for the different alloy groups.

In the end, it will be difficult to predict which technological and economic solutions will be implemented to make the metals industry more sustainable, as multiple boundary conditions set by legislation, societal acceptance, tax incentives, investment intensity and economic viability enter into this decision tree. However, what is certain is that, in order to solve this task, it is important to identify and study the scientific fundamentals, in order to provide ideas for long-term solutions for a more sustainable metallurgical sector, particularly for decoupling of the further increase in market demand for metals from the rise in CO_2_ emissions. The mission is urgent, as the sixth IPCC climate report 2021 clearly states.^106^

## 2 Rebound Effects Associated with Metals Needed for Green Technologies

### 2.1 The Conflict of Building Sustainable Technologies with Non-sustainable Metal Production

The term “rebound effect” refers to a situation where measures taken to improve the efficiency or quality of products and processes trigger growing consumption of goods and energy to realize such goals, that can partly cancel out the originally targeted savings.

**Figure 22:**
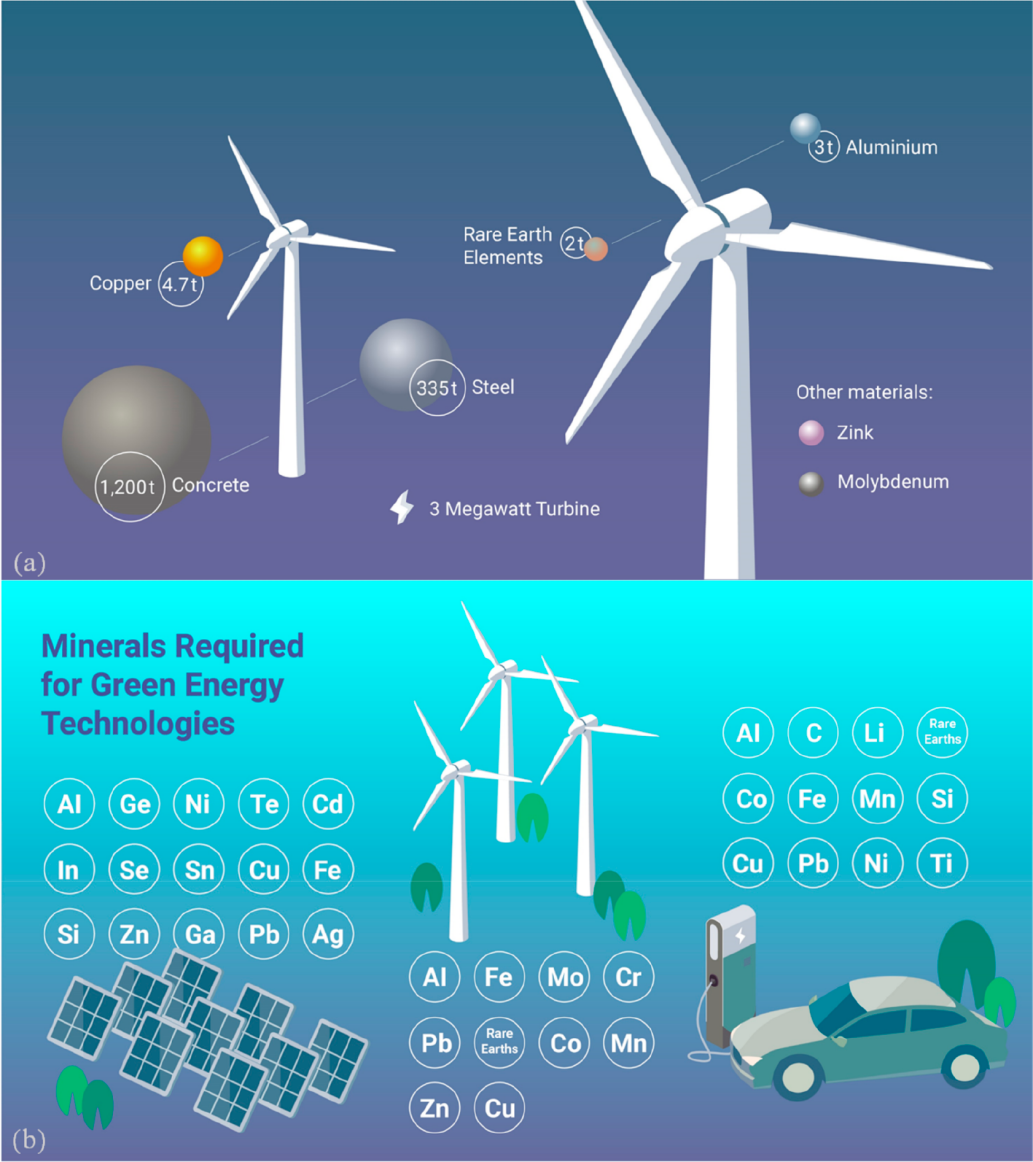
Rebound effect in terms of the over-proportional use of metals (produced with the current CO_2_ footprint) for realizing sustainable technologies. (a) Specific example of material usage for a 3 MW wind power plant. (b) Use of critical elements (with high CO_2_ footprint) required for manufacturing and erecting various types of sustainable technologies.

**Figure 23:**
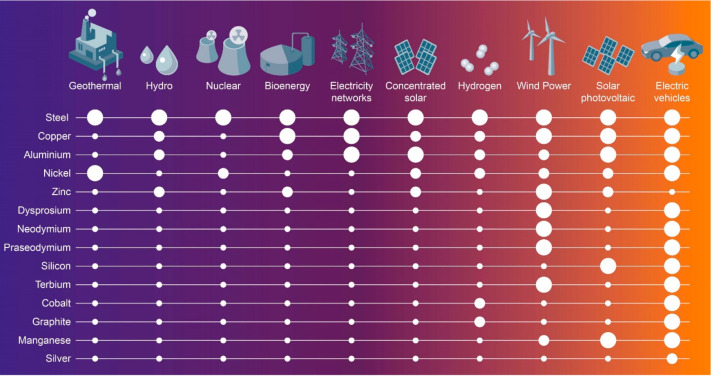
Origin of the rebound effect in the metallurgical sector: using sustainable technologies will be beneficial on the long run for the environment, but manufacturing these technologies with metals that are produced by the currently used CO_2_-intense technologies is not. The figure shows the intensity of material use for different technologies, revealing that a high amount of steel is needed for all of them.^108^

**Figure 24:**
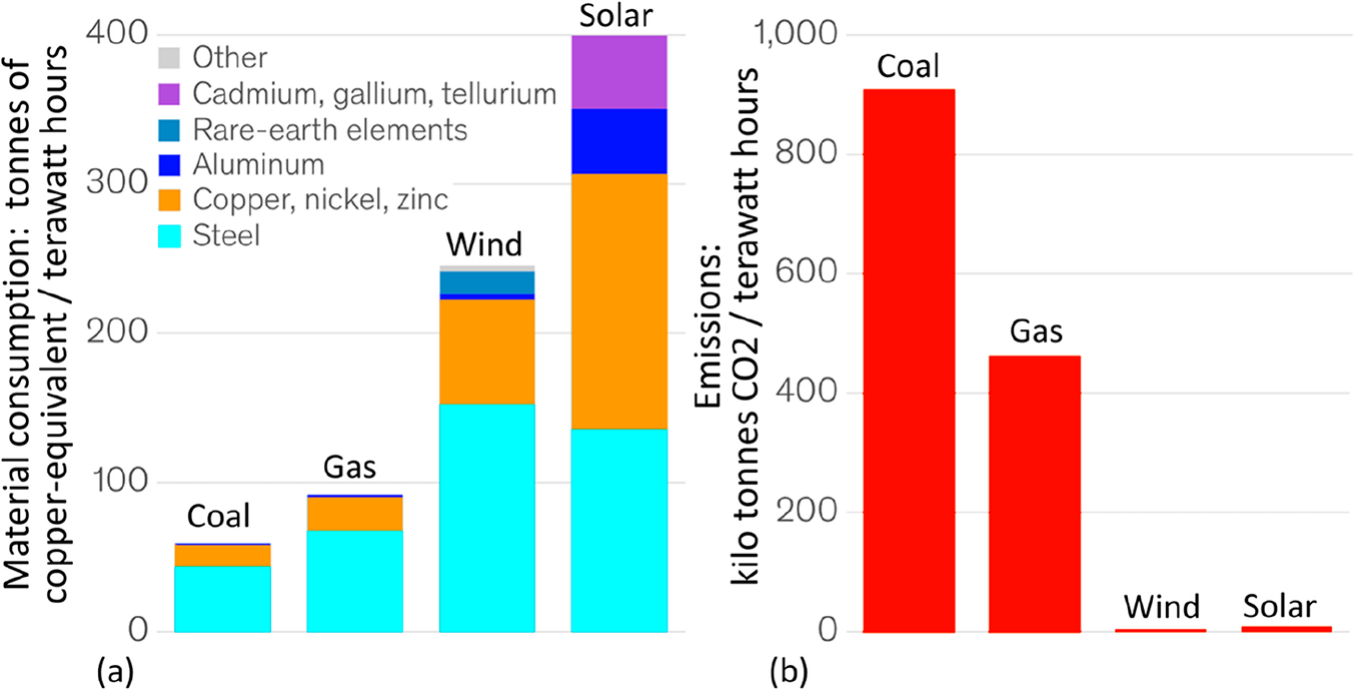
Rebound effect in the metallurgical sector for the case of power generation. For significantly reducing the greenhouse gas emissions from power generation a transition from conventional fossil-fired power plants to sustainable power generation technologies must be realized (particularly wind and solar). However, these latter technologies are much more material-intense than conventional fossil-fueled power plants. Since all the metals (and other materials) required for low-carbon power generation are produced by using the currently existing CO_2_-intense mining and production methods, the introduction of sustainable power generation will not lead to a reduction but instead to a temporary increase in greenhouse gas emissions.

**Figure 25:**
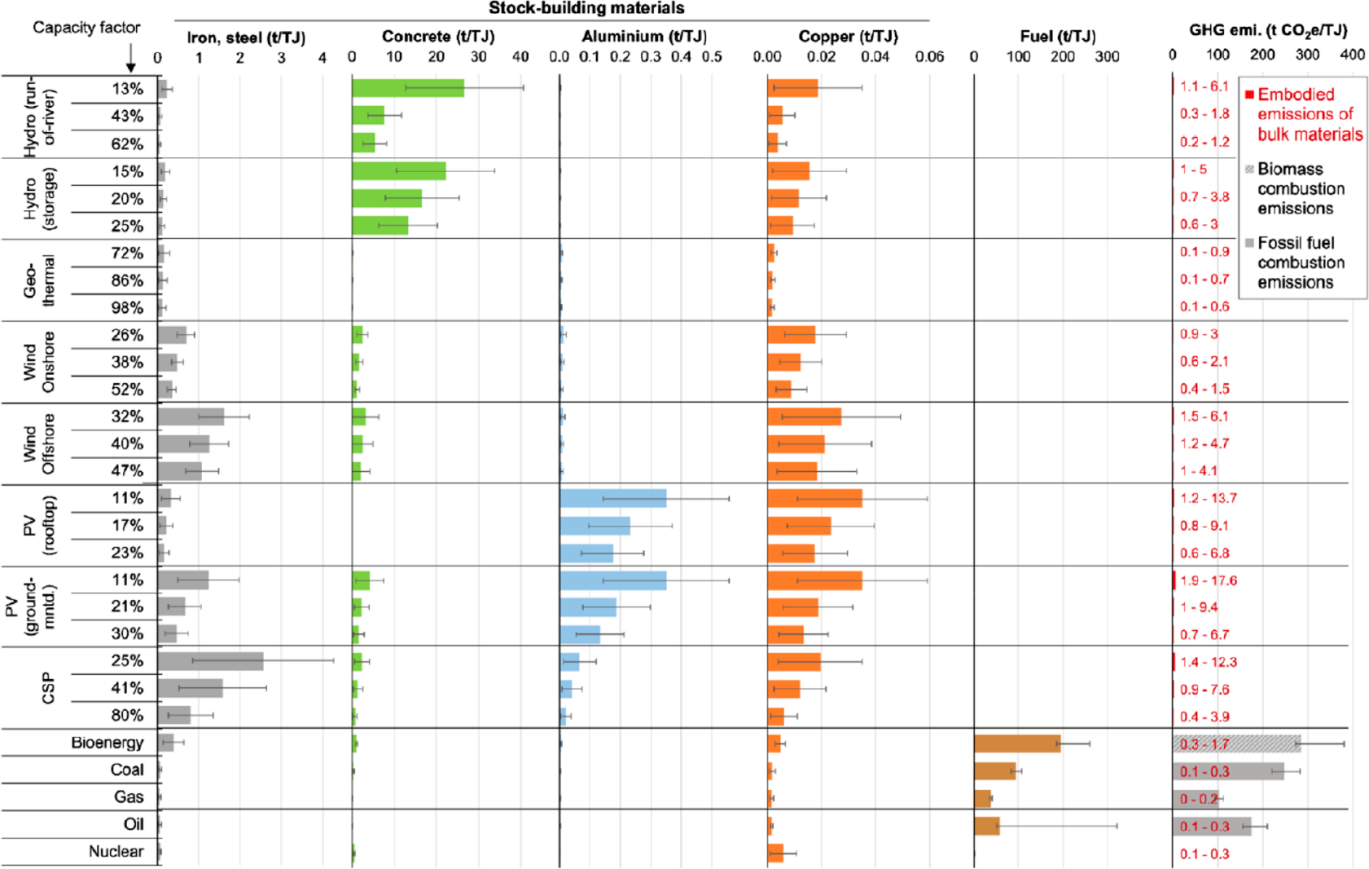
Material stocks used in the global electricity sector.^109^ Color bars represent estimates for the materials embodied in power plants related to the total electricity generation they enable over typical lifetimes. The uncertainties originate from ranges for material requirements in the literature (iron and steel; concrete; aluminum; and copper) and in the case of fuel consumption from ranges of typical efficiencies and calorific values of fuels. For example, colored bars for fuel consumption of coal power plants are valid for anthracite, while error bars include results for lignite; the high maximum value for “oil” represents oil shale power plants. Greenhouse gas emissions are broken down by combustion emissions and embodied emissions of bulk materials (the data shown in red font). The ranges of embodied emissions result from uncertainties in material intensities as well as material production emissions. GHG, greenhouse gas; PV, photovoltaics; CSP, concentrated solar power. Details are explained in the original paper by Kalt et al.^109^ Figure is reproduced from ref (109) with permission. Copyright 2021, Elsevier.

When applied to metals, this means that the *use* of green technologies such as wind farms, batteries and solar cells is good for sustainability but *making* these devices is not, due to the massive use of non-sustainably produced metals to build them, Figure 22. This applies particularly to the current situation where many parts of our technology, transport, household supply and industry systems are being transformed toward improved sustainability: all the machines and techniques needed for this (in large numbers) use metal production technologies that are actually not very sustainable today. Examples are concrete, steel and aluminum production (see details in section 2.2), Table 3. However, many sustainable technologies, particularly those required for reducing greenhouse emissions (wind farms, solar cell parks, etc.) are very material-intensive, thus creating a considerable rebound effect,^39,107^Figures 23–25.

This can be underpinned by numbers: the global primary energy demand per year is about 560 EJ (Exa-Joules, 10^18^ J) which equals 155680 TWh (terawatt-hours). In global average, about 85% of this energy demand comes today from fossil fuels. When replacing the fossil fraction by a more sustainable energy supply, all the necessary machines and processes must be produced and designed. The metals required for that must be provided, together with other resources such as water, land and concrete. As an example, producing one unit of sustainable electrical power from windmills and solar cells consumes approximately 200–300% more metals than producing the same amount of energy from a fossil-fired conventional power plant, when using a so-called copper-equivalent basis.^110^ This means that limiting global warming requires on the one hand a huge growth of the renewable energy sector, but on the other hand this creates a rebound effect which causes further increase rather than mitigation of greenhouse gas emissions, at least during the coming transition period in which these power plants are built using current metal production technologies.^111^

Several authors have assessed the magnitude of this rebound effect.^32,38,109^ Such estimates use a scenario where the current fossil energy production is globally replaced by a sustainable energy mix consisting of 32% wind energy, 45% photovoltaics and direct solar thermic plants, 9% hydropower, and the rest provided by fossil energy, geothermal energy, and biomass. According to a model of the International Renewable Energy Agency, the rebound effect can then be quantified. When referring here only to the fraction of the wind power as an example, this transition translates to seven million additional wind turbines worldwide, which is forty times the currently installed capacity. Two to three times more concrete and metal would be needed to build these machines, instead of building fossil power plants. Also, these power plants for renewable energy would have to be rebuilt from scratch, whereas fossil-fuelled power plants could be refurbished, and a considerable proportion of the material could be reused or recycled. It should also be noted in that context, as an additional alternative, that such conventional power plants could not only be modernized for higher efficiency, but they could also be operated and fuelled by using more sustainable energy carriers. This includes, for example, the direct injection of hydrogen (or ammonia, methane, methanol, etc.) as additional fuels or the injection of metallic powders as an energy-supplying fuel,^112,113^ provided that these substances are produced by using sustainable energy (see also section 10). In contrast, many of the metals required for erecting completely new sustainable power plants are not currently available on the market. This means that they would essentially have to be completely synthesized from primary raw materials, using current technologies which are associated with high greenhouse gas emissions, Table 3.

Particularly the required construction materials that are needed for this transition have sometimes been neglected in the calculation of such scenarios. The estimated demand (per unit of power produced by such plants) of steel, aluminum and copper for the required wind and solar energy exceeds the entire current world production by a factor of 3–15, depending on the specific scenarios and metals addressed, according to the raw materials fact sheet data from the EU action plan for critical raw materials and to data from the International Energy Agency,^114^Figure 22.

**Figure 26:**
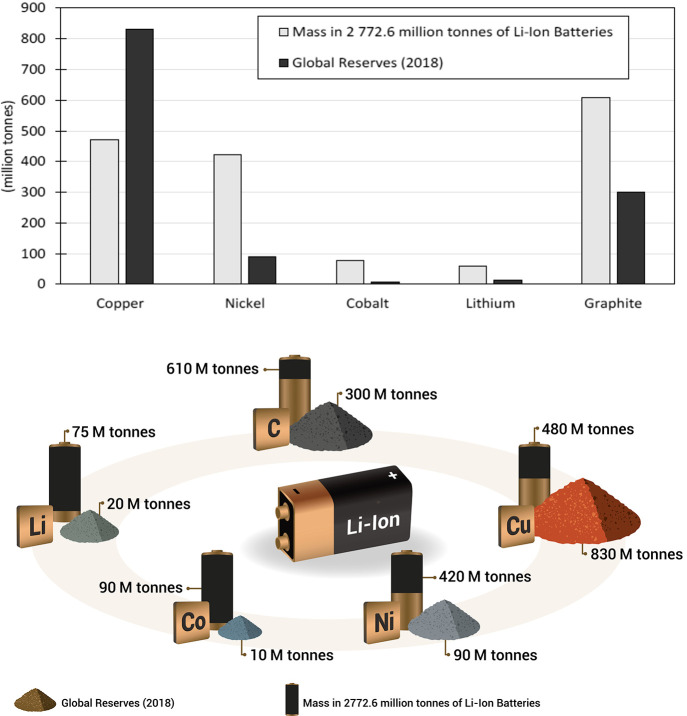
Amount of metals that are needed to buffer and phase out fossil-driven energy supply by using batteries (translating to a total battery mass of about 2773 million tonnes) according to data from the US Geological Survey Mineral Statistics for global reserves.^43^ The scenario calculation includes the massive introduction of electrical vehicles. For some metals the associated demand exceeds the globally accessible reserves. The figure is reproduced from the webpage https://countercurrents.org/2022/08/is-there-enough-metal-to-replace-oil/ with permission. Copyright 2022, countercurrents.org.

Not even included in these considerations is the provision of metals for the corresponding buffer technologies for the necessary energy storage. Buffering with batteries or artificial fuels is required because solar radiation and wind are both highly intermittent energy sources, Figure 26. Also, all the metals needed for the additional electrical (and gas) grid infrastructure and the required non-fossil fuel power stations for electrified transportation etc. have not yet been included in these rough estimates. As an example, the amount of metals needed to build an energy grid and buffer system for sustainably fuelling a global electrified vehicle fleet exceeds the amount needed to build these vehicles in the first place. More specifically, Figure 26 shows a rather drastic scenario of the expected metal shortage for the upper bound case that would be created when using only batteries for the phasing out of the fossil-driven energy supply. This means that the required power storage capacity to buffer the intermittency of the global renewable power supply from solar and wind was delivered entirely using lithium ion batteries. This translates to a total battery mass of about 2773 million tonnes, according to data from the US Geological Survey mineral statistics for global reserves.^43^ The scenario calculation includes also the massive introduction of electrical vehicles. For some of the key metals required in batteries, the associated demand exceeds the globally accessible reserves.

Another factor is the often moderate longevity of current sustainable technologies. The limited lifetimes of batteries and the high failure rates of wind turbines give ample proof of that. When reaching their lifetime ends, these products have to be scrapped like any other product also. This means that it must be considered in that context that green technologies must be strictly subjected to the same sustainability and recyclability requirements like any other product.

Owing to insufficient sustainable energy supply and insufficient electrification of metallurgical production, a large fraction (currently about 70%) of most of the metals needed for “green” technologies would have to be produced conventionally via synthesis from primary minerals using fossil energy and fossil reductants. This would produce massive additional emissions. When using the metal’s current CO_2_ footprint data, Table 3, this translates to an additional amount about 28 billion tonnes of CO_2_ emissions. This is about 75% of the current total global annual emissions of about 37 billion tonnes of CO_2_, which would be emitted only for providing the metals for the transition to one lifetime generation of the equipment and machines needed for a renewable energy sector. Time is also an important factor, because the reduction of the CO_2_ emissions from the metallurgical sector is an urgent task. Yet, when projecting the current growth rate in the renewable sector, it would take several hundred years to build up such infrastructures.

These very rough estimates make the need for a rapid decarburization of metallurgical production clear, to mitigate these massive rebound effects and enable growth of the sustainable technology markets with the least possible additional increase of the associated greenhouse gas emissions. As will be discussed in detail below, the main pillars for this are the massive electrification of primary metal production and of all downstream manufacturing steps with sustainable energy (see sections 7.5, 7.7, and 8); use of non-fossil reducing agents (see sections 6.1 and 7.4.6); massive growth of the recycling sector (see section 6.3); storage and transformation of carbon dioxide (although being only a transition technology for emissions mitigation); and a reduction in the range, amount, and chemical complexity of metallic alloys used (see section 9).

A further question associated with the rebound effect is if at all enough primary feedstock for this additional metal production is available (see section 6.2). In this context, it must be taken into account that the implementation of the first generation of a sustainable energy supply as well as the electrification of transport currently underway worldwide and the subsequent wave of electrification of industry and households must be produced essentially based on primary resources.^66^ This means that the materials used for this transition will then only become later available to the market as new recycling feedstock after a complete use generation of often several decades, Figure 11. It also implies that the metals for this additional production wave of green power and transport currently have to be provided (on global average) on the basis of about 85% fossil energy use and to a large extent via primary synthesis from minerals. This means that for the first generation of renewable energy and transport there is no feedstock for recycling available so that the required raw materials will have to come from traditional mining. However, an estimate from the International Energy Agency^114^ shows that the amount of metal required for one technology generation for a global renewable economy model is, according to current mineral reserves and mining rates, in part not sufficient to produce all the renewable energy technology needed.

### 2.2 Rebound Effects in Sustainable Metallurgy

**Figure 27:**
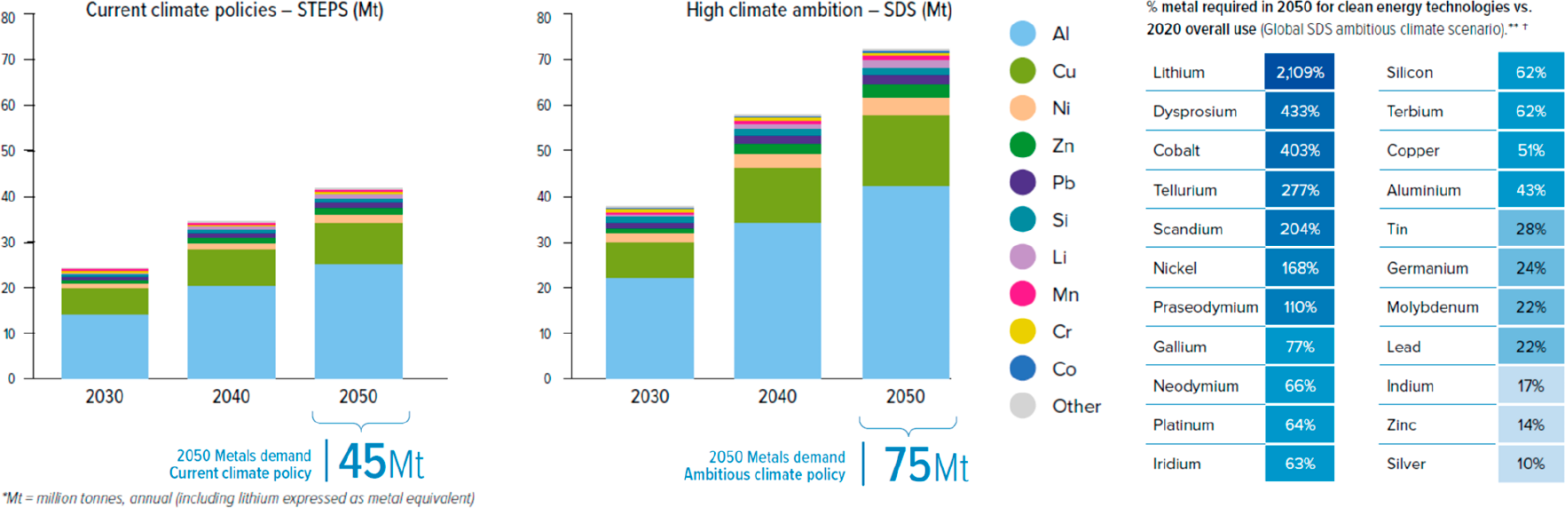
Demand for a number of metals for different sustainability transition scenarios (left, STEPS scenario; middle, SDS scenario). The data show that it depends on a number of decisions which specific research questions could emerge in the field of sustainable metallurgy in the near future. The scenarios were summarized based on data from the International Energy Agency for several 2020–2050 technology scenarios for global and EU climate pathways, presented in ref (30). The list on the right-hand side shows the estimated demand for various metals for the case of a sustainable development transition scenario. STEPS, Stated Policies Scenario (conservative benchmark); SDS, Sustainable Development Scenario (ambitious benchmark). The figure is reproduced (in modified form) from https://eurometaux.eu with permission. Copyright 2022, Eurometaux.

Figure 23 and Figure 24 show the specific future metal demands for the implementation of green technologies.^30^ This leads to the question how these metals can be provided in a sustainable fashion and to which research topics in sustainable metallurgy this translates to.^43^ The future sustainable availability of these metals depends on a number of factors, including particularly the choice in technologies that are used to realize this transition, Figure 23, Figure 24, and Figure 27.

Besides the general rebound effect that the transition from a fossil-propelled to a sustainable society and industry causes, many other rebound scenarios in the metallurgical sector deserve attention, each with specific challenges and opportunities for basic research:

1. Sustainable-Technology Rebound Scenario: The rebound effect from building a green society: this is the rebound effect from the need for metals required to build a renewable energy supply, an electrified transport and industry system, as well as grids (electrical, hydrogen, methane, etc.) and energy buffer infrastructures. As outlined above in more detail, this is a challenging process. It is driven by the transition of a more than 100 year grown and highly complex and well optimized industry and society “ecosystem”, built on an affordable and high energy density power source such as oil in a world which had projected practically “unlimited” feedstock and mineral resources into an entirely new system with similar or even higher complexity on the basis of intermittent and expensive energy and partially limited minerals supply conditions for a rapidly growing global population. The associated requirements for metal supply are shown for example in Figure 24. A specific example is the rebound effect coming from the very high future demand for cobalt and nickel, used for cathodes in lithium-based car batteries (see e.g. section 4.5). These two metals not only generate the highest CO_2_ emissions per tonne of metal produced (about 13–24, depending on synthesis methods and ores used), but they also have, especially in the case of cobalt, substantial social impact, such as child labor and the release of numerous harmful substances into the environment when produced under inadequate safety precautions. A related sustainability concern lies in the fact that the increasing consumption of such metals as nickel, cobalt, and copper and their already today limited supply from mining mean that less and less rich mineral deposits have to be exploited. And since there is an inverse relationship between the mineral richness in these metals and the CO_2_ footprint associated with their mining and extraction, this means that the drastically growing use of such metals for a sustainable energy and industry transition comes at the cost of using very dilute mineral deposits and an increased CO_2_ footprint of their extraction. Similar rebound aspects apply to some of the metals used to produce the magnetic materials that are needed for the electrification of industry and transport. Resulting research opportunities: All metals used for this industrial revolution—especially for the provision of sustainable technologies—must also be produced sustainably. Secondary synthesis in particular should be used here, i.e. the use of scrap, and as many metallurgical process steps as possible along the entire value chain should be electrified. For many of the particularly critical metals, e.g. rare earths, lithium, copper, nickel or cobalt, synthesis processes with lower CO_2_ emissions and less harmful waste products must be developed. Due to the emerging shortages of some of these metals and their very high dilution in the currently exploited mineral resources, the use of substitute materials should be considered with high priority. Examples are replacing copper with aluminum for electrification, nickel- and cobalt-lean battery electrodes, and magnetic materials devoid of rare-earth metals or steels with lean chemical composition. The scientific aspects of these approaches are discussed below (e.g., in section 9). Similar aspects apply to the mass-produced and highly CO_2_-intensive metals like steels and aluminum which are, for instance, massively used in the wind energy sector.
2. Scrap Rebound Scenario: The rebound effect from using scrap for synthesis (see also section 6.3): there is a potential metallurgical rebound effect associated with scrap. Collecting, sorting and melting scrap into new alloys is by far the fastest and most efficient way to rapidly reduce the massive CO_2_ emissions that come from the primary synthesis of metals. However, the major bottlenecks in this context are (a) the insufficient availability of scrap for many metals that are additionally required for the decarburization requirements sketched above; (b) the compositional quality of the scrap (mixed and post-consumer scrap versus new and well-sorted in-production scrap); and (c) the dispersion effect, which can cause a scrap-specific additional rebound effect. The latter problem comes from the problem of scrap distribution as well as the surface-to-volume ratio of the metal parts collected and subjected to recycling: if scrapped metals are too finely dispersed, then the CO_2_ emissions generated by collecting, sorting, cleaning and transporting these resources can exceed the CO_2_ savings from replacing primary by secondary synthesis. A similar concern applies to the ratio between coated surfaces and the underlying metal volume. In this context, the removal of surface layers such as paints and anticorrosion coatings can lead to higher CO_2_ emissions and other negative environmental effects such as toxic by-products than are saved by recycling very thin-walled material. Another rebound effect is that (d) a further increase in the use of scrap quantities in the smelting of new alloys will cause the prices of scrap available today to continue to rise sharply. Whereas scrap used to be in earlier decades a rather moderately used raw material (except for gold, copper, silver, and stainless steels), it has now become the most important and readily available raw material for producing metals with reduced CO_2_ emissions. Therefore, the global demand for scrap is increasing very strongly, which leads to an increase in prices. As a result, the competitiveness of recycled materials will decrease compared to alloys produced from primary synthesis. Resulting research opportunities: Scrap must be collected and sorted as early as possible in the production process and during dismantling of products in order to preserve its maximum value and not contaminate it. This also includes product design changes such as the capability to remove batteries, magnets and electronic parts form products prior to scrapping and shredding the whole product, a practice that currently stands strongly against responsible use of materials for secondary synthesis. Scrap should be mixed as little as possible and collected sorted by alloy. The number of different alloys (in terms of chemistry, not in terms of microstructure differences) in products should be lowered to reduce the compositional range of materials that are mixed during scrapping, thus reducing cross-contamination. Alloys should be developed in such a way that they become more mutually compatible when they are returned to the material cycle as scrap.^7,115,116^ This concerns particularly the ever-accelerating development of improved metallic alloys with an ever increasing complexity in chemical composition that offer an ever wider and better performance spectrum compared to their predecessors. Here microstructure- and processing-based alloys design in compositional systems with low numbers of alloying elements should dominate as an approach over chemically oriented alloy design with large numbers of alloying elements. While constant progress in alloy development enables, for example, the weight reduction of cars and aeroplanes and thus considerable energy savings, the recycling of chemically highly complex materials is becoming more and more demanding due to the necessity to meet the exact chemical specifications of such high-performance alloys also in new alloys made from recycled materials and can thus also lead to a rebound effect. In short, this paragraph illuminates the problem that very demanding and chemically complex materials are often also more difficult to recycle. Finally, alloys should generally be developed to be more chemically robust against impurities and impurity variations.^117−121^ Casting and downstream processing should be dynamically adjustable to equilibrate and balance chemical variations that intrude from scrap. More scientific details are discussed in the ensuing sections.
3. Hydrogen Rebound Scenario: There is another rebound effect created by the global trend to use hydrogen as reductant, fuel, and chemical energy carrier (see details in sections 7.4.3, 7.4.6, and 7.5). Specifically, a large contribution to this rebound effect comes when introducing hydrogen to replace fossil energy carriers and reductants in the metal industry. Two main effects matter most. The first one is the international sea transport of hydrogen from the sources of production to consumers. Long distances have to be bridged, and the hydrogen usually has to be liquefied for this purpose. However, during this liquefaction, up to 35% of the energy stored chemically in the hydrogen is lost again, required for cooling. The second rebound challenge is that the existing land-based pipeline networks are generally not designed for high partial pressures of hydrogen. Instead, there would first be a high level of new investment required for expanding the pipeline networks with steels that tolerate high hydrogen partial pressures, without undergoing hydrogen embrittlement.^122^ While many existing steel pipes are currently capable of tolerating moderate hydrogen partial pressures, the current infrastructure is not sufficient to provide the same amount of energy as currently provided by natural gas, which means that the partial pressure of hydrogen would have to be substantially enhanced. The reason is that not the amount of hydrogen transported matters but the amount of energy that is delivered to the customer. The additional steel production required for this again generates very high CO_2_ emissions if current production technologies are taken as a basis. Another effect in this context is that there is not enough sustainable energy available to produce green hydrogen in the required quantities (110–150 million tonnes of green hydrogen would be needed every year to remove fossil reductants and fossil heat from the metallurgical sector), nor sufficient electrolysis capacity for water splitting, where particularly the lack of the required catalyst quantities is an issue. This will lead to the use of less sustainable hydrogen production methods, which will again lead to high additional CO_2_ emissions. One possible strategy to counteract these various rebound effects associated with the introduction of hydrogen as a non-fossil reducing and heating agent is to move metal production to the locations of sustainable hydrogen production, i.e. to reduce the metal from its ore at the same location where the minerals are mined. This means that it would be more efficient to transport the reduced metal across the oceans to the consumers instead of the hydrogen (or hydrogen vector gas carriers such as “green” ammonia or methanol etc.). Such an approach would be generally more sustainable because metals have a much higher energy density than most of the chemical energy carriers used today. This means that metals are better energy buffers than lithium batteries, hydrogen or ammonia. Resulting research opportunities: Improving materials to withstand hydrogen embrittlement for a hydrogen-capable distribution system that allows very high partial pressures. Development of alternative methods to produce sustainable hydrogen. Investigation of reduction and heat treatment processes in the metal industry with different mixtures of non- (or low-) fossil reducing and combustion agents. Investigation of primary synthesis based on different mixtures of reducing agents as well as the use of hydrogen-containing plasma in direct reduction (solid phase reduction) and in smelting reduction (liquid phase reduction). Study of the fundamentals of reduction processes that achieve the highest possible stoichiometric efficiency (exploitation) of hydrogen at highest metallic yield.^123^ Currently, too many processes are being discussed that may not use hydrogen efficiently enough; i.e., hydrogen must be seen as any other expensive raw material in this sector, and reduction methods must be developed which use it very efficiently. Recovery and closed loop refeeding of unused hydrogen in the field of primary synthesis is also important. Finally, for some metallurgical synthesis and processing operations it is more sustainable to avoid the use of hydrogen altogether but to use instead direct electrification, provided the electrical power comes from renewable sources. Another pathway is to use metal powders directly as renewable fuel for combustion systems. More scientific details are discussed in the ensuing sections (e.g., 7.4.6 and 7.5).
4. Coastal Protection Rebound Scenario: Another scenario is that, at the current rate of global CO_2_ emission reductions, it is not very likely that the Paris Agreement, which was supposed to limit global warming to an increase of no more than 2 °C, will be met. In order to achieve this goal, much more drastic cuts in global CO_2_ emissions would have to be made practically instantaneously, measures which are globally currently not in sight. This will lead to a rise in sea levels and thus to the flooding of many coastal regions, including many cities with millions of inhabitants. Hence, enormous structures will have to be built for the corresponding flood protection, using very large quantities of steel and concrete. Numerous examples for arriving at a corresponding estimate of the quantities of materials needed for coastal flood protection can be made on the basis of corresponding structures in The Netherlands or the UK. The same applies to the expected drying out of large regions, which will make it necessary to provide huge quantities of freshwater from desalination. These huge industrial plants will require large amounts of stainless steel and titanium, as well as huge amounts of energy. These are only two of many other examples of how the expected global warming will lead to a strongly disproportionate consumption of metals, and thus further massively promote the CO_2_ increase, if no corresponding sustainable processes for metal synthesis and further processing are developed. Resulting research opportunities: Development of drastically energy- and CO_2_-reduced synthesis and production methods for metallic materials (and for concrete).

## 3 Categories for Research in Direct Metallurgical Sustainability

Research on direct sustainability of metallic materials is not a homogeneous well-developed discipline. To identify research opportunities with high leverage on sustainability requires consideration of the minerals, metals, processes and amounts produced. For example, research challenges differ profoundly among such diverse problems as the reduction of CO_2_ emissions in iron making^124−128^ (see sections 7.4.2–7.4.8); development of scrap-tolerant aluminum alloys^7,115,129−132^ (see sections 6.3.6, 9.1, and 9.2); electrochemical recovery of gold and copper from consumer electronics^11,133−135^ (see sections 6.3.9, 6.3.10, 6.3.11, and 6.5); extraction of lithium from scrapped vehicle batteries^111,136,137^ (see section 6.3.10); sustainable production of nickel and cobalt^111,138,139^ (see sections 6.2.9 and 6.2.10); or recovery of precious metal catalysts^140^ (see section 4.8).

Besides these element- and alloy-specific perspectives, the field is divided further into many more topics and subdisciplines (such as processing; thermodynamics; kinetics; in-operando probing; etc.), each with different relevance and specific research challenges when targeting the sustainability of metals. This section therefore collects and contrasts a number of processing methods, metallurgical mechanisms, leverage effects and alloy categories that can be used to organize and guide research opportunities for improved direct sustainability of metals. It serves as a basis to identify and contextualize promising research topics with high relevance and also helps to organize this article, Table 6.

In the chemical engineering literature, categories for metal extraction are well established. Traditionally, the science and technology of winning metals from raw materials (such as ore minerals, tailings, industry and urban waste, scrap, etc.) under rejection of undesired gangue elements is referred to as extractive metallurgy, including intermediate steps such as separation, accumulation and benefication. The final step is the actual extraction of the metallic material, either as pre-alloy, due to the often mixed and contaminated mineral and waste feedstock, or in elemental form. Some details of these methods are discussed in section 7.

Extraction processes are grouped into pyrometallurgy (use of high temperatures), hydrometallurgy (use of liquid solutions) and electrometallurgy (use of electricity), of course with some degree of overlap. In the context of sustainability, new subdisciplines of these fields gain momentum, such as, for instance, solvo-, plasma-, iono- and biometallurgy.^93,141,142^

More specifically, pyrometallurgy refers to the treatment of ores or scrap at high homologous temperatures, converting them into metals or intermediate compounds (see section 7.4). Hydrometallurgy deals with aqueous solutions for recovering metals from ores, waste or solutions of recycled content (see section 7.6). Electrometallurgy encompasses metal extraction methods based on electrolysis, making direct use of electrical energy to produce metal by cathodic deposition from solutions (see section 7.7). Electrometallurgy overlaps with hydrometallurgy and includes methods such as electrowinning from molten metal-containing salts or electrorefining where metals are dissolved in or extracted from solutions.

Iono- and solvometallurgy are variants of hydrometallurgical methods which target metal extraction using non- or low-aqueous solutions.^142^ The difference to traditional hydrometallurgy is its focus on sustainability, avoiding water as a solvent phase, using instead either organic or inorganic solvents of sustainable origin. Ionometallurgy employs particularly ionic liquids and salt eutectics for electrowinning and metal leaching at low temperatures, a technique which can be of high relevance, for instance, for the room-temperature reduction of iron oxide. In principle hydrometallurgical processes are often better suited for the extraction of metals from low-grade mineral mixtures and often allow better control of by-products. The role of hydrometallurgical extraction might gain momentum over the next decades owing to the gradually decreasing quality of accessible ores (which often advocate hydro- over pyrometallurgy) and owing to the rapidly growing role of heavily mixed post-consumer scrap and also from tailings, from which particularly precious and harmful elements are otherwise hard to recover.

Plasmametallurgy uses ionized electrically neutral gas mixtures consisting of free electrons and ionic and neutral species for efficient melting, ore reduction and refinement of industrial residues^143−145^ (see section 7.5). Both thermal and cold plasma states are studied in this context, where special attention with respect to sustainable metallurgy is placed on the use of fossil-free hydrogen-based plasmas.

Electro- and plasmametallurgical techniques are attractive for sustainable metal synthesis owing to their direct use of (potentially sustainable) electrical energy for operating the electrolysis or, respectively, generating the plasma states, for instance in electric arc furnaces.

**Figure 28:**
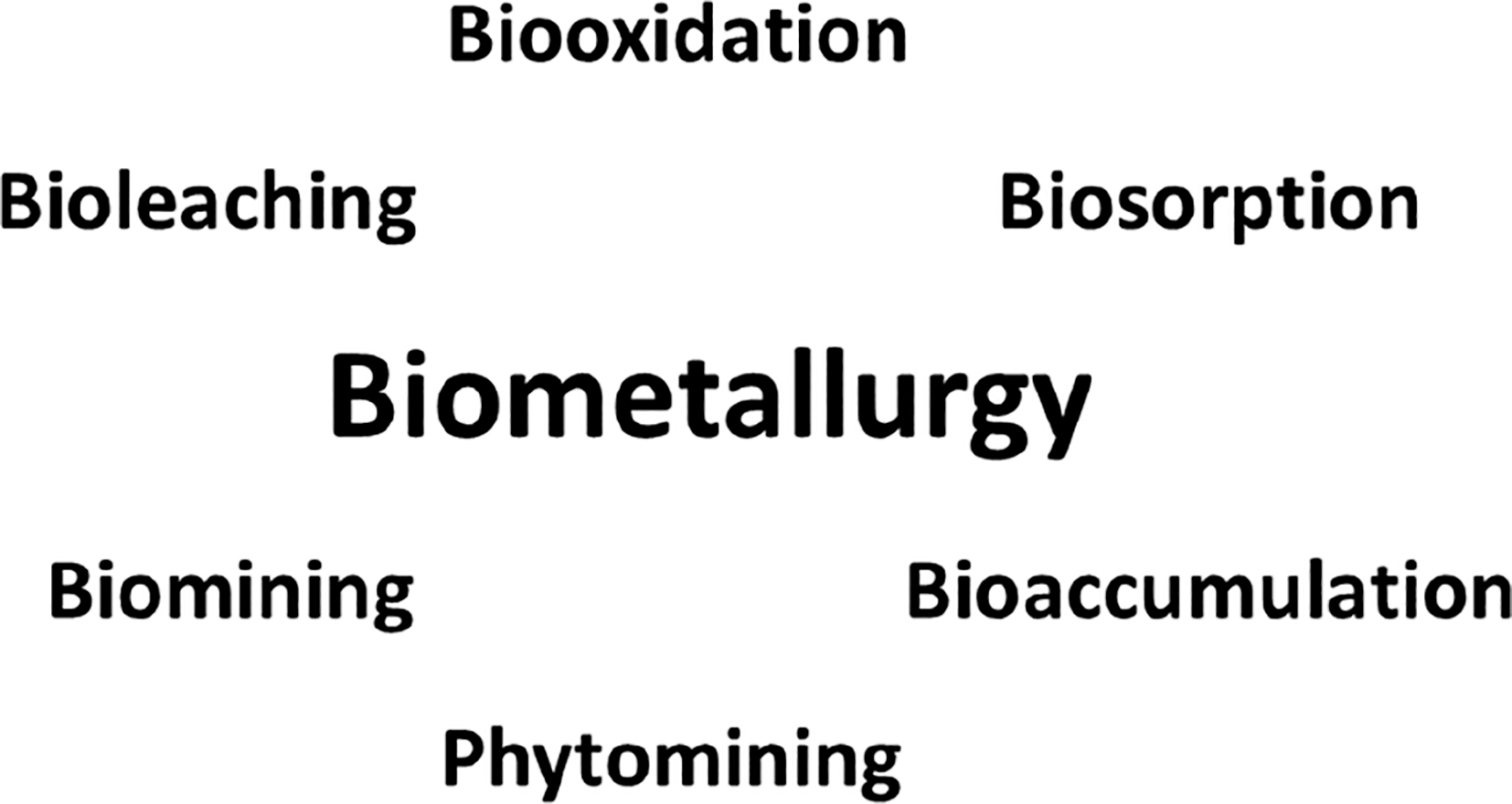
Main methods used in biometallurgy: biomining, -leaching, -sorption, -oxidation, -accumulation as well as phytomining.

Biometallurgy refers to a set of methods where microorganisms such as bacteria and fungi can serve to mine, accumulate, leach and bioadsorb metals from oxidized and waste feedstock,^93^Figure 28 (see section 7.8). Bio-leaching describes the dissolution of metals directly by microorganisms or through their metabolism, producing organic components which help to accumulate and solubilize elements. Biometallurgical methods are often used together with hydro- and solvometallurgical methods. Like electro- and plasmametallurgical approaches, they are promising for sustainable recovery of (currently mostly precious) metals from ores and waste streams, Table 6.

Some of these established extractive metallurgy methods are currently revisited in research, with the aim to render them more sustainable, as some of the conventional procedures involved were environmentally hazardous. Examples of technically questionable process steps include, in particular, environmentally harmful chemical solvents; toxic intermediate and waste products; contamination of water and air with pollutants and greenhouse gases; use of unnecessarily high temperatures or pressure conditions; inefficient use of oxidizing and reducing agents; or the use of toxic amalgams, to name but a few important challenges for research on possible process improvements in this field.

Also, many of the conventional extraction and synthesis methods were originally designed and optimized for the use of very specific (fossil-based) reductants, minerals, energy carriers and solvents.

This means that many of these processes must be revisited, to accommodate or even altogether “reinvent” them for new types and combinations of mineral, scrap, reductant, or solvent feedstock materials, Figure 21. The main changes in boundary conditions for the more sustainable operation of metallurgical processes are primarily the reduction in (a) greenhouse gas emissions; (b) energy consumption, (c) harmful by-products, (d) water usage and (e) soil and water contamination. The removal of any feedstock items of fossil origin from the equation is among all targets the most important one, with the highest leverage on improved sustainability of the metallic sector. This is a huge research challenge, as many of these processes have been designed and optimized—literally often over centuries—for the use of fossil energy carriers and reductants.

For example, traditional pyrometallurgical methods have often been significant producers of greenhouse gas and dust emissions. Conventional hydro- and electrometallurgy require large volumes of acids such as H_2_SO_4_, HCl, KCN, and NaCN, which have in part only limited selectivity, for instance when it comes to the recovery of precious metals from mixed electronic scrap. Even highly toxic methods, such as cyanidation and Hg-based amalgamization are still used in some regions.

Another opportunity is that the vast majority of metallurgical process techniques has in the past been designed for the extraction of metals from minerals, but multiple new techniques and basic mechanisms must now be found to recover metals from the rapidly growing mass of (mixed, contaminated, and nanostructured) scrap, in a sustainable way. This includes a variety of scrap types, ranging from large volume materials with rather homogeneous and well-defined chemical composition that can as so-called new or runaround scrap be recovered directly during production (see sections 6.3.2, 6.3.4, 6.3.5, and 6.3.6) to nanoscrap with parts only a few atoms in thickness and which are often mixed with more than 40 other elements such as, for instance, in microelectronics parts (see sections 6.3.9–6.3.11 and 7.8.2). These examples show that new technologies must be found to recover metals under such complex and variable conditions from primary, secondary and tertiary feedstock. Yet, the established extractive metallurgy methods can match only some of these challenges, which opens up multiple opportunities for basic research in this field.

One cornerstone of these considerations is that the circular portion of the metal economy will drastically grow in the next decades (e.g., from about 1/3^rd^ to 2/3^rd^ of the iron and aluminum mass markets until 2050–2060) and the volume of metal extracted from minerals will shrink to about 1/3^rd^ from 2/3^rd^. This means that research has opportunities to not only improve sustainable primary synthesis pathways but also find sustainable secondary (from scrap) and tertiary (from re-mined material) synthesis pathways and mixed metal retrieval where mineral, scrap and waste material can be jointly treated by extraction, with high selectivity and under use of sustainable reductants and heat sources. The reason is that the next decades will be characterized by a massive transition from scrap being the current minority metal source toward scrap being the majority source for metallurgical synthesis in the future.^133,146−153^ This does not only bring up the question how metals are won from scrap, with high selectivity, efficiency, and cleanliness, but it also means that alloys, joints, parts and manufacturing processes must be seen as both a product and a circularly reappearing future feedstock when returning as scrap. The consequence of this approach is that any alloy and any product-containing metals must be considered and designed from both perspectives, as a consumer good and also as a future provider of scrap.

This means that the field of direct sustainable metallurgy is branching far beyond the traditional bounds of conventional extractive metallurgy, chemical engineering or physical metallurgy. The reason is that cross-disciplinary aspects such as the thermodynamics and kinetics of sustainable reductants; low-quality and compositionally highly mixed mineral feedstock and fine ores; mixed scrap and post-consumer waste feedstock types; or re-mining and metals extraction from dumped waste deposits will play much larger roles in the future. These challenges require us to gain understanding of how to get every atom from such mixed material streams back into the manufacturing chain; how to efficiently extract metals from heavily contaminated feedstock; how to treat all such feedstock types (waste, minerals, ores of different quality, and old and new scrap) in the same reactors; and how to achieve all these goals without fossil reductant or fuels and at low energy input. The latter point is an essential constraint, as many reactions encountered in sustainable metallurgy are endothermic and not exothermic (like in the case of fossil-based reactions); i.e., huge amounts of heat must be provided (with minimum carbon footprint) (see some details in section 5.1). This is a point often overlooked, as electrification and sustainability of the metallurgical sector has traditionally used fossil feedstock not only as a reductant but also as an endothermic reactive source of heat, an advantage which is usually lost when developing carbon-free synthesis methods. Basic knowledge about most of these topics and the consequences of the reaction driving forces associated with sustainable reductants is still in its infancy.

Finding solutions to these challenges requires us to bridge the disciplinary and educational bounds that have evolved over many decades between the extractive disciplines such as pyrometallurgy, hydrometallurgy and electrochemistry, on the one hand, and physical metallurgy and metal physics, on the other. The design of sustainable manufacturing processes, products, and alloys must be seen together, as each part has a life as product and as raw material after its use.

Such a more holistic view becomes clearer on a practical example: let us take a step back and place all these ingredients and constraints on a mind map, for instance, for the case of steel.

Only considering the multiple types of possible feedstock (minerals, scrap, re-mined waste, reductants, etc.), their aggregate states and their origin (how was it made and which energy source was used) reveal how many possible process pathway combinations emerge, all equipped with different sustainability figures. Figure 21 shows this for the case of iron and steel production. Specific subtopics of this overview diagram will be discussed in the next sections (6.2.2–6.2.5 and 7.4.2–7.4.8).

The complexity of the figure shows that it can be helpful to get back to the roots and interrogate even very basic processes which were developed in part over centuries, and revisit their scientific foundations under nowadays’ more holistic sustainability constraints.

The compilation of topics sketched in Table 6 collects some of the main facets and opportunities in basic materials research behind the direct (synthesis- and recycling-oriented) aspects of sustainable metallurgy introduced above. They serve as starting points to discuss the different effects in more detail and select from them a few which are discussed in greater detail in this paper. The categories also may help to give this large, inhomogeneous, multifacetted research field some structure, to better identify the main common emerging research themes. The sections in this paper follow the categories listed in this table.

**Table 6:** Possible Categories to Group Research Subjects Related to Direct (Production-Related) Sustainability of Metallic Materials^a^

Category	Materials-science-related research topics on direct sustainability
**Alloy-class-specific direct sustainability research topics**	
Alloy-class-specific differences with respect to primary and secondary synthesis	Structural alloys (carry loads, huge quantities, high leverage on greenhouse gas emissions and energy consumption, high amounts of scrap) and functional alloys (scarce, hazardous, precious, nanoscale integrated in products, small quantities but high value, difficult to recycle, multi-metal recycling, high dispersion in scrap); high-leverage research directions can be grouped (in absolute or mass-normalized numbers) along categories such as the metal quantities produced (e.g., Fe, Al); greenhouse gases emitted (e.g., Fe, Al, Ti, Ni, Co, Mn, Si); specific energy demand (Ti, Al, Ni, Co, Fe); low vs high end of life recycling rates (e.g., Pt group metals vs Al alloys); waste and new (runaround) scrap generated during production and manufacturing (Ti, Al, Fe); bulk availability of scrap (Fe, Al); electrical, electronic and nanoscrap (Cu, Au, Ag, RE); high vs low recovery grade (e.g., Pb, certain Al alloys, stainless steels); closed loop recycling (specific alloy to alloy recycling) vs open loop recycling (sorting required); environmental harm caused by mining, by-products and tailings (Al, red mud; Au, mercury); strategic relevance and mineral scarcity (e.g., rare earth elements, platinum group metals, Au, Cu); social and labor standards associated with production (Co, Ni); re-mining of metals from dumped waste
**Relevance of intrinsic properties of specific metals and alloys with respect to direct sustainability**	
Intrinsic physical properties of specific metals and alloys	Redox thermodynamics for different metals; potential for the use of different types of reductants and their mixtures; transport, nucleation and general kinetics in redox reactions during reduction; effect of impurities and gangue elements on thermodynamics and kinetics of direct reduction, plasma reduction, and scrap melting; differences in (multi-metal) recycling for metals with low or high melting points; use of pyrolysis methods in recycling; solubility and intermetallic phase formation from scrap- and mineral-related impurity elements; magnitude of the free energy of the main minerals used for reduction; scarce or abundant; toxic or harmless; toxic gangue or not; difference in free energy of the main mineral types; thermodynamic competition between primary and secondary synthesis
**Types of feedstock for sustainable metal production: mineral and metallic solids, liquids, plasma, gas**	
Principal differences in feedstock types, feedstock quality and reduction methods	Synthesis from primary (minerals), secondary (scrap) or tertiary (re-mining of deposited waste: “urban feedstock”) feedstock; basis of linear metal economy (mining, agglomeration, by-products landfill); evaluation of circular economy effects for different metals; re-mining metal economy; low price feedstock such as banded ores, fine ores and mixed scrap; use of less pure, mixed and cost-efficient reductants (science of “dirty” feedstock and the science of “dirty” alloys); competition between direct reduction in liquid or solid state; electrolysis for direct use of sustainable electrical energy; competition and trade-offs with regard to feedstock costs, sustainability and efficiency for hydrometallurgy, pyrometallurgy, electrochemistry, plasmametallurgy, and bio-hydrometallurgy.
Primary (mineral) feedstock types/ores	Quality, concentration, dispersion and abundance of mineral feedstock; benefication and agglomeration techniques; waste and by-products including hazardous by-products; use of dilute and impure ores vs rich ores; gangues elements in ores; primary synthesis methods for less pure mineral mixtures; removal and inheritance of gangue elements into alloys and impurity element removal
Secondary feedstock types/scrap	Bulk and well sorted alloy-specific scrap versus unsorted multi-element and post-consumer scrap; availability of scrap; mixing scrap with primary reduced and partially reduced minerals; metals and alloys with high recycling rates (can stock; stainless steels) versus low recycling rates (catalysts, electronic materials, magnetic materials); recycling-oriented alloy design; source-, sink-, receiver-, acceptor-, and donor-alloys with regard to scrap use and scrap source; closed-loop-specific alloy-to-specific alloy recycling; open-loop-specific alloy-to- general alloy class recycling; the science of “dirty” alloys; effects of variable chemical composition from scrap on downstream production
Tertiary feedstock types/industry waste/re-mining	Re-mining and recycling methods for dumped waste materials as new feedstock (“urban feedstock”); multi-element recovery from re-mined feedstock; rare-earth element extraction from re-mined material.
Biological and organic feedstock	Use and genetic design of bacteria and fungi for bio-hydrometallurgy; use and genetic design of super-accumulator plants for improved accumulation and plant mining; use of organic waste as reductant; biomining; urban biomining; moderate use of biomass (so as to not compete with crop production)
Reductants	Use of variable and mixed non-fossil reductants; use of low-purity reductants
**Sustainable approaches in extractive metallurgy: pyrometallurgy, plasmametallurgy, hydrometallurgy, solvometallurgy, ionometallurgy, electrometallurgy, biometallurgy**	
Pyrometallurgy	Direct reduction methods, mechanisms and process parameters; smelting reduction methods; use of mixed reductant feedstock; thermodynamics of pyrometallurgical processes; differences in feedstock and fuels; role of microstructure and transport in direct reduction; types of ores and pellets with different purity; multi-element pyrometallurgy
Plasmametallurgy	Plasma-based reduction methods and mechanisms; exited states and plasma species; nonequilibrium effects; metal evaporation and contamination of plasma during reduction; spatial distribution of temperatures and species; contact dynamics between plasma and oxides; complex mineral reduction by plasma; plasma spectroscopy; direct solid-plasma-state reduction; liquid-plasma reduction; slag formation in plasma reduction; carbon-free plasma arc electrodes; and their reactivity, influence of gangue, nucleation and growth phenomena in oxide melts; slag metallurgy in the case of less pure ores: kinetics and metallization in plasma reduction; CO_2_ reduction and efficiency; near stoichiometric use of non-fossil reductants
Hydrometallurgy, solvometallurgy and ionometallurgy	Leaching mechanisms and kinetics; solvent extraction mechanisms; energy; efficient process design; hydrometallurgical methods tailor-made for metal extraction from recycled intensely mixed, impurity-contaminated and high-component materials (such as electronic scrap); recycling of all raw materials used in hydrometallurgy; precious and harmful metal recovery from post-consumer scrap and tailings; new solvents and leaching from ionic liquids; electro-leaching; mechanisms of selective precipitation
Electrometallurgy	Electro-slag formation, improved salt solutions; electrochemical foundations; inert and carbon-free electrodes; electrowinning of metals from aqueous solutions; low-temperature electrometallurgy; ionic liquids as electrolytes; variable cell operation (to use sustainable intermittent sustainable electricity)
Biometallurgy	Optimization of bacterial and fungi-based accumulation and reduction; microbial catalysts for new bioprocesses in mining; ore, waste and scrap feedstock pretreatment and beneficiation suited for bio-hydrometallurgy; bio-leaching and recovery of metals from low grade ores and wastes; biotechnical treatment of process waters and effluents from mining and metallurgy; characterization of microbial communities in bio-hydrometallurgical processes and mining environments; microbiological element recovery from contaminated soils
**Relevance of downstream processing and manufacturing in direct sustainability**	
Lean casting and forming processing	Near net shape casting; thin strip casting (mm- thickness), thin slab casting (few cm thickness range); large-scale squeeze casting of scrap-tolerant and sink alloys; microstructure design for alloys that require less rolling reduction and less homogenization treatment; lower temperatures during hot rolling; additive manufacturing; in-line scrap collection; adaption of dynamic processing for variable chemical alloy content; processing for “dirty” alloys
Sustainable heat treatment	lower temperature and shorter homogenization heat treatments; low-temperature and shorter precipitation heat treatment; alloy design that allows reduction and/or elimination of homogenization and precipitation heat treatment; heat treatments with nonconstant and variable (chemistry-specific) temperature profiles; alloy-specific simulation of heat treatments with regard to minimum energy consumption; adaptation of heat treatments for recycled alloys with higher impurity contents; replacing CH_4_-based combustion technology for furnaces with H_2_-based combustion and with electrical heating
**Sustainable alloy concepts**	
Compositionally and microstructurally sustainable alloy concepts	Alloy design by microstructure and not by chemistry; sustainability of existing alloy concepts and questioning of exiting chemical alloy specifications; composition-tuned alloys for a circular economy; hard-to-recycle versus easy-to-recycle alloys; chemically insensitive alloys; overalloyed versus lean alloys; alloys with high impurity content; reduction in number and variation of alloys; cross-over alloys; alloy design for circularity and recycling; compositionally robust alloys; sink alloys; cross-over and uni-alloys; microstructure-tuned alloys versus composition-tuned alloys; compositionally lean alloys; alloys born from scrap; dirty alloys; high-entropy alloys made from post-consumer scrap; recycling-oriented multicomponent alloys; alloys with resettable microstructures; self-healing alloys; alloys with microstructure-based repair mechanisms; new scrap class and scrap contamination by new alloy types; element accumulation in scrap and effects on alloys made from scrap; stainless steels with less Ni; effects of higher scrap- and ore-related impurity content on alloy properties
Alloy competition and alloy replacement	Copper versus aluminum as resistive conductors; compositionally lean and sustainable alloy concepts for hard and soft magnets; rare earth element-free magnets; replacement of precious/platinum group metals as catalysts, for instance by high-entropy alloys; nickel and cobalt replacement as electrode materials in batteries by manganese; replacement of lithium by sodium in batteries; manganese-containing stainless steels; low-alloyed maraging steels
**Leverage, potential impact and technology readiness**	
Magnitude of leverage (on greenhouse gas reduction, use of sustainable electrical energy, better energy efficiency, quantity)	Absolute quantities produced; specific CO_2_ emission per ton of metal produced; efficiency of effects to reduce greenhouse gas emissions, primarily CO_2_ emissions; opportunities for electrification of synthesis, production, heat treatment and downstream manufacturing; processes and mechanisms that allow reduction of the energy consumption in synthesis and downstream production processes; big numbers first: identification of which metals, alloys, products and processes have the largest leverage on sustainability improvement; use of green hydrogen in steel production; use of hydropower in Al production; secondary and tertiary production over primary production

a Not all of the topics listed in this table are discussed in this paper as the focus here is primarily on the materials science of synthesis aspects that help to reduce CO_2_ emissions and energy consumption.

## 4 Element- and Alloy-Specific Direct Sustainability Topics

### 4.1 Introduction to Element- and Alloy-Specific Sustainability Challenges

Sustainability research in metallurgy is confronted with very different types of challenges, potential leverage effects, and aims, depending on the metal and alloy class considered. Examples are the respective magnitude of the greenhouse gas emissions (absolute and per tonne of metal produced); impact of mining and tailings; potential for electrification of synthesis and processing; richness, dispersion and abundance of ores; or recycling rates, Figure 14.

**Figure 29:**
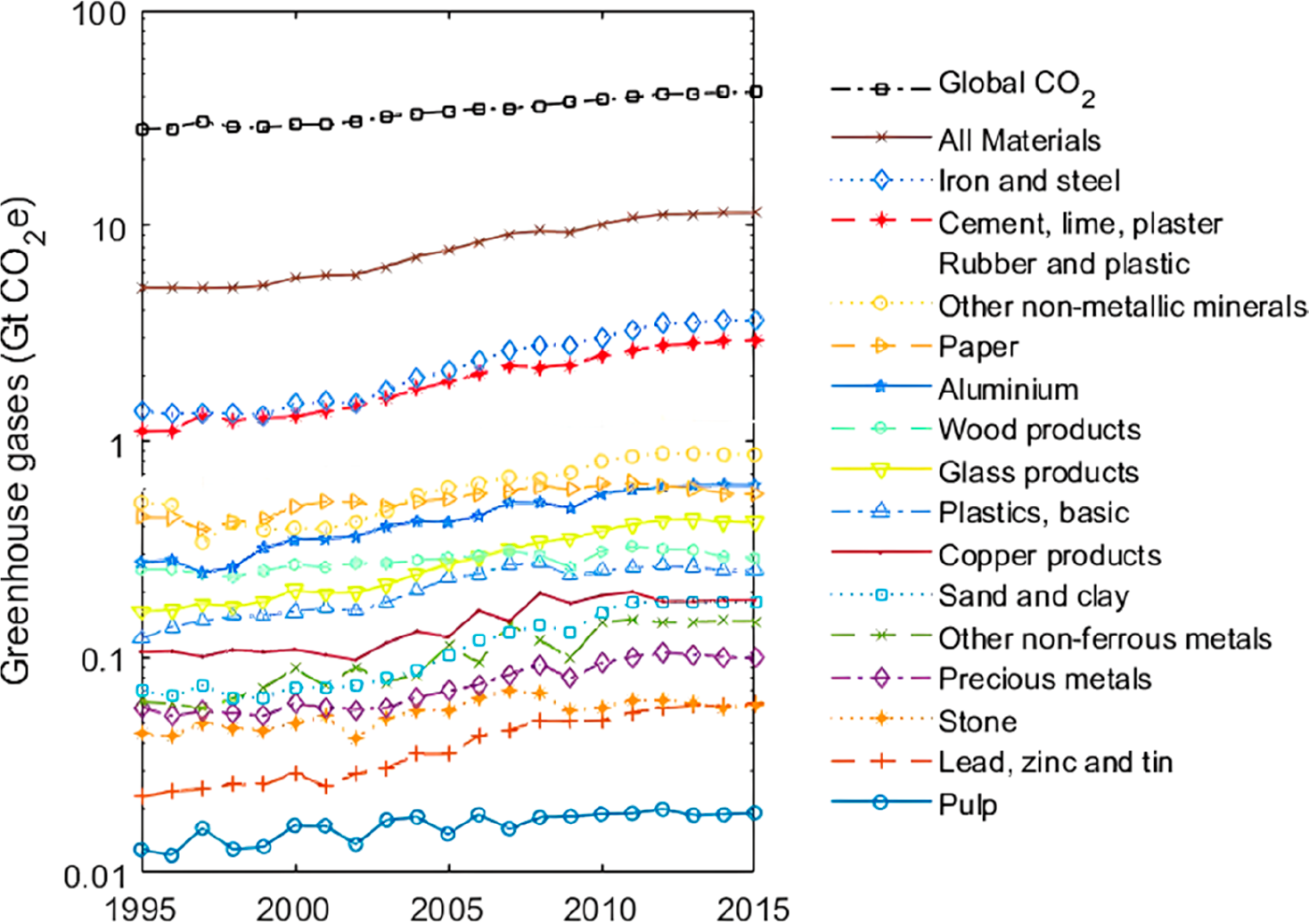
Emission of greenhouse gases caused by the manufacturing of materials from 1995 to 2015.^155^ The carbon footprint of the materials presented is calculated and compared to the global CO_2_ emissions (note the logarithmic scale). Emissions connected with the input of other materials are included. The figure is reproduced from ref (155) with permission. Copyright 2021, Nature. CO_2_e, carbon dioxide equivalent. Gt, gigatonnes.

Research in this field can therefore in principle address any aspect along the entire value chain, from mining, production through primary (mineral), secondary (scrap), or tertiary (re-mining) sources, or reducing of metal use in products and reuse, Figure 29–Figure 31. Metal-specific data on material flow, losses, scrap and final use were published in several overviews, for instance by using corresponding Sankey diagrams,^1,2,154^Figure 31 and Figure 32.

**Figure 30:**
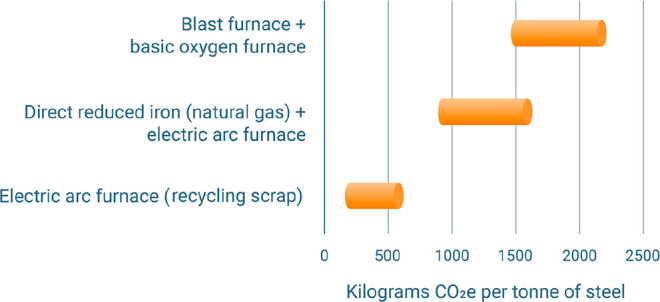
Ranges of CO_2_ emissions from steel making for different kinds of synthesis routes, presented by using data from the World Steel Association. CO_2_e, carbon dioxide equivalent.

**Figure 31:**
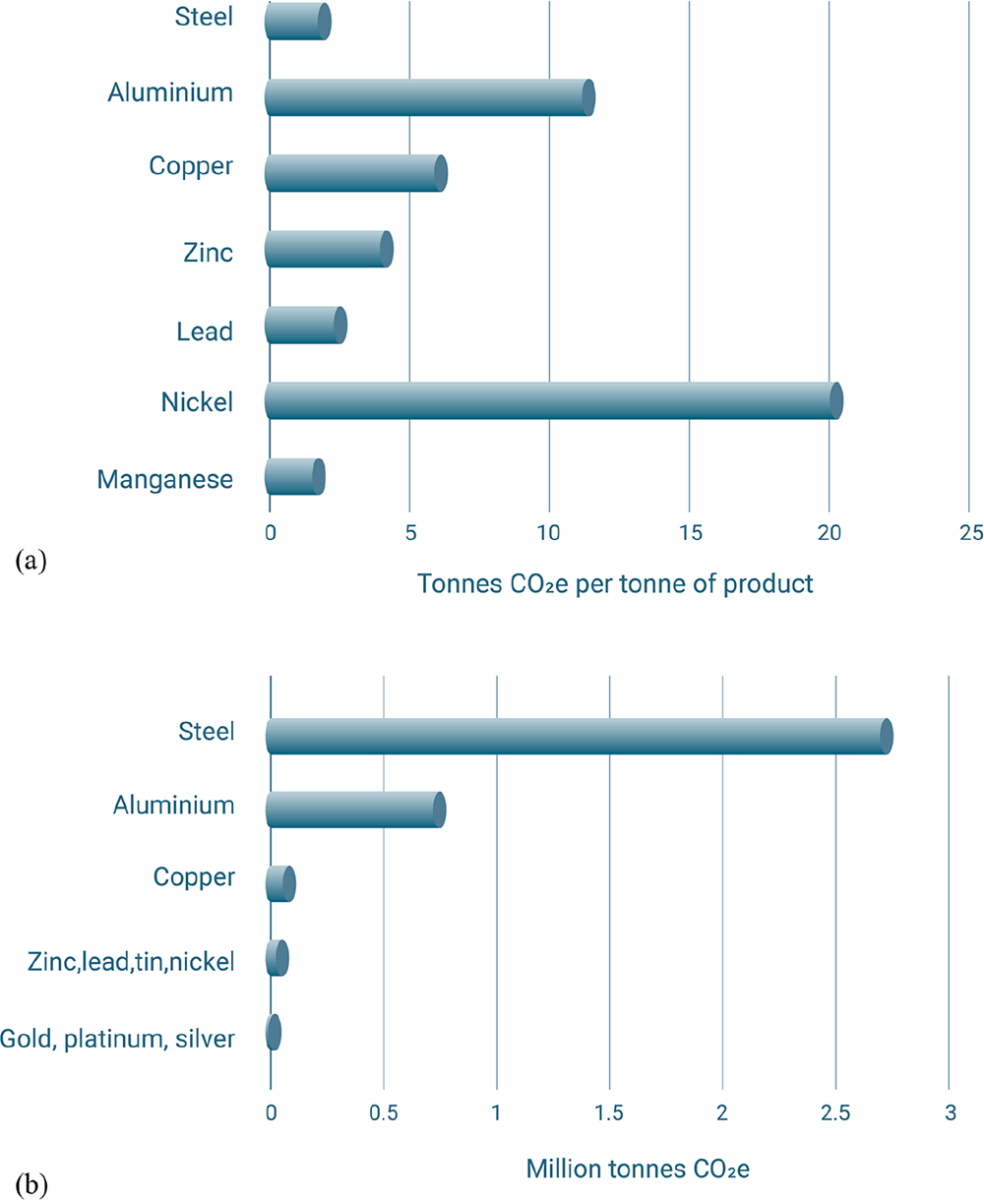
(a) CO_2_ emissions for different metals per tonne of product. (b) Total amount of CO_2_ emissions for different metals, scaled by their respective total production volumes. Note that the numbers can vary substantially when considering different (e.g., best practice vs worst practice) synthesis methods (see also Figure 30). Numbers are taken from the paper of Van der Voet et al.^88^ CO_2_e, carbon dioxide equivalent.

**Figure 32:**
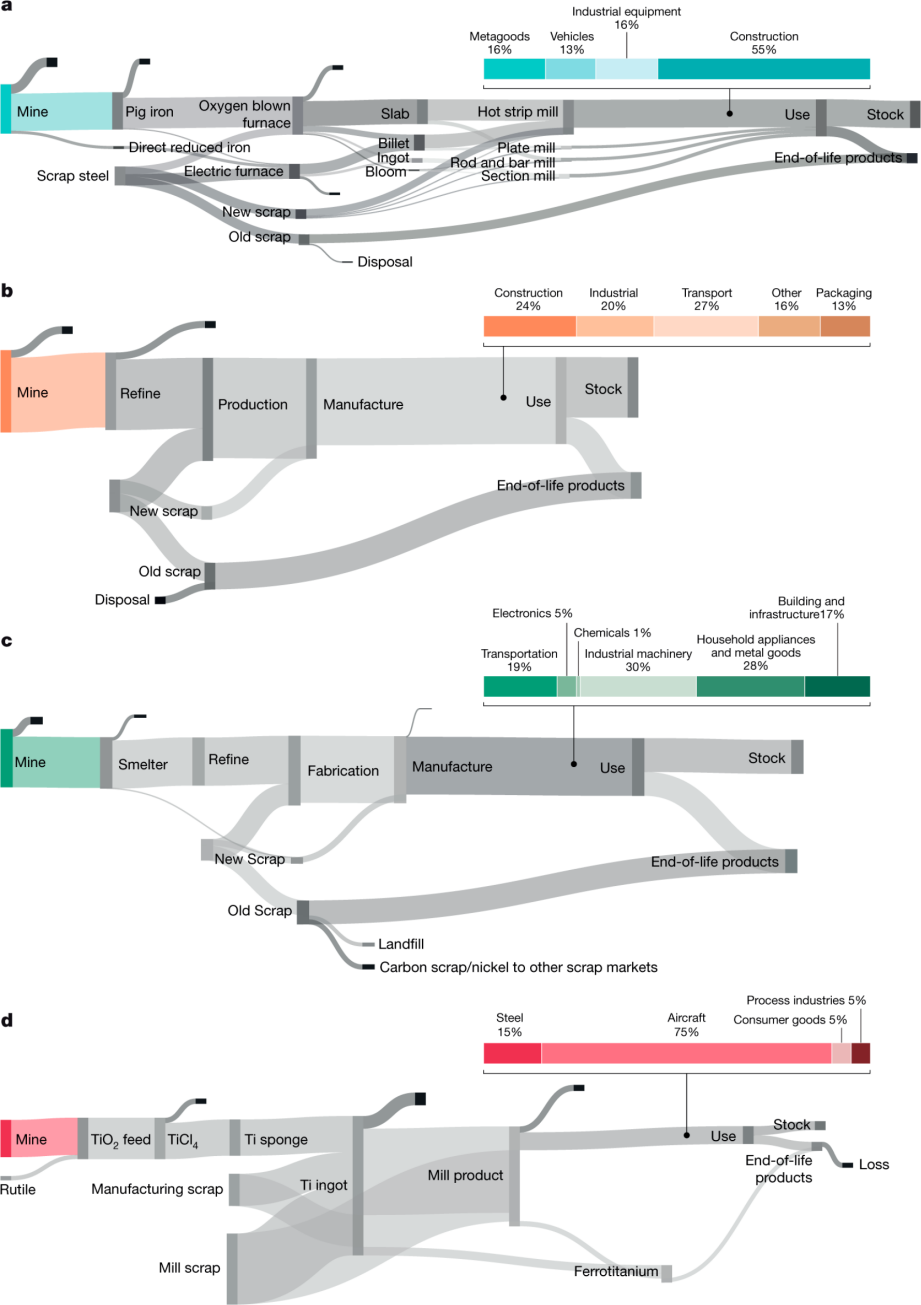
Metal-specific material flow data in the form of Sankey diagrams.^2^ The data are from the year 2017. (a) steel, (b) aluminum, (c) nickel, (d) titanium. The width of the flowchart arrows represents the quantities in terms of material weight. The figure is reproduced with permission from ref (2). Copyright 2019, Nature.

In metallurgical sustainability, size matters: a fully circular economy (i.e., based essentially on scrap recycling and on re-mining metals from dumped waste materials) works only if the metal in circulation matches the market demand for new metal, minus entropy-related losses.^156^ Currently, the ratio for many mass-produced metals is in average only about 1/3^rd^ made from scrap to 2/3^rd^ made from mineral feedstock, Figure 16. The latter contribution produces by far the highest amount of emissions, tailings and waste, which qualifies this sector as a prime target for sustainability research. This means that today the majority of the metal production comes from mineral sources, which causes the largest environmental burden in the overall metallurgical values’ chains. Within each alloy group the CO_2_ emissions differ substantially, depending on the specific reduction methods employed, Figure 29 and Figure 30.

**Figure 33:**
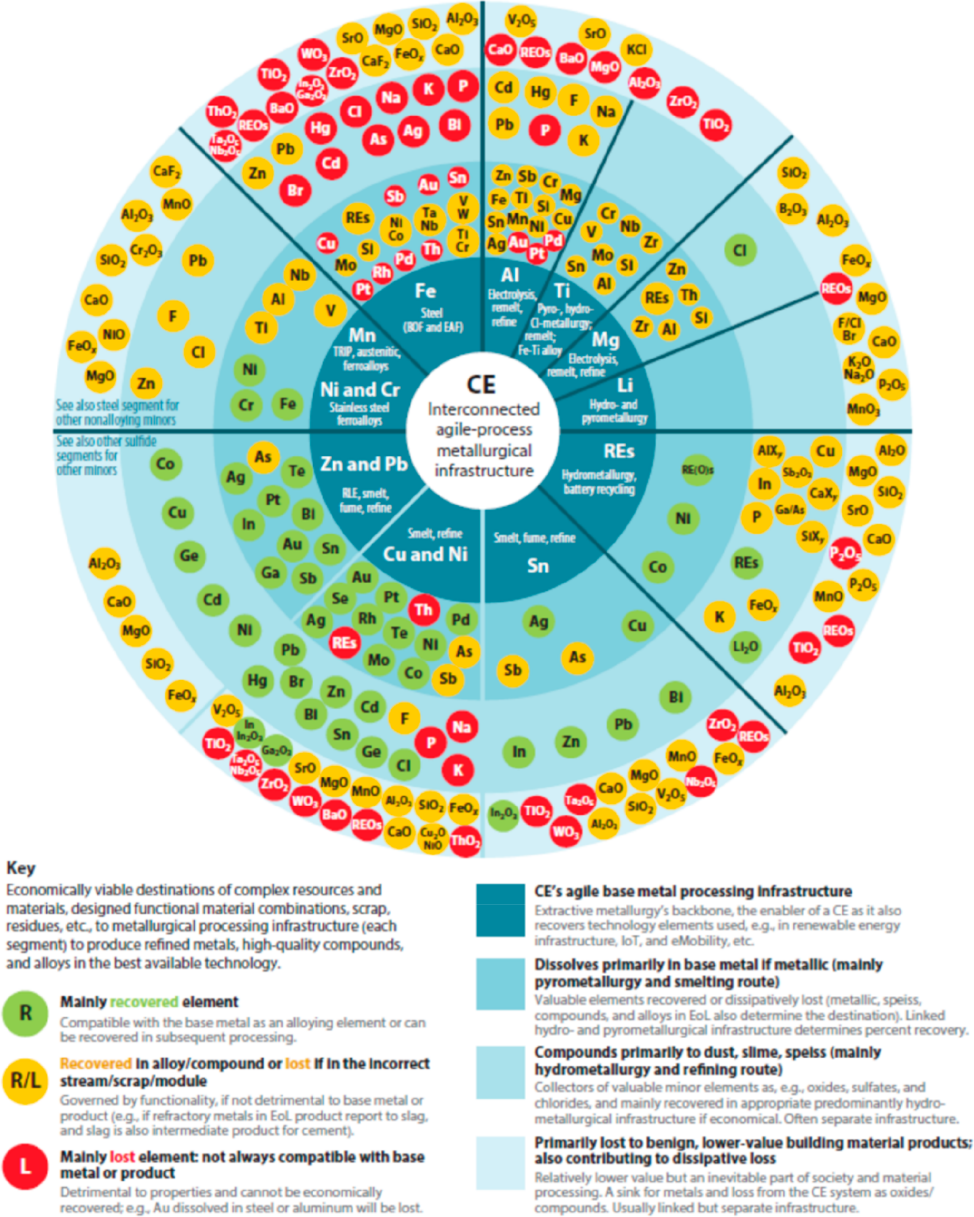
Different recycling challenges for different base elements and base alloy groups (see the inner ring) according to a classification suggestion of Reuter et al.^21,67^ The “Metal Wheel” shows groups of metallic elements and some of their oxides with respect to their joint appearance in products and waste streams together with metal-group-specific refinement and recovery techniques.^156^ BOF, basic oxygen furnace; CE, circular economy; EAF, electric arc furnace; EoL, end of life; IoT, Internet of things; REOs, rare earth oxides; Res, rare earth elements; RLE, roast leach electrowinning; TRIP, transformation-induced plasticity steels. The figure is reproduced with permission from ref (11). Copyright 2019, Annual Reviews, Inc.

**Figure 34:**
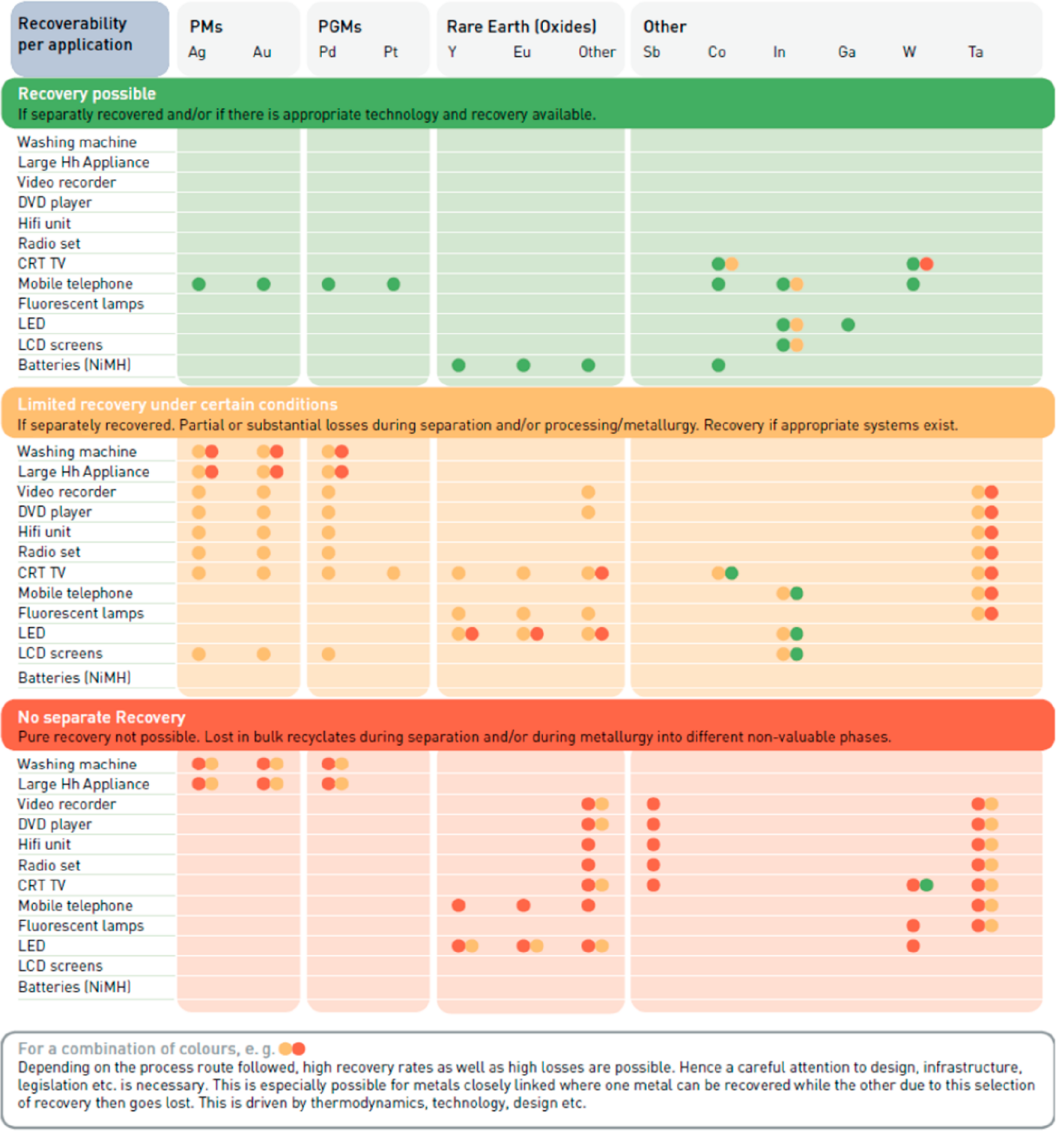
Metals that can be recycled from several types of products and metals that are hard to recover.^156^ The figure is reproduced with permission from ref (156). Copyright 2013, UN Environment Programme UNEP.

This means that for the different metals, potential high-leverage research directions can be grouped (in absolute or mass-normalized numbers) along categories such as the absolute quantities produced (e.g., Fe, Al); greenhouse gases emitted (e.g., Fe, Al, Ti, Ni, Co, Mn, Si); energy demand by mass-produced (Ti, Fe, Mg, Al); low vs high end of life recycling rates (e.g., Pt group metals vs Al alloys); waste and new (runaround) scrap generated during production and manufacturing (Ti, Al, Fe); bulk availability of scrap (Fe, Al); electrical, electronic and nanoscrap (Cu, Au, Ag, RE); high vs low recovery grade (e.g., Pb, certain Al alloys, stainless steels); closed loop recycling (specific alloy to alloy recycling) vs open loop recycling (sorting required); environmental harm caused by mining, by-products and tailings (Al, red mud; Cu, arsenic; Au, mercury); strategic relevance and mineral scarcity (e.g., rare earth metals, Pt group metals, Au, Cu); as well as social and labor standards associated with production (Pt group metals, Co, Ni, Cu), Table 6. Other metallurgical sustainability classification schemes, grouped along recycling-, recovery rates-, element-, and method-oriented categories, with particular emphasis on the circular metals economy, have been suggested by Reuter et al.,^21^Figure 33 and Figure 34. A few categories according to their work have been summarized in Table 7.

**Table 7:** Metal- and Alloy-Specific Metallurgical Sustainability Classification Criteria According to Reuter et al.^21^

Category	Features
Group 1	Metals that can be extracted from mineral sources using established processing pathways on available industrial metallurgical infrastructures. These include well-established extractive metallurgy processing techniques. The authors refer to them as “backbone technology” and see them as crucial enablers of a circular metallurgical economy.
Group 2	Metallic elements that dissolve preferentially in other base metals. They can be recovered in principle via pyrometallurgical techniques and specifically via smelting. Currently, the authors included both valuable elements that have been recovered and those that have been lost into this category. The authors suggest that advances in hydro- and pyrometallurgical technologies can boost recovery rates in this area.
Group 3	Here the authors include compounds and valuable minor elements that are mostly found in dust, slime, and other residual forms and that can be collected, sorted and refined hydrometallurgically.
Group 4	Elements that are predominantly lost as oxides to lower-value material products. These elements are usually not recovered. Metals in this group also include entropy-related losses, which mark, besides global market growth, a natural limit of a fully circular economy.

In the following sections some element- and alloy-specific aspects are discussed in more detail, placing focus on opportunities for basic research with high leverage along the sustainability categories mentioned above. The following sections aim primarily at identifying research tasks for the different alloy classes with potentially high leverage for sustainability along the categories mentioned above. The specific research opportunities that arise from this are then discussed in more detail in specific sections later, addressing particularly feedstock types, extraction methods, alloy design measures, etc. The assignment is not to give an overview over all the possible synthesis and processing methods for these materials, as this has been covered by previous textbooks and overview papers, but to focus on topics with high potential for reduced energy consumption and greenhouse gas emissions.

### 4.2 Sustainability-Related Research Topics in Iron and Steel Production

**Figure 35:**
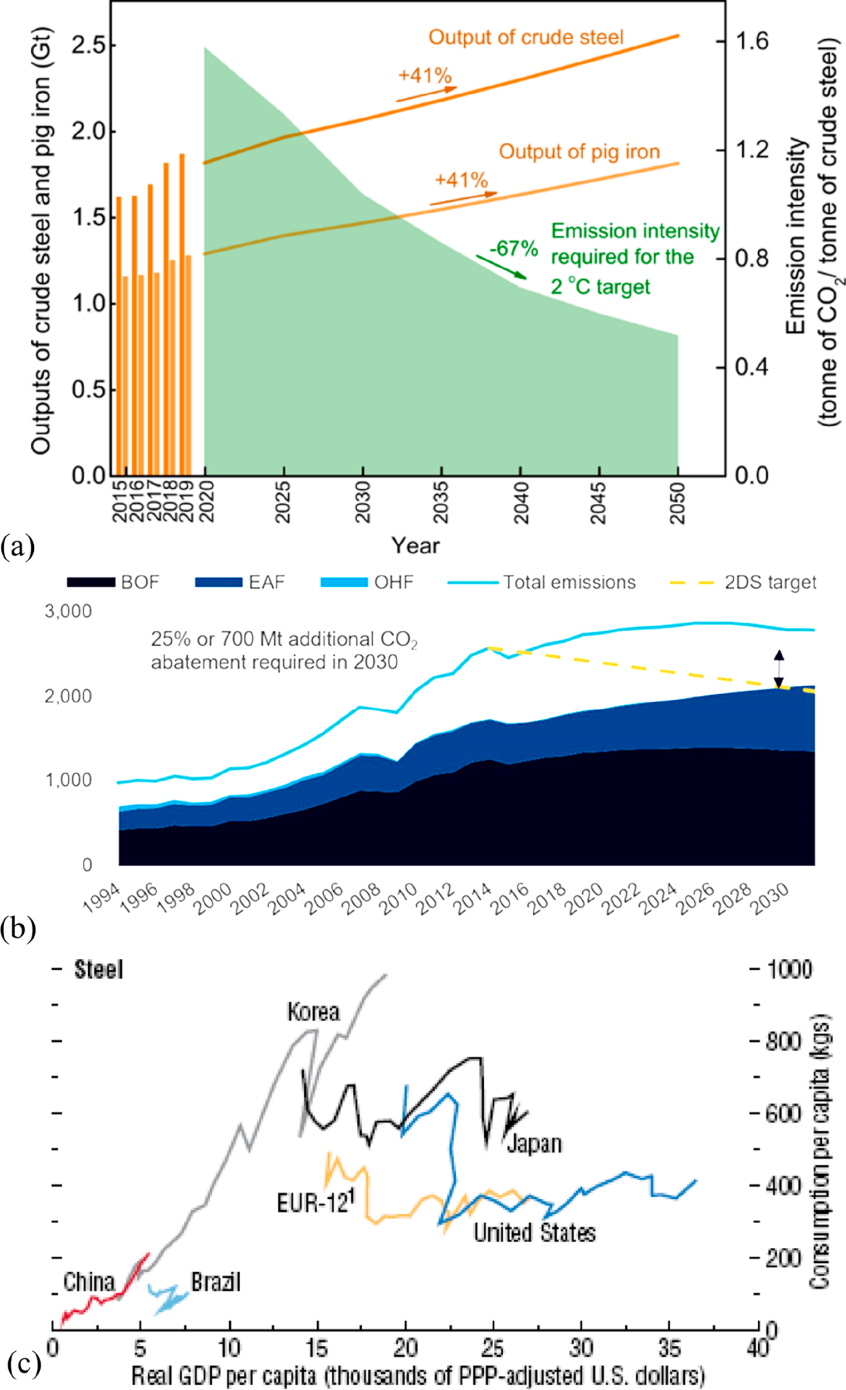
(a) Production of pig iron, steel, and CO_2_ emission intensity. (b) Total CO_2_ emissions from steel making. (c) Global per capita consumption of steel (data taken from IMF World Economic Outlook). Gt, Gigatonnes; BOF, Basic Oxygen Converter; EAF, electric arc furnace; OHF, open hearth furnace; 2DS, 2 °C target fixed by the Paris Agreement. Part (a) is reproduced with permission from ref (157). Copyright 2022, Nature. Parts (b) and (c) are reproduced with permission from ref (158). Copyright 2010, The Open University, Milton Keynes, U.K.

Steel is the most widely used and most important metallic material since more than 3 millennia, in terms of volume produced and application scope, Figure 6. It is also the most important alloy in terms of feedstock quantity, reductants consumed, greenhouse gas emitted, and energy used, Figure 29. Steel is therefore by far the most essential and impactful alloy class when it comes to sustainability, qualifying it as a top candidate material for basic research, Figure 35 (see scientific aspects in sections 6.2.2–6.2.5, 6.3.2–6.3.5, 7.4.2–7.4.8, and 7.5). Different from metals like copper and nickel, which are needed for electrification, steel consumption does not grow monotonously with a region’s gross domestic product but reaches a plateau, particularly for wealthy societies in their postindustrial phase. In emerging and growing economies, however, the increase in steel consumption is strongly coupled to the gross domestic product, so that these regions will drive the growth in the next decades, Figure 35.

The expected market growth for steel in the coming decades is not limited by a lack of traditional feedstock materials (as for instance needed for blast furnace operation), as sufficient supplies of ores and coal are available on the market. Instead, the targeted global transformation of steel making, as the largest single producer of greenhouse gases, into a more sustainable industry, is rather limited by the availability of sustainable and non-fossil reductants, such as green hydrogen or green ammonia, and by the renewable electrical energy that is needed to make these reductants and/or to operate the electric arc furnaces in case of secondary synthesis. The availability of sustainable electrical energy is currently growing faster than the availability of sustainably produced hydrogen, opening up a window of opportunity for directly using sustainable electricity in iron and steel production. This fits better to the current market development and could also have an altogether slightly better total efficiency and CO_2_ balance, depending on the reduction technique that is used.

The development and upscaling of feedstock-, energy-, and hydrogen-efficient metallurgical extraction reactors is currently hampered and slowed down by insufficient understanding of some of the elementary underlying transport-, reduction-, and purity-related mechanisms governing the efficiency of new sustainable synthesis routes and the quality of the steel produced that way. Introduction of new metallurgical extraction methods can also offer the opportunity to widen the spectrum of feedstock. Some established and often expensive feedstock types commonly used today have been developed for very specific aggregates such as blast furnace iron oxide pellets, methane-based iron oxide pellets, etc., but new reduction methods might be suited to make use of so far less used and less expensive raw materials.

This brings up additional new research questions such as the role of the different oxide types and their respective impurity contents; chemo-mechanical size effects of the feedstock (fines, pellets of different size); the abrasion and sticking behavior of the pellets or fines; the heterogeneity of the reduction reactions; the influence of gas pressure and temperature; chemical composition and impurity content of the green iron; use of plasma excitation in both solid-state and liquid-state reduction; or the influence of mixed reduction gases containing, for instance, methane, ammonia and hydrogen in different fractions, to name but a few interesting aspects for basic research in this field. Reduction methods that make direct use of electricity such as electrolysis (see details in section 7.7.5) and reduction in electric arc furnaces (see details in sections 5.7, 5.8, and 7.5) are confronted with other research questions such as the electrode reactions, electrode lifetimes, electrode emissions and decay, or all the scientific details associated with plasma reduction smelting for instance.^159−161^

Another important aspect for steels is the growing role and market of scrap for steel making. Steel, like most other metals, can be recycled infinitely. With an average global recycling rate of ∼70%, it is actually the most recycled material on the planet.^1,11,162^ The use of steel scrap as feedstock (secondary synthesis) instead of minerals (primary synthesis) is even expected to substantially increase, from about one-third worldwide today, to more than two-thirds in 2050, Figure 36. This means that research in sustainable steel making must also target secondary synthesis. Interesting options also lie in the study of hybrid synthesis pathways that can make use of mixed charging consisting for instance of traditional high-purity ores, low-quality ores, scrap, and re-mined tertiary resources that had already been dumped as mineral-rich waste from other processes.

**Figure 36:**
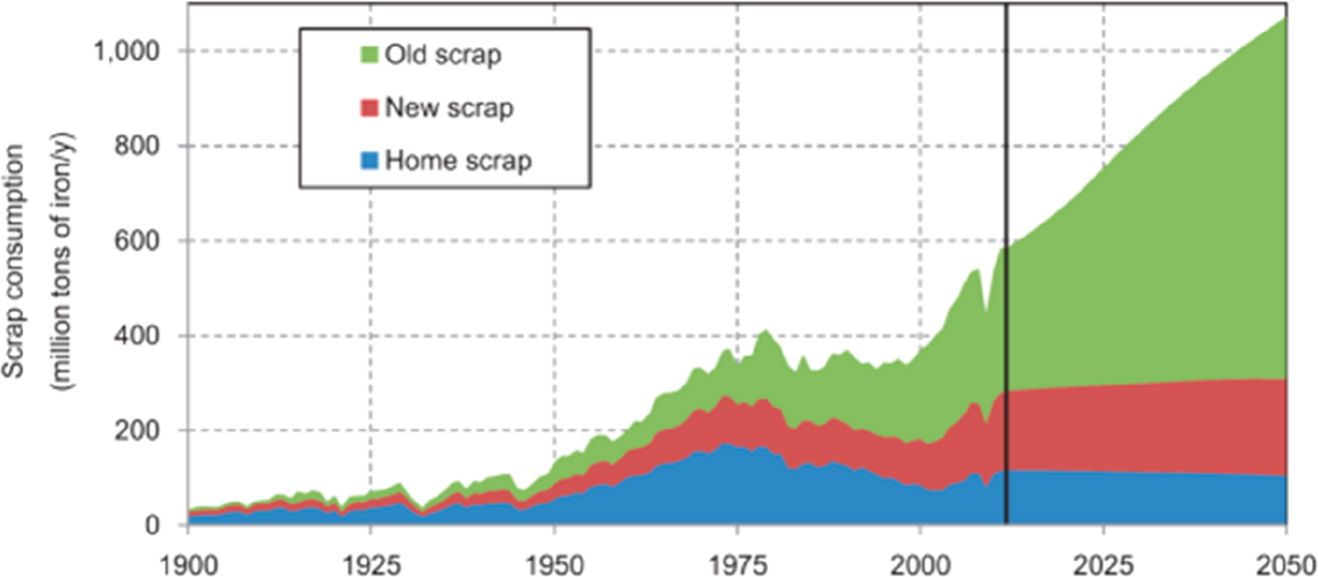
Estimated future development of the global steel scrap market.^165^ The more intense use of steel scrap in the future is the most efficient and fastest pathway toward rapid decarburization of the global steel production, at least to about 2/3^rds^ of the total production, a target that can be probably reached by the years 2050–2060. The authors distinguish between old scrap, new scrap, and home scrap. Old scrap is mixed scrap from end-of-life products (also called obsolete scrap). New scrap is generated along the manufacturing chain prior to use by an end customer (sometimes also called prime or prompt scrap). Home scrap is the material which is internally generated at a company site during the production of the new steel (in steel mills, steel foundries, etc.). Home scrap usually remains on site for recycling and does not leave the steel making area. Note that there may be several slightly different definitions in the literature. The figure is reproduced with permission from ref (165). Copyright 2013, Elsevier.

A considerable challenge in this context is that the use of higher scrap levels may result in the introduction of undesirable impurity levels into the steel.^163−165^ This leads to the question of how to remove or tolerate the multiple impurity elements that enter the steels from scrap. Some elements intruding from scrap are particularly harmful (such as Cu, Pb, Sn and Zn), potentially affecting the performance of advanced high-strength steels in the future.^166^

**Figure 37:**
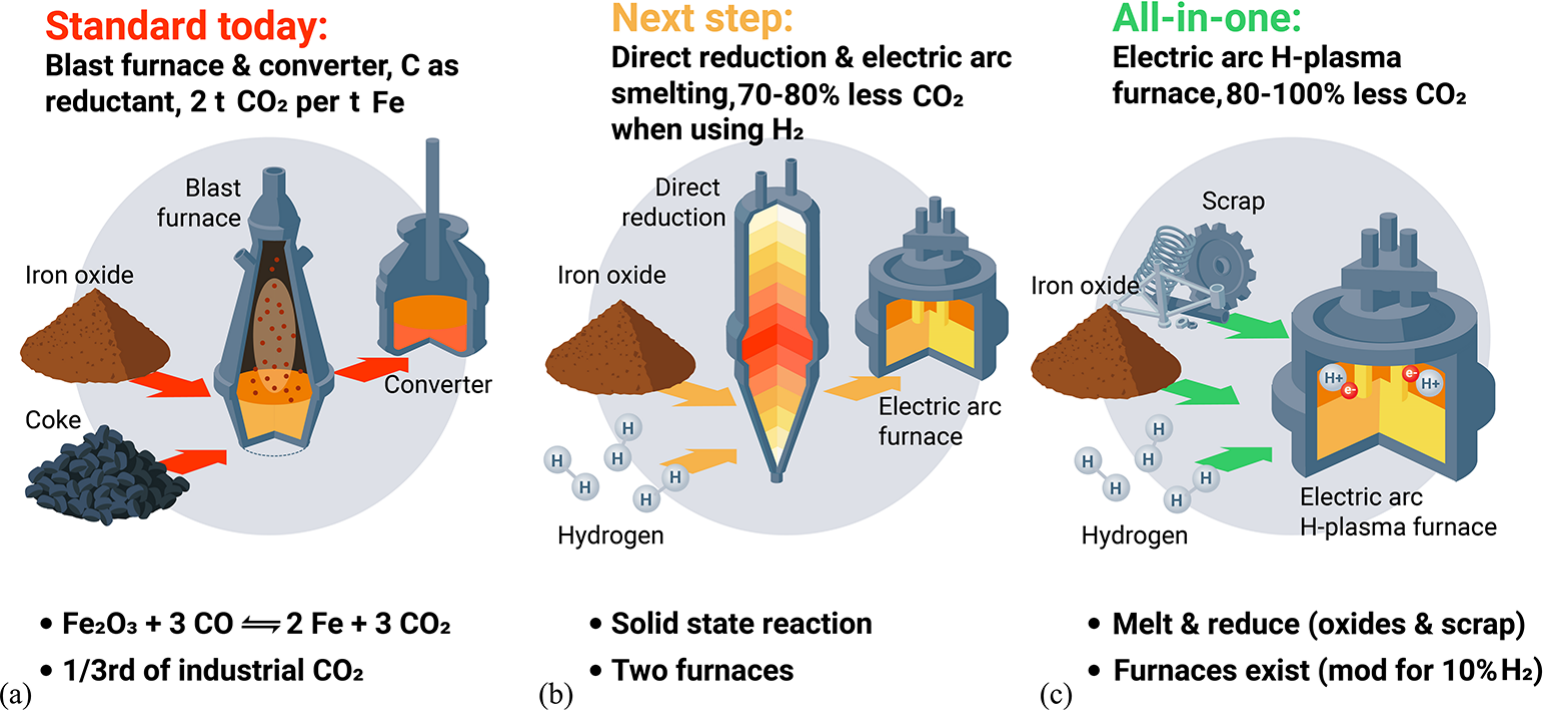
(a) Steel making through the classical blast furnace and downstream basic oxygen converter route with the use of CO as reductant in the blast furnace, producing ∼1.9–2.2 tonnes CO_2_ per ton of steel produced. This qualifies the blast furnace process as the largest single CO_2_ emitter on the globe. (b) Direct reduction: solid-state reduction of hematite (or magnetite) with either methane or hydrogen (or reductant mixtures). The hydrogen-based direct reduction variant is capable of reducing the carbon footprint of iron making by 70−80% when using hydrogen from renewable production. (c) Reductant-containing plasma (e.g., hydrogen-based plasma) atmospheres in electric arc furnaces used for liquid-state oxide reduction (also called hydrogen plasma smelting). This furnace could also be additionally charged with steel scrap. This means that plasma reduction offers an all-in-one process of melting, mixing, and liquid-state reduction.

However, it is also clear that scrap alone cannot satisfy all of the future demand.^167^ Steel products, particularly in buildings, machines, and vehicles, have often very high longevity, so that more than 75% of all steel ever made is still in use. This means that today only ∼1/3^rd^ of the production can come from recycling, yet with a growing trend. Hence, fresh steel must be produced in huge quantities, for a rapidly growing global market, Figure 6. This new material is won from mineral ores. These are usually oxides, mainly hematite (Fe_2_O_3_). The synthesis of iron from them is called iron making. It proceeds mostly through reduction of iron oxide ores in blast furnaces. In this process CO serves as reductant, provided via the Boudouard reaction from the CO_2_ that is produced by burning the coke that is charged into the furnace. This traditional blast process currently operates at the staggering annual production amount of 1.3 billion tonnes^168^ (∼70% of the global production), Figure 37.

The near-eutectic Fe-C alloy tapped from conventional blast furnaces inherits a very high C content (∼4–4.5 wt %). This so-called pig-iron must be refined into steel, by reducing its high C content, usually to values far below 1 wt %.^28^ This is done in a basic oxygen converter where O_2_ is blown into liquid iron to bind the C in CO_2_. This converter steel can be compositionally adjusted by alloying, usually with C, Mn, Cr, Si, etc. This means that a large amount of C is first used to extract iron from its ore in the blast furnace and then removed again from that Fe-C raw material by use of oxygen in the converter. Therefore, every ton of steel produced generates in global average ∼1.9–2.2 tonnes of CO_2_, Figure 35.

This qualifies steel making as the largest single greenhouse gas emitter on earth,^162^ with ∼8% of all emissions (∼35–40% in the industrial sector). CO_2_ storage as a mitigation strategy is not a long-term sustainable solution because the available volume to store all CO_2_ from the steel industry satisfies only a few percent of the total demand and leakage from such caverns might harm soil and water.^169^ These staggering CO_2_ volumes can be drastically cut only by disruptive changes, such as using non-fossil reductants and electrification in primary synthesis and the use of higher scrap fractions in secondary synthesis.

**Figure 38:**
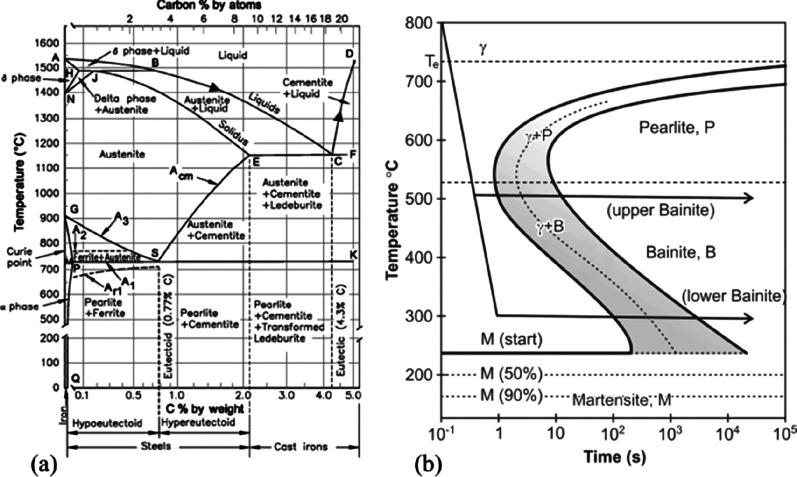
(a) Metastable part of the Fe-C phase diagram. (b) Time–temperature-transition diagram that shows some of the types of microstructures that are accessible when kinetic effects during cooling and thermomechanical processing are used, producing several types of nonequilibrium microstructure features. The diagrams thus highlight some of the microstructures that can be used to produce several hundreds of types of Fe-C steels by exploiting different heat treatment and deformation pathways. These many nonequilibrium microstructure variants make steel the most versatile and most recycled material in human history.

**Figure 39:**
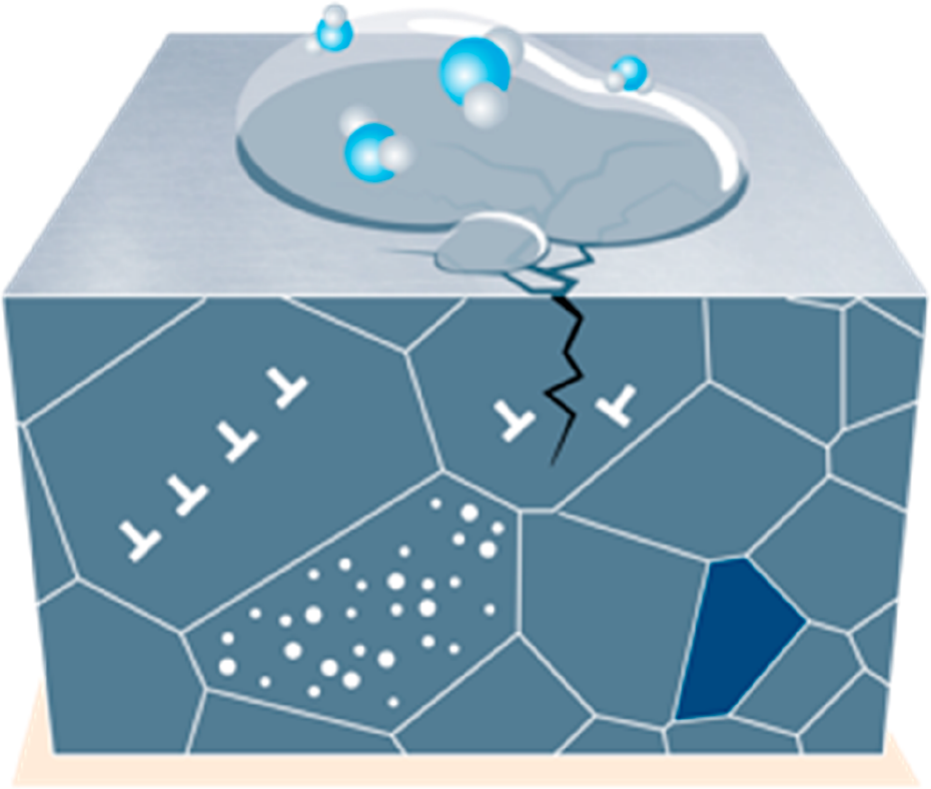
One approach to enhance the sustainability of metallic materials can be to replace chemical (compositional) complexity in composition-dominated alloys (such as high-entropy alloys, hard magnets, superalloys, stainless steels or high-strength aluminum alloys) to some extent by microstructural complexity (at lean chemical composition). In the latter approach processing gains higher momentum, as many of the requested properties of alloys can in part be achieved by equipping the materials with a range of lattice defects (such as dislocations, interfaces, second phases, etc.)^166,172^ instead of only through significant chemical changes.

Another important sustainability-related feature of steel is that a variety of alloy variants can be produced from the simple quaternary chemical system iron-carbon-manganese-silicon. Hundreds of different types of steel can be achieved using essentially these 4 elements, covering a wide range of properties, and some even using only iron and carbon, Figure 38. This is made possible by the many nonequilibrium phase transformations and the resulting microstructure variants of steels (examples are pearlite, bainite, martensite, and so on). This principle follows an alloy design philosophy that can be termed “microstructural complexity from chemical simplicity”. It is an approach to design high-volume alloys with particularly high sustainability. It implies that such “chemically simple” steels do not differ much from each other in terms of their chemical composition but only in terms of their microstructure and properties, Figure 39.^166,170,171^ While the former feature is a conserved quantity, the latter features are not. This circumstance qualifies chemically lean steels on the one hand as ideal (i.e., chemically well-matching) donor materials for melting new alloys from their scrap and on the other hand as acceptor materials that can be produced by melting down from such chemically related steels. This makes plain carbon steels an ideal recycling material, in conjunction with the significant advantage that this alloys class is produced in large volumes, which at some point return to the market as scrap (see sections 6.3.2–6.3.5 and 7.4.8).

Nonetheless, an emergent challenge lies in the fact that also steel scrap must be increasingly subjected to composition-dependent sorting, for separation of the large steel groups such as Fe-C, medium-manganese-, maraging-, chromium-, and nickel-containing steels, etc. (see sections 6.3.2–6.3.5 and 7.4.8).

What makes steel in general a topic of eminent importance in metallurgical sustainability research is surely that every—even tiny—step in improved understanding of fossil-free synthesis and smelting methods has a potentially huge leverage on the mitigation of greenhouse gas emissions. Revolutionizing steel production is one of the largest single attack points to mitigate global warming. On the other hand, it is absolutely clear that steel cannot be dispensed with in the future, but its demand will instead continue to increase dramatically, not only due to infrastructure and construction but also, in the worst case, due to the necessary protection of coastal areas against the rising floods, a measure that can only be coped with by huge amounts of concrete and steel.

**Figure 40:**
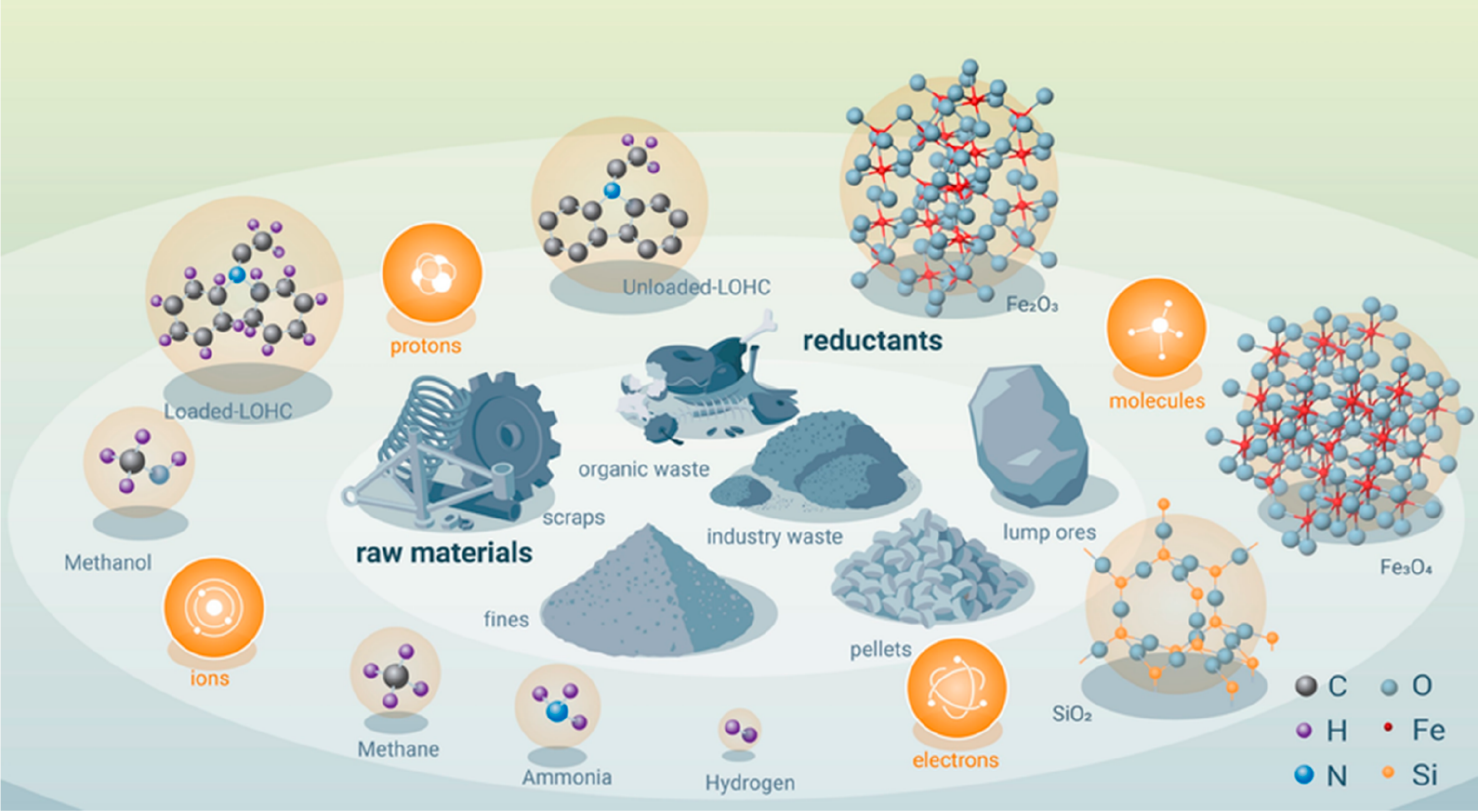
Possible combinations for reducing lump ores, pellets, or fines, using several types of reductants, aggregate states, and reaction principles. Possible iron carrier feedstock materials could be fines (fine ores sized ∼30–250 μm, used for example in fluid beds), pellets (sintered polycrystalline oxide granulates made from fines, sized about 1–1.2 cm in diameter, for use in static direct reduction shaft furnaces and blast furnaces), lump ores (bulk ores ranging ∼5 mm–15 cm), and scrap (used steel which has remained in products between weeks and up to 100 years) suited for remelting. The currently mostly used ore types are hematite (Fe_2_O_3_: most frequently used iron ore) and magnetite (Fe_3_O_4_ iron ore). Also shown are several types of reductant states, including ions and electrons (electrolysis), protons (hydrogen-based plasma reduction), and molecules (direct reduction) as well as different types of hydrogen carrier molecules, that can serve as sustainable reductants, when produced with green hydrogen.

**Figure 41:**
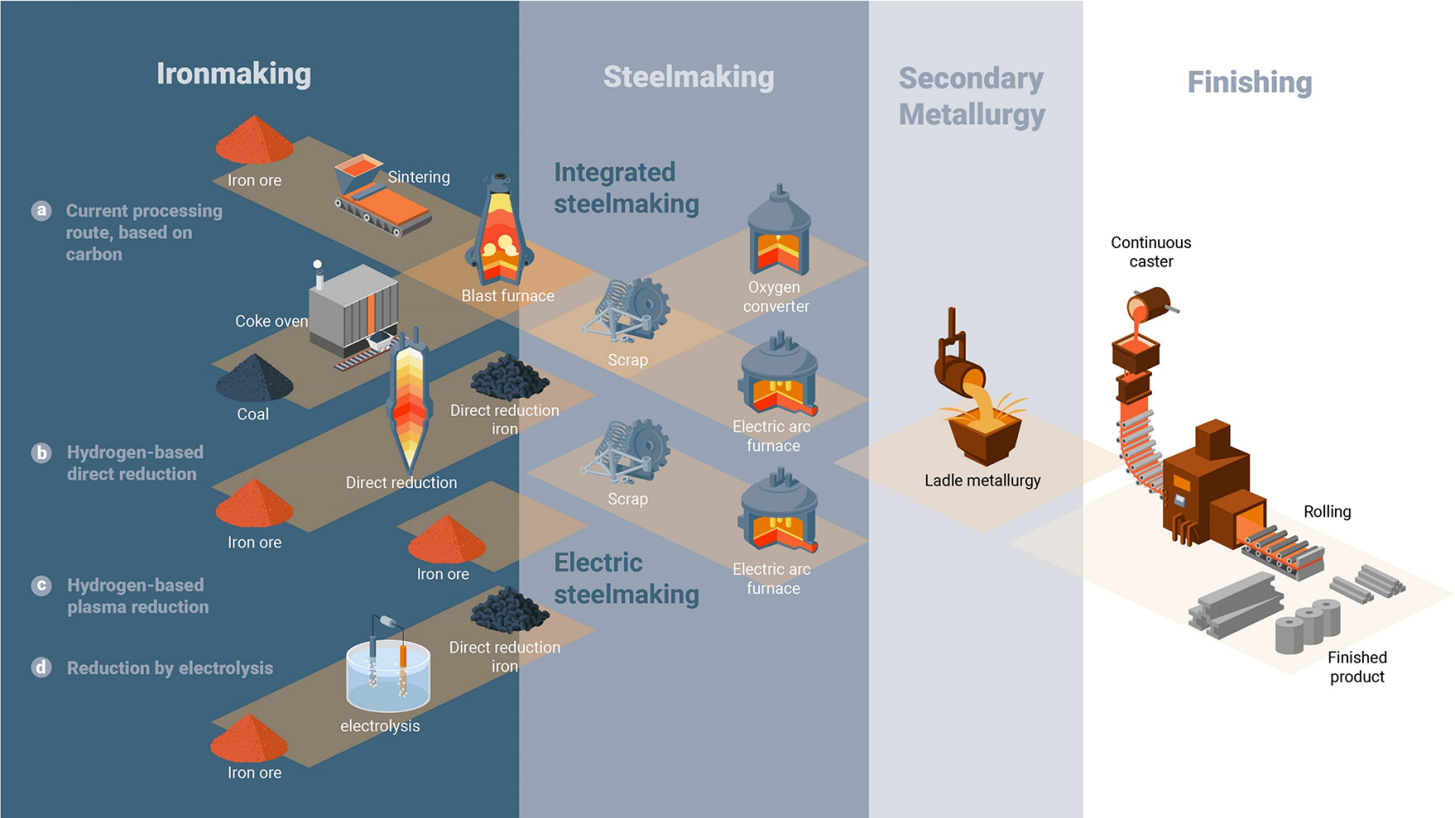
Different pathways for steel making, highlighting several types of reduction reactors and downstream processing pathways, including the current steel making practice (a) and also several processing variants for more sustainable steel making (b, c, d).

Basic metallurgical research topics regarding the production of steel with less or no CO_2_ emissions through non-fossil reductants and the use of electricity will be addressed in detail in dedicated subsections below. Figure 40, Figure 41, and Table 8 present several possible research areas in this field: Figure 40 gives an overview over different combinations of feedstock, reductants, and aggregate states. It shows different types of iron carriers (fine ores, lump ores of different grade, different types of scrap, etc.), carbon-free reductants (in the form of solids, molecules (H_2_, NH_3_, etc.), ions, protons, electrons), while Figure 41 shows several types of reduction reactors and downstream processing pathways, including also several processing variants for sustainable steel making.^2,108,128,173−179^ The most important ones are the blast furnace where coke-based CO reduces iron ores to a near-eutectic Fe-C alloy; the shaft furnace for direct reduction, in which solid-state cm-sized ores or pellets are in static piles exposed to a gaseous reductant; fluidized bed or rotary furnaces where fines of sub-mm-sized ore particles are as dynamic agglomerates (particles in motion) exposed to a gaseous reductant; and the electric arc furnace, which is a standard industry furnace with an electric arc ignited between electrodes and solids (today usually scrap) or liquids. The electric arc can turn the gas atmosphere inside the furnace into a plasma. A plasma is a reactive and electrically charged gas-like substance consisting of molecules, atoms, ions and free electrons. Finally, electrolysis is also a promising approach, where oxygen is removed from liquid oxides by electricity.

**Table 8:** Opportunities for Basic Metallurgical Research Related to Sustainable Iron Production, with Potentially High Leverage on Improved Sustainability

**Process- and mechanism-related research on sustainable iron production**
Primary synthesis: All measures that enable reduction of carbon emissions in pig iron production; replacement of blast furnace and basic oxygen converter technologies by hydrogen- and/or methane-based direction reduction, plasma reduction, and electrowinning methods; basic transport mechanisms in direct reduction; phase transformation mechanisms; reduction and purity-related mechanisms governing the efficiency of new sustainable synthesis routes and the quality of the steel; different oxide types; impurity content of feedstock; element partitioning between reductants and iron; low-price feedstock (for instance Si-containing ores/banded ores); variable feedstock and mixed ores and scrap smelting plus reduction methods; chemo-mechanical size effects of the feedstock (fines, pellets of different size); heterogeneity of direct reduction reactions; influence of reduction gas pressure and temperature; chemical composition and impurity content of the iron; use of plasma excitation in both solid-state and liquid-state reduction; influence of mixed reduction gases; electrode reactions; electrode lifetimes; plasma metallurgical reduction reactions; thermodynamics of direct reduction; use of renewable energy sources in iron production (particularly in case of endothermic redox reactions); use of renewable electrical energy for all reheating steps along the production chain; efficiency increase in smelting and reduction techniques; use of moderate amounts of biomass and waste polymers as feedstock; exploration of new iron ore deposits with low environmental impact
Secondary synthesis: Improved scrap collection and sorting; separation of steels with higher manganese content from carbon steels and stainless steels in scrap collection; removal of copper, zinc, lead and related accumulating tramp elements from scrap-based melts; increase in the fraction of scrap used in the basic oxygen converter route
Tertiary synthesis: Re-mining of iron via extraction from dumped waste materials; use of slags from steel making; refinement and ladle metallurgy as feedstock for other materials and processes; use of red mud for steel making

Some advantages and disadvantages apply regarding the many different types of reduction pathways, and the specific scientific research challenges behind these technologies will be discussed in more detail in the ensuing sections.

**Figure 42:**
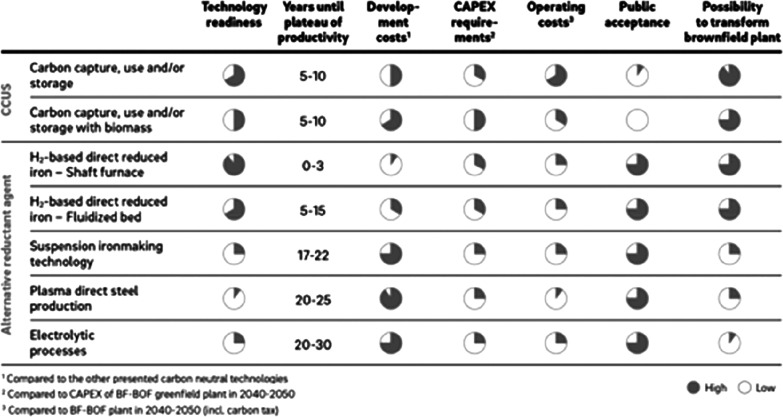
Comparison of different steel making technologies with respect to technology readiness and sustainability, using currently available engineering solutions.^180^ It must be considered, however, that such evaluations do usually not consider opportunities from more recent disruptive technologies with lower technology readiness level. CCUS, carbon capture and storage. CAPEX, Capital Expenditures (referring to the magnitude of the investments required to realize a certain technology). The figure is reproduced with permission from ref (180). Copyright 2020, Roland Berger GmbH.

Identifying the most promising production pathways based on the disruptive mechanism combinations with highest leverage for the reduction in greenhouse gas emissions requires consideration of four main aspects, namely, (1) which of these reduction workflows has potential for large-scale practical industrial application (i.e., billion ton scale); (2) which are the main scientific questions and bottleneck problems that require attention from materials science to arrive at a better understanding of the rate-controlling mechanisms, for more efficient industry upscaling; (3) which are the reduction and production processes that allow optimal exploitation and maximum direct use of renewable electrical energy (in order to realize processes with highest overall efficiency and least consumption of sustainable reductants); and (4) how can the green hydrogen and its carrier reductants be used in the most efficient way (as the availability of this reductant will remain the major bottleneck in green steel making in the coming decades), Figure 41 and Figure 42.

**Figure 43:**
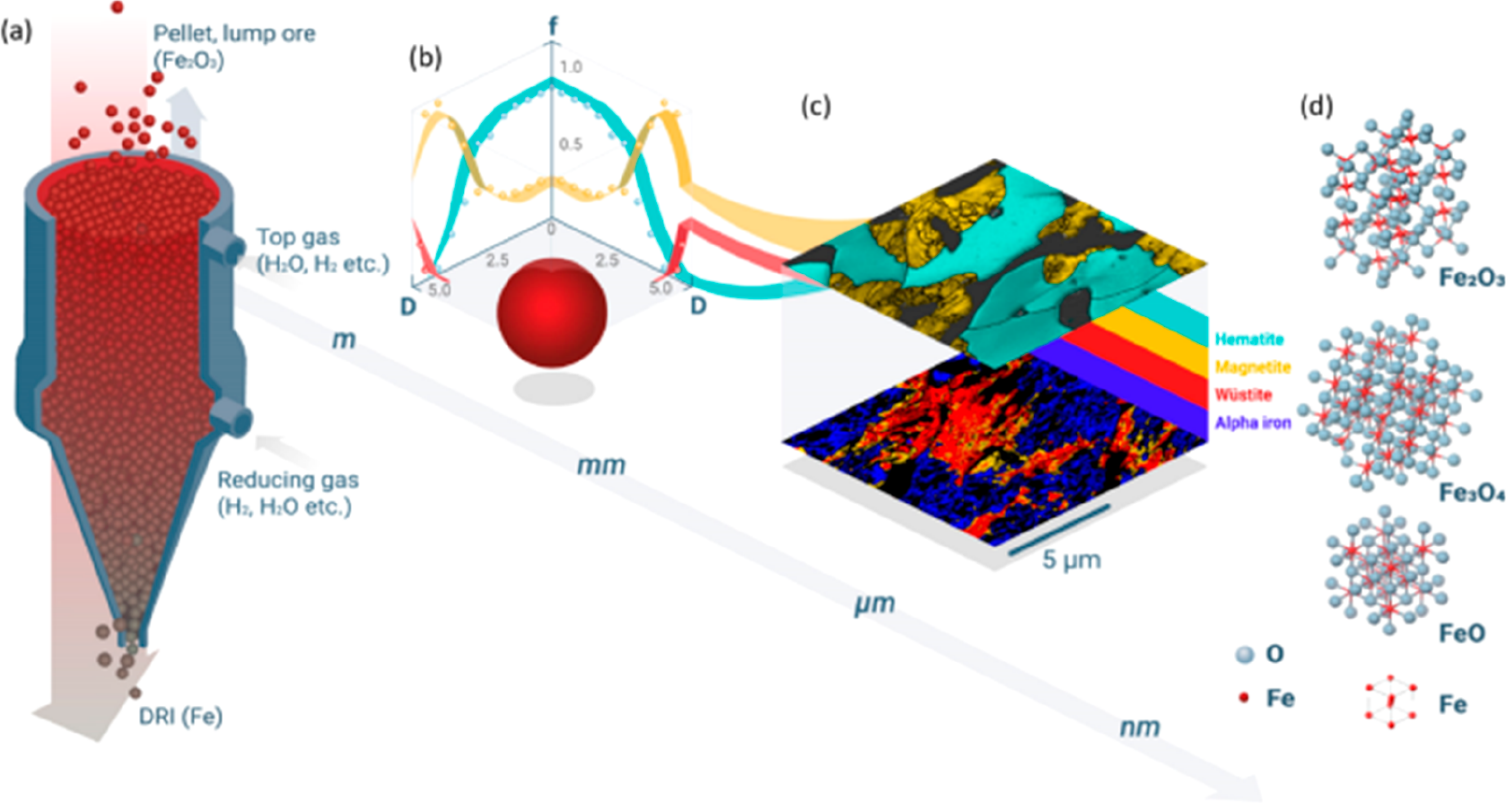
Gradient effects^181^ (across pellet and reactor dimensions) and a few examples of the multiple scales involved regarding reactor concepts in the sustainable metallurgy of steels, here shown exemplarily for the case of hydrogen-based direct reduction in a static bed shaft reactor.^181,182^ Most of these challenges are similar to those observed in other reactor concepts and other processes in sustainable reduction.^143,182,183^ The figure is reproduced with permission from ref (181). Copyright 2022, Springer, Creative Commons Attribution 4.0 International License. DRI, direct reduced iron.

In addition to technology-specific research challenges, there are also fundamental approaches and research tasks that are common to the whole research field of sustainable iron oxide reduction, independent of the specific processing used. This includes the need to develop suitable simulation methods that can describe the complex interaction of microstructure, chemistry, and mechanics, as well as new in situ and in operando measurement methods that can map the corresponding redox reactions with the highest possible accuracy in terms of chemical precision, phase sensitivity, and spatial resolution in order to better understand the underlying mechanisms, Figure 43.

Table 8 lists some topics worth studying with respect to a more sustainable iron production.

### 4.3 Sustainability-Related Research Topics for Aluminum Alloys

**Figure 44:**
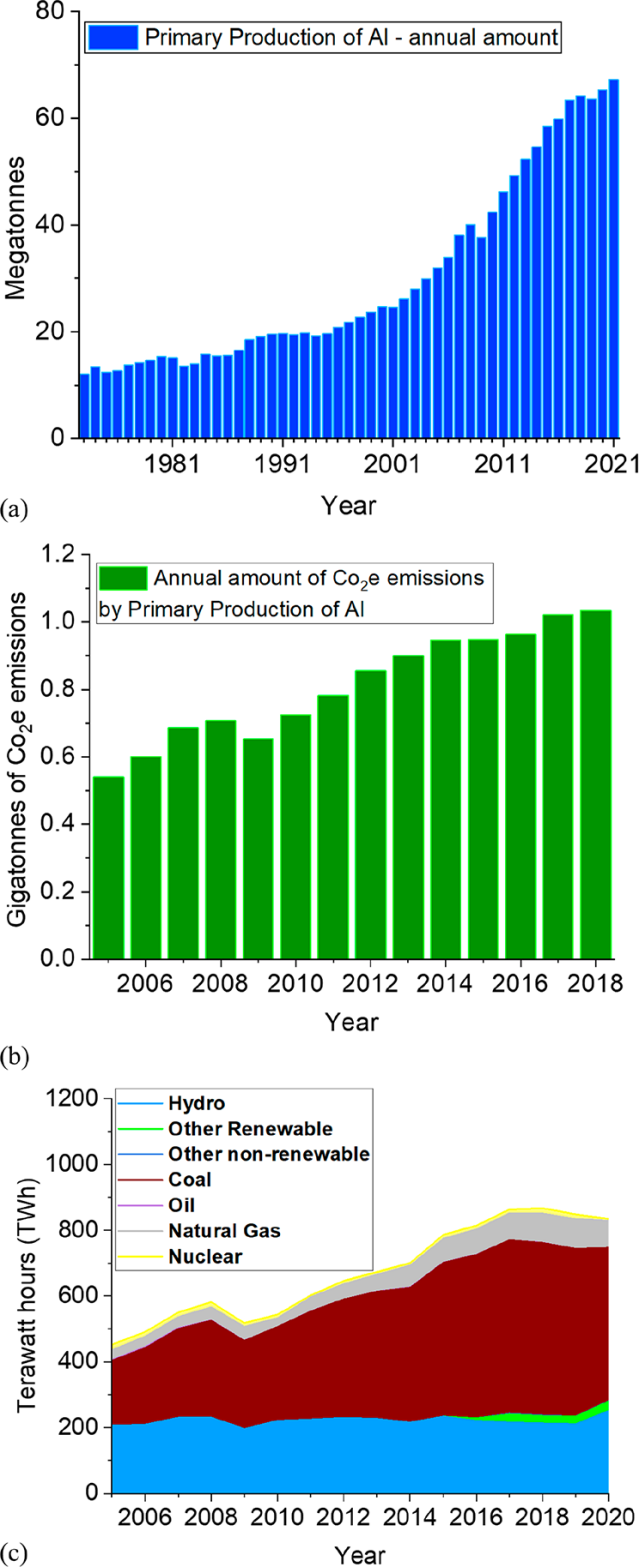
(a) Global amount of primary aluminum production in megatonnes (Mt) for the years 1973–2021.^185^ (b) Calculated global annual amount of CO_2_eq emissions emitted by primary production of aluminum in gigatonnes (Gt) for the years 2005–2018.^185^ (c) Global annual energy consumption for the primary production of aluminum with the origin of the energy sources given in terawatt hours (TWh).^185^ The figures are reproduced with permission from data of the International Aluminum Institute (IAI).^185^ Copyright 2022, International Aluminum Institute.

**Figure 45:**
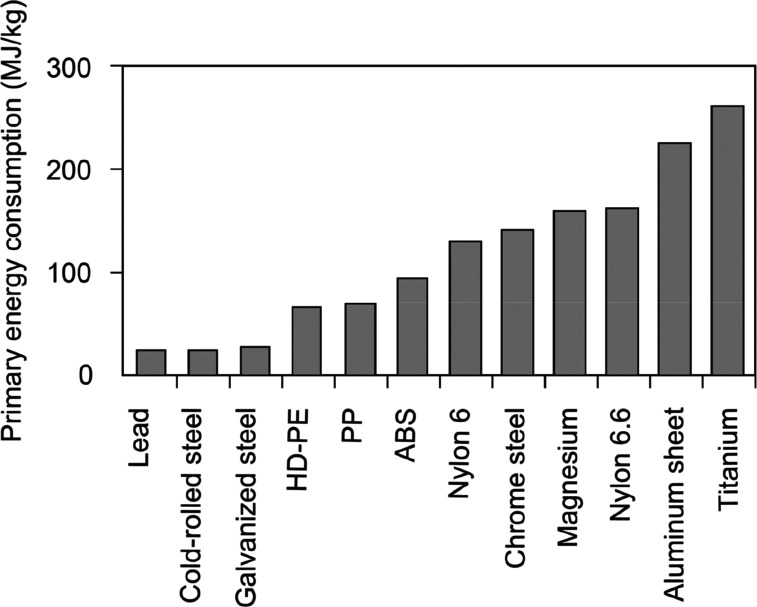
Embodied energy in terms of the primary energy consumption associated with manufacturing for a few selected materials per mass. HD-PE, high-density polyethylene; PP, polypropylene; ABS, acrylonitrile butadiene styrene. Figure is reproduced from ref (186) with permission. Copyright 2008, Elsevier.

Aluminum and its alloys establish the second most important metal group in terms of production volume, greenhouse gas emissions, demand for electricity for primary synthesis via electrolysis (see section 7.7.4), and the volume of harmful by-products such as red mud (see section 6.4.2), Figure 44 and Figure 45.

The metal has one of the highest levels of embodied energy and emissions of all mass-produced metals, with a global average of 11–25 tonnes CO_2_ per tonne of aluminum produced,^184^Table 3 and Figure 44. This value varies substantially between regions, from 25 tonnes CO_2_ in countries with a high fraction of fossil-powered electrical energy to values as small as about 0.5 tonnes CO_2_ in regions which operate the electrolysis plants with hydropower. Most of these emissions are indirect ones, stemming from electricity generation, consumed in electrolysis, Figure 5. On global average, 14.5 MWh of electricity is required per tonne of aluminum in this main primary synthesis stage. Another quarter of the emissions come from the degradation of carbon anodes during electrolysis and from perfluorocarbon emissions from the electrolyte. The rest of the emissions come from the production of alumina, the key raw material for aluminum smelting, Figure 44.

**Figure 46:**
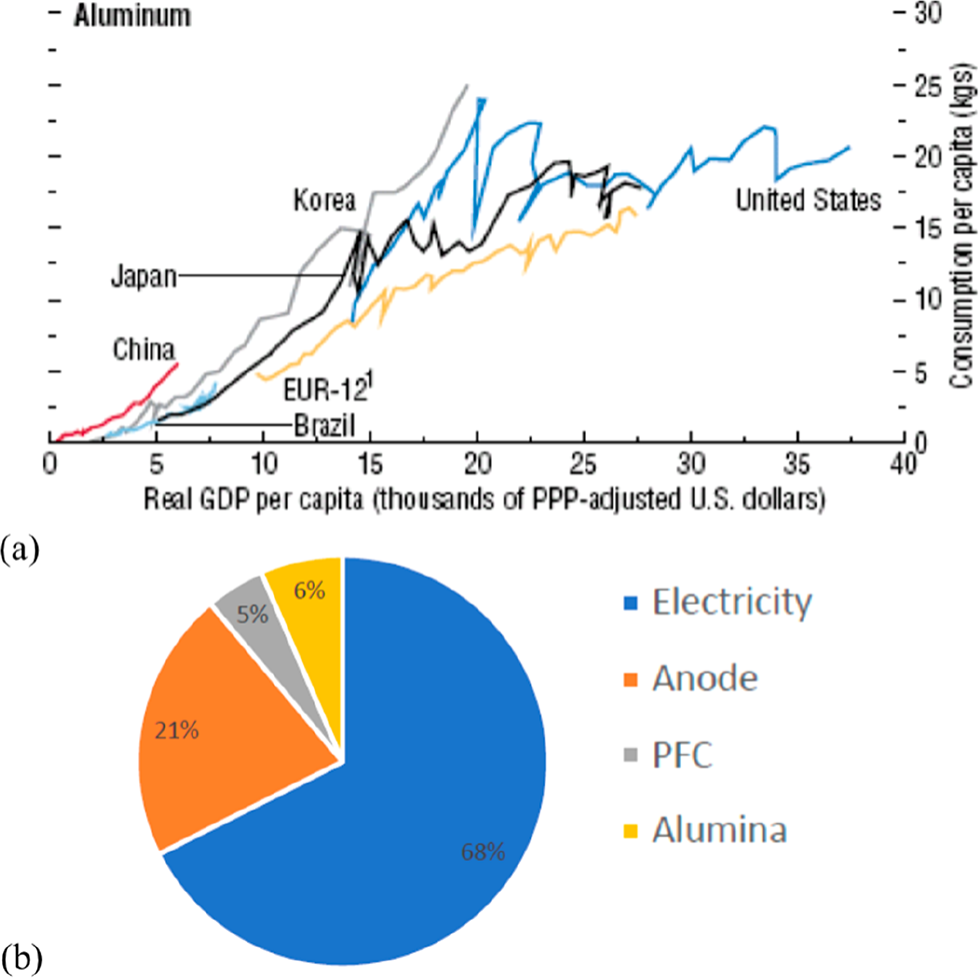
(a) Global per capita consumption of aluminum. Data from IMF World Economic Outlook presented in ref (158). (b) CO_2_ emissions in aluminum production by source, also showing that the CO_2_ emissions associated with the source of electricity used is the main factor. The figure is reproduced with permission from ref (158). Copyright 2010, The Open University, Milton Keynes, U.K. PFC, perfluorocarbon emission. This legend entry refers to efforts that need to be taken to reduce the effects of PFC emissions due to anode effects.

**Figure 47:**
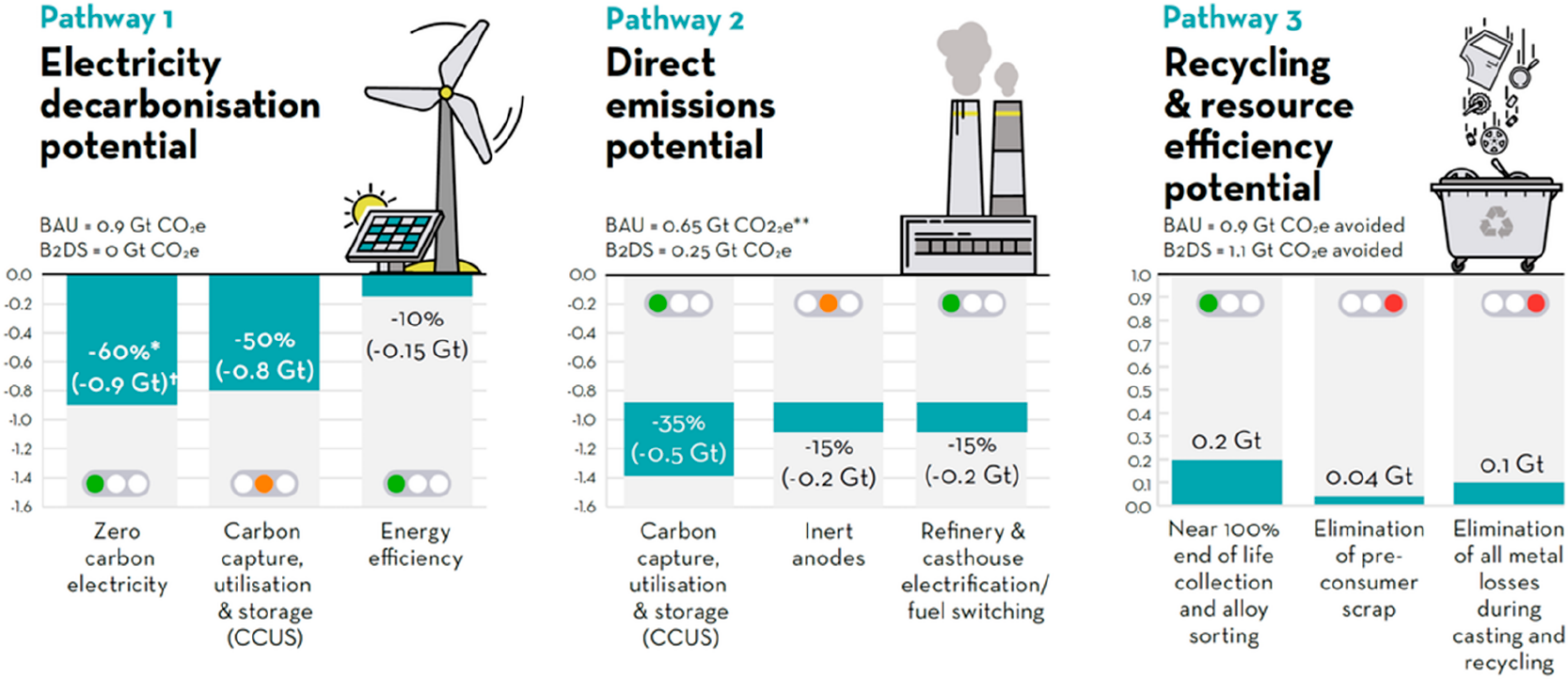
Three different pathways for reducing CO_2_ emissions in the global aluminum production. The analysis reveals that the highest potential for improved sustainability lies in using renewable electricity (for the electrolysis). Data and image are taken from the International Aluminum Institute. Full information about the details of the calculations are in the original report.^187^ The figure is reproduced with permission from ref (187). Copyright 2021, The International Aluminum Institute. Gt, gigatonnes; CO_2_e, carbon dioxide emission equivalent.

The global per capita consumption of aluminum is growing, driven by lightweight design demands in electrical vehicles, a trend which also amplifies opportunities for the use of higher recycled fractions in synthesis in the coming decades, to make these products more sustainable,^188−192^Figure 46 and Figure 47.

The advantage of aluminum in terms of scrap use is that up to 95% energy can be saved by melting scrap instead of making it from oxides via electrolysis, because of the metal’s low melting point. However, there is also an aluminum-specific disadvantage, namely its low solubility for other elements. This means that most of the impurity elements introduced into the material by the scrap become intermetallic phases and these can deteriorate the materials’ mechanical and corrosion properties. This creates a high incentive for basic research in this field, namely, to develop alloys that are more robust against impurities and form less-harmful, i.e. less brittle, and well dispersed intermetallic precipitates.^7,193,194^

Another advantage of aluminum is that its primary synthesis is based on electrolysis. This means that sustainable electrical energy can be used for its primary production. A challenge here lies in replacing the current carbon-based electrode materials by inert materials, to reduce the CO_2_ emissions associated with their gradual burnup. Similar to iron, a more sustainable primary synthesis of aluminum is not limited by a lack of raw materials but rather by the availability of sustainable electricity.^147,195^

Both large metal classes, steels and aluminum alloys, play also a huge role in indirect sustainability, through the weight reduction they enable, for instance in vehicles, an effect which translates linearly to a reduced consumption of gasoline or electrical power, respectively.

A large trend, triggered by downstream automotive, packaging, laptop, and cell phone manufacturers, is to purchase preferentially low-emission aluminum grades, where the material comes, for instance, from hydropower driven electrolysis and from alloys made from high scrap fractions, opening up ample room for novel and disruptive approaches in this field. More details about promising basic research avenues in sustainable aluminum making are presented in the ensuing sections.

**Table 9:** Opportunities for Basic Metallurgical Research for Sustainable Aluminium Production, with Potentially High Leverage on Improved Sustainability

**Process- and mechanism-related research on sustainable aluminum production**
Primary synthesis: Use of inert and carbon-reduced electrode concepts; use of renewable electrical energy along the entire production chain; increase the efficiency of energy usage; reduction of red mud production during bauxite processing; reduction in greenhouse gas emissions and other pollutants associated with alumina production
Secondary synthesis: All measures that increase the amount of recycled aluminum used in production, thus reducing the need for new raw material gained from electrowinning; improved scrap collection and alloy-specific sorting; avoidance/removal of iron (and of other harmful scrap-related impurity elements) from scrap parts; automated and alloy-specific scrap sorting
Tertiary synthesis: Re-mining of aluminum oxide from red mud and use as feedstock in electrowinning. One should note that not only aluminum but also iron, titanium, and a few rare earth as well as some precious metals can be recovered from red mud
Alloy design measures: Scrap-oriented alloy design; science of “dirty alloys”, i.e. systematic study of effects of generally higher impurity element content on the properties of the alloys; development of alloys that can make use of scrap types with less market demand (such as for instance high-Si-containing aluminum scrap); development of cross-over or respectively uni-alloys; rapidly solidified alloys with higher impurity content; alloys that can act as donors for and as acceptors of scrap

Table 9 lists some topics worth studying with respect to a more sustainable aluminum production.

### 4.4 Sustainability Aspects Associated with Copper

**Figure 48:**
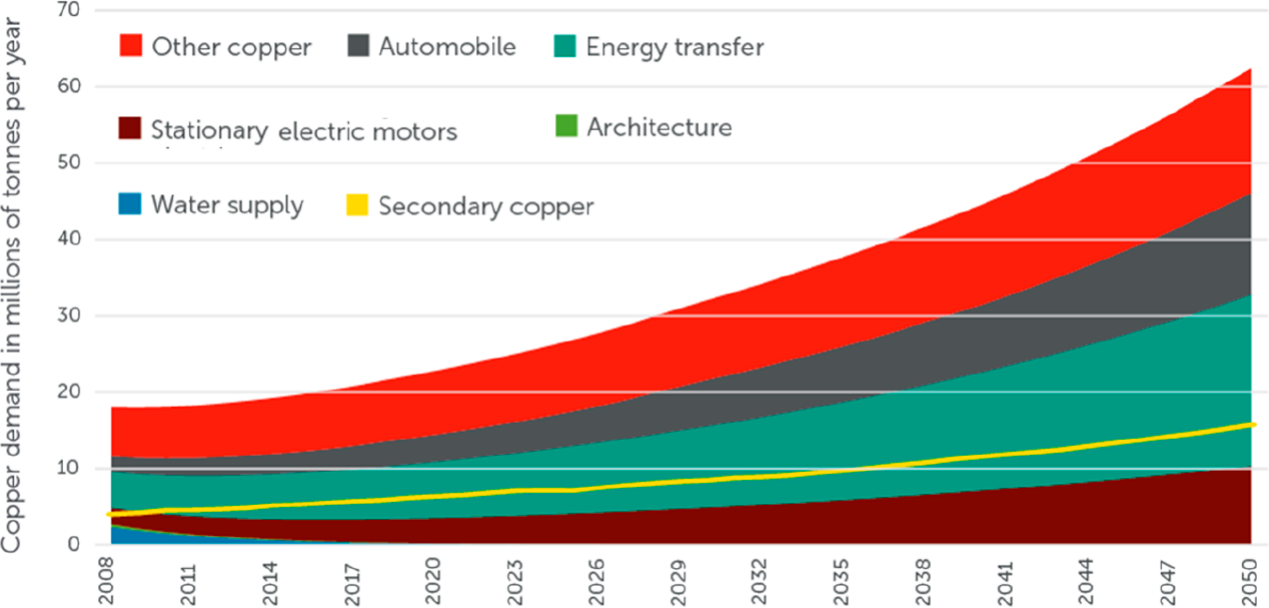
Estimated growth in copper consumption by sectors until 2050. The numbers are taken from the Copper Alliance.^196^ The yellow line shows the fraction of secondary copper. The figure is reproduced with permission from ref (196). Copyright 2021, The Copper Alliance.

While high-strength steels and aluminum are used for lightweight and safe load-bearing parts, copper is the key metal for the electrification of transport, energy supply, and industry.^5,71,111^ It is an essential material for low-resistive loss current transport, electrical motors, solar panels, wind turbines, and electric vehicles. Its high electrical conductivity makes copper the most important element when it comes to the direct use of sustainable electrical energy, Figure 48–Figure 50 (see also sections 6.3.7, 7.4.12, and 7.6.4).

**Figure 49:**
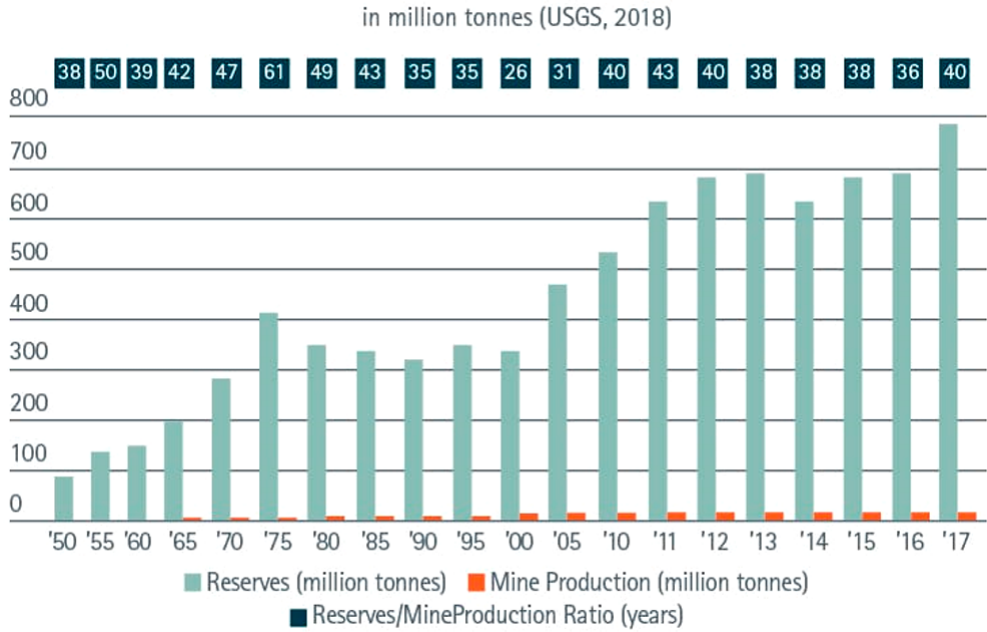
Historical copper production.^196^ The figure is reproduced with permission from ref (196). Copyright 2021, The Copper Alliance.

**Figure 50:**
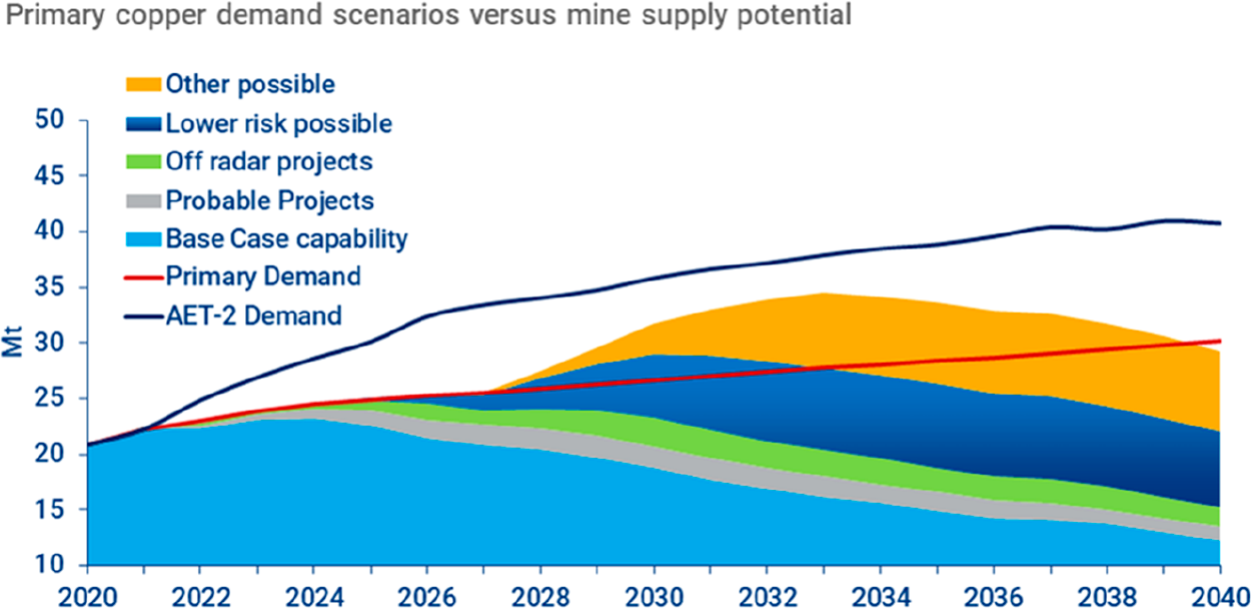
Potential mismatch between growth in copper demand vs available mining resources, plotted for different mine supply scenarios. The term “AET-2 scenario” refers to an accelerated energy transition case, in which particularly high volumes of copper would be needed. This scenario assumes a market demand for copper propelled by electrification and renewable technology investments, to limit the average global temperature increase to 2 °C (according to the Paris Agreement) above 1990 levels. It would increase copper demand growth to 3.5% per year. The base case demand scenario refers to a case where the demand for primary copper is set to grow by an average of around 2% per year over the next 20 years. Numbers and estimates are from ref (197). Mt, million metric tonnes. The figure is reproduced with permission from ref (197). Copyright 2019, Wood Mackenzie.

Unlike iron and aluminum, which are both equipped with rather abundant ore deposits, copper is supply limited; i.e., there are insufficient resources of accessible ores to meet the rapidly growing market demand, Figure 15 and Figure 23–Figure 27. This is particularly important in the context of the rebound effect explained above, which creates a huge additional market pull, driven by the massive growth of the sustainable energy sector and the need to bring this energy to consumers (wind farms, solar parks, electrical vehicles, etc.).

Global demand for refined copper will drastically grow in the next decades due to the electrification of industry, transport, and households. The total demand in 2010 of about 16 Mt of copper is expected to grow to 23 Mt in 2030, at greenhouse gas emissions of approximately 87 kt CO_2_ equivalent emissions, depending on the technologies used. The specific numbers will substantially depend on progress in decarbonizing the electricity generation that is used in copper mining, extraction, and downstream processing.^71,198,199^

Pyrometallurgical processing accounts for over 80% of the world’s primary copper.^200^ According to current mineral reserve projections, if no new mines are opened, the annual copper supply shortage could be as high as 10 million tonnes by 2030, Figure 50. Other potential mining sites, however, provide either only very deep resource locations, of lower grade, or they are in protected environments, according to the current status of exploration. Another concern is that some new mining spots for copper carry a high content in arsenic, one of the most toxic elements to have as byproduct. For these reasons, the sustainable recycling of copper is an extremely important topic. Yet, this is challenging because copper is often hidden in scrapped parts in a highly dispersed way, such as in joints, electrical devices, and electronic products.^69,111,201^ Particularly the intense material integration in the latter group of products is problematic, as it creates a mixed type of scrap material that can be termed nanoscrap, with often consists of up to 30–40 chemical elements mixed at tiny dimensions.^89,90,202^

Recycled copper covers about a third of the global copper demand. The current global end-of-life recycling rate for copper is between 30 and 40%. In some regions more than half of all copper is recycled after use. As all the energy for mining, crushing, and benefication is obsolete in recycling, the total energy requirements for secondary copper production from scrap are between 30–80% below those needed for primary production. This translates to a global average in greenhouse gas emission per tonne of copper produced from secondary resources to about 0.2–1.9 t CO_2_. The recycling process and its efficiency depend on the types of scrap used, the associated impurity elements, and its copper content.

**Figure 51:**
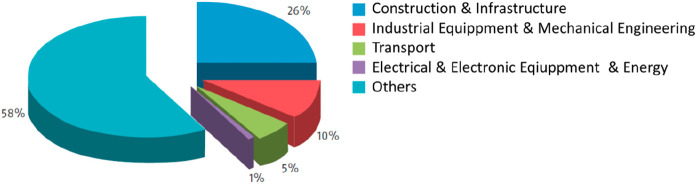
Use of copper in different branches.^196^ The figure is reproduced with permission from ref (196). Copyright 2021, The Copper Alliance.

Copper has very high longevity: materials used in architecture and to wire homes and buildings are often more than 50 years and more in use, so that they do not feed the scrap market in short cycles. Therefore special attention must be placed on retrieving the copper from short-lived products such as consumer electronics, Figure 51. Detailed research opportunities for sustainable copper production will be addressed in dedicated sections below, Table 10.

**Table 10:** Opportunities for Basic Metallurgical Research Related to Sustainable Copper Production, with Potentially High Leverage on Improved Sustainability

**Process- and mechanism-related research on sustainable copper production**
Primary synthesis: Use of efficient combined reducing plasma and smelting methods, where contaminated scrap can be processed; synthesis methods with reduced energy consumption and increase in the efficiency of energy usage in copper production; use of less poisonous mineral resources in copper production; use of less water in copper production; responsible mine tailings management including measures to safely manage and dispose of waste materials generated during the mining process to reduce environmental impact, particularly of arsenide contaminated waste; new methods for the processing and extraction of copper from (oxidic and sulfidic) low-grade ores; reducing the need for high-impact mining of rich copper deposits; responsible water management such as reduced water usage and minimized impact of waste water from copper production on local ecosystems
Secondary synthesis and alloy design measures: All methods to increase the amount of recycled copper used in production; better scrap collection and sorting; alloy-specific scrap sorting; sustainable recycling of copper from electronic waste (nanoscrap recycling).

### 4.5 Sustainability Aspects Associated with Nickel and Cobalt

**Figure 52:**
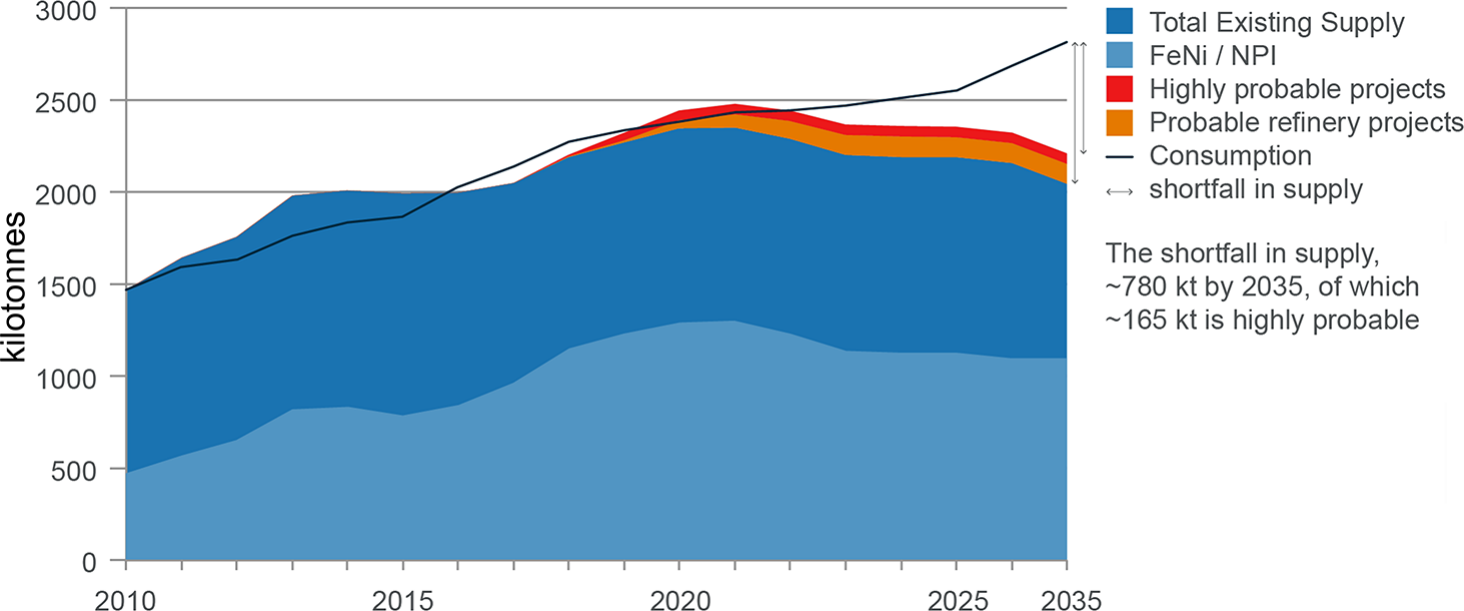
Nickel market in kilotonnes and expected gap between demand and availability by the year 2035.^205^ NPI, nickel pig iron, a low-price and low-grade ferronickel pre-alloy as low-cost alternative to pure nickel. This material can be used for example for the production of FeCrNi stainless steels. The figure is reproduced with permission from ref (205). Copyright 2022, The Multidisciplinary Digital Publishing Institute.

Like most metals, nickel and cobalt have two sides with regard to sustainability,^87,203,204^Figure 52. When used in alloys, they enable many products that are needed for a sustainable economy and that have high longevity (see also sections 6.2.9, 6.2.10, and 6.3.10). Examples are electrical vehicles, where they serve in the cathodes of lithium ion batteries; hard magnetic materials used in the sustainable electrification of industry and transportation; special and stainless steels which make products corrosion resistant and lend the material high strength and formability; and superalloys in which they enable microstructures and phases that equip these alloys with high creep resistance, as key components for parts in thermal engines with high Carnot efficiency. These applications make nickel and cobalt indispensable elements for green technologies and electrification. Their other side is that they have one of the highest CO_2_ emissions per metal unit produced of all the elements, Figure 29.

**Figure 53:**
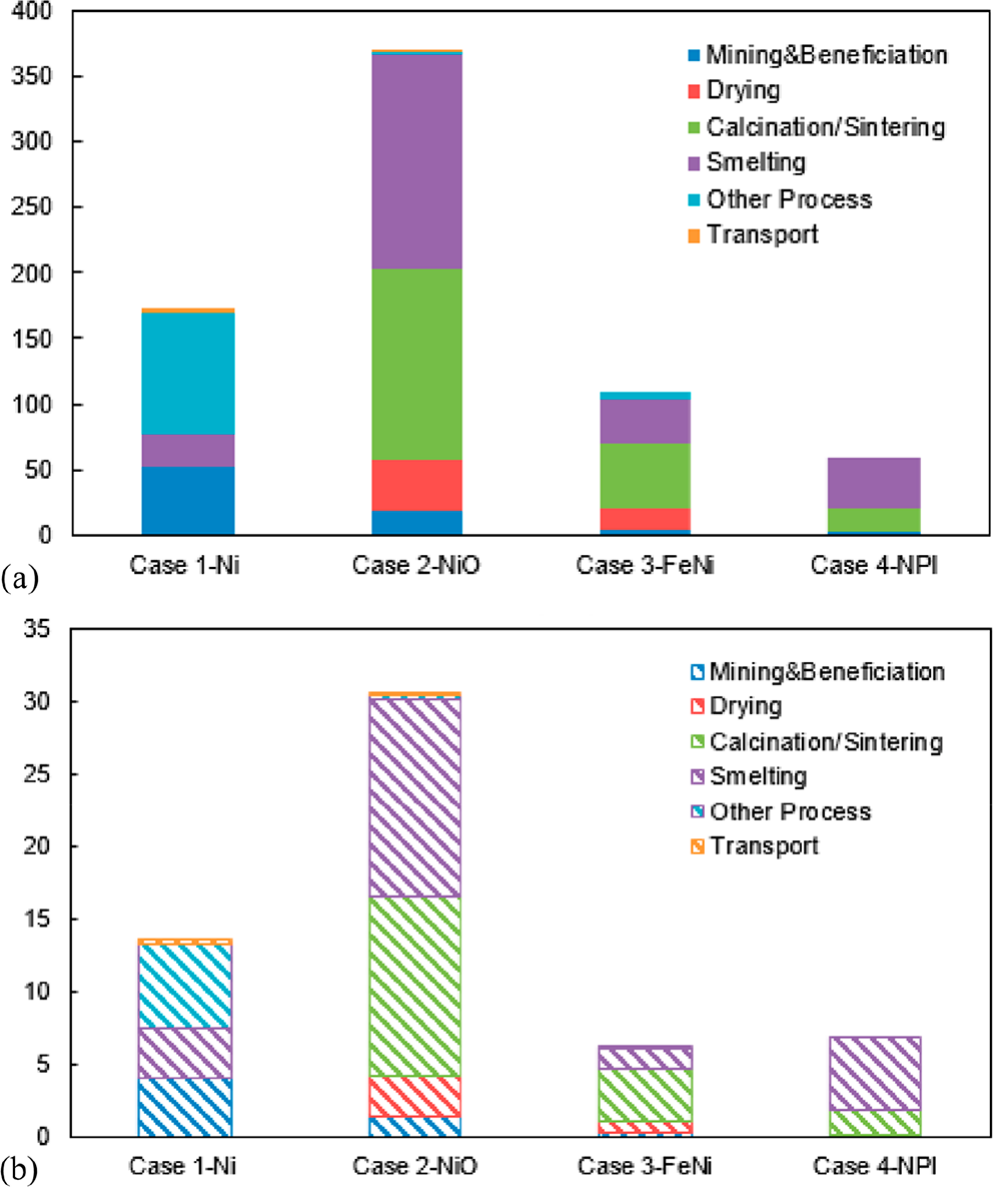
(a) Energy consumption in gigajoule (GJ) per tonne of metal produced. (b) Greenhouse gas emissions in tonnes CO_2_eq per tonne of metal produced together with the individual process steps.^206^ NPI, nickel pig iron. The figure is reproduced with permission from ref (206) under an open access Creative Commons CC BY license. Copyright 2020, MDPI.

**Figure 54:**
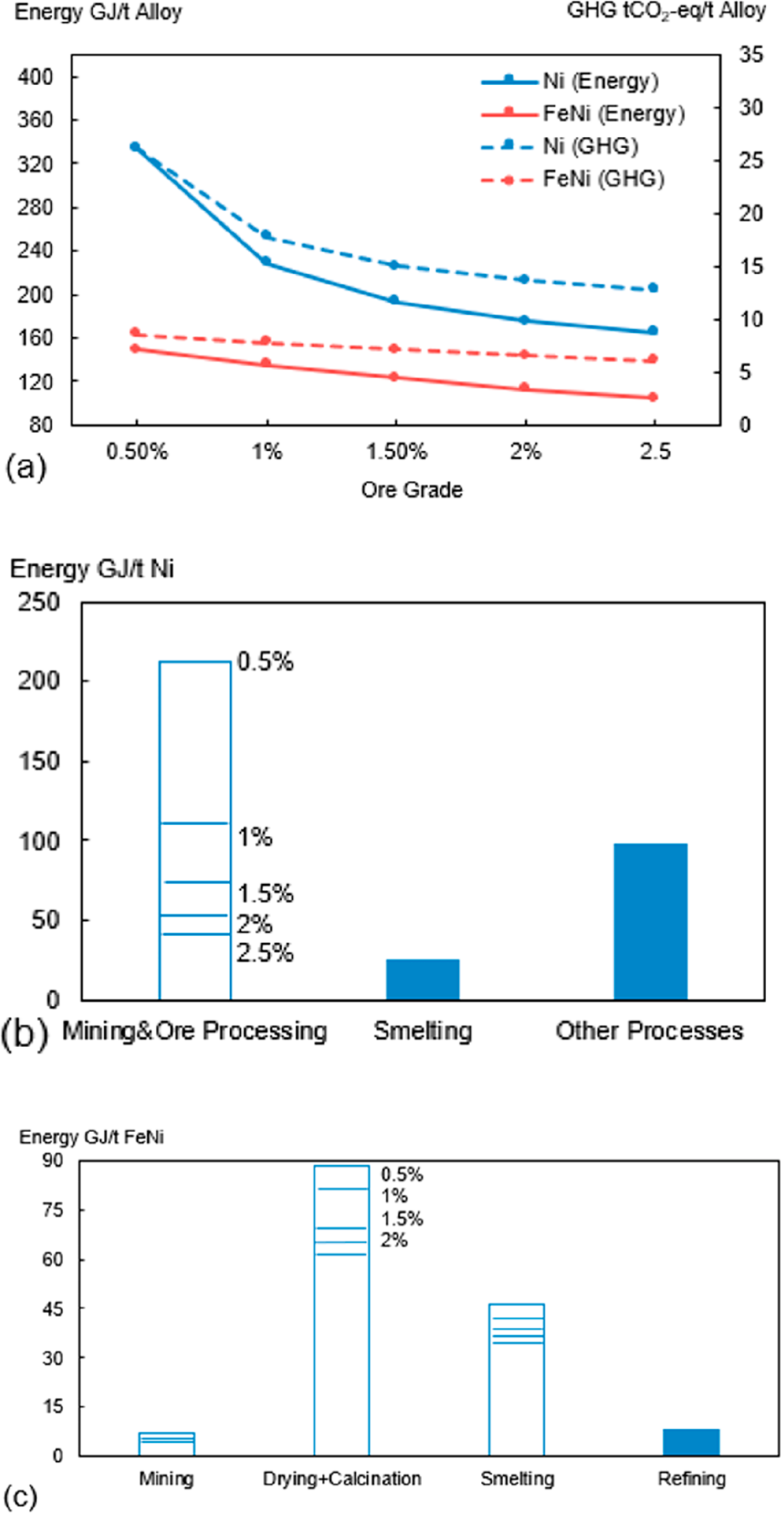
Energy consumption and CO_2_ costs associated with nickel production with respect to ore grade.^206^ (a) Effect of ore grade on total energy consumption and greenhouse emissions of nickel (Ni) (blue) and ferronickel (FeNi) (red). (b, c) Effect of ore grade on energy consumption of subprocess for production of (b) nickel metal and (c) ferronickel (FeNi). The figure is reproduced with permission from ref (206) under an open access Creative Common CC BY license. Copyright 2020, MDPI.

Important nickel- and cobalt-containing minerals used for synthesis are sulfides, arsenides, oxides and hydroxides. Depending on the mineral source and reduction process used, the processing and the CO_2_ emissions per tonne of nickel and cobalt metal produced vary substantially, Figure 53. The global average is around 20 tonnes CO_2_ per tonne of metal. About 60% of these CO_2_ emissions are usually scope 1 emissions, Table 1; i.e. they are direct emissions, produced onsite, in the processing plant. About 15% are in global average due to indirect emissions, related to the consumption of electricity used; i.e. they are scope 2 emissions. Yet, with these values for the respective CO_2_ emissions in connection with nickel and cobalt production, it should be noted in particular that the specific CO_2_ emissions for these metals are dependent on the specific metal content of the ore used and the processes employed for enrichment and extraction. This results in a huge range from only 3–7 tonnes to 35 tonnes of CO_2_ emissions per tonne of nickel and cobalt metal produced, Figure 54.

In its final delivery state to downstream industries, two types of nickel are differentiated in the market, namely, class 1 nickel and class 2 nickel. Class 1 nickel meets a purity standard of 99.8%, or better. Class 2 comprises nickel-containing products with a purity below 99%, Figure 54. This material is often used in stainless steel and alloy applications with a robust refining step in the process so they can take materials that are less pure or they can make use of the iron content that usually comes along with ferronickel. Although the classes do not necessarily make a statement where the material comes from, class 1 nickel production originates to about 70% from sulfide ores, which are concentrated, smelted, and refined, and approximately 30% from limonite ores, through the high-pressure and acid leached process explained above. Class 2 nickel is mostly produced from saprolites and limonites, which are popular for their use in the stainless steel industry due to their high iron content and moderate production costs (see further details in sections 6.2.9, 6.2.10, and 7.4.10).

In the production of stainless steels, in addition to class 1 nickel, mainly ferronickel (20–40% nickel content) and increasingly also nickel pig iron (5–15% nickel content) are used as alloy ingredients, Figure 52. Class 1 nickel is nowadays mainly used for the production of nickel alloys and in casting industries. Nickel chemicals, especially nickel sulfate, are used for electroplating and increasingly for battery production.

More than 3/4^rd^ of all nickel mined today is used in stainless steels. Batteries currently stand for less than 8% of the nickel market, yet, with a rapidly growing trend if no alternative and stable battery cathode materials are identified. In 2030, more than 25% of the global nickel demand, which is estimated at around 4 million tonnes of nickel annually at that time, is already expected to be used for battery production alone.^35,207^ This corresponds to almost half of primary nickel production in 2019.

For the emerging lithium-ion battery industry, the quality of the nickel is of the highest relevance for the performance of the cathodes in which it serves as an alloying element, influencing the quality and performance of the entire battery.

Furthermore, following similar concerns about the origin of another battery raw material, cobalt, electric vehicle manufacturers and their clients are seeking to ensure that the raw materials used in their products are mined and refined in an environmentally responsible manner, with positive impacts on local communities, and with a limited carbon footprint.

However, nickel is one of the most technically challenging and energy as well as greenhouse gas intensive metals to process and refine, with details depending on the type of ore deposit and reduction process, Table 3. This has a substantial impact on the use of nickel (and cobalt) on sustainable technologies. As an example, large electric vehicle batteries currently on the market store about 100 kWh. With a consumption of 15 to 25 kWh per 100 km, sedan- and SUV-sized electric vehicles can run between 400 and 600 km on one battery charge. About 66 kilos of nickel are needed for such a 100 kWh battery.

Nearly 70% of nickel from post-consumer scrap is recycled, and about 15% enters the carbon steel loop. Yet, still about 17% is lost to landfills, mainly in metal goods and in waste electrical and electronic equipment.

Due to the high CO_2_ emissions, one interesting research aspect is to find alternative materials that are capable of replacing nickel, for instance, in the stainless steel sector or in battery electrodes. Also the growing market for alloys with Invar properties (low thermal expansion coefficient) is an attractive research field, to find alloys containing less costly and more sustainable elements.^208−210^

Another big disadvantage of nickel lies in its highly speculative market position—the metal is traded, and only a tiny fraction of it is finally used in products. This means that the supply and price of nickel are some of the biggest factors of uncertainty for customers. Most of the actual market value of nickel is just due to speculation and does not reflect the real industrial need. This is a typical market mechanism; however, it makes it very challenging for producers to cope with the predictability of the price of such a material, which is again another incentive for trying to find solutions that can be realized without the use of this element.

The recycling of nickel and cobalt from diverse sources makes up the secondary supply side. Stainless steels, catalysts, cobalt-containing scrap, superalloys, hard metals, magnets, and batteries have been the most important sources up till now. Battery recycling, in particular, will contribute significantly to supply in the future. The product’s service life, and hence a time delay before recycling, as well as losses in the recycling process, must be considered. Some emerging basic research topics related to more sustainable nickel and cobalt are shown in Table 11.

**Table 11:** Opportunities for Basic Metallurgical Research Related to Sustainable Nickel and Cobalt Production, with Potentially High Leverage on Improved Sustainability

**Process- and mechanism-related research on sustainable nickel and cobalt production**
Primary synthesis: All synthesis methods with reduced energy consumption and increase in the efficiency of energy usage in production; better water management and less water usage in production: because cobalt and nickel sulfide minerals require for extraction and benefication about 1,100–1,500 liter of water per tonne of ore and laterite ores need even up to 4,500 liter of water per tonne of ore, less water-intense benefication is of highest importance; use of efficient combined reducing plasma and smelting methods, where contaminated scrap can be processed; low-energy synthesis (sulfidic ores require up to 2.5 GJ energy per tonne of ore and lateritic ores require up to 8 GJ energy per tonne of ore); electrification of process steps; responsible mine tailings management including measures to safely manage and dispose of waste materials generated during the mining process to reduce environmental impact, particularly of arsenide contaminated waste; new methods for metal extraction of low-grade ores; minimized impact of waste water from metal production on local ecosystems
Secondary synthesis and alloy design measures: All methods to increase the amount of recycled nickel and cobalt used in production; better scrap collection and sorting; alloy-specific scrap sorting; science of “dirty alloys” (study of effect of generally higher impurity element content); alternative and compositionally more lean electrode materials; stainless steels with lower nickel content

### 4.6 Sustainability Aspects Associated with Titanium and Its Alloys

Titanium it one of the most important structural materials for advanced lightweight applications, due to its low mass density of 4.5 g cm^–3^ and high strength, coupled with excellent corrosion and high-temperature creep resistance. Titanium is usually characterized in terms of specific grades according to the ASTM standard. Grades 1 to 4 denote pure titanium of various degrees of purity, mainly referring to its oxygen content.

**Figure 55:**
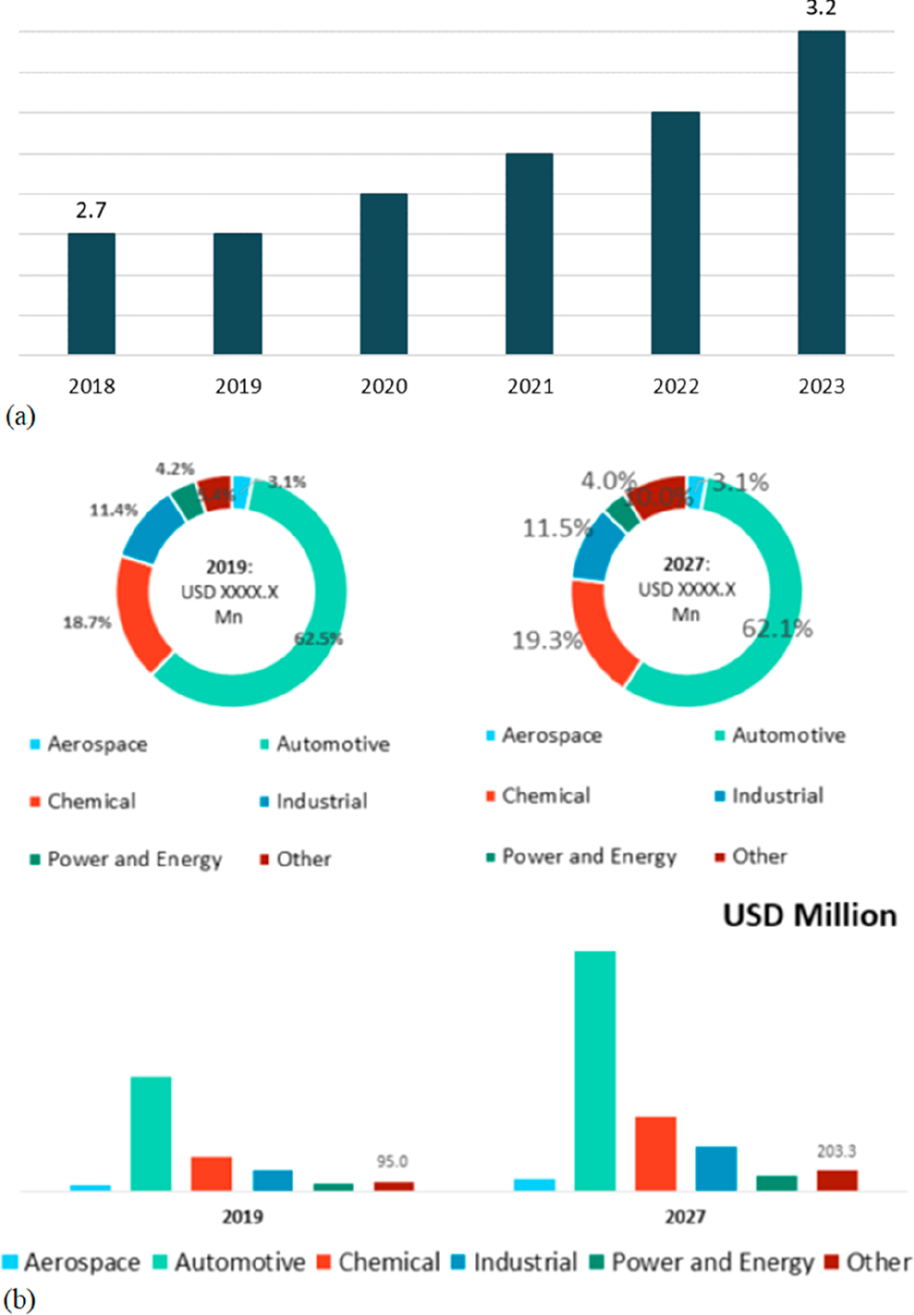
(a) Global titanium alloy market in billion US dollars and (b) in terms of markets and downstream products.^220^ The figure is reproduced with permission from ref (220). Copyright 2020, DataIntelo.

The most important alloy employed commercially is the alloy Ti-6Al-4V (with 6 wt % aluminum and 4 wt % vanadium, with a duplex microstructure containing bcc (beta phase) and hcp (alpha) phases, used in aero-engine parts and airframes, Figure 55. Titanium also serves in orthopedic implants, power and desalination applications, automotive and ship building, chemical industries, and the aerospace sector.^211−214^

The expected annual growth rate of the titanium market is about 4.5% over the next decade, driven by weight reduction requirements in vehicles and planes and the global growth of the defense and space sectors.^215^ Owing to its very attractive properties, titanium could be a structural material of high relevance in future electrical vehicle concepts, provided that cheaper and more sustainable synthesis methods could be identified.^216^ As an example, the use of just 1.3 kg of titanium in every new vehicle would lead to a doubling of today’s global demand, Figure 55.

The high costs of producing titanium parts distribute over several process steps, including sponge production, refining, and semifinished part production. Titanium is one of the most expensive mass-produced metals due to the complexity and energy-intensive nature of the Kroll process, which is used to produce primary titanium, and the required feedstock minerals (see details in sections 6.2.8, 6.3.8, 6.4.4, and 7.4.11).^216^

Unlike for some other transition metals, most of the titanium scrap is not coming from end consumers but it is produced and collected during industry smelting and fabrication. Thus the collection and recycling rate of titanium is rather high, approximately 50% at a global scale. The main impurity elements are oxygen (from corrosion and oxidation during machining) and iron (abrasion from machining tools).^150,217^

The fact that titanium is primarily used in the aerospace sector is a critical factor in terms of sustainability.^218^ Here, forming processes are usually avoided and instead machining is used to reduce internal stresses. However, machining produces titanium chips, and up to 90% of the material is lost and turned into scrap in this manufacturing step.^150,219^ The chip material is usually highly contaminated and oxidized by the lubricants and tools used for machining.

Some of this oxygen- and iron-contaminated scrap is used for the production of ferrotitanium, serving as precursor ingredient in the steel industry, for the production of microalloyed steel grades. However, the more highly contaminated scrap material cannot be recycled and must instead be downcycled to titanium dioxide.

This means that rewarding research tasks in this sector are the removal of oxygen and iron from contaminated scrap and the development of machining methods that lead to lower contamination in the first place.^150^ Other efforts along these lines are the direct recycling of titanium scraps in titanium production via molten salt electrolysis.^219^ These aspects make the question of secondary synthesis from scrap a very important research topic, which will be discussed in more detail below (see sections 6.3.8, 6.4.4, and 7.4.11). Table 12 lists some topics worth studying with respect to a more sustainable titanium production.

**Table 12:** Opportunities for Basic Metallurgical Research on Sustainable Titanium Production, with Potentially High Leverage on Improved Sustainability

**Process- and mechanism-related research on sustainable titanium production**
Primary synthesis: Reduced energy consumption and increase in the efficiency of energy usage in production; more energy efficient primary synthesis methods; electrification of production; more efficient process variants (e.g., Kroll, Hunter, Armstrong processes)
Secondary synthesis: Clean titanium scrap that can be used for recycling; less chipping in part design; less contaminating machining methods; better scrap collection and sorting; alloy-specific scrap sorting; use of titanium chips for powder production for additive manufacturing; removal of oxygen and iron from scrap melts; molten salt electrolysis for recycling of titanium scrap; vacuum arc melting of titanium scrap; cold hearth melting of titanium scrap; induction skull melting of titanium scrap; hydrogen plasma arc melting of titanium scrap; developing low-grade titanium for less demanding parts, with higher impurity tolerance

### 4.7 Sustainable Metallurgy of Lithium

Lithium is the lightest of all metals. It is abundant in the Earth’s crust, but with very high dispersion.^221,222^ It is nowadays an essential metal, due to its use in batteries.^40,223^ Particularly, large vehicle batteries drive the market: the global volume of the lithium market was about 400 thousand tonnes in 2021, and it is expected to grow by about 20% every year during the next decade (see also sections 6.2.11, 6.3.11, and 7.6.3).

**Figure 56:**
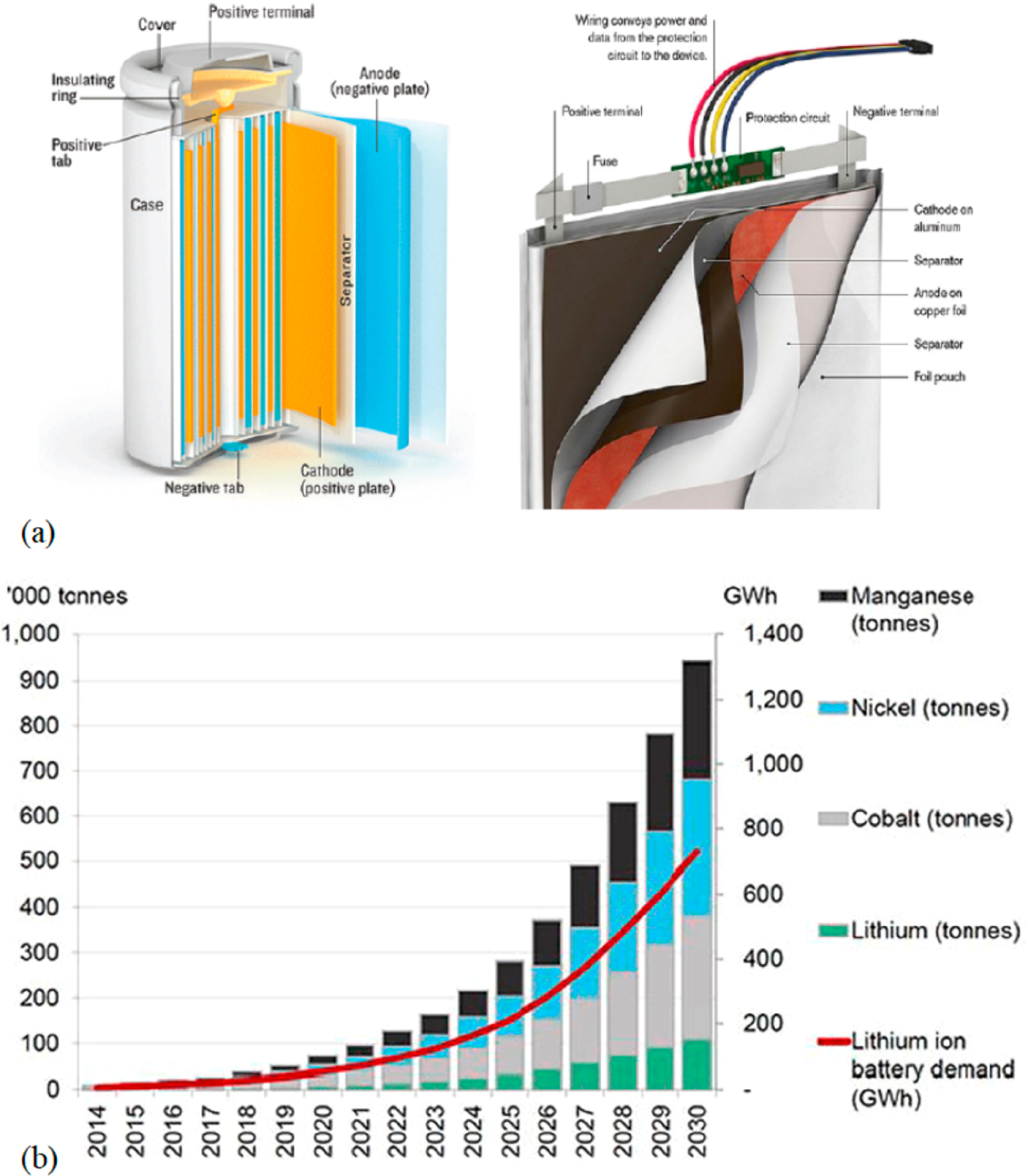
(a) Li battery structure and material composition. (b) Metal market growth due to battery market demands for electrical vehicles. Numbers and images are taken from refs (232 and 233). The figures are reproduced with permission from refs (232 and 233). Copyright 2018 and 2020, ISSRD International Conference.

Commercial lithium-ion battery cell packs currently serving in electrical vehicles have a complex internal structure and a likewise complex chemical composition and material mix. In average the batteries contain 20–25 wt % steel or aluminum for the casing; 25–35 wt % cathode materials such as LCO (lithium-cobalt-oxide), NMC (nickel-cobalt-manganese), NCA (lithium-nickel-cobalt-aluminum-oxide), LFP (lithium-iron-phosphate) or LMO (lithium-manganese-oxide); 14–19 wt % graphite as anode material; 10–15 wt % LiPF6 electrolyte, which is usually dissolved in organic compounds; 5–7 wt % aluminum cathode current collector foil; and 5–9 wt % copper anode current collector foil, and the rest is separator material and a few additives, Figure 56. This translates for an average 1000 kg–120 kWh vehicle battery to 15 kg lithium, 80 kg nickel, 25 kg manganese, 27 kg cobalt, and up to 250 kg graphite.^40,223−226^

Electric cars are very expensive, and the battery cell pack accounts for about 40% of the cost of the vehicle, with about 80% of that coming from the metals lithium, nickel, and cobalt. The prices of these ingredients have doubled within a single year. This means that it is essential to solve the two most important problems in this context through sustainable metallurgy, namely, (1) to reduce costs by enhancing availability of these metals and (2) to do that by developing efficient and sustainable synthesis and recycling methods for these elements.^35,40,227−230^ Otherwise the rebound effect will lead to extremely high costs which will make such cars and the associated beneficial reduction in transport-related CO_2_ reductions unaffordable. A treatise of options for research in recovery of lithium from batteries will be given below.

Primary lithium can be mined as a traditional ore (e.g., in Australia) or from brines in salt deserts, so-called salars (e.g., in Chile and Argentina).^231^ In the latter case the lithium-carrying salt water brines from underground lakes are pumped to the surface for the water to evaporate or for later partial reinjection. The remaining salt solution is processed until the lithium is suitable for use in batteries. A serious sustainability concern associated with lithium brine extraction is that it requires large amounts of water: it is used to dissolve lithium from the brines and to maintain a consistent production process. The brine is pumped from the ground and into evaporation ponds where the water is evaporated to concentrate the lithium and other minerals. This process can take several months to a year, during which time the water is constantly being replenished. After the lithium has been concentrated, the remaining brine is typically disposed of by injecting it back into the ground or evaporating it, both of which again require or respectively waste huge quantities of water. Due to the increasing demand for lithium and the increasing competition for water resources in dry regions, the water intensity of lithium production has become a major sustainability concern, Figure 56.

Another promising approach for lithium mining is to combine geothermal energy production with lithium extraction to create a new composite technology. In a first step, the lithium ions are filtered out of the thermal water pumped to the surface for energy production and further concentrated in a second step until lithium can be precipitated as salt. The existing infrastructure of geothermal plants can already be used for this technology. The additional land required is small, and unlike in classic mining, hardly any overburden is produced. Details will be discussed in more detail below (see sections 6.2.11, 6.3.11, and 7.6.3).

Table 13 lists some promising research topics for a more sustainable lithium production.

**Table 13:** Opportunities for Basic Metallurgical Research Related to Sustainable Lithium Production, with Potentially High Leverage on Improved Sustainability

**Process- and mechanism-related research on sustainable lithium production**
Primary synthesis: Lithium winning methods with less water consumption compared to brine evaporation methods; brine evaporation production with less land use; more efficient precipitation of lithium carbonate; exploration of alternative lithium sources such as brine lakes and geothermal wells; development of more efficient and less wasteful extraction methods; better waste management and water usage practices in lithium mining operations
Secondary synthesis: Advanced sorting and separation techniques to separate different battery chemistries and metals; new efficient and less harmful hydrometallurgical processes for lithium extraction; pyrolysis methods for lithium extraction; improved recycling efficiency; recycling of lithium ion from mixed battery scrap to reduce reliance on mining; replacement of lithium by sodium as charge carrier in batteries; pyrometallurgical processes for recovering metals from batteries

### 4.8 Platinum Group, Gold and Rare Earth Metals

Commercially relevant metals of the platinum group are for example ruthenium, rhodium, palladium, osmium, iridium, and platinum. They are important in a variety of sustainable technologies, particularly for fuel cells, hydrogen production via electrolysis of water, vehicle emissions control, information technology, and consumer electronics. They are thus also important for hydrogen-based electromobility, long-term chemical energy storage, and power-to-gas technologies.^96^ In most of these technologies they serve as catalysts. Similar application fields apply for gold. Jewelry applications are not considered here, as they follow specific market and recycling metrics (see also further aspects tackled in sections 6.3.11, 6.4.5, and 7.4.9).

**Figure 57:**
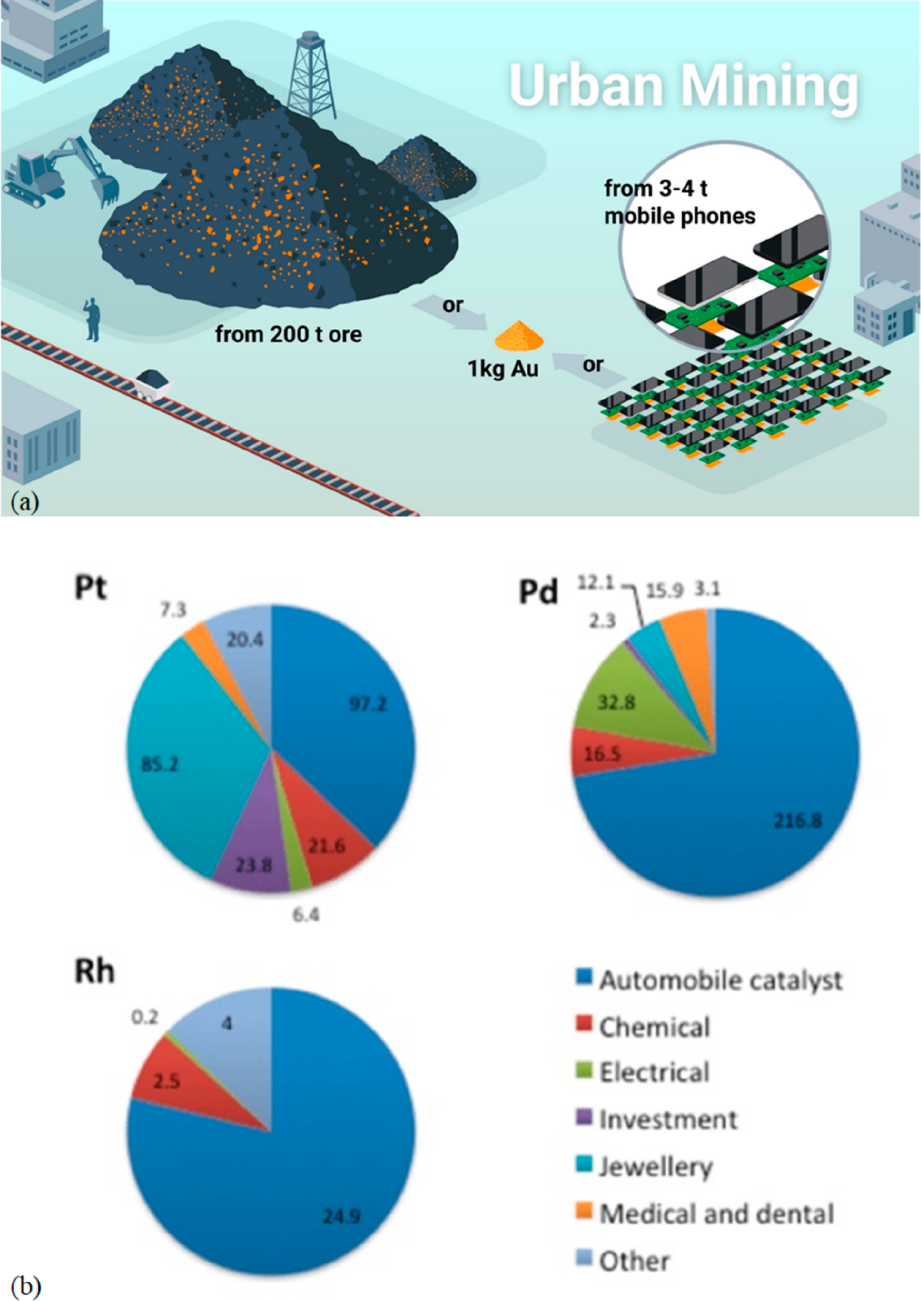
(a) Urban mining versus mineral mining for the case of gold.^133,234^ (b) Platinum group metals used in automotive catalytic converters.^235^ Figure (b) is reproduced with permission from ref (235). Copyright 2020, Waste Advantage.

A few quantitative examples make the importance of these precious metals more clear: computer chips and motherboards contain per one tonne around 200–250 g gold and around 80 g palladium; cell phones contain per tonne up to 350 g gold and 130 g palladium; and automotive catalytic converters may contain per tonne even up to 2000 g platinum group metals in the ceramic catalyst brick, which is the active part of the converter. These values are significantly higher than the platinum group metal or gold content in any of the primary ores. These have on average a content below 10 g per tonne of mineral, Figure 57.^96^

Platinum group metals have a high technical recyclability, which indicates that once scrap containing these elements reaches a suitable refining facility equipped for these metals, more than 95% of the metal can be recovered. This means that it is less the technological challenges but the collection of scrap that matters.

The high metal values make recycling economically attractive, and due to the much higher concentration compared to mining of ores, they also help to reduce the environmental burden, thus qualifying these metals prime targets for urban mining. Even more valuable for recycling are platinum group metals and gold used in jewelry, as these are typically concentrated at an even higher level.

The specific challenge for gold and platinum group metals is that they are often used in nanoscale dimensions and in concert with a large number of other elements (a state which can be referred to as nanoscrap), which makes recycling via hydro- and electrometallurgical methods challenging. Another problem is that catalyst materials in particular are often gradually degraded in the course of their use in the field of catalysis and are thus often lost through molecular reactive or abrasive mechanisms, so that a high level of entropy often stands in the way of recycling in this area.

The rare earth elements include yttrium, neodymium, dysprosium, praseodymium, terbium, europium, cerium and lanthanum. They are needed (mostly as ingredients in hard magnetic materials), for example, for batteries, photovoltaic systems, wind turbines, motors and generators.^12,236^Table 14 lists some promising research topics for a more sustainable gold and platinum group metals production. Further details are given in sections 6.3.1, 6.3.11, 6.4.1, 6.4.2, 6.4.5, 7.2, and 7.9.

**Table 14:** Opportunities for Basic Metallurgical Research Related to Sustainable Gold, Rare Earth, and Platinum Group Metals Production, with Potentially High Leverage on Improved Sustainability

**Process- and mechanism-related research on sustainable gold, rare earth, and platinum group metals production**
Primary synthesis: Metal winning without the use of mercury for amalgams; efficient and less wasteful extraction methods, such as nontoxic leaching processes; better waste management, water usage, and habitat protection
Secondary synthesis, tertiary synthesis, and alloy design measures: Urban mining from electronic, catalyst, and magnet waste; recovery of the metals from dumped mining operations, such as from red mud; new methods for more efficient recovery from nanoscrap where the metals occur in high dispersion; hydrometallurgical and bio-hydrometallurgical recycling of nanoscrap and scrap with high dispersion

## 5 Some Thermodynamic and Kinetic Foundations of Direct Sustainability

### 5.1 Thermodynamic Reduction Analysis: Ellingham Richardson and Baur Glässner Diagrams

In the field of sustainable metallurgy, new raw materials and process conditions have to be considered and studied. Some of the new feedstock has a chemical composition different from those of traditional raw materials, and new reducing agents and their dissolution and oxidation states also change the energetic conditions of the redox relations behind sustainable extractive metallurgy. This makes it necessary to thermodynamically analyze and evaluate the various possible new extraction and reduction paths. In particular, the future avoidance of fossil carbon-carriers as fuels and as reducing agents changes the thermodynamic equilibrium conditions, in some cases quite significantly. Furthermore, some of the new reduction processes envisaged open up the use of new (cheaper and less highly concentrated) ores that have not been taken into account in the classical processes so far and that have a different chemical composition and, therefore, also require consideration of the correspondingly changed equilibrium conditions for reduction.

**Figure 58:**
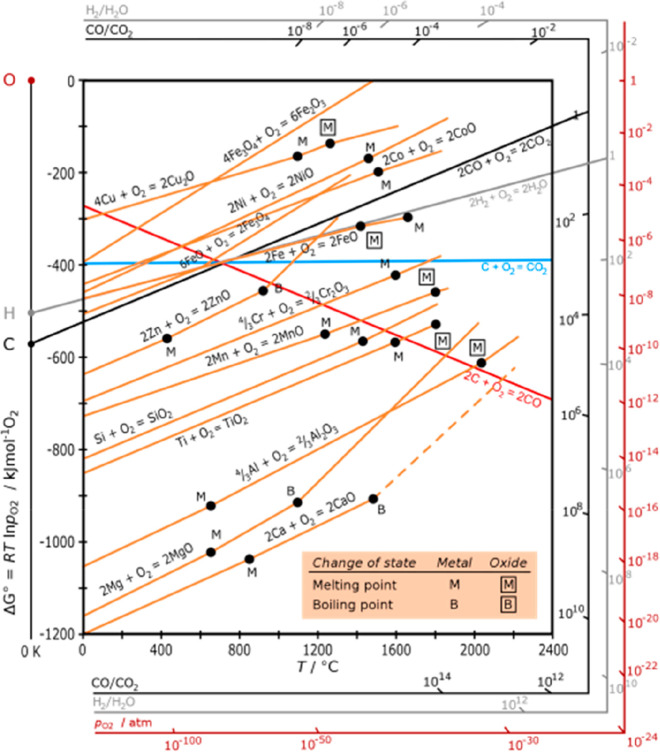
Ellingham Richardson diagram showing the Gibbs free energy of formation vs temperature curves (Δ*G*–*T*) of several important metal oxides and the corresponding oxygen partial pressure in thermodynamic equilibrium. This type of diagram serves as a basis for the selection of suited reductants and temperature ranges for reduction. Similar diagrams can be plotted for assessing the reduction of sulfides and carbonates.

These considerations are formalized via the free energy: the Gibbs free energy (Δ*G*) balance of the relevant oxidation and reduction reactions can be used to answer the questions of how much energy is necessary to fuel a complete redox reaction set, in which direction of a reaction the energy balance points, and which specific reductant (including its dissociated and exited states, for instance in the case of plasma-based reduction) is suited best for extracting a certain metal from its most common oxidized states it assumes in its ores. For metallurgical reactions this is conveniently presented in the form of so-called Ellingham Richardson diagrams, Figure 58. The key quantity Δ*G* is calculated by the enthalpy Δ*H* minus the absolute temperature multiplied by the entropy Δ*S*, i.e. Δ*G* = Δ*H* – Δ*S*. It is important to note that the enthalpy and entropy values change upon phase transformation. Particularly, it must be differentiated between the solid and liquid states when designing reduction reactions.

The Gibbs free energy allows calculation of the thermodynamic driving force for a specific reaction, i.e. if a reaction is spontaneous (Δ*G* < 0, referred to as exergonic) in the forward or reverse direction (Δ*G* > 0, referred to as endergonic) or if it is at equilibrium (Δ*G* = 0). The term “spontaneous reaction” refers to a chemical transformation process that occurs without the use of external energy. Such thermodynamic information about a reaction’s directionality, on the other hand, says nothing about kinetics, i.e. how fast or slow it proceeds.

The enthalpy Δ*H* is a measure for the heat released upon reaction, i.e. of the actual energy that becomes available when the reaction takes place. When negative (Δ*H* < 0, exothermic), the reaction gives off energy, and when positive (Δ*H* > 0, endothermic), the reaction requires external energy to proceed.

The entropy Δ*S* is a measure for the change in the system’s disorder upon reaction; i.e., it counts the change in the number of possibilities for different configurations in the (output) product phases compared to the (input) reactant phases.

If the process is exothermic (Δ*H* < 0) and the entropy of the system increases upon the reaction (Δ*S* > 0), then Δ*G* would always be negative at any temperature as both the enthalpy gain and the entropy increase point in the same direction. Such reaction is always spontaneous. If the process is endothermic (Δ*H* > 0) and the entropy becomes lower upon reaction (Δ*S* < 0), then Δ*G* is always positive and the reaction is never spontaneous.

Exothermic reactions that reduce the entropy of the system are driven by the change in the product phases’ bonding energy. Such reactions are enthalpy-determined, and they are only spontaneous at low temperatures, where the entropy plays a small role.

Endothermic reactions that increase the system’s entropy are driven by the change in the product phases’ disorder. Such reactions are entropy-determined, and they are only spontaneous at high temperatures, where the entropy is weighted highly, Table 15.

**Table 15:** Look-up Table for Trends if a Reaction Is Spontaneous or Not and if It Is Enthalpy- Or Entropy-Driven.

Entropy change (Δ*S*) and enthalpy change (Δ*H*)	Δ*H* < 0	Δ*H* > 0
**Δ*S* > 0**	Exothermic and entropy-driven reaction; spontaneous at any temperature as Δ*G* < 0	Endothermic and entropy-driven reaction; spontaneous at high temperatures where *T*Δ*S* is large
**Δ*S* < 0**	Exothermic and enthalpy-driven reaction; spontaneous at low temperature where *T*Δ*S* is small	Endothermic and entropy-driven reaction; nonspontaneous at any temperature as Δ*G* > 0

These thermodynamic features of a reaction can be visualized in the form of an Ellingham Richardson diagram, which plots the Gibbs free energy of formation versus temperature for metal oxides (or other typical feedstock compounds such as metal sulfides or carbonates etc.) and the corresponding oxygen partial pressure in equilibrium. The Δ*G* values appear as straight lines with the slopes changing upon phase transformation of the compounds. The curves’ slopes represent the entropy changes and the intercepts the enthalpy changes. For the equation C (s) + O_2_ (g) → CO_2_ (g), as one key reaction behind many of the commonly used fossil-based carbothermic reduction processes, the entropy of the solid is practically negligible, so that one molecule of O_2_ gas is resulting in one molecule of CO_2_ gas, at almost no change in the entropy; hence, the slope is nearly horizontal. In contrast the curve 2C (s) + O_2_ (g) → 2CO (g) (the actually active reductant in carbothermic metallurgical reduction processes), where one mole of O_2_ gas yields two moles of CO gas as products, gives a positive entropy contribution, hence the downward slope.

The Ellingham Richardson methodology is used widely in extractive metallurgy and corrosion science to determine what temperature, reduction agent, and pressures are required to reduce metal oxides or sulfides, respectively, depending on ore type, Figure 58.

More specifically, it allows one to identify (i) the energy costs for reducing a particular metallic oxide (or sulfide etc.) into metal, (ii) the partial oxygen pressure in equilibrium state with that oxide at a specific temperature, and (iii) the ratio of carbon dioxide to carbon monoxide (or of other reductants such as hydrogen, ammonia, methanol, or their exited plasma species) which is capable of reducing such metal oxides into metal at a certain temperature.

Reactions in the upper portion of the diagram show metal oxides which are rather easy to reduce (i.e., Cu, Fe), and those in the lower portion show the oxides of very reactive metals (with oxygen and related oxidants), which are difficult to reduce (i.e., Mg, Al, Ti).

**Figure 59:**
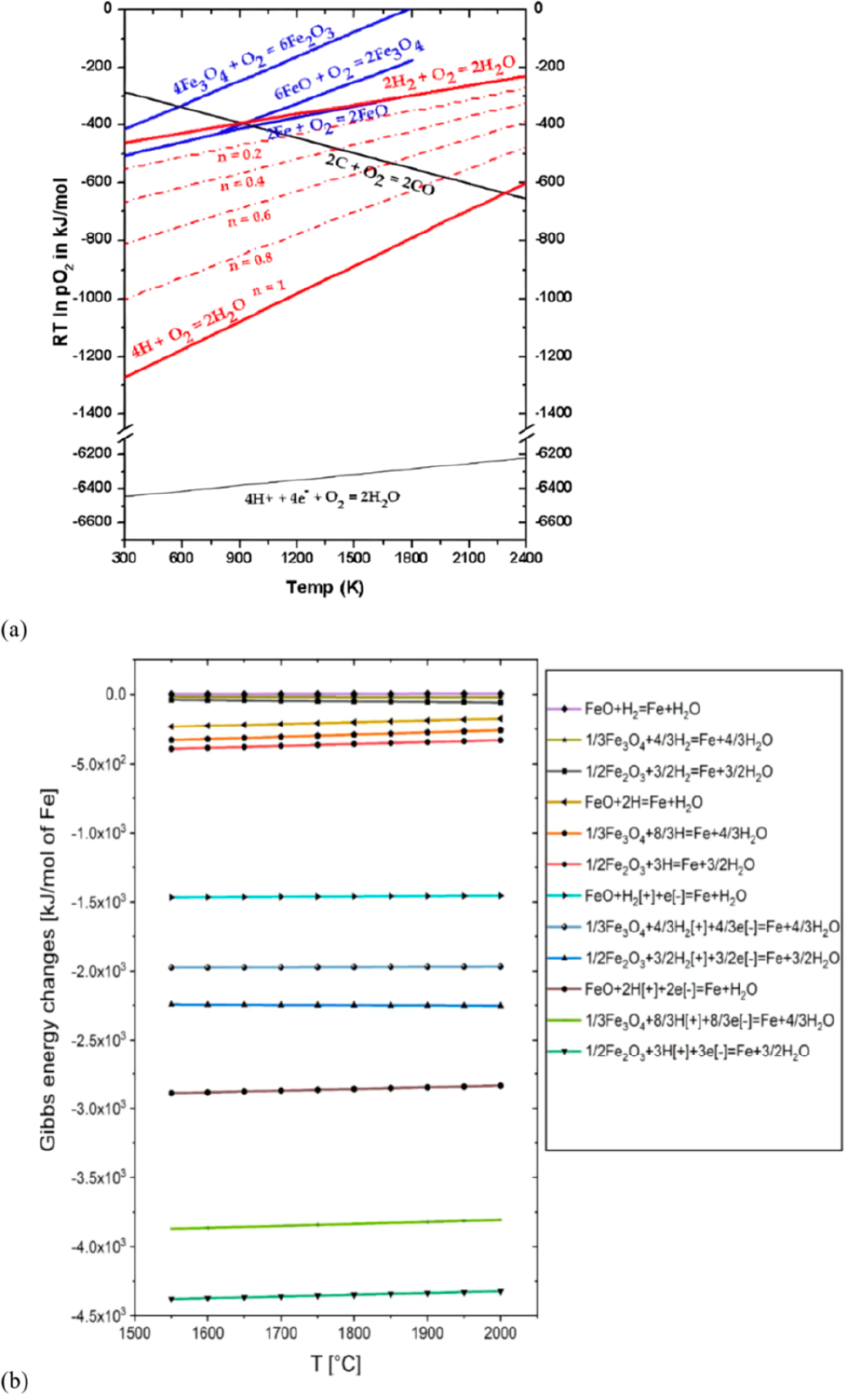
(a) Version of the Ellingham Richardson diagram by Sabat and Murphy^237^ for the reduction of iron oxides with hydrogen plasma, covering the solid-state temperature range for the oxides (solid-state plasma reduction). Note that the temperature axis is plotted in Kelvin. This type of analysis is important when exploiting exited states from a hydrogen-containing plasma for solid-state iron oxide reduction. The index “n” shown in the diagram refers to the influence of different levels of dissociation of the H_2_ molecule into 2 H atoms. The curve at the bottom shows the free energy data for the proton H^+^. The figure is reprinted with permission from ref (237). Copyright 2017, Springer Nature. (b) High temperature version of the Ellingham Richardson diagram by Naseri Seftejani and Schenk^238^ for the case of a hydrogen-based plasma smelting reduction; i.e., in this case the oxides are liquid. Note that the temperature axis is plotted in degrees centigrade. The data show the Gibbs free energy changes for reduction of different liquid-state iron oxides, namely, hematite (Fe_2_O_3_), magnetite (Fe_3_O_4_), and wüstite (FeO) by different types of exited hydrogen species as a function of temperature, calculated by using the thermodynamic software package FactSage 7.1 in conjunction with the database FactPS 2017. The data show the sequence in the reduction potential of different hydrogen plasma species. The data also reveal that liquid FeO is more stable when exposed to hydrogen than the other commonly used liquid iron oxides. For both cases, i.e., solid and liquid oxides, it should be noted that when hydrogen-containing plasma mixtures are used for the reduction, the gas is by no means completely turned into a plasma, but only a certain fraction of the hydrogen occupies ionized and excited states. The distribution function prevailing in each case must be taken into account when calculating the respective energy balance and the reaction rates. The figure is reproduced with permission from ref (238) under an open access Creative Common CC BY license. Copyright 2018, MDPI.

Figure 59 shows variants of Ellingham Richardson diagrams for the case of hydrogen-based (“green”) iron and steel making (a) from the work of Sabat and Murphy^237^ (for the case of the reduction of solid-state iron oxides with hydrogen plasma) and (b) from the paper of Naseri Seftejani and Schenk^238^ (for the case of liquid-state reduction of different iron oxides with hydrogen). More specifically, Figure 59(a) shows the Gibbs free energy change for the reduction of solid iron oxide species by using hydrogen in different dissociated and exited (ionized and vibrational) states. While the reduction of iron oxide with molecular hydrogen gas H_2_ is thermodynamically not favored, the use of certain dissociated and excited states, such as for instance realized through a hydrogen-containing plasma, makes the reaction take place spontaneously. Typical species found in hydrogen-containing plasmas are for instance H^+^, H, H_2_, H_2_^+^, H_3_^+^, and H* (ionized, indicated by +; vibrational, indicated by *). This type of thermodynamic analysis becomes very important when using exited states in a hydrogen-containing plasma for solid-state iron oxide reduction, for instance by using a microwave plasma setup. The index “n” in the diagram refers to the influence of different levels of dissociation of the H_2_ molecule into 2 H atoms. The curve at the bottom shows the free energy data for the first exited state, viz. for a proton H^+^.

In contrast to this solid-state analysis, Figure 59(b) shows hematite (Fe_2_O_3_), magnetite (Fe_3_O_4_), and wüstite (FeO) in their respective liquid states, for analyzing possible reduction reactions when exposed to different types of hydrogen species as a function of temperature. Such reaction scenarios are referred to as plasma smelting reduction. The data show the sequence in the reduction potential of different hydrogen plasma species, revealing that liquid FeO is more stable when exposed to hydrogen than the other commonly used liquid iron oxides.

When using Ellingham Richardson diagrams for an energy assessment of reduction reactions, a few limitations are worth noting; namely, the diagram presents equilibrium energy values. This means that kinetics is not considered; i.e., these diagrams do not allow conclusions about reaction rates. Also, no nucleation barriers are considered. It is further worth noting that when using non-fossil reductant gases and their respective excited states (in the case of plasma-assisted reduction), the progressing reduction reactions, which are creating an increasing amount of metal among the oxides, can introduce catalytic effects as an additional factor into the reductant dissociation and reaction rates. This can change the reaction rates and energy barriers quite substantially. This means that for the design of real-world reactors, particularly in conjunction with non-fossil reductants, the influence of nucleation, dissociation, and catalysis must be taken into consideration. Another important boundary condition of high relevance for the overall reaction kinetics under real reactor conditions lies in the microstructure and size of the oxides.^239,240^ These features significantly determine the diffusion rates not only of the inbound diffusion of the hydrogen but also particularly of the outbound diffusion of the oxygen. Particularly the latter quantity is the main kinetic bottleneck in the case of hydrogen-based solid-state reduction.^241^ Another kinetic factor that seems to be quite important, at least in the hydrogen-based direct reduction of metal oxides, is the internal storage of the redox product, i.e., of the water. While under equilibrium and open surface conditions it could be assumed that the water is removed from the reaction front, in experiments it is instead observed that the water gets stored inside of the reduced material, specifically inside of the pores (up to 50% of the volume). These pores continuously form and coagulate as a consequence of the mass loss caused by the oxygen removal from the oxide. It was recently observed that this undesired containment and storage of the redox product (water) can substantially reduce the overall reaction kinetics. All these microstructural aspects are not considered in these equilibrium thermodynamic diagrams, which only show the thermodynamic direction of a reaction.

**Figure 60:**
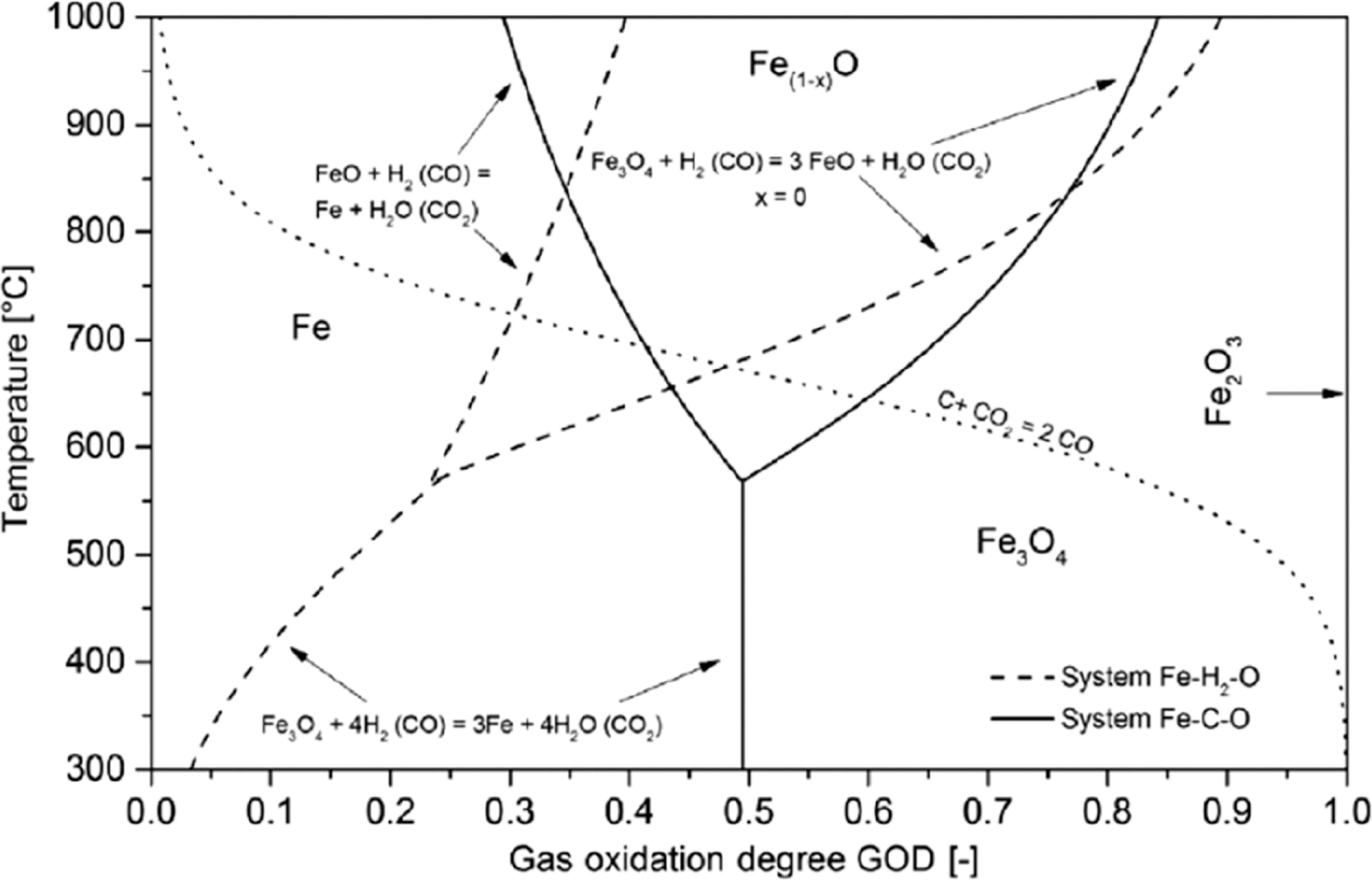
Baur–Glässner diagram for the reduction of iron oxide with either carbon monoxide (Fe–O–C system) or hydrogen (Fe–O–H_2_ system) including the Boudouard equilibrium for the case of 1 bar and a carbon activity of 1.^179^ The figure is reproduced with permission from ref (179). Copyright 2019, Steel Research International, Wiley.

Another helpful thermodynamic equilibrium diagram is the so-called Baur–Glässner diagram, which can be derived from the Ellingham Richardson diagram, Figure 60. It shows the equilibrium existence ranges of a metal and its different oxidation states at different temperatures and partial pressures of the reductant or reductant mixtures. It is particularly interesting for practical applications such as required for instance for the design of reduction reactors with respect to the optimal working points regarding the chosen feedstock oxides, the reduction agent mixtures, and the temperature.

Knowing the existence ranges and sequence of certain oxides during a reduction process is specifically important, as it provides a basis for identifying the relevant associated structural and transport properties of the different oxide states, such as the respective diffusion coefficients for hydrogen and oxygen. It also provides important information about the influence of certain ratios between the reductant and the redox product.

### 5.2 The Role of Kinetics in Sustainable Metallurgy

Similar to the new types of raw materials and the new resulting redox systems mentioned in the previous chapter with regard to a thermodynamic evaluation, the same applies to the consideration of kinetics. The various possible new process routes and raw materials in the field of sustainable metallurgy also lead to different kinetic conditions. Examples are the transport of hydrogen, oxygen, and water in the porous (partially) oxidic solids in the field of hydrogen-based direct reduction of iron ore with the help of hydrogen-containing reduction mixtures or the use of new ionic liquids in the field of hydrometallurgy.

**Figure 61:**
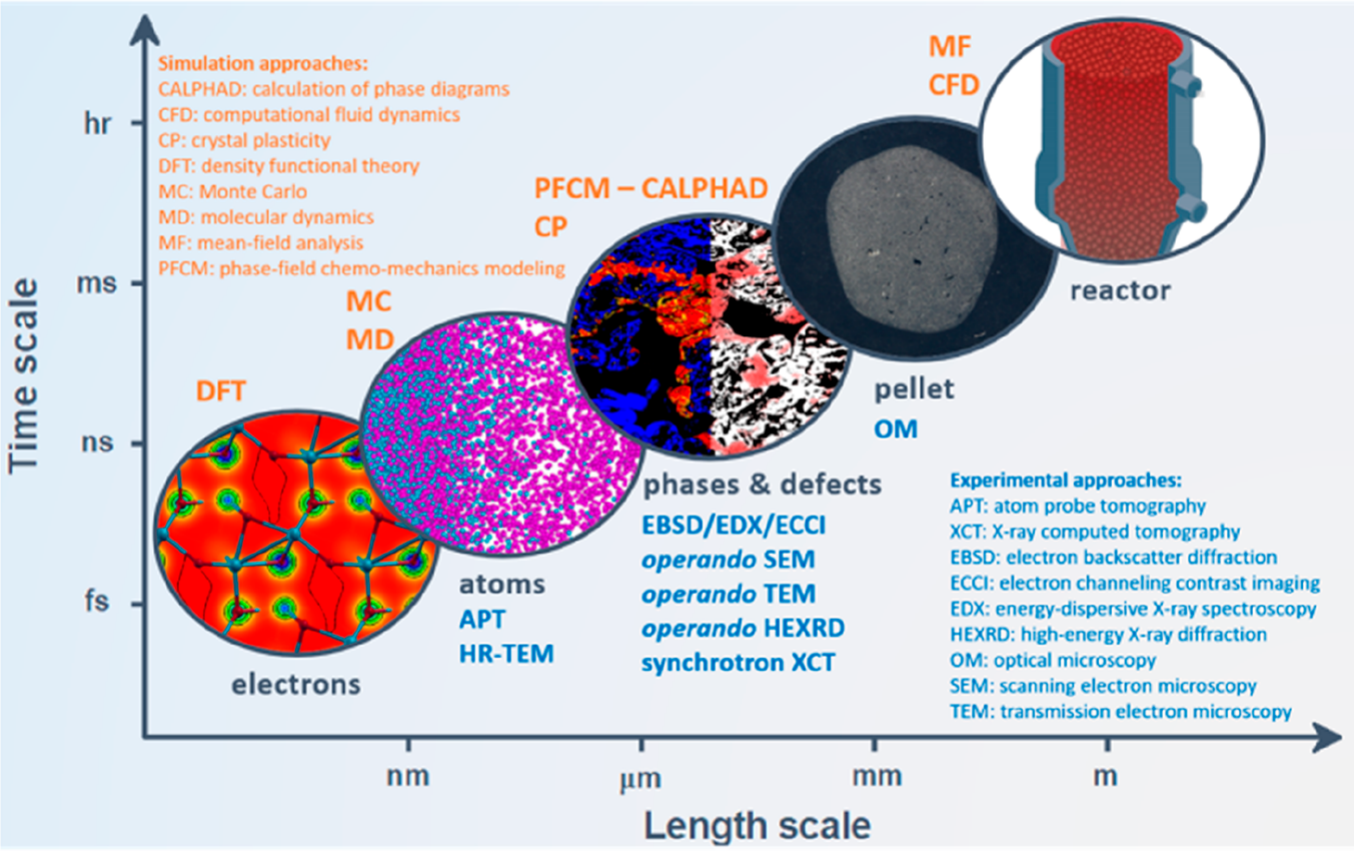
This figure shows an example of the multiple scales in the context of kinetic and thermodynamic simulations (above the images) and experiments (below the images) for the example of the solid-state direct reduction of iron oxides by using hydrogen.^182^ The figure is reproduced from ref (182) with permission. Copyright 2022, Elsevier.

This chapter does not provide an exhaustive overview of transport, nucleation, and growth kinetics in metallic materials, as this is covered by standard textbooks, but it highlights some important kinetics aspects that are of particular relevance for the direct sustainability of metallurgy. In this context, different research topics in the field of kinetics can be distinguished, Figure 61.^182^

**Figure 62:**
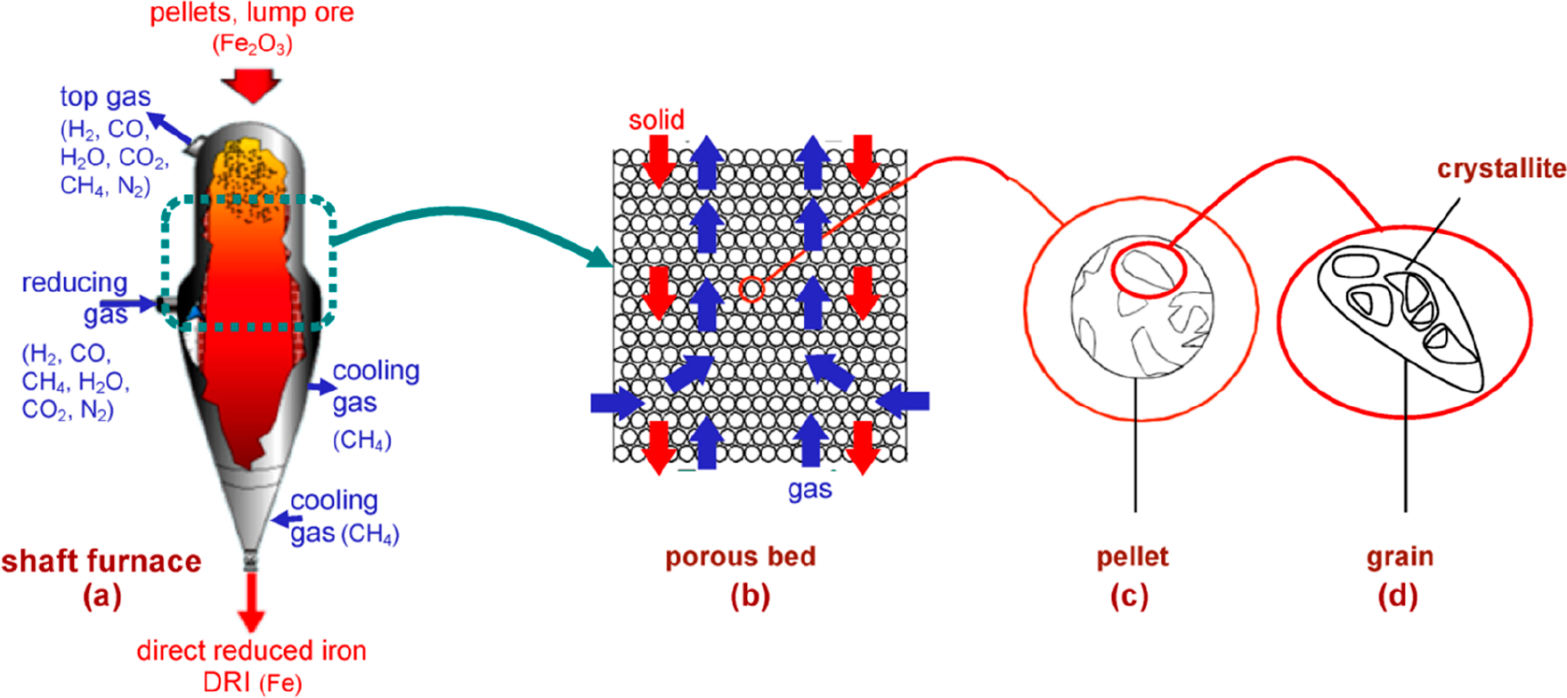
Different scales considered for the simulation and experimental analysis of a (hydrogen-based) iron oxide reduction reactor. (a) Shaft reactor model for formulating and solving the macroscopic thermal, balance, and chemical reaction equations. (b) Gas percolation simulation though the porous bed of the iron oxide pellets. (c) Pellet scale simulation. (d) Crystal-scale simulation.^242^ Figure is reproduced with permission from ref (242) under an open access Creative Common CC BY license. Copyright 2020, MDPI.

**Figure 63:**
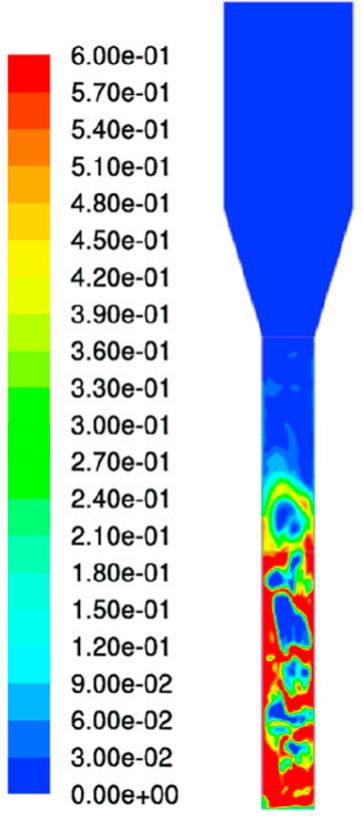
Computational fluid dynamics simulation of iron oxide reduction kinetics for the boundary conditions of a large-scale fluidized bed reactor.^243^ The color code refers to the solid volume fraction in the simulated reactor. The figure is reproduced with permission from ref (243). Copyright 2020, Steel Research International, Wiley.

**Figure 64:**
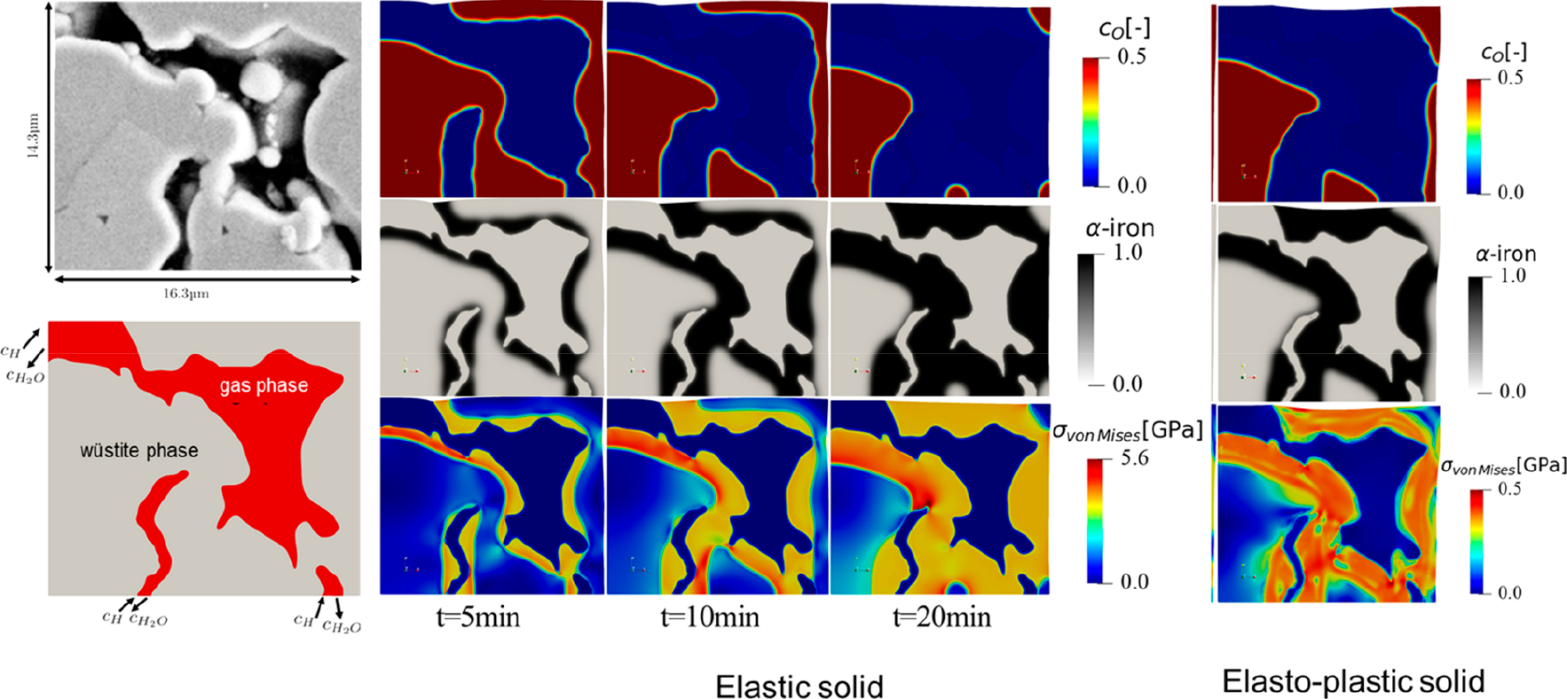
Chemo-mechanically coupled phase field simulation.^183^ The calculations are based on the Cahn–Hilliard (for the conserved species) and Allen–Cahn (for the non-conserved species) theories and allow the simulation of kinetics and thermodynamics under consideration of the mechanical stresses arising from the phase transformation and oxygen mass loss during the reduction (here simulating the hydrogen-based reduction of wüstite into bcc iron). The images on the left-hand side show the microstructure patch used for setting the boundary conditions for the phase field simulations. The middle part shows the phase field simulation results under the assumption of a purely linear elastic constitutive response of the solids, and the simulations on the right-hand side show the results for an elastic-plastic material response. The three rows refer to the oxygen concentration (upper row); the formation of the bcc iron phase (middle row, iron plotted in black); and the von Mises equivalent stress (bottom row). The figure is in modified form reproduced with permission from ref (183). Copyright 2022, Elsevier. bcc, body centered cubic crystal structure, referring to the ferritic iron phase.

(i) Macroscopic consideration of mass transport, percolation, temperature, and chemical reaction kinetics. On the one hand, in the field of green metallurgy, especially in the design of reactors, macroscopic methods for balancing material flows and heat transport are of great importance, i.e. for example fluid and gas dynamics inside of large-scale reactors and porous media. Here, not only material transport must be considered, but also the local temperature conditions and the most important chemical reactions of the system, which often differ across the macroscopic dimensions of a reduction reactor.^242^ Such simulations are usually based on the solution of the chemical reaction kinetics based on the local boundary and initial conditions, the classical Navier–Stokes equation or corresponding mesoscopic variants such as the Boltzmann lattice method and the associated local energy balance, Figure 62.^242^Figure 63 shows an example where such a simulation has been developed for fluidized bed reactors, using a dense discrete phase model.^243^ Important research questions in this context are, for example, the consideration of mineral raw materials with different sizes and degrees of gangue impurity content as well as mixed use of different reducing and fuel agents in the same reactor. The latter aspect is of particular relevance for sustainable metallurgy because many of the underlying reactions that use for example hydrogen as reductant are endothermic and not exothermic so that often additional fuels might be needed to maintain the reactors’ operation temperature in a processing window of high metallization and efficiency.
(ii) Mean-field diffusion simulations of the respective reactants through gases, melts, and solids, usually solved for simplified boundary conditions as well as core–shell models or simple spherical symmetry conditions. For the calculation of reduction kinetics in the mesoscopic range, e.g. at the level of individual oxide particles exposed to a corresponding reduction medium, core–shell models are usually formulated and solved on the basis of classical solid-state diffusion equations, Figure 62 and Figure 63.
(iii) Mean-field calculations of phase transformations considering either a nucleation-based or a spinodal transition mechanism and of the corresponding interfacial mobility, for example via statistical Avrami-based models of nucleation and phase growth. Physics-based simulations which consider all the underlying transport, phase transformation, redox, and mechanical mechanisms can help in that context, Figure 62.
(iv) Mesoscopic models involving chemo-mechanical coupling, e.g. based on phase field theory, Figure 64.^183^ Such models can also be coupled with corresponding gas dynamics or fluid dynamics models for conducting simulations for scenarios that involve porous media.^183^
(v) Microscopic models of atomic mass transport as well as chemical reactions on the basis of atomistic calculations with the help of simplified interatomic potentials on the basis of molecular dynamics. So-called reactive force field models can also be used to simulate certain chemical reaction steps in such calculations.
(vi) Electronically based ab initio models that can represent the details of reactive chemistry, such as density functional theory together with kinetic Monte Carlo calculations.

### 5.3 General Electrochemical Aspects of Primary Metal Synthesis and Recycling

**Figure 65:**
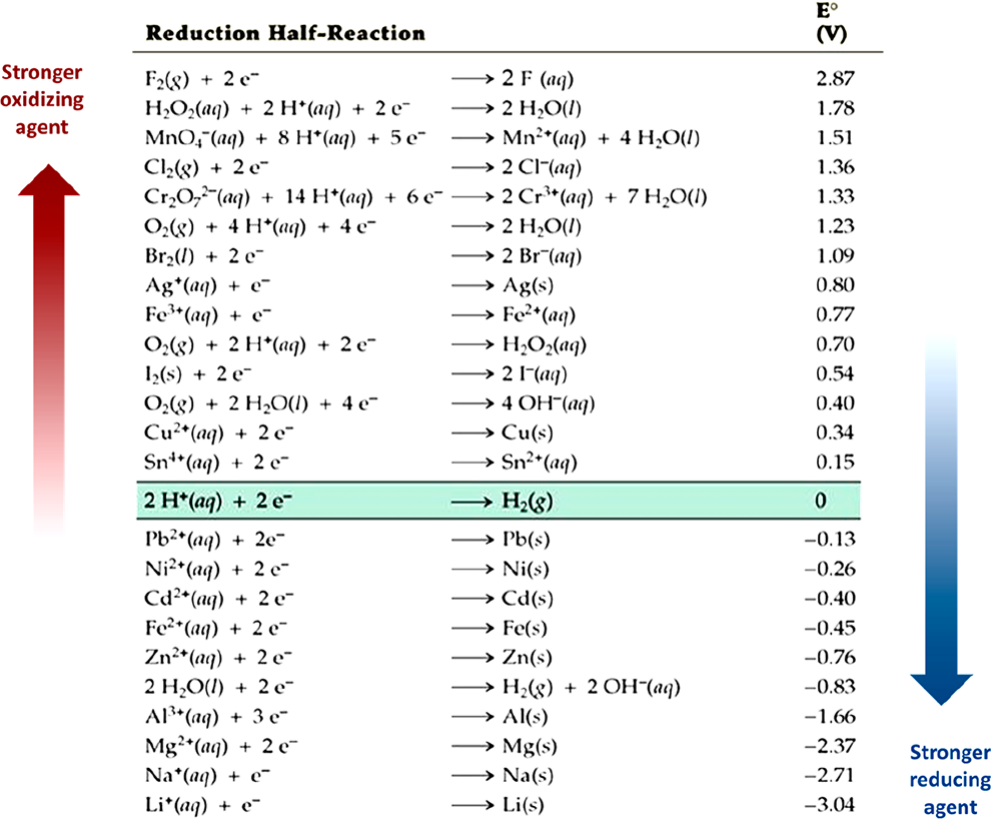
Standard reduction potentials of a number of metal electrodes quantified using a standard hydrogen electrode as reference electrode.

Electrolysis uses electrons to separate compounds dissolved in a conductive liquid that acts as an electrolyte. Direct current is passed between two electrodes immersed in the electrolyte. The voltage source causes a shortage of electrons in the electrode connected to the positive pole (anode) and an excess of electrons in the other electrode connected to the negative pole (cathode). The solution placed between the two electrodes contains positively or negatively charged ions as charge carriers that constitute the electrolyte’s properties. The positively charged cations migrate to the negatively charged cathode when a voltage difference is applied between the two electrodes. At the cathode, the ions accept one or more electrons and are thus reduced. The opposite process takes place at the anode. There, the negatively charged anions loose electrons; i.e., they are oxidized. The amount of electrons transferred at the anode is equal to the amount transferred at the cathode. According to Faraday’s law of electrolysis, the mass of a substance that gets deposited at an electrode during electrolysis is linearly proportional to the charge, i.e. to the intensity of the electrical current multiplied by the time, Figure 65.

Electrolysis is an important metal extraction method in metallurgy and results in the deposition of reaction products from the chemicals in the electrolyte at the electrodes. It is specifically used in this field for the electrolytic dissolution of metals in or separation from aqueous media, for deposition, or for metal purification. Electrolytic gold and copper refining from dissolved electronic scrap are examples of this.

The lowest theoretical voltage value that must be applied for electrolysis is the decomposition voltage. However, a higher voltage is typically required for the process to happen. The redox potential can be used to calculate the necessary deposition potential. One can get further indicators from the redox potential, such as for the acid-induced electrolytic dissolution of metal electrodes or the lowering of the decomposition voltage.

The sequence in cathodic separation tendency for metal extraction from mixed (scrap-originated) hydrometallurgical solutions is in principle determined by the electrochemical series, Figure 65. If there are several reducible cations in an electrolyte solution (such as in solutions produced for instance from electronic scrap), the cations that have a more positive (less negative) potential in the redox series (voltage series) are reduced first. Normal electrolysis of an aqueous saline solution results in the formation of hydrogen, not sodium, at the cathode. Even though there are various anions that can be oxidized, the ones that are employed first are those that are closest to the voltage zero point in the redox series or that have a weaker positive redox potential. Thus, normally, when aqueous NaCl is electrolyzed, oxygen rather than chlorine is produced at the anode. After exceeding the decomposition voltage, the strength of the electric current increases proportionally with the increase in voltage.

The value of the voltage, also referred to as overpotential (or sometimes also as overvoltage), is crucial in addition to the redox potential. Voltages that are much higher than those predicted by redox potentials are frequently needed to achieve kinetic inhibitory effects at the electrodes. The overpotential is thus the potential difference between the thermodynamically determined reduction potential of the half-reaction and the voltage at which the desired redox event is actually observed experimentally, i.e. the measured voltage difference. Depending on the material properties of the electrodes, the overpotential effects can also change the redox series, so that other ions are oxidized or reduced than would have been expected according to the redox potential. These aspects are particularly essential when confronted with the task to electrochemically recover metals from rapidly growing multi-metal waste material streams that are for instance produced when scrapping electrical and electronic equipment.

Overpotentials can occur at the cathode as well as at the anode and thus increase the required voltage compared to the calculations according to the Nernst equation. The values for the required overvoltage are sometimes considerable, for instance in the case of gas formation (e.g., hydrogen and oxygen formation). The applied overvoltage energy is lost as dissipative heat, and therefore, it does not contribute to material recovery, thus reducing the efficiency of the electrochemical extraction process. The overpotentials also vary depending on the type of metal. The electrical current strength and the temperature also influence the magnitude of the overpotentials. Also, an increase in the imposed current slightly increases the overvoltage, whereas an increase in temperature tends to decrease it.

For some metals, mostly those with high oxygen affinity, electrolysis is the primary synthesis and metal recovery method. For example, aluminum and magnesium are produced using fused-salt electrolysis, where the metal oxides form low-melting mixtures with a salt-like electrolyte (see section 7.7.4). Copper, silver and gold can be also produced and refined electrochemically, particularly when recovered from electrical and electronic scrap (see section 7.7.7). Also zinc and nickel can be won and purified electrochemically. Several alkali metals and most alkaline earth metals are also obtained by fused-salt electrolysis. Electrochemical aluminum production makes by far the largest volume of material won by electrolysis in metallurgy and hence deserves particular attention in the context of sustainability.^154,244−248^ Recently, driven by the approach to use more renewable electrical energy directly for metal recovery via mineral reduction, without the efficiency losses associated with producing renewable chemical reductants such as hydrogen, electrochemical iron winning has also been pursued as an alternative synthesis pathway (see details in section 7.7.5).^249−252^

### 5.4 The Nernst Equation

The Nernst equation is a fundamental electrochemical equation that describes the relation between the standard electrode potential and the actual potential of a redox reaction at nonstandard conditions, for instance for nonstandard temperature and concentration conditions. The equation can be used for the calculation of the voltage of an electrochemical cell and for the determination of the thermodynamic feasibility and direction of a redox reaction.

The Nernst equation is essential for the electrochemistry of metallurgical reactions because it connects the first central quantity, namely, the electrode potential (voltage), with the second main quantity of relevance, the chemical concentration. This is required for instance to deal with questions such as the electrochemical reduction of molten salt mixtures used in the aluminum industry for primary electrowinning from oxides (see sections 6.2.6 and 7.7.1–7.7.4) or for the electrochemical recovery of dissolved and mixed chemical elements from scrapped electronic circuits (see section 7.7.7).

More specifically, the Nernst equation relates the dependence of the equilibrium electrochemical potential of the electrode, *E*_eq_, for each half-cell reaction in an electrochemical cell to its standard potential, *E*_0_, based on the Gibbs free energy of a redox reaction, Ox + *z*e = Red, on the chemical activities (concentrations; or partial pressures in the case of gases) of the substances involved and the temperature, according to *E*_eq_ = *E*_0_ – (*RT*)/(*z*_e_*F*) ln(activity of reduced species/activity of oxidized species), where *R* is the gas constant (8.3145 J K^–1^ mol^–1^), *T* is the absolute temperature, *z* is the number of moles of electrons involved in the reaction, and *F* is the Faraday constant (96485 C mol^–1^). The notation “activity of reduced species” represents the product of the activities (concentrations, partial pressures) of all the species that appear on the reduced side of the electrode reaction, raised to the power of their stoichiometric coefficients. The notation “activity of oxidized species” represents the same measure for the oxidized side of the electrode reaction. The Nernst potential *U*_0_ multiplied by the charge *zF* for a molar mass transfer *zFU*_0_ gives the Gibbs energy Δ*G* = −*zFU*_0_. The Nernst potential thus gives the chemical energy of the electrochemical reaction divided by the charge involved.

### 5.5 The Pourbaix Diagram in Electrometallurgy

**Figure 66:**
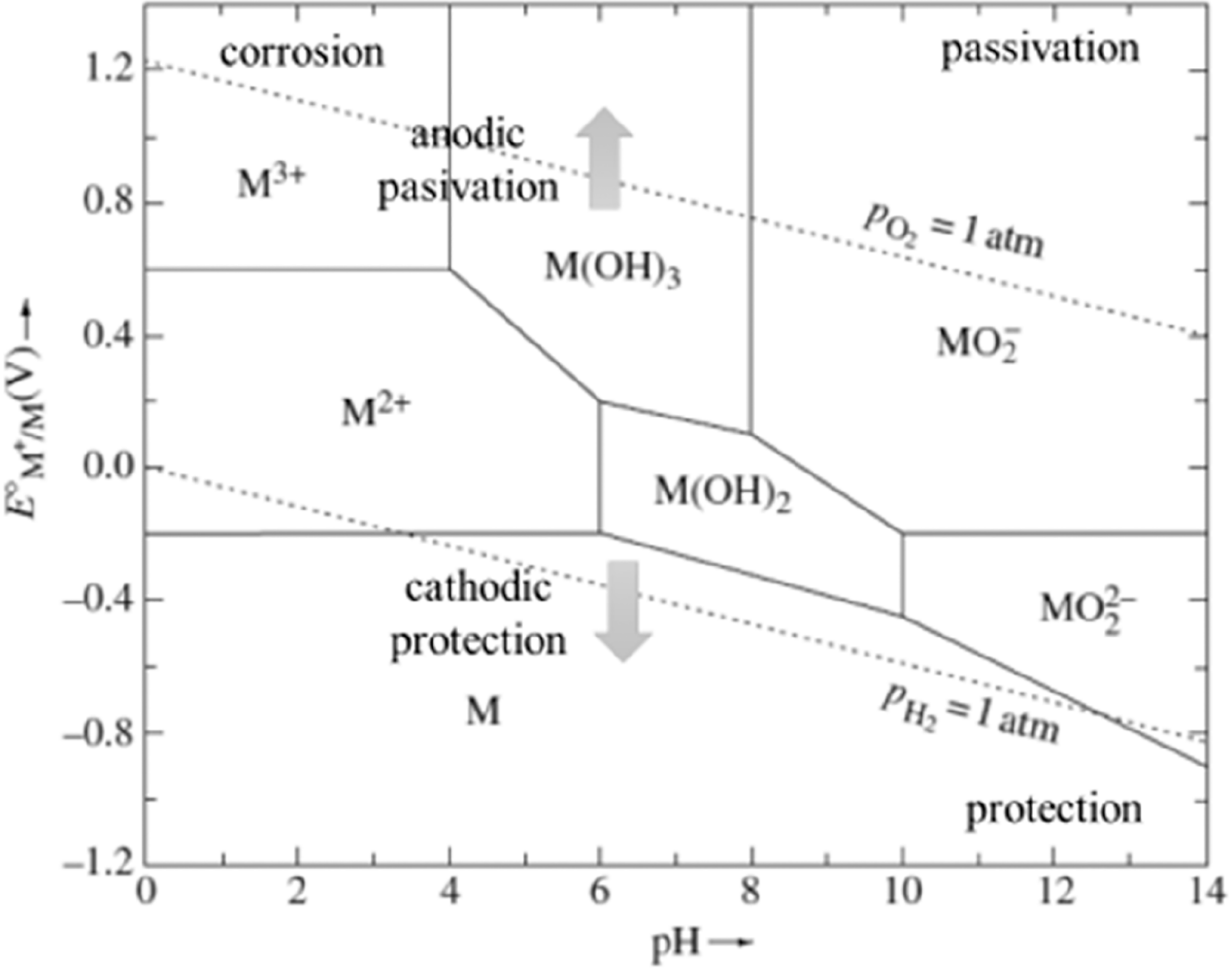
Pourbaix diagram showing regions of phase stability ranges of metals and metal oxides in water as a function of pH and reduction potential.

A Pourbaix diagram, also referred to as electrochemical potential diagram or *E*–pH diagram, is a graphical representation of the stability of different chemical species in aqueous solution as a function of both the redox potential (*E*) and the pH value. It is used in electrochemistry to predict the speciation of aqueous solutions and to evaluate the thermodynamic feasibility of redox reactions under different conditions of pH and imposed electrochemical potential. The diagram shows the regions of stability of different oxidation states of a metal ion or molecule and can be used to predict the electrochemical stability ranges of metals and oxides and, hence, of a material’s corrosion or reduction behavior for certain potential and pH combinations, Figure 66.

With these features, Pourbaix diagrams assume a central role in electro- and hydrometallurgy, where they are used to graphically represent thermodynamic equilibrium stability ranges in metal–electrolyte systems, where the pH value is ranged on the abscissa and the potential, determined by the Nernst equation, on the ordinate. Pourbaix diagrams are usually plotted for a standard temperature of 25 °C and a reference concentration of 1 mol^–1^. Pourbaix diagrams can thus not only be used to predict potential and pH conditions that are needed to solubilize metals and metal oxides, but they serve also as tools to calculate under which conditions metals can be recovered from different types of metal-bearing species and solutions.

In electrometallurgy they allow us to assess the stability ranges of metals and ions in aqueous solutions. The boundaries among different regions in such potential–pH diagrams show the transitions between different thermodynamically stable species (all in equilibrium). The potentials for the evolution of H_2_ and O_2_ are commonly shown both at 1 atm pressure.

To construct a Pourbaix diagram, the underlying Nernst equations are used. As the Nernst equation is a thermodynamic equilibrium equation, the Pourbaix diagram thus also shows which species is thermodynamically stable at a given voltage and pH value. Both, the Nernst equation and the Pourbaix diagram make no statements about metastable states or the kinetics of the electrochemical processes.

To express this more practically, when placing a metallic material into contact with an aqueous solution, it becomes an electrode. Its tendency to dissolve in the solution into the electrolyte is measured by its electrode potential. In addition to the electrode potential, the pH value of the solution has a profound effect on the product of the anodic dissolution. Generally, a metal ion concentration of 10^–2^ or 10^–3^ mol kg^–1^ of water is taken as a boundary condition when constructing Pourbaix diagrams for hydrometallurgical applications.

In Pourbaix diagrams three main regions can be commonly distinguished, namely, the corrosion, the passivation, and the immunity regimes. The corrosion range is characterized by a proportion of the dissolved metal ions above 10^–6^ mol L^–1^. The passivity regime is characterized by the formation of stable oxides and/or hydroxides, usually with high adhesive strength, which protect the material against further oxidation. In the immunity range, the value of the dissolved metal ions is below 10^–6^ mol L^–1^.

### 5.6 McCabe–Thiele Diagram for Solvent Extraction

**Figure 67:**
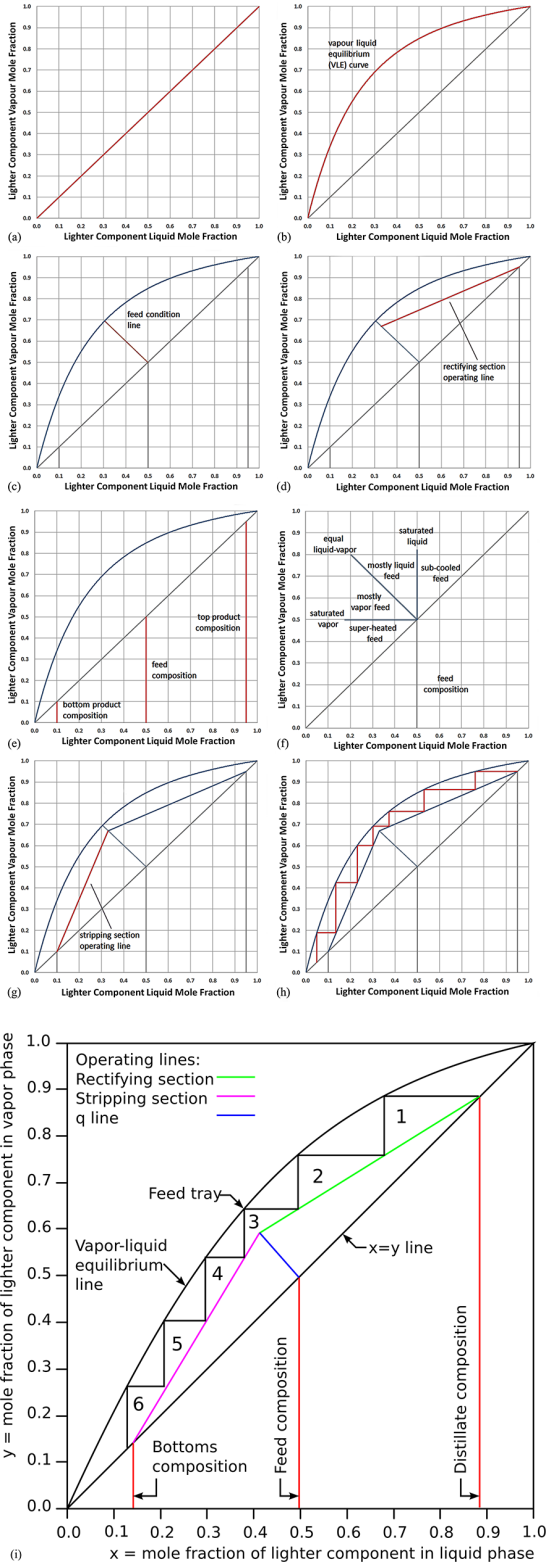
McCabe–Thiele diagram: graphical representation for the thermodynamic equilibrium for vapor–liquid phase mixtures in each stage of a solvent extraction process. This diagram is an important tool for the design of hydrometallurgical extraction processes.

A typical question in sustainable metallurgy is how to extract a metal ion from a solution, a problem known in chemistry as distillation. For such processes the McCabe–Thiele diagram can be constructed and used. This diagram is a graphical representation of the vapor–liquid equilibrium in each stage of an extraction process, Figure 67. It helps us to understand the equilibrium thermodynamics of metal separation and to calculate the number of trays required for the distillation of a binary mixture (under thermodynamic equilibrium conditions). It also allows us to analyze the impact of reflux rate, feed composition, product composition, and vapor–liquid equilibrium on extraction reactor design.

It is constructed based on differences in volatility or vapor pressure between the solvent and the metal in solution. The construction uses the fact that the composition of a binary solution from which a metal is to be extracted at each point of equilibrium is determined by the mole fraction of one of the two components. It then allows us to analyze the case of constant molar overflow, based on certain constraints. These are: (a) the solution is a binary mixture; (b) the heats of vaporization of the two components are equal, which means that if one mole of the heavier component is vaporized, one mole of the lighter component is correspondingly condensed; (c) negligible heat of dissolution; and (d) 100% tray efficiency. These constraints qualify calculations according to the McCabe–Thiele plot as theoretical or equilibrium trays.

The data needed for the construction of a McCabe–Thiele diagram are the vapor–liquid thermodynamic equilibrium data for the two components in binary solution and its chemical composition, the elements’ boiling and dew points, and the temperature of the solution.

For the construction of the McCabe–Thiele plot, with the abscissa showing the mole fraction of the lighter component in the liquid phase and the ordinate giving the mole fraction of the lighter component in its vapor phase, several steps must be taken. A 45° line is first plotted to connect the origin to the top right corner of the diagram as shown in Figure 67(a). Next the vapor–liquid equilibrium curve for the specific binary solution of interest is drawn, Figure 67(b). Then the compositions of the feed and the top and bottom products are marked on the abscissa and a vertical line is plotted from each of these points to the 45° line, Figure 67(c). This chart helps to determine the relationship between vapor fraction and extraction process feed temperature using the feed condition line. This line passes through the intersection of the 45° line and the feed composition line. Its inclination can be calculated as a function of the properties of the feed. At the position where the feed assumes a value of 50 mol % liquid, the line will be perpendicular to the 45° line. When the feed is a saturated liquid, the line is vertical, and when it is a saturated vapor, it is horizontal. For feeds between a saturated vapor and a saturated liquid, one can use a ratio to determine the slope of the feed condition line, *m* = *q*/(*q* – 1), with *q* being the mole fraction of the liquid in the feed, Figure 67(d). For feeds outside this range (e.g., undercooled or overheated), one has first to determine the extent of this and then feed the result (*q*) into the equation to determine the slope of the feed condition line. For an undercooled substance, the slope is between 45° and vertical, and for overheated vapor, it is between 45° and horizontal. After calculating its slope, the feed condition line is plotted into the chart from the point where the feed line meets the 45° line until it intersects the vapor–liquid equilibrium curve, Figure 67(e).

When vapor leaves the reactor, it is cooled and liquefied. Some of this stream is taken away as the top product while the rest is returned as new feed. This reflux diffuses down the reactor in the opposite direction to the rising vapor that leaves the reactor chamber. The liquid thus swaps heavy components in the vapor for light components in the liquid, concentrating the light component in the vapor. The rectifying section operating line describes the amount of liquid sent back down the rectifying section as reflux. Due to assumptions of the McCabe–Thiele method, the operating line estimates how much the composition can change at each tray: increasing reflux results in bigger steps and thus less trays, Figure 67(f). As with the rectifier section, the working line of the stripping section represents the gas that returns to the reactor column after leaving the reboiler. This hot gas evaporates the light components in the liquid, which move along the column against the condensation of the heavy components of the gas.

To plot the operating line for the stripping section of the reactor, design one has to start at the point where the vertical bottom product line meets the 45° line and then plot a line to the point where the rectifying section operating line meets the feed condition line. Once the rectifying and stripping section lines are completed, the theoretical trays are drawn. Starting from the point where the top product line meets the 45°, a horizontal line is drawn until it intersects the vapor–liquid equilibrium curve. A vertical line is then drawn down from this point until it meets one of the two operating lines. This process is repeated until the last vertical line falls to the left of the bottom product line, Figure 67(g). The theoretically required number of trays for such a scenario can then be determined by counting the number of times the horizontal steps touch the vapor–liquid equilibrium curve.

### 5.7 Plasma Reductants in Extractive Metallurgy

A plasma is a mixture of neutral and ionized molecular species, containing molecules, electrons, and (usually multiple) ionic forms of the molecular species of the original gas. A plasma is formed by ionizing a gas by electrical excitation. One can distinguish nonthermal plasmas from thermal plasmas. A nonthermal plasma, also referred to as nonequilibrium or cold plasma, is a plasma that is not in thermal equilibrium among its constituent species. This means that the kinetic energies of the different particle types in the plasma can differ significantly from each other; i.e., the electrons, the neutral molecules, and the ionic species have different temperatures. In a thermal plasma, also referred to as equilibrium or hot plasma, the temperatures of all species are the same, due to frequent collisions and the resulting thermal equilibration.

In non-fossil hydrogen- (or ammonia-) containing metallurgical reduction processes, the use of thermal plasmas prevail; however, they often change into a nonequilibrium state at the reaction interface with molten oxidic mineral mixtures owing to evaporation processes from the reaction interface. Thermal plasmas can reach peak temperatures of up to several 10 thousand Kelvin. Such high temperatures and the high energy density in the plasma plume make it a suitable tool for heating, oxidation and reduction in the field of heat treatments and extractive metallurgy (see section 7.5). Depending on the way in which the plasma is generated, different categories can be distinguished. Most common in metallurgy are direct current plasma torches that can deliver power from about 100 kW up to several MW.

Thermal hydrogen-containing plasma methods for the reduction of metals from their oxidic mineral mixtures can be applied to solids (Figure 59(a))^237^ or to liquids (Figure 59(b)).^145,238^ Due to the high temperature and multiple dissociated, excited, and reactive hydrogen species, thermal plasmas offer, particularly in liquid-state processes, in principle very fast single-step reduction processes, at rather low carbon footprint (depending on the electrode material).^123,143^ Such approaches are also referred to as hydrogen-based smelting reduction processes. These methods are in principle similar to classical electric arc melting processes of metallic scrap, but they use the additional effect of the hydrogen-containing plasma for the reduction when charged with oxides (rather than metallic scrap, which has only to be melted but does not need to be reduced). Recently, also interesting kinetic advantages have been reported for the cases of “low-temperature” hydrogen plasma-assisted solid-state direct reduction processes.^144,253^

In principle most gas species can be ionized and thus brought in plasma form. However, in extractive metallurgy one is in the context of more sustainable approaches primarily interested in hydrogen-based plasmas, as a renewable reductant to recover metals form ores, provided that the hydrogen had been won from sustainable energy sources via electrolysis. Hydrogen-containing plasmas can be produced by the application of direct current, alternating current, radiofrequency, microwave, and other portions of the electromagnetic spectrum. Hydrogen (usually as part of a gas mixture) in plasma form can contain a wide spectrum of exited states. This can include dissociated hydrogen H and a variety of ionized species (such as the proton H^+^ and H_2_^+^, H_3_^+^, etc.) as well as rotational and vibrational excitations (usually denoted as H*). The most frequent exited types occurring in metallurgical hydrogen-containing plasmas are free electrons, ionized hydrogen molecules, atomic hydrogen, and protons.

In contrast to the near-thermal hydrogen-containing plasmas used mostly in plasma-assisted reduction of liquefied oxides, nonthermal plasmas are such plasmas where the heavier molecular species and the fast electrons are not in thermal equilibrium and assume different temperatures. They are also referred to as cold plasma because the gas portion of the mixtures remains at much lower temperature than the electrons. The latter can absorb energy from the excitation source in the same way as in a thermal plasma, but they do not transfer this energy to the heavy molecular species, owing to an insufficient collision frequency. This means that in nonthermal plasmas the electron temperature is by far the highest (at a similar temperature as in a thermal plasma, i.e. up to ten thousand Kelvin and more), exceeding that of the vibrational, rotational or ionic modes.

### 5.8 The Thermodynamics of Plasma-Based Reduction of Metal-Containing Minerals

The thermodynamics associated with using a hydrogen-containing plasma, considering all its different types of radical (ionized) species, can be plotted in the form of an Ellingham Richardson diagram. Like for any other redox process it depicts the energetic balance of the reduction reaction from the Gibbs standard free energy change among the redox reaction partners, considering their respective oxidized and reduced states. When the energy balance is negative, the reduction can take place (in thermodynamic equilibrium).

**Figure 68:**
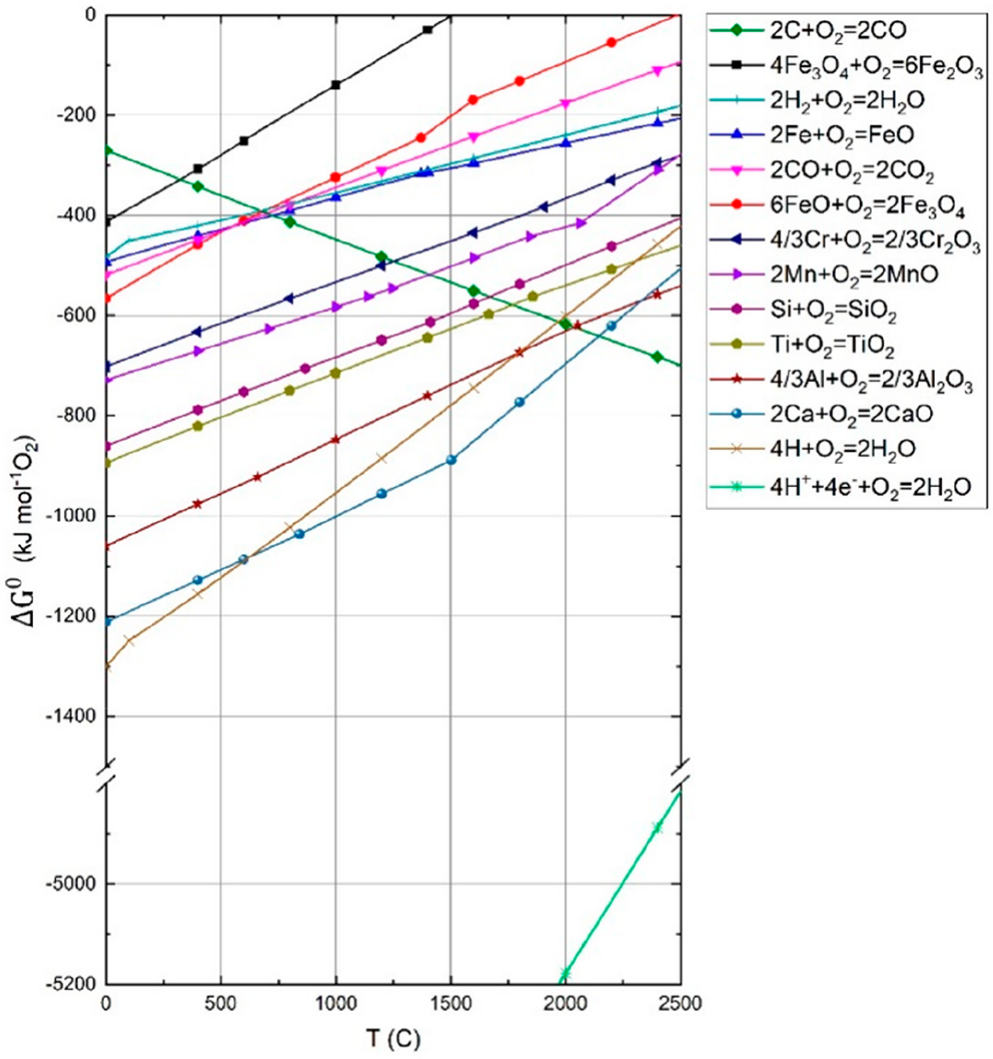
Ellingham Richardson diagram showing the Gibbs free energy for several metal oxides together with Gibbs free energy lines for hydrogen and the H^+^ radical,^143,145,254^ present (among many other ionized species) in a hydrogen-containing plasma.^255^ Other versions of Ellingham diagrams with several types of higher-order plasma-related hydrogen radicals have been published in refs (145, 237, 253, and 256−258) (see also Figure 59). The figure is reproduced with permission from ref (259) under an open access Creative Common CC BY license. Copyright 2022, MDPI.

Two important points must be considered when constructing such an Ellingham analysis for plasma metallurgical reduction reactions that involve reactive species that are in ionized state, Figure 68. First, the Boltzmann–Saha distribution function must be used to calculate for a given temperature and for the different ionization energies the respective equilibrium occupation numbers of the different ionized species (with their respective ionization energies). For instance, for hydrogen-containing plasmas the fractions of H_2_, H, H^+^, H_2_^+^, etc. must be calculated. Here, also the partial pressures of the other gas species (such as of the argon or nitrogen) must be considered. Second, all reactions according to the availability of these different reductive species must be taken into account so that for a plasma reduction process not only a single reaction must be calculated following the Ellingham analysis, but a set of reduction reactions pertaining to all the different species that are available in their respective fractions that have been calculated by the distribution function analysis must be accounted for. Another important aspect is that at the actual reaction interface between the hydrogen-containing plasma and the liquid (or respectively, solid) oxide, it is very likely that nonequilibrium states apply, owing to the evaporation of species from the (mostly mixed oxide-metal) melt (or solid) into the plasma atmosphere.

### 5.9 Thermodynamic Aspects of Primary versus Secondary Synthesis of Metals

**Figure 69:**
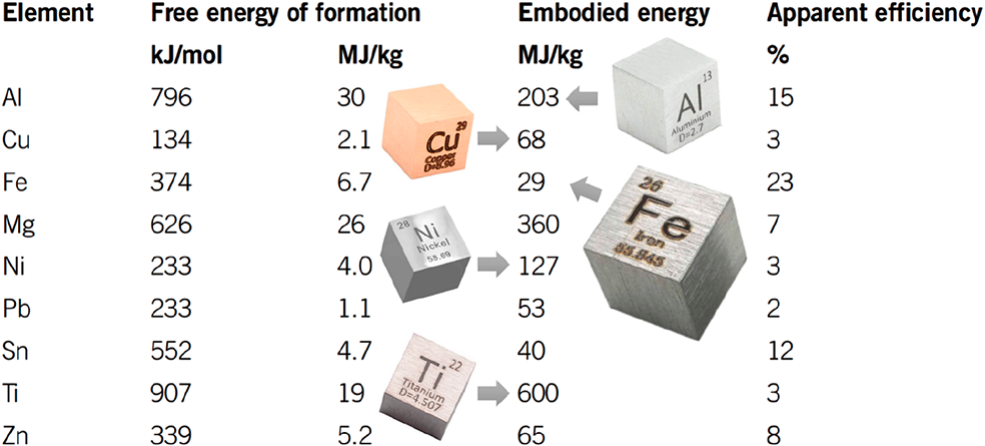
Extraction efficiency under real industrial boundary conditions versus the lower thermodynamic limit of the minimal necessary energy, using numbers taken from ref (162).

**Figure 70:**
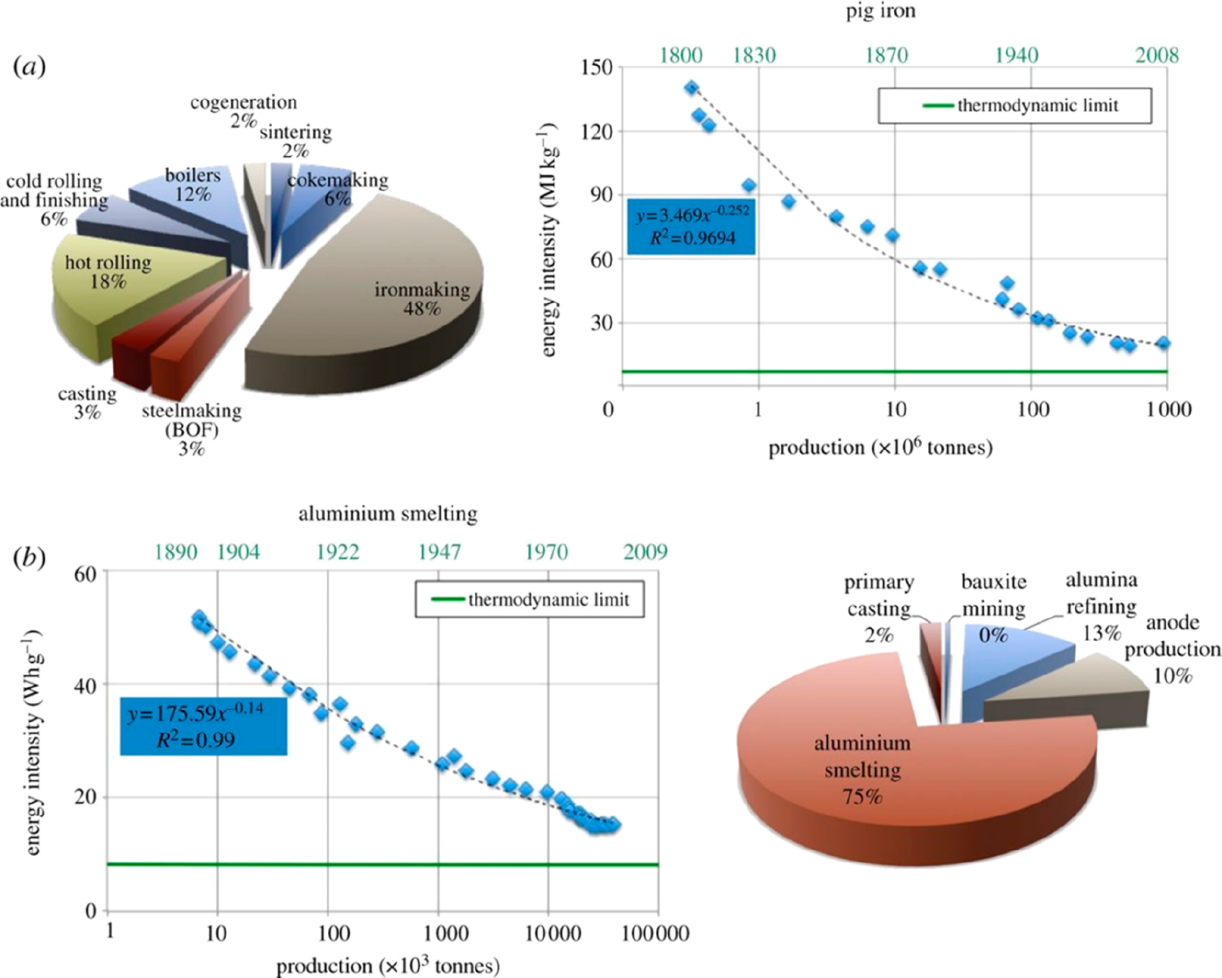
Different contributions to the embodied energy for the two main greenhouse gas emitters steel and aluminum.^17^ The figure also shows the historic evolution of the gradual decrease in the amount of energy needed for metal extraction, slowly approaching the thermodynamic limits of the underlying reduction processes for the case of pig iron production from hematite oxide by using coke as reductant (a) and for the case of aluminum production by using electricity in the molten salt electrolysis process (b). The figure is reproduced in modified form with permission from ref (17). Copyright 2013, The Royal Society.

Figure 69 illustrates the large difference between (a) the thermodynamically embodied energy, which would represent the minimum energy required to extract the metal from its ore in the case of a purely hypothetical 100% conversion efficiency, and (b) the energy actually consumed for the real production of metals. This comparison clearly shows that most metals have an integral extraction efficiency below 10%. However, the metals iron and aluminum, which are particularly important due to their huge production volumes, have a much higher production efficiency. Figure 70 shows for these two metals the composition of the energy fractions consumed for the individual extraction steps. It should be taken into account that, due to the use of fossil reduction agents and fuels required for heating and energy production, these efficiency trends and the total embodied energy qualify as an approximate measure for the carbon footprint of the different metals.

**Figure 71:**
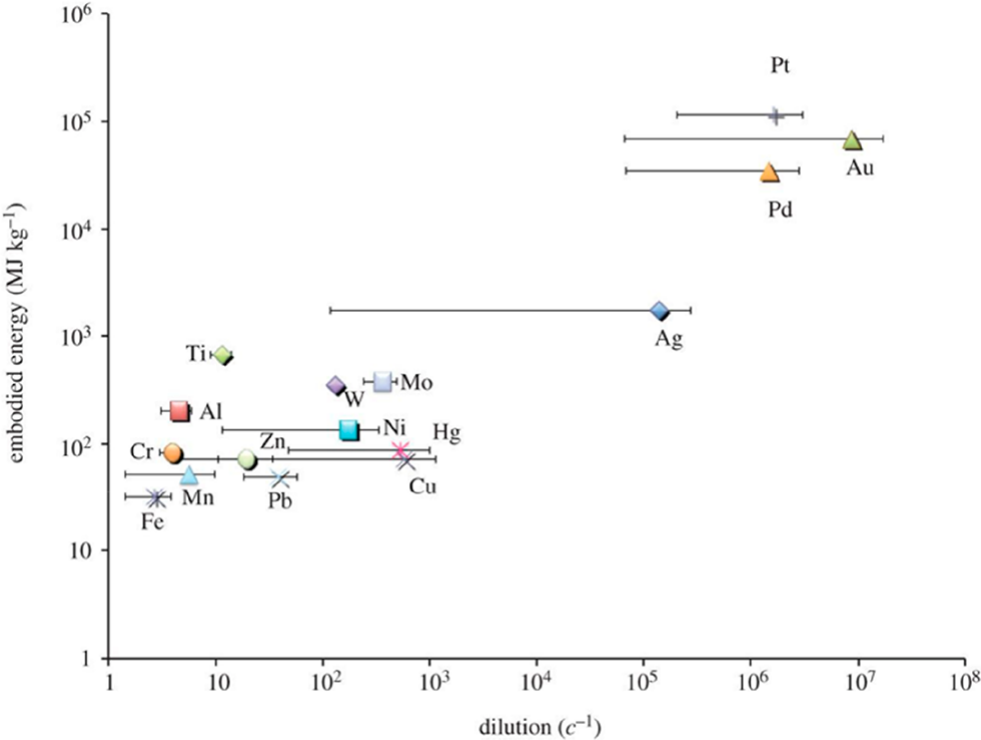
Relationship between dilution and embodied energy.^17^ Figure is reproduced in modified form with permission from ref (17). Copyright 2013, The Royal Society.

It is also a suited approach to approximate the real embodied energy of a metal though its dilution in the Earth’s crust. This translates to the energy costs associated with the higher efforts required for the accumulated mining, refining, and benefication steps to extract the metal from its ores, Figure 71.

Another energetically important aspect regarding the dilution of metals in their original ores is that during the successive metallurgical process steps required for their enrichment and extraction, large amounts of slag are formed. These slags consist of all these gangue minerals and oxide tramp elements which are undesirable and from which the actual metal has to be separated. The slag quantities get larger with higher dilution of the target metal in the mineral from which it originates. The large amounts of slag are heated during the corresponding mostly pyrometallurgical process steps, and this costs large amounts of wasted energy, which does not benefit the actual product.

It could therefore become attractive in the future to think about metallurgical extraction methods in which a whole series of accompanying and gangue elements are extracted and synthesized at the same time, i.e. the co-extraction of elements that have so far been mostly regarded as waste products. This is already today being done for several expensive metals, such as the joint extraction of nickel and cobalt or tantalum and niobium etc., but for some mass-produced metals, such as aluminum, which is extracted from its oxide previously enriched from the bauxite mineral, it is not yet sufficiently commercially attractive. Another target for such research could be silicon-rich banded iron ores.

This means that for highly dilute metals, an interesting new research direction could be the simultaneous sustainable extraction of different elements from complex and commercially inferior minerals. A commercial attractiveness here could be based on the fact that many of these mixed minerals are (a) not attractive for the classical synthesis routes and thus are much cheaper to obtain on the market and (b) may contain minor fractions of rare and precious metals, the extraction of which might render such processes affordable.

As an example form a thermodynamics perspective, the minimum energy for the reduction of hematite to pure iron is 6.1 MJ/kg. This is the free energy of oxidation of iron, thus representing the minimum thermodynamically embodied or stored energy. This is also the thermodynamic limit of the energy (assuming near 100% efficiency) that could be theoretically gained from its combustion or from its use as a metal-air battery.

The actual average used energy to make iron, i.e. the actually embodied energy, however, is no less than 18–25 MJ/kg; i.e., current processes have in average a conversion efficiency of 22–33% and the rest is waste heat.

In these overview diagrams, the energy actually required for the extraction of the different metals is made up of the relative fractions of the different synthesis methods (primary and secondary) according to the current market situation. It is interesting to note in this context that especially the two large metal groups, i.e. steel and aluminum, are currently only manufactured about one-third from scrap and two-thirds are obtained from ores. However, as described above, this trend will be reversed in the coming decades and much higher quantities of scrap will be used in the future.

**Figure 72:**
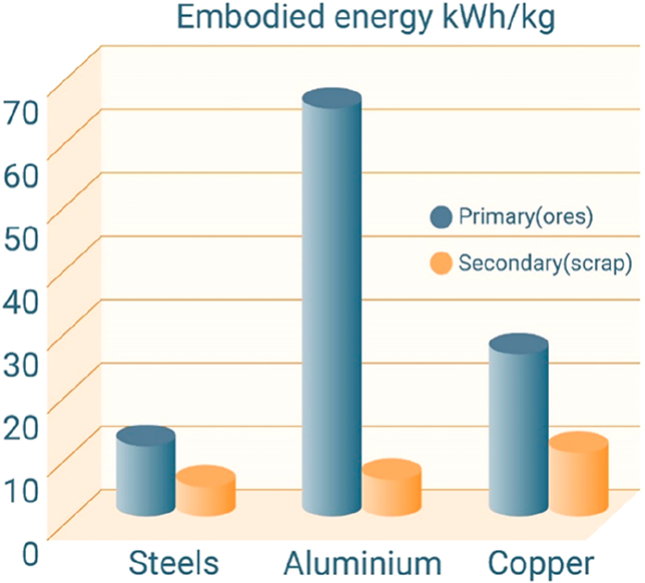
Difference in energy demand for primary and secondary syntheses. The discrepancy among the different metals and specific alloys is in part due to the corresponding differences in melting points, heat capacity (determining the energy required for smelting scrap), processing pathways and the embodied energy (of which the thermodynamically embodied energy is only a certain portion, i.e. between 25 and 75%).^9^ Magnesium and aluminum have by far the largest difference between primary (reduced from minerals) and secondary (melted from scrap) production, due to their (i) high bonding energy to oxygen (making primary reduction very energy intensive, see also data in Figure 58 and Figure 68) and (ii) low melting points (Al, 660 °C; Mg, 650 °C; i.e. resulting in a fairly low energy required for melting).

For this reason, it is particularly interesting to look at the energy differences between primary and secondary syntheses in the context of sustainable metallurgy. It becomes clear from Figure 72 that especially for metals with a low melting point on the one hand and a comparatively high absolute value of the Gibbs free energy of its oxide on the other hand (see Figure 58 and Figure 68), secondary synthesis of the material from scrap can save enormous amounts of energy, in the case of aluminum up to 95%.

## 6 Feedstock for Sustainable Metal Production: Minerals, Metals, Liquids, Plasma, and Gas

### 6.1 Principal Differences in Feedstock with Respect to Sustainable Metallurgy

**Figure 73:**
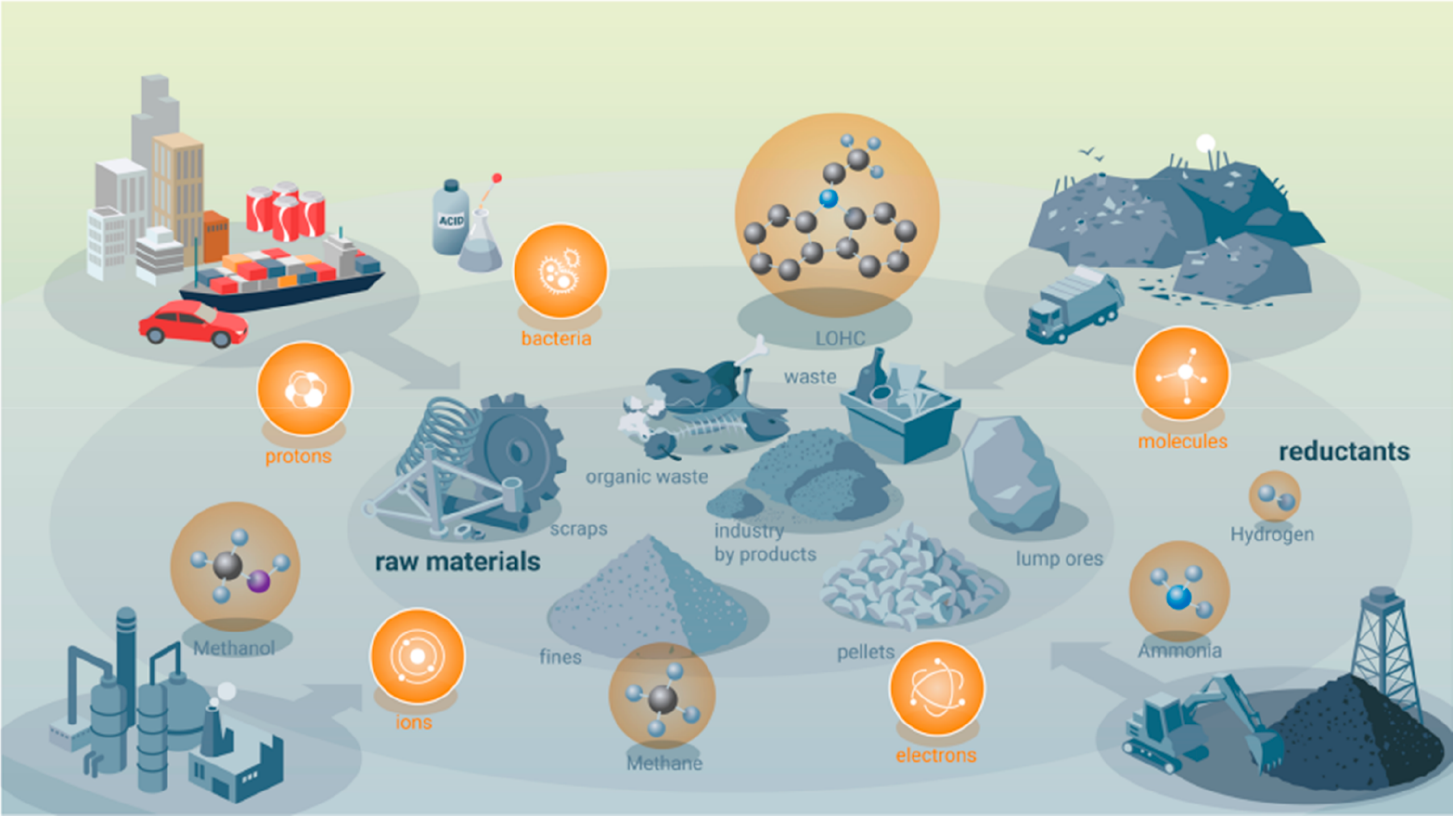
Different possible combinations of metal feedstock (scrap and minerals of different grade and dispersion) and reduction pathways in different aggregate states. The overview shows some of the currently emerging opportunities for basic research for the case of iron making, considering the origin of the feedstock, coming for instance from primary resources (mining), secondary resources (scrap), or tertiary resources (re-mined material, dumped waste). The latter resources can be both of mineral origin (e.g., red mud, as metal carrier) or of biological origin (biomass, as reductant).

Sustainable metallurgy has to take into account a large variety of raw materials of both mineral and metallic nature, and different types of reducing agents and their mixtures. These feedstock groups do not represent homogeneous material classes in themselves and thus offer feedstock-specific basic research opportunities in the context of sustainable metallurgical production, Figure 20. Figure 73 provides a more specific overview of some of these aspects for the case of steel, with similarities for related transition metals such as nickel and manganese.

First we consider the feedstock needs for the linear parts of the metallurgical economy, namely, the underlying ores, Figure 12. These minerals that are used for the metallurgical synthesis sector, occupy, due to the huge quantities of currently about 3.2 billion tonnes of mineral ores mined per year, a particularly important role in connection with the sustainability of metal production, Figure 1.

Five aspects are particularly important to distinguish: (a) dilution of the metal in the Earth’s crust;^17,162^ (b) dispersion, porosity, and granularity;^43,85,260^ (c) chemical impurity content, also referred to as gangue;^56,110^ (d) degree of dilution or respectively richness of the sought-after metals in the ores, i.e. minerals of different degree of quality and purity;^21^ and (e) thermal, mechanical, and chemical processing methods required for mineral benefication before the actual metallurgical use, such as roasting, sintering, or pelletization,^261^Figure 71.

These aspects are of general importance in the selection of raw materials for conventional metallurgical processes, but they deserve especially close scrutiny in connection with sustainability, since particularly the exploitation of hitherto less profitable raw materials or the avoidance of benefication can be of interest as alternative feedstock options when new sustainable metallurgy processes are developed. The motivation to revisit these questions is that the established fossil-based metallurgical reduction processes such as blast furnaces or direct reduction furnaces require highly optimized mineral states, in terms of both richness, impurity content, mechanical strength, thermal stability, etc. However, when many of the currently used furnaces and reduction gases have to be replaced anyway by new reduction methods, workflows, and smelting technologies during the next two decades, it can be of potential interest to consider also alternative (e.g., less costly, less pure, etc.) feedstock to operate them.

**Figure 74:**
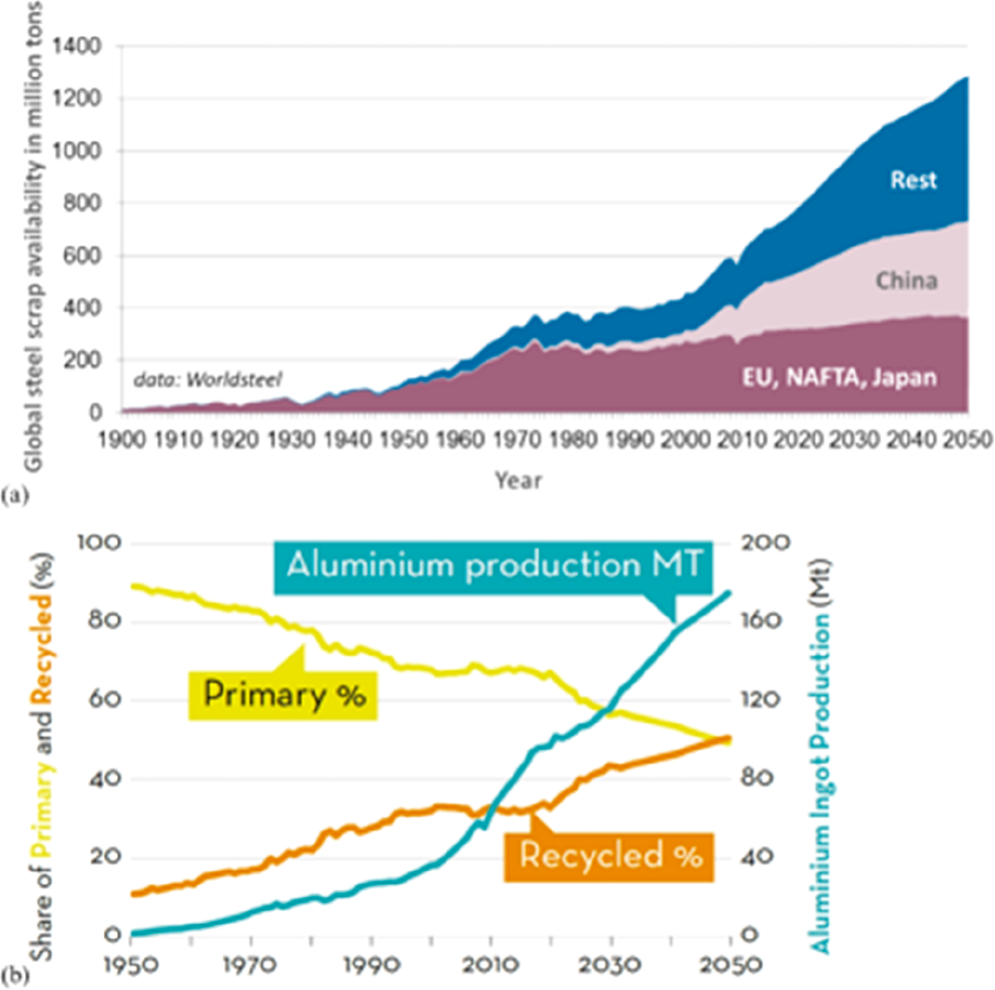
Estimated growth of the scrap markets for the dominant load-bearing alloy groups responsible for the highest CO_2_ emissions, namely, (a) steel^137^ and (b) aluminum alloys.^7^ The data have been taken from the International Steel Institute and the International Aluminum Institute, respectively. MT, million tonnes. The figure is reproduced in modified form with permission from (a) ref (137) (Copyright 2019, Elsevier) and (b) ref (7) (Copyright 2022, Elsevier).

**Figure 75:**
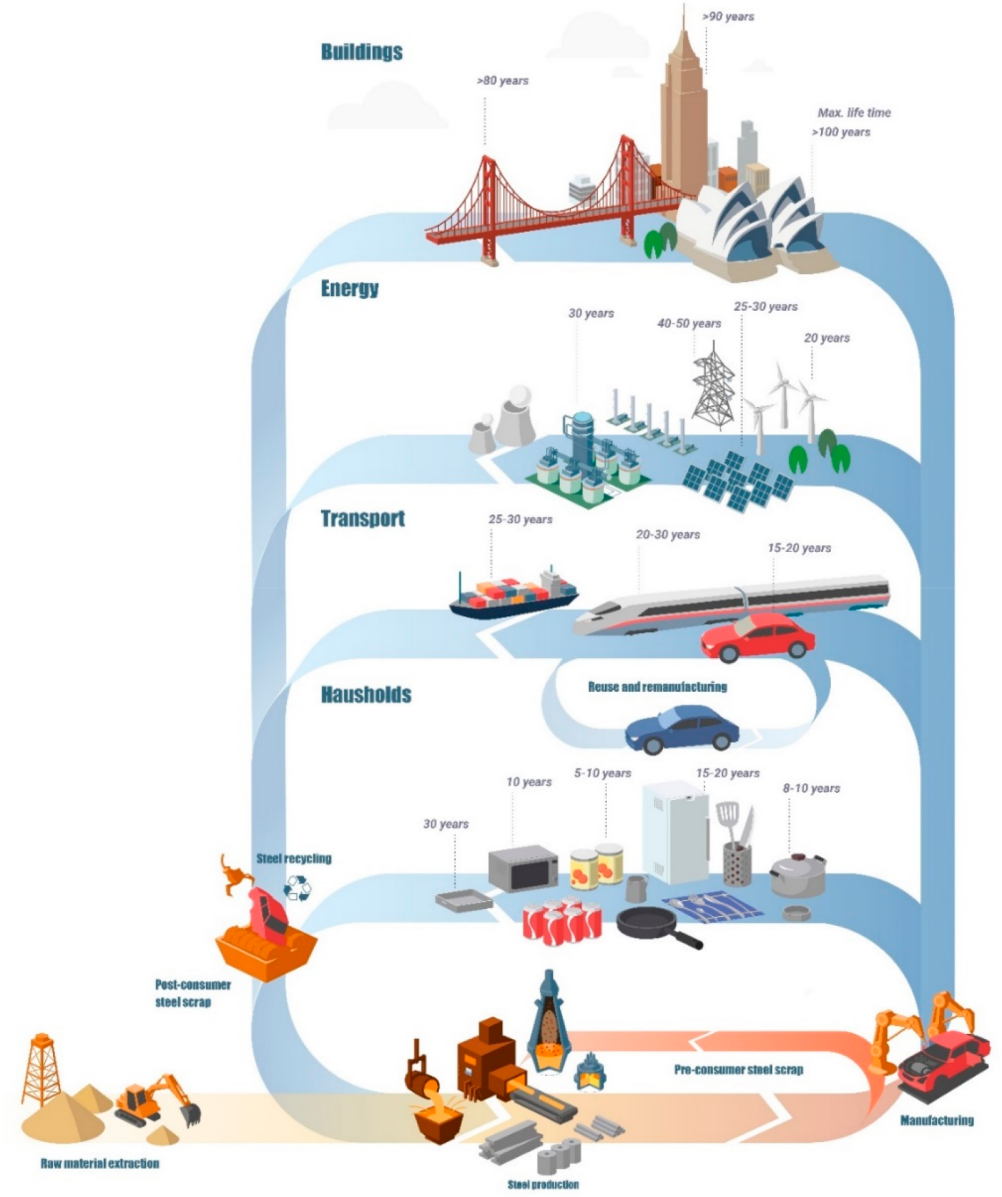
Relationship between scrap markets and longevity of metallurgical products, using steel as an example. Particularly the huge growth of the markets in Asia during the past decades will lead to a staggered sequence of likewise huge scrap streams that enter the scrap markets, serving as new feedstock for sustainable steel making. A peak in scrap return is expected around 2060. With these scrap streams it might be possible by 2050–2060 to reach a global average of up to 2/3^rd^ of the mass-produced metals being made from scrap.

**Figure 76:**
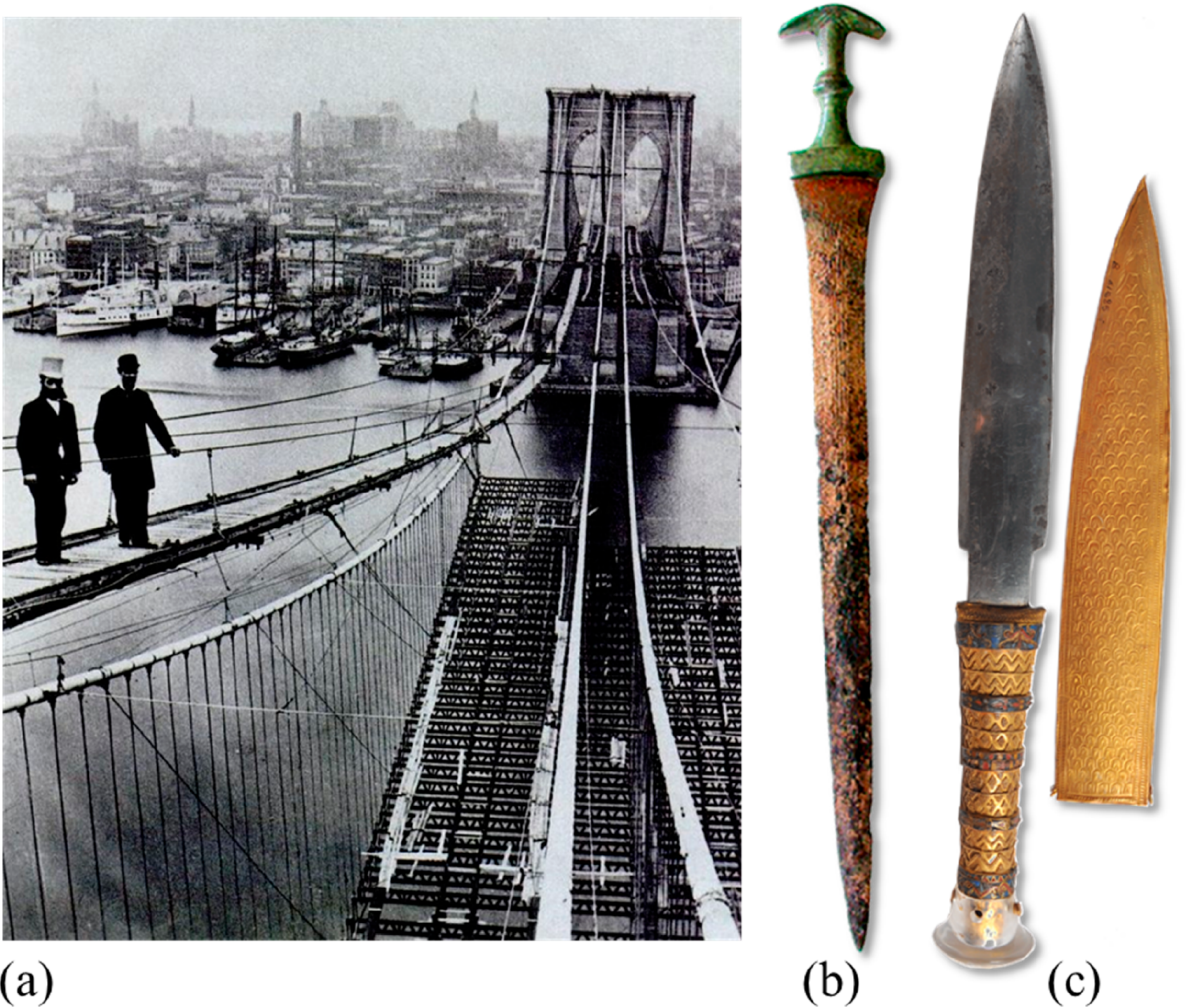
A few (extreme) examples of long-lasting iron products: (a) Brooklyn Bridge, built 1869–1883; (b) Hittite sword from the early iron age, about 1200 BC; (c) iron dagger of Pharaoh Tutankhamun, 1330 BC, manufactured not from reducing ores (because the Egyptian empire was in the bronze age at this time) but from an iron-nickel meteorite via forging. These examples show that time must be considered as an essential factor when estimating scrap return quantities.

While these points apply to the sector of primary synthesis, another aspect comes from secondary production, where new alloys are made from scrap: The use of scrap instead of ores will particularly dominate the future mass production of the major structural alloy groups for load-bearing applications, such as iron, aluminum, and nickel, fuelling the rapidly growing circular portion of the metallurgical economy, Figure 74 and Table 16. The delay in feeding large quantities of metal as scrap back into a circular economy has to do with the high longevity of many metallurgical products, particularly in buildings, ships and vehicles. However, in the wake of the huge industrial growth period of the Asian markets, the next decades will bring huge amounts of scrap back into the material feedstock stream, Figure 75 and Figure 76.

**Table 16:** Scrap-, Energy- and CO_2_-Related Numbers on the Main Structural Alloy Classes^a^

	Global annual production	Synthesis based on scrap input	Energy consumed (EJ/yr)	CO_2_ emissions	Material scrapped in manufacturing
**Steel**	1850 million tonnes per year	45%	40	3.7 Gt CO_2_eq per year	25%
**Al**	92 million tonnes per year	30%	13	0.7 Gt CO_2_eq per year	40%
**Ni**	2.1 million tonnes per year	25%	0.25	26 Mt CO_2_eq per year	20%

a Data taken with permission from a paper of Raabe et al.^2^ showing data from the year 2019.

In that context it must be taken into account that scrap types can be very different in chemical composition and dispersion. Figure 20 shows bulk and compositionally homogeneous mass scrap (i.e., collected in-production); less well sorted, mixed, contaminated, and post-consumer scrap (so-called old scrap); or scrap from electronic and electrical nanointegrated products from which—for a circular economy—often more than 30−50 elements would have to be won in a near 100% recycling scenario. A particularly challenging case in that context is the recycling of multiple metals from integrated circuits and battery components (so-called nanoscrap).

This means that the designs of corresponding metallurgical processes do not only have to take into account the amount, origin, sorting, transport, and purity of the corresponding scrap, but they must also consider processes in which, for example, mineral raw materials can be processed together with scrap, possibly even in the same aggregate.^135,167,262−264^ Another interesting research strand is to develop impurity-tolerant alloy variants that can be made from the largest possible fractions of scrap.^2,7,265−267^ A related consideration is not only that scrap is used to make new alloys but also that these new materials become scrap themselves when they reach the end of their product life. They must then again enter the (secondary) synthesis cycle. This brings up the question whether it might make sense to reduce the number of available alloy variants, in order to reach higher elemental homogeneity of the scrap streams. All these quite different research directions will be illuminated in more detail in the next sections.

**Figure 77:**
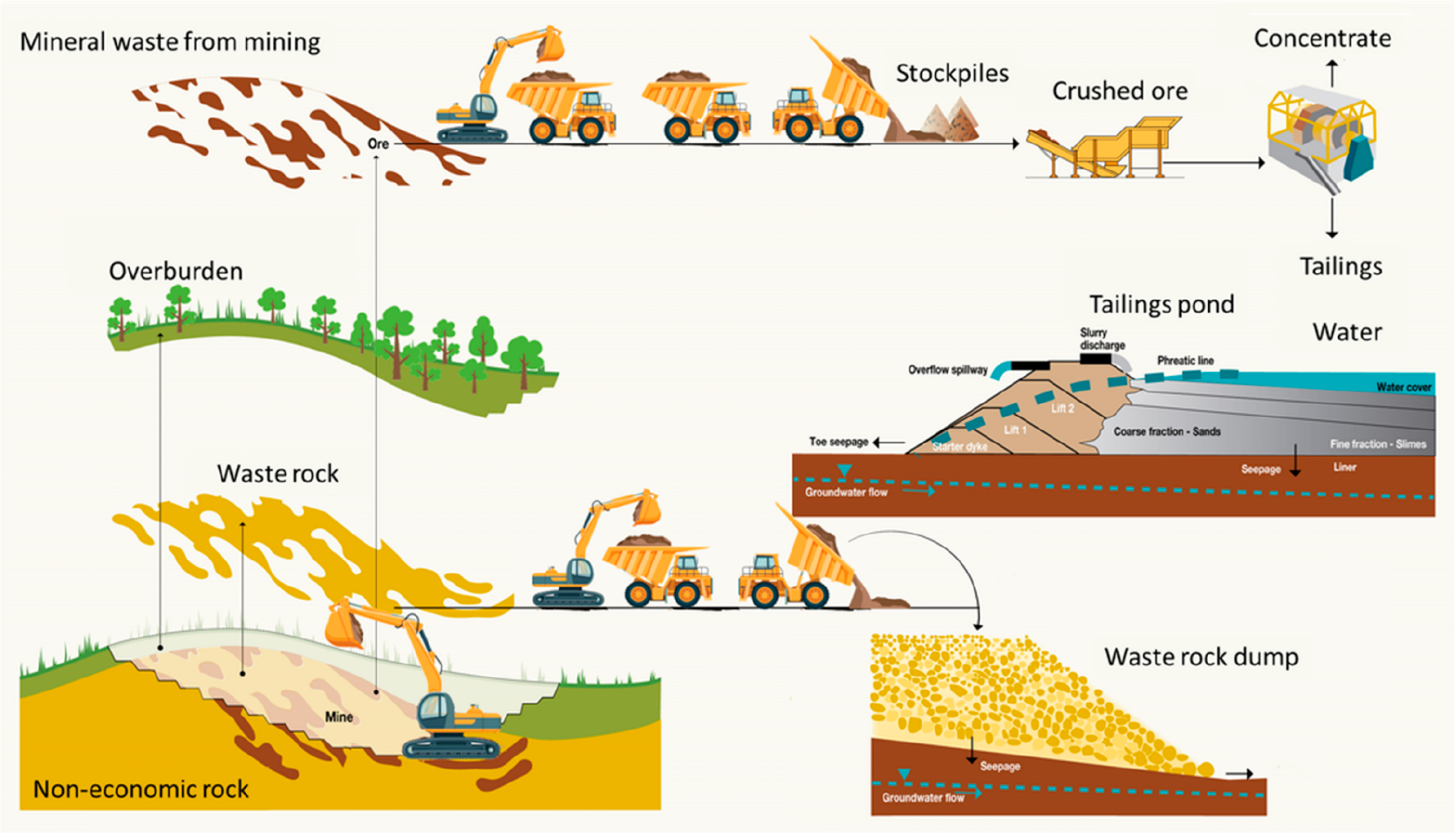
Challenges associated with metal-containing mineral mining and valorisation of mine waste and by-products. Important future opportunities for research lie in the re-mining of dumped waste, low-quality ores, and mineral-processing residues as well as in the pertinent usage of tailings.

The tertiary feedstock basis to sustainable metallurgy consists in using old and dumped waste and landfill material; i.e., it aims at bringing lost and often hazardous material back into the material value stream. This type of raw material can be termed “urban feedstock”, and the process to recover valuable material from it has been sometimes termed “re-mining”.^60^ Feeding dumped waste back into the synthesis stream is the only approach that actually allows us to decouple the strong growth in the demand for metallurgical products from the markets for minerals and scrap, Figure 77.

It also creates a substantial commercial incentive since dumping such industry waste materials becomes in many regions an increasingly costly factor for companies, which means that sustainable metallurgical synthesis technologies can commercially better compete with existing fossil-based metallurgical processing pathways and business models which rely on dumping many of their residues.

**Figure 78:**
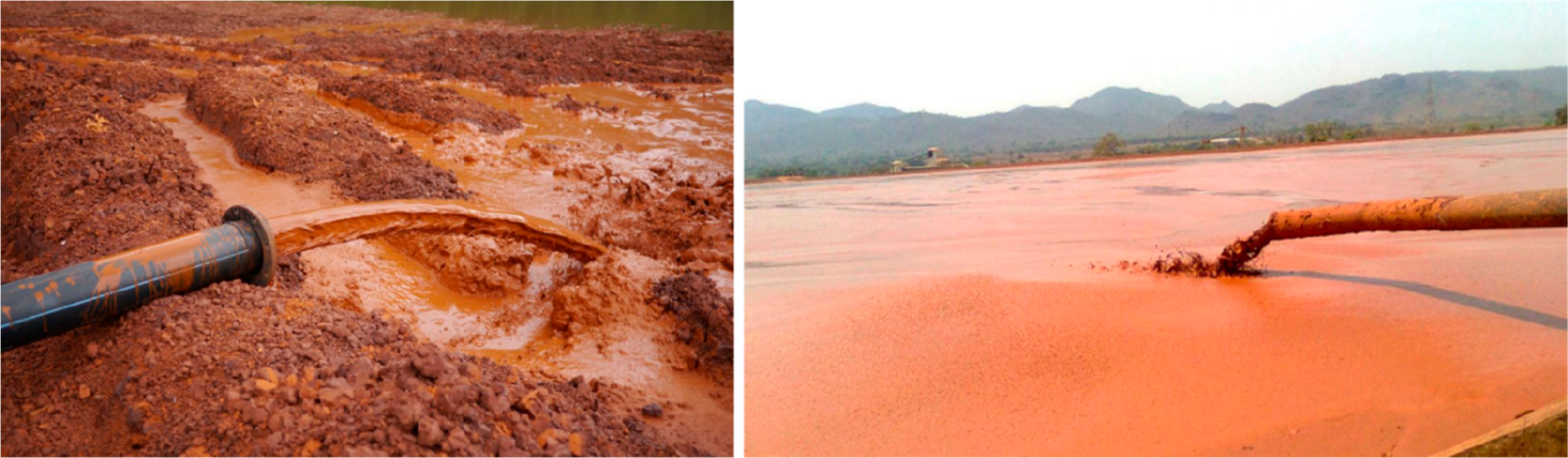
View at typical red mud dump sites. Red mud is a bauxite residue, rich in iron oxide and titanium oxide as well as in a few rare earth metals. Bauxite serves as primary feedstock mineral for producing the alumina which is then used subsequently in fused-salt electrolysis to produce metallic aluminum. The red color comes from the high proportion of iron oxide compounds in the bauxite. In the context of developing re-mining opportunities, such dumped residues can become potentially commercially viable feedstock sources for future sustainable metallurgical production (see sections 6.4.2–6.4.5).

For many of these deposited industrial waste materials it is often quite surprising and insightful to learn how huge their volume actually is. An example is the so-called red mud, a residue material from aluminum production, which will be discussed in more detail below, Figure 78 (see for instance sections 6.4.2–6.4.5).

In this context considerations of other waste products from industry, agriculture, and the consumer sector also play a role, for example the use of sustainable and renewable carbon carriers. This essentially refers to organic waste that can be used as carbon and hydrogen carriers, serving for instance as reductants in metal oxide reduction processes. In such considerations, carbon dioxide continues to be released into the environment as a product of the underlying redox reactions, but it is also reabsorbed by plant material, so that a carbon cycle is created. Although this approach appears plausible at first glance, it must yet be seen critically because it would not lead to the required rapid reduction of net carbon dioxide emissions. Also, the large-scale industrial use of biomass would trigger markets to serve the metallurgical sector that would compete with crop production, a side effect that must be avoided. The approach also underestimates the huge quantities that would be needed by the metal sector to create such a bio-based carbon cycle.

Another interesting variant for sustainable metallurgy comes from biology.^268,269^ Here, three main categories are of interest, which open up new research possibilities for a more sustainable metal economy. These include the use of (a) bacteria and fungi, for example in the field of hydro- and electrometallurgy for metal enrichment, as well as (b) plants that can enrich certain metals during growth (also called hyper- or superaccumulating plants) and finally the use of (c) organic waste (biomass), for example from agriculture, as renewable reducing agents and carbon suppliers (see section 6.5).

**Figure 79:**
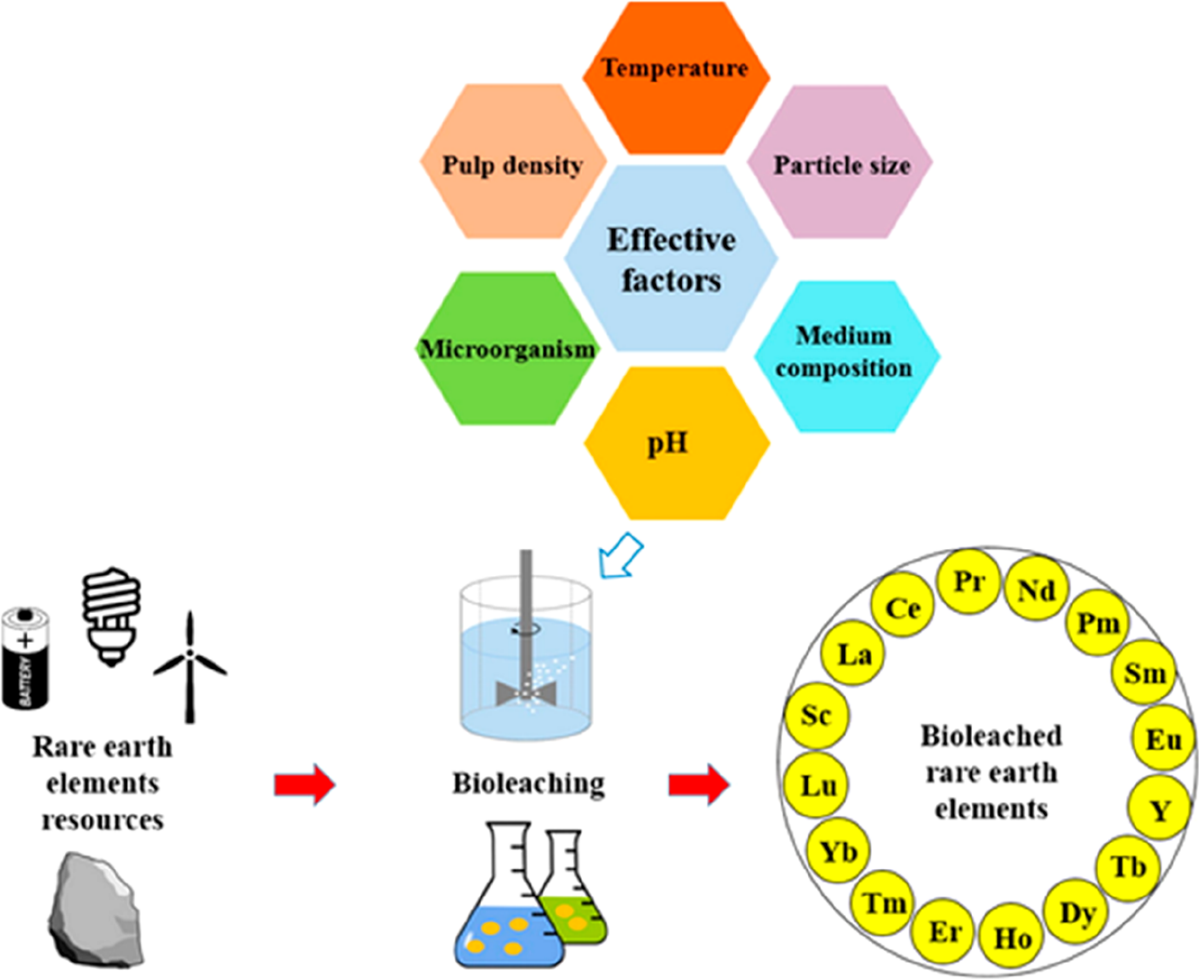
Example workflow and parameter field for recovering rare earth elements from mixed waste and mineral feedstock as an example where biometallurgical methods are gaining momentum, using a bio-leaching process based on microorganisms. The figure is reproduced with permission from ref (274). Copyright 2021, Taylor & Francis.

Important target metals to be retrieved by the use of bio-hydrometallurgical methods are particularly elements that occur in highly dilute form in mixed electrical, electronic, battery, and magnetic scrap, such as gold, copper and rare earth elements, Figure 79(270−273) (see details in section 7.8).

**Figure 80:**
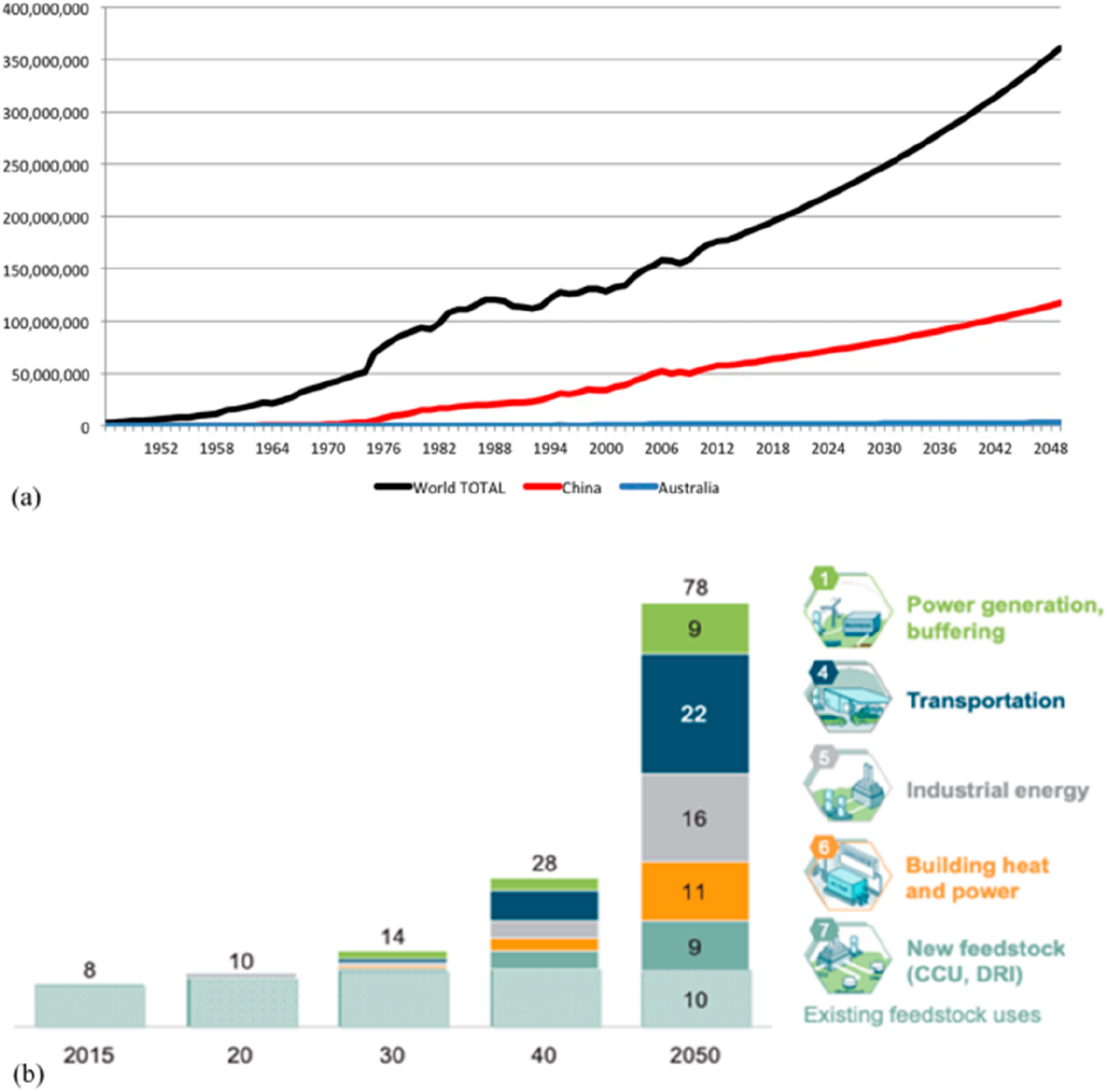
(a) Development and forecast for the global market demand for ammonia (NH_3_) in metric tonnes according to numbers of the US geological survey. (b) Forecast for the global energy supply delivered by hydrogen as energy carrier and areas of projected consumption in units of Exa-Joule (EJ). An interesting aspect to consider in this context is the possibility that the “green” hydrogen produced from renewable energy sources, due to its initially very high price, will be affordable primarily for fuel cell applications in the transport sector rather than for use as a reducing agent in the metallurgical sector. On the other hand, it should be noted that the hydrogen used for fuel cells must have very high purity, whereas the hydrogen that could be used as a reducing agent in the metal industry only needs to have very low purity. This could mean a decisive price advantage for the attractiveness of hydrogen as a sustainable reductant.

Another important research aspect with regard to the raw materials required for sustainable metallurgy is the next generation of (sustainable) reducing agents. The future of metallurgical synthesis can no longer be resting on coal and related carbon vectors as reductant feedstock, but must turn toward either using electrons directly (see section 7.7) or using sustainably produced gaseous hydrogen vectors (green hydrogen, ammonia, methane, methanol, etc.) as fossil-free reductants (see section 7.4.6). This topic represents possibly one of the biggest changes in the field of metallurgy during the next decades, because it means to replace all fossil, i.e. carbon-containing, compounds by hydrogen-bearing compounds or by the direct use of sustainable electricity in all reduction processes. The same applies for all downstream processes (subsequent to synthesis) for heat generation in the metallurgical process chain (for melting scrap as well as for all heat treatments and heat-holding stations). This raises some very fundamental new questions, such as how certain hydrogen-containing compounds can be efficiently used as reducing agents and what the underlying specific redox reactions are and what effect these compounds or their reaction products have on the final alloy and product. Important examples for future research directions in that context are the direct reduction of iron oxides by using green hydrogen and green ammonia, Figure 80 (see section 7.4.6).^240,241,275−278^ Also, it seems worth studying how different reductants would interact with each other and possibly influence the reduction kinetics and also the compounds that are created when jointly used in the same reactor (some reductants would most likely not only produce pure metal during the reduction but also other compounds).^279,280^

Table 17 lists some topics and opportunities for basic research related to suited feedstock materials in sustainable metallurgy. Most of the specific topics suggested in this table will be discussed in more detail below in separate sections.

**Table 17:** Opportunities for Basic Research Related to Suited Feedstock Materials as a Basis for Sustainable Metallurgy

Mineral and solid reductant feedstock: Use of less pure minerals; use of banded ores; high Si-containing minerals; pelletized versus fine versus lump ore use (all associated with different mining, tailings, and greenhouse gas footprint); minerals that cause less environmental harm, waste, water use, and tailings when mined; reduced mineral processing (such as sintering) prior to use in reduction processes; moderate use of renewable biomass as reductant (avoiding competition with food production); use of feedstock with reduced beneficiation requirements
Reductant feedstock: Replacement of fossil reductants by sustainably produced hydrogen carriers; use of mixed reductants; reductants produced from power-to-fuel and power-to-reductant processes (such as sustainably produced hydrogen, methane, methanol, ammonia, etc.)
Mixed feedstock: Mixing feedstock of different (cost-efficient and sustainable) origin in reduction and smelting operations; design of reactor concepts that can cope with flexible reductant (and mineral) charging
Bio-hydrometallurgical feedstock: Plant, fungi, and bacteria as biological feedstock in precious metal, rare earth, nickel, and cobalt recovery via bio-leaching

### 6.2 Primary Mineral Feedstock Types and the Role for Sustainable Production

#### 6.2.1 Introduction to Mineral Feedstock

Nearly all metals are in oxidized state when mined (except gold, silver, platinum, or copper nuggets, which are sometimes found in reduced state, yet only in tiny quantities). Most of the minerals mined today (and also waste feedstock materials that are being re-mined^281,282^) are mixtures of several types of oxides, sulfides, and sometimes also carbonates. The task of extractive metallurgy (see next sections) is to recover one or more specific target metal(s) from these mineral mixtures. In this context the richness or respectively the dilution state of the metal in the mined mineral is of highest relevance, both for deciding (a) if it is commercially viable and sustainable to extract the metal from it and (b) which extraction method(s) might be most suited. These aspects are essential because the sustainability of metal extraction from ores usually increases with the richness of the mineral and it becomes vice versa very poor when the metal fraction inside of the mineral becomes very low, Figure 70 and Figure 71. This is an important aspect which is sometimes overlooked because it is not the integral abundance of a certain metal (somewhere) in the Earth’s crust that matters for sustainable metallurgical extraction but only its aggregation state, i.e. its local accumulation. It is particularly those metals that occur in highly dilute state which are the ones with the highest energy and carbon footprint when extracted from their respective minerals, Figure 31. This implies that research has a particularly effective leverage in those areas where the extraction and production of important metals from highly dilute ores have the highest energy and carbon footprint. Examples are for instance nickel, cobalt, aluminum, titanium, rare earth metals, copper, and iron (the latter for its huge overall quantity produced).

Most metals occur in different oxidation stages, mostly bound in the form of oxides (bound to oxygen) and sulfides (bound to sulfur). Some others can also form silicates (bound to silicon), halides (bound to fluoride or chlorium), and carbonates (bound to CO_3_). Examples for commonly used oxide ores in metallurgy are hematite (Fe_2_O_3_), magnetide (Fe_3_O_4_), chromite (FeO·Cr_2_O_3_), cuprite (Cu_2_O), alumina (Al_2_O_3_·H_2_O), rutile (TiO_2_), pyrolusite (MnO_2_), cassiterite (SnO_2_), or wolframite (Fe(Mn)WO_4_). Typical examples for commercially used sulfides are chalcopyrite (CuFeS_2_), chalcocite (Cu_2_S), molybdenite (MoS_2_), sphalerite (ZnS), pyrite (FeS_2_), cinnabar (HgS), galena (PbS), millerite (NiS), pentlandite [(NiFe)_9_S_8_], and stibnite (Sb_2_S_3_). Common silicates in the metallurgical sector are zircon [Zr(Hf)SiO_4_] or titanates such as ilmenite (FeOTiO_2_). Carbonates of commercial relevance are for example magnesite (MgCO_3_), azurite [2CuCO_3_Cu(OH)_2_], dolomite (MgCO_3_CaCO_3_), witherite (BaCO_3_), and malachite [CuCO_3_Cu(OH)_2_]. Halides of relevance are CaF_2_ (fluorite) and NaCl (halite).

**Figure 81:**
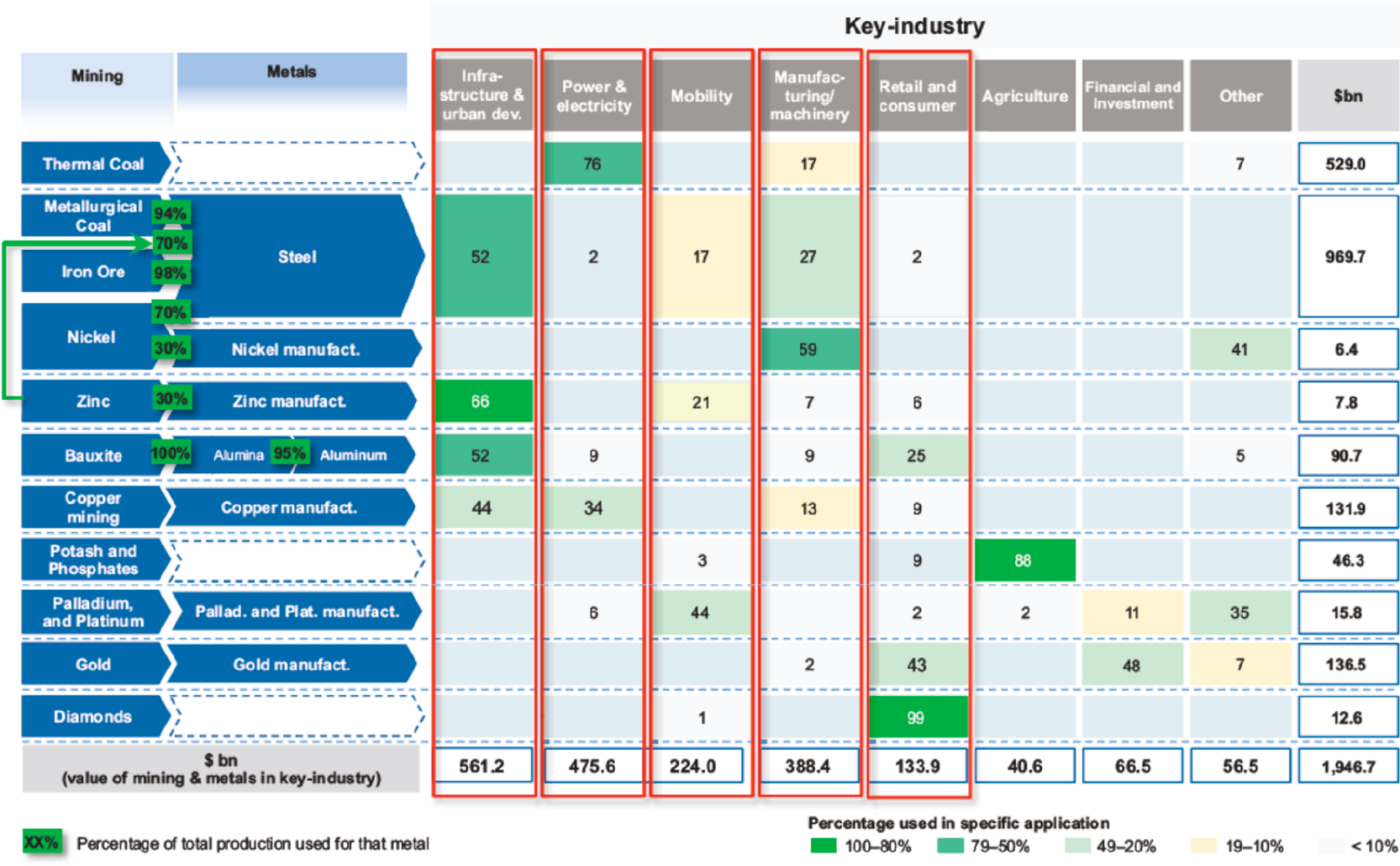
Overview of the main feedstock minerals and material classes and their respective percentages used in specific application fields in key industry sectors. The flow data are shown in units of billion US dollars.^44,45^

The fact that most of these oxides, sulfides, etc. make up only a minor fraction in the naturally occurring ores and minerals, sometimes below 1 wt %, means that the majority of the mined material is an undesired by-product, referred to as gangue. Examples for typical gangue minerals are silica and alumina. Therefore, the first step that precedes the extraction of metals from these ores is the removal of gangue from the ore containing the metal. For this purpose, several mineral beneficiation steps are used. Examples are incorporating comminution, preliminary thermal treatment, and concentration by magnetic separation, heavy media separation, and flotation, Figure 81.

#### 6.2.2 Introduction to Iron Ores and Their Role for Sustainability

Iron and aluminum production are by far the largest consumers of natural ores, with iron ores alone standing for 3 billion tonnes of minerals mined per year, with a growing trend, Figure 1, Figure 6, and Figure 44. Common iron-carrying minerals are magnetite (Fe_3_O_4_, 72.4% Fe), hematite (Fe_2_O_3_, 69.9% Fe), goethite (FeO(OH), 62.9% Fe), limonite (FeO(OH)·*n*(H_2_O), 55% Fe), and siderite (FeCO_3_, 48.2% Fe). The numbers in brackets indicate the maximum stoichiometric metal content, but the real content is usually much smaller in natural deposits, because these ores are mixed with secondary minerals. For ion ores this is particularly the case for the so-called banded ores, where iron oxides are mixed with silicon oxides. Limonite occurs mostly as a mixture of several types of iron-based oxy-hydroxides such as goethite (α-FeOOH), akaganeite (β-FeOOH) and lepidocrocite (γ-FeOOH).

Among all these minerals only hematite and magnetite are commercially used for iron making, serving mainly in coke operated blast furnace iron production and—to a much smaller market volume—in methane-operated direct reduction furnaces. Mostly they are charged as beneficated pelletized products, sintered for specific ranges in shape, size, porosity and high-temperature mechanical properties for carrying the required loads in these huge aggregates.

**Figure 82:**
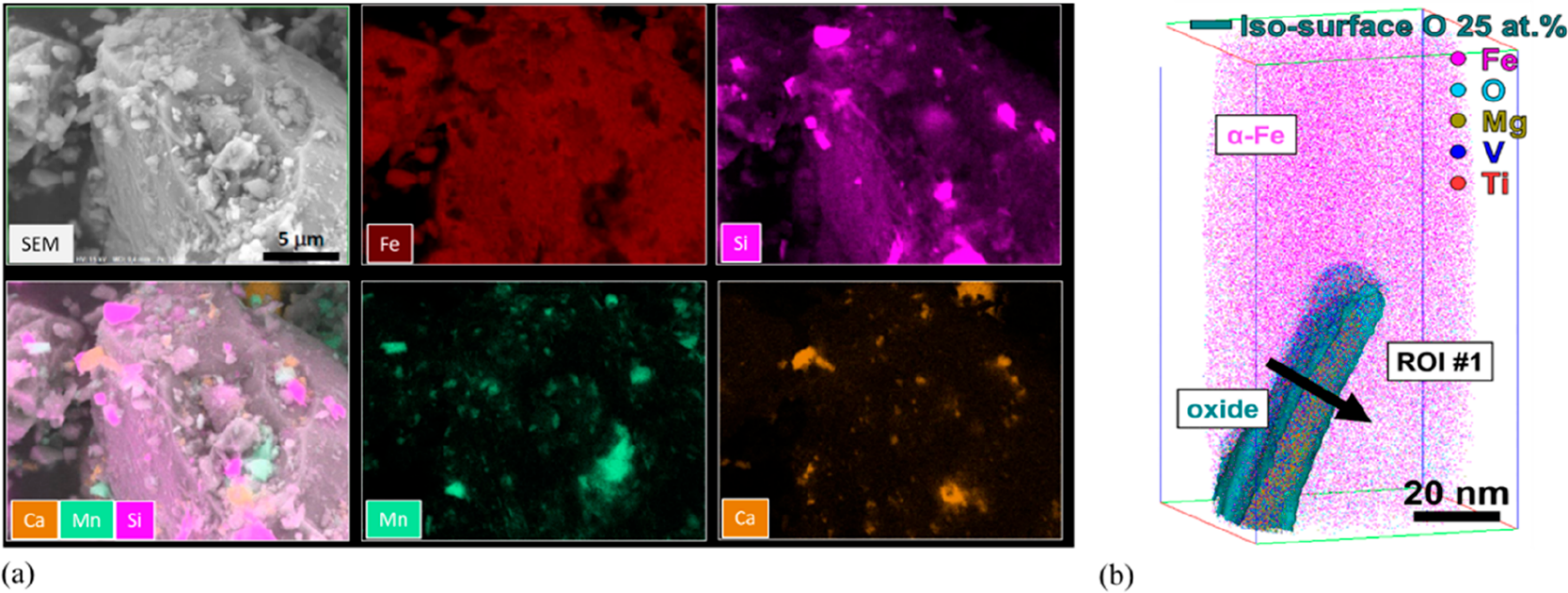
Example of gangue elements in hematite ores: measurement of retained impurity content after reduction in a pure hydrogen atmosphere at 700 °C.^241^ (a) SEM-EDX analysis of gangue element content and distribution. (b) Atom probe tomography of nano-oxides containing gangue elements such as Mg, V, and Ti, which tend to form stable oxides. This result shows that the impurity content of iron ores can play a role for the kinetics and thermodynamics of the hydrogen-based direct reduction of such minerals and must be taken into account in the design of the reactors, in the composition of the reducing agent mixtures, in the determination of partial pressures and reaction temperatures, and in the selection of the ores. The figure is reproduced in modified form with permission from ref (241). Copyright 2021, Elsevier.

The iron ores hematite and magnetite, which are mainly used in iron production via the blast furnace route, are both expensive and limited in quantity in the medium term, especially those ores with low gangue, viz. low impurity contents, Figure 82. For this reason, a future sustainable metallurgy will also have to answer the question of how lower-grade and less-iron-rich ores can be reduced to iron. A particular role in this is played by some of the reduction processes currently under consideration, which appear to be more suited for the processing of such low-grade ores than the traditional blast furnace route, for which very tight quality specifications apply.

This makes the issue of future ore selection in the context of sustainable metallurgy interesting, particularly from two perspectives: (1) ore availability and (2) process and product robustness to impurities. The latter aspects matters in particular, i.e. all research efforts that consider the issue of tolerable impurities both in the raw materials but also in the final produced metals and alloys, are of particular importance for all questions of improved sustainability in the metallurgical sector. The reason for this is that the elimination of impurity elements is associated with high additional energy costs on the one hand, but on the other hand is by no means of equal importance for all final product properties. This means that the question of the presence, the tolerability, as well as the inheritance of ore-related impurities between successive process steps is important in the context of the sustainability of the final metal products. This motivates us to introduce the term of the “science of dirty ores”. Some aspects related to metallurgical sustainability in the context of iron ores are listed in Table 18.

**Table 18:** Opportunities for Basic Research Related to Iron-Containing Minerals

Oxide types: Mixing of several oxide types suited for reduction: low-grade iron ores; ores of lower price; higher gangue-related impurity content, specifically S, P, and Si content variation; magnetite versus hematite ores; dispersion of nonferrous inclusions and effects of gangue elements
Oxide size: Use of inexpensive fine ores instead of expensive lump ores or sintered pellets and mixtures of these ore dispersions
Microstructure of ores: Size, inherited porosity (from sintering of the oxide fines); acquired porosity (from the solid-state direct reduction process); granularity and dispersion; percolation behavior of reductant gases through pellets and ores; sintering and abrasion effects of ores and pellets during furnace operations; interplay between microstructure and chemical composition of ores; gradient effects (of size, composition, density, defect density, fracture, gangue, porosity, pore percolation, transport properties, etc.)
Mechanical properties: High-temperature mechanical properties and fracture mechanics of the minerals and of the partially reduced feedstock

#### 6.2.3 Banded Iron Ores

Among the low-grade iron ores, the so-called banded iron ores are of high interest, because more than 60% of all global iron ore reserves belong to this group. These are mineral mixtures that occur as layered or laminated compounds containing at least 15% weight iron, mostly accompanied by iron-containing silicates, chert, or carbonate (mostly siderite) and stilpnomelane (a potassium, iron, magnesium aluminosilicate). The ores contain thin magnetite (Fe_3_O_4_) or hematite (Fe_2_O_3_) iron oxide layers ranging from a few mm to a few cm in thickness, Figure 20.

Several papers studied the suitability of such low-grade banded iron ores with fine liberation size as feedstock for conventional metallurgical reduction,^283^ for instance in large aggregates such as blast furnaces. It was mostly found that conventional mineral processing techniques were ineffective and showed less promising results in their reduction response.^284^

It was observed that the intermix of the hematite with the silicate phase opposed the direct use of these ores for iron making. Several reduction experiments reported in the literature, using different reductants, yielded mostly limited iron enrichment, except for microwave carbothermal reduction, which yielded an enriched iron concentrate with 58% iron at 85.6% recovery rate. Several other authors showed that by altering the conventional pyro- and hydrometallurgical processing routes, low-cost methods of mineral processing could be identified to refine and reduce such banded ores.^285,286^

Among the various pyro- and hydrometallurgical processing methods, several authors studied the effectiveness of methods such as magnetizing roasting, direct reduction, pyrometallurgical pretreatments on nonferrous ores, such as sulfidizing roasting, roast-flotation, segregation process, and matte separation processing, as well as hydrometallurgical pretreatments to extract valuable metal values by applying the leach-precipitation-flotation method and ion flotation. Interestingly, similar combined methods of chemical metallurgy and mineral processing were also applied to solid waste materials, such as nonferrous slags, smelter flue dusts and anode slimes.^283^

Interesting questions arise here with regard to the suitability of such ores, for example, for hydrogen-based direct reduction or for plasma-based reduction processes using hydrogen. In the case of the latter process, the possibility of reduction in the liquid oxidic state with the aid of hydrogen-containing reduction atmospheres of such mixed ores appears to be an interesting research topic, since the elements iron and silicon with their significantly different densities and oxygen affinites could possibly be well separated in such a process.^123,143^

**Figure 83:**
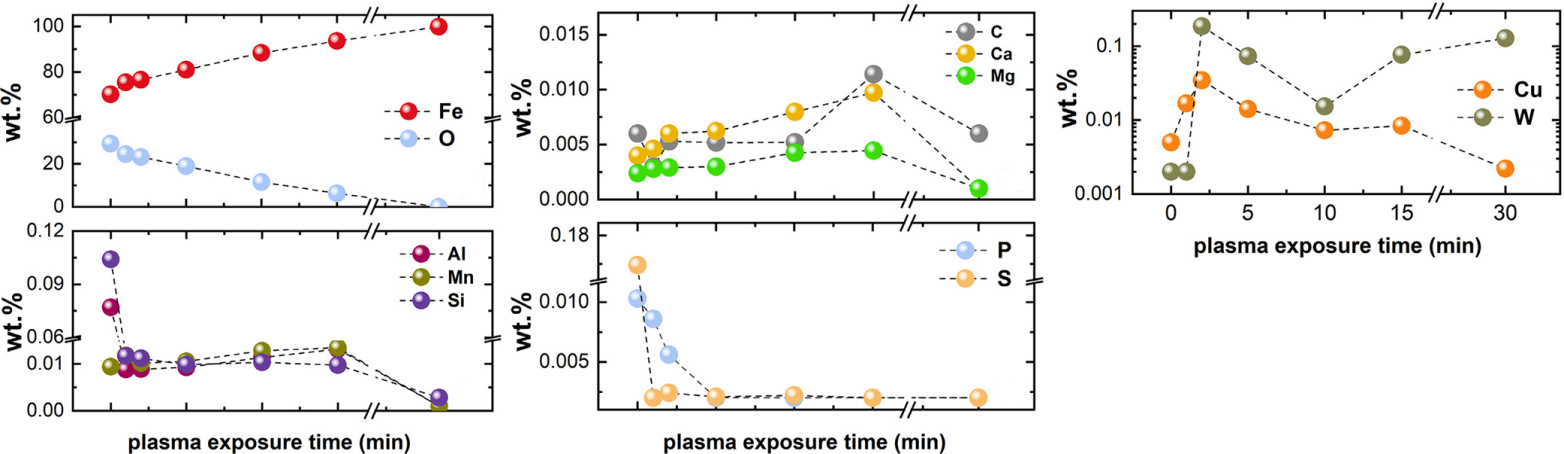
Example of a hydrogen-based liquid plasma reduction method which is capable of removing several critical impurity elements from low-grade ores.^143^ The figure is reproduced in modified form with permission from ref (143). Copyright 2021, Elsevier.

Also other impurities intruding from lower-grade ores could potentially be cleaned better by plasma-based liquid reduction processes using hydrogen-containing plasmas than by conventional solid-state direct reduction processes. In a recent paper on this topic,^143^ it was for example shown that gangue-related impurities such as sulfur, phosphorus, and even copper can be eliminated in plasma reduction processes, Figure 83.

These aspects, associated with the possible use of low-grade ores, are of special interest in connection with improved sustainability, since particularly the use of less profitable raw materials and the avoidance of expensive and non-sustainable ore benefication processes are of interest as alternative feedstock options when new sustainable metallurgy processes are developed.

#### 6.2.4 Porosity of Pelletized Mineral Feedstock Used in Direct Reduction of Iron Oxide

Another aspect in connection with mineral materials that serve as feedstock for sustainable metal production processes is their porosity as well as their mechanical properties at higher temperatures.^181,182,250,287−289^ In the latter context, in particular compressive strength, fracture toughness, and abrasion response matter.^290−294^ The reason for placing attention on these specific features is that the ores as well as their refined and sintered pellet agglomerates experience high static and dynamic loads in shaft and fluidized bed furnaces, all at high temperatures between 600 and 950 °C. During these loading scenarios they can undergo fracture, softening and abrasion processes which can lead to the accumulation of fine oxide dust between the ore pellets. This can lead to backing, sticking and loss of gas percolation.

These properties are of particular importance in the field of pyrometallurgy, especially for direct reduction, in which mineral solids (up to now mostly processed pelletized hematite iron ores) are exposed to a reducing gas mixture at temperatures above 500 °C (mostly in the range 800 to 900 °C). In the field of sustainable metallurgy, the use of hydrogen as a reducing gas (likely together with other reductant gases such as methane and syngas mixtures) will be of particular importance for this process in the future, a method referred to as hydrogen-based direct reduction.^123,182,241^

The importance of porosity for the reaction kinetics in hydrogen-based solid-state metal oxide reduction has been shown in several studies.^241,287,290,293^ A distinction must be made here between (a) the porosity which is inherited from the preceding sintering process of the fine ores into pellets and (b) the complex porosity topology which gradually builds up during the course of the reduction. The latter effect is due to the mass loss of oxygen. The oxygen atoms removed from the material by the reduction initially form vacancies in the metal oxide. These can move at the high reaction temperatures (possibly decorated by hydrogen) and reduce their energy by forming clusters of vacancies, which grow into nanopores and then coagulate and coarsen further.

The feedstock’s inherited porosity and its further evolution during reduction have an important effect on all mass transport and redox processes during hydrogen and mixed-gas direct reduction in sustainable iron making.^179,182,183,240,242,276,288,295^ The literature discusses in the context particularly the sluggish last stage of the reduction, namely that of wüstite into iron. This proceeds in 3 overlapping kinetic stages where the porosity and its percolation seem to play a big role. These stages are (a) oxygen depletion, (b) iron nucleation and (c) the growth of Fe^296,297^ (hydrogen is a fast diffuser in direct reduction and is thus not considered a bottleneck for the overall reduction kinetics).^275^

The kinetics of the first step^298,299^ relates to oxygen diffusion to the next surface. This depends specifically on the pore and defect structure, i.e. on its microstructure. An originally fully dense iron oxide has obviously a longer diffusion length for oxygen and also fewer free-surface nucleation sites than an oxide material with multiple pores. Bahgat et al.^300−302^ studied iron nucleation in wüstite and found a high number of iron nuclei near grain boundaries, where transport coefficients are higher and nucleation barriers are lower. Bahgat et al.^300−302^ interpreted this finding consequently in terms of faster transport of vacancies and divalent iron cations via interface diffusion.^303^ Hayes,^298,304,305^ Turkdogan^239,240,276^ and Gleitzer^306−308^ classified the morphologies during reduction into porous iron, porous wüstite covered by iron, and dense wüstite covered with dense iron. After the nucleation of iron inside of the wüstite the last reduction stage was suggested to consist in the growth of iron layers around the wüstite islands.^298,305,309^ Of importance for the kinetics during this last stage seemed to be the defect state of the iron layers that form a shell-like layer around the gradually shrinking wüstite.^276,299^ If the iron films around the oxides form closed layers and have no internal defects such as interfaces, pores, and cracks etc., outbound oxygen diffusion through these iron layers is slow.^310−312^ In other works it was found that fracture and porosity were clearly shown to allow much faster surface and defect diffusion of oxygen to the nearest surface,^287,301,313,314^ where it recombines with hydrogen to form water.^304^ This means that wüstite reduction into iron can be a nucleation-controlled process, particularly during the initial stages, or a more oxygen diffusion-controlled process, particularly during the later stages, depending on the microstructure of the iron and of the remaining wüstite.^182,241^ This kinetic interpretation is plausible as the Fe_2_O_3_ to Fe_3_O_4_ reduction as well as the Fe_3_O_4_ to FeO reduction only stand for modest stoichiometric oxygen losses of 1/9 and 1/4 units of O, respectively, whereas in the final step from FeO to Fe, FeO loses a full unit of O.^295,315,316^

Patisson et al.^242,317^ and Bai et al.^183^ considered these experimental findings and developed more detailed redox models for hydrogen-based direct reduction processes, accounting for the granularity of the pellets.

Bonalde et al.^287^ studied the reduction of Fe_2_O_3_ pellets with high porosity through H_2_-CO gas mixtures. They concluded that the interface reactions and the oxygen diffusion acted as competing processes during the first reduction stage, and the internal gas diffusion as a rate-controlling step during the last stage.

**Figure 84:**
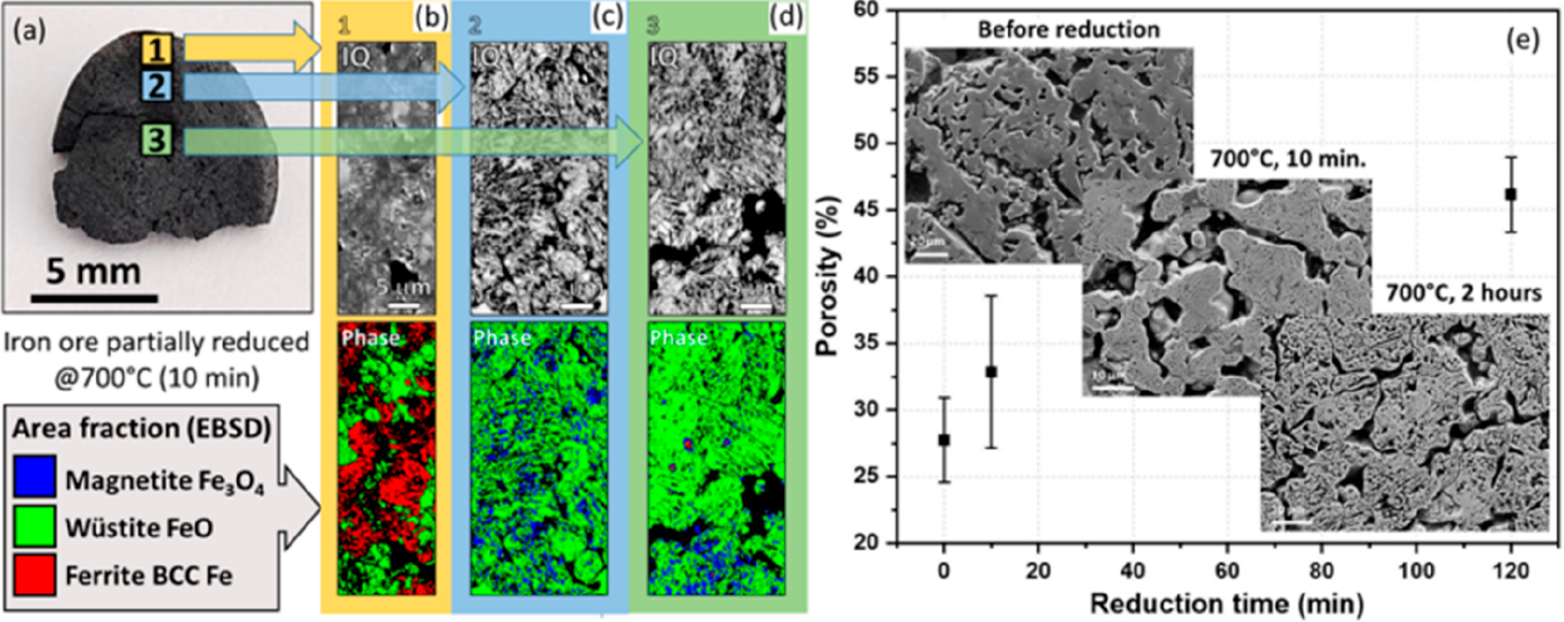
Overview of the influence of microstructure and porosity on hydrogen-based direct reduction of hematite pellets: (a) Fe_2_O_3_ pellet, obtained by commercial sintering of oxide fines, analyzed through the thickness after partial hydrogen-based direct reduction at 700 °C for a duration of 10 min. The color frames show the phase regions, as probed by electron backscatter diffraction (EBSD) in an SEM. (b) Image quality and phase map from region 1 (surface). (c) Image quality and phases from region 2 (mid-thickness). (d) Image quality and phase maps from region 3 in the pellet center. (e) Porosity change during reduction (pellet center). SEM, Scanning Electron Microscope. The figure is reproduced in modified form with permission from ref (241). Copyright 2021, Elsevier.

**Figure 85:**
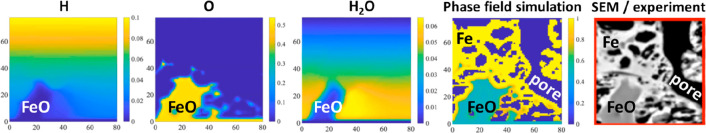
Phase field simulation, considering mass-loss-related stresses (i.e., assuming that the loss of oxygen linearly changes the lattice parameter of the oxide, hence creating a corresponding elastic stress); transport; reactions; and phase transformation for hydrogen-based direct reduction at 700 °C of wüstite (FeO) into bcc iron.^183^ The size of the simulation box is 8 μm × 8 μm. bcc, body centered cubic iron (ferrite). The figure is reproduced with permission from Bai et al.^183^ in modified form. Copyright 2022, Elsevier.

The use of electron microscopy on partially reduced commercial hematite pellets exposed to hydrogen at 700 °C showed an evolving porosity and crack distribution in the oxides (in initial and reduced states),^241^Figure 84. While the initial sample had 28 vol % pore volume from pellet manufacturing, the intermediate reduction stages reveal a large number of additional cracks and pores.^241^ The reduction at 700 °C for 10 min results in an increase in the free volume by about 5% and a smaller average diameter of pores, due to a huge number of small pores < 2 μm.^276,318^ After reduction at 700 °C for 2 h the porosity increases by >10%. This observation means that the microstructure of the reduced pellets does not only inherit its porosity from pellet processing but also acquires additional free volume, together with multiple lattice defects (e.g., dislocations and cracks). Figure 84 not only reveals a significant increase in porosity and tortuosity as reduction proceeds but also shows that gradients in the reduction state exist through the thickness of the pellets. Only a few core–shell structure features can be observed in the individual grains (i.e., with FeO in the center, enclosed by a closed ferrite iron layer around it); i.e., the reduction process observed experimentally does not agree with the topological scenario mapped by the classical core–shell model.^288^ The arrangement of the emerging iron phase regions, scattered amidst the FeO, also implies that the classical shrinking core models do not consider the pellet’s real microstructure. This observation suggests to use instead phase-field simulations,^319−324^ in which pores, cracks,^325−329^ elasto-plastic deformation and other microstructure features and the associated topo-chemical effects can be explicitely considered,^19,20,23,36,37^Figures 64 and 85.

#### 6.2.5 Pelletized versus Fine Iron Oxide (Fines) Mineral Feedstock

The enrichment and mechanical stabilization of ores (mostly oxidic hematite iron ores) is achieved by pelletization via sintering. This treatment is a very cost-intensive process step which is associated with high energy consumption and high CO_2_ emissions.^290,330−332^ They amount to about 55–65 kg CO_2_ per tonne of iron ore pellet produced. About half of this amount comes from the actual mineral extraction, processing and transportation of the raw materials, fuel and electricity.^97,126,333^

**Figure 86:**
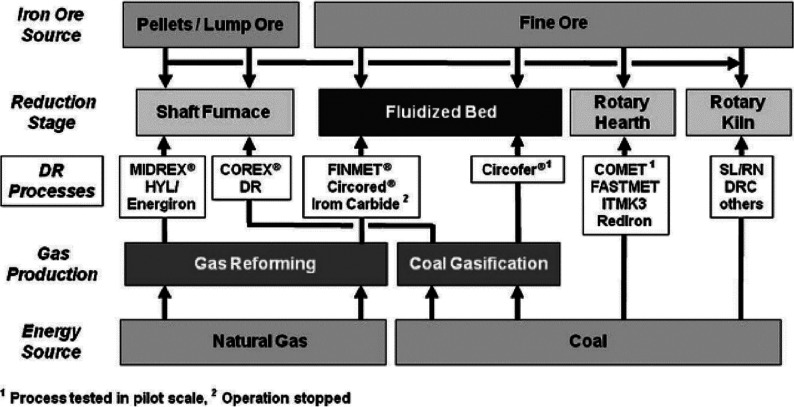
Classification of different types of (hydrogen-based or hydrogen-containing) direct reduction methods (showing in part their commercial trade names) in conjunction with different alternatives regarding the required granularity and sintering of the corresponding ores in conjunction with different types of reductants.^336^ Figure is reproduced with permission from ref (336) under an open access Creative Commons CC BY license. Copyright 2022, MDPI.

For many of the conventional pyrometallurgical processes, this form of enrichment and stabilization of the starting ores is of great importance in order to achieve an appropriate mechanical stability and percolation capability of the raw materials and reducing agents in large-volume counter-current reduction reactors. Some process pathways (liquid or solid reduction), feedstock types and dispersion (pellets, lump, fines) and reactor types (shaft, fluidized bed) are shown in Figure 86.^334−336^

If, however, in the course of sustainable metallurgy, fundamentally new reduction processes have to be developed anyway, which then do not work with fossil reducing agents but with sustainable reducing agents such as hydrogen, ammonia, or renewable carbon carriers, the types of ores used in these novel process variants can then also be considered as an additional variable set An important example in this direction is reducing plasmametallurgy,^143,145,254,337,338^ in which untreated ores can be used directly. A second example is the use of fluidized bed reactors for powdered fine ores, which also do not require any upstream sinter compaction.

A very important aspect that needs to be taken into consideration when shifting from a pellet-based shaft reactor design toward a fluidized bed reactor design, which uses powder-type oxides as feedstock, is the so-called sticking phenomenon.^335,339,340^ This effect refers to the undesired sintering of the partially or fully reduced powder particles during operation which can lead to the blocking and freezing of the entire reactor dynamics. This means that the design of efficient fluidized bed reactors that could possibly work in conjunction with the use of hydrogen as reductant requires a good understanding of the complex interplay between the powder size distribution, its reduction kinetics, its sticking behavior, and the fluid dynamics in the reactor.

Depending on the production site and the underlying mineral, typical size distributions of fine ores range from 100 nm and below to sizes of up to 100 μm and larger. This means that—depending on their origin—these fine ores can span size distributions of more than 3 orders of magnitude. Also, they can be endowed with similar impurities as coarse and lumpy ores. This is an important detail because the use of fine ores in corresponding fluidized bed reactors must satisfy a certain reproducible size distribution in order to achieve sufficient metallization and reduction kinetics in interaction with the fluid dynamics of the reactor without causing undesired particle sticking and thus freezing of the reactor.

**Figure 87:**
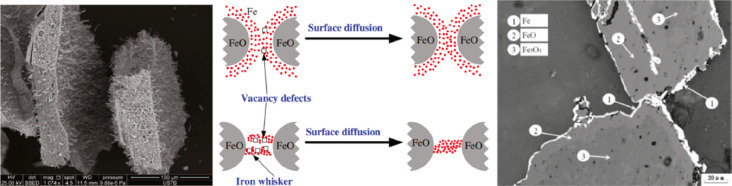
Iron oxide sticking mechanisms during solid-state reduction of fines.^343^ Figure is reproduced with permission from ref (343). Copyright 2020. J-Stage.

So far the sticking problem among the partially reduced iron ore particles has hindered a more widespread adoption of iron ore fines as inexpensive feedstock. Several author groups have studied this phenomenon,^275,340−342^ for the case of different types of iron ores and also for other metal oxides. For example Guo et al.^343^ studied the sticking behavior of fines in fluidized bed direct reduction iron making. They suggested as main reasons for sticking the accumulation of newly formed metallic iron on the surface of the iron ore fines and the formation of iron whiskers among adjacent particles. As a possible solution they suggested to change the surface properties during the reduction of iron ore fines, so as to reduce the surface viscosity, considering both the fine particle itself and the external reduction conditions, Figure 87.

Zhong et al.^341^ investigated the sticking behavior of hematite oxides and also of fully reduced iron powders in a fluidized bed reactor. They found that the amount of iron needed to stick decreased with increasing fluidization temperature. Different from other studies, they did not find whiskers in their experiments, and the sticking response was evaluated in terms of the surface nano- and microstructures of the freshly reduced iron that had formed on the surface of the oxide particles with respect to cohesiveness and sticking tendency. They also reported that owing to the interface reaction the sticking temperature of hematite was always below that of iron particle sticking for particles of the same size ranges. Due to the sintering mechanism via surface diffusion, the tendency of sticking was enhanced by the progressing reduction reaction through reinforcing mass transfer to the particle surfaces. Therefore, even when iron whiskers were suppressed during reduction, the sticking phenomenon could occur due to surface-dependent mass transfer, diffusion, and agglomeration among cohesive particles.

Zhang et al.^344^ conducted a systemic study where Fe_2_O_3_ particles with 150–224 μm diameter were reduced in a fluidized bed setup with CO-N_2_ gas mixtures at 700–900 °C. They found that more sticking was observed with higher temperature and accelerating reduction rate. Also, they stated that sticking depended strongly on the metallization ratio, indicating the importance of the frequency of iron-to-iron contact during collision. The reduction degree had only indirect influence on sticking.

#### 6.2.6 Bauxite Mineral as Raw Product for Aluminum Synthesis

Bauxite is a widespread mineral mixture from which aluminum oxide (alumina) is extracted, which then serves as oxide feedstock in fused-salt electrolysis for aluminum production.^79,345^ Bauxite usually occurs in the form of thin weathering layers in shallow depth in tropical and subtropical regions.^79,260,346^ The layers are often only 2–5 m thick, found just below the surface or even directly on the ground. This allows the use of inexpensive open pit mining methods for extraction. Global production is about 250 million tonnes, with an annual growth rate of 5%, Figure 1.

Bauxite contains different minerals in varying concentrations. The important ones that carry the highest metal fractions are the aluminum hydrates boehmite γ-AlO(OH), gibbsite (hydrargillite) γ-Al(OH)_3_, and diaspore α-AlO(OH). Other minerals are the iron oxides hematite Fe_2_O_3_ and goethite FeO(OH), the clay mineral kaolinite, and small amounts of the titanium oxide anatase TiO_2_. Gibbsite-rich bauxite is usually preferred in the metallurgical sector because it can be refined at lower temperatures than the other hydrates, a feature with high relevance for more sustainable aluminum production.^7^

According to their geological evolution, in laterite bauxites which form as a result of weathering of aluminum-rich rocks, silicate bauxites and carbonate bauxites can be distinguished. The economic importance of the carbonate bauxites, which usually occur in Europe weathered clay-rich deposits, has decreased compared to the richer laterite bauxites from the tropical belt, Table 19.

**Table 19:** Composition Ranges (in weight %) of Dry Karst Bauxites (Carbonate Bauxites) and Laterite Bauxites (Silicate Bauxites)

Mineral	Karst bauxites	Laterite bauxites
Al_2_O_3_	45–60	54–61
SiO_2_	3–7	1–6
Fe_2_O_3_	15–25	2–10
TiO_2_	2–3	2–4
CaO	1–3	0–4
Gangue elements: Zn, Sc, V, C, ...	traces	traces

Almost 95% of the bauxite produced worldwide is used to make aluminum through the Bayer process. This is a wet leaching process at 150–200 °C used for refining bauxite into alumina, the feedstock to extract aluminum. It involves dissolving the aluminum-containing minerals in the bauxite (mostly gibbsite and boehmite) in a caustic soda solution to produce a highly concentrated alumina solution. The solution is filtered and the alumina is precipitated out using seed crystals. The remaining iron-rich residue is known as red mud. The precipitated alumina is then washed, dried, and calcined to remove any residual impurities. This material is then reduced to aluminum metal by fused-salt electrolysis.

In the context of metallurgical sustainability, the composition of the bauxite mixture is therefore of high importance, since it also determines the chemical composition of the red mud. Red mud is currently an industrial waste material, which, however, could in a future sustainable metallurgical sector serve as raw material for further synthesis processes, due to its appreciable content of iron, titanium and several rare metals, Figure 78.^79,82,281,347−349^

The specific red mud composition and volume (per volume of metal produced) depends primarily on the aluminum oxide content of the bauxite used. This is between 53% for West African and Australian bauxite and around 38% for ores from Australia and Central Asia. The yield of the chemical decomposition in the Bayer process also plays a role, which typically lies between 94–96% and depends on the specific technology used.

Depending on the type of ore, between 1.8 and 3.2 tonne of bauxite (dry) are used for the production of 1 tonne of alumina, around 2.3 tonnes in global average. For each tonne of alumina produced, about 0.5–1.5 tonne of red mud (about 0.7 tonne in global average) are generated. When set in relation to a tonne of primary aluminum produced, this translates to about 1–3 tonnes of red mud. Consequently, in the course of the global production history of aluminum, about 4 billion tonnes of red mud have been deposited worldwide so far, Figure 78.

**Figure 88:**
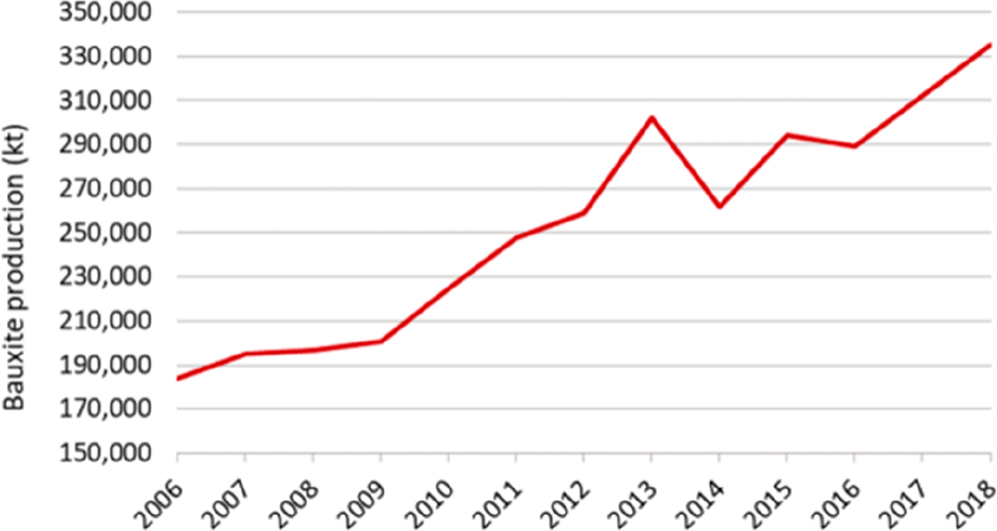
Trend in bauxite production.^345^ Figure is reproduced with permission from ref (345). Copyright 2020, EU Open Data Portal.

Another important sustainability aspect is the absolute magnitude of the amount of material that is mined, moved, and refined for aluminum production. Aluminum is the most important nonferrous metal in terms of production volume. The huge annual quantity of material required—compared to many other metals—alone translates to a likewise staggering corresponding energy and CO_2_ footprint.^7,17,350−353^ Only iron, with nearly 2 billion tonnes produced per year, comes at an even larger amount of raw, mined and deposited materials.^2^ Besides the relationship between the bauxite input and the final metal yield, also the volume and weight of the moved soil must be considered. For the production of 1 tonne of primary aluminum metal, not only about 5–7 tonnes of bauxite (measured in the form of a wet raw ore) are used, but also about 3 tonnes of dead rock (also called overburden) are moved, dissolved, and transported, Figure 77. This means that on average 7–9 tonnes of solid residues are produced in the various extraction processes for 1 tonne of primary aluminum, Figure 88.

As stated above, bauxite deposits occur mostly in the form of a few meter thin horizontal layers, leading to very extensive large area surface mining. For example, an opencast mine with an average production of 2 million tonnes of bauxite per year and an assumed deposit thickness of 5 m on average would require a mining area of about 20 ha per year (i.e., about 40–50 soccer fields) for the extraction of the ore alone.

Besides the bauxite, 85–100 kg of caustic soda, 50 kg of lime, 330 kg of (currently fossil) fuels and about 250–420 kWh of electrical energy are needed to produce 1 tonne of aluminum oxide in the Bayer process. In addition, the average water consumption in the Bayer process is 1.4 m^3^ per tonne of alumina. The average specific energy consumption in the Bayer process is about 12 GJ per tonne of alumina. About 90% of this energy demand is covered by coal and natural gas and only about 10% by electricity.

**Figure 89:**
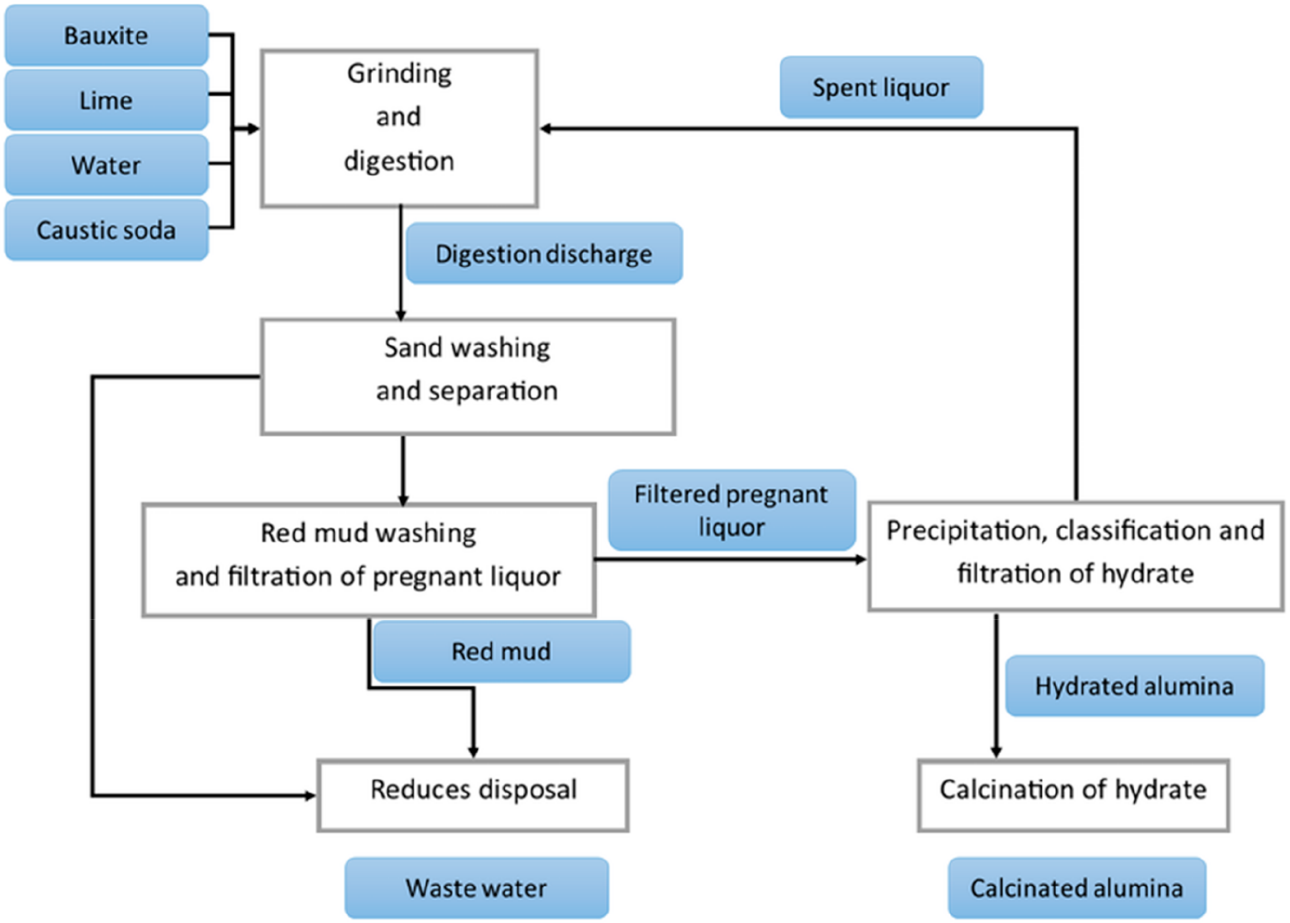
Production and waste stream in aluminum production.

Several authors made suggestions to make the Bayer process for alumina production from bauxite more sustainable.^79,260,348^ They discussed and evaluated the different process steps involved, the alumina yield, energy consumption, and the amount of waste produced, Figure 89. Interestingly they came to the results that the Pedersen process variant which is based on the processing of laterite ores with a low ratio between the target oxide Al_2_O_3_ and the accompanying Fe_2_O_3_ is actually more sustainable than the Bayer process with regard to the absence of red mud production and with production of consumable added-value by-products such as pig iron and gray mud. In contrast, the Bayer process is not well suited to digest such lean ores and produces the well-known huge quantities of red mud and also comes at high alumina losses. The Pedersen process includes a smelting step and uses lime (unlike the Bayer process), which makes the process less affordable, but the integral technology can in the future become more cost-efficient due to the production of valuable and consumable pig iron and gray mud.^348,354^ Also, the Pedersen process produces no red mud. This means it does also not create any downstream burden associated with dumping, neutralization or re-mining. A disadvantage of the Pedersen process is that it currently uses coke for smelting, hence creating CO_2_ as a redox product. This means that the Pedersen method must be redesigned in a way to replace the coke by non-fossil reductant and/or capture and reuse the CO_2_.

#### 6.2.7 Sustainability Aspects of the Mineral Feedstock for Copper Production

Copper is found only very rarely as reduced, pure metal. In mineral form it occurs as copper sulfide (e.g., in minerals such as chalcopyrite and chalcocite), as copper oxide (e.g., in the form of cuprite), and in copper carbonates (e.g., in the minerals azurite and malachite).^355^ As many of these established and commercially accessible copper minerals have meanwhile rather low metal content (see details below), in some regions copper arsenide minerals gain momentum.^356^ Examples are enargite (Cu_3_AsS_4_), tetrahedrite (Cu_12_Sb_4_S_13_) and tennantite (Cu_12_As_4_S_13_). They are found in part together with oxidic and sulfidic copper deposits and may contain significant amounts of copper along with small amounts of arsenic. The mining industry tends to avoid exploiting these deposits, because arsenic is a serious threat to health and the environment. A few of the enargite-containing deposits are of special interest because they can contain also gold and silver. The main challenge here however lies in the separation of the arsenic-containing phases from the other minerals.

**Figure 90:**
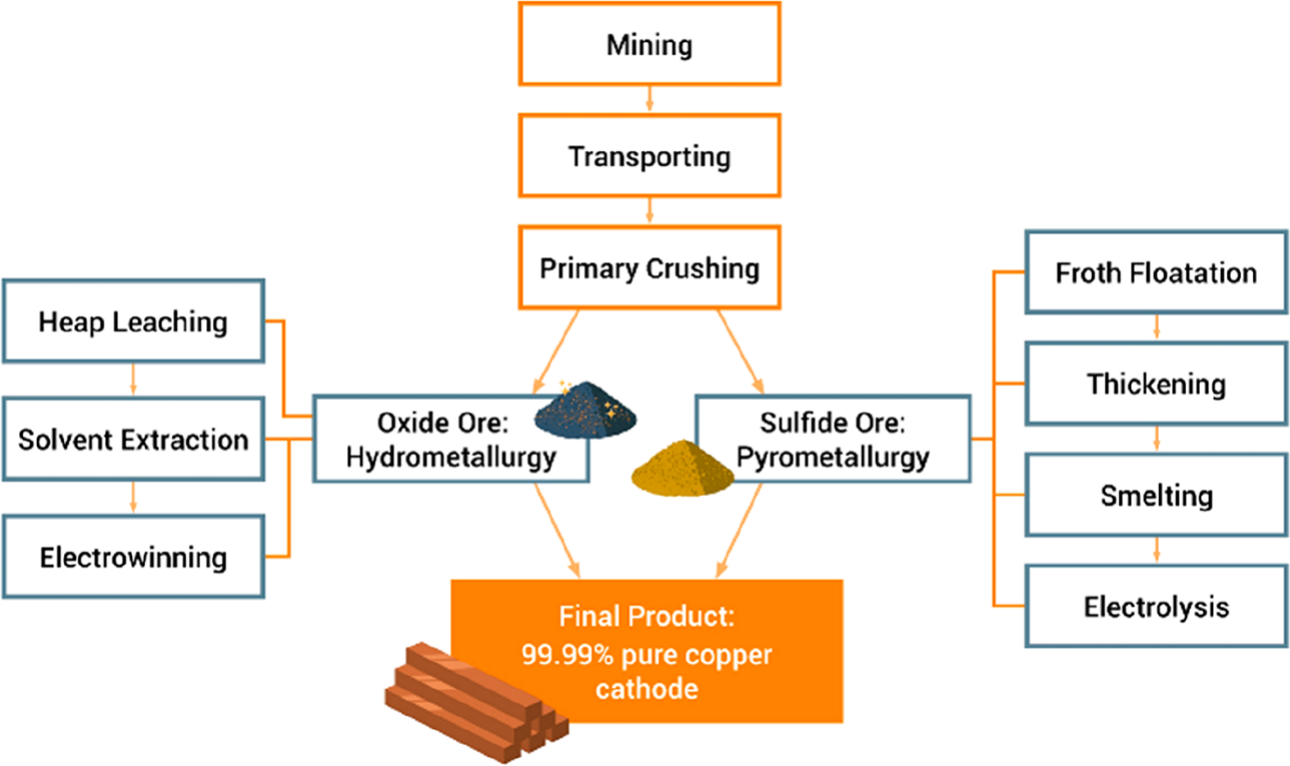
Different reduction and purification processes are used for the recovery of copper form (left) copper oxide ores and (right) copper sulfide ores.

Most copper ores contain less than 1% copper. Due to the differing chemistries of the two most prevalent forms of the ores, i.e. copper oxides and copper sulfides, two different reduction pathways are used, in extractive metallurgy, namely, hydrometallurgy for the oxides and pyrometallurgy for the sulfides, respectively, Figure 90.

The former ore type (oxides) is processed using aqueous (water-based) solutions to extract and purify copper from copper oxide ores, usually in the three steps heap leaching, solvent extraction and electrowinning.

The latter types of ores (sulfides) are processed using physical steps and high temperatures to extract and purify the copper, usually along the four processing steps froth flotation, thickening, smelting and electrolysis.

Near the earth’s surface, copper oxides are the more common ore type, but the deposits are mostly low-grade ores with a low copper content. Oxides can still be mined profitably even if this method necessitates extracting and processing more ore. On the other hand, copper sulfides are less common, yet they have higher copper concentrations so that ultimately a higher copper yield is achieved when extracted from sulfide ore deposits.^84,199,357^ Although the expenses of processing are higher, more copper can sometimes be retrieved, depending on the specific mine site. In the different deposit sites, mineral composition, concentration, and quantity can vary significantly, affecting the respective ore processing method that will finally be the most profitable one. The majority of the ores are put into primary crushers, to reduce them to the size of a golf ball.

#### 6.2.8 Ores and Sustainability Aspects in the Primary Synthesis of Titanium

Titanium is the 10th most abundant element in the Earth’s crust, and it is bound in more than 100 different types of titanium-containing minerals. However, only a few of them are of economic relevance. For the production of titanium metal, mainly rutile (TiO_2_) and ilmenite (MeTiO_3_ where the symbol “Me” stands mainly for Fe, Mn or Mg) are used. The level of TiO_2_ content is a measure of the quality of the raw materials. For the extraction of titanium and titanium dioxide, rutile is preferred because of its high TiO_2_ content, despite its lower occurrence compared to ilmenite.

The production steps associated with the main downstream products made from rutile, i.e. titanium tetrachloride (TiCl_4_) and titanium dioxide, are very energy-intensive processes that result in significant emissions of greenhouse gases and other pollutants, making it important to adopt sustainable practices in this field.^358^ Several specific sustainability concerns deserve attention in that context. The processing of titanium ore is water-intensive and can result in the depletion of local water resources, and the large energy consumption of the production of titanium is currently provided by fossil fuels, thus contributing to greenhouse gas emissions. Also, the refinement comes with significant waste generation, including also hazardous waste products.

Process steps of specific interest in that context are for example the important chlorination step. In this part of the process chain, the rutile is mixed with chlorine gas to produce titanium tetrachloride (TiCl_4_). Next the titanium tetrachloride is purified by distillation to remove impurities. In the subsequent oxidation step, the purified titanium tetrachloride is turned into titanium dioxide. This is usually done by reacting the titanium tetrachloride with oxygen in a high-temperature reactor or by passing it over a heated catalyst, both being very energy-intense process steps.

#### 6.2.9 Ores for the Primary Synthesis of Nickel and Cobalt

Demand for nickel and cobalt ores is expected to nearly double from the current 2.2 million tonnes by 2030, driven by the vehicle battery industry. An important sustainability aspect related to the extraction of metals from their mineral feedstock is the dilution of the elements in these rocks, a feature which also determines the energy and greenhouse gas emissions associated with their extraction. While the primary synthesis of many mass-produced metals such as iron and aluminum benefits from the fact that most of the underlying minerals that serve as ores are relatively rich in these elements, many other metals, including for example copper, nickel and cobalt, only occur in very dilute form (often below 1 mass %), which makes it particularly challenging and CO_2_-intensive to extract them, Table 3. Particularly nickel ores are problematic as feedstock for primary synthesis: although it is the fifth most abundant element on earth with a share of around 1.7%, it is one of the rarer metals on the Earth’s surface and occurs only in low agglomeration states.^204,205,338^

The extraction of the metal from nickel-containing minerals proceeds via a variety of methods, depending on ore type and nickel content. The ores most used for extraction are laterites and sulfides. The former one prevails; i.e., about 70% of the world’s primary nickel production comes from lateritic ores and about 30% from sulfides. Although sulfides allow simpler and less costly extraction, production nowadays shifts more and more toward laterites, due to the increasing market demand, Figure 53. Laterite ores contain in average only 2–2.5% nickel, yet some garnierite pockets can contain as much as up to 40%.

Laterites are weathered surface deposits that account for nearly 75% of the world’s accessible nickel resources. They are now becoming a major source of nickel-containing minerals. Historically, sulfides were instead used primarily for production, because they are easy and inexpensive to process. Yet, current production is driven by a dramatic increase in market demand that cannot be met by sulfides alone, so that laterites are increasingly used also, Figure 53.

There are several types of laterite, limonite and silicate minerals. Garnierite (NiMg)_6_Si_4_O_10_(OH)_8_ is a commercially important nickel-magnesium silicate, usually the richest one in terms of the net nickel recovery outcome. Nickeliferous limonite (Fe, Ni)O(OH)·*n*H_2_O is the largest fraction of mined nickel and cobalt minerals from lateritic origin. Limonite deposits contain up to 2% nickel, while silicates may contain pockets of up to 40% nickel, but not more than 2.5% in average. This means that silicate ores are today the main source of nickel production. Most of the sulfidic ores contain nickel, cobalt, copper, and iron. Relevant nickel sulfide ores are pentlandite, (Ni, Fe)_9_S_8_ and pyrrhotite (FeS-Fe_7_S_8_). Chalcopyrite, CuFeS_2_, is the dominant copper mineral in these ores, with small amounts of another copper mineral, cubanite, CuFe_2_S_3_.

Mining, beneficiation, and processing of nickel-rich ores can be associated with high dust loads contaminated with nickel itself, copper, cobalt, and chromium and with large emissions of sulfur dioxide. The metallurgical extraction processes and opportunities for basic research in this field are described in the processing-related sections below. An interesting emerging alternative class of nickel feedstock is the so-called manganese nodules, which are discussed in the next section. Cobalt is a by-product from nickel extraction from laterites. It is extracted as a by-product from leaching of nickel laterite ores and smelting of nickel sulfide ores. Both processes produce an intermediate sulfide that is refined by hydrometallurgical techniques.

#### 6.2.10 Manganese-Copper-Nickel-Cobalt Nodules from Deep Sea Mining

**Figure 91:**
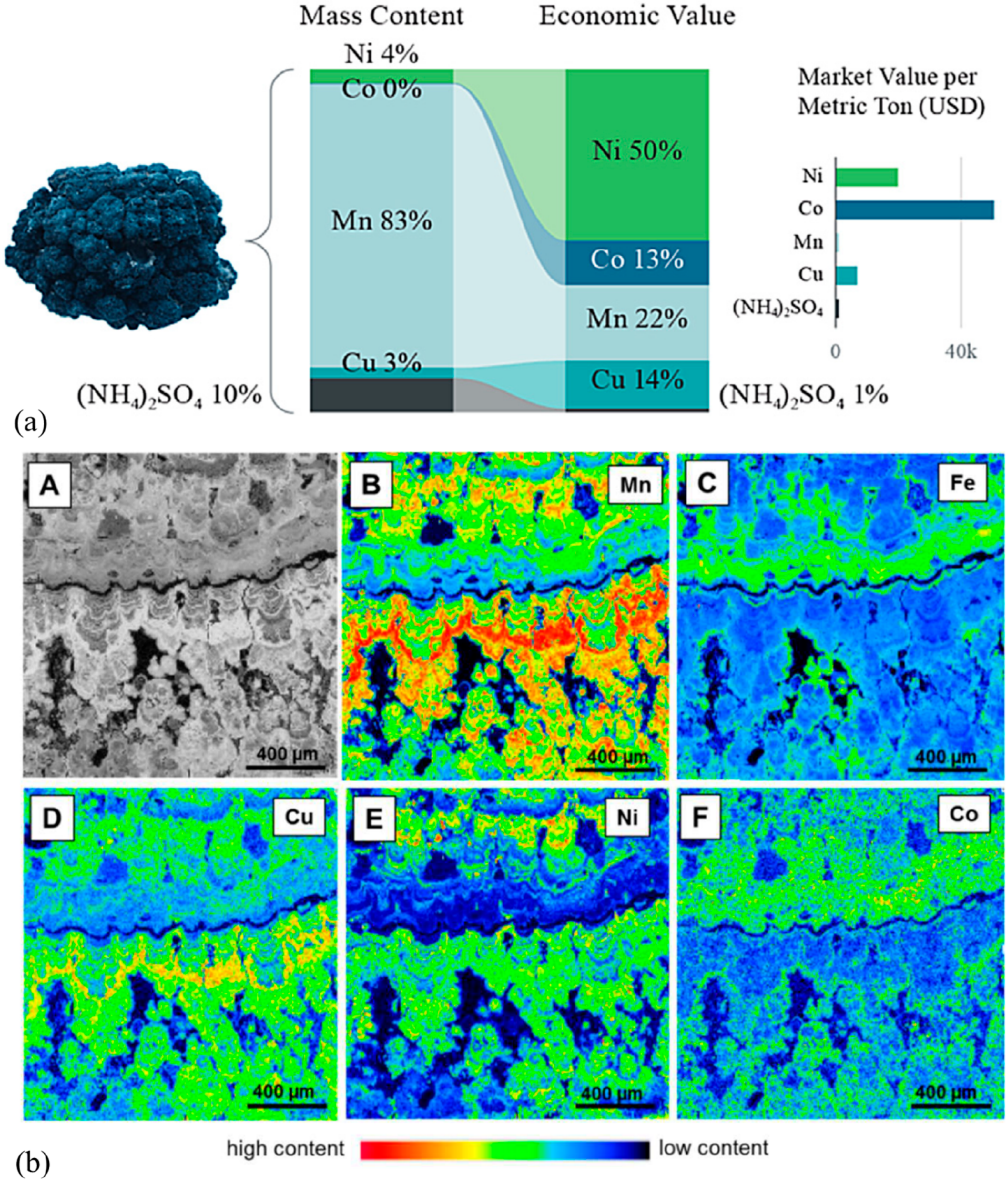
(a) Average mass-weighted chemical composition and price of elements in polymetallic “manganese” nodules.^29^ The average price development was estimated on the basis of metal price value projections from 2025 to 2055 estimated by CRU International. (b) (A) Backscattered electron (BSE) image and (B–F) chemical element distribution maps of Mn, Fe, Ni, Cu, and Co of growth structures of a polymetallic nodule from the Clarion-Clipperton Zone in the Pacific ocean. The data reveal different growth structures and the different Mn and iron-(oxy)hydroxides in an intergrown pattern.^360,362^ The figure is reproduced with permission from ref (362) under an open access Creative Commons CC BY license. Copyright 2018, MDPI.

Manganese nodules are polycrystalline minerals with a lamellar morphology, ranging from 1 to 15 cm in diameter.^359−362^ They are found in deep-sea plains below 3000 m. Their formation takes millions of years to build around a core of oxide minerals in oxygen-rich water. The nodules are actually polymetallic deposits, rich in several metals, some of which are very expensive and needed for the electrification of industry and society: besides manganese (25–30 wt %) they contain iron (<6 wt %), silicon (<5 wt %), aluminum (<3 wt %), and several trace metals. Some types of nodules are particularly of high interest because they contain also copper (1–1.4 wt %), cobalt (0.2–0.25 wt %), and nickel (1.35–1.5 wt %). In massive sulfide nodules, in addition to nonferrous metals such as copper, zinc, and lead, also even precious metals like gold and silver as well as trace metals like indium, tellurium, germanium, bismuth, cobalt, and selenium have been observed, Figure 91.

**Figure 92:**
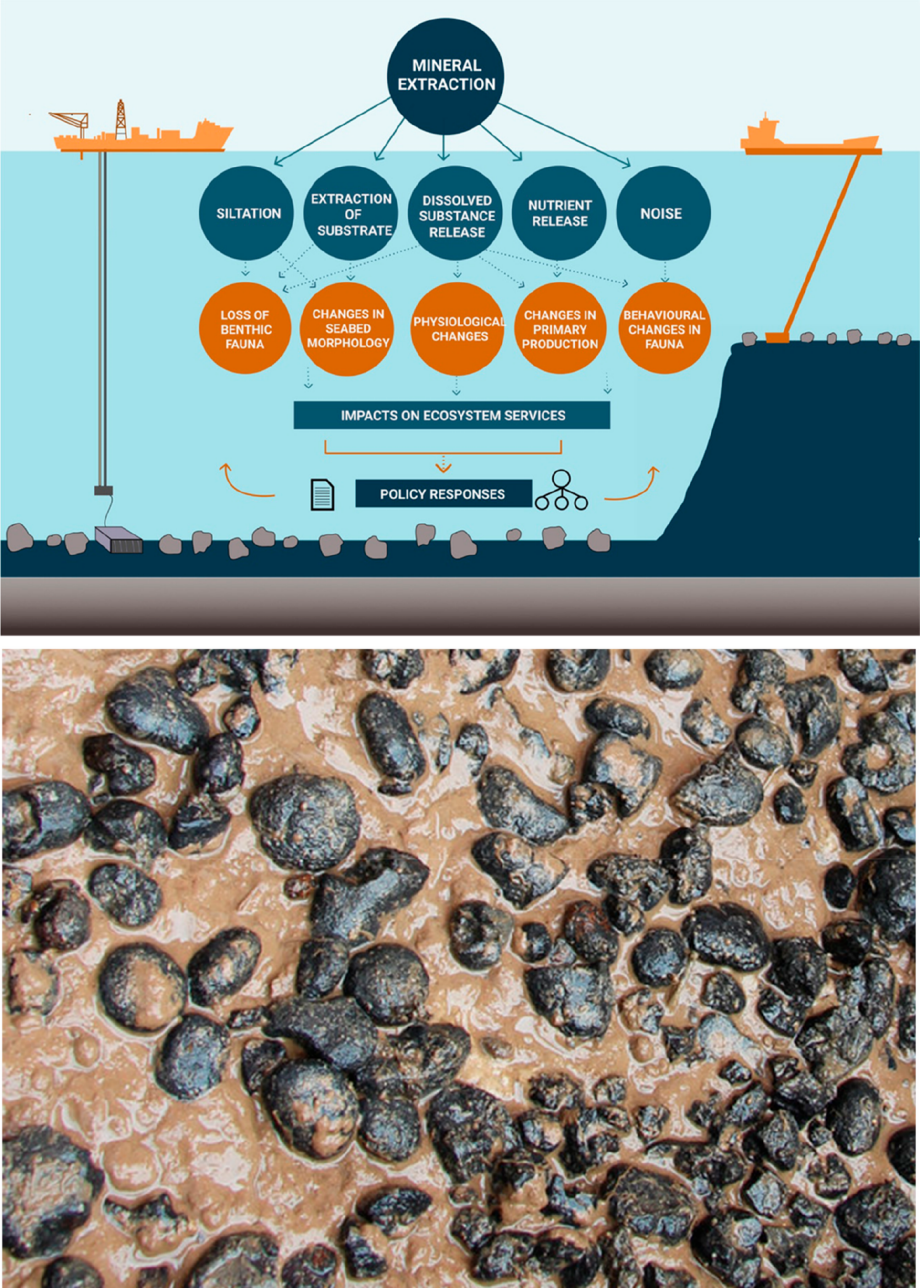
Top: Sketch of typical deep-sea deposit mining (e.g., of manganese nodules) and the associated risk factors and side effects.^368^ Bottom: As-mined manganese nodules on a belt. The figure is reproduced with permission from ref (368) under a 4.0 International Creative Commons license (CC BY 4.0). Copyright 2018, Elsevier.

The nodules are usually found loosely scattered on the seabed. In some regions only a few nodules per square meter occur while in others up to 1000 are found. In some areas they are so close together that up to 20 kg per square meter can be mined, Figure 92. The economically most significant deposit so far has been found in the Clarion-Clipperton Zone’s manganese nodule belt in the North Pacific’s equatorial zone.^363^ Additional deposits were found in the Southeast Pacific’s Peru Basin, the West Pacific’s Penrhyn Basin, and the central Indian Ocean. The Clarion-Clipperton Zone alone is approximately 5,000 km long and 1,000 km wide. Estimates suggest that it contains nodule deposits of about 25–40 billion tonnes.^364−366^ Nodules from the Clarion-Clipperton belt have high content of manganese (30 wt %), nickel (1.4 wt %), copper (1.1 wt %), and cobalt (0.2 wt %). Compared to any of the land-based ores, the nodules of this region alone contain about 3.4–5 times more cobalt, 1.8–3 times more nickel, and 1.2 times more manganese. The nodules also contain comparatively high proportions of trace metals such as titanium, molybdenum and lithium.^359,360^

The nodules have formed over millions of years, by deposition of the metals dissolved in the sea- and sediment water. The partitioning of the metals to the nodules through occurs diagenetic and hydrogenetic processes. Diagenetic enrichment is caused by the direct precipitation of metal oxides from marine sediment pore waters. In this pore water, some transition metals are dissolved as ions. They diffuse upward, propelled by the chemical potential gradient, to the seafloor, where they oxidize and precipitate, gradually forming concentric oxide layers around the core. Other metals dissolved in pore water, such as copper and nickel, become trapped in manganese oxides. They are primarily the result of microbial decomposition of organic matter. Manganese nodules get >80% of their metal content from sedimental pore water. Other nodules develop through a hydrogenation mechanism, a direct deposition of colloidal nano- to micron-sized hydrated manganese and iron oxide particles from seawater. The majority of these nodules form near submarine volcanoes, and their composition is determined by water chemistry and biogeochemical processes that take place between seawater and the particles it contains. Hydrogenogenic nodules accumulate more cobalt and rare earth elements than diagenetic nodules.

As the chemical composition and structure of these mineral agglomerates are distinctly different from those of other ores, processes for extracting (all of the) metals from them are still only accessible at laboratory scale.^367^ Conventional mechanical processing of nodules by means of magnetic separation, density separation, or flotation is often not feasible because the carrier phases of the metals (manganese and iron oxides) are very fine-grained and intergrown. Therefore, the nodules have to be completely broken down after drying and primary crushing. As discussed by Kaikkonen et al.,^368^ deep sea nodule harvesting can have several serious side effects during the mining process, as shown in Figure 92.

Several groups studied various types of sustainable approaches to recover the metal content form these precious mineral deep sea deposits.^362,367,369,370^ For instance, Friedmann and Friedrich^369^ developed a process in which the material is charged into an electric arc furnace. Chemical reduction of the oxides with carbon produced an iron-nickel-copper-cobalt-molybdenum metal phase. Furthermore, a manganese-rich slag is obtained, which was subsequently further reduced to ferromanganese and silicomanganese. Another product of the pyrometallurgical process was a calcium- and silicon-rich slag. The iron-rich metal phase formed in the first melting step was transferred to a converter for purification of the target metals nickel, copper, cobalt and molybdenum and their separation from the iron. The resulting iron-poor metal alloy was further hydrometallurgically refined using ion exchange methods, solvent extraction, or recovery electrolysis. Final products from their approach included for instance cobalt salts that are suited for the further downstream production of lithium-ion batteries.

Sommerfeld et al.^367^ developed what they referred to as a “zero-waste” metal recovery approach for the pyrometallurgical processing of manganese nodule slags obtained from the metal extraction from polymetallic deep-sea nodules. In this process the manganese that had been discarded in the slag was recovered in a second smelting step as ferromanganese. The authors had used thermodynamic simulations for modeling the individual smelting variants. For increasing manganese yield and to alter the composition of the metal alloys, different fluxes had been investigated.

Beolchini et al.^366^ studied the fungal-mediated leaching efficiency of valuable metals from such deep-sea polymetallic nodules, which they also had obtained from the Clarion-Clipperton Fracture Zone. Biometallurgical leaching was studied using Aspergillus and mixed cultures of *Aspergillus niger* and Trichoderma under different conditions. The results were compared to chemical treatments using citric acid. The authors found that 11 days of treatment with *Aspergillus niger*, growing under optimal medium conditions, produced the best results, yielding extraction of more than 80% of the copper, manganese, and nickel and 70% and 30% of the cobalt and iron, respectively. The findings showed that biotechnological processes, specifically fungal-mediated leaching, can be a more sustainable approach in terms of carbon footprint for the extraction of metals from such nodules, compared to chemical extraction strategies.

Several sustainability aspects deserve consideration when extracting metals from these nodules at industry scales. An obvious one is that the required deep-sea mining can result in the destruction of fragile habitats and species and in the release of pollutants into the ocean. Also, like for any other minerals, the extraction of metals from manganese nodules is a nonrenewable resource, and once depleted, these minerals will not be available for future generations. This means that recycling (e.g., of the coming huge quantities of vehicle batteries) must have highest priority over extracting metals from nodules. Also, it must be considered that the extraction of nodules from deep sea deposits poses significant technological challenges, including the development of mining technologies that can operate in the deep ocean and the management of waste generated from the mining process.

#### 6.2.11 Feedstock for Primary Lithium Production

**Figure 93:**
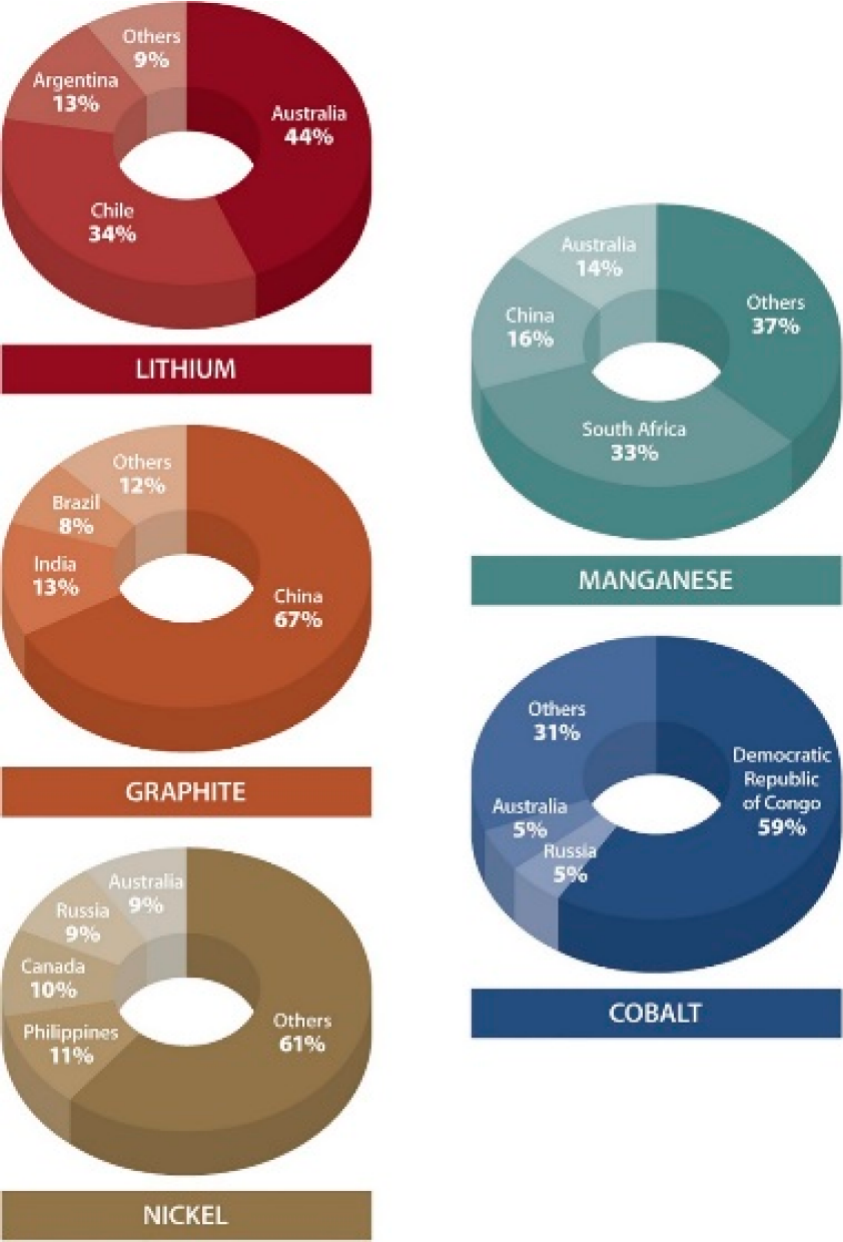
Regions of origin for the production of the main materials (lithium, nickel, graphite, manganese, cobalt) used in lithium-ion batteries.^137^ The figure is reproduced with permission from ref (137). Copyright 2019, Elsevier.

Like nickel and cobalt, lithium has become an indispensable material for vehicle batteries,^221,222^Figure 93–Figure 95. Generally, lithium can be extracted either from brines, in which lithium occurs as an enriched solute element in the groundwater, or from minerals in the form of hard rock deposits such as petalite, although the former source dominates.^141,371^ Sedimentary rocks are not (yet) used for lithium extraction on a larger industrial scale. Depending on the downstream production method, the lithium is processed into lithium carbonate, lithium chloride or lithium hydroxide. These products can then also be processed into elemental lithium through electrolysis.

**Figure 94:**
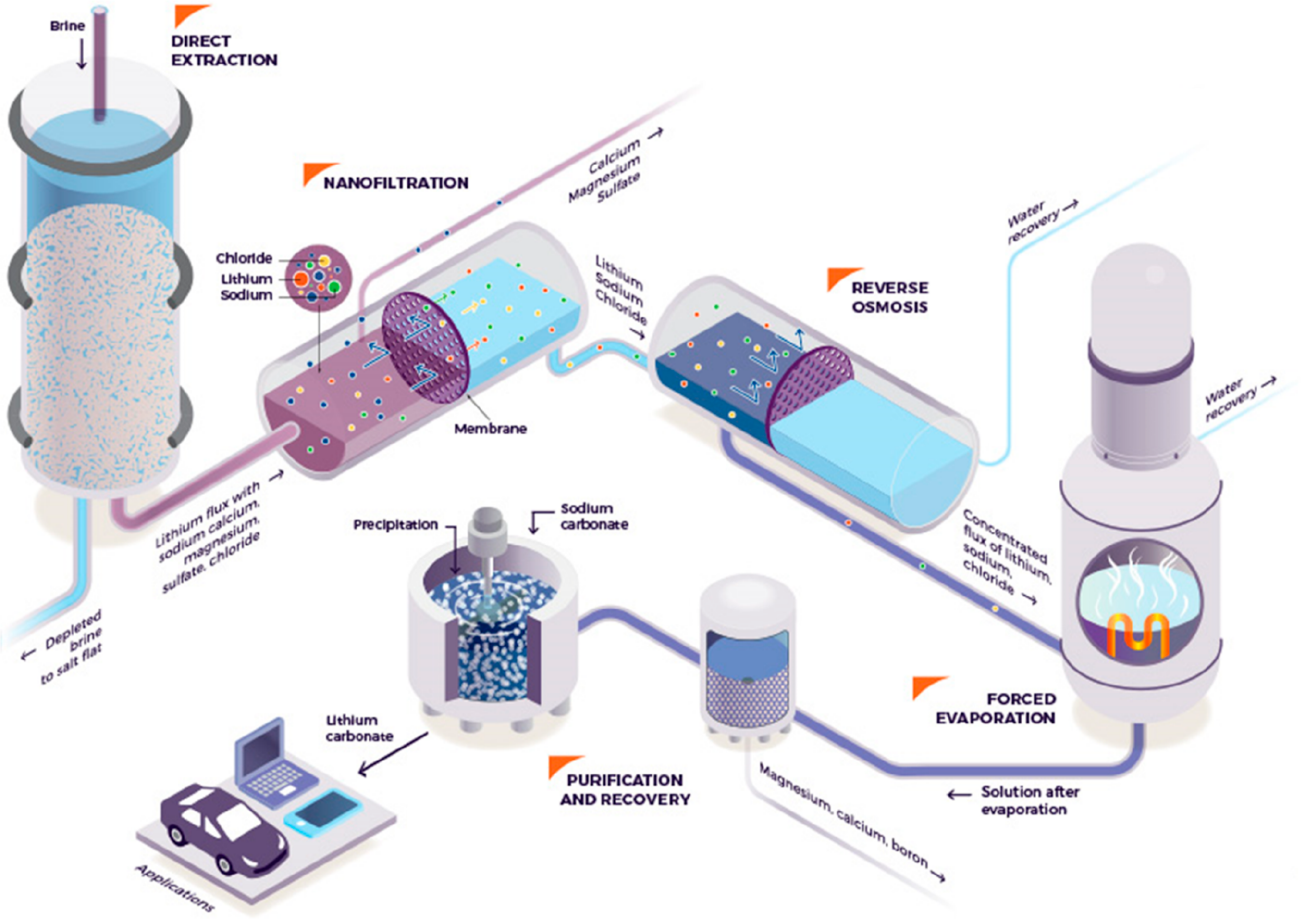
Sustainable approach to a lithium extraction process chain from deep groundwater brines. Figure reproduced with permission from Eramet.^375^ Copyright 2022, Eramet.

**Figure 95:**
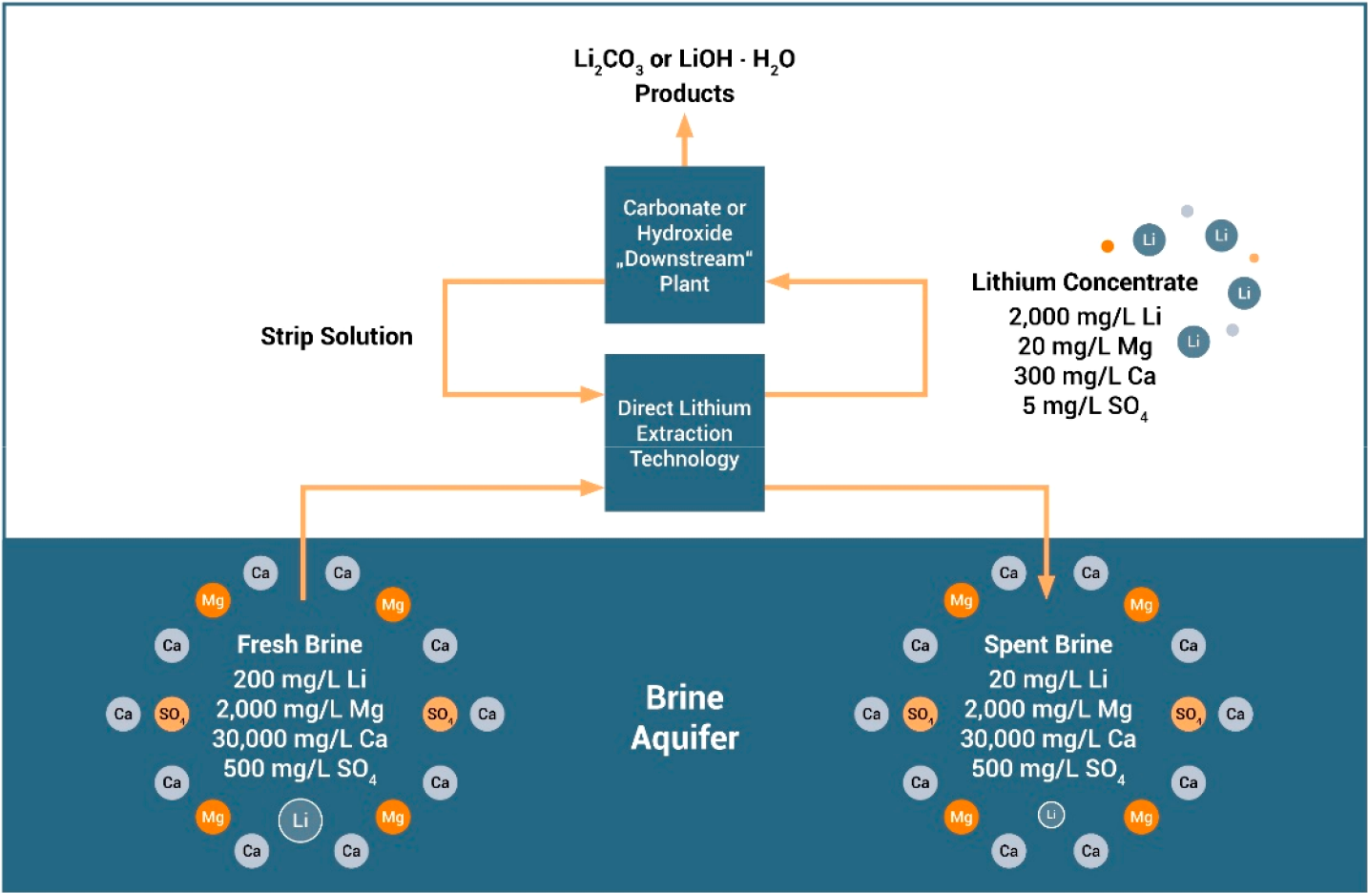
Details of the process chain for lithium winning which can be further coupled with geothermal heat recovery.

To extract lithium from brines, mineral-rich groundwater is pumped together with all its impurities into huge shallow basins. In these brine basins the lithium becomes gradually concentrated by natural evaporation of the water. The length of time spent in the pool is determined by the initial mineral water content, solar radiation and precipitation. A typical accumulation time in South American basins is 12–18 months. When the lithium content of the salt solution reaches 0.5%, sodium carbonate (soda: Na_2_CO_3_) is added to the brine. Approximately 1.8 tonnes of sodium carbonate are needed per tonne of lithium carbonate produced. Due to the lower solubility product of lithium carbonate, it precipitates.

Yet, for extraction from brines, even the currently existing evaporation ponds, although being very rich in lithium, require the occupation of huge surface regions, a process which has natural limits in many regions.

An alternative and likely more sustainable way of lithium winning emerges through the co-exploitation (using both the heat and the minerals) of deep groundwater brines.^372,373^ These resources can be tapped by combining geothermal heat and electrical power generation with lithium filtering. Common water sources used in this context have a temperature between 100 and 170 °C and come from 2 to 5 km depths. After filtering the lithium from this warm groundwater, it can be reinjected underground. Chemically promising lithium-carrying deep brines in Europe and North America that are accessible to such groundwater extraction approaches have a concentration of dissolved Li ranging between 100 and 400 mg per liter. Compared to the currently exploited near-surface brines in South America with up to 1500 mg per liter, concentrations in geothermal brines are thus relatively low. However, due to the high production rates of several tens of liters per second, several hundred tonnes of lithium-carbonate could be produced as a by-product per year from geothermal power generation, provided a sufficiently selective extraction method is available.

Dugamin et al.^372^ looked at the richness of these sources in more detail and conducted a systematic screening of the lithium concentration reported for about 3000 different samples of groundwater from 48 different basins worldwide. The highest values of lithium, namely, >10^2^ mg per liter, were documented for high-salinity waters with a total dissolved solid content above 10^5^ mg per liter, with a lithium concentration in the same range as brines from the most efficient salar mines. Conservative estimations made by the authors based on fluid volume and lithium concentration for specific deep reservoirs suggest that these lithium resources are comparable to salars and hard-rock mines (0.1–10 million tonnes of lithium). The authors thus concluded that lithium in groundwater from sedimentary basins could make a substantial contribution to the growing lithium market.

Due to the complex mineral composition of many deep groundwater geothermal brines, different extraction methods have been studied and adapted to match the specific local brine chemistry. The most discussed options for selective lithium extraction include direct precipitation, filtration, ion exchange with resins, liquid–liquid extraction, and sorption processes, where the latter approach shows promising results when using lithium-manganese oxide sorbents.^374^ In that context it is important that the extraction process is selective for lithium, with only minimal coextraction of other cations. Due to the high flow rates in such geothermal power plants of up to 100 liters per second, a methodology with fast reaction kinetics is required to realize the synergy between lithium and energy winning, necessitating temperatures of up to 200 °C and water pressures of up to 25 bar.

In the next processing step after mining, the won lithium carbonate is separated and treated with hydrochloric acid. Lithium chloride is dissolved, and gaseous carbon dioxide is generated. A vacuum evaporator is used to confine the solution until the chloride precipitates. This process step is done in special stainless steel or nickel tanks due to the highly corrosive nature of the hydrochloric acid. The elemental metallic lithium is then produced at large scale via fused-salt electrolysis at 400–460 °C from potassium chloride and lithium chloride (45–55 wt %).

Besides in brines and underground water, lithium also occurs in more than 140 different types of rock, yet commercial winning has only been applied so far to petalite, spodumene, lepidolite, amblygonite and eucriptite. Depending on the mineral origin, different extraction processes are used, described in the technical literature. At present, production from groundwater exceeds that from solid mineral materials by a factor of about 3 to 4, since the use of natural solar radiation in the enrichment of lithium-containing groundwater requires significantly less efforts and costs than mineral extraction.

### 6.3 Secondary Feedstock Types (Scrap)

#### 6.3.1 Types of Metal Scrap and Associated Research Challenges

The reduction of greenhouse gas emissions and energy consumption required for extraction and production can for most metals be drastically reduced when shifting from primary to secondary synthesis.^7,71,84,219,376,377^ Smelting metals from scrap, with its globally growing market, can be 30% to 95% more sustainable compared to primary synthesis, depending on alloy class, scrap quality and melting technologies. This means that the specific effects on sustainability depend substantially on the type of alloy, the scrap considered, and the recycling process; i.e., potential benefits associated with secondary synthesis must in each case be very carefully analyzed by a life cycle assessment. This applies particularly to the recovery of metals from highly integrated products such as in electrical and electronic components.^89,99^

In the metallurgical sector two types of scrap have to be principally distinguished, namely so-called “old” scrap and “new” scrap, Figure 11. The former refers to mixed and often quite highly chemically contaminated scrap from consumer products, infrastructures, buildings, machinery, appliances, etc. The latter refers to in-line industrial scrap that is collected already during production and downstream manufacturing, thus also referred to as in-line, “runaround”, or pre-consumer scrap.^9,167,378−383^ Examples are waste materials produced during casting, blanking, stamping or chipping. The amount of in-production scrap can be as small as a few percent for some special steels as well as for many precious metals, but it can be also as high as 90% and more, as for instance in the case of titanium when it is chipped into parts for aerospace vehicles.^11,150,219^ New scrap has usually a known and well-defined chemical composition and can be collected sort-specifically. In contrast, old scrap is mostly chemically contaminated and this type of mixed scrap is growing on the global market, often exceeding the amount of available new scrap. This means that future scientific works must address how to turn contaminated low-quality scrap again into useful products or processes.

It is in many cases not only ecological but also commercially attractive to switch from primary to secondary synthesis, provided that sufficient amounts of scrap are reliably available on the global market (i.e., equipped with robust supply chains and in sufficient quantity) and that the scrap is of high quality and well sorted.^10,116,165,188,384,385^ This means that in some cases the transition from primary to secondary synthesis substantially enhances metallurgical sustainability and can in some cases even reduce costs. This is an important factor for downstream customers of metallic materials, as the typical fraction of costs for the materials is in the downstream manufacturing sector for many products with 30–55% far higher than the energy costs (2–8% in manufacturing). Therefore, reducing the prices of metals through sustainable synthesis from scrap instead of the more greenhouse gas and energy-intensive primary synthesis can be economically very attractive, when reliable and projectable scrap flows can be established.^165^

**Figure 96:**
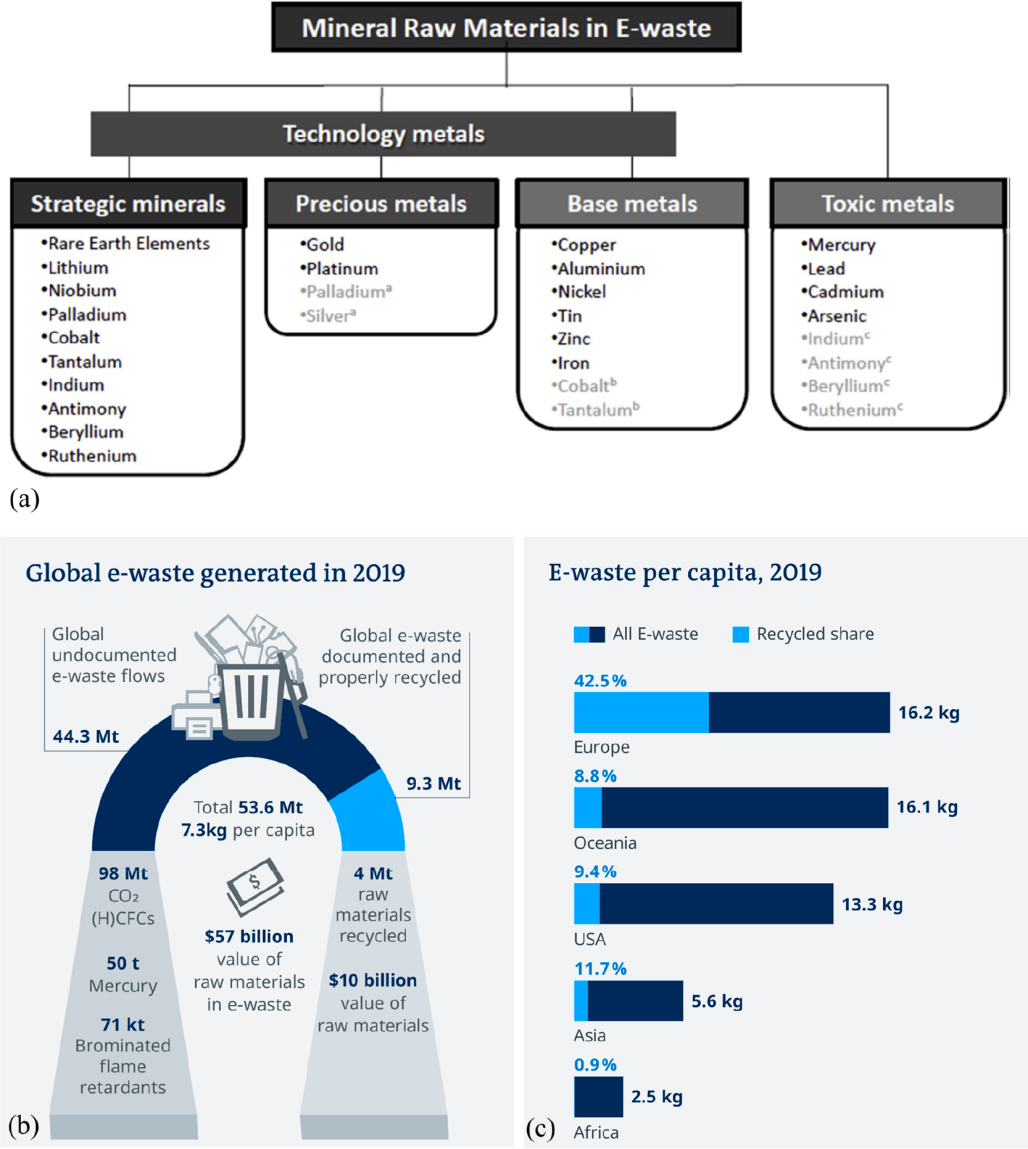
(a) Increasing use in the number of elements in advanced consumer and industry products. Particularly electrical and electronic parts can use up to 60 elements in a single product, often in nanoscale integrated form, which makes it very challenging to recover metals from such products (see also Figure 17). Waste from such products is also referred to as nanoscrap or nanowaste, due to the close integration and mixing of the material in such products, particularly in the electronic circuits and printed boards.^386^ The figure is reproduced with permission from ref (386). Copyright 2021, Elsevier. (b) Increase in global waste from electrical and electronic parts (data from the UN Global E-Waste Monitor for the year 2020).^24^ To give a reference, the total amount of electrical and electronic waste produced in 2019 alone has the same weight as about 350 huge cruise ships. Alone through this trend, billions worth of valuable metals such as gold, silver, and copper were dumped or incinerated in 2019 as electronic waste produced globally jumped to a record 53.6 million tonnes, or 7.3 kg when counted per person. The UN has forecast that the amount of such waste will increase to the staggering amount of 74 million tonnes by 2030.^24^ (c) E-waste per capita in different regions and recycled fraction. Figures (b) and (c) are reproduced with permission from ref (24). Copyright 2020, United Nations University (UNU)/United Nations Institute for Training and Research (UNITAR).

However, in such a quick assessment it must be taken into account that the winning of new metal in a circular economy is not necessarily generally cheaper or more sustainable, due to the high requirement for a very narrow selectivity in the collection and processing of scrap.^87,89,133^ This means that materials produced from scrap are only cheaper if the costs for their collection, separation, cleaning, and processing outweigh the costs of the primary synthesis, which is currently the case for only a few metallic alloys. This means that secondary synthesis can even have negative effects on sustainability, for instance if the scrap contains too many impurities or if its collection, sorting, and recovery (e.g., from highly dispersed or nanoscale mixed and integrated sources) cost more energy and CO_2_ than what is gained from melting. Examples for highly dispersed scrap are often found in packaging and for “nanoscrap” in the field of electronic equipment,^8,89,202^Figure 96.

These aspects reveal an essential and highly problematic target conflict in sustainable metallurgy, pointing at the difficulty in reconciling two opposing trends and constraints. The first one is the massive demand for highly sophisticated high-end alloys, made from chemically clean feedstock, for better strength, magnetic properties, high conductivity, optical appearance, corrosion resistance, high-temperature strength, hydrogen-embrittlement resistance, etc. Such metallic alloys are indeed urgently needed because of their beneficial indirect sustainability effects on many products and processes such as weight reduction, low electrical resistance, low magnetic losses, good creep resistance in high-temperature energy conversion, improved fatigue resistance for improved longevity, and so on.^2^ The second one is the likewise urgent need to turn the metallurgical sector away from less sustainable mining and primary synthesis methods toward the more sustainable secondary synthesis approach.^1,17,18^

In order to achieve the first goal, i.e. delivering advanced high-performance metallic materials to enable a transformation of modern industries and transport systems into more sustainable ones, it is usually necessary to work with very clean and well-defined materials in terms of chemical composition and within very narrow permissible impurity limits. However, this condition is usually in conflict with the quest of using a higher scrap content in the alloys, since often significantly higher impurity contents enter and gradually accumulate in the material via scrap use.^7^

This means that one of the most important future research tasks seems to lie in resolving this conflict of objectives. Specific research targets are on the one hand the need for a better understanding of the chemical tolerance limits for high-performance alloys. This can lead for example to improved basic insights on scrap-related impurity effects and—at least for some scrap-inherited elements—to a resulting redefinition of these chemical tolerance limits toward higher values.^7^ On the other hand, it is required (from the influx side where the scrap re-enters the value chain) to bring a smaller variety of alloys onto the market in the first place. This latter approach would make it much easier to separate the scrap by type. Also, in general it is required to intensify in-production scrap collection in concert with strict sorting and scrap separation. A further quest is to better understand the effect of less understood and new types of impurity elements which have not yet been studied very much and which intrude into the alloys from the use of mixed scrap coming from novel products such as electrical vehicles. The latter point has generally to do with the fact that most of the well-studied effects of impurity elements on alloys have so far mainly referred to those elements that enter the alloys from the ores or through partitioning from the reductants. These tramp elements, however, are quite different from those elements that can enter the material from impure, less well sorted scrap, also referred to as called “old” scrap. These aspects leverage a number of completely new research questions for a more sustainable metal sector.

Also, as will be shown below in more detail, collecting scrap and feeding it back into sustainable metallurgical production is again not a homogeneous research field but branches out into quite different subdisciplines, where different research approaches are needed. Examples are the smelting of bulk plain carbon steel scrap:^49,387−389^ e.g. from infrastructures, machinery and vehicles; recovery of rare earth metals from hard magnets that are “hidden” in many complex electrical and electronic products;^12^ platinum group metal elements used in nanodispersed form in catalysis;^11,235^ lithium from vehicle batteries or precious elements such as gold and copper from electronic scrap.^137,226,229,233,390^

All these quite different metals and their likewise very different dispersion in products on the recycling side and the specific factors of influence of the various impurity elements on the alloys concerned on the other hand, which necessarily result from the use of higher scrap inputs in the melting of alloys, have, however, one basic scientific theme in common: this is the fundamental question of how the occurrence of a variety of impurity elements can affect the properties of the alloys made from scrap. To this end, it is necessary to consider effects that have received little attention to date, such as those resulting from phase formation in high-dimensional phase diagrams. If a metal contains a large amount of unwanted tramp elements, it is more likely to form intermetallic and multiple metastable phases or to change precipitation phases unfavorably. This can then have a negative effect and change for example embrittlement and corrosion response. The basic research field behind these challenges associated with such multi-element scrap-related impurity effects has been termed the “science of dirty alloys”.^7^

The topic is particularly important because the internationally available amount of “old” scrap (which refers to highly contaminated post-consumer scrap) grows much faster for some of the metals in need than the chemically well sorted and less contaminated high-quality industry scrap.^391,392^ Hence, the research community must find out how metallic alloys can be made more robust against elemental contamination or how such impurities can be even turned into a benefit.

Another aspect in the early planning of the availability, quality, and impact of the use of metallic scrap is the rapid development of the products from which the scrap comes. Two aspects have to be taken into account here. On the one hand, this is the retention time of metals in the products, which can range from more than 100 years, e.g. in buildings, to a few days, e.g. in the case of packaging. This aspect is essential for the planning of new scrap sorting-, smelting-, and alloy-design methods, because there is sometimes a considerable delay before products return to a circular economy as new raw materials, Figure 75.

A specific example is the recycling of aluminum-silicon casting alloys that serve as large-volume combustion engines in about 1.6 billion vehicles around the globe. These materials are now gradually being returned to the material cycle as scrap. Yet, there is currently hardly a viable market for these alloys because of the worldwide transition from combustion to electric vehicles. In these new types of cars, large cast combustion engines and the corresponding alloys from which they are made are no longer needed.^7^

The second important aspect is the fact that due to the continuous development and performance improvement of many products, the metals and alloys previously used in them are not congruent with those used in the corresponding successor products. This often raises the question of whether certain recycled alloys can still be used at all for new or successor products. In a nutshell, this means that it must be taken into account that metallic alloys on the one hand return to the material cycle as “donors” for new materials but on the other hand also have to consume and recycle returning scrap which brings them into the position of an “acceptor” or “receiver” material. These two boundary conditions do not always fit together, because products evolve and continue to develop at a rapid pace, so that some scrap that returns to the market may no longer be needed in the corresponding successor product. In addition to the entropy-related losses, this is also a very important research and development challenge for the development of a corresponding circular economy. Ideal are thus fully closed loop metallurgical recycling approaches, where the same alloy is used as donor and acceptor; i.e., the scrap has the same chemical composition as the target alloy, and collection is in the best case sort-specific.

Another important consideration in metallurgical recycling is the distinction between source alloys and sink alloys.^393,394^ Source alloys are all materials (and—in the negative case—their mixtures) that return to production as scrap. Sink alloys are those alloys that are particularly suitable for using different or even slightly contaminated scrap as raw material. As a rule, these are alloys that are more “good-natured” with regard to the use of scrap. Specifically, in the case of metals, these are often casting alloys, which are in many cases more tolerant of the use of less well sorted scrap, whereas wrought alloys usually undergo considerable (local) deformation during production and react much more sensitively to variations in the chemical composition of scrap and the impurities it introduces.

In general, one can differentiate between closed-loop recycling and open-loop recycling.^395^ The former refers to any recycling approach, where no waste, except entropy-related losses, is produced. Open-loop recycling refers in contrast to a scenario where disposal is delayed by turning manufactured goods at their end of life again into new products, yet via dumping some of the material and replenishing (sweetening) the material from primary production.

In addition to composition-related classification, scrap is also grouped according to its physical size and dispersion. Typical criteria are bundling, shredder state, heavy melt parts, sheet, and plate, etc.

These general aspects related to scientific challenges associated with scrap as a feedstock can be filtered into a few specific research fields, also considering similar discussions from the literature,^67,396^Table 20.

**Table 20:** Categories, Research Opportunities, and Possible Scenarios Pertaining to Different Scrap-Related Challenges in the Metallurgical Sector

New scrap (sort-specific, recovered early on during manufacturing, pre-consumer scrap) vs old scrap (mixed and contaminated post-consumer scrap) vs re-mining (of dumped scrap, tailing, residue and waste sources)
Nanoscrap vs bulk scrap vs shredder scrap
High-quality recycling in multi-metal recovery from mixed scrap
Alloy-to-alloy recycling
Alloy design for maximum scrap use (maximum acceptor principle)
Alloy design concepts for suited source and sink alloy groups, receiver alloys, donor alloys, acceptor alloys
Runaround scrap, pre-consumer scrap, e.g. from stamping, blanking, etc.
Closed loop recycling (same alloy-to-alloy-specific recycling) vs open loop (non-alloy-specific reuse of scrap)
Scrap-oriented alloy design, to make alloys more resilient against scrap-related impurity intrusion
Alloy design for maximum compatibility to needed alloys (maximum donor principle)
Entropic losses of rare and precious elements “hidden” in complex products and nano-scaled parts
Consideration of “new” so far not-considered types of impurity elements coming from novel types of products when scraped (e.g., electrical vehicles, electronic scrap, electrified equipment etc.)
Novel electrochemical, hydrometallurgical, biometallurgical, and pyrometallurgical processes for treating mixed electrical and electronic scrap
Optimisation of metal recycling under increased quality constraints (effect of quality constraints on recycling rate)
Circular-economy-oriented alloy and product design
Degree of required dismantling vs processing vs shredding
Development of new products that specifically exploit the availability of inexpensive high-scrap containing alloys
High impurity content vs high safety; discrepancy between use of high-quality materials for optical appearance and safety in products and higher requirements to use more scrap
Materials with high recycling rates (can aluminum stock, stainless steels) and low recycling rates (catalysts)

#### 6.3.2 Role of Carbon Steel Scrap in Sustainable Metallurgy

**Figure 97:**
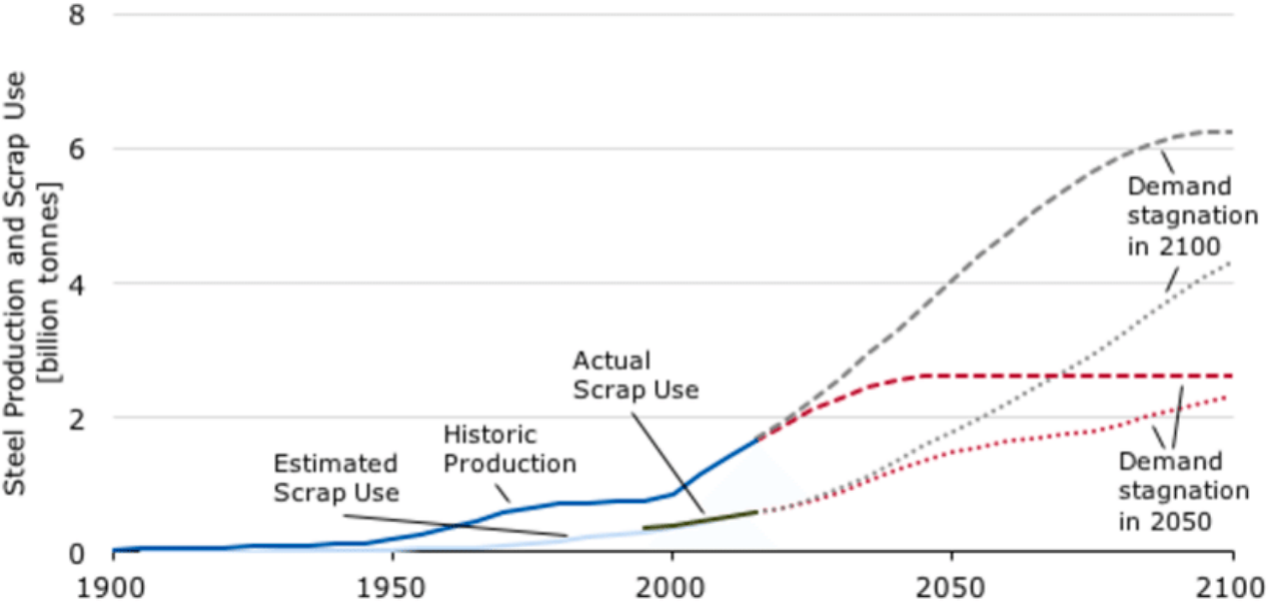
Steel production and scrap use, with projections for different saturation and stagnation scenarios.^399^ The figure is reproduced with permission from ref (399). Copyright 2015, Elsevier.

Every year, approximately 630 million tonnes of steel scrap are recycled globally, Figure 74. Remelting them into new steels (usually by using electric arc furnaces and as cooling scrap in oxygen converter furnaces) helps avoid approximately 950 million tonnes of CO_2_ emissions. This contribution from conventional recycling is currently the biggest single factor for the mitigation of global warming in the metallurgical sector that is already in place and must be further developed.^388,397−399^ Reducing CO_2_ emissions further along this direction requires us to increase the amount of scrap use for steel making, beyond the current global average of about one-third when averaged over all steel grades produced. This is a system-critical approach that will have the greatest and most immediate impact on improving metallurgical sustainability, Figure 97–Figure 99.

**Figure 98:**
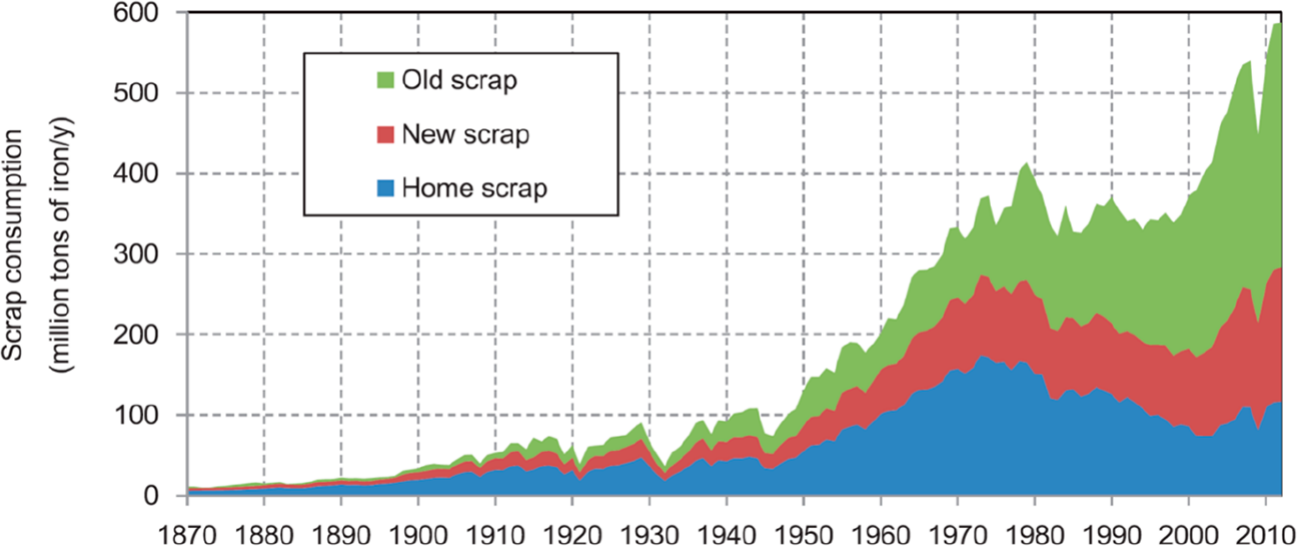
Current and past availability of steel scrap.^165^ Old scrap refers to mixed post-consumer scrap collected from end-of-life products. New scrap is generated along the manufacturing chain prior to use by an end customer. Home scrap is material which is internally generated at a company site during the production of the new steel. The figure is reproduced with permission from ref (165). Copyright 2013, Elsevier.

**Figure 99:**
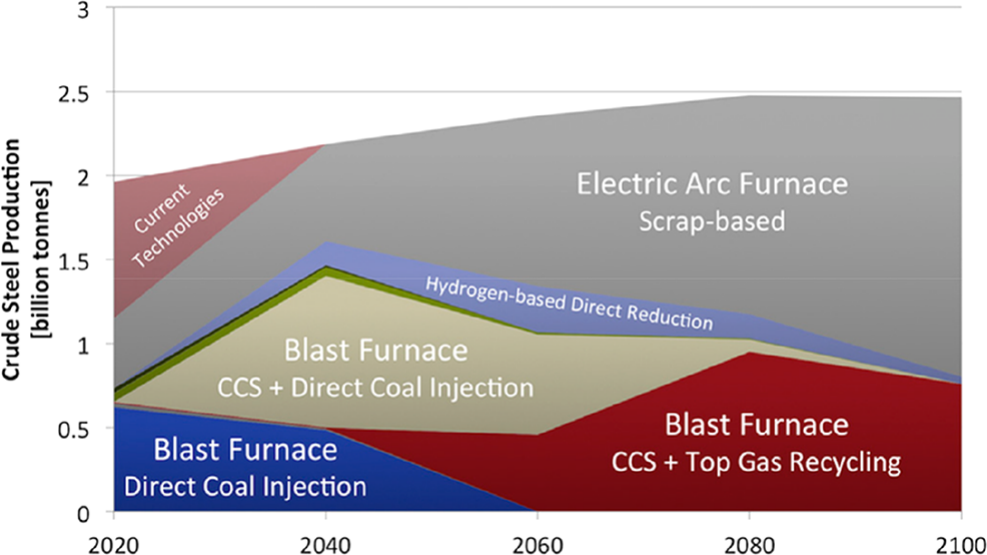
Projections of the expected shift in scrap melting, reduction smelting and other reduction techniques for oxide and steel, respectively, as a function of higher steel scrap use.^399^ One should note the assumed huge (yet realistic) future market demand scenario for steels. This analysis makes again clear that research in the field of sustainable metallurgy can potentially have a particularly high leverage when it comes to steel production, simply due to the huge amounts of metal produced. The figure is reproduced from ref (399) with permission. Copyright 2015, Elsevier.

However, there is a supply bottleneck due to demand growth. Scrap prices, which have traditionally followed iron ore and coking coal trends, are likely to diverge from the prices of other feedstock goods as a result of the decarbonization pressure and the importance of using more scrap to reduce CO_2_ emissions. This means that the price of carbon steel scrap will rise because recycled materials are much more environmentally friendly in terms of CO_2_ emissions and energy intensity. This will make carbon steel scrap in the future a highly attractive feedstock material in the metallurgical sector, irrespective of its long-term use in products, before it returns back to the market as scrap, Figure 75.

Although scrap has always been used as cooling material in oxygen converters (because of the exothermic reaction of carbon and oxygen into CO_2_ from the removal of the pig-iron carbon from the blast furnace with oxygen), the increased availability and use of scrap as feedstock are linearly connected with the increased use of electrical furnaces. The currently used electric arc furnaces (about 200 in the European Union alone) might in the future probably also see competition from other furnaces such as induction furnaces, due to higher electrical efficiency.

Besides CO_2_ and energy reduction, also the exploitation and efficient use of the alloying elements contained in the scrap is an essential potential benefit, in order to not oxidize and thus loose valuable alloying elements into the slag.^160,167,400^

The electric steel secondary synthesis process uses steel scrap and/or direct-reduced sponge iron from direct reduction as raw material(s) in an electric arc furnace. The charged material is melted down via graphite electrodes using electrical energy, which should ideally be of sustainable origin. The process is currently operated with up to 100% steel scrap, except for those plants that already operate methane-based direct reduction plants and use also the iron sponge (usually in compacted form) as additional feedstock. The effect on sustainability, when using scrap only, is considerable: compared to primary production via the blast furnace and converter route with its CO_2_ emissions of about 2 tonnes CO_2_ per tonne of crude steel, only about 0.3–0.5 tonnes CO_2_ per tonne of crude steel are emitted from the electric arc furnace route when 100% scrap input in used. However, globally, about 70% of the crude steel is still produced via the blast furnace route and only about 30% via the electric-arc-based scrap melting route. Since the latter approach saves up to 1.7 tonnes of CO_2_ per tonne of crude steel, steel production with scrap in electric arc furnaces can help to save up to 85% of CO_2_ emissions per year.^388,397−399^

It is also worth noting that the use of scrap as feedstock for secondary production of steels is also the fastest pathway for (sustainable) electrification of this sector.^49,387,401,402^ This means that while the blast furnace and converter route provides the required energy and heat through carbon-based exothermic chemical redox reactions with the oxides, the melting of scrap can be done to a large fraction by using electrical power, because no substantial chemical reactions take place. This means that the energy for melting scrap is essentially needed for vaporization of water and other organic substances, heating the scrap, and the phase transformation from the solid into the liquid phase, but no reduction takes place. This allows the use of sustainable electrical energy to operate the corresponding electric arc furnaces. This is an important side effect which leads to an altogether much higher efficiency of the secondary production route through scrap compared to most other chemically driven processes. Most of the classical primary production methods such as the operation of blast furnaces or of conventional direct reduction reactors cannot be electrified. This means that the use of scrap in conjunction with electric arc furnaces has a 2-fold positive impact on improved sustainability.

**Figure 100:**
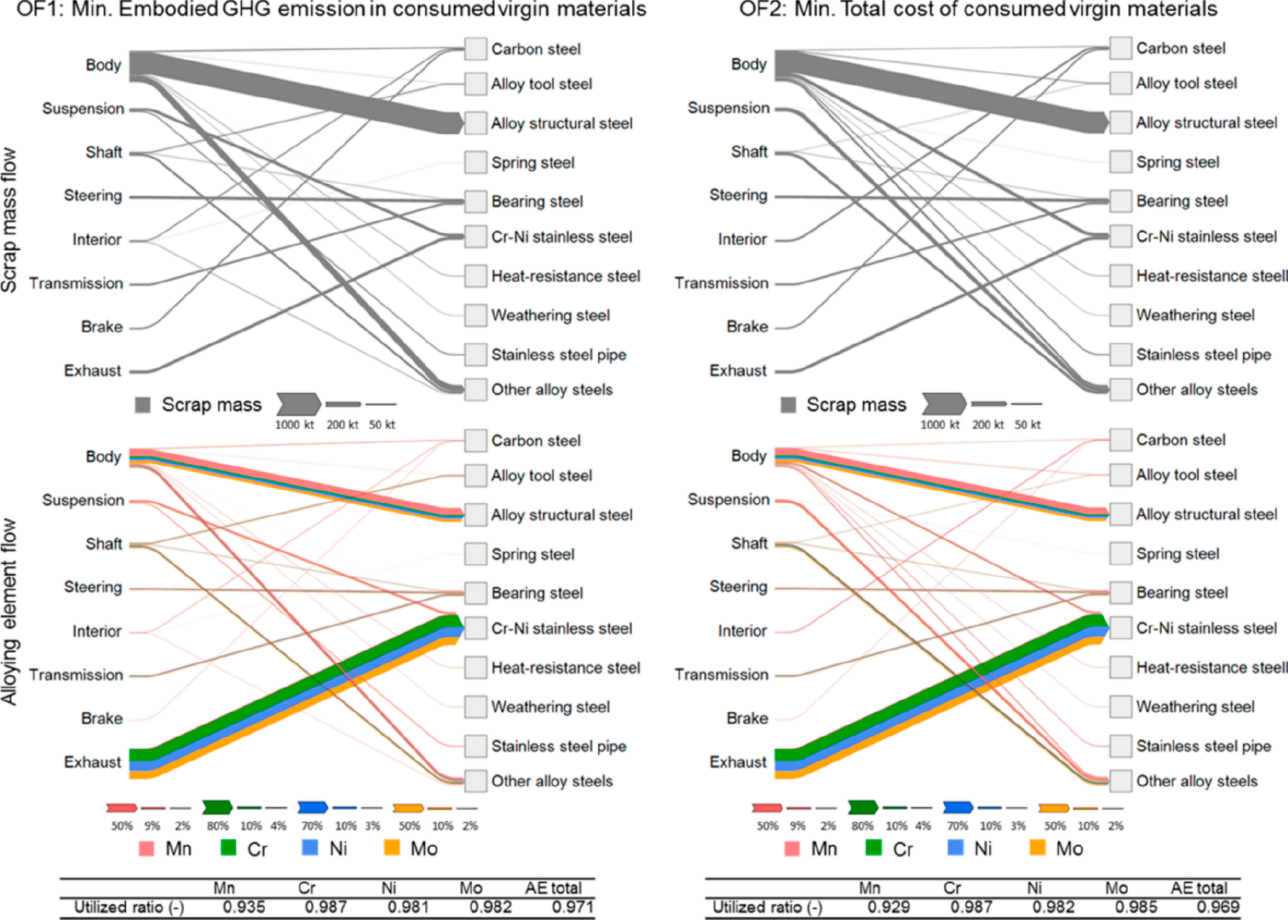
Sankey flow diagram for steel scrap, quantified by mass flow (upper half) and by alloying element flow (bottom half) using two objective functions (left half and right half) with demands for alloying elements in electric arc furnace (EAF) based steel production in Japan in 2005.^160^ The left-hand side and right-hand side of each diagram show scrap to be used for steel production by EAF melting, and alloy steel grades chosen to be produced by using parts scrap from 19 steel grades as well as from plain carbon steel, respectively. Steel grades not appearing in the figure were incompatible with available scrap sorts, due to significant mismatch between chemical contents and requirements for certain alloying elements.^160,167,376^ The figure is reproduced with permission from ref (160). Copyright 2017, American Chemical Society.

Figure 100 shows a study of Ohno et al.^160,167,376^ about optimized flow models for scrap in terms of mass (upper half) and in terms of alloying elements (bottom half) for the scrap-based electric arc furnace synthesis route. The main objective of this detailed study was the question of optimal steel scrap match to specific processing routes and to specific new steel grades. The data for these two objective functions (quantity of steel and demands for alloying elements) were taken for the steel industry in Japan in the year 2005. The two opposite sides of each Sankey diagram show separated scrap to be used for secondary steel production with electric arc furnaces and the specific alloy steel grades considered in this analysis.

The scrap cycle was also studied by the same author group, placing specific focus on end-of-life vehicle scrap. It was concluded that vehicle scrap plays an important role as urban mine feedstock with high and predictable growth rates. The reason for that is that customers in developing countries experience rising living standards and automobiles are increasingly made with high-quality materials to comply with increasingly stringent environmental and safety regulations. The majority of automotive materials, particularly steel, have been recycled at high rates in the past already. However, expensive alloying elements and elements from magnets and electrical and electronic components in vehicles are usually nowadays not recovered at all.

Ohno’s study highlights the maximal potential of quality-oriented recycling of end-of-life vehicle steel, by exploring the utilization methods of scrap, sorted by parts, to produce electric-arc-furnace-based crude alloy steel with minimal losses of alloying elements. Using linear programming, he observed that adoption of parts-based scrap sorting could result in the recovery of around 94–98% of the alloying elements occurring in these parts and hence also in the corresponding scrap (manganese, chromium, nickel, and molybdenum), an approach which could be the basis to replace 10% of the virgin sources in electric-arc-furnace-based crude alloy steel production.

Today, the steel recycling industry supplies the steel industry usually with up to 20 different types of bulk carbon steel scrap, Table 21. Some typical metallic contaminant elements in the carbon scrap deserve particular mentioning, owing to the substantial potential influence on the quality and properties of the recycled steels.

**Table 21:** European Specifications for Steel Scrap^a^

Scrap category	Properties
Old scrap	Specification E1: Post-consumer thin steel (<6 mm thickness) cut to dimensions and prepared in a manner for direct furnace charging. Such scrap can include vehicle wheels, but no vehicle body scrap and no domestic appliances. Must be free of rebars and merchant bars, free of metallic copper, tin, lead and their alloys. The aimed analytical residual contents are copper < 0.25 wt %; tin < 0.01 wt %; ∑Cr,Ni,Mo < 0.25.
	Specification E3: Post-consumer thick steel scrap (>6 mm thickness), cut to dimensions and prepared in a manner for direct furnace charging. This scrap can include tubes and hollow sections. Excludes vehicle body scrap and wheels from light vehicles. Must be free of rebars and merchant bars, free of metallic copper, tin, lead and their alloys. The aimed analytical residual contents are copper < 0.40 wt %; tin < 0.02 wt %; ∑Cr,Ni,Mo < 0.30 wt %.
New scrap (low residuals, uncoated)	Specification E2: Thick new production steel scrap predominantly more than 3 mm thick prepared in a manner to ensure direct charging. The steel scrap must be uncoated unless permitted by joint agreement and be free of rebars and merchant bars even from new production. Must be free of metallic copper, tin, lead and their alloys. The aimed analytical residual content is <0.300 wt % over all impurity elements combined.
	Specification E8: Thin new production steel scrap predominantly less than 3 mm thick prepared in a manner to ensure direct charging. The steel scrap must be uncoated unless permitted by joint agreement and be free of unbound ribbons to avoid trouble when charging. Must be free of metallic copper, tin, lead and their alloys.
	Specification E6: New production thin steel scrap (less than 3 mm thick) compressed or firmly baled in a manner to ensure direct charging. The steel scrap must be uncoated unless permitted by joint agreement. Must be free of metallic copper, tin, lead and their alloys. The aimed analytical residual content is <0.300 wt % over all impurity elements combined.
Shredded	Shredded steel scrap: Old steel scrap fragmentized into pieces not exceeding 200 mm in any direction for 95% of the load. No piece, in the remaining 5%, shall exceed 1000 mm. Should be prepared for direct charging. The scrap shall be free of excessive moisture, loose cast iron and incinerator material (especially tin cans). Must be free of metallic copper, tin, lead.
Steel turnings	Specification E5H: Homogeneous lots of carbon steel turnings of known origin, free from excessive bushiness. Should be prepared in a manner to ensure direct charging. Turnings from Free Turning Steel must be clearly identified. The turnings must be free from all contaminants such as nun ferrous metals, scale, grinding dust and heavily oxidized turnings or other materials from chemical industries. Prior chemical analysis could be required.
	Specification E5M: Mixed lots of carbon steel turnings, free from excessive bushy and free from turnings from Free Cutting Steel. Should be prepared in a manner to ensure direct charging. The turnings must be free from all contaminants such as nonferrous metals, scale, grinding dust and heavily oxidized turnings or other materials from chemical industries.
High residual scrap	Specification EHRB: Old and new steel scrap consisting mainly of rebars and merchant bars prepared in a manner to ensure direct charging. May be cut, sheared or baled and must be free of excessive concrete or other construction material. Must be free of metallic copper, tin, lead and their alloys.
	Specification EHRM: Old and new mechanical pieces and components not accepted in the other grades prepared in a manner to ensure direct charging. May include cast iron pieces (mainly the housing of the mechanical components). Must be free of metallic copper, tin, lead and alloyed parts such as bearing shell and bronze rings.
Fragmentized scrap from incineration	Fragmentized incinerator scrap: Loose steel scrap processed through an incinerating plant for household waste followed by magnetic separation, fragmentized into pieces not exceeding 200 mm in any direction and consisting partly of tin coated steel cans. Should be prepared in a manner to ensure direct charging. The scrap shall be free of excessive moisture and rust. Must be free of excessive metallic copper, tin, lead and their alloys.

a Note that this table is a shortened version of the original specifications descriptions, which contain more details.

As discussed below in more detail, standard carbon steel scrap must be particularly free of metallic copper and its alloys (see sections 6.3.4 and 7.4.8). Examples of products through which copper enters into mixed steel scrap are for instance solders, cables, electric motors, copper coated materials, radiator cores, and tubings.^10,76,403^ Also, tin must be omitted from scrap. This element enters typically through tin cans, tin coated materials, etc. as well as through bronze products. Likewise, lead which can enter scrap via batteries, solders, and wheel weights must be removed prior to smelting. These elements can all lead to the formation of low-melting phases at the grain boundaries which can lead to catastrophic embrittlement of the final product.

More specifically, copper, lead, and tin can accumulate beneath the scale layer. Already at moderate homologous temperatures they diffuse along the lattice defects and start to decorate particularly grain boundaries and other internal interfaces, driven by the adsorption isotherm. Once decorated, these regions can trigger formation of low-melting phases which can lead to severe liquid embrittlement under tensile, shear, and bending loads. Also, higher amounts of chromium, nickel, manganese and molybdenum contained in scrap must be omitted in carbon steel scrap, as they affect phase transformation, precipitation and hardening mechanisms. The magnetic response of the scrap is usually used as a measure for scrap sorting in that context. Opportunities for sustainability-related research in scrap-related steel synthesis are listed in Table 22.

**Table 22:** Opportunities of Research Tasks Related to the Use of Steel Scrap as Feedstock for Sustainable Secondary Synthesis (Green Steel Making).

Higher fraction of cooling scrap in converters
Carbon-free inert electrodes for electric arc furnaces
Removal of critical and difficult-to-remove elements that are introduced from mixed vehicle scrap and negatively change the steel quality, particularly causing liquid metal embrittlement (e.g., copper, lead, tin)
Introduction of new sorting methods to ensure that medium-manganese steel scraps are separated from iron-carbon steel scrap
Development of more efficient electric furnace technologies and/or alternative electrical melting techniques for scrap
Part-to-part closed-loop recycling pathways, to guide the flow of valuable alloying elements
Removal of elements that affect phase transformation and hardening such as nickel, chromium and molybdenum from scrap used for carbon steels
Plasma-based removal of tramp elements such as copper, lead and tin
Build-up of closed loop sorting for manganese-containing steels as feedstock platform for the sustainable production of medium-manganese high-performance steels
Steel alloy design for higher impurity tolerance; development of copper-containing steels (e.g., weathering steels, silicon transformer steels, manganese-containing stainless steels and Invar alloys etc.)

#### 6.3.3 Highly Alloyed Iron-Manganese-Based Steel Scrap

Another aspect concerns the introduction of high-manganese and medium-manganese steels,^170,172,404−406^ whereby the latter class of materials in particular will gain in importance in the future but will also introduce significantly higher manganese contents into the previously rather manganese-poor iron-carbon scrap.^166,170^ The new class of medium-manganese high-strength steels refers to alloys with an enhanced manganese content ranging between 3 wt % and 15 wt %.^407−409^ The maturation of these materials is driven by the demand for compositionally lean yet very strong and formable steels for the automotive sector.^170,172^ These steels differ from other steel groups regarding chemical composition and microstructure features, exploiting a huge microstructure variety in terms of phases and microstructure constituents including (metastable) austenite, ferrite, martensite, bainite, pearlite, and carbides, with a wide tunability of their size, dispersion, morphology, distribution and percolation.^166^ The interplay of these features allows us to exploit many kinds of strengthening and strain hardening mechanisms in versatile combinations. The cosmos of mechanisms includes solid solution strengthening, interface strengthening, precipitation strengthening, transformation-induced plasticity (TRIP), twinning-induced plasticity (TWIP), dislocation strengthening, dynamic strain aging and multiphase composite strengthening. These properties and their microstructure-based design render them a very attractive alloy class with lean composition and high sustainability.^407,410,411^ Producing such medium-manganese steels in the future primarily through scrap would make them an ideal material class for the automotive sector, with very high sustainability, as the properties are mainly obtained through a smart microstructure design rather than through overalloying with expensive and harmful or rare and expensive elements.

In this context, however, it must be taken into account that there are not yet sufficient quantities of suitable iron-manganese scrap in the desired compositions for this attractive class of materials on the world market. On the other hand, the specific advantage of this alloy class is that its basic alloy composition, apart from the manganese, is relatively close to that of ordinary iron-carbon steels.

This means that there is a coming opportunity to produce these materials by electric arc furnaces with a very high scrap content from the classical iron-carbon scrap route and then to alloy them in the secondary ladle metallurgy with pre-alloyed iron-manganese material from the primary synthesis. This approach would combine two sustainability advantages, namely on the one hand the advantage that the outstanding properties of these materials are essentially determined by their complex microstructure tailored to the application and not by the chemical composition and on the other hand the huge advantage that these materials could then also be produced with a very high scrap content.

In this context, however, it must also be taken into account that in improving the sustainability of high-performance materials, it is desirable not only to use the highest possible proportion of scrap but also to ensure that the materials have good donor properties. The latter aspect means that the same materials, if they are scrapped, should also be used again in new alloys. However, this also means that a separate sorting track would have to be opened for such medium-manganese steels, as these materials must not be mixed with conventional carbon steels and also not with high-alloy stainless steels. This means that to the current two large scrap groups for steel (carbon steels, stainless steels), a third one will have to be added, namely one for medium-manganese steels.

#### 6.3.4 Roles of Specific Scrap-Related Impurity Elements in Steels

Particularly old scrap has varying composition and can frequently contain harmful tramp elements. Especially elements such as copper, nickel, chromium, molybdenum and tin can accumulate to high fractions in old scrap and affect casting, hot and cold cracking, and corrosion if not removed or diluted.^76,403,412^

**Figure 101:**
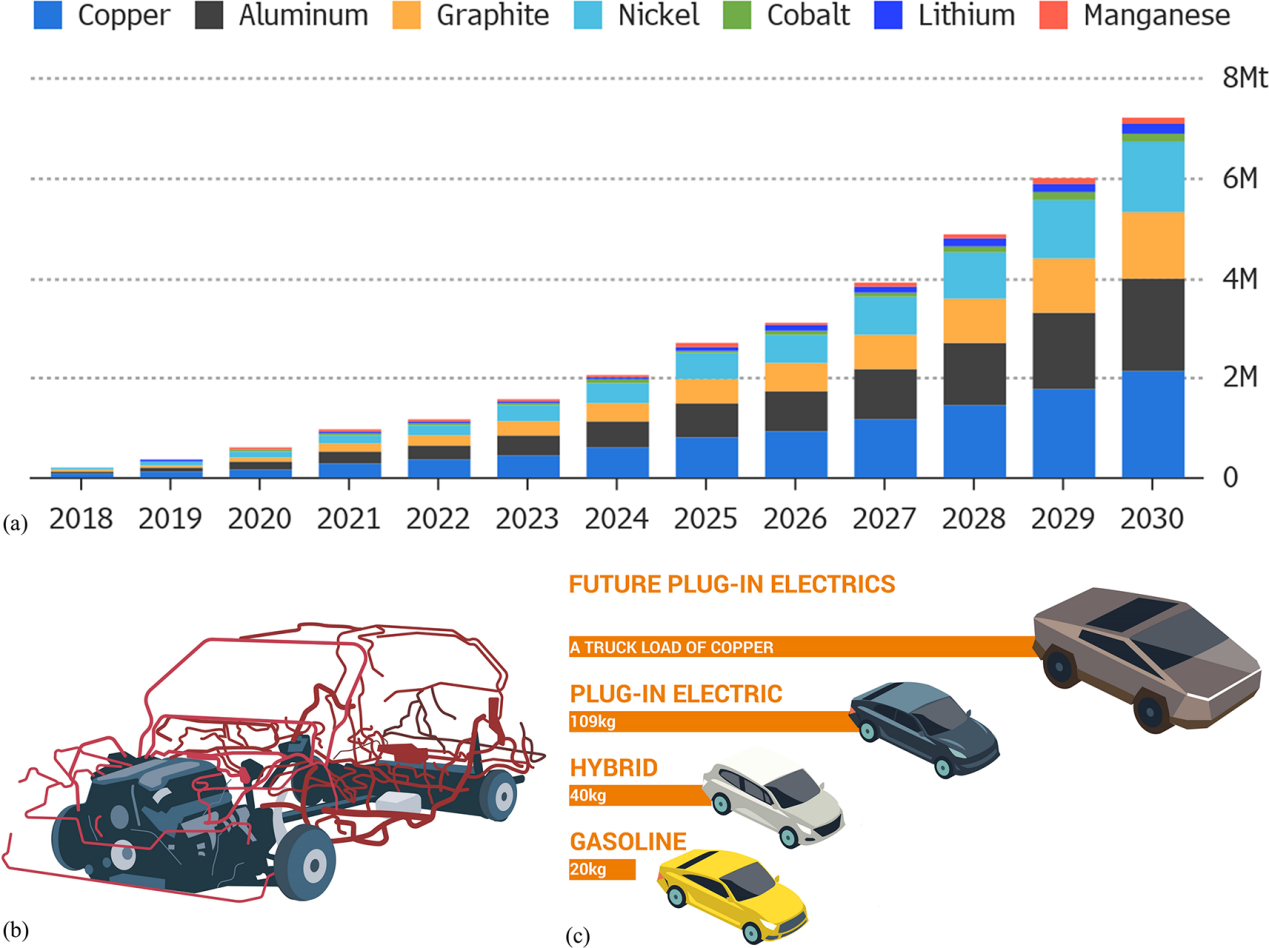
(a) Projected increase in the amount of several elements used in vehicles in million tonnes (Mt) per year, due to the growing electrification of the transport sector (data taken from Bloomberg). (b, c) Increase in the amount of copper in mixed vehicle scrap, due to the switch from combustion to electrical vehicles. The figures are reproduced in modified form with permission from BHP (c) and Bentley (b). Copyright 2022, BHP and Bentley.

**Figure 102:**
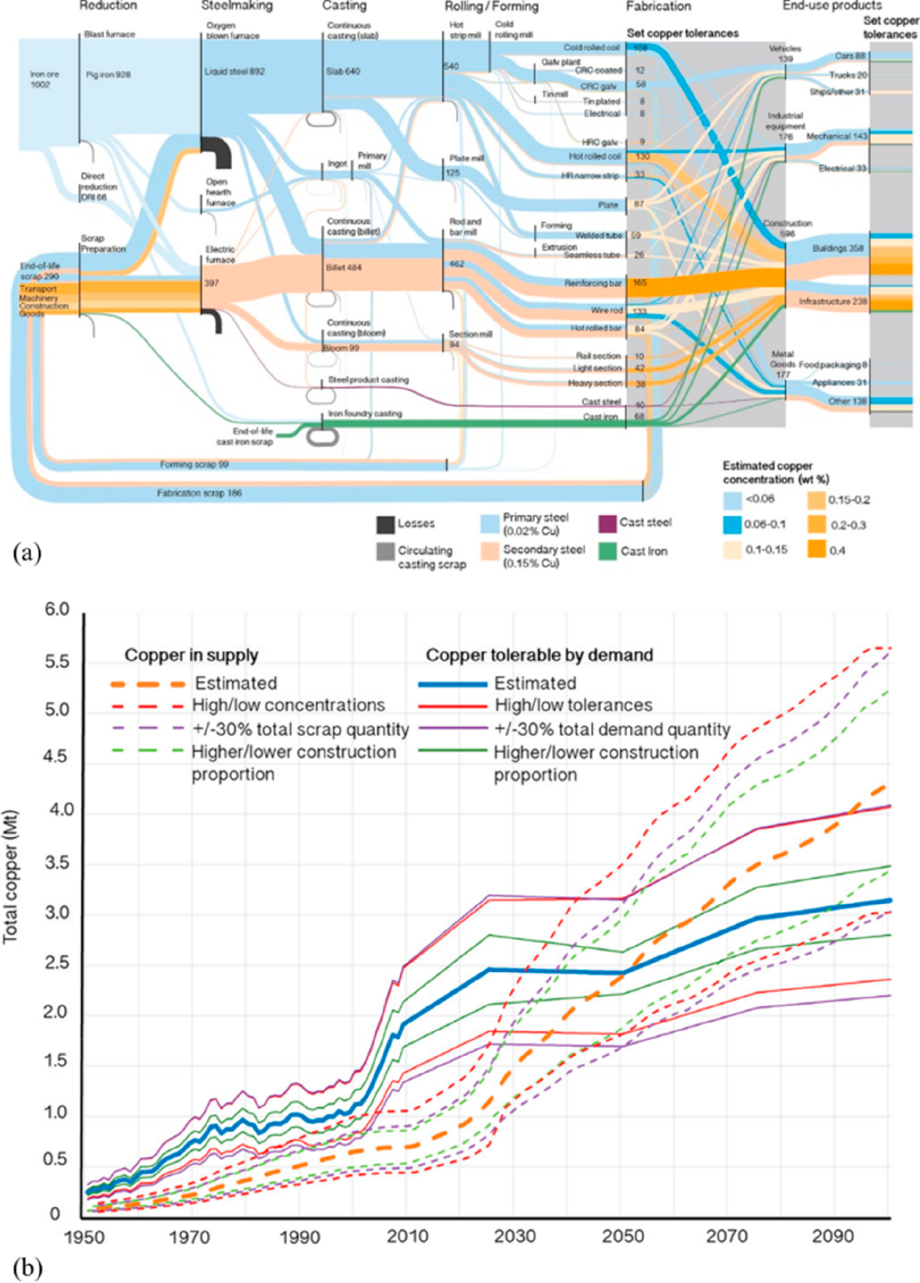
(a) Daehn et al.^10^ developed a Sankey diagram for the global steel flows. The data are plotted in million tonnes, considering the estimated copper concentrations. The gray zones contain copper tolerance limits of intermediate products and of end-use products with the aim to compare copper in the system versus maximum tolerable amounts. (b) Amount of copper in the end-of-life scrap supply and copper tolerable by demanded products from 1950 to 2100. The figures are reproduced with permission from ref (10). Copyright 2017, under a Creative Commons Attribution (CC-BY) License.

Copper and tin are both potentially harmful tramp elements that can enter the steel scrap streams through the growing use of these element in electrified vehicles, in solderings, and generally through the increasing electrification of many products and processes, Figure 101 and Figure 102.

**Figure 103:**
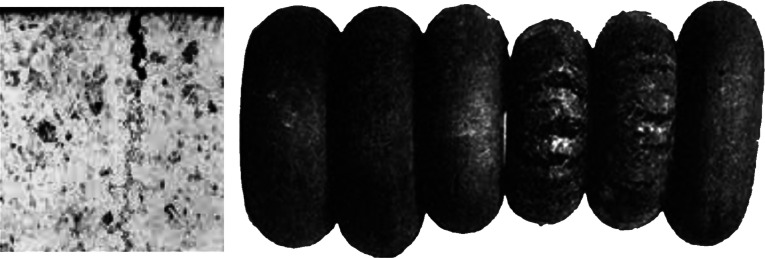
Increased amounts of copper, tin and lead in steels can lead to liquid-metal embrittlement through the formation of low-melting eutectics on decorated grain boundaries. The image shows a specific example of the effects of copper on the embrittlement of steel.^413^ The figure is reproduced with permission from ref (413). Copyright 1931, Verlag Stahleisen GmbH.

Many of these elements with high segregation coefficients to grain boundaries and moderate or low melting points such as lead, tin, and copper can cause a set of damage phenomena in steels that are all characterized by grain boundary weakening, formation of soft and/or low-melting phases at interfaces, and the associated intercrystalline fracture at higher temperatures such as experienced during hot rolling and under bending and tensile loading conditions, Figure 103. The underlying mechanisms can be related either to grain boundary decohesion effects or to liquid metal embrittlement.

While in the past such metals intruded into the steels through joining processes such as soldering, they are nowadays accumulating rapidly through their content in mixed steels scrap, Figure 102. For the case of copper this effect occurs already at such low concentrations as 0.1 wt %. Tin has a similar effect, yet already at concentrations as low as 0.04 wt %.

Slightly higher copper contents are permissible for long products such as beams, structural steel or rails. Here too, however, the upper limit for the copper content is usually less than 0.4% by weight.

One circumstance that makes the separation of copper from steel particularly problematic is the fact that, especially in shredding processes, the copper is often cold forged and welded together with the iron and can therefore hardly be separated in the solid phase by manual sorting.

Daehn et al. explored several strategies to remove copper from steels,^76^Figure 102. They proposed a framework for defining potential separation routes and evaluating their ability to remove copper, as well as estimating energy and material input requirements while taking into account the thermodynamic, kinetic, and technological constraints of the various methods to reduce copper in steels to a range below 0.1 wt %. The authors suggested that by using a reactor that minimizes radiation heat losses, vacuum distillation would be a possible approach. They further concluded that—although high-temperature solid scrap pretreatments would use less energy than melt treatments—their effectiveness with normal shredded scrap has yet to be proven.

Further tramp elements that can intrude through scrap or lubricants are phosphorus, sulfur, aluminium, silicon, manganese, and carbon, and these elements must usually be removed as far as possible prior to the subsequent ladle metallurgy. These refining operations depend on the partial pressure of (injected) oxygen. The oxygen preferentially reacts with most of these elements to form oxides which then partition into the slag. The oxygen also forms CO_2_ with the carbon. This latter aspect is particularly important when using a high fraction of methane-based direct reduced iron for diluting polluted scrap-based melts, as the material from direct reduction has a different carbon content than the pig iron from blast furnaces.

The partitioning of phosphorus into the slag depends on temperature, slag basicity (i.e., the slag’s CaO content), and the FeO content of the slag. An increased slag basicity (i.e., a high CaO level) promotes phosphorus removal, but saturation of the slag with CaO leads to an increase in slag viscosity. Sulfur partitions mainly as a sulfide into the slag. The oxygen also transforms aluminum, silicon and manganese into metallic oxides which then partition into the slag.

In the case that some of these tramp elements become too much enriched in scrap-based steels, high-quality iron for dilution can be also charged not only in the form of new scrap (with better controlled chemical composition) but also in the form of direct reduced iron which currently enters the markets for instance as hot briquetted iron. Regarding sustainability particularly, the use of hot briquetted iron produced via hydrogen-based direct reduction charged together with scrap into electric arc furnaces is a promising and relatively fast to realize low-carbon route (compared to other green steel concepts) for sustainable steel making.

A very interesting aspect in this context is also the property of iron as an energy carrier of very high energy density, compared to hydrogen or methane. This means that in the future it could be a very worthwhile scenario for the global workflows in sustainable metallurgy not to transport the reducing agents, such as hydrogen or natural gas, transcontinentally but instead to transport the metal directly reduced on site due to its much higher energy density. Reduced metals are, so to speak, the most efficient energy carriers and can be conveniently converted back into liquid alloys at the point of need via induction or electric arc furnaces, ideally by using renewable electrical energy.

In summary some opportunities for sustainability-related research in this field of the inheritance of tramp elements through steel scrap melting are listed in the next section.

#### 6.3.5 Stainless Steel Scrap

**Figure 104:**
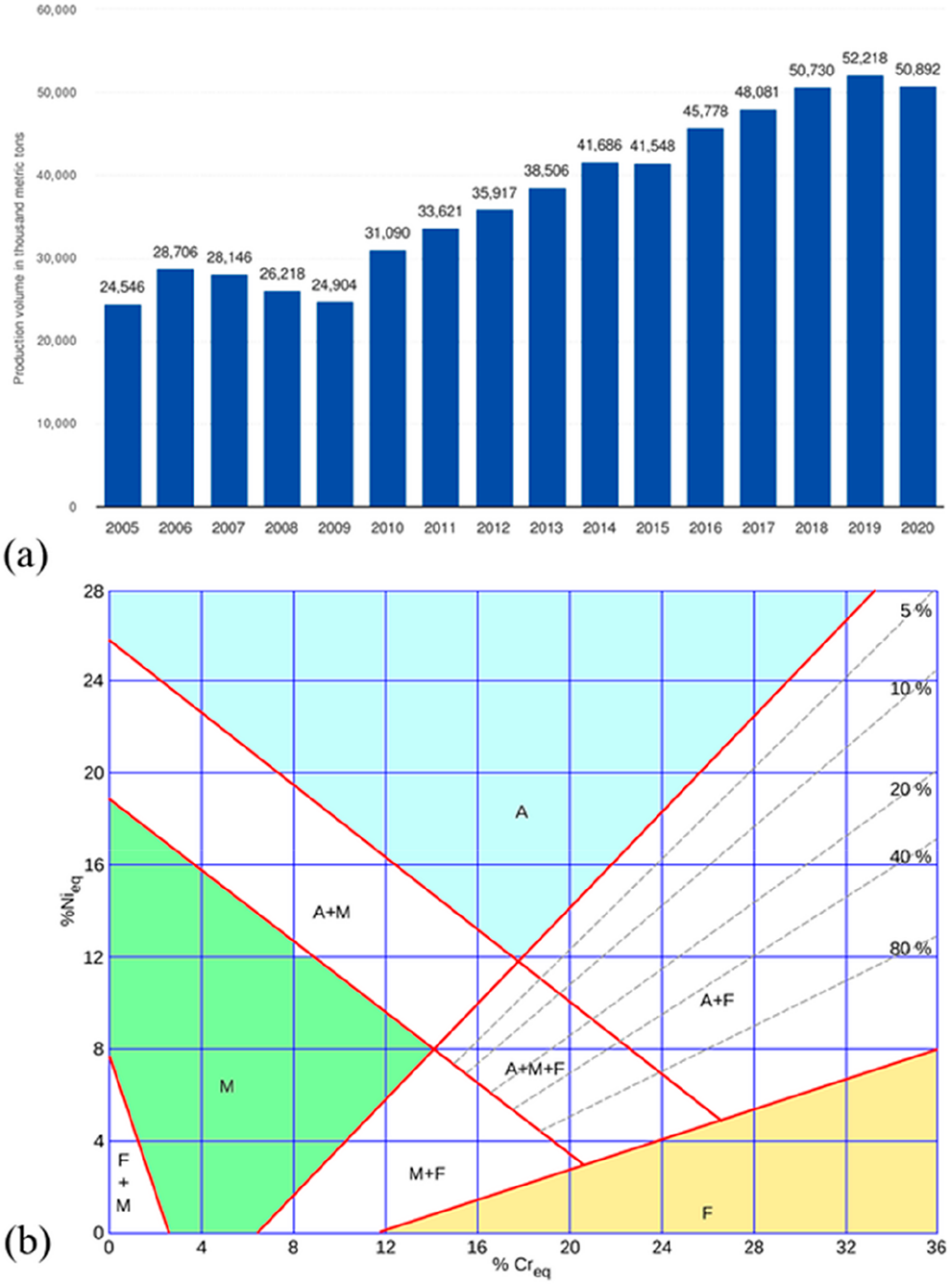
(a) Global demand for stainless steel in terms of melt shop production numbers in units of thousand metric tonnes (data from the International Stainless Steel Forum). (b) Different types of stainless steels, arranged in a nickel-equivalent (austenite stabilizers) versus chromium (ferrite stabilizers) equivalent map. Such maps are referred to as Strauss–Maurer or respectively as Schäffler diagrams. A, austenite; F, ferrite; M, martensite. The fraction numbers inside of the diagram indicate specific stainless steel grades with different chemical compositions, particularly regarding the nickel, chromium, and carbon content, that have not a single phase/single microstructure state but a mixed phase state. Figure (a) is reproduced with permission from the International Stainless Steel Forum. Copyright 2020, International Stainless Steel Forum.

The energy and commercial value embodied in stainless steels are huge, due to the use of expensive and critical metals such as nickel, molybdenum, and chromium, Figure 104. Due to the resulting high value of end-of-life stainless steel items, the recycling rate for stainless steel is exceptionally high. The greenhouse gas savings in the production of stainless steels based on the secondary raw material stainless steel scrap via the electric steel route are thus significant, due to the recycling not only of the iron itself but particularly also of the alloying elements nickel, chromium and molybdenum. This translates to savings of approximately 4.5 tonnes of CO_2_ per tonne of stainless steel scrap depending on the input material and the specific steel composition. Stainless steels are characterized by very high recycling rates: more than 90% of the material is returned at the end of its service life, according to data of the International Stainless Steel Forum (ISSF). Almost 100% of the production waste is recycled. Scrap collection of stainless steels requires, similar as for aluminum alloys, a well specified chemical-metallurgical analysis and alloy separation. Some research topics are shown in Table 22.

#### 6.3.6 Aluminum Alloy Scrap

Due to the low melting point of aluminum (660 °C, and only moderately different for its alloys), the energy required for melting is only about 5% of that required for primary production. This qualifies secondary aluminum synthesis via use of scrap as the most effective and rapid pathway toward a more sustainable metallurgy of the aluminum sector,^2,7,9,54,101,392^Figure 72.

Market projections show that the amount of aluminum scrap will grow as enormously as that of steel scrap, reaching a level in which up to two-thirds of the new aluminum could be theoretically made from scrap by 2050, Figure 74. However, it should be noted that this is an average value taken over all the different aluminum alloys. This is an important consideration because aluminum alloys are typically not readily mutually miscible without significantly affecting the properties of the final material. Therefore, alloy-specific data about scrap availability should be instead collected and evaluated when estimating realistic measures of the future scrap fraction (and its price development) that can be used to make aluminum. Bulk wrought alloys of the most important alloy groups 3xxx, 5xxx, 6xxx, and 7xxx used for packaging and transportation require special attention in that context (3xxx, Al-Mn; 5xxx, Al-Mg; 6xxx, Al-Mg-Si; 7xxx, Al-Zn-Cu-Mg).

**Figure 105:**
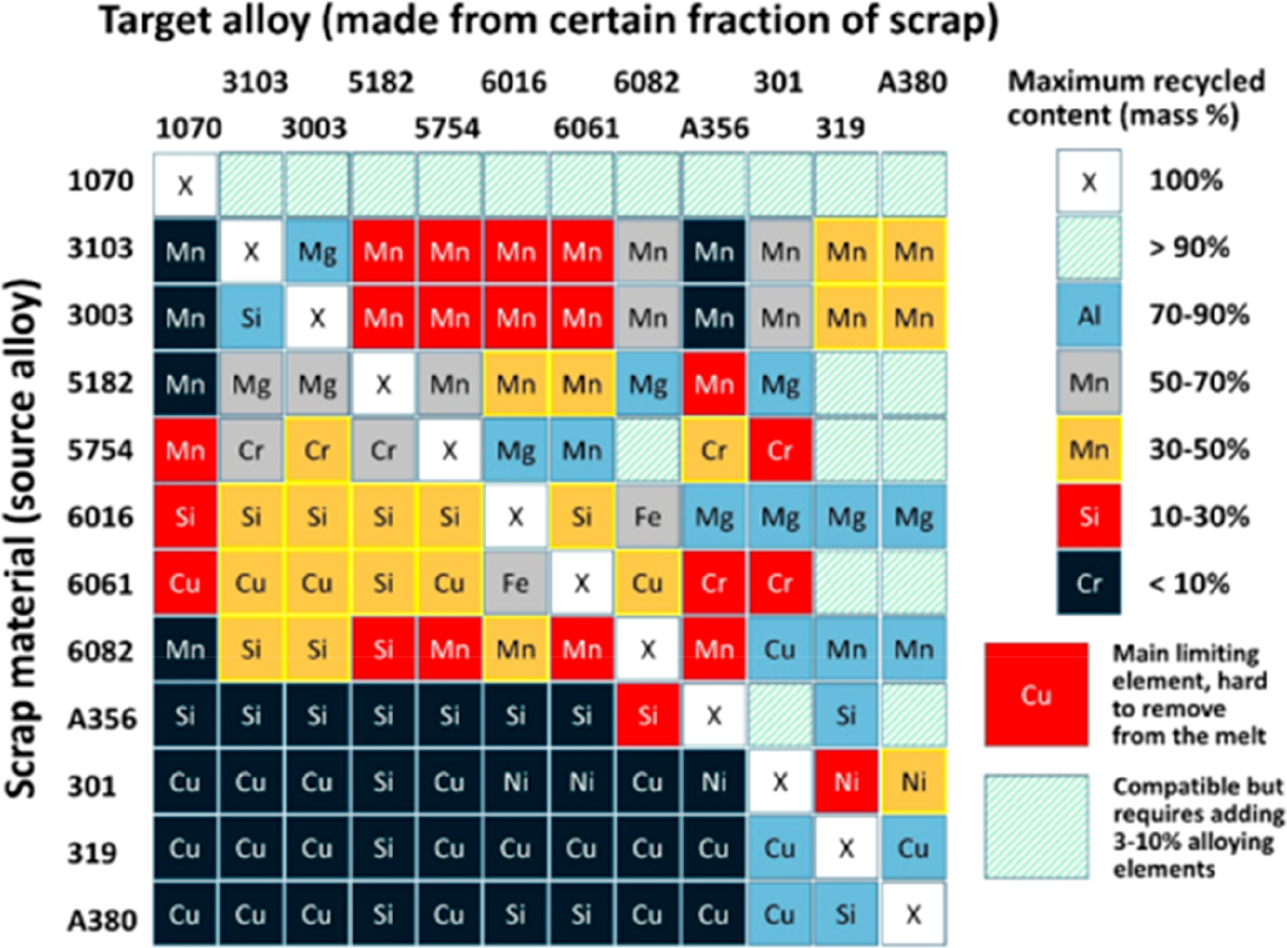
Recycling compatibility among aluminum scrap alloys (donator material) and target alloys (acceptor material) for the case of automotive applications.^7^ Many Al-Si alloys for making cast products are more tolerant regarding high scrap usage than alloys designed for sheet forming variants such as the Al-Mg and Al-Mg-Si systems, which are more sensitive to the intrusion of scrap-related impurities. Alloys A380, 319, 301 and A356 are materials for cast products. The other alloy classes are subjected to extrusion and foil and sheet forming operations (6xxx, age hardenable Al-Mg-Si alloy system; 5xxx, solid solution Al-Mg alloy system; 3xxx, solid solution Al-Mn alloy system; 1xxx, commercial purity alloys with >99 wt % Al). The figure is reproduced with permission from ref (7) under a 4.0 International Creative Commons license (CC BY 4.0). Copyright 2022, Elsevier.

It is important to consider in that context that the majority of the aluminum scrap returns to the market as low-quality, contaminated post-consumer scrap, Figure 74 and Figure 105. This means that it is not provided to aluminum melt shops as well-sorted pre-consumer scrap (collected in manufacturing) that can be readily remelted into alloys of similar composition as shown in Figure 105.

**Figure 106:**
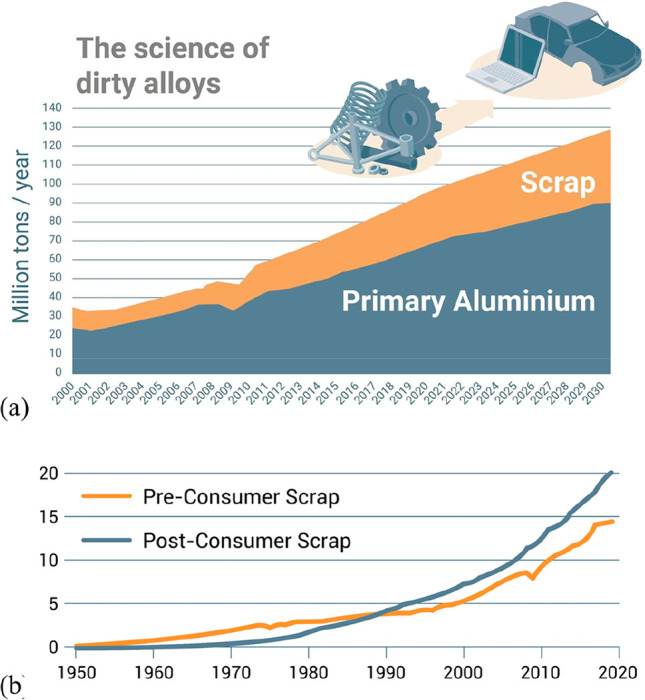
(a) Global growth of the aluminum market, showing both the primary and secondary markets’ fractions. (b) Fraction of new aluminum scrap (sorted alloy-specific scrap, collected during production and downstream manufacturing) and old aluminum scrap (mixed, post-consumer scrap).^7^ The figure is reproduced with permission from ref (7) under a 4.0 International Creative Commons license (CC BY 4.0). Copyright 2022, Elsevier.

This trend is likely to intensify in the coming decades, as the majority of the future aluminum scrap will be old scrap, i.e. impurity contaminated post-consumer material, Figure 106. This means that the return of compositionally mixed scrap is an increasing problem for the production of high-performance alloys.^7,11,265,378^ While large amounts of scrap are being returned to the market, the question of how mixed and contaminated scrap can be used in new alloys is thus becoming a more pressing research issue. This means that if this problem is not resolved, the huge potential savings in primary energy consumption in this sector from the use of scrap cannot be fully exploited, Figure 106.

This becomes also clear from the fact that the collection and presorting of post-consumer aluminum scrap are usually not oriented along the chemical classification scheme of the materials along the usual alloy classes, but rather along the product origin and the processing form. In most cases, different classes of aluminum scrap are considered according to their product origin and collected separately. Examples are packaging material (mainly cans), flat sheet material, extrusion components, castings, wire, aluminum residues combined with polymers, etc.

Certainly, there is a certain fit with the chemical alloy classification. For example, cans usually consist of 3xxx alloys and castings usually have a high silicon content, but there are also undesirable mixtures, e.g. for the case of sheet materials which can belong to completely different alloy classes (such as 1xxx, 3xxx, 5xxx, 6xxx, and 7xxx), which should not be mixed when melting new alloys. Of course, this problem does not exist when collecting the scrap sort-specifically within the production lines. In this case, the scrap can in principle be collected and remelted in a sort-specific way.

The scenario of the compositional heterogeneity of the aluminum scrap market has triggered a more general new research direction which consists in reducing the number of chemically different alloys and in improving the chemical tolerance of aluminum alloys against variations in scrap-related tramp elements, often summarized as the science of “dirty” alloys.

More specific, this research direction considers a number of basic metallurgical questions, including the development of cross-over alloys which can probably act likewise as acceptor and donor alloys for a larger variety of scrap types and alloys, respectively; the development of more impurity-tolerant aluminum alloys, an approach described as “sustainability alloy design”; the modification of existing commercial aluminum alloys in a direction to modify possibly detrimental intermetallic phases by further doping the material in a way that they become less harmful; advanced and fully automated and machine learning enhanced scrap sorting methods; and the detailed investigation of the origin and justification of certain strict alloy specifications with respect to less strict specifications which would facilitate higher fractions of scrap in the secondary synthesis.

In general, it must be considered that most of the alloying elements in aluminum have very low solubility, a feature which makes aluminum alloys fundamentally different from steels. This means that compositionally lean aluminum alloys are preferable to overalloyed materials because they can be recycled in other alloy grades more easily.

Cross-over alloys, also referred to as uni-alloys by some authors, aim at combining several well-established aluminum alloy concepts in the form of new alloy variants that combine averaged composition concepts from different material classes.^414−416^ This mixed alloy approach is intended to result in less chemical sensitivity and in a reduction in the large number of aluminum alloy variants that are nowadays thus in use and return as scrap. The advantage of cross-over alloys is also demonstrated by their ability to adjust strength through microstructure manipulation. For example, alloy 5182 has a tensile strength range of 300 to 550 MPa in the cross-over variant, i.e. after modification with Zn, Cu, and Ag.

Further attractive research options in this field could for instance address the question of alloy-specific processing of such cross-over alloys and how promising precipitation features and hardening mechanisms from different alloy families can be combined. Such novel types of broad-band, multipurpose alloys must be designed to be universal rather than niche alloys. They should have a wide composition tolerance and application range to serve as scrap feedstock when recycled.

Some of the key questions related to the use of scrap in aluminum alloys are summarized in Table 23. They include fundamental studies about the effects that the different tramp elements intruding from the scrap have on the alloy properties, specifically on formability, optical appearance and corrosion.^7^ It is also important to study how scrap and impurity-tolerant alloys can be developed and which tramp elements matter the most and what the upper limits are for those.^7^

**Table 23:** Research Opportunities on the Role of Scrap for Sustainable Aluminium Production (Green Aluminium)

Influence of scrap-related tramp elements in aluminum alloys on the thermodynamics and kinetics of precipitation reactions and their mechanical and electrochemical effects
Effect of scrap-related impurities on interface decohesion, phase formation, precipitation-free zones, precipitation kinetics, precipitation-free zones around grain boundaries, surface finish, mechanical properties and corrosion
Quality of thermodynamic and kinetic databases for the study of impurity-related spinodal, metastable and intermetallic phases
Influence of scrap-related contaminant elements on vacancy formation enthalpy and mobility
Inoculant systems suited for scrap-contaminated cast alloys
Scrap-related impurity effects on cast aluminum microstructures. Adjustment of solidification, solutionizing and heat treatment processes to cope with the effects of contaminant elements and the associated intermetallic phases
Possibilities of adjusting processing parameters for higher scrap tolerance such as higher cooling rates
Design and processing of aluminum alloys with the highest possible scrap fractions, using low-quality scrap and scrap types which match only a few target alloys when recycled
Improved and fully automated scrap sorting, including automated chipping, spectroscopy and artificial-intelligence-assisted alloy detection, classification and sorting
In-production scrap collection, including alloy-specific scrap collection during synthesis and manufacturing
Post-consumer scrap use along the development of low-grade and composition-tolerant alloys
Development of products that can tolerate and accommodate higher scrap-related contaminant content in alloys
Design of scrap-tolerant alloys for high-scrap-related tramp element content
Development of alloys that use less harmful and critical alloying elements
Selection of alloying elements according to lowest energy consumption and greenhouse gas emissions.
Avoidance of rare and less-responsible elements as alloying ingredients
Recycling as part of the entire alloy design workflow. This includes to ensure that alloys and by-products can be collected and recycled so that recyclability becomes an integral aspect of alloy and process design, considering also optimal material recovery of auxiliaries, scrap and unintended by-products
Closed scrap collection loops in-house and with customers
Methods for the removal of alloy-specific harmful tramp elements from scrap melts
Development of cross-over alloys and generally of alloys with higher impurity robustness to act as better scrap acceptor materials and more composition-friendly scrap donor materials

#### 6.3.7 Copper-Containing Scrap

The big challenge in copper recovery from old scrap is that this metal is often used in a highly dispersed and dilute way in products; i.e., it is used as cable or brazing material within complex and often micro- or even nanostructured products such as integrated circuits.^71,134,135^ Also in automobiles it gets mixed with many other metals when the vehicle is scrapped. The situation is particularly extreme for copper in electrical and electronic components, where it occurs in highly integrated form in nanoscopic dimensions. This means that special attention must be placed on the efficient recycling of copper (and other precious metals in such devices) from miniaturized system devices and products. This challenge can be also referred to as recycling of nanoscrap and as multi- or polymetal recycling.^87,109,111,135,417^

The retrieval of copper via recycling must consider three main stages, namely, sorting processes, separation techniques, and the metallurgical recovery and its by-products.^135^

A very important aspect is the gradually decreasing global availability of new scrap, which has high purity, as it is directly produced during manufacturing, i.e. not mixed with post-consumer scrap. The reason for this trend is the improved manufacturing efficiency. This means that the focus on copper recycling is shifting its focus toward the collection, processing and recycling of old scrap, also referred to as end-of-life or post-consumer scrap, with much lower copper content and much higher impurity content.

**Figure 107:**
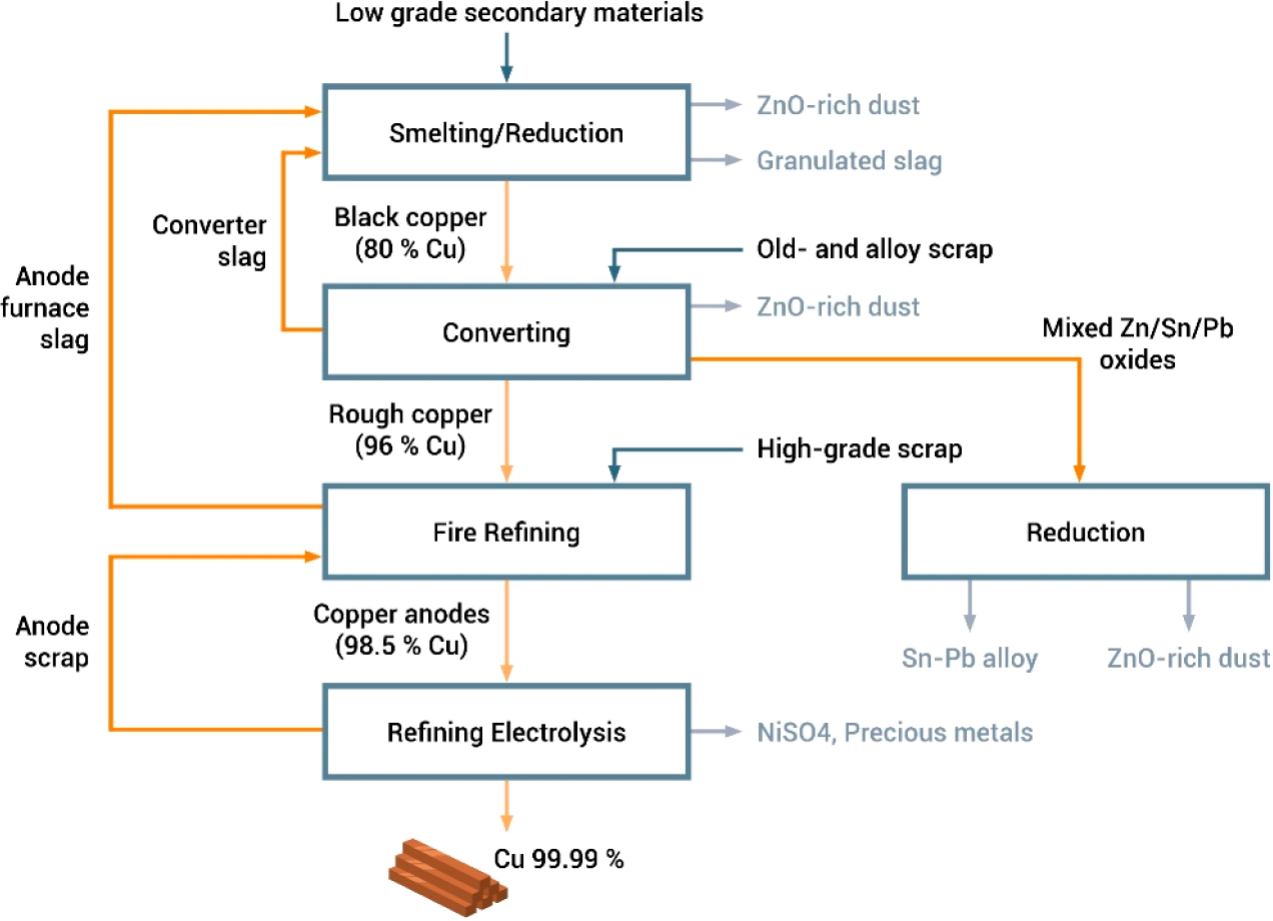
Different recycling pathways for the recovery of copper from residues of the copper industry.^418^

Copper scrap with high metal content can be recovered by conventional pyrometallurgical melting methods, for instance in induction or shaft furnaces. It can then be refined and processed into new products, Figure 107.

Slags, dusts, drosses, and sludges are examples of medium- and lower-grade copper scrap, Table 24. Higher-quality scrap comes from mixed alloy scrap or various types of shredder. Another form of scrap comes from waste electrical and electronic equipment, abbreviated “WEEE”. Using such resources is referred to as urban mining.

**Table 24:** Some Typical Scrap Types Used for Recovering Copper

Type of secondary material resource	Copper content (wt %)	Origin of copper scrap
Computer scrap	15–20	Electronic devices, consumer electronics
Ferrous copper material (lumpy or crushed) from fittings, stators and rotors	10–20	Electrical devices, vehicle amatures, electrical vehicle components
Industry slags from copper and brass production	10–40	Slags, dusts, drosses and sludges from foundries
Copper coolers	60–65	Vehicles
Industry copper scrap	<80%	Copper industry and downstream sheet and cable manufacturing

Most scrap materials can be divided into two categories: metallic and nonmetallic. Metallic residues often contain a high copper content and do not require complicated treatment.

Plastics are removed from copper using mass density sorting, and harmful companion metals such as iron are sorted out using magnets from secondary raw materials. Iron is a particularly undesirable tramp metal in this context as it increases the electrical resistivity of copper substantially.

Copper-rich alloy scrap, which primarily consists of brass and bronzes, is fed into secondary metallurgy, where the alloying elements mostly accumulate in the slag and flue dust.

Specific topics related to the recovery of copper and other metals from WEEE are discussed below in a separate section. General research topics related to copper scrap are summarized in Table 25.

**Table 25:** Metallurgical Research Opportunities Related to Sustainable Copper Production from Secondary Resources

Secondary synthesis and alloy design measures for scrap-tolerant copper production
Process and alloy adaption to cope with reduced availability of new scrap and increasing amount of old scrap, with high impurity content
Improved scrap sorting, including removal of harmful tramp elements such as iron, which substantially reduced conductivity
Alloy-specific scrap sorting, including sensor-and machine-learning enhanced scrap sorting
Science of “dirty” copper alloys, including effects of higher impurity element content on copper alloys
Multi-element and nanoscrap recycling, due to the nanoscale integrated use of copper (and other precious metals) in electronic devices
Copper alloy design and process design for better recycling

#### 6.3.8 Titanium Scrap: Recovery versus Downcycling

Titanium is a very durable material, but its recycling process is complex and expensive. Due to titanium’s high melting point, it can be difficult to separate out from other materials during recovery processes. At a global average approximately 80% of the titanium production is used in aerospace parts. Machining (instead of sheet forming or extrusion) is mostly used in the aerospace industry to avoid internal stresses. During the machining of titanium components, a large portion of the raw material is disposed of in the form of chips, which are produced by milling, turning, and grinding, to give workpieces the desired shape by removing excess material from raw parts without bending, extruding or welding. The latter types of processes, which are standard operations for aluminum and steel products in vehicle production for instance, can lead to undesired internal stresses, damage initiation, and oxidation in sensitive aerospace parts. Machining rates for large components used for aircraft structures, for example, are therefore often above 90 wt %, the highest in the metal sector.^150,219,380^

As an example, in the coming years, the production of the more than 1500 planned midsized airliners currently on order around the world will generate approximately 220,000 tonnes of waste titanium in the form of oxygen- and iron-contaminated chips. This equates to a monetary value of approximately seven billion euros. Titanium is about 20 times more expensive than common steel alloys because of its complicated, energy-intensive production so that this huge amount of material lost during machining poses a substantial sustainability burden.

During machining, the titanium chips are heavily contaminated, including oxidation, cooling lubricant residues, and abrasion particles from tools, mainly steel. As a key customer for titanium components, the aerospace industry has very high standards for the material’s quality, making it impossible to recycle high-quality titanium chips to date. Due to this contamination, chipped titanium can currently mostly only be downcycled, i.e., used to produce titanium dioxide as preproduct for white wall paint or as an additive in steel making, to act as a microalloying element for carbide formation. Yet, the metal is actually far too valuable and too energy-intensive to produce for sacrificing it readily to such downcycling methods.

This demonstrates that, while recycling is often not commercially viable today, it consumes far less energy than titanium extraction from ore. As a result, large consumers, such as the aerospace industry, frequently purchase titanium from overseas providers and ship the scrap back for processing, primarily for downcycling. With the introduction of novel recycling approaches, this might change. One promising approach is to use moderately reducing plasma furnaces, in which the contaminated metal can be melted by using heated and ionized gas, while in vacuum arc furnaces, the intrusion of impurities is avoided in the absence of air.

Another research direction is to reduce the corrosive contamination of titanium chips in the first place during the course of ablative machining, Table 26. This is due to the fact that titanium components are manufactured in machine tools through milling, drilling, and grinding. Coolant constantly flows onto the area where the tool is working to prevent the temperature of the components from becoming too high during machining, which would change the material’s microstructure and thus affect the strength, toughness, and ductility of the final component. However, these conventional cooling fluids permanently contaminate the chips, due to the high affinity of titanium to oxygen.

**Table 26:** Research Opportunities Related to Titanium Scrap

Use of lubricants which cause less corrosion during machining of titanium parts (less oxidation)
Less tool abrasion contamination during machining (lower iron content)
Use of titanium chips for powder production for downstream use of additive manufacturing
New smelting techniques for recycling of moderately contaminated titanium chips (instead of downcycling)

As a result, research is being conducted on new coolants that can be completely washed off and oxidize and contaminate the titanium less. The shape of tools, such as milling heads and drills, which are precisely adapted to today’s coolants, must also be changed. The material from which they are made is also being improved. This is because fine particles in the contamination abrasively chip off the tools during machining. It is also investigated whether such less-contaminated chips can be used for the production of titanium powder for additive manufacturing. The aim is to bypass the energy-intensive melting process and to feed the chips directly into an atomization process, which is intended to produce fine powder suited for applications in additive maunfacturing.

Compared to primary synthesis, the use of chips as input material in powder production makes it possible to reduce energy consumption and CO_2_ emissions by up to 80%.

#### 6.3.9 Electrical and Electronic Scrap as Resource for Urban Mining

**Figure 108:**
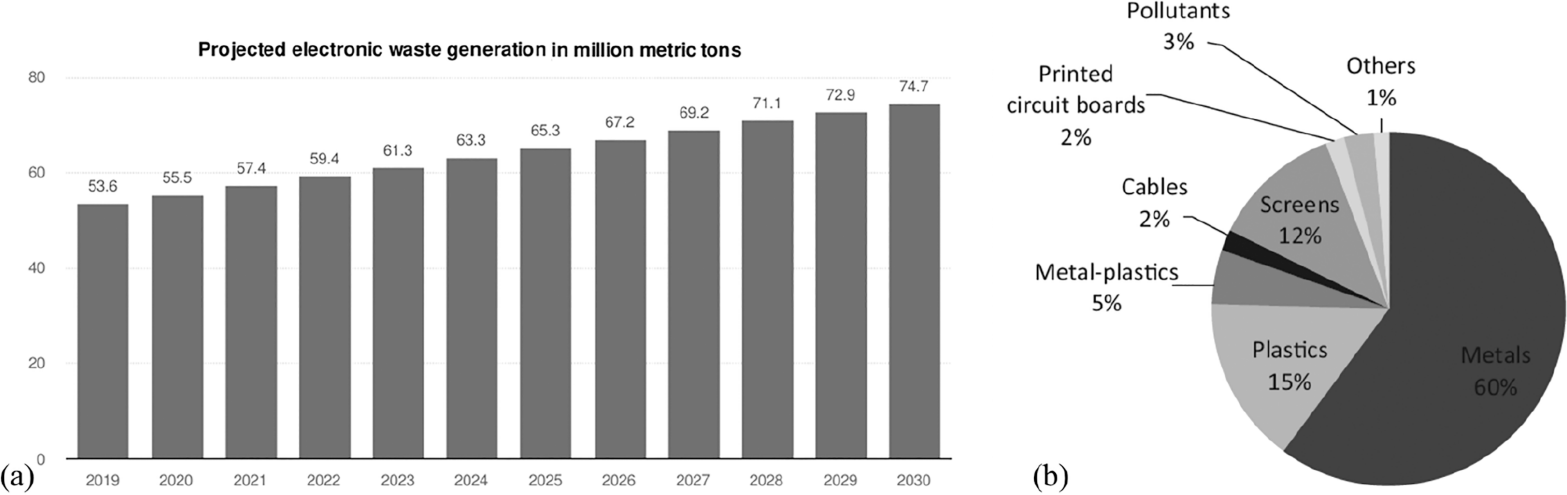
(a) Total global electronic and electrical waste in million tonnes per year. (b) Metal fraction in electrical and electronic equipment.^8,419^ Figure reproduced with permission from Statistika. Copyright 2020, Statistika.

Over the last five years, the amount of electrical and electronic waste has increased three times faster than the global population and 13% faster than the total global gross domestic product.^8,202^ Today, the global annual amount of electrical and electronic waste is about 62 million tonnes, about two-thirds of which are metals, when counted by weight, Figure 108.

The main reasons for the growth of this type of waste are the increasing consumption of electrical products, their often very short lifespan and the difficulty of having electrical appliances and gadgets repaired, Table 27. The latter point is quite relevant in that context because recycling-oriented design of many electrical and electronic products and parts, that would allow for instance to readily separate the battery, the magnets, and the electronic components, etc. during disassembly, would greatly improve the recyclability and thus the sustainability of such products.

**Table 27:** Typical Origin of Waste Form Electrical and Electronic Equipment and Metals That Can Be Extracted

Type of electrical and electronic equipment	Parts	Metals that can be recovered
Mobile phones, telephones and computer pads	Batteries, electrodes, contacts, wires, LCDs, storage media, metal casings, lead frames	Cu, Au, Ni, Co, Cd, Ag, Al, Nd
Personal computers and television sets	Wires, cables, storage media, speakers, batteries, LCDs, lead frames, wires	Cu, Au, Ag, Al, Nd, Dy
Refrigerators, freezers, air conditioning equipment	Tubes, liners, condenser, wires	Cu, Fe, Ni, Cr, Au
Small household equipment	Vacuum cleaners, microwaves, irons, toasters, copying equipment, electric knives and kettles, electric shavers, scales, calculators, radio sets, video cameras, video recorders, hi-fi equipment, toys, printers	Cu, Fe, Ni, Cr, Au, Co
Large household equipment	Washing machines, dryers, dish washers, cookers, stoves, hot plates, musical equipment, large printing machines	Cu, Fe, Ni, Cr, Au

A big challenge is particularly the short life span of many electrical products. Every electronic gadget and equipment with their often very limited life span generates a huge mass of waste which has traditionally gone to conventional household waste incineration, landfill disposal, or sometimes directly to smelters. When for instance only considering cell phones, the fraction that is at all collected and fed into appropriate recycling workflows is less than 20%.

The increasing complexity of materials and equipment containing a mixture of metallic and nonmetallic substances (sometimes even more than 50 elements in one product and/or material system) presents a huge challenge, as simple melting does not allow us to produce new material from such waste, but instead complex highly product-specific polymetallurgical recycling workflows must be developed to cope with such multi-element and nano-integrated waste streams.

The continuously increasing mass of electronic waste and spent catalysts (including three-way catalytic converters, diesel oxidative catalysts, and petroleum catalysts) is a huge challenge that also falls into this category. Many electronic components and also catalysts appear in the corresponding products in nanostructured form, with an integration of often only a few nanometers, creating nanoscrap. This intense mixing is a huge challenge when it comes to the recycling of the individual elements particularly of the precious metals in them. This means that the problem of electronic, electrical, and catalyst waste is 3-fold, namely, first, their nanoscale integration into the material system they are embedded into, second, the fact that many of these products and processes often occur together with up to 50 or even 60 different elements in the same part, and third, that most of these parts have a very short lifetime, Figure 17. Another important driver for the use of functional materials and compounds where such multi-metal mixtures commonly occur is the rapid growth in the use of thermal machines such as refrigerators and air conditioners. This is mainly due to growing consumption in hot countries, where these appliances significantly improve the standard of living.

Figure 108 shows how this massive amount of scrap will continue to rise steeply in the coming years. It is estimated that the global annual electronic waste production could reach 74 million tonnes by 2030; i.e., it is forecast to more than double in just about the next 16 years. Currently, only about 17% of the world’s total electrical and electronic waste is recycled while 83% of it is disposed in landfills or incinerated, and with them, valuable or even toxic materials contained in electrical appliances. As an example, for the latter elements such scrap usually contains chlorine and fluorine from packaging and cable sheathing which requires special attention when the material is recycled. Even something as valuable as gold is only recovered to about 30% of its original value.

The amount of electrical and electronic waste, when figures from 2019 are taken as a basis, contains over 50 billion euros—in the form of gold, silver, copper and other valuable metals. But so far, most of these end up in unused and nonrecycled scrap. As a benchmark, that exceeds the gross domestic product of many countries.

**Figure 109:**
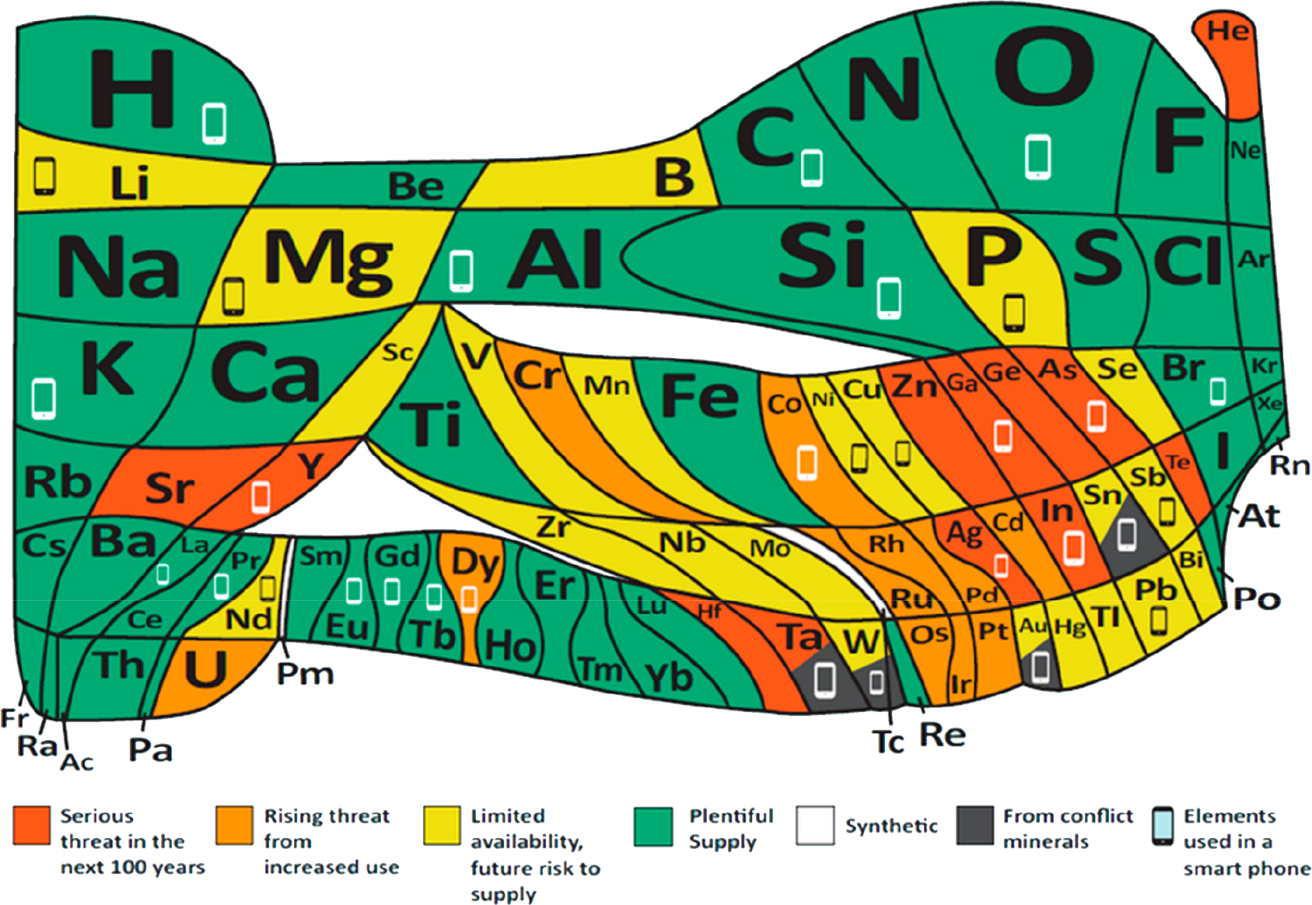
Periodic table of the elements distorted according to element abundance. Many of the particularly scarce elements are used in consumer electronics. The figure is reproduced in modified form with permission from the European Chemical Society, under a Creative Commons Attribution licence (NoDerivs CC BY-ND). Copyright 2022, European Chemical Society. The original figure can be found at https://www.euchems.eu/euchems-periodic-table.

**Figure 110:**
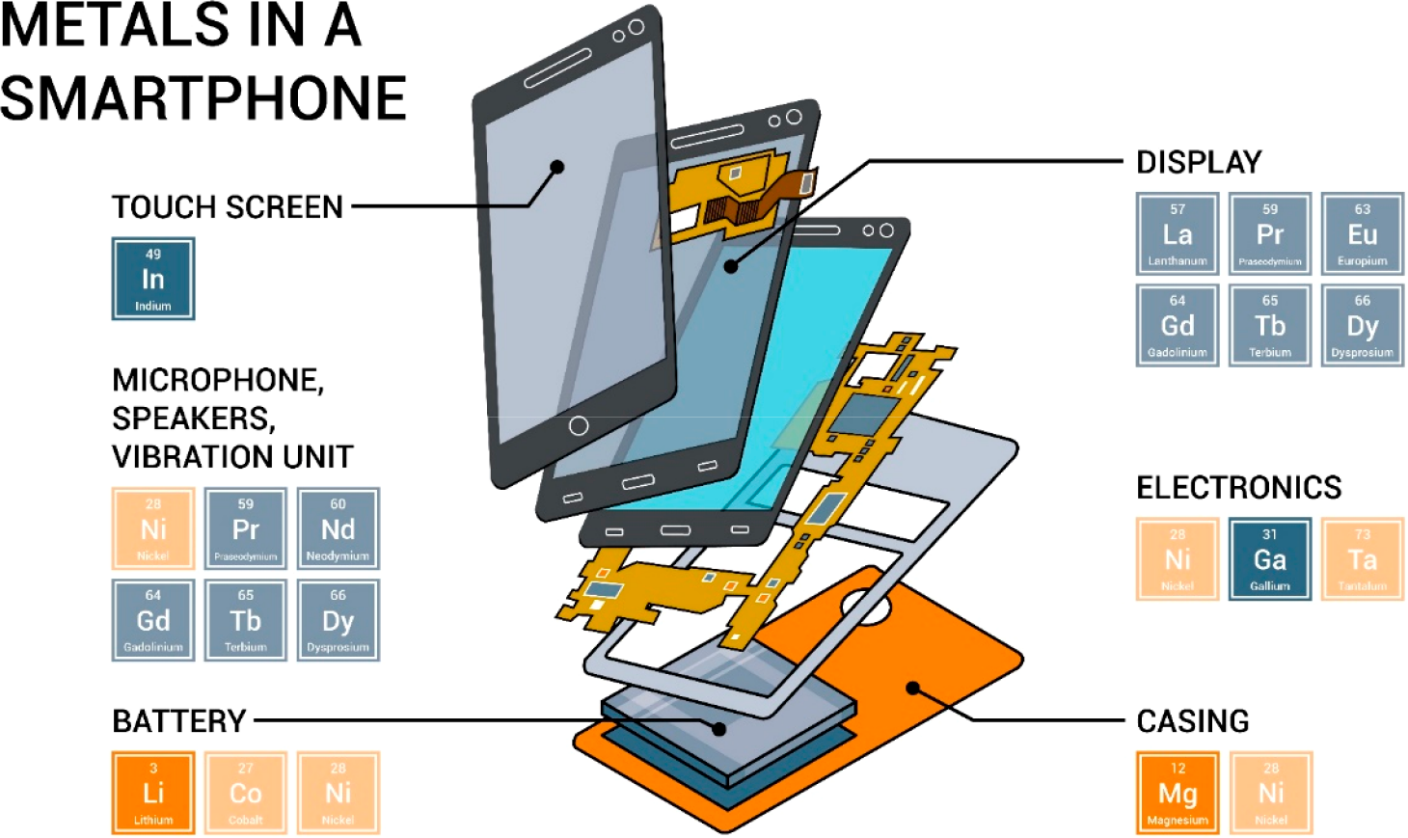
Important elements used in cell phones.^23^ See also Figure 17.

This loss is 2-fold problematic. On the one hand, huge values are lost, and on the other hand, electrical appliances contain many important and, above all, critical and rare raw materials, Figure 109. Many electronic devices contain more than half of all elements of the periodic table, especially smartphones and laptop computers, Figure 110. If they are not recycled, the future production of these devices will become much more expensive.

A third important aspect, besides loss of value and the loss of critical and strategically important elements, is toxicity. Older electrical appliances in particular, which only gradually end up in the scrap, often also contain toxic substances such as mercury: it is estimated that 50 tonnes of mercury were contained in the undocumented electronic waste streams alone last year, for example in monitors, circuit boards or energy-saving lamps.

A trivial reason for this insufficient level of recycling is often the fact that recycling cannot currently compete financially with primary synthesis. However, this state of affairs is problematic because many of the elements found in electronic waste have a strategic role and are rare in nature. This may not be a sufficient incentive for a specific company to blend this point in their recycling and pricing considerations, but it can become an issue for a state. In addition, wealthy societies outsource some of the hand sorting from these vast quantities of scrap metal to poor regions of the world where sorting is done often under unsatisfactory labor conditions or even by child labor sometimes without any safety precautions.

Recovering the elements from electronic waste is a polymetallurgical recycling challenge: in principle, most of the precious metals and also many other metals can be recovered from such waste, for example, from a printed circuit board, if state-of-the-art hydrometallurgical and electrochemical processes are used.^201,202^ However, such polymetallurgical recovery requires a carefully staggered workflow that involves a variety of recovery methods and must be adjusted for the specific products recycled.

It must be considered that the product accessibility for dismantling is very different for the different electronic and electrical components. For example, an automotive catalyst or personal computer motherboard is easily accessible for dismantling and downstream sorting, whereas a circuit board used in car electronics is hidden behind complex parts and is thus usually not so accessible, raising again the urgent demand for better recycling-oriented product design that has the “gene” of sort-specific disassembly and metal recovery already built-in. As long as circuit boards, magnets, battery parts, etc. are isolated and dismantled before the car is put through the shredder, the precious and rare metals they contain can be metallurgically recovered, as will be discussed in more detail in the sections below.

Economic viability is an essential driver in this field. A dismantled motherboard electronic circuit has a positive net value; therefore, recycling it is viable by itself. In contrast, a dismantled ultrathin platinum group metal-coated hard disk from a personal computer usually has a negative net value when recycled, due to the cost of processing it. Recovering the platinum and/or ruthenium from it would currently not be economically viable.^96^

It is also important to establish collection workflows and incentives for customers to ensure that the product is at all made available for recycling. If collection mechanisms are not in place, items such as old computers or mobile phones may end up being stored in households or discarded into the waste bin for landfill or municipal incineration. The precious metal they contain would effectively be lost to the recycling chain.

A very important aspect lies also in trade chains: Most of the electronic waste, particularly electronic circuits, mobile phones, or cars containing electronic parts, magnets and catalysts are often sent (either legally or illegally) to countries without proper infrastructure for recycling. This can also result in precious metals being lost to the recycling chain.

Another aspect of such scrap types is better sorting: it is essential that items such as circuits from computers or cell phones are not mixed with other low-grade electronic waste and channelled into a shredder process without prior removal of the precious metal-containing circuit boards. The same applies to the platinum group metal-containing catalyst in a car or fuel cell, Table 28.

**Table 28:** Research Opportunities Related to Electrical and Electronic Scrap

Better sorting technique, including sensor-, spectroscopy- and machine learning-assisted scrap sorting
Better integration of product design for material-specific disassembly and corresponding recycling workflows
Multi-metal recycling techniques (see specific sections 7.4.12, 7.6.4, and 7.7.7 below)
Mutual element contamination from multi-metal recycling techniques

The recovery of metals from such waste streams including collection, sorting, and the actual metallurgical processing thus requires us to not only revisit the fundamentals of extractive metallurgy techniques for recovering a few specific precious metals (such as gold, copper etc.), but also the multi-metal challenge of extracting many more metals from such waste streams must be studied, Figure 17. These questions include the downstream processing and separation, material liberation, urban mining with halide-, cyanide-, thiosulfate-, and thiourea-based lixiviants, purification, precipitation, adsorption, supercritical fluid extraction, biomediated approaches, solvent extraction, and chromatographic techniques, etc. Details of the pyro-, hydro- and electrometallurgical methods to recover the metals from such types of waste are tackled in separate sections below.

#### 6.3.10 Recovery of Metals from Battery Scrap

**Figure 111:**
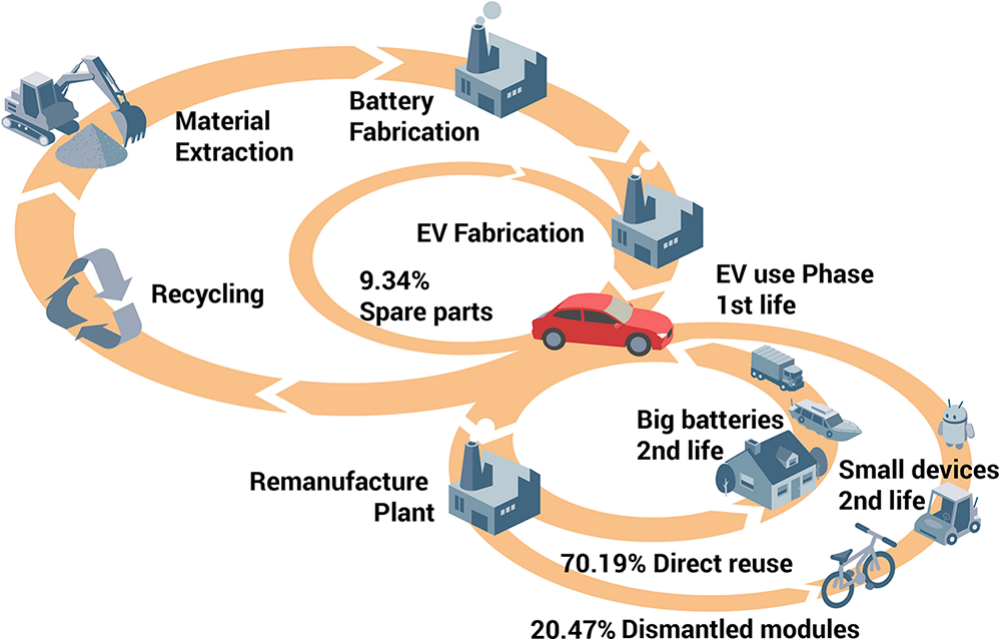
Remanufacturing, repurposing, reuse and recycling are three alternatives for handling used batteries.^426^ Figure redrawn after numbers reproduced with permission from ref (426). Copyright 2017, Omniascience. EV, electrical vehicle.

Today, less than 100,000 tonnes of lithium ion batteries are globally recycled.^19,420^ However, due to the rapid expansion of lithium-ion battery use in electric vehicles, there will be a significant and rapidly growing amount of used lithium-ion batteries in the near future. Remanufacturing, repurposing, and recycling are three alternatives for appropriately handling used batteries, Figure 111–Figure 113.

**Figure 112:**
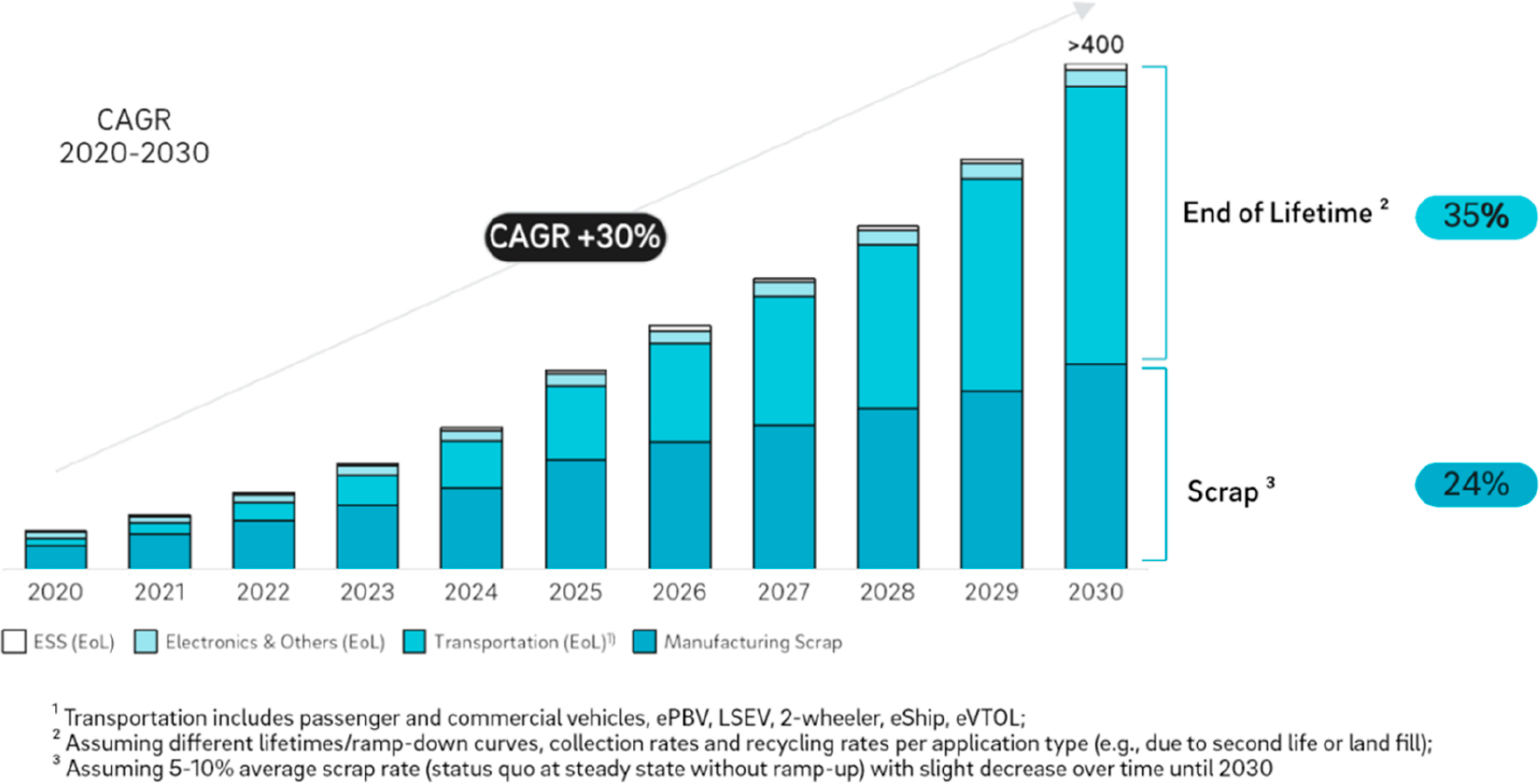
Development of globally available lithium-battery scrap material for recycling in units of GWh equivalent.^427^ CAGR, compound annual growth rate. GWh, gigawatt hours. Figure reproduced with permission from ref (427). Copyright 2019, Roland Berger.

**Figure 113:**
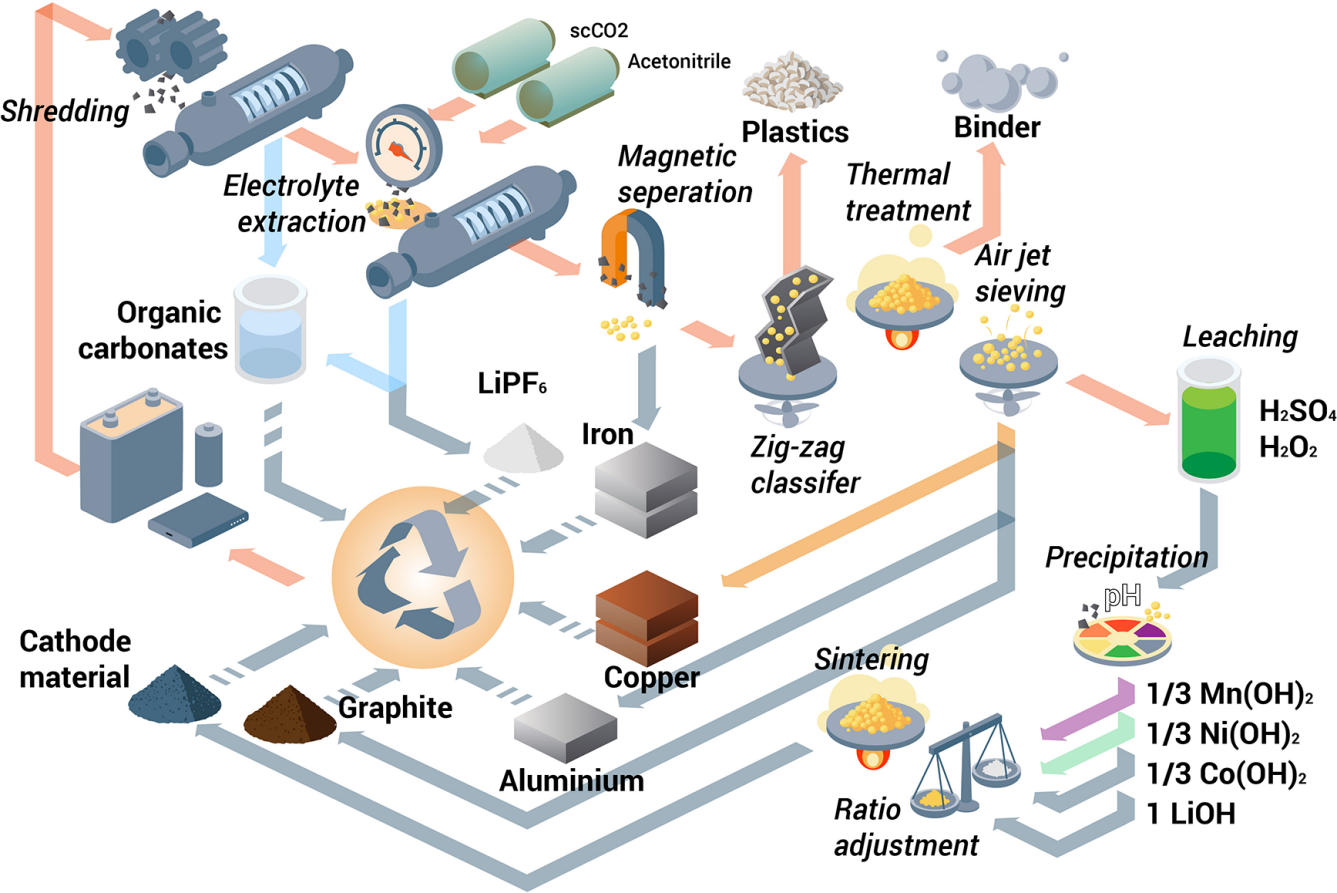
Recycling pathways and methods for lithium-ion battery recycling.

Lithium-based ion batteries contain a large number of valuable materials that can at least in part be recovered and for which more efficient and high-quality recycling processes must be developed. The product life span of lithium-ion batteries is estimated to be about 2.5–8 years for batteries in electronic devices, and 8–10 years in electric vehicles.

The recovery of metals from batteries is not only a matter of sustainability also but an economic necessity, because many of the elements in them are sparse and expensive. When assuming about 1.5–3 million tonnes of batteries being recycled by the year 2030, a quarter of a million tonnes of active materials in them as electrode or electrolyte materials such as nickel, cobalt, manganese, and lithium as well as the electrical contact metals such as copper and aluminum but also polymers and graphite could be recovered. This number translates for instance to around 20–30% of the future global cobalt and nickel demand, showing the magnitude of the coming battery market and the economic leverage of the task.^111,230,231,421^

**Figure 114:**
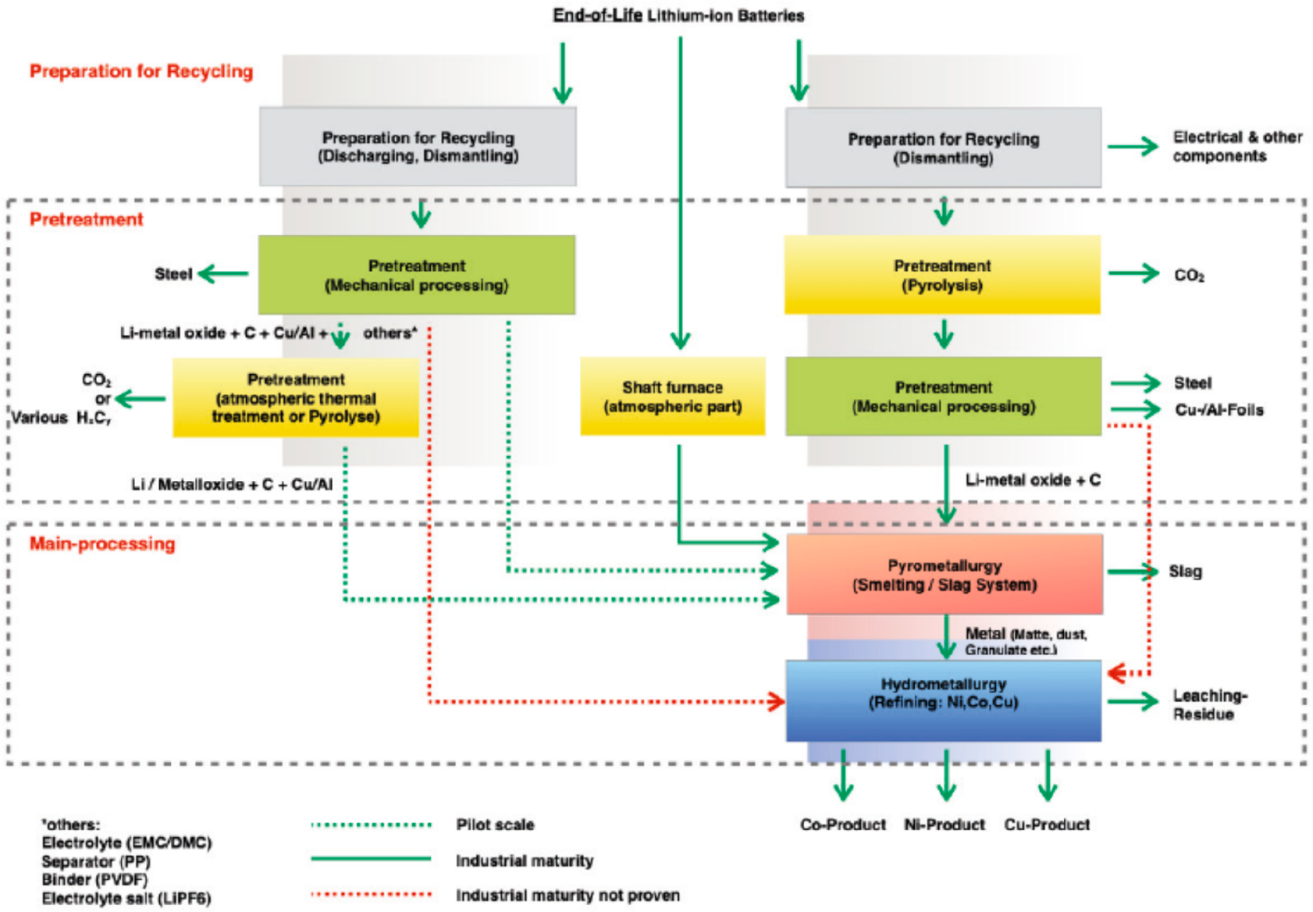
Different recycling routes for polymetallurgical recycling: example of metal recovery from vehicle batteries.^427^ Figure reproduced with permission from ref (427). Copyright 2019, Roland Berger.

**Figure 115:**
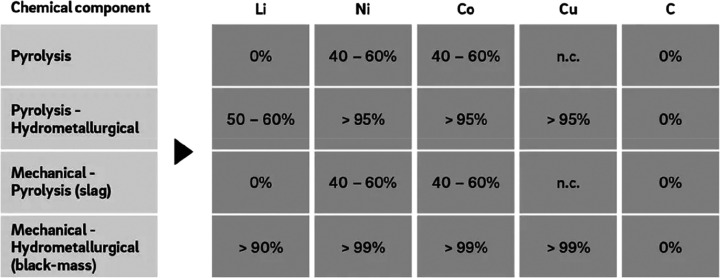
Recycling efficiency achieved by several metallurgical process methods.^427^ Figure reproduced with permission from ref (427). Copyright 2019, Roland Berger.

Pyrometallurgy, hydrometallurgy, and direct recycling are the three main recycling processes used to recover metals from spent lithium-ion batteries,^223,229,422,423^ as discussed below in more detail, Figure 114 and Figure 115. Therefore, research into sustainable battery recycling and element recovery, including product design aspects such as the separate disassembly of battery parts, must become an integral part of this technology, but current recycling technologies and the consideration of recycling upfront in product design are not yet well developed.

Mostly, recycling procedures for batteries start with dismantling, separation of metallic and polymer fractions, discharging, electrolyte evaporation, and inert or cryogenic shredding. Especially the materials used for contacting copper (at the graphite anodes) and aluminum (contacting the lithium-metal-oxide cathodes) can be recovered through this first process step.

Prior to most metallurgical treatments, the batteries’ reactivity has to be first reduced. This can be achieved by a cryogenic, moderate vacuum, or inert gas treatment of the batteries. The batteries are crushed (for instance under inert or cooled conditions) and then treated with an alkaline solution. Lithium salts can then be extracted from the resultant cake. One approach is that lithium carbonate is produced by adding a lithium salt solution in a tank. Lithium carbonate is then used as feedstock for several types of lithium compounds. Other processes use instead lithium oxide or lithium is recovered via evaporation, via a pyrolysis process where the temperature is gradually increased so as to sequentially exploit the different evaporation, oxidation, and melting points of the different components in the battery.

Like in the case of metal extraction from electronic scrap, the recovery of the different chemical elements from large-scale vehicle battery systems is a polymetallurgical recycling problem.^35,40^ Due to the large differences in quantity, melting points, vapor pressure and oxidation behavior of the different elements in such multicomponent battery scrap, the use of conventional pyrometallurgical methods alone, which essentially melt scrap, is usually not efficient and leads to small recycling rates and to low-quality recycling of material that can often only be downcycled and not be used in new battery products. This means that a conventional pyrometallurgical treatment allows us to recover only a few specific elements such as copper and nickel, while for example low-melting or very reactive elements are turned into slag or dust. Particularly the valuable and scarce lithium is oxidized into the slag during conventional pyrometallurgical recycling processes. In principle this lithium which has turned into a mixed slag can subsequently be recovered through hydrometallurgical processes, combining flotation, extraction, concentration and precipitation steps.

A certain advantage of pyrometallurgical recycling is that it does not require that the spent batteries are entirely discharged, which simplifies the processing, particularly when applying processes with a slow rise in temperature.

For the recovery of nickel and cobalt from the electrode materials and the copper from the contacts of vehicle batteries, different approaches have been introduced so far. In principle two types of recovery pathways prevail, namely, a pyrometallurgical process, in which nickel, cobalt, and copper are recovered through smelting, and a hydrometallurgical approach, where the components are dissolved via acid leaching.

Other methods involve burning of the entire battery at 1000 °C, which eliminates combustible components like the electrolyte and separators. After that, the batteries are crushed and sieved and magnetic separation of iron (and to some extent also of the nickel and cobalt) from the residue is conducted. The majority of the powder that passes through the sieve is made up of carbon and different variants of lithium-, cobalt-, and nickel-rich oxides.^226^

Alternative methods subject the fine shredder fraction to a hydrometallurgical treatment process. The complex hydrometallurgical refining step usually consists of an autoclave, (chlorine) acid leaching of the intermediate product from pyrometallurgical treatment, precipitation, and filtering of non-noble metals or undesirable elements, followed by solvent extraction and nickel electrowinning, ion exchange and cobalt electrowinning.

The acidic transition metal sulfite solutions and the lithium salt solutions can be isolated by precipitation and filtration. For this purpose, some process workflows conduct treatments of the lithium salt solution in an ion exchanger. The lithium salt solution is then split into acidic (hydrochloric or sulfuric acid) and alkali components (lithium hydroxide monohydrate). The lithium hydroxide must usually be further purified with the help of crystallization. The metal sulfite solution contains, among other things, cobalt, nickel, manganese and aluminum and is also processed.

Further roasting, dissolving, precipitation, filtering and electro-extraction processes take place in parallel and sequentially on side routes to extract copper and other by-metals such as lead, precious metals, etc. An economically viable recovery of the most important and valuable components of a lithium-ion battery will in the near future mainly focus on metals such as cobalt, nickel and copper, which is only possible after a detailed pretreatment and pyrometallurgical intermediate purification in order to finally feed these metals as high-quality recycled products back into production.

Most of the current recycling processes combine pyrometallurgical with hydrometallurgical methods. In such process chains, electrolytes contained in a furnace are at first gradually evaporated in a preheating zone by slowly increasing the temperature. The slow rise in temperature minimizes the risk of explosion and eliminates the need for an upstream discharge step of the spent batteries. In the following polymer pyrolizing regime, temperatures of up to 700 °C are reached and the plastic parts are melted down and can be separated. In addition, halogenated products can be recovered in this process step. At the higher temperatures, several smelting and reduction process take place, in which oxygen-rich air is added and several material fractions are produced. One portion contains mainly aluminum, silicon, calcium and iron, while a second one contains an alloy of copper, cobalt, nickel and some of the remaining (undesired) iron. The latter metal is unwanted in this step, as it is not compatible with the downstream metallurgical refining of the copper, cobalt and nickel mixtures. This second fraction is further processed and separated. However, the lithium remains in this process in the state of a lithium oxide in the resulting slag or as dust and is often lost for reuse.^224,390^

Different from nickel, cobalt is not used in such large fractions as an alloying element in other metallurgical products such as stainless steels.^424^ Therefore, recycling has played a minor role in the cobalt market so far. An average recycling volume of around 13 thousand tonnes of cobalt per year was estimated for the past years. The contribution of recycling to the total cobalt supply is only around 10% today. It can be expected that integrated recycling systems may eventually become important in value chains by contributing to reduced production costs and particularly to improved sustainability for the production of electrodes for lithium-ion batteries of electrical vehicles. Even though only small quantities are currently made available to the market, the recycling sector will therefore play a stronger role in supplying cobalt and other battery metals in the long term. From battery scrap, cobalt is usually recovered as a sulfate, carbonate or tetroxide. In this state it can be directly used during the subsequent manufacturing of new cathode precursor material.^40,233,371,422,424^

Another important aspect that is often less considered in the discussion of recycling and the corresponding development of more sustainable battery systems (particularly for the growing vehicle market) is the replacement of certain critical metals used in them today. This includes, for example, the replacement of the less abundant, expensive and environmentally unfavorable lithium by the widely available sodium (e.g., in seawater), as well as the development of electrode materials that do not require the use of nickel and cobalt.^425^ Intensifying the basic research in this direction alone could make a considerable contribution to increasing the sustainability of modern battery systems. In addition, there is also a growing market for the intermittent electrochemical storage of sustainable electrical energy with the help of batteries that are not used in vehicles but in stationary form. Here, the size and weight of the battery only play a subordinate role. Such relaxed boundary conditions would therefore also allow for the use of less demanding and more sustainable materials that would be less attractive for mobile applications due to volume or weight.

Table 29 lists some research topics related to metal recovery from battery scrap.

**Table 29:** Research topics related to metal recovery from battery scrap.

Better integration of product design and recycling requirements. More specific, recycling-oriented product design should allow the recovery of the battery prior to shredding or related disassembly and scrapping steps where the materials are mixed more than necessary
Replacement of critical metals in batteries by environmentally less harmful ones, such as the replacement of lithium by sodium and also of certain cathode materials such as nickel and cobalt by other transition metal oxides
Development of multi-elemental recovery methods with product- and metal-specific staggered sequences of pyrometallurgical and hydrometallurgical recovery techniques (see more details in the sections below)

#### 6.3.11 High-Quality Recycling in Multi-Metal Recovery from Mixed Scrap

As discussed in the previous sections, the biggest challenge in recovering metals from scrap is that materials should in an ideal case be collected and sorted by type or subsystem (such as alloy-specific structural components, extrusion parts, cast parts, batteries, thermoelectric materials, (hard and soft) magnets, catalysts, electronic circuits, etc.) and then be fed into a product- and metal-specific recycling process.

This is a particularly big challenge in recycling for two main reasons. The first problem is that most of the scrap available on the global market is not sorted by alloy, but it is highly mixed and thus contaminated material.^7,133,134^ This is a particular problem for the mass market of aluminum alloys and practically all functional metallic materials (magnets, conductors, electrodes, catalysts, and so on), as these alloys are particularly sensitive to the intrusion and gradual accumulation of scrap-related impurities.

Second, there is the rapidly growing challenge of a scrap type that can be called nanoscrap (or even Ångström-scrap), especially for particularly expensive but also for harmful or even toxic elements. This is scrap from (mostly electrical and microelectronic) products in which the integration of different elements is so intensive that they can no longer be separated or even presorted by classic shredding and sorting processes.^89,202^ This refers particularly to nanometer- and even Ångström-scale integration in modern integrated circuits, which in turn are closely integrated and “hidden” in dispersed form in more complex consumer gadgets and machines such as vehicles and production lines, from which they cannot be recovered, Figure 17 and Figure 110.

This establishes a very strong demand for better overlap with future recycling-specific design requirements of electronic (or likewise battery, magnetic, thermoelectric, etc.) parts and their use in more complex machinery, processes and products. For example, the future design of complex products must take into account that batteries, electronic components (especially computer chips), and magnetic components, etc. are taken out separately before scrapping them, instead of simply scrapping them all together with the whole product, without any pre-separation, as is currently the case.

Several authors referred to this approach as “high-quality” or “high-tech” recycling.^11,19,202^ This is based on the understanding that it is much more important to pre-sort scrap in product disassembly with respect to the separation of those elements that are mutually particularly “poisoning” when it comes to the subsequent pyro- or hydrometallurgical separation and sequential multi-element recovery process chains, rather than achieving high nominal recycling rates of highly contaminated scrap, which is practically useless.

This means that for many of these particularly impurity-sensitive and often expensive products and alloys mentioned above it is particularly important to find out which the most “poisoning” and harmful tramp elements are and make sure that these elements are removed already in the presorting process, prior to the actual metallurgical downstream recycling processes, so as to eliminate the most harmful intruding elements from the scrap or even from the original alloy design in the first place.

A critical example is for instance to avoid mixing the highly reactive metals from batteries such as lithium or sodium with the contact and cell metals such as iron, copper and aluminum.^423^ Also it is important to avoid mixing electrode materials such as nickel, cobalt and manganese with highly reactive materials or with precious metals. In cases where this cannot be avoided, the focus must be placed on the recovery of the most valuable metals. For instance, while copper, nickel, cobalt and lead can be treated in the same or at least in similar metallurgical recovery process chains with precious metals, contamination with iron must be strictly avoided and lithium is generally rendered into the slag in such processes, owing to its high affinity to oxygen. Particularly all the non-noble metals such as lithium and rare earth elements must therefore be separated before from copper, nickel, cobalt and lead as well as from precious metals.^40,422^

An important problem in this context of electrical and electronic scrap is the magnetic separation technology, where from shredded electronic waste parts which contain ferromagnetic magnetic particles with high residual magnetic moment (e.g., from iron, nickel, cobalt or rare earth magnet particles) the valuable gold and copper components are often sorted out as useless material, together with magnetic iron particles, and are thus lost.^223^

In the field of aluminum alloys one of the most typical and harmful contaminant elements is iron.^74,384,428^ This means that for instance welded or glued composite parts of steel and aluminum must be eliminated from waste streams before they are melted together.^7,429,430^ A similar strategy should be applied to steel scrap where particularly copper, tin and lead are the most harmful elements that must be avoided in entering scrap streams as good as possible.^10,143,160,431^ Applying such elementary boundary conditions during the separation and presorting steps early on in the recycling workflow paves the path toward more high-quality recycling.

### 6.4 Re-mining of Tertiary Feedstock: Recovery of Metals from Dumped Waste

#### 6.4.1 Introduction to Tertiary Feedstock

The mining and metal industry has produced huge amounts of processing waste, slags, ashes, dust, waste rock material, and mine tailings, usually summarized as “extractive waste”, over multiple decades or even centuries,^28,57,432^Figure 77. This waste can become new feedstock for extractive metallurgy (and for other branches, e.g. in construction and ceramics), Figure 78. Such feedstock sources are different from secondary ones (metallic scrap) because the metallic elements in the dumped materials are usually in oxidized state, i.e. not in metallic form, and can thus be termed “tertiary” feedstock. This and the fact that such waste materials are mostly mixtures of several compounds make the task of extracting metals from them via reduction more similar to the field of primary synthesis than to the field secondary synthesis where scraps are molten.

This material mainly comes from the re-processing of older mine residues or from the use of dumped downstream processing waste. In the future such resources could become a third pillar of sustainable metallurgy and re-integrate material into the value streams that had already been lost for a circular economy. Approaches to win such waste materials back and to use and re-integrate them into a circular economy are often referred to as “re-mining”.^56,58,78^

Tertiary or re-mined raw materials also differ from secondary raw materials such as scrap because they are often returned to the market in a highly chemically contaminated form, containing the elements of interest in a very diluted and mostly in oxidized state. For this reason, many of these industrial and post-consumer residues have so far been usually more economical to be dumped as landfill than to recycle. In this way, a cost and sustainability competition arises between the landfill costs on the one hand and the costs and the impact on the environment in the case of a possible reprocessing and return to the circular economy on the other hand. It must be emphasized that some types of reprocessing of residual materials are energetically and chemically so costly that they cannot be regarded as more sustainable solutions in comparison to a linear economic component, where they are simply dumped, Figure 11 and Figure 12. However, when pursuing a closed loop recycling approach as a basis of a future sustainable metallurgical sector, every atom in a product or process should return as a feedstock material (minus the entropy related losses). However, the environmental consequences must in all such solutions be first proven by adequate life cycle assessment calculations to not turn an idea that sounds reasonable upon first sight into a finally environmentally harmful solution.^14,29,128^

Tertiary raw materials of economic importance in connection with metallurgy are, for example, red mud from aluminum production,^433−435^ tailings from preprocessing of sulfidic ores,^28^ or shredded polymer waste^436^ which can be made available either as a reducing agent in conventional fossil-based oxide reduction furnaces or as additional fuel (as many hydrogen-based reduction methods are endothermic and require additional heat), Figure 78.

The preconditions that the re-mining of dumped tailing and industry resources become viable from a commercial and particularly from a sustainability perspective are recent advances in process technology and the growing market pull for some of the more expensive metals. Regarding progress in pyro- and plasmametallurgical processes, advanced hydrometallurgical and bio-leaching methods, pre-separation and sorting methods, more efficient fine flotation processes, separation of tailings into different grades such as high-/low-sulfur or high/low-arsenic ones, etc. and more efficient hydrometallurgical methods for residual metal extraction are needed.

From a market perspective, of course, particularly the precious materials such as platinum group metals, rare earth elements, gold, silver, nickel, cobalt, tin, tungsten, lithium, and copper, etc. could be attractive future target elements to re-mine from such tertiary resources.^82,349,433,437,438^

#### 6.4.2 Red Mud as a Mineral-Rich Ternary Resource from the Aluminum Industry

Red mud is a bauxite residue that is produced during the large-scale extraction of aluminum oxide (alumina)—an intermediate product of aluminum production—from the aluminum-containing bauxite ores,^246,439,440^Figure 78. Bauxite is a multicomponent mineral mixture consisting mainly of aluminum oxide and aluminum hydroxide as well as iron oxide and iron hydroxide. Secondary constituents are mainly titanium oxide, silicates, traces of heavy metals and even rare earth metals that can be found and recovered from this waste material.^79,81,82,281,434,437^

To extract aluminum oxide, the soluble part of the bauxite is dissolved in caustic soda under high temperatures and pressure in the Bayer process. The aluminum compounds contained are converted into water-soluble sodium aluminate, Na[Al(OH)_4_], and separated from the water-insoluble remainder by means of extraction. Aluminum hydroxide (Al(OH)_3_) is precipitated as sodium aluminate solution by dilution and cooling. This is then fired to aluminum oxide (Al_2_O_3_) in fluidized bed plants or in rotary kilns and reduced to metallic aluminum by fused-salt electrolysis in the Hall–Héroult process.

The iron and heavy metal compounds remain as a suspension or dispersion in a strongly alkaline solution with a pH value of 10–12, forming a substance referred to as red mud. The characteristic red color comes from the high fraction of solid particles of iron(III) compounds such as for example iron(III) hydroxide and iron(III) oxide, suspended in the sodium hydroxide solution. In order to operate the Bayer process as efficiently as possible and to reduce production costs, as much caustic soda as possible is usually removed from the residue in various substeps and reused.^348,435,441^ This results in a residue with lower alkalinity.

The amount of red mud per tonne of aluminum produced depends on the composition of the bauxite feedstock, which varies depending on its origin. For tropical bauxite, about 1.6 tonnes and for European bauxite 3.2–3.7 tonnes of wet red mud are typical values.^351^

Red mud contains all the gangue substances contained in the bauxite ore it comes from, such as mainly iron and titanium oxides and various silicic acid compounds. The minor components it contains to a lesser extent vary with the origin of the ore. More specific, red mud can also contain several heavy and toxic metals such as arsenic, lead, cadmium, chromium, vanadium or mercury. The composition of the main constituents of red mud varies in the ranges Fe_2_O_3_, 5–20 wt %; Al_2_O, 5–30 wt %; TiO_2_, 0.5–15 wt %; CaO, 1–15 wt %; SiO_2_, 3–30 wt %; and Na_2_O, 1–10 wt %. The average median size of the particles constituting the red mud is normally in the range of 5–15 μm with a broad particle size distribution ranging from a few nanometers to several millimeters.

**Figure 116:**
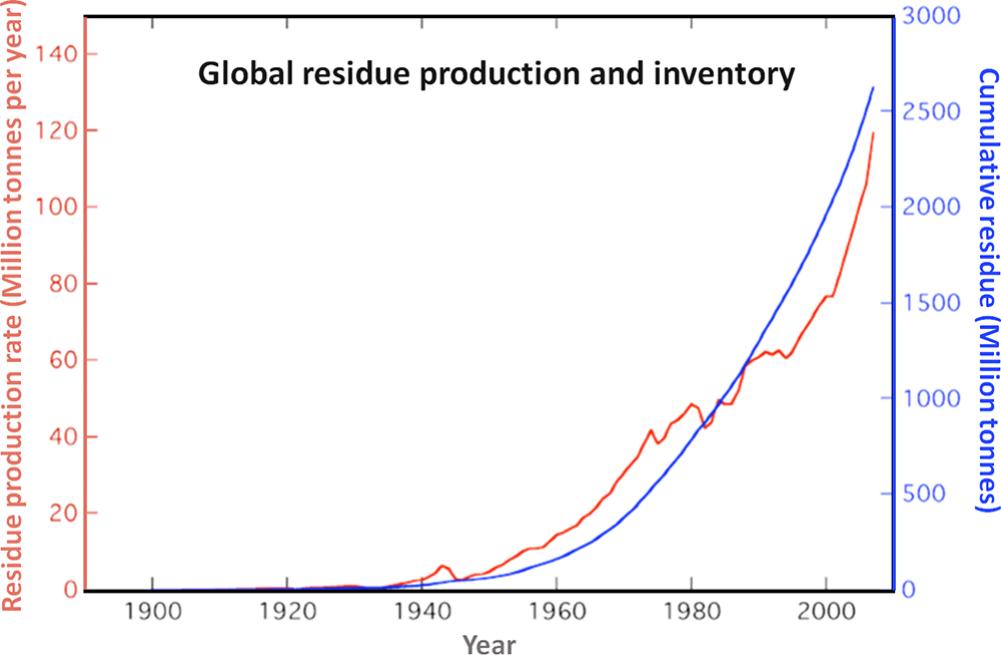
Global red mud production from the aluminum industry.^435,444^ Red mud has today piled up to the staggering amount of 4 billion tonnes on the globe.^441^ The figure is reproduced with permission from ref (441). Copyright 2009, CSIRO Minerals Australia.

**Figure 117:**
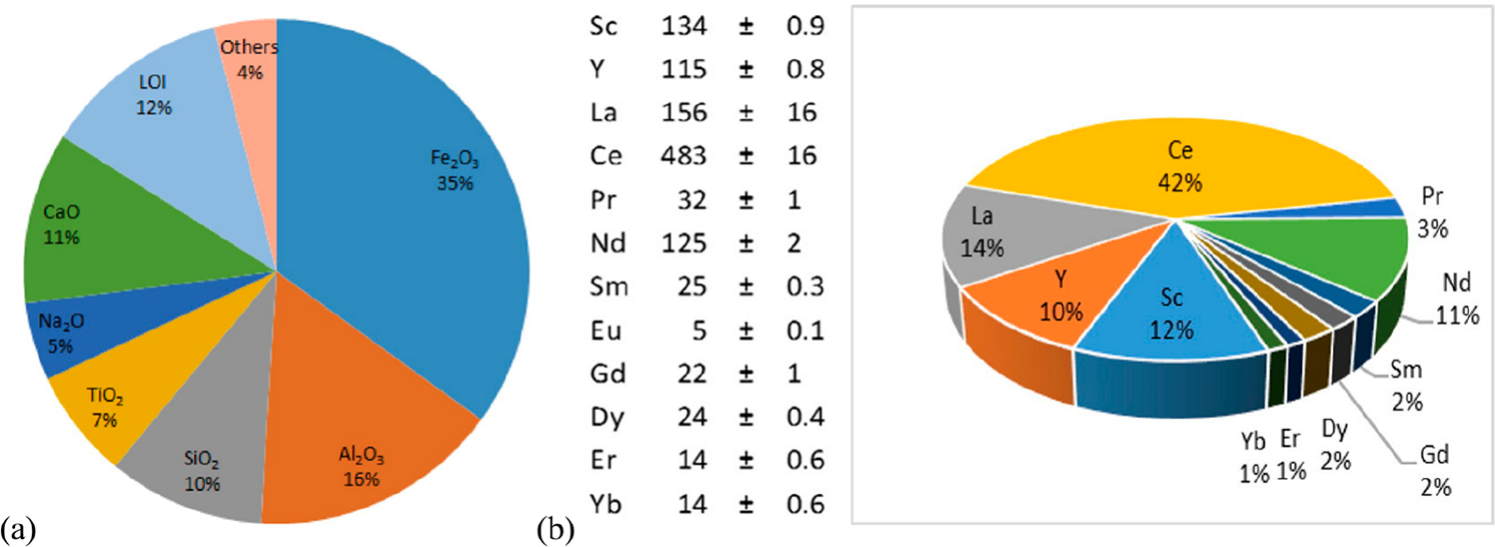
(a) Global average red mud composition.^351^ Here not only the recovery of the large dumped material quantities could be of interest (such as iron and titanium) but also that of the rare earth and precious metals. (b) Rare earth elements found in red mud, recovered via selective leaching from red mud, using a functionalized hydrophobic ionic liquid.^438^ Figure reproduced with permission from ref (351). Copyright 2021, Springer.

About 150 million tonnes of red mud are produced globally each year, with a growing trend, Figure 116 and Figure 117. Less than 2% of red mud is recycled today. Due to its very high basicity and the leaching of hazardous elements, more responsible deposition or re-mining approaches are a pressing environmental challenge and task.^349^

Estimates suggest that 2–3.5 million tonnes of the 150 million tonnes of bauxite residue annually produced could be reused already today, where the main consumers could be the cement industry with up to 500,000 to 1,500,000 tonnes per year and the steel industry with 400,000 to 1,500,000 tonnes per year.^442,443^ Various categories can be considered regarding the possible uses of bauxite residues. These include the extraction of iron, titanium and rare earth or use of the residues in constituents of building materials such as used for concrete, bricks, tiles and soil amendment.

#### 6.4.3 Iron Winning from Red Mud

Red mud is a large-scale residue and harmful yet metal-rich by-product of the Bayer process used for extracting aluminum oxide from bauxite ore. Bauxite, the dominant mineral feedstock for aluminum production, is a sedimentary rock consisting mainly of the aluminum minerals gibbsite (Al(OH)_3_), böhmite (γ-AlO(OH)) and diaspore (α-AlO(OH)), mixed with two iron oxide variants goethite and hematite, the aluminum clay mineral kaolinite, and the titanium oxides anatase (TiO_2_) and ilmenite in the form of FeTiO_3_ or FeO mixed with TiO_2_.

The iron oxides, together with aluminum oxide, typically make up the majority of red mud.^282^ As a result, it has been repeatedly investigated and exploited as a potential alternative oxide material in place of or combined with mined hematite iron ores.^442,445^ Red mud has not yet been used commercially or sustainably for the manufacturing of steel, despite the material’s high alkaline content and the effectiveness of the reduction techniques. However, due to the ongoing efforts to mitigate the greenhouse gas emissions of the global steel industry, recently several reduction methods have been studied to revitalize this idea. A successful reduction of the iron-containing oxides in the red mud into metallic iron (and possibly also into other metals) would combine one of the largest industrial waste burdens, namely, red mud, with one of the largest metal markets and greenhouse gas emitters, namely, steel production.

Two directions of recovering iron from red mud have been pursued so far. The first group encompasses roasting methods followed by magnetic separation, and the second one is based on a usually fossil-reductant-driven smelting-reduction process to which the bauxite residue is subjected in an electric furnace. Detailed parameter studies for both processing pathways were conducted by Li et al.^446^ They found that the main factors influencing the metallic yield and the energy efficiency of the processes are the magnetic field strength, the roasting temperature and time, and the ratio between the carbon and the red mud. The optimum reduction reaction conditions were reported to occur for 1 wt % of carbon added to the red mud at temperatures of about 1450 °C and a roasting time of 60 min, followed by subsequent magnetic separation. It must be considered that these numbers likely vary when other red mud compositions are used. Under these test conditions, the authors recovered a 65% iron containing compound at a total iron recovery rate of 67%. Li et al.^446^ suggested that the use of an electromagnetic induction furnace is preferable over the use of a resistive furnace for this purpose.

Kaußen and Friedrich^447^ studied various types of reductive smelting processes for different chemical compositions of red mud. They focused on the amount of reductant required and the composition of the resulting iron and slag phases. They combined thermodynamic simulations with laboratory-scale electric arc furnace melting tests. Bhoi et al.^443^ studied the same problem. They used red mud with a 55 wt % hematite fraction, using a process chain consisting of reduction roasting, magnetic separation and a subsequent hydrogen plasma smelting route. Valeev et al.^282^ conducted reductive smelting of red mud with up to 60% hematite content in a laboratory-scale Tammann furnace by using carbon as reductant in a temperature range between 1650 and 1750 °C, to recover the iron from red mud. They obtained iron with fractions of titanium, phosphorus, and vanadium, but with a low sulfur content.

For chloride-rich solutions, Aliquat solutions showed good iron extraction performance with less than 10% loss of other metals.^434^ Another approach is direct leaching: in their study Bonomi et al.^346^ showed that red mud could be efficiently leached using an acidic ionic liquid. They achieved almost a total dissolution of iron and 30–40% dissolution of aluminum and sodium as well as a high recovery yield of titanium (90%) and scandium (almost 80%).

#### 6.4.4 Titanium Recovery from Red Mud

Besides iron and aluminum, also titanium is an attractive element to recover from bauxite residue.^434^ Pietrantonio et al.^434^ introduced a hydrometallurgical method to extract titanium from red mud, which is a very alkaline material. Utilizing a hydrochloric acid leaching procedure, ammonia precipitation, and solvent extraction employing toluene as a solvent, quantitative recovery of titanium with a high purity level above 95% was achieved in his work. The same authors also looked into the removal of other metals from aqueous red mud solutions and discovered an order of adsorption according to the sequence iron > lead > copper > manganese > zinc. They came to the conclusion that red mud may therefore serve as a promising re-mined future metal feedstock material.

With a focus on the titanium leaching from red mud, Alkan et al.^80^ observed that sulfuric acid is the best treatment option for red mud, with a titanium leaching effectiveness of 67%. In a different paper, the authors suggested smelting red mud at temperatures between 1500 and 1550 °C as a pretreatment step to increase the efficiency of scandium and titanium recovery at reduced consumption of less acid. Smelting allowed recovering iron to the metal phase and concentrating other major and minor elements in the slag.^448^

The impacts of different processing parameters on titanium (and scandium) recovery from red mud were examined by several authors with regard to the leaching of iron, titanium, scandium and the residual aluminum in the red mud.^80,81,434,449^ It was observed that a few processing factors were of particularly high relevance of the recovery rate, namely, assistance by ultrasonic waves, acid concentration, reaction time and the reaction temperature. These parameters were observed to have the greatest effects on leaching efficiency.

#### 6.4.5 Rare Element Recovery from Red Mud

The rare earth elements as well as scandium, gallium, yttrium, uranium, and thorium are among the trace elements in red mud that are interesting candidate materials for re-mining.^433,438,442^ Particularly certain rare earth elements can be found in bauxite residues as tramp cations that replace other elements in some of the minerals’ lattice structures or as ion absorbates on the mineral particle surfaces.^281^

The unique features of some of these elements, for instance some of the rare earth metals, make them irreplaceable in many products, for instance in hard magnetic materials. Some of these elements are of strategic relevance in many countries in terms of economic importance and supply risks. Due to the difficulties in economically mining rare earths and the rising demand, sustainable re-mining strategies to recover these elements from discarded wastes like red mud have been considered.

At least some of these elements can be recovered from the red mud by using hydrometallurgical methods or combinations of pyrometallurgical and subsequent hydrometallurgical techniques. Also, bio-leaching methods were tested in that context.^437^ Most methods for the recovery of rare earth elements from red mud are based on dissolving it in a 20–50 times diluted acid solution that leaves the iron in the red mud.

Several studies have tested different leaching conditions and extraction techniques for recovering rare earth elements from bauxite residues.^281,433,438,442^ It was reported for example that leaching with HNO_3_ with a liquid-to-solid ratio of 50:1 allowed recovery of 80% of the scandium, 90% of the yttrium, and 70% of the heavy lanthanides dysprosium, erbium and ytterbium, 50% of the medium lanthanides neodym, samarium, europium and gadolinium, and 30% of the light lanthanides lanthanum, cerium and praseodym.^434^

When utilizing sulfuric acid leaching, comparable recovery rates for certain of these elements were reported, albeit at a smaller liquid-to-solid ratio of 20 and shorter leaching periods of 2 h at room temperature.^349,433^ For leaching from red mud, additional acid solutions, dilution ratios, exposure temperatures, and periods, as well as the associated recovery rates, were described in the literature.^281^

#### 6.4.6 Polymers and Carbon-Containing Post-consumer Waste as Reductants

Using polymer-containing shredder residual materials as an alternative reducing agent in the smelting and reducing processes is one way to re-integrate carbon-containing waste material into the metallurgical production cycle.^450,451^ These materials can come from both post-consumer sources and industrial deposits.^452^ Long polymeric chains with a carbon-based backbone and regular hydrogen side chains make up the majority of polymers. Both substances have the potential to replace or enrich other reducing agents. Therefore, polymer waste has long been used to reduce the usage of fossil (mainly coal-based) reductants, and it was thought that using such waste material in this way would be more sustainable than just depositing it.

This strategy has been widely used in the steel industry since the 1990s, primarily as a supplementary source of heat and reductants in blast furnaces, but it is also used to produce steel in electric arc furnaces as a substitute CO source that reduces iron oxides and provides heat and combustion.^453^ Depending on the reactor, the waste is injected either in shredder form or in a fine dispersion using plastic injection systems. However, one significant drawback to using such polymer-based waste for the production of iron is the impact of some of the impurity metals that are present, where copper is particularly detrimental.

The general approach, however, unquestionably merits in-depth further consideration by the research community, given the abundance of polymer waste that is currently available and that is contaminated with significantly lower copper contents. Such polymer material could, therefore, be recycled into metallurgical production, for example waste from the tire industry or polymers recovered from oceans. This creates the opportunity to study for example the intrusion of particular impurity elements and their impact on the reduction process, its effectiveness, and the final metallurgical product, similar as in the case of other re-mined raw materials like low-grade ores or red mud.

Some of the impurity-related disadvantages mentioned above turned out to be less harmful in the case of nonferrous bath-smelting processes. For nonferrous smelting and reduction processes, in which mainly coal-based reductants are used today, it is known that coal contributes to reduction through the gasification reaction of carbon in char. Yet, most polymers decompose under the presence of a low amount of char and a high amount of volatiles. Studies on polymers used in blast furnaces show the formation of C1–C4 hydrocarbon products, as well as H_2_, and CO, which can all participate in reduction reactions. Yet, for the use of larger fractions of the often quite heterogeneous types of waste polymers as alternative reducing agents, more detailed studies about their reducing effects and the associated impurities must be conducted.

Besides the fact that all these approaches release further greenhouse gases into the atmosphere, they are from a process perspective also challenging to handle because they often come with quite variable carbon content (depending on the waste and shredder composition) so that close monitoring of the reduction progress is required.

In principle, such approaches are, however, of interest in the future, as they create a nexus and point to solutions to two of the biggest environmental problems we currently have, namely polymer pollution and greenhouse gas emissions from the metal industry. This combination would not only reduce the consumption of primary resources such as coke, coal and methane, but it would also help to reduce the burden associated with tertiary sources such as waste polymers, qualifying this field for more systematic studies.

### 6.5 Biological and Organic Feedstock in Sustainable Metallurgy

#### 6.5.1 Bacteria and Fungi for Bio-leaching and Bio-oxidation

Processes that fall under the umbrella of biomining encompass methods where microorganisms serve in mineral processing, re-mining and recycling. Organisms such as bacteria and filamentous fungi assist through their metabolism in converting otherwise poorly soluble minerals such as for instance copper sulfides into leachable water-soluble copper sulfates. Such bio-leaching techniques are increasingly considered as process steps in hydrometallurgical processing chains for biological metal extraction from ores and recovery from waste materials.^93,273,454,455^

As organic feedstock, two major types of microorganisms and their respective metabolic mechanisms are currently mainly used for material conversion and bio-leaching of valuable metals from mixed minerals or waste materials. These are archaea and bacteria.

Archaea are unicellular organisms that do not have a nucleus with a nuclear membrane like eukaryotic cells, but—much like bacteria—they possess self-contained DNA molecules which are arranged in the form of circular chromosomes that are present in the cytoplasm as a nuclear equivalent without a shell. Bacteria, like archaea, are prokaryotes, which means that their DNA is not contained in a nucleus separated from the cytoplasm by a double membrane as in eukaryotes, but in them, as in all prokaryotes, the DNA lies freely in the cytoplasm, crowded together in a narrow space, the nucleoid (nuclear equivalent).

Examples for archaea used in biometallurgy are acidophilic lithoautotrophic archaea and for bacteria the acidithiobacillus ferrooxidans, translating to “iron-oxidizing bacillus” as well as organoheterotrophic bacteria and fungi.^456^ Such microorganisms can be used in industrial biomining because of the ability of their respective metabolism to oxidize reduced iron and sulfur compounds with atmospheric oxygen (aerobically) to form sulfuric acid, dissolve metal sulfides, and thereby dissolve metals.^269^ Some of these organisms can also oxidize reduced sulfur compounds without the presence of atmospheric oxygen by reducing Fe(III) in iron oxides or hydroxides (e.g., goethite) to Fe(II) ions, dissolving the iron compounds and dissolving valuable metals bound therein. These microorganisms are therefore referred to as anaerobically organisms. The *acidianus brierleyi* is even thermophilic; i.e., it prefers high-temperature environmental conditions, which is an important feature to accelerate the corresponding accumulation and leaching processes.

As an example, such reducing bio-leaching mechanisms have led to the development of a novel laboratory process for nickel and cobalt recovery from laterite ores.^270,457^ Chemically, bio-leaching by acidophilic lithoautotrophic archaea and bacteria is thus based on acid formation (called acidolysis of minerals), on the one hand, and on redox reactions (called redoxolysis), on the other. Details about the specific application of bacteria in biomining and bio-leaching along the otherwise hydro- and electrometallurgical extraction process chains are discussed in the section below on biometallurgical processes (see section 7.8).

#### 6.5.2 Use of Organic Waste and Biomass as Carbon-Based Gaseous Reductants

Gaseous renewable carbon-based reductants from organic, industrial, and post-consumer waste can be provided in the form of biomass and from incineration plants.^178,331,333,402,458^ This approach makes thus use of waste products from industry, agriculture and the consumer sector, in the form of sustainable and renewable carbon and hydrogen carriers. In such considerations, CO_2_ continues to be released into the environment as a product of the underlying redox reactions occurring during the metal oxide reduction, but when reabsorbed by plants a carbon cycle can, in principle, be created.

Two main approaches have been considered. The first one uses solid biomass directly as a reducing agent in metallurgical processes, due to its high carbon and hydrogen content. The second approach is indirect, whereby suitable sustainable gaseous reducing agents are first obtained from the biomass, such as methane, methanol or hydrogen, via power-to-gas technologies, such as biomass reforming for example.

In the first scenario biomass is used in the same way as waste polymer; i.e., it can be for instance charged into blast furnaces by using high injection rates and thereby reduce the amount of coke required.^49,178,458−460^ This process is pertinent but to some extend limited on the one hand because the internal structure and percolation in a blast furnace only work if a certain structural framework of coke is maintained. On the other hand, it must be taken into account that there is simply not enough renewable or sustainable biomass available for the replacement of all the coke in the operation of blast furnaces.^49,164,459,461,462^ This means that the extensive use of biomass and biogas as a carbon-based reductant is limited by the availability of regional cropland. In addition, their energy and industrial utilization is in competition with other industries. Social resistance exists especially against the use of land for energetic and industrial biomass applications instead of using it for growing food crops. Also, it must be avoided that, in the case of corresponding market incentives from the metal sector, this approach could lead to a global trend to cut down forests and plant instead fast-growing products for this approach, which would certainly not be regarded as a sustainable approach. Furthermore, it has to be taken into account that for the transition of the metal economy the required biomass quantities exceed by far the available biological stocks. Extensive use of biomass in steel production is therefore not a long-term realistic solution to the greenhouse gas problem in the metallurgical sector, simply due to the immense global market demand and the competition with food production.

### 6.6 Alternative Reductants to Replace Carbon in Sustainable Metallurgy

A variety of carbon-free or carbon-reduced molecular carriers and their mixtures can be used in metallurgical reduction and heating processes.^128,177^ The most promising are sustainably produced (green) hydrogen, ammonia, methanol, and methane, as well as mixtures of these media, with the required partial pressures and temperatures derived from the underlying thermodynamic equilibria as shown in Figure 58–Figure 60. Their use as a substitute for coke in metallurgical reduction is especially promising. In principle, they can be used as injection gases in blast furnaces, direct reduction shaft furnaces, or fluidized bed furnaces, and they have also been used in plasma reactors in the form of electric arc furnaces. It is important to note that for the metallurgical sector, these gases do not have to be as clean as those required for fuel cell applications, which is an important aspect for cost reduction, namely by using lower purity gaseous reductants.

In this context green hydrogen (and reductants made from it such as green ammonia) is a very attractive and efficient reductant (for extractive metallurgy) and fuel (for downstream heat treatment of metals during manufacturing), provided it can be produced at the currently required quantity of about 110 million tonnes per year for the metallurgical sector alone, using exclusively renewable energy sources. However, the transcontinental transport of hydrogen is an issue. Bringing chemical energy in the form of green hydrogen stemming from the currently prevalent electrolysis production from regions with a high fraction and low price for renewable energy to regions of high consumption (such as for instance the metallurgical sector) is inefficient, as its cooling (required for shipping) consumes up to 35−40% of the energy it embodies. Therefore, liquid green ammonia or other green chemical hydrogen vectors are alternative molecular forms for transcontinental chemical energy and reductant transport. Ammonia, with the chemical formula NH_3_, has a high volumetric hydrogen content (∼121 kg-H_2_/m^3^ versus 70.8 kg-H_2_/m^3^ in liquid hydrogen at 21 K) and a high energy density (4.25 kWh/L versus 2.81 kWh/L of liquid hydrogen). Ammonia thus demonstrates itself as an efficient and cost-competitive hydrogen carrier and energy storage vector, that can also be used for other purposes, such as fertilizer in agriculture. The main bottleneck or respectively key question in this context is not only the use of ammonia in reduction processes but also the production of sustainable ammonia based on green hydrogen, as otherwise it is not a sustainable reductant carrier. The electrically driven Haber–Bosch process is such an approach, as it enables the production of green ammonia. In fact, ammonia has played a key role in global fertilizer production since one century, with high technology readiness in production, liquefaction, storage, and transport. These advantages explain the high interest to use the green ammonia-mediated energy and hydrogen cycle for future sustainable steel production.

Ammonia can be liquefied by pressurization (∼8 bar at 25 °C) or refrigeration (33 °C at 1 bar). This means that in the past the hydrogen in ammonia was used as a vector for the chemical transport of nitrogen for fertilization in agriculture, while in future, with regard to sustainable metal production, the nitrogen in the ammonia might conversely be used as a vector for the chemical transport of hydrogen to be available as a sustainable reducing agent, for example in the steel industry. Currently, however, due to the dominant market position of the classical Haber–Bosch process, which is mostly operated worldwide for this purpose, ammonia production itself has a very high carbon footprint. For this reason, research on the sustainable production of ammonia is of utmost importance for sustainable metallurgy. Considerations regarding the joint efficiency and sustainability of production and transport such as outlined here for the specific case of green ammonia can be also made for other power-to-gas molecules that have a potential to serve as reductants and fuels in the metallurgical sector, such as methanol or methane.

## 7 Sustainability Aspects in Extractive Metallurgical Processing

### 7.1 Role of Raw Material Quality for Sustainable Extractive Metallurgy

Extractive metallurgy is the step in which the metals are recovered from their mostly oxidized and/or compositionally mixed states. It is by far the most energy- and greenhouse gas-intense step in metal production. Like the other sections, this chapter is not a general overview of the field, but focus is placed on aspects of high relevance and leverage for improving metallurgical sustainability. This means that the following sections make no claim to completeness with regard to the fundamentals of extractive metallurgy, but they rather concentrate on those processes that have high leverage on improving the sustainable of metal recovery.

**Figure 118:**
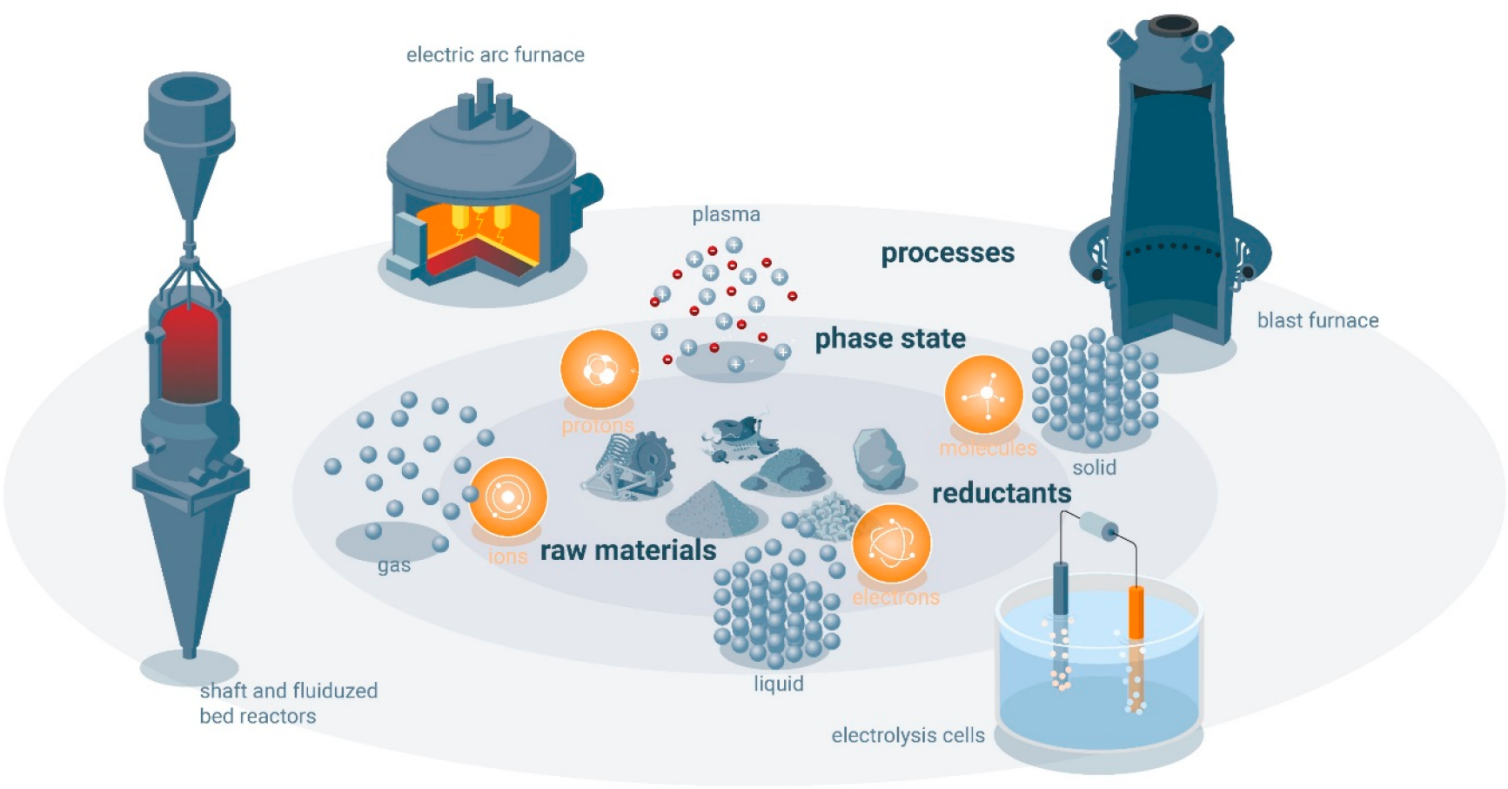
Possible combinations among different types of raw materials, (sustainable) reductants, their aggregate states and associated processes and reactor types for metal reduction and extraction, shown here exemplarily for the case of iron making.

Some of the feedstock-related opportunities for improving metallurgical sustainability discussed in the preceding sections depend on innovations in process metallurgy. This concerns possible changes of existing aggregates and workflows, the use of alternative reductants and their mixtures, or the invention of entirely new process chains altogether. For this reason, there is a close relationship of this section with the topics discussed in the previous chapters, which presented many of the new possible raw materials, reductants, and benefication processes, Figure 118.

A closer look at metallurgical reduction and metal extraction techniques is also important because these processes usually generate not only huge CO_2_ emissions but also large volumes of waste and residues, which have to be treated, disposed of, and/or reused. Due to the enormous future market growth, the demand for metals will drastically increase over the next decades, and so will the linear portion of the metallurgical sector, where all these challenges but also the research opportunities become larger and not smaller, Figure 15.

In general, due to the expected market growth and the resulting higher market costs, the metallurgical industry will in the future have to consider raw material sources with in part lower metal content than before, Figure 71. This applies for both primary (e.g., mineral ores) and/or secondary and ternary (e.g., slags, tailings, municipal waste) raw materials.

While the situation for a mass product such as steel is still relatively good in terms of raw materials, i.e. the ores and scrap usually have a comparatively high metal richness, the situation is much worse for many other metals. This means that for every tonne of metal produced, up to 20 times as much soil is moved, treated, contaminated or produced as waste material for finally recovering some of the elements. For example, in the production of many critical elements such as nickel or cobalt, due to their very high dilution in the naturally occurring mineral sources, a huge amount of slag is always produced as well, which is also constantly heated and treated in the corresponding pyrometallurgical enrichment and extraction methods. This creates huge burdens in terms of energy consumption and greenhouse gas emissions. This means that there is a direct correlation between the dilution in which certain metals are available in the raw materials and the energy required to extract them in metallic form, Figure 71.

As a result, mining, waste recycling, and metallurgical processing need to be considered jointly, to develop more selective, efficient, and ecologically friendly mineral and metal processing methods.

### 7.2 Introduction to Extractive Metallurgy and Its Role for Sustainability

Extractive metallurgy encompasses all mechanisms and process steps that are used to recover metals from their oxidized states, including purification and in part also recycling.

Most metals occur in mineral form as mixed oxides and sulfides in the earth’s crust. The metallurgical liberation of the metals from them proceeds through (electro-) chemical redox processes where the oxidized metals get reduced and the reductants become oxidized. The magnitudes of the thermodynamic driving forces required for metallurgical extraction are calculated from the energy balance of the underlying oxidation and reduction processes, Table 15 and Figure 58. Depending on the bond strength of the oxidized metal in its respective oxide or sulfide mineral state, different types of reductants—including also electrochemical or additional thermal support via cofueling—must be used to trigger the corresponding redox reactions.

Prior to these actual (electro-)chemical reduction processes, extractive metallurgy workflows usually begin with a variety of enrichment steps. Depending on the ore type, these can include the aggregation and accumulation of the minerals, beneficiation processes such as comminution, various thermal and floatation treatments, magnetic separation, etc. to remove undesirable gangue minerals such as for example veracious oxides of silicon, aluminum and titanium. The techniques employed rely on the kind and amount of gangue minerals, their distribution within the ore, the level of metal concentration, and the chemical similarity between the gangue and the targeted metal mineral.

The next set of processes is referred to as beneficiation. It can include several techniques to further enrich and concentrate the gangue-reduced target mineral agglomerates, form chemically purified minerals, or convert them into chemical forms more suitable for subsequent metal extraction.

The next step is the actual metallurgical extraction, involving reduction and recovery of the metal from its oxidized state.

Traditionally a distinction is made between the fields of nonferrous and ferrous metallurgy. The latter includes the reduction of iron oxides, carbonates and sulfides into iron, and its further refinement and alloying with other metals to make steel. Nowadays, only oxidic ores are commercially relevant. The other branch is nonferrous metallurgy. It includes the (electro-) chemical extraction of all the other metals. This field is usually further broken down into a few subgroups. The largest groups are (a) light metal extractive metallurgy which includes the recovery of the metals magnesium, aluminum, scandium, and titanium, owing to certain similarities in their chemical behavior during extraction (sometimes also including tin extraction); (b) base metal extractive metallurgy, which is concerned with the recovery of lead, zinc, copper, cobalt and nickel; and (c) precious metal extractive metallurgy which deals with the recovery of gold and silver and the platinum group metals.

The actual metallurgical extraction can be done via pyrometallurgy, plasmametallurgy, hydrometallurgy, solvometallurgy, ionometallurgy, electrometallurgy and biometallurgy. Some of these approaches overlap and fall into the main groups pyrometallurgy, hydrometallurgy and electrometallurgy.

Metallurgical extraction is referred to as pyrometallurgy when high temperature processes are involved; hydrometallurgy when liquid solutions are involved; and electrometallurgy when electricity is used. Transitions between these techniques and emerging subdisciplines with relevance for sustainable metallurgy are discussed below in more detail.

Pyrometallurgy includes methods such as melting (phase transformation from solid to liquid), smelting (phase transformation from solid to liquid together with reduction), high-temperature solid-state reduction, fire refining, roasting (of sulfidic ores) and calcination (of carbonatic ores). Roasting, an intermediate process required to prepare sulfidic ores for subsequent metal recovery, proceeds by heating the sulfides in the presence of oxygen wherein the sulfur is oxidized and driven off as sulfur dioxide. Some metals in this process remain in sulfidic form, while others are turned into oxides. The metal can partition into either chemical form.

The term “smelting” refers here to the recovery of metal from its oxidized state by processes that involve reduction, heating and melting. It has to be differentiated from the term “melting”, which refers to the phase transformation of an already reduced metal (e.g., scrap) from the solid into the liquid state, without changing its oxidation state.

Oxidative smelting is in principle an operation that is similar to roasting, but it differs slightly in the way that the temperatures used in oxidative smelting are high enough to promote melting of the oxidic raw materials. Some minerals are more resistant to oxidation, so they remain in the sulfide form, while other minerals are completely oxidized and form compounds with additives, often referred to as flux. Molten sulfides and oxide compounds split in two layers because of the different specific weights. The by-products of these operations are usually sulfur dioxide and carbon dioxide.

Hydrometallurgical methods are mostly based on the use of aqueous solutions to liberate metals from their oxidized states. After purification of the resultant solutions, most hydrometallurgical techniques involve processes referred to as leaching, metal precipitation by pH and oxygen adjustment, gaseous (pressure) reduction, precipitation and/or cementation. Bio-hydrometallurgy is often considered a subtopic of hydrometallurgy. Leaching describes a process for the chemical dissolution of the desired minerals in aqueous solutions. Due to the difference in the dissolution rates, it is possible to separate the compounds of different metals. Often, some oxidative reagents need to be added to enable and promote leaching.

Hydrometallurgical methods offer in principle attractive extraction solutions in the field of sustainable metallurgy because many of these techniques function at moderate temperatures. They are often also more selective in extraction when transforming individual target minerals contained in mixed solutions into an extractable state, for instance through strong acid or alkali leaching. Hydrometallurgical ion exchange methods are successfully used for the enrichment and purification of lean leach solutions and for the separation of chemically similar elements. Solvent extraction methods are used for the selective transfer of metallic ions from aqueous solutions to an organic phase, used for example for purification and separation of rare earth and nuclear elements. Halogenation is often used as an intermediate step in the production of titanium, zirconium, hafnium, uranium, etc. to convert oxides into chlorides or fluorides prior to the final reduction step.

Electrometallurgy uses electrical current for reduction. This approach is particularly important in sustainable metallurgy because it is often more efficient to directly use sustainable electric energy instead of using this energy for the production of sustainable buffer reductants and fuels such as hydrogen, methanol or ammonia. Electrometallurgy is usually applied to reduce the metals contained in relatively pure (refined) molten minerals that are blended into aqueous solutions, ionic liquids or fused salts. If the metal is extracted from the electrolyte using an insoluble anode, the method is called electrowinning. On the other hand, if the impure metal (in the form of a sacrificial melting anode) is refined using a suitable electrolyte, the method is known as electrorefining.

### 7.3 Differences between Extractive Metallurgy Methods Regarding Sustainability

The different extractive metallurgical techniques introduced and sketched above are characterized by a number of general trade-off factors. These include questions related to metallization (metallic yield), efficiency and extraction rates as well as process-specific harmful (or advantageous) by-products and energy consumption regarding sustainability. For example, highly reactive metals such as magnesium or aluminum, which have a high binding energy to oxygen, can be reduced into a metallic state by fused salt electrolysis. Electrowinning is often used as a final refining step at the end of a hydrometallurgical extraction chain. Hydrometallurgy is often used for reducing metals from lean, less pure and compositionally complex mineral feedstock. The disadvantage of electro- and hydrometallurgical reduction is that they have usually slower kinetics compared to pyrometallurgical methods.

The disadvantage of pyrometallurgical techniques is that they usually require large amounts of fuel and/or reductants, currently from fossil origin.

These aspects are currently gaining importance in the field of sustainable metallurgy because the use of electrochemical processes and hydrogen-based processes for heat treatment and reduction is in many cases endothermic and therefore substantial amounts of additional (currently mostly fossil) fuels must be used in order to reach the high temperatures that are often required to initiate the underlying redox reactions, Table 15, Figure 58, and Figure 59.

This is a significant difference to conventional reduction methods, which are mostly based on fossil reducing agents, as these usually run exothermically and the carbon-based substances serve thus both as heat sources and as reducing agents. In general, the high temperatures required in the field of pyrometallurgy are also a significant cause for the emission of greenhouse gases and harmful flue dust.

Hydrometallurgical processes work usually at much lower temperatures, which makes them quite attractive also with regard to sustainability. However, they entail the consumption of large volumes of lixiviants such as H_2_SO_4_, HCl, KCN, and NaCN which have limited selectivity. Moreover, despite the restriction imposed in some regions, cyanidation is still considered the prime process technology to recover gold from ores. Mercury is also used by artisanal miners in less economically developed countries to concentrate gold and silver from minerals, despite its high toxicity.

Biometallurgy makes use of living organisms, such as bacteria and fungi, and although this method demands only the input of O_2_ and CO_2_ from the atmosphere, it requires low solid-to-liquid ratios and long contact times, which significantly reduce space-time yields, Table 30.

**Table 30:** Specific Environmentally Problematic Features and Research Opportunities Associated with the Different Metallurgical Extraction Techniques

Metallurgical process technique	Environmentally problematic features	Possible research opportunities
Pyrometallurgy	Use of high temperatures; energy comes today mostly from combusting fossil energy carriers; processes are mostly using fossil reductants	Low-temperature pyrometallurgy; use of renewable and sustainable energy sources and reductants; use of non-fossil fuels for aggregate heating (particularly important for endothermic processes)
Hydrometallurgy	Use and dumping of harmful chemicals	Development of “green” hydrometallurgy, by using less harmful solvents: emergence of the field of solvometallurgy
Biometallurgy and phytometallurgy	Requires low solid-to-liquid ratios and long contact times, which significantly reduces space-time yields; use of plants with highly metal-ion accumulative properties, for instance to clean contaminated soils or materials that are contaminated with radioactive elements	Biometallurgy with improved efficiency; design of improved microorganisms for biometallurgical processes
Plasmametallurgy	Highly localized input of energy; not always efficient for large scale operations; strong interaction with vessels; unwanted evaporation and loss of elements; better understanding of slag formation and interactions with the melts	Efficient plasma reduction reactors; solid-state plasma reduction; liquid-state plasma reduction methods; use of renewable and sustainable energy sources and reductants
Electrometallurgy	High energy intensity; carbon emissions from graphite electrodes	Longer-lasting and carbon-free cathodes; use of sustainable electrical energy for electrolysis; higher cell efficiency and avoidance of cell freezing also for seasonally variable power availability; reduction of the cryolite melting point (in fused-salt aluminium extraction); use of red mud as a feedstock resource instead of dumping; use of sustainable electrical energy for electrolysis; high cell efficiency and avoidance of cell freezing also for seasonally variable power availability; reduction of the cryolite melting point; use of red mud as a feedstock resource instead of dumping

These first considerations are of course only of a very general nature, and in the following chapters, more specific research requirements pertaining to the different metal groups will be examined in more detail.

### 7.4 Emerging Sustainability Topics in Pyrometallurgy

#### 7.4.1 Introduction to Sustainability Aspects in Pyrometallurgy

The production of many transition metals is dominated by pyrometallurgical reduction. Pyrometallurgy belongs to metallurgical extraction and purification of metals by thermal treatment of mixed minerals, ores and concentrates to trigger physical and chemical transformations in the materials to enable metal recovery. Roasting, calcination, smelting and refining are the most important pyrometallurgical categories. All operations involve chemical redox reactions where the metal gets gradually reduced and the reductant gets oxidized.

Roasting includes several thermal solid–gas treatment processes that are conducted between 350 and 800 °C, to trigger chemical redox reactions between the mineral or its concentrate and the furnace’s ambient reactive atmosphere. Roasting can include different types of (partial) redox processes, such as the feedstock’s oxidation, sulfation, reduction, chlorination, and pyrohydrolysis. An important roasting process is the oxidation of sulfide ores, in which the metal is converted to an oxide. In the presence of air, the metal sulfide is heated to a temperature that permits the oxygen in the air to react with the sulfide to generate sulfur dioxide gas and solid metal oxide, replacing the original sulfide state.

Oxidizing roasting procedures are also referred to as “dead” roasting when the temperature and gas conditions are adjusted such that the sulfide feed is entirely oxidized. When pretreating reverberatory or electric smelting furnace feed, the roasting process is sometimes carried out with less oxygen than is required to thoroughly oxidize the feed. Because the sulfur is only partially eliminated in this situation, the technique is referred to as partial roasting. Finally, sulfation roasting occurs when the temperature and gas conditions are adjusted so that the sulfides in the feed react to generate metal sulfates rather than metal oxides. The process is characterized as “selective roasting” or “selective sulfation” when temperature and gas conditions are maintained such that a mixed sulfide feed (for example, a feed comprising both copper sulfide and iron sulfide) reacts such that one metal forms a sulfate while the other forms an oxide.

Calcination is a term used to describe the changes in solid-state feedstock resulting from roasting. More generally it refers to all kinds of high-temperature processes in which volatile substances are removed from the processed solids and/or where the oxidizing partners or the oxidation stages of a compound are altered. This means that during calcination minerals are thermally decomposed and/or chemically transformed. Volatile products that are usually expelled during calcination are CO_2_ and H_2_O. For example, carbonate ores are often calcined to remove the CO_2_, forming a metal oxide as outcome. Most carbonates thermally decompose at temperatures around 400–500 °C. Besides the removal of moisture or other gases at a temperature range below the ore’s melting point, calcination also serves to reduce, oxidize or dissociate minerals into simpler chemical compounds, which are then downstream more amenable to further refinement and reduction. Calcination processes are conducted in furnaces like rotary kilns, shaft furnaces, and fluidized bed reactors.

Smelting involves thermal and often also a sequence of staggered redox reaction steps in which at least one product is a molten phase, often separating into two or more steps in the course of the chemical reactions. Metal oxides can then be smelted either using either traditional fossil reducing agents, which liberate oxygen in the form of CO_2_, or—in the case of hydrogen-based reduction—in the form of H_2_O, producing a metal product. Such smelting operations are usually conducted above the metals’ respective melting points, where the element partitioning from the respective reductant has to be taken into account when determining the melting points of the reaction products. The process of reducing hematite oxides in blast furnaces into pig iron using coke as carbon provider for the CO production which then serves as fossil reductant is the best known example for such a reductive smelting method. Similar fossil-based processes apply also for the reduction and smelting of lead, copper and tin ores, usually conducted in high-temperature furnaces such as a reverberatory furnace, electric furnace or Outokumpu furnace.

Refining generally refers to thermal operations that target the removal of gangue-related impurities from feedstock. A wide spectrum of methods is used for refining, involving various types of pyrometallurgical processes or electrolytic operations. Pyrometallurgical refining processes are sometimes also referred to as “fire refining”.

In all these process variants, pyrometallurgical processing serves to produce either pure metals, intermediate compounds or alloys, suitable as feedstock for downstream processing. It must be noted that terms such as “pure metals” refer here to a state where the materials can contain substantial amounts of gangue elements and elements that are partitioned from the reductants they had been exposed to. Some of the technique explained above do thus not actually produce the metals themselves but only solute alloys or eutectics (such as pig iron, being a near-eutectic iron-carbon alloy which does not yet have any steel-like properties). This means that the gangue and tramp elements can become inherited to the metal produced either—depending on the mineral type used as feedstock—from the ores or from elemental partitioning between the metals and the reductants, for example the coke. Typical examples of transition metals extracted by pyrometallurgical processes include all the oxides of the less reactive transition elements such as iron, nickel, cobalt, copper, zinc, chromium, tin, and manganese. Aluminum is an exception, due to its strong bond to oxygen, which requires electrolysis to reduce it, Figure 68.

These aspects show that significant differences exist among the pyrometallurgical reduction methods for the production of the different transition metals. These differences are due to the different types of ores used as raw materials for the reduction (e.g., oxides, sulfides or carbonates or mixtures of these minerals); the concentration of the different transition metals in the respective minerals; the gangue elements in the minerals and their partitioning behavior to the target metals; the required enrichment and refinement methods; the kinetics of the corresponding redox relations and the thermodynamic parameters with which the metals to be recovered are bound in the respective ore mixture.

To reach the needed operating temperatures, most pyrometallurgical processes require energy input. This energy is commonly supplied by a combustion process or, less frequently, by electrical heat, for instance though induction, radiation, conduction or plasma. Electrical heating is an important access point for sustainable metallurgy because future processes should be designed in a way to provide heat primarily from sustainable energy sources.

When the underlying redox reaction is exothermic (see thermodynamic details in section 5.1) and if sufficient material is present in the feedstock to sustain the process temperature solely by this exothermic reaction (i.e., without the addition of fuel or electrical heat), the process is referred to as autogenous process. For instance, the pyrometallurgical processing of some sulfidic minerals makes use of the exothermicity of the underlying redox reaction.

It must be noted though that many of the currently studied more sustainable reduction processes (e.g., by using hydrogen as a reductant) are not exothermic over the entire operation range (like in the case of many reductants of fossil origin), Figure 58 and Figure 59. This means that many of the targeted sustainable pyrometallurgical reduction processes require significant external heating (often provided by fuels from fossil origin today, hence, equipped with a high CO_2_ footprint), which is one of the big challenges when designing metallurgical extraction methods with reduced CO_2_ emissions. This important factor must be included in the evaluation of the overall sustainability balance of novel pyrometallurgical processs variants.

**Figure 119:**
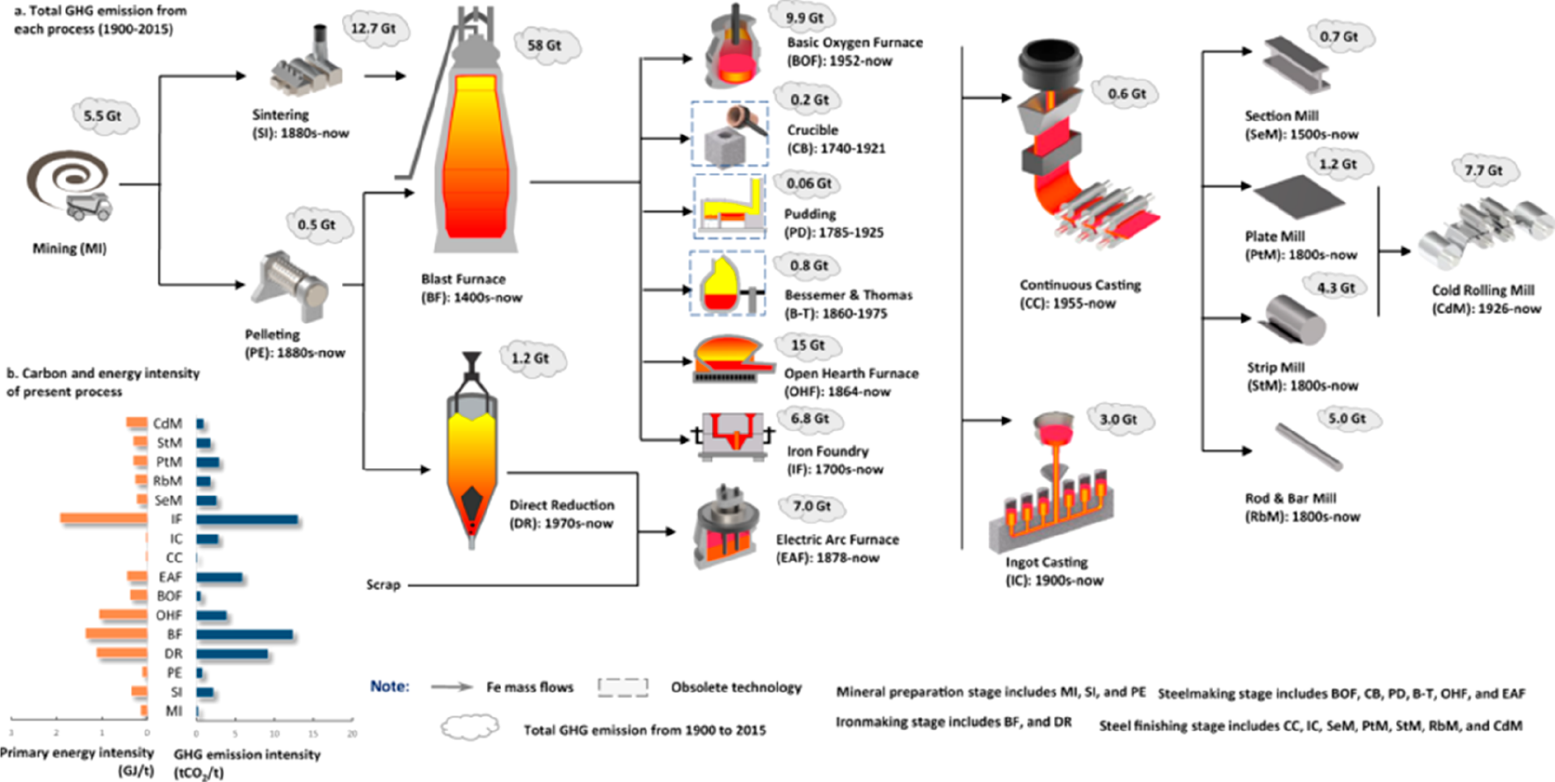
Wang et al.^463^ studied the process-specific origin in greenhouse gas emissions from the traditional steel industry for the years 1900 to 2015. They found that the substantial efficiency improvements, by up to two-thirds for some of the processes, were overcompensated largely by very carbon-intense steel production methods employed in emerging industry regions, so that the total net emissions have stagnated since 1995. (a) Main process steps and total carbon emissions from 1900 to 2015. (b) Energy intensity and carbon intensity level for each process (details are given in the original paper^463^). MI, mining; SI, sintering; PE, pelleting; BF, blast furnace, DR: direct reduction, BOF: basic oxygen furnace, CB: crucible, PD: puddling; B-T, Bessemer & Thomas; OHF, open-hearth furnace; IF, iron foundry; EAF, electric arc furnace; CC, continuous casting; IC, ingot casting; SeM, section mill; PtM, plate mill; StM, strip mill; RbM, rod bar mill; CdM, cold rolling mill. The figure is reproduced from ref (463) with permission. Copyright 2021, Springer Nature.

Iron and steel making stand by far for the highest amounts of greenhouse gas emission and energy consumption so that most of the ensuing sections on emerging sustainability trends in pyrometallurgy are concerned with this material, Table 3. The massive sustainability tasks in this field include massive electrification (provided the electricity comes from renewable sources), the use (green) hydrogen and its carriers as possible reductants for replacing coke and methane, higher use of scrap, waste minimization, and better energy efficiency. Particularly the latter point, viz. efficiency, has significantly improved over the last decades. However, Wang et al.^463^ showed that this improvement in efficiency was overcompensated by the substantially grown total production volume and the market growth in industry regions with carbon-intense steel production. They analyzed the amount and process-specific origin in greenhouse gas emissions from the global steel industry over nearly one century, from 1900 to 2015, using material flow analysis and life cycle assessment. They concluded that the high improvement in process efficiency that had been achieved over the years by permanent process and feedstock improvement, namely up to two-thirds, was offset by a 44-fold increase in total steel production, resulting in a 17-fold net increase in annual emissions. In essence, this means that the steel industry’s decarbonization progress at the global scale has largely stagnated since 1995 mainly due to expanded production in emerging countries with high carbon intensity, Figure 119.

In the traditional iron and steel making industry that operates via reduction of iron ores, the pyrometallurgical process technologies which cover most of the market volume are currently to 70% of the global production volume coke-fueled blast furnaces in conjunction with downstream basic oxygen converter plants and, to a minor fraction, methane-gas-operated direct reduction plants plus electric arc furnaces, Figure 119. The former route (blast furnace and oxygen converter) has by far the highest carbon footprint of all steel manufacturing methods, Figure 37. The trend for the avoidance of carbon during production is pursued mainly by the scrap-based electric arc furnace melting route and the iron-ore-based steel making route with direct reduction plus electric furnace route using natural gas and/or hydrogen as the reducing agent, both process pathways where the use of coal or coke to reduce the iron ore is completely avoided, Figure 41.

The introduction of direct reduction via hydrogen and subsequent melting of the sponge iron in electric arc furnaces would require in future staggering amounts of hydrogen and CO_2_-free electrical energy. Estimates suggest a demand volume of about 100–110 million tonnes of hydrogen every year.

This chapter does not aim to serve as a textbook for pyrometallurgy. Instead it aims at addressing some important aspects that can have a high leverage for improving sustainability in metallurgy and where appropriate new technological and scientific approaches can be identified which qualify as promising research topics, Table 31.

**Table 31:** Pyrometallurgical Methods and Process-Specific Sustainability Challenges

Energy and reductant supply: Less use of fossil-based heating and fossil reductants since they are the main cause of the massive greenhouse gas emissions in the metallurgical sector; transition to carbon-free reductants; efficiency improvement
Feedstock types: Improved methods for the use of scrap instead of/mixed with ores as feedstock; fluidized bed reactor with power oxides instead of sintered pellets; use of low-purity feedstock types
By-products: Less production of dust, fume and off-gas: less use of sulfuric acid, arsenic fumes, NO_*x*_, dioxin, CO_2_
Hazardous by-products: Several purification processes create by-products containing arsenic, cadmium, mercury, lead and zinc; loss of valuable resources; one method’s waste is another method’s feedstock
Non-fossil reductants: Changes in free energy balance upon change in chemical composition of reactants; competition in reaction for use of mixed reductants (e.g., use of methane and hydrogen)
Mineral feedstock: Energy balance for low-grade, chemically less pure mineral feedstock (example: reduction of banded Fe-Si-oxides); transport coefficients as a function of chemical composition of reactants; mobility of reactants in high-component oxides and sulfides; nucleation mechanisms in phase transformations under redox conditions; transport vs nucleation limitation
Thermodynamics of slag: Equilibrium relations between melt and slag
Elemental partitioning coefficients: Partitioning between melt, slag and reductant atmosphere, i.e. which elements partition into slag, vapor and melt
Modeling: Scale bridging modeling of direct reduction (shaft reactor and fluid bed reactor) and plasma-based reduction; multiphysics models that account not only for the chemical reactions but also for mechanics, transport limitations and defects
Kinetics: Diffusion mechanisms during oxidation and reduction
Impurities: Influence of gangue-, scrap-, reductant- or process-related tramp elements on kinetics
Artificial intelligence: Machine learning and text mining in pyrometallurgy method development

#### 7.4.2 Options for Reducing Greenhouse Gas Emissions from Blast Furnaces

Blast furnaces and the associated upstream and downstream operations including the converter plant, sintering and coke plants, etc. are by far the largest producers of greenhouse gas emissions and have the largest energy consumption of all metallurgical aggregates, Figure 119. Steel production stands for about 1/3^rd^ of all industrial greenhouse gas emissions and about 8% of the total energy consumption, Table 3. Therefore, this field deserves the highest attention when it comes to making metallurgy more sustainable.^463^ A number of measures can be and have been taken to improve the steel industry’s overall efficiency,^464^ reduce energy consumption, and mitigate CO_2_ emissions.^465^ While the total efficiency in steel making is already quite high nowadays compared to many other branches of metallurgy, the CO_2_ emissions are still staggering and act as the biggest single source of global warming.^33^ Also, further CO_2_ emission reduction alone by efficiency improvement is approaching its thermodynamic limits, Figure 70.

In the sintering process, iron ore is fired at temperatures around 1000–1300 °C. The recirculation of waste heat offers an option for efficiency and sustainability enhancement in this process step. Difficulties in the dissemination and reuse of waste heat utilization technologies from sintering plants are based on the one hand on high concentrations of pollutants in the waste gas, the required investments for using this waste heat, and the change in product properties, such as changes in grain and in particle size.^466^

Coke dry quenching is another option: in a coke plant, coal is converted into coke at temperatures of 900–1400 °C, after which it is cooled immediately and the heat it carries is mostly lost unused.^465^ In the wet coke cooling process about half of the energy used for coking is not utilized but is lost as water vapor. In dry coke cooling, the coke is cooled with an inert gas such as nitrogen. The gas mixture of nitrogen and other components heats up to about 880 °C. Steam or electricity can be generated via gas purification and a waste heat boiler, allowing up to 90% of the coke heat to be recovered. This process allows about 1,400 MJ per tonne of dry coke to be recovered in the form of steam. This corresponds to about 40% of the energy consumption of current plants. In terms of the oxygen steel production process, this represents potential savings of 0.5 GJ per tonne of oxygen steel, or around 3%. In addition to the energy savings, lower pollutant loads are also an advantage.^159^

Integrated metallurgical plants are largely optimized in terms of energy. However, there is further efficiency potential in optimizing the metallurgical gas network, including modern process controls and sensors. This potential lies mainly in the coordination of production and use of metallurgical gases at the various production plants. The different combustion properties of the metallurgical gases, such as calorific value and adiabatic combustion temperature, must be taken into account when using the gases. For example, the blast furnace gas has a combustion temperature of around 1200 °C and thus cannot be used on rolling mill furnaces, which require a temperature of around 1300 °C. Also, metallurgical gas networks could combine blast furnace, converter and coke oven gas networks in a better way. The optimization of the gas network minimizes flare losses by coordinating production, so that generation and consumption are better aligned. Also the optimal design of storage facilities, valves, and control technology also reduces energy losses.

Blast furnace gas recirculation is another possible measure.^467^ The typical composition of blast furnace gas by volume is about 55% N_2_, 20–22% CO, 20–22% CO_2_, and up to 3% H_2_. During blast furnace gas recirculation, the CO_2_ of the blast furnace gas is first separated. The remaining gas can be heated and fed back into the blast furnace as an additional reducing agent. Compared with conventional blast furnace operation, cold oxygen can be fed via the lower blow mold level instead of hot air. The injection of hot reducing gas into the lower shaft area of the blast furnace is intended to produce a very high degree of pre-reduction of the iron ores before they enter the lower area of the blast furnace. This effect can result in a significant reduction in the Boudouard reaction, which consumes a lot of heat. The Boudouard reaction is the central redox equilibrium between CO_2_ and CO that occurs during the reaction with hot carbon, providing the reductant gas (CO). This approach can lead to a reduction in carbon requirements of around 20%. By using blast furnace gas again in the blast furnace process, around 80% of the energy made available to the energy network of an integrated steel mill today in a conventional blast furnace operation is lost. The energy required to heat the reduction gas must also be taken into account. For a massive CO_2_ reduction with this process variant, final storage of the separated CO_2_ is required. In addition to around 92% CO_2_, the scrubbed gas also contains up to 6% CO and small amounts of H_2_ and N_2_. For final storage, cryogenic treatment is likely to be required for CO_2_ enrichment.

Related approaches that make use of blast furnace gas recirculation have injected oxygen so that the CO gets oxidized into CO_2_. The blast furnace gas is collected and purified. In the process, the CO is split from the CO_2_ and blown into the blast furnace again to react to form CO_2_. With this process the concentration of CO_2_ in the blast furnace gas is increased to such an extent that CO_2_ separation becomes possible. Refeeding the CO back into the blast furnace reduces coke consumption. The potential for CO_2_ emission reduction by this CO accumulation and refeeding approach in the blast furnace is estimated to about 15%.^468^

#### 7.4.3 Hydrogen Injection in Blast Furnaces for Iron Making with Reduced CO_2_ Emissions

**Figure 120:**
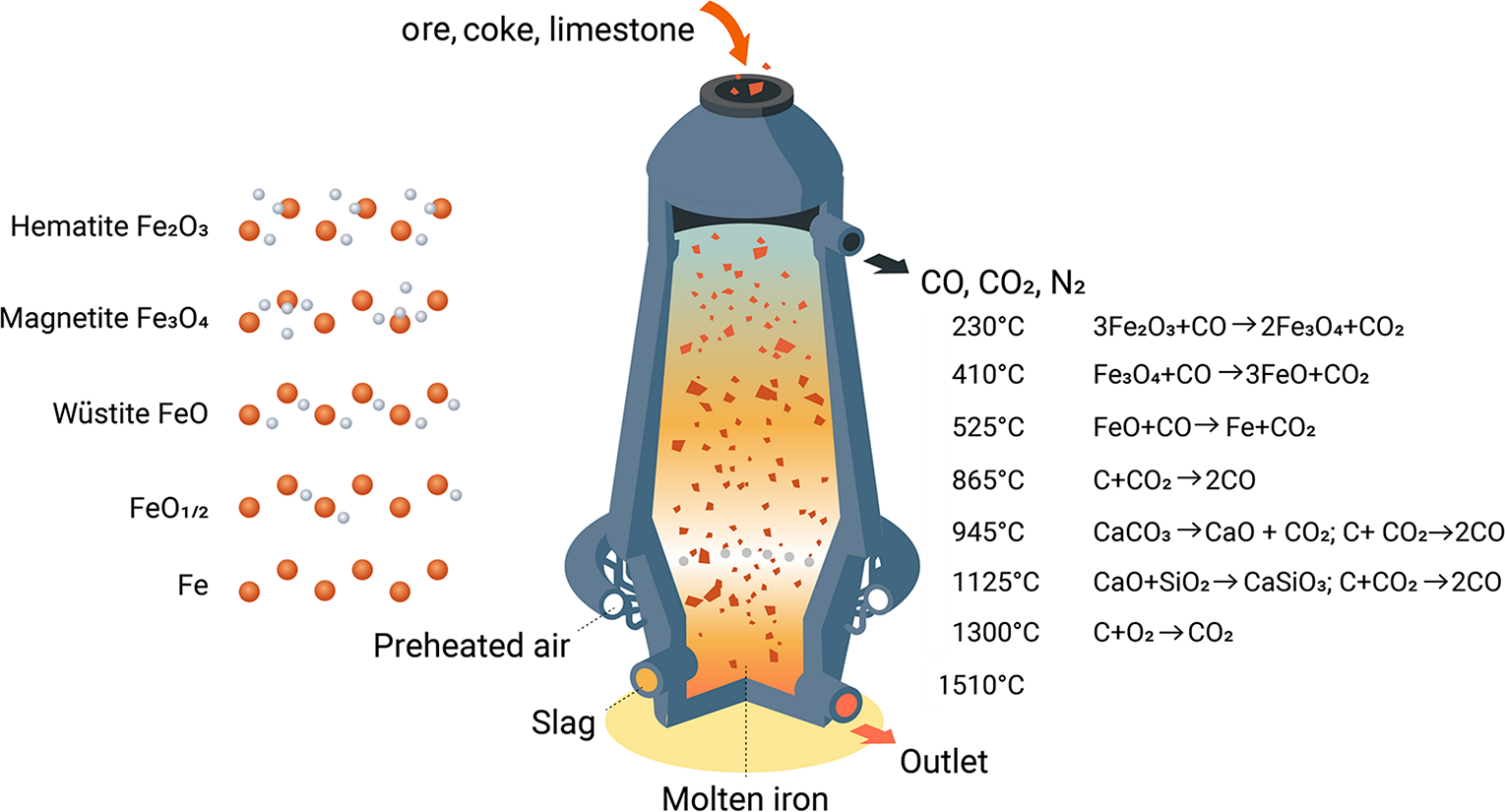
A conventional blast furnace including the locations of the main reaction zones and chemical reactions when operated with fossil reductant.

Iron making is the largest producer of CO_2_ in the industry, underlining that in sustainable metallurgy, size matters. In the traditional blast furnace plus oxygen converter route, which stands for about 70% of the global steel production, CO_2_ emissions amount to about 2 tonnes per tonne of crude steel, produced along the steps coking, sintering, blast furnace, converter and the subsequent processes of casting and forming, Figure 41. Among these, the blast furnace process accounts for the largest share of CO_2_ emissions.^33^ Here, the reduction of iron ores with CO unavoidably produces CO_2_, which is then emitted into the atmosphere. In the blast furnace, liquid pig iron is produced at a temperature of approximately 1500 °C, which has already been largely freed of the rock constituents (gangue) of the iron ores via a slag. The resulting blast furnace slag is usually granulated and used in cement production as a substitute for Portland clinker, leading to considerable CO_2_ savings there. For physical reasons, it is not possible to operate the blast furnace without coke.^331,469,470^ The coke is necessary and cannot be fully replaced in the blast furnace process, as it ensures the maintenance of the permeability in the area of the softening and melting zone of the iron ores in the blast furnace (cohesive zone), Furthermore, the coke enables the drainage in the pig iron and slag rack and it forms a supporting framework for the burden column above the cohesive zone, Figure 120.

In the blast furnace around 500 kg of solid and/or pulverized coke are used to produce 1 tonne of pig iron. Pulverized fossil reductants can be injected into blast furnaces via blow molds in the lower shaft area. The coal dust can serve as additional reducing agent.

Conventional fossil-based blast furnace operations involve a number of separate processes. In the charging process iron ore, coke and other feedstock such as lime are fed into the top of the blast furnace. Often pulverized fossil reductants are additionally blown into the blast furnace via blow molds. The injected coal reacts with oxygen into CO and CO_2_, depending on the exact position of the Boudouard equilibrium in terms of temperature and chemical potential of the reactants, Figure 58, Figure 59, and Figure 120. The reductant percolates upward inside the furnace, transporting also the heat. In the preheating zone inside the furnace, the hot reduction gases rise from below and both heat and dry the ores and the coal. In the indirect reduction zone, carbon monoxide flows up from below and the iron ore is reduced in a chemical reduction with the CO. The oxygen content of the iron ore is reduced as a result. In the direct reduction zone, the mass slides further down in the furnace. Due to the higher temperatures, the carbon there can react directly with the iron oxides in the ore, reducing it further, thus producing the pig iron. In the melting zone, the carbon has accumulated in the iron toward the eutectic point of the Fe-C phase diagram, which drastically reduces its melting point where the near-eutectic pig iron melts, Figure 38. In the product tapping region, the slag and pig iron collect in the lower part of the blast furnace and are released separately. During the gas release process at the upper end of the blast furnace, the remaining reduction gas escapes as so-called blast furnace gas, being essentially a type of exhaust gas. One approach to make existing blast furnaces at least moderately more sustainable is to replace some of the fossil reductants by injected natural gas,^471,472^ polymer waste shredder,^178,453,473^ biomass^49,459,462^ and/or hydrogen.^474^

As the use of hydrogen produces water vapor, unlike pulverized carbon-based reductants which are nowadays typically injected, up to 20% CO_2_ could in a best-scenario case be saved at this point in blast furnace production when counting in stoichiometric terms.^179^ However, this approach has not been scientifically scrutinized in detail yet, and it is also conceivable that some portion of the so injected hydrogen is probably more likely to burn directly at the injection point rather than competing with the CO for the oxide reduction in the upper part of the furnace, i.e., without becoming available as reducing agent for the iron oxide. Several groups have addressed this challenge with corresponding blast furnace simulations.^470,471,475,476^ Furthermore, the injected hydrogen competes with CO, which is provided by the Boudouard reaction and which has the much larger heat of reaction with iron oxide compared to hydrogen and will therefore be the preferred reactant.

On the positive side, though, the advantage of this approach—should it work and should enough green hydrogen be available on the market—lies in the use of existing infrastructures at least for a transition period, as currently about 70% of the crude iron production comes from blast furnaces. This means that even tiny improvements in the efficiency in operating these huge aggregates has a potentially huge leverage toward partial decarburization.

Yet, like in the case of using polymer shredder and biowaste,^178,473^ a rigid framework of coke is still required for percolation of gas and for energy supply, as the reaction between iron oxide and hydrogen is endothermic.

Another challenging aspect that affects all large-scale hydrogen-based reduction methods to some extent is the efficiency of the hydrogen exploitation as reductant. If more than 100 million tonnes of hydrogen were needed annually for the conversion of the entire global steel industry, any corresponding technology must use this raw material extremely efficiently. This means that reactor technologies should be developed that operate close to the stoichiometric limits of the redox reactions and waste as little hydrogen as possible, since this must be produced expensively and laboriously, ideally from electrolysis operated with renewable energy.

The use of organic and fossil shredder reductant in blast furnaces has been done already for quite some time to a certain extent simply due to cost reasons, and it can be seen as standard industrial routine nowadays.^261^ However, this approach has clear limits regarding the availability of renewable biomass in the sufficiently large quantities required in the steel sector.

Another concern is that a commercial incentive to produce biomass as reductant for the steel industry could lead to a shift from food toward biomass production which would be a competition that should be avoided.^461,473^ Finally, it leads to more permanent emissions of CO_2_ into the air, which is also not a sustainable approach. A further aspect that limits the use of organic and polymer shredder in blast furnaces is that a certain skeleton and gas circulation system must be maintained by using coke and pellets. If too much waste material is used in blast furnaces, this condition could no longer be maintained.

Rendering these existing processes more sustainable and less CO_2_ intensive is challenging, as any individual process change usually involves extremely high investment costs. This means that many processes are initially adapted step by step to the use of hydrogen instead of fossil fuels and reducing agents. This also applies in particular to the limited use of hydrogen as an additional reducing agent in the blast furnace.

Depending on availability, future blast furnace operations might use green hydrogen, injection of coal and polymer shredder or a mixture of these reducing agents.^126,331,477^ The main technical challenge is that hydrogen has different reaction kinetics from injected coal. This means that the two substances react at different rates and intensities in the furnace and are accordingly “consumed” at different rates and in different zones of the furnace. Hydrogen also releases more heat than coal—the blow molds are therefore subjected to higher stress. It is therefore studied how various blowing combinations as well as concepts to protect the blast furnace material during operation can help solve this deficit.

Bernasowski et al.^478^ conducted thermodynamic calculations about the influence of hydrogen in the Bosch gas on wüstite stability under blast furnace process conditions. The calculations revealed a possible mechanisms of wüstite reduction and also the role of the water-gas shift reaction and specifically also of hydrogen itself. Nishioka^479^ also studied the hydrogen-based reduction in blast furnace operations. They found that with the injection of gases with high H_2_ concentrations such as coke-oven gas or a reformed version of it, the required input of carbon and the associated output of CO_2_ decreased by about 3% when using such hydrogen-containing gas mixtures. The mutual interplay and competition of fossil and hydrogen reductants in hydrogen injection into blast furnaces was modeled by Nogami et al.,^470,475^ assuming a constant Bosch gas flow rate as well as adiabatic flame and hot metal temperatures. They found that the temperature in the stack was decreased with the increase in hydrogen injection ratio. This resulted in the lowering of the top gas temperature and retarded the reduction of iron oxide, especially the reduction of the magnetite phase. However, the simulation showed that the injection of the hydrogen decreased the coke consumption rate. Although this decrease in coke consumption deteriorated the permeability of the burden materials in the furnace, the pressure drop in the furnace was reduced. Since the molar flow rate of the reducing gas was kept constant, the decrease in the gas density due to the increase in the hydrogen content was mainly considered to lead to the decrease in the pressure drop. The water-gas shift reaction played an important role in the generation of the field of gas composition; thus, this reaction has to be carefully discussed for further utilization of hydrogen in the blast furnace.

**Figure 121:**
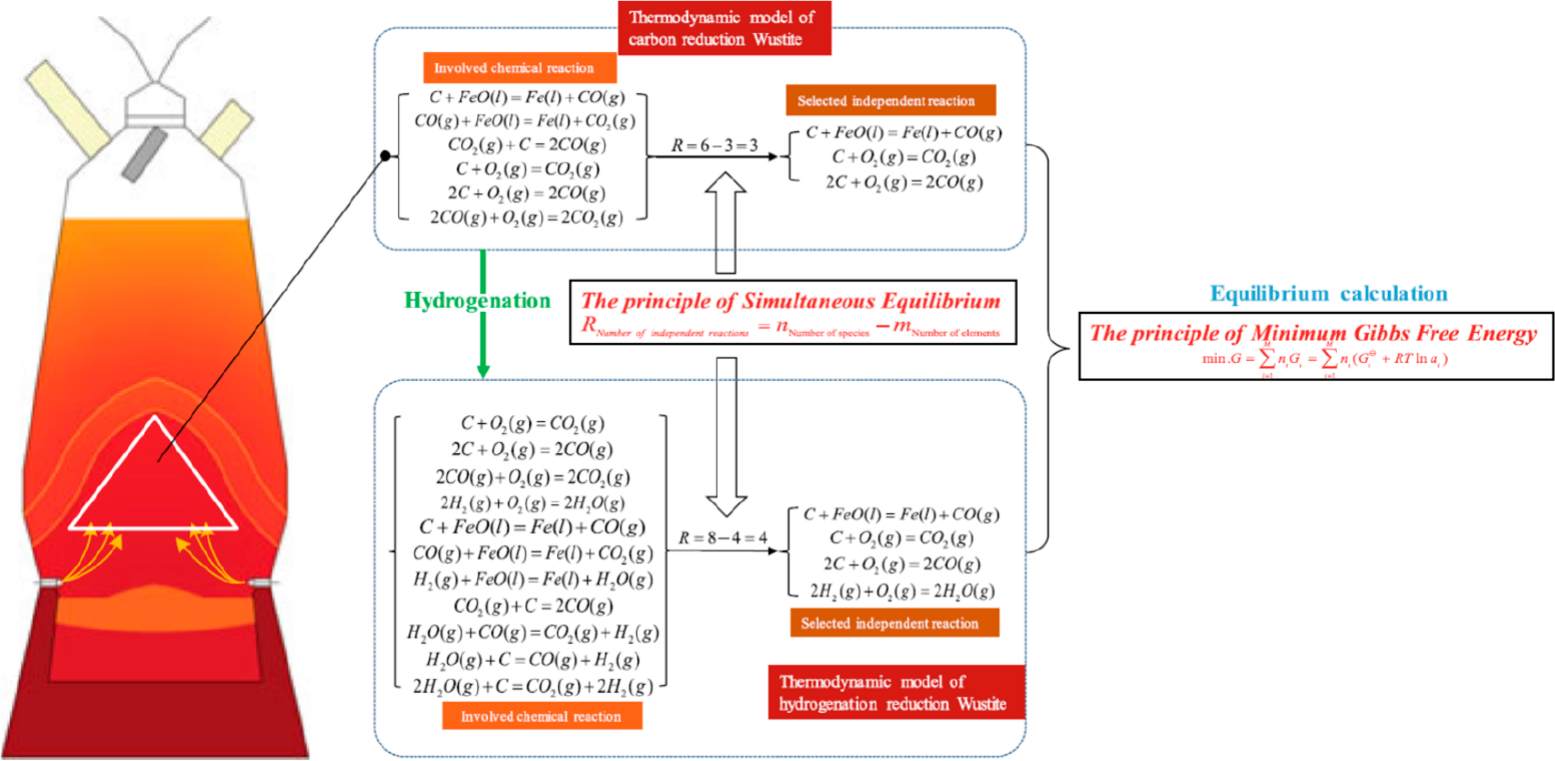
Elementary equilibrium reaction considered in the simulation work of Tang et al.^482^ about the influence and efficiency of hydrogen injection on the reduction processes in the blast furnace from a thermodynamics perspective. The elementary reaction steps considered in the simulation are shown on the right-hand side. The figure is reproduced from ref (482) with permission. Copyright 2021, Springer.

Several groups^480−482^ studied the free energy balance of using hydrogen as co-reductant in the lower portion of the blast furnace via injection. It was reported that the hydrogen plays a crucial role in the thermal balance of the system and the reduction process of wüstite. When the amount of heat supplied by hydrogen injection fell below 25%, the gas utilization ratio increased by injecting hydrogen. In this scenario, wüstite can be completely reduced by carbon, and no water is formed because H_2_ only acts as a transmission medium. The situation becomes totally different when its fraction is above 25%, where then the coexistence of carbon and wüstite could be observed, Figure 121.^482^ A related approach lies in the injection of syngas produced by oxygen-rich gasification of low-carbon waste material into the blast furnace.

Table 32 lists some possible topics for basic research on more sustainable blast furnace operations.

**Table 32:** Some Open Questions for Basic Metallurgical Research Related to Sustainable Blast Furnace Operations

Use of waste materials as fuel sources; injection of any low- or zero-fossil gaseous fuel/reductant such as green methane, hydrogen or ammonia injection into blast furnaces
Use of renewable bio-based reductants (in moderate quantities, to not compete with crop production)
Simulation of hydrogen and mixed gas atmospheres in blast furnace operations
Co-charging of steel scrap
Co-charging of fully or partially direct reduced iron sponge
Charging of pellets instead of sinter
Reuse and recycling of furnace top gas emissions
Reducing carbon emissions through better gas cleaning
Stove oxygen enrichment
Flue gas recycling
Gasification of injectant feedstock, fuels, and reductants for better mixing, higher efficiency and faster kinetics
Mechanism and effectiveness of hydrogen injection; competition between hydrogen-based reduction and CO-based reduction; hydrogen loss due to oxidation prior to reduction reaction

#### 7.4.4 Iron Production by Reducing Calcination of Carbonate Ores

Another approach to reduce iron more sustainably lies in the process of reducing calcination, where carbonate iron ores are reduced by hydrogen into iron. This reducing calcined fine ore is then enriched with iron-containing components by physical separation. Furthermore, the CO- and CO_2_-containing waste gas from the reduction can be converted with hydrogen to methane. The resulting waste heat could be used to heat the reduction reactor via heat coupling.

Reduction calcination could be a way to use siderite as a raw material for iron production. As a result, CO_2_ emissions can be reduced by up to 60% and the amount of reducing agent used can be reduced by up to 33% compared to pig iron production by roasting and CO reduction from carbonate ore. With this reducing calcination approach, the usual roasting step which would be the precondition for a downstream blast furnace use of this raw material prior to reduction is not required, since the CO_2_ of the iron carbonate is split off from the iron in the reduction reactor itself. Here, in contrast to the conventional processes, CO is not used as the main reducing agent, but only H_2_.

The ores containing siderite enter the reduction reactor as fines after being crushed in the reducing calcination process, where the reducing agent flows through them. In comparison to operating with pure fine ore in a blast furnace, there is no specific problem associated with gas permeability because of the relatively low operating temperatures of about 350–425 °C. A magnetic concentration process follows the reduction reactor, separating the nonferrous impurities. H_2_, CO, CO_2_, and CH_4_ are typically present in the gas that leaves the reduction reactor on the other side. It contains the gases CO and CO_2_, which can afterward be methanized with H_2_ to produce CH_4_. Thermal coupling of these two reactors makes economic sense. This is because the heat released in these highly exothermic reactions is of the same order of magnitude as the heat required by the reduction reactor. An additional feed line between the reduction reactor or the reduction reactor and the methanation can supply the excess hydrogen required for the methanation. The entrance through the reduction reactor should be preferred since the reduction benefits from a higher hydrogen content in the reduction gas. The CH_4_ created during the methanation process can subsequently be used for instance as additional feedstock for the natural gas system or it could be fed back into the reduction reactor.

#### 7.4.5 Combined Smelting and Reduction Processes

Smelter reduction techniques are methods for employing gaseous reductants in a reduction shaft and melter gasifier reactor set in order to produce liquid iron. Smelting reduction usually works without the use of conventional metallurgical coke. Feedstock can consist of mixtures of fines, lump ore, pellets and sinter which are charged into the reduction shaft and reduced to approximately 93% of metallized direct reduced iron by a reductant gas in a counterflow configuration.

Non-coking coal enters the melter gasifier unit. The reduction gas usually consists of 95% CO + H_2_ and is produced in the melter gasifier as the result of a coal gasification with oxygen. Flowing from the melter gasifier, the gas is cooled by the recycled process gas to the required reduction gas temperature between 800 and 850 °C. After that it is fed to the reduction shaft where the lump ores or pellets are converted to direct reduced iron. This material is charged into the melter gasifier, where it melts. The tapping procedure, the tapping temperature, and further processing of the hot metal are the same as those for the blast furnace.

The main benefits of the combined smelting and reduction processes are the possible use of a wide range of iron ore types; the elimination of the environmentally very harmful coke process; high operational flexibility of the plant; flexible feedstock choice; lower hot metal production expenditures of up to 20% in comparison with blast furnaces of similar production capacity; satisfying quality of the produced iron; and lower greenhouse gas emissions compared to conventional blast furnace operations. The reduction plus smelting technology thus competes with coke-based blast furnace reduction and can supply raw materials to a downstream conventional oxygen converter. Similar to the blast furnace, reduction smelting yields liquid pig iron. The so-called “Corex” method, which works with iron ore pellets, was the first commercial approach in this reactor category. It is realized in a two reactor setup, consisting of the static bed shaft reduction reactor and the melter gasifier unit. Iron oxide feedstock in the form of lump ore, sintered pellets, or mixtures thereof is at first fed into the static shaft reduction reactor, where it is (partially) reduced to direct reduced iron by the reduction gas in a counterflow operation. This material is then charged into the melter gasifier reactor for smelting. The “Finex” process, which uses fine ores and non-coking coal, is a further method in this double reactor process category. It charges the fine iron oxide particles into a fluidized-bed reactor cascade, where the ore is reduced into iron powder by utilizing a reduction gas that is produced from the gasification of coal in the melter gasifier unit. The reduction product is then fed into the melter gasifier as in the Corex approach. In either case, the main goal of the smelter reduction methods is to increase the efficiency of producing iron with an improved carbon footprint by removing the cost- and CO_2_-intensive pellet and coke production steps that are otherwise—for the case of conventional iron making—placed before the actual blast furnace charging.

Compared to the recent progress in hydrogen-based direct reduction, however, the currently used version of the smelting reduction method must be seen as a less sustainable approach, as it operates with coal—a harmful fossil carbon carrier—instead of using renewable reductants and fuels. Feeding green hydrogen as additional reductant into such aggregates might, however, be an option that is worth being studied. Table 33 lists some possible topics for basic research on more sustainable combined smelting and reduction operations.

**Table 33:** Some Open Questions for Basic Metallurgical Research Related to Combined Smelting and Reduction Operations

Co-injection of low- or zero-fossil gaseous fuel/reductant such as methane, hydrogen or ammonia
Co-feeding of renewable biomass reductants
Co-feeding of other hydrogen vectors
Co-charging of steel scrap
Co-charging of fully or partially direct reduced iron sponge
Co-charging of pellets together with lump ore, fine ore, or sinter
Reuse and recycling of furnace gas emissions
Oxygen enrichment
Flue gas recycling
Gasification of injectant feedstock, fuels, and reductants for better mixing, higher efficiency and faster kinetics
Mechanism and effectiveness of hydrogen injection; competition between hydrogen-based reduction and CO-based reduction; hydrogen loss due to oxidation prior to reduction reaction

#### 7.4.6 Hydrogen-Based Direct Reduction of Iron Oxides in Static (Shaft) and Dynamic (Fluidized Bed) Reactors

**Figure 122:**
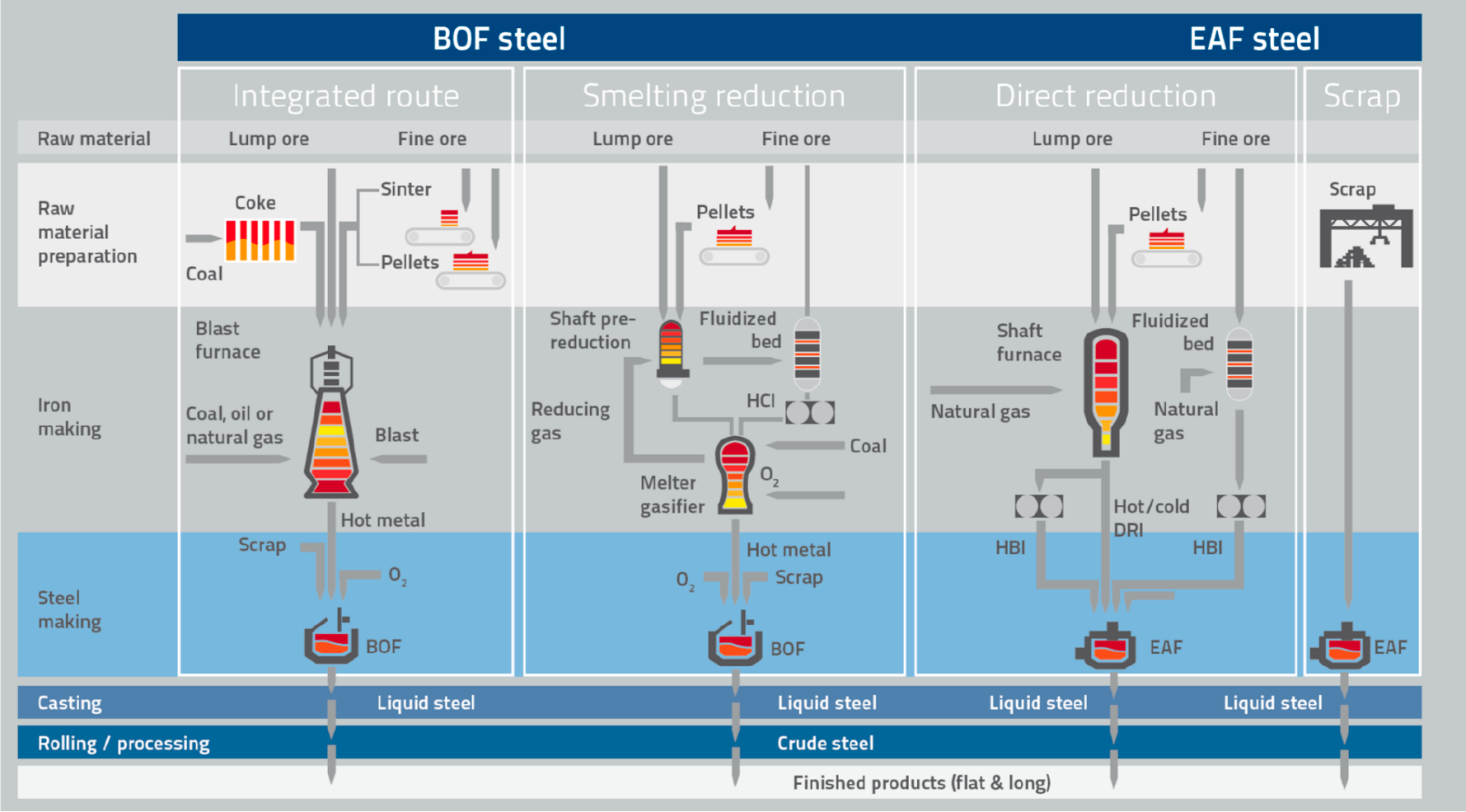
Overview over the different principles and process pathways for conventional shaft furnace reduction, smelting reduction, direct reduction and scrap-based synthesis routes.^241^ The currently mainly pursued process variants for a fossil-free iron production in industry are the direct reduction of sintered pellets in shaft reactors where hydrogen can be used instead or together with methane and fluidized bed reactors in which the less costly iron ore fines can be used as oxide feedstock.^128,397^ Also biomass can be used—instead of coal or methane gas—as additional reductant feedstock.^462^ One has to note that this overview diagram places focus on synthesis pathways with high TRL level (TRL, technology readiness level). It does not yet contain the recently matured liquid oxide smelting reduction methods which can be realized through hydrogen-based plasma operations in electric arc furnaces (plasma-winning of iron) and it does also not include the fused salt electrolysis process (electrowinning of iron). BOF, basic oxygen converter; EAF, electric arc furnace. Figure is reproduced from ref (484) with permission. Copyright 2021, EU Open Data and Reports (PU, public).

**Figure 123:**
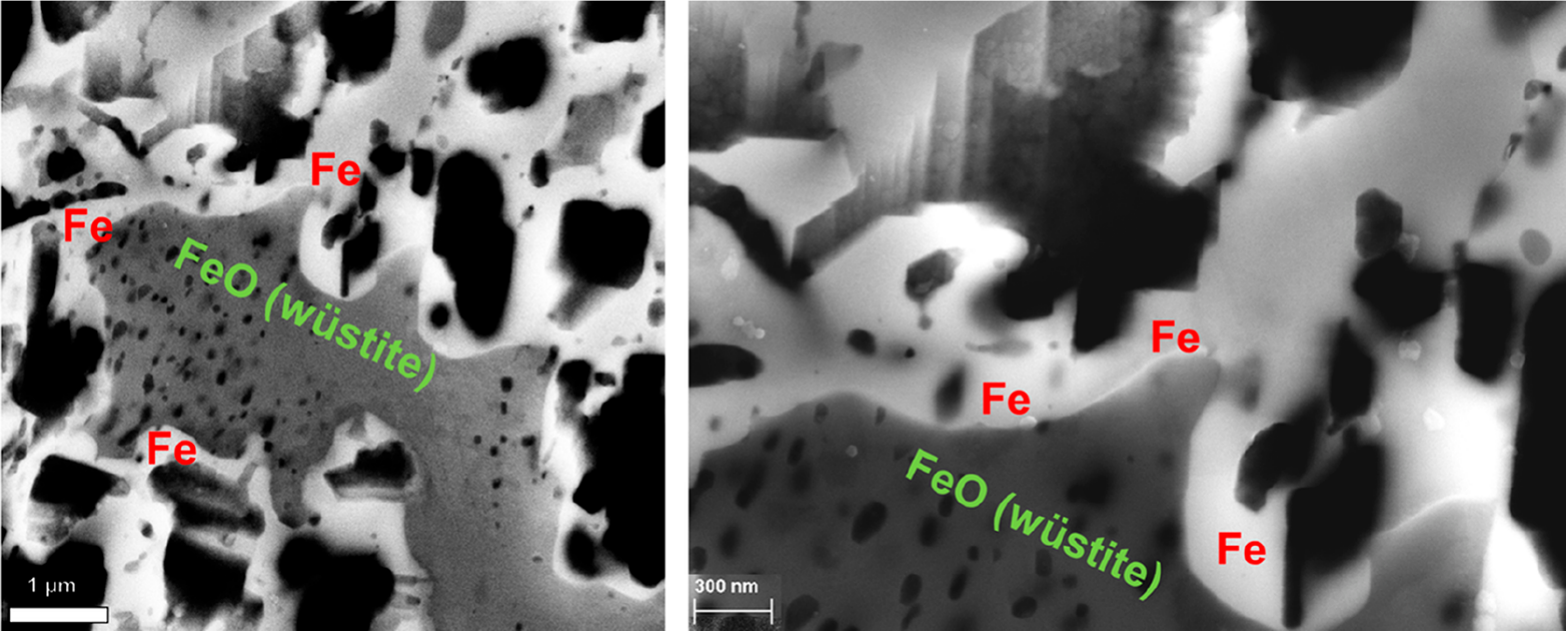
Development of the porous structure of a direct reduced hematite iron ore pellet at 700 °C under pure hydrogen reduction conditions^241^ (see also Figure 84 and Figure 85). The images show microstructure patches taken by scanning electron microscopy of a cross section through a partially reduced hematite pellet after hydrogen-based direct reduction at 700 °C in the final stage, where wüstite transforms into iron. The figure is reproduced in modified form with permission from ref (241). Copyright 2021, Elsevier.

Direct reduction techniques for sustainable iron making include processes in which solid iron oxide is exposed to a gaseous reducing agent, Figure 41.^159,313^ This can be done in the form of heated solid oxide lumps such as pellets in static shaft reactors or in the form of fluidized bed or flow reactors in which fine oxide powders are exposed to the reductant, Figure 122. The reduction product from the more common static shaft reactors is referred to as direct reduced sponge iron, owing to its porous inner structure, Figure 84, Figure 85, and Figure 123.^303,483^ The material produced from direct reduction furnaces (which currently operate on syngas and methane) reaches a metallization of 85–95%. The reduction product is very pyrophoric; i.e., it tends to reoxidize during transport and handling, due to its high surface-to-volume ratio.^314^ For this reason, the sponge iron is after direct reduction usually compacted into a form referred to as hot briquetted iron for safe transport and storage. It is mostly melted in electric arc furnaces, where it is used as a substitute and/or in addition to scrap, Figure 122. Direct reduction with methane as reductant is already widely used in regions with low-cost natural gas resources. Coal or other organic carbon carriers can be used as further reducing agents, yet at the costs of an increased CO_2_ footprint.

Different types of direct reduction process principles can be distinguished. On the one hand there are static shaft reactors using natural gas; rotary kilns using coal; fluidized bed reactors (sometimes with several subsequent furnace units); and rotary hearth processes using coal as reducing agent and gas as fuel, Table 34.

**Table 34:** Main Existing Process Technologies for the Direct Reduction of Iron Oxide Ores in Pelletized, Lump or Powder Form in Static or Dynamic Reactor Operation^a^

Technology	Associated research topics when using non-fossil reductants
Shaft furnaces based on exposing pellets to a reducing gas atmosphere	Shaft furnaces operating with non-fossil reduction gas; the most widely used process is the MIDREX process (with worldwide 200 plants with a capacity of about 45 million tonnes per year of direct reduced iron produced)
Coal-based rotary kilns	These furnaces reduce lumpy iron ore, pellets or fine ores with coal in a rotary kiln; capacity of such rotary kilns is limited by the length and diameter of the kiln and by the mechanical stability of the oven
Fluidized bed reactors	Fluidized bed plants can operate based on (sustainable) non-fossil reduction gas or on coal; they allow, in particular, the use of fine ores; use of gas-based fluidized beds; a typical industry process is the Circored process, which uses natural gas to directly reduce iron ore; this process is a direct reduction process based on the integrated gasification of coal; the process can also operate with hydrogen, for example extracted from natural gas in a reformer or from sustainable water splitting to produce direct reduced iron; the sponge iron can be hot charged into an electric arc furnace or can be hot briquetted for transport
Rotary hearth process	Rotary hearth process (coal as reducing agent, natural gas as fuel); use of fine ores is possible

a All concepts can, in principle, be combined with the use of hydrogen or ammonia as (additional) reductant.

**Figure 124:**
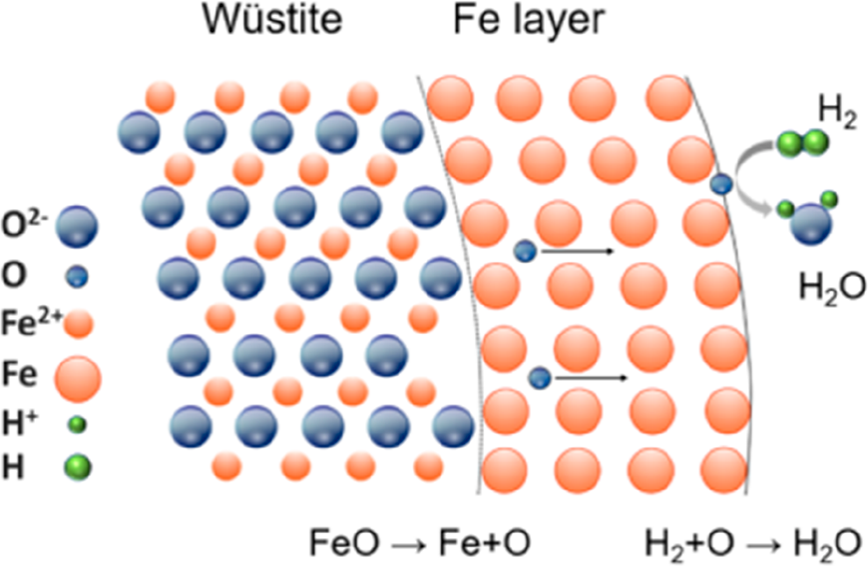
Some of the transport and redox processes involved during hydrogen-based direct reduction, where after the first surface reduction step a thin iron layer forms so that the oxygen must diffuse either through this solid material or through cracks and other defects that form due to the volume mismatch between the two phases (iron and oxide).^241^ See also Figure 84, Figure 85, and Figure 123. The figure is reproduced in modified form with permission from ref (241). Copyright 2021, Elsevier.

The common feature of all direct solid-state reduction processes is that there is an interfacially dominated redox reaction between the solid mineral and the gaseous reducing agent and the necessity for solid-state diffusion. This applies also for cases where hydrogen (or a hydrogen vector gas) instead of/or together with methane is used as a reductant, Figure 124. The use of methane as a reducing agent in direct reduction has a 50–75% improved CO_2_ emission balance compared to the blast furnace plus oxygen converter route (the latter route with 2 tonnes of CO_2_ emitted per 1 tonne of metal produced). In the long run, a reduction with sustainably produced hydrogen (or other hydrogen-providing sustainable reductant gas mixtures) instead of methane would no longer produce any CO_2_ during the reduction (but only in the downstream electric arc furnace melting of the sponge iron, when using graphite electrodes). Therefore, currently the main research priorities in the area of direct reduction with regard to the question of sustainable steel production are concerned with the mechanisms of hydrogen-based direct reduction. Since the early 1970s, natural gas rich in hydrogen and other hydrogen-rich gases have been already employed as a reducing agent in the direct reduction of iron ores.

Coke oven gas is a currently employed alternative for these hydrogen-rich reducing gases. This mixed reductant gas is produced by dry distilling of coal through pyrolysis. All integrated steel companies have access to this mixed gas as a natural resource. The calorific value of the purified coke oven gas is 15.5–18.9 MJ m^–3^ (4.5 kWh m^–3^), which is roughly half that of natural gas. During the coking process, a lot of gaseous materials are created. Depending on the coking plant and the feed coal, the purified coke oven gas typically contains the following components: 55% H_2_, 25% CH_4_, 10% N_2_, and 5% CO. Additionally, the raw gas contains trace amounts of higher hydrocarbons, ammonia, hydrogen sulfide, carbon dioxide, and aromatics, all of which are typically eliminated before being used further.

**Figure 125:**
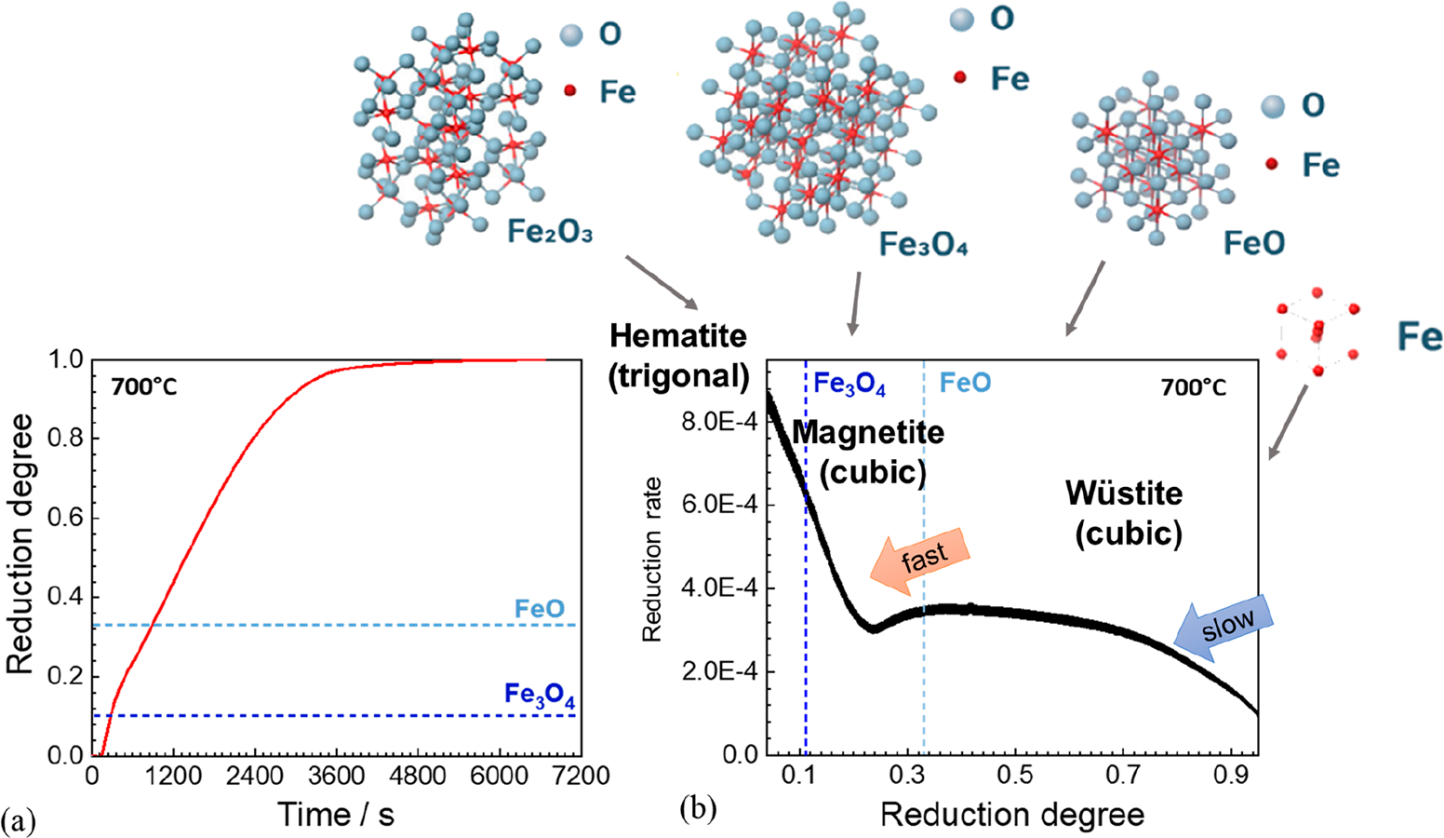
Reduction kinetics of direct reduction hematite pellets at 700 °C under pure hydrogen at a flow rate of 30 liter per hour, measured by using thermogravity analysis. (a) Mass change and temperature versus time. (b) Reduction rate versus reduction degree. The end of the abrupt slope between the 0.11 and 0.15 reduction degrees marks the end of the first stage of the reduction. It is characterized by the fast transition from hematite (Fe_2_O_3_) to magnetite (Fe_3_O_4_), and the subsequent stage of decelerating reduction rates roughly ends at the local minimum of ∼0.23, indicating the Fe_3_O_4_ to FeO (wüstite) transition regime. The further reduction of the FeO into bcc Fe becomes then the rate-limiting process. The figure is reproduced in modified form with permission from ref (241) Copyright 2021, Elsevier.

The advantage of the direct reduction in shaft furnaces is the availability of an existing scalable industry technology for these reactors that have up to now primarily been operated with CH_4_ as reductant.^164,242,330,485,486^ Also, several studies on the reduction kinetics have been published with using H_2_ as reductant.^241,242,277,301,397,487−490^ The disadvantage of the direct reduction method, whether using methane or hydrogen, is the relatively slow reduction kinetics. The reason for this is that this process is a solid-state reaction in which the (relatively fast) in-bound hydrogen diffusion and, above all, the (sluggish) outbound oxygen diffusion occur through the solid oxide and through the evolving iron shell that surrounds the inner oxide cores, Figure 124. These inner transport processes are relatively sluggish because of the small associated diffusion coefficients of both species in these two phases, which makes the overall process relatively slow, especially during the last part of the reduction, where the remaining FeO (wüstite) is transformed into iron. Also, due to this core (FeO)−shell (Fe)-related transport problem and the unclear role of gangue elements which provide in part very stable (and thus hard to reduce) oxide states, the process is often limited to moderate metallization degrees of about 85–95%, Figure 125.

For all direct reduction processes, it must be considered, that—although the iron sponge is a largely reduced iron product—it accumulates all gangue elements in the solid state from the iron ores. On the other hand, unlike in coke-based blast furnace reduction, there seems to be only little or even no partitioning of harmful elements from the reductant into the iron, so that the direct reduction material maps essentially the purity of the oxide feedstock it stems from. Depending on the respective reduction and feedstock scenarios, the CO_2_ emissions of externally sourced materials, such as iron ore pellets or hydrogen production, must also be taken into account in the overall CO_2_ balancing.

Besides shaft reactors, also fluidized bed reactors are considered as potentially viable large-scale process options for hydrogen-based reduction.^334,335,491−494^ In such furnaces, oxidic solid fines are exposed to reducing gases or reducing powder mixtures. In fluidized bed reactors, the key benefits of both fluid process engineering and chemical reaction engineering are combined. These benefits include frequent particle–particle and particle–wall collisions, high relative velocities between the continuous fluids and the dispersed solid phase, and continuous and intensive mixing of the particles, which can result in very high overall efficiency of such processes. These fluidized bed plants are increasingly being explored as a method with high reaction kinetics for the reduction of finely distributed and dispersed iron ore particles. Current research is here focused particularly on the reaction kinetics of the reduction of individual fine iron ores with different reaction gas mixtures including also hydrogen.

In one type of reactor, a modified version of the so-called “Finex” process, a reducing gas made of a mixture of H_2_ and CO stirs up the fine ore at a temperature of roughly 800 °C. The ore particles are transformed into tiny sponge iron pieces in a sequential array consisting of four reactor vessels. These are fed into a melter gasifier after being squeezed by rollers into larger bits. The process is thus chemically related to the lower level reduction mechanisms occurring in a blast furnace. The fluidized bed reactor needs to be heated to temperatures above 2,000 °C, which are produced by the combustion of gasified coal with oxygen. The resulting mixture of carbon monoxide and hydrogen is then released into the reactor as a reducing gas. The export gas used to fuel for instance an energy generating plant is a valuable by-product. Similar to a blast furnace, a melter gasifier can be used to extract iron and slag.

Table 35 lists some possible topics for basic research on direct reduction by use of non-fossil and sustainable reductants.

**Table 35:** Opportunities for Basic Metallurgical Research Related to the Use of Non-fossil and Sustainable Reductants in Direct Reduction, Considering Both Shaft Reactor and Fluidized Bed Reactor Concepts

Direct reduction efficiency in terms of energy and hydrogen consumption for different types of direct reactor design
Effect of temperature and hydrogen partial pressure in hydrogen-based direct reduction
Solid-state diffusion mechanisms (of oxygen, hydrogen and iron), phase nucleation and growth mechanisms
Principles of phase transformation under chemically driven boundary and redox conditions
The role of high mechanical stresses on reduction and transformation kinetics and on transport mechanisms in direct reduction
Role of pellet microstructure, mechanics (internal stresses) and porosity inherited from sintering and evolving during reduction
Oxide sticking and dendrite formation that can lead to unwanted iron particle sintering processes
Influence of mixed reduction gases
Effects of mixed mineral and pellet feedstock
Effect of mineral and pellet purity
Effect of gangue content
Catalysis effects in direct reaction redox reactions
Chemo-mechanical coupling in solid–gas direct reduction reactions
Multiscale simulation of pellet reduction and of complete reactors
Size dependence of pellet reduction aspects
Translation of mechanisms into predictive and physics-based simulation and multiscale simulation
Coupled thermodynamic, kinetic and gas dynamics models for solid–gas direct reduction reactions and the associated multiscale, multiphysics solvers
Size effects and gradients of all solid feedstock ingredients
Friction and abrasion effects among oxide particles in direct reduction
Influence of less pure feedstock and reductant mixtures
Differences among sustainable reductants that can be synthesized by using green hydrogen
Differences in degree and rate of metallization for different parameters and reactor types
Removal mechanisms of oxygen and water; water storage in pellets; reoxidation effects
Porosity and pore percolation evolution during reduction
Roles of pellet deformation, micromechanics and fracture mechanics during reduction
Effect of plasma on solid-state reduction
Effect of reductant partial pressure and temperature on reduction kinetics and metallization
Iron oxide feedstock types and role of chemistry of oxides
Roles of different reductants (H_2_, CH_4_, biomass, LOHC, power-to-reductant, etc.)
Reduction principles and thermodynamic energy balance of different ore–reductant pairings
Role of the dispersion of oxide fines and role of reactor flow and percolation conditions
Role of slag formation and impurities

#### 7.4.7 Flash Iron Making

Flash iron making is another variant of the direct reduction method. It can make use of iron oxide fines or finely dispersed iron ore concentrates which require only limited pretreatment. The oxides are reduced in a gas–solid flash reaction using gaseous fuels and reductants.^471,495,496^

The reduction can be carried out with both methane, hydrogen, or corresponding reduction gas mixtures such as syngas. This qualifies this process as a potentially sustainable reduction method that can take benefit from variable sustainable reductants that can help reduce CO_2_ emissions. The process corresponds in principle to a high-temperature direct reduction approach, conducted on conventional fine oxidic ore feedstock. It is likely to be scalable to large capacities, although only laboratory-scale operations have been conducted so far.

Due to the concentrate particles’ fineness and the typically high reaction temperatures (usually at temperature ranges between 1200 and 1350 °C), a very rapid reaction rate can be achieved, leading to extremely fast reduction times.

Like in fluidized bed reactor methods, a specific advantage of flash iron making is its capability to process iron ore fines, a process which allows circumventing the costly and energy-intense sintering stage. Like for other direct reduced iron products, the reduced powder material can be (co-)charged into the usual downstream electric arc furnace or even as a co-feed into the classical basic oxygen converter processes (instead of or together with cooling scrap).

The underlying microscopic reduction and melting processes are not only used in a stand-alone flash reduction process, but they can also be exploited and used in the smelting reduction method, where the iron ore can be injected into a cyclone furnace, where the particles—when guided by a well-controlled gas suspension injection stream—are partially reduced and melted. In this approach it was observed that the iron oxide fines that are injected into the smelting cyclone furnace are not fully reduced.^497^ A recently suggested process by Sohn^471,495,496^ targets a stand-alone operation to full reduction of such fines at temperatures in excess of 1400 °C.

Chen et al.^497^ wrote an overview article about high-temperature (above 1200 °C) flash-type reduction methods. They found that the reduction of iron oxide particles in suspension proceeded via thermal decomposition of hematite during suspension reduction;^497^ that the rate-determining constants of iron oxide particles were between 10^–2^ per second and 1 per second so that particles get reduced within seconds; and that melting of particles is accompanied with retardation of the suspension reduction process. The reduction of the individual iron ore fine particles was not observed as the rate-limiting mechanism at these high temperatures, according to available kinetic data.^496,498^ Chen et al.^497^ concluded that the reduction kinetics of suspended iron oxides during the flash exposure followed an unreacted core shrinkage model. The authors also discussed the gangue content in the minerals, which can affect the transformation and kinetics during reduction, as also observed by other authors.^241^ Several papers concerned with flash reduction also compared the phase transformation mechanisms and kinetics as well as the effects of gangue elements on the reduction rate at high temperatures above 1200 °C with static iron oxide reduction.^496,497^

#### 7.4.8 Scrap Melting under Nonreducing Atmospheres Using Electric Arc Furnaces

Electric arc furnaces are used to melt scrap, and depending on the charged material, they may also employ chemical energy as a secondary energy source. For example, the technique is well-established as a standard technology for commonly used carbon-based steels and corrosion-resistant stainless steels, both of which are employed, for example, in the construction industry.

The scrap used in such aggregates comes mainly from the two source types “old” scrap (vehicles, buildings, machinery, etc.) and “new” scrap (industrial and in-production scrap). The latter type includes scrap from steel making, forming, cutting, stamping, cropping, etc. and has usually a well-defined chemical composition which old scrap does usually not always have. Details of the scrap types were shown in Table 21. The graphite electrodes provide the access to the electricity in these furnaces. The scrap is melted by an electric arc that the electrodes produce with it, as a counter-electrode. During the process, the (graphite) electrodes are worn down gradually, thereby producing carbon dioxide. Oxygen or fossil fuel carriers are two ways that chemical energy and reductants can be introduced into the process. This process variant will be covered in a different section because these compounds are transformed into a plasma state in the electric arc.

**Figure 126:**
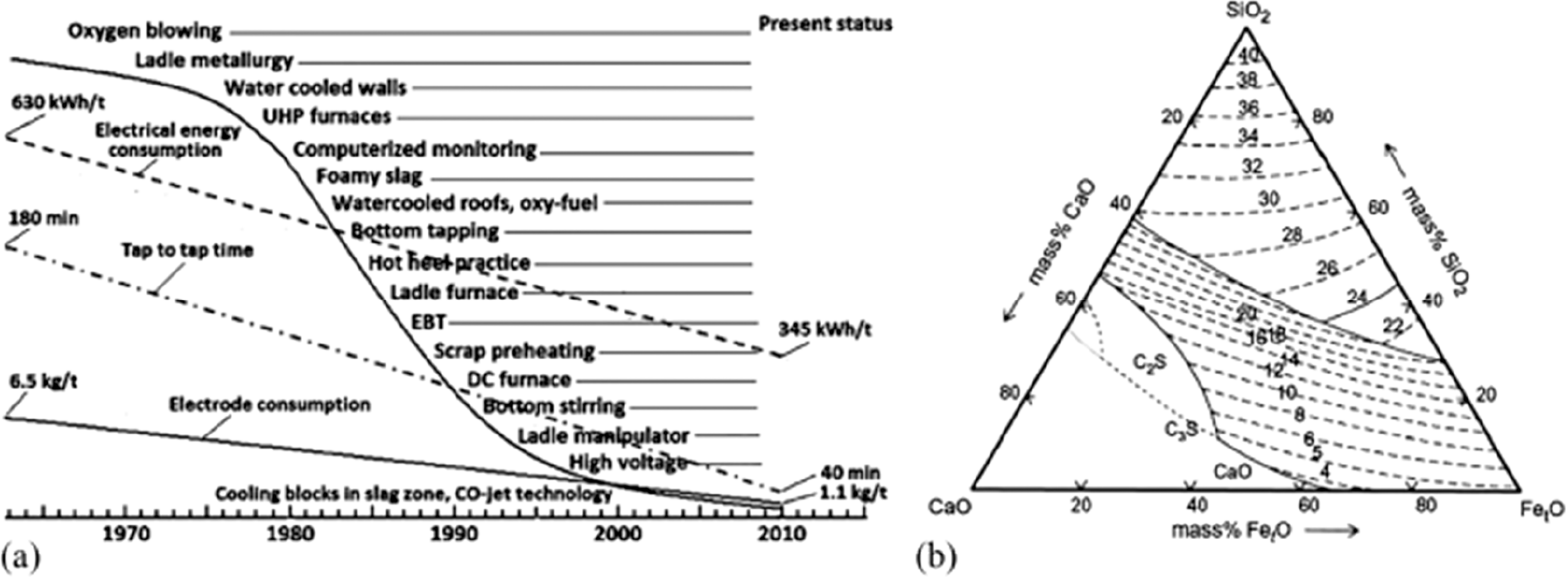
(a) Engineering trends in electric arc furnace technology since 1965. (b) Isothermal cross section of the CaO-SiO_2_-FeO diagram, relevant for slag production in electric arc furnaces, displaying the MgO saturation lines in the liquid pool.

**Figure 127:**
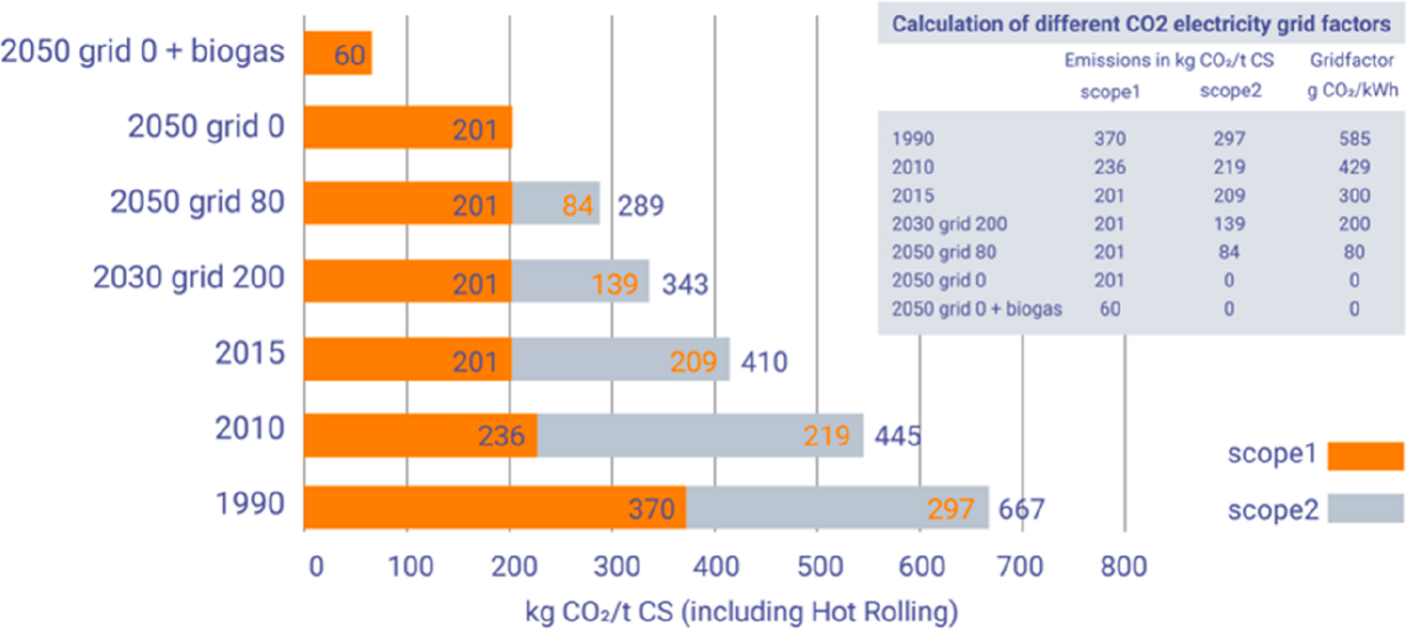
Influence of fuel and energy sources used for secondary steel synthesis via the electric arc furnace route including the subsequent hot rolling process on the resulting CO_2_ emissions. Data have been summarized from refs (397, 463, and 499). The diagram includes the scope 1 and the scope 2 emissions. Scope 1 emissions cover only the direct emissions from the steel manufacturing plant while scope 2 data include the indirect emissions caused by the generation of the purchased electricity, steam, heating and cooling consumed during the secondary steel manufacturing (see also Figure 5). CS, crude steel.

Furnaces used in the production of steel typically use 500–1000 kVA of power per tonne of furnace capacity. Most aggregates operate at a maximum electrical power input of 0.75–0.85 MW per tonne of furnace capacity, depending on the quality of the transformer. The largest electric arc furnaces can therefore produce as much power as 500–700 high-end electric cars, to provide a reference of the immense power operating in such processes. The electric energy usage with 100% scrap charge in the newest generation of aggregates can be as low as 350 kWh per tonne of metal produced, Figure 126. The highest leverage for making secondary synthesis by electric arc furnace melting more sustainable lies obviously in the use of renewable electrical energy, Figure 127.

The high ratio of scrap used as feedstock for this process can translate to high contamination content, depending on scrap quality, and thus, strict scrap specifications apply, Table 21. A related problem is the introduction of dissolved gases such as nitrogen, hydrogen and oxygen into the alloys.

As with the production of any metallic alloy based on using scrap, some issues can be solved by adding extra charges of metal from primary production, called hot metal charging in this instance. However, this strategy frequently results in unwanted carbon intrusion from primary production, which is either difficult to decrease and remove in such aggregates or necessitates additional process stages for introducing oxygen.

A wide range of alloys, including the majority of stainless steels, construction steels, microalloyed and ultralow carbon steels, line-pipe steels, etc., are nowadays produced using electric arc furnaces.

Important research questions to improve the sustainability of this technique, which will rapidly gain momentum owing to the increasing global scrap volume (Figure 97), are technologies with less electrode oxidation, improved linings, higher mechanical electrode stability (increased electrode fracture toughness), scrap preheating, improved slag formation, improved bath dynamics, additional oxygen injection for decarburization, chemical reactions between slag and metal, and slag foaming.

The latter feature, i.e. the formation of a highly foamed slag on top of the melt, is desirable because it prevents the liquid steel from reacting with the environment and boosts electrical efficiency by burying the electrode arc. This feature can be obtained by gas injection. This increases the furnace’s thermal efficiency and enables it to run at higher voltages without harming its walls and roof. Aside from preventing N_2_ from being exposed to the arc, where it might dissociate and enter the steel, burying the arc also helps. This is significant because at a temperature of about 1600 °C, nitrogen has the highest solubility in steel.

One way to improve the sustainability of this process lies in better utilizing electric arc furnace waste heat, such as for power generation. In electric arc furnace operations, a lot of waste heat is released. Scrap can for instance be pre-heated using this waste heat. However, because of the impurities in the scrap, this can result in a rise in the creation of pollutants like dioxins and furans in the waste gas. As a result, the energy advantage would be lost and post-combustion at temperatures above 800 °C would be required to decompose these gas components. In principle, it is worth noting that only a portion of the CO_2_ emissions in the scrap-based electric furnace pathway are caused directly by the manufacturing operations. Since the electric furnace route itself does not produce any energetically useful process gases, the majority of the CO_2_ emissions originate from the CO_2_ burden of the externally obtained electrical energy for the operations (scope II emissions, Table 1). The CO_2_ emission of this route is about 400−450 kg/t of crude steel with a CO_2_ load of the electrical energy of about 300 g/kWh.

The general advantage of scrap-based secondary synthesis is that markets for steel scrap will grow strongly in the coming decades and thus significantly increase the availability of this sustainable raw material, Figure 36. It is foreseeable that the market share of steel scrap could be up to two-thirds of the total market by about 2050. However, the longevity of many steel products, especially those in buildings, is still a difficult variable to estimate. The melting of the scrap via electric arc furnaces allows immediate use of sustainable electrical energy and thus makes the overall process potentially very sustainable. In this respect, the production of steel from house and infrastructure scrap is certainly one of the most sensible methods to produce green steels. A significant challenge of the increasing supply of sustainable steel produced from scrap alone is that it gradually accumulates undesirable tramp elements. Some of these undesirable chemical ingredients can be removed via the slag, but some others remain and accumulate in the molten iron. Some of these tramp elements can have a significant impact on the properties of the steels produced. At present, the increasing intrusion of copper is of particular importance here because it can no longer be removed from the steel by current metallurgical processes, but at the same time it can also significantly change its properties, mostly negatively, in the form of liquid metal embrittlement. Another aspect is the increasing diversification of steel grades, with a significantly wider range of different chemical compositions compared to conventional iron-carbon steels used in construction. This will require a chemical-grade-specific separation of the scrap in the future, similar to what is already the case today with aluminum alloys and with stainless steels. Table 36 lists some topics for basic research on more sustainable electric arc furnace melting of scrap.

**Table 36:** Some Open Questions for Basic Metallurgical Research Related to More Sustainable Electric Arc Furnace Melting of Scrap

Reduced electric power consumption and less use of fossil co-fuel
Inert electrodes; less electrode oxidation, higher mechanical electrode stability (increase in the electrode’s fracture toughness)
Recuperative scrap preheating
Slag foaming
Improved inert and mechanically stable refractory materials
Secondary synthesis: removal of copper, tin and zinc from scrap-based steel melts; alloy-specific scrap sorting; science of “dirty alloys” (study of effects associated with higher impurity element content)

#### 7.4.9 Sustainability Aspects in Pyrometallurgical Refining of Precious Metals

The ever-increasing complexity of advanced electronic and electrical equipment and the closely integrated use of multiple metallic and nonmetallic materials in such devices is a serious challenge for disposal and recycling. Some electronic parts contain more than 50 elements in one product or material system.^70,234,432,500^

Any recycling procedure that aims at recovering material from such equipment is confronted with the challenge that such practices always compromise the value of minute amounts of precious metals present in it more than that of the other alloy elements.

As a result, neither landfill disposal nor improper material treatment can be considered a sustainable process in the exploitation of precious metals through the recycling of urban mine resources. The ever-increasing mass-use of electrical and electronic waste, as well as spent catalysts, such as three-way catalytic converters, diesel oxidative catalysts, and petroleum catalysts, imposes a significant sustainability burden.

The current status in this field is based on extractive metallurgy of precious metals from such urban mine resources by using halide-, cyanide-, thiosulfate-, and thiourea-based lixiviants, purification, precipitation, adsorption, supercritical fluid extraction, biomediated approaches, solvent extraction, and chromatographic techniques.

#### 7.4.10 Sustainable Pyrometallurgy for Producing Nickel and Cobalt

**Figure 128:**
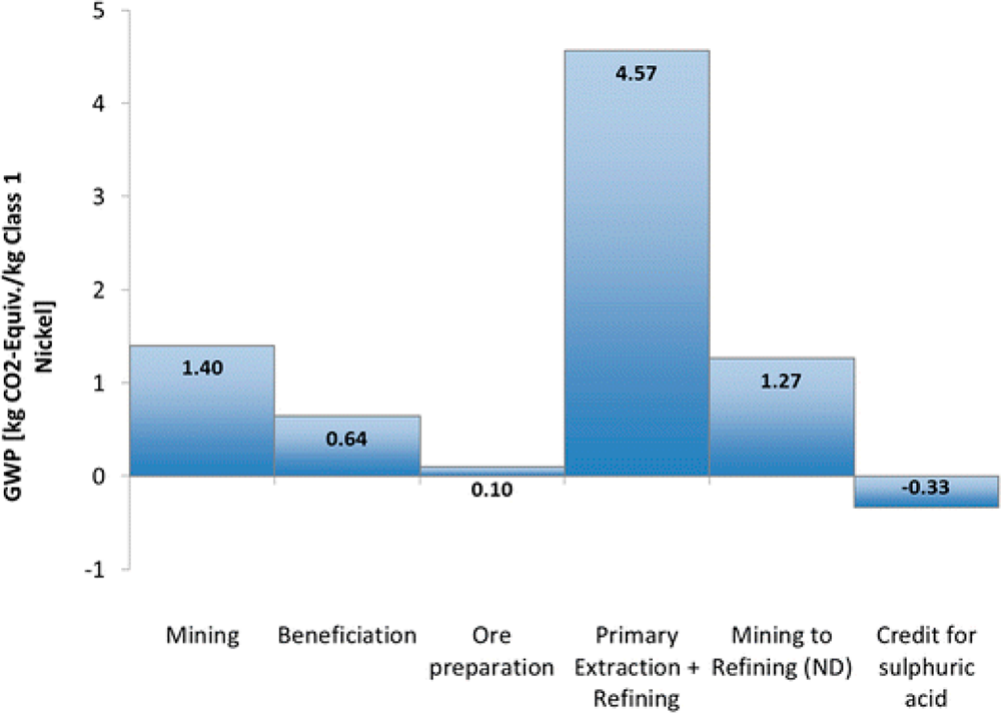
Greenhouse gas footprint associated with the individual nickel production steps.^138^ The main contribution comes from primary extraction and refining. GWP, global warming potential. The figure is reproduced with permission from ref (138). Copyright 2009, CISRO.

Nickel and cobalt are mostly produced from lateritic or from sulfidic ores, at a huge carbon footprint, exceeding that of most other metals, in part by a factor of 10, Figure 29 and Figure 128. The latter ore type usually also contains copper, so that metal recovery from sulfidic ores is a polymetallurgical extraction task, where copper, nickel, cobalt and several precious metals can be jointly won. The nickel content in laterite ores is usually low, rarely exceeding 1.0%, while the nickel content in sulfide ores can reach up to 2.5%.^138,205,417^

**Figure 129:**
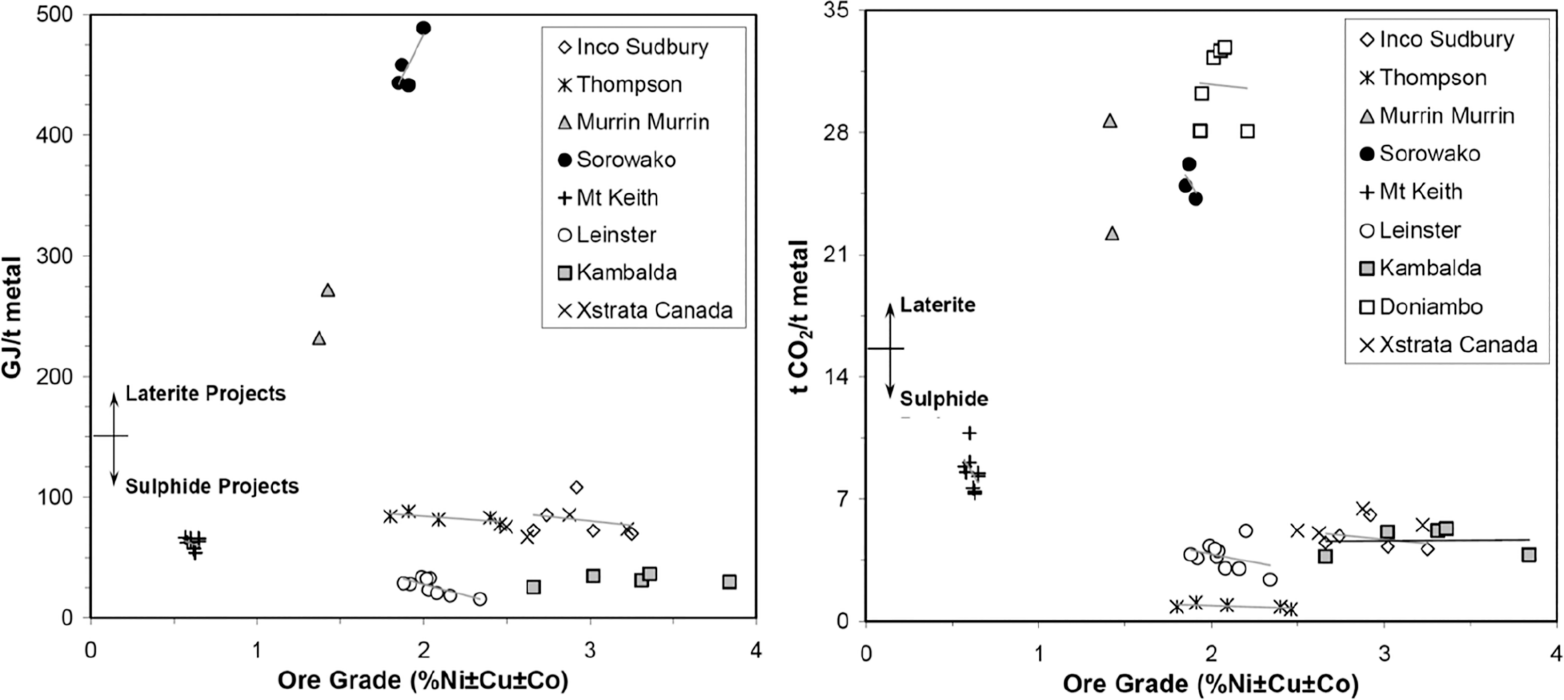
Energy and carbon footprint of Ni + Cu + Co extraction from different types of ores in terms of units of energy costs and units of carbon dioxide emissions with respect to ore grade.^138^ The figure is reproduced with permission from ref (138). Copyright 2009, Canadian Metallurgical Society, Sudbury, Ontario, Canada.

Recovery of nickel, cobalt, and copper from their ores is, for the most part, quite similar, Figure 129.^205^ The use of high-temperature refractories and the increased cooling required to accommodate the high operating temperatures in nickel and cobalt production are the major requirements. The specific processes used are determined by whether the ore is a sulfide or a laterite mineral. In the case of sulfides, the reaction of oxygen in the ore with iron and sulfur provides some of the heat required for smelting. Oxide ores, on the other hand, do not produce the same reaction heat, so that external energy must be used.

The extraction of nickel and cobalt from sulfides begins with crushing and grinding the ores in order to separate nickel sulfides from gangue via selective flotation. For this purpose, the ore is mixed with special reagents and stirred by mechanical and pneumatic devices that produce air bubbles during this process. As they rise through the mixture, the sulfide particles adhere to their surfaces and are collected as a nickel concentrate of 6–12%.

Magnetic separators can be used instead or in addition to flotation, because some of the nickel-containing sulfides are ferromagnetic. In cases where the copper content of the ore is nearly equal to that of nickel, the concentrate is subjected to a second selective flotation in which the copper is floated to produce a low-nickel copper concentrate and a separate nickel concentrate, each of which is processed in its own smelting line.

As with copper, nickel concentrates can be leached with sulfuric acid or ammonia, or dried and melted in flash or bath processes. Nickel requires higher smelting temperatures of around 1350 °C to produce matte, an artificial nickel-iron sulfide containing 25–45% nickel. Iron in the matte is then converted to oxide, which is combined with a silica flux to form slag. The process is carried out using a rotating converter, similar to the ones used in copper production. The slag is removed, leaving a matte that is 70–75% nickel rich. Because directly converting nickel sulfide to metal would require an extremely high temperature above 1600 °C, the removal of sulfur at this stage of the converting process is controlled to produce the only 70–75% nickel containing matte, which has a lower melting point. On the other hand, the relatively high sulfur-to-nickel ratio in most nickel concentrates increases the smelter’s sulfur containment burden.

This nickel matte can be subsequently treated in a variety of ways. In the ammonia pressure leach process, nickel is recovered from solution using hydrogen reduction, and sulfur is recovered as ammonium sulfate.^138,141,205,501^ The matte could also be roasted to create high-quality nickel oxides. These are pressure leached, and the resulting solution is electro- and carbonyl refined. Nickel is electro-refined and deposited on pure nickel cathodes from sulfate or chloride solutions. This is done in electrolytic cells with diaphragm compartments to prevent impurities from passing from anode to cathode. Nickel and iron carbonyls (Ni(CO)_4_, Fe(CO)_5_) are then produced by passing carbon monoxide through the matte in carbonyl refining. Nickel carbonyl is a highly toxic and volatile vapor that is decomposed on pure nickel pellets. Copper, sulfur, and precious metals are separated from the residue and treated separately.

In comparison to nickel and cobalt extraction from sulfides (and, similarly, copper extraction), their extraction from oxidic laterite is more difficult.^138^ On the other hand, because the laterite ores are free of sulfur, laterite nickel and cobalt deposits do not cause a pollution problem as do the sulfide ores. However, they do require substantial energy input, and their mining can have a detrimental effect on the environment, e.g., in terms of substantial soil erosion.

**Figure 130:**
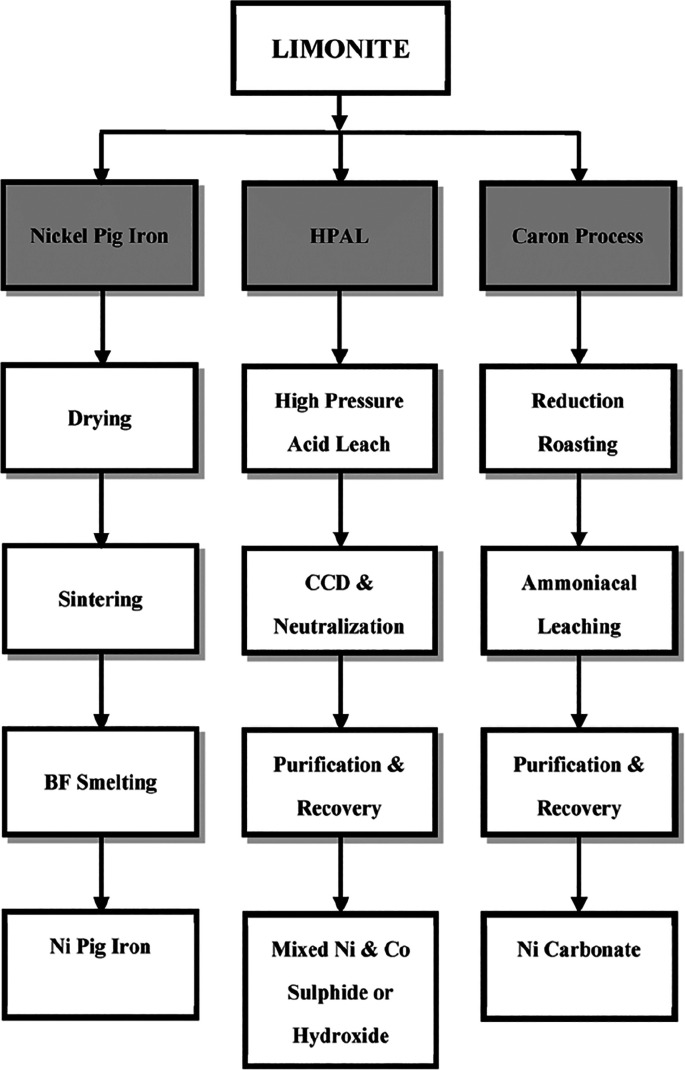
Different processing routes for the extraction of nickel and cobalt from limonite laterites.^204^ HPAL, high pressure acid leaching; BF smelting, blast furnace smelting; CCD, counter-current decantation. The figure is reproduced with permission from ref (502) under an open access Creative Commons CC BY license. Copyright 2017, MDPI.

Laterite extraction necessitates beneficiation prior to processing, and traditional flotation is ineffective for these mineral types. The majority of laterite ores contain iron-rich limonite and saprolite. Limonite is more amenable to hydrometallurgical processing (such as leaching and electrowinning), whereas saprolites are more amenable to pyrometallurgical processing (e.g., using rotary kiln furnaces). Laterite mineral ores contain large quantities of water, up to 35–40%, in the form of moisture and also in chemically bound form as hydroxides.^14,203^ Drying of moisture and removal of the chemically bound water are therefore major and costly operations. These are carried out in large rotary-kiln furnaces. Dryers up to several tens of meters in length and several meters in diameter are quite common today, while reduction kilns of a few meters in diameter and more than 100 m length are required to handle the large tonnages of ore and to provide the necessary retention time, Figure 130.

**Figure 131:**
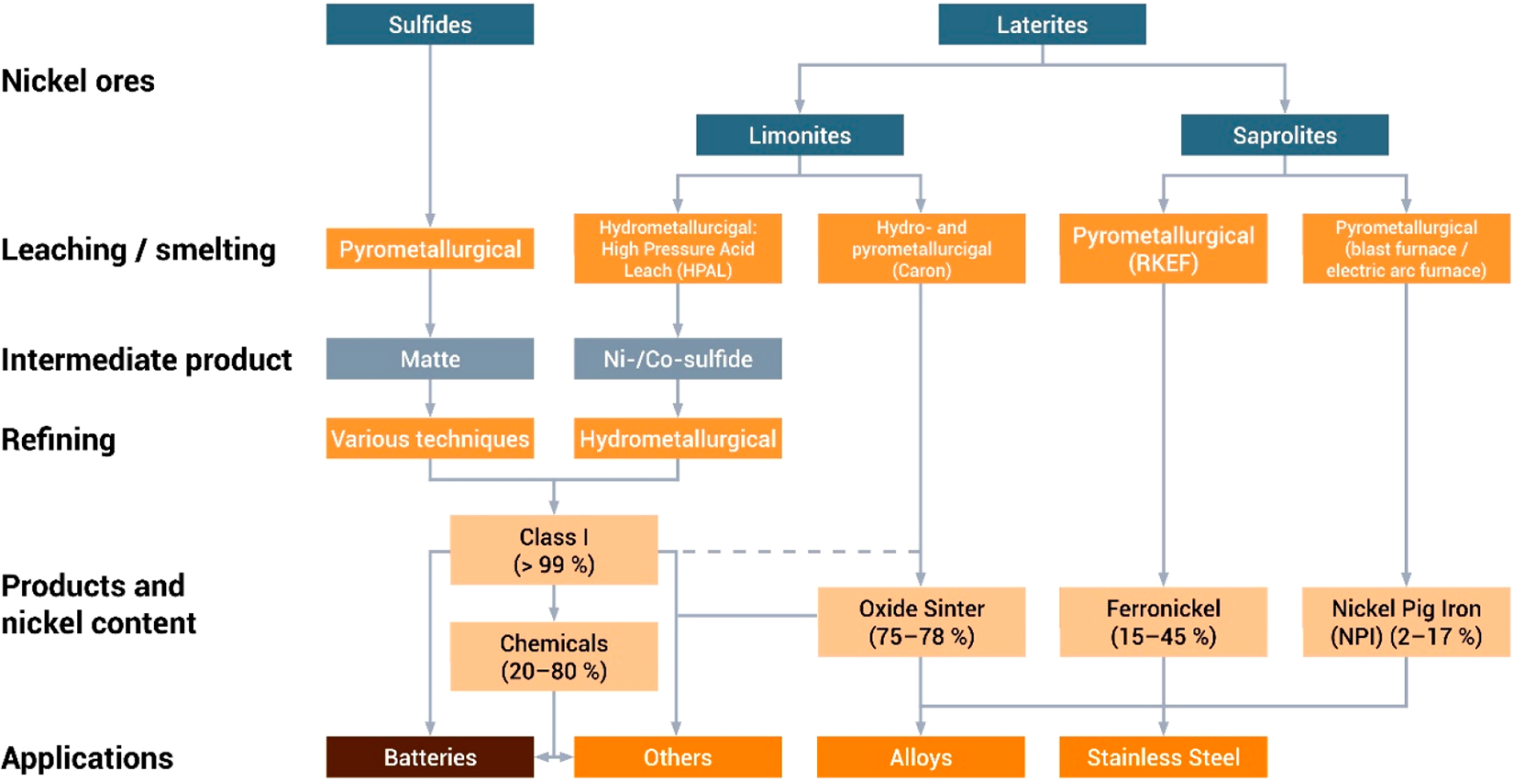
Overview of the most important nickel production pathways.

A typical leaching method is the high-pressure acid leaching where the laterite is leached with sulfuric acid at very high pressure values with up to 5.4 MPa and at temperatures in the range between 245–270 °C, usually in a titanium-clad autoclave.^204^ The metal-rich solutions are then treated in a hydrometallurgical solvent extraction plant after preceding solid–solution separation via counter current decantation, Figure 131. This processing chain of laterite ores needs substantially more water, energy and chemicals to produce nickel than from sulfides. An alternative process for laterite mineral processing uses the so-called “Caron” process, a combination of hydrometallurgy and pyrometallurgy. In this process, the ore is dried and reduced. It is then subjected to high-temperature ammonia leaching, where cobalt is an essential by-product (from all nickel laterite processes), especially from high-pressure acid leaching.

Next, it is necessary to reduce the oxide to nickel metal. Electric furnaces operating at 1300–1600 °C are commonly used as laterite nickel smelters. The high magnesia content in most laterite ores and the liquidus temperature of the furnace products necessitate these higher smelting temperatures, which in turn makes cooling within the refractory lining of the furnace necessary. In some plants, sufficient sulfur is added to produce a furnace matte that can be further processed like matte from a sulfide smelter. However, the majority of laterite smelters produce a crude ferronickel, which, after refining to remove impurities such as silicon, carbon, and phosphorus, is marketed as an alloying agent in the steel industry.

A challenge is that the market for nickel is traditionally divided into two parts: most of the growth in supply in recent years was due to so-called class II nickel, which is mainly used for the production of stainless steel. Class II is however usually not suitable for the production of the precursor nickel sulfate needed for batteries. Up to now, this has been obtained mainly from class I nickel. However, it is expected that the supply of class I nickel will grow by an average of only 0.8% per year until 2040. The additional nickel supply would therefore not relieve the tight supply situation for nickel sulfate for the time being. Some producers start to convert material extracted from class II nickel into nickel matte, which in turn can serve as base material for nickel sulfate. This process might become a game changer in this field, solving shortages of nickel sulfate, yet the feasibility and commercial viability of this process have yet to be demonstrated,^207,371^Figure 131.

An important final step for the production of high purity nickel is the so-called Mond process which serves to clean nickel-based metals via a chemical transport reaction. In this process, the transport medium is carbon monoxide and the compound transported via the gas phase is nickel tetracarbonyl, Ni(CO)_4_. The transport occurs from a cooler zone of the furnace at a temperature of approximately 80 °C to a hotter zone with about 200 °C. Nickel tetracarbonyl forms at lower temperatures and decomposes again into nickel and carbon monoxide at higher temperatures. The process cleans the metal as the reaction from nickel to nickel tetracarbonyl is exothermic. This means that the chemical equilibrium at high temperatures is on the side of the elemental nickel. Impurities are either not brought into the gas phase at the lower temperature or they no longer partition back into the nickel at the higher temperature.

Similar to nickel, cobalt-containing iron sulfides are roasted to produce iron oxide, which is then slagged with silicon dioxide to form iron silicate, producing the so-called raw stone. This intermediate product contains nickel, copper and other elements in sulfidic or arsenic form in addition to cobalt. The sulfur is then further reduced by roasting with sodium carbonate and sodium nitrate. The sulfates and arsenates are leached out with water, while the metal oxides are retained and treated with sulfuric or hydrochloric acid where cobalt, nickel and iron go into solution. Cobalt can be selectively precipitated as cobalt hydroxide by using chlorinated lime. Through a heat treatment it is converted into a cobalt oxide spinel and further reduced to cobalt with a reducing agent such as coke or aluminum.

Most of the cobalt is currently won as a by-product of nickel and copper reduction. Froth flotation can be used to separate cobalt from copper and nickel, in which surfactants bind to various ore components, resulting in cobalt ore enrichment. Roasting converts the ores into cobalt sulfate and oxidizes copper and iron. By washing out with water, the sulfate is then extracted.

A new emerging mineral source entering the market from deep sea mining are manganese nodules, Figure 91 and Figure 92. They contain 1–2 wt % nickel and cobalt and might open new research opportunities for sustainable synthesis. Owing to the specific composition of these nodules, which is profoundly different from conventional land-based ores, new extraction pathways must be found. An important aspect here is that not only the expensive nickel and cobalt are extracted from such manganese nodules, and many of the other elements such as manganese, aluminum, iron, silicon and copper are deposited as waste. Rather, in this context, when such new raw materials enter the market, sustainable processes must be developed from the onset that function in a CO_2_-neutral way on the one hand and extract all the ingredients of these minerals and put them to use on the other hand.

**Figure 132:**
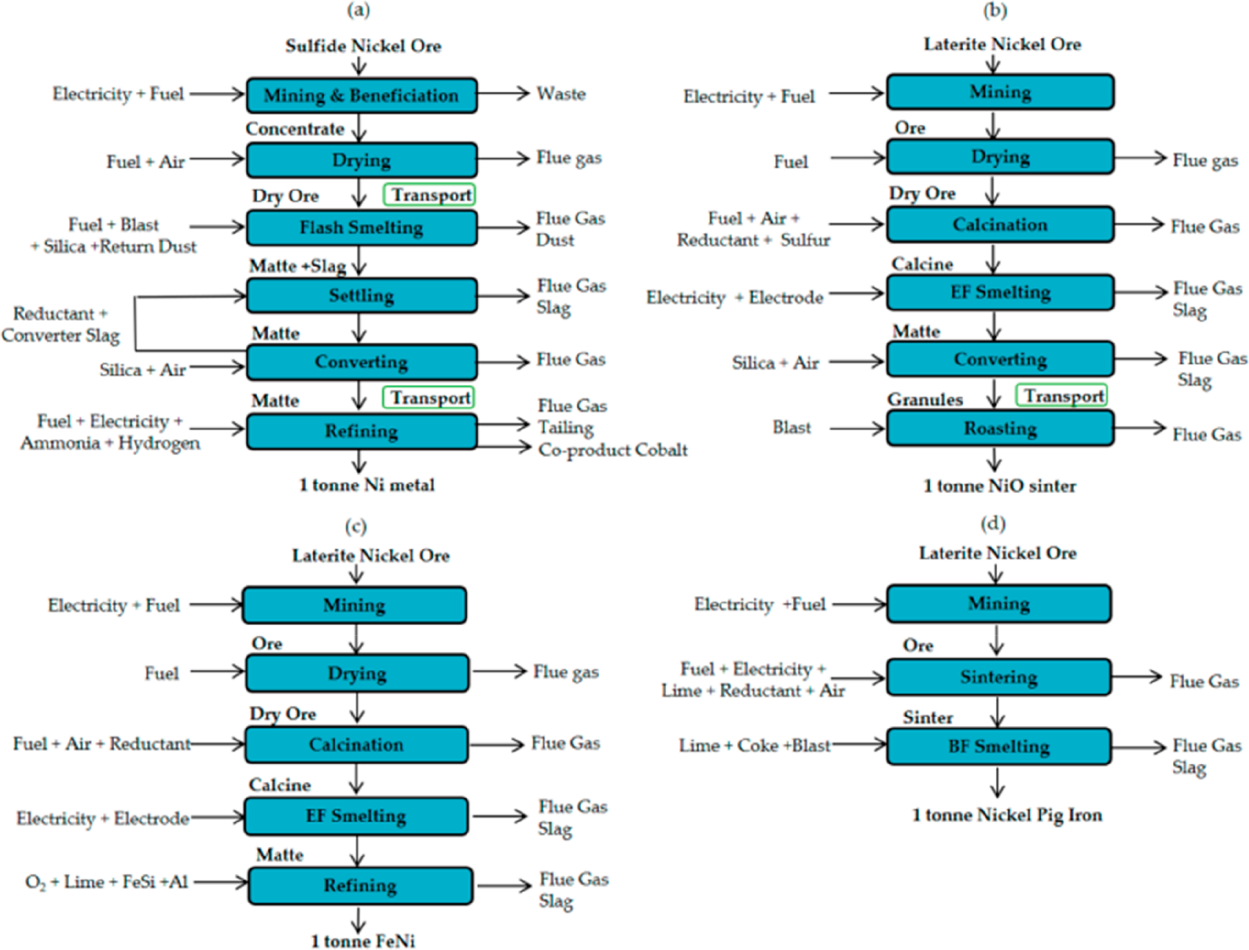
Four different types of nickel production pathways (for the four main different smelting nickel product types), including several options for using more sustainable reductants.^206^ The figure is reproduced with permission from ref (206) under an open access Creative Commons CC BY license. Copyright 2020, MDPI.

In summary, Table 37 lists some interesting topics for a more sustainable nickel and cobalt production via pyrometallurgy, Figure 132.

**Table 37:** Opportunities for Basic Metallurgical Research Related to Sustainable Nickel and Cobalt Production, with Potentially High Leverage on Improved Sustainability

More efficient and CO_2_ reduced primary production of nickel from laterite ores, as this process is much more energy intensive than making nickel from sulfide ores
Use of organic biowaste as fuel and reductant
Slag waste heat recovery in ferronickel smelting
Bath smelting technology for ferronickel production (instead of rotary kiln/electric furnace process)
Plasma reduction, also by using non-fossil reductants
Reduction of nickel and cobalt ores by methane or by hydrogen
Metal extraction from manganese nodules, including CO_2_-free extraction of all metals from the nodules (zero-waste multi-metal extraction)

#### 7.4.11 Sustainability Aspects of Pyrometallurgy for Titanium Production

**Figure 133:**
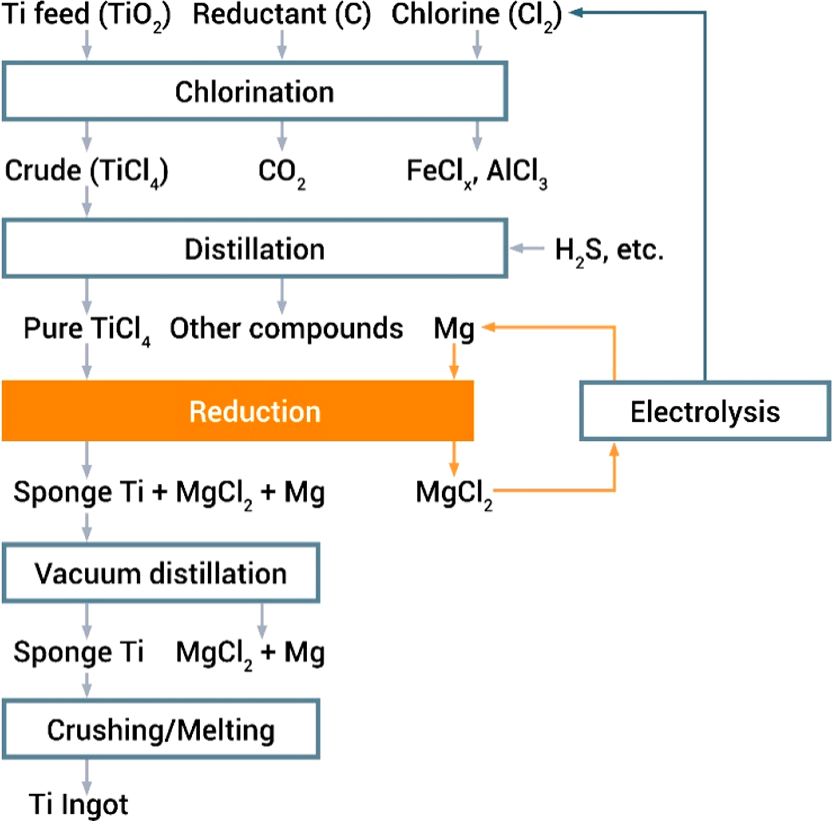
Overview of conventional titanium sponge production pathways through the Kroll process. The Hunter process is similar but uses sodium instead of magnesium as reactant.

Today the primary synthesis of titanium is conducted mostly using the Kroll process, mainly for cost and scalability reasons, Figure 133. Ilmenite (FeTiO_3_) serves mostly as feedstock mineral for this process, although the less abundant rutile mineral (TiO_2_) is actually the more preferred ore, because of its higher metal content. Rutile is produced from ilmenite by reducing it with (usually fossil) carbon carriers in an electric arc furnace. The liquid iron portion sinks to the bottom of the furnace where it can be tapped off. Instead of going through this lengthy separation step, in the industrial practice often natural or synthetic rutile is used. This can be obtained for instance by HCl leaching of ilmenite or from oxidized titanium.

The raw oxides cannot be reduced directly with coal (as for instance iron oxides), as this results in the formation of titanium carbide, which is very difficult to dissolve, or titanium nitride, in the presence of air. Therefore, the rutile is first exposed to chlorine and coke at temperatures around 750–1000 °C to form titanium(IV)-tetrachloride (TiCl_4_), which is the starting material for the actual Kroll process, in which the titanium is produced, using the chemically less-noble magnesium as a reductant. In the Hunter reduction process, an alternative processing method, sodium is used instead of magnesium as a reductant. Titanium tetrachloride, which is also the raw material used for white pigment production, is after purification by fractional distillation reduced to metallic titanium with magnesium at temperatures around 850–1150 °C under inert gas, to avoid hydrolyzation of the TiCl_4_. The so produced redox product magnesium chloride (MgCl_2_) is tapped off discontinuously, and the reduced titanium metal gets gradually accumulated to about 55–65% titanium content. Residues of encapsulated magnesium chloride and unreacted magnesium are either dissolved from the titanium sponge with hydrochloric acid or removed by vacuum distillation if higher purity is required. The typical chemical impurities that accumulate from the ores, such as iron, vanadium or silicon, are also separated from the TiCl_4_ by fractional distillation. The resulting product from the Kroll process is a hard and porous sponge. This material is next formed into electrodes for downstream processing into ingot form. It is remelted several times in a vacuum arc furnace to produce sufficiently clean and homogeneous titanium ingots.

The high manufacturing costs associated with the Kroll process have so far prevented the widespread use of titanium alloys. Therefore, alternative synthesis pathways to the Kroll process are being explored, both for sustainability reasons but also for cost reasons. A more widespread use of titanium alloys would be an attractive option in terms of indirect sustainability. Titanium alloys allow massive weight reduction in part design, taking advantage of the material’s low mass density (4.5 g cm^–3^), high durability, and up to 2–3 times higher strength compared to current high-strength automotive aluminum grades used for instance in electrical vehicles. High-strength titanium alloys can compete with advanced high-strength steels, and also they do not require costly and sometimes environmentally questionable corrosion protection measures.

An (actually older) alternative to the Kroll method is the Hunter process. It is based on the same process steps as the Kroll method to produce TiCl_4_, but uses sodium as a reductant, Figure 133. The reduced titanium metal adheres in the Hunter process less strongly to the inner wall of the reduction container, and the level of contamination by iron and other elements originating from the container wall is lower than in the Kroll process. To remove the remaining NaCl and other tramp elements, titanium is leached with dilute HCl. Unlike the Kroll process, the titanium produced this way is a powder (also referred to as sponge fines), which can be used as a low-cost raw material in powder metallurgy. Because the vapor pressure of NaCl as used in the Hunter process is lower than that of the Kroll reductant MgCl_2_, the distillation process is difficult, to separate out the NaCl. As a result, NaCl is removed through leaching in an aqueous solution. Furthermore, recovering the by-product, NaCl, from the aqueous solution necessitates additional energy.

Another method is the Armstrong process,^216,503,504^ an approach for a potentially more sustainable and yet scalable titanium production. It consists of two steps, involving the reduction of titanium tetrachloride with magnesium to produce titanium and magnesium chloride, followed by the purification of titanium via a distillation step. At first, the TiCl_4_ reacts with magnesium (or sodium) in a reactor to produce titanium metal and magnesium (or sodium) chloride. The reaction is exothermic and thus releases heat, which assists in driving the reaction toward completion. In a second step, the titanium produced is then distilled to remove the inherited impurities. This process is energy-intensive and requires careful temperature control to prevent overheating and combustion. The Armstrong process is nowadays widely used for the production of high-purity titanium or pre-alloyed material and is considered to be a cost-effective method for producing titanium on a large scale.

A further variant for high-purity titanium production is the Van Arkel–de Boer process. This approach is based on a transport reaction, where a solid starting material is converted into a halide. This halide is in gaseous form at a low temperature and decomposes back into the starting materials at a high temperature. The titanium sponge is purified by transposing almost exclusively the main component, but the impurities hardly react at all. The high-purity titanium is deposited in crystalline form on a tungsten wire. The reaction takes place in a vacuum to prevent the oxidation of titanium.

Several further alternative extraction processes have been developed during the last few decades to produce titanium and its alloys in a more sustainable and cost-effective way.^212,216,504−506^ Recent work on titanium production from molten salts or even via ionic liquids by electrochemical reduction has shown promising results, as will be discussed below in the chapter about electrochemical reduction methods.

Most of the currently used processes for titanium production are—like the Kroll process—inherently quite expensive. The main reason is that they generally require a high energy for the underlying redox processes to work and this involves also the use of strong and expensive reductants. This is due to the high energy of the bond between the titanium and the oxygen, as presented in terms of the position of the free energy for the titanium oxide (just above that for aluminum oxide) in the Ellingham diagram in Figure 58. This leads to a high energy consumption; use of expensive raw materials and expensive reductants; multiple process steps, including reduction and purification; complex equipment and facilities; high sensitivity and affinity of titanium toward impurities; and rather low yield of titanium metal compared to the input raw materials.

Table 38 gives some research topics for a more sustainable and efficient titanium production.

**Table 38:** Opportunities for Basic Pyrometallurgical Research Related to a More Sustainable and Efficient Titanium Production

Improved purification during reduction
Alternative reduction methods using electrochemical reduction or plasma reduction
Hybrid methods combining (oxygen- and iron-contaminated) scrap melting and reduction
Smelting methods for upcycling contaminated titanium chips

#### 7.4.12 Sustainability Aspects of Pyrometallurgy for Copper

**Figure 134:**
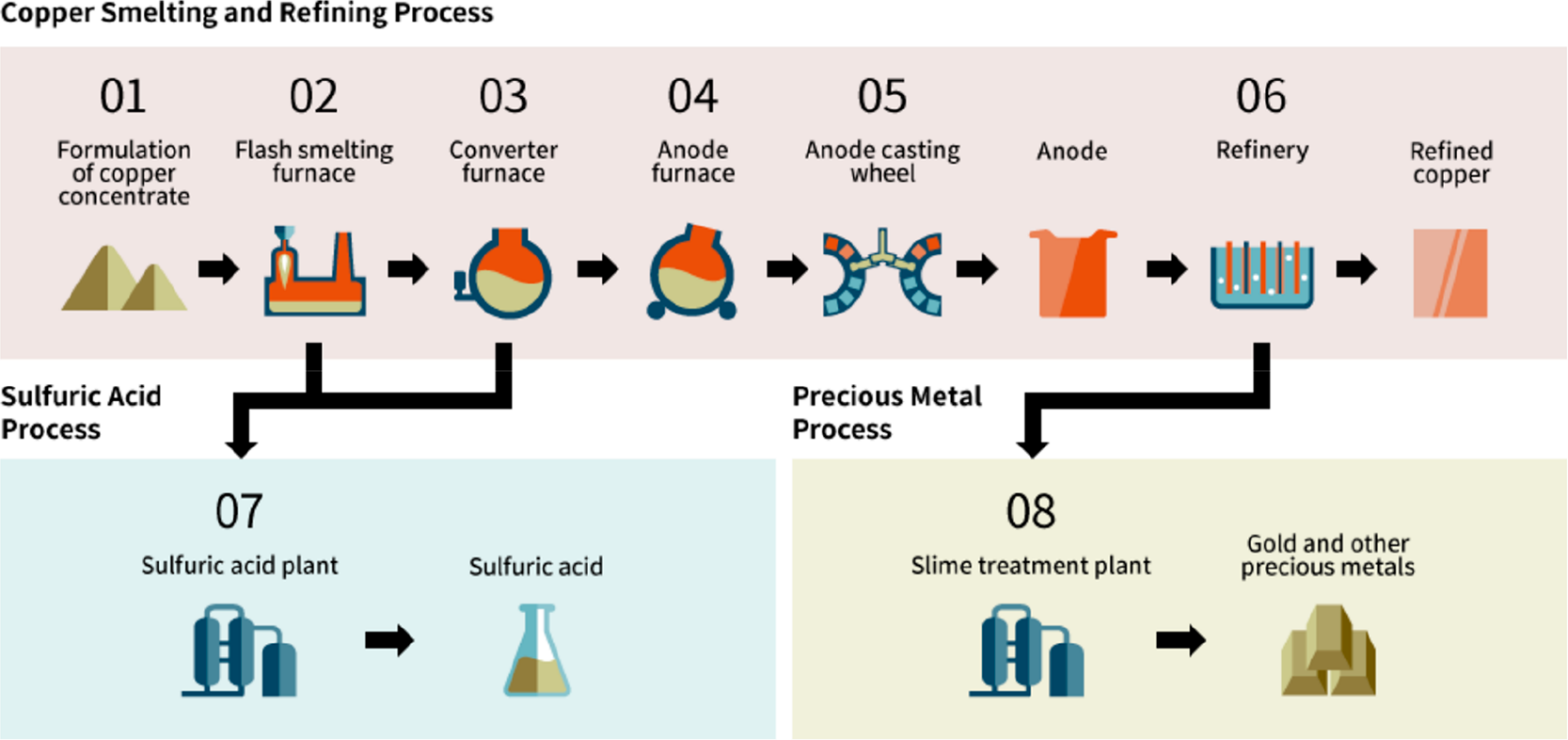
Schematics of copper production from minerals. The figure is reproduced with permission from www.ppcu.co.jp/eng. Copyright 2022, Pan Pacific Copper.

Most of the copper ores used today are copper sulfide-containing minerals, with a copper content usually not exceeding 2–3%. Three main metallurgical pathways are used to produce copper, namely, (a) primary production via extractive metallurgy using both pyrometallurgical and hydrometallurgical process methods; (b) bulk secondary production via direct smelting of new scrap and of bulk compositionally homogeneous end-of-life products; and (c) joint pyrometallurgical and electrometallurgical recycling from electrical and electronic post-consumer scrap, Figure 107 and Figure 134. Primary extraction has by far the highest carbon and energy footprint while secondary production from bulk scrap allows reduction of the total energy consumption by more than 80%.

**Figure 135:**
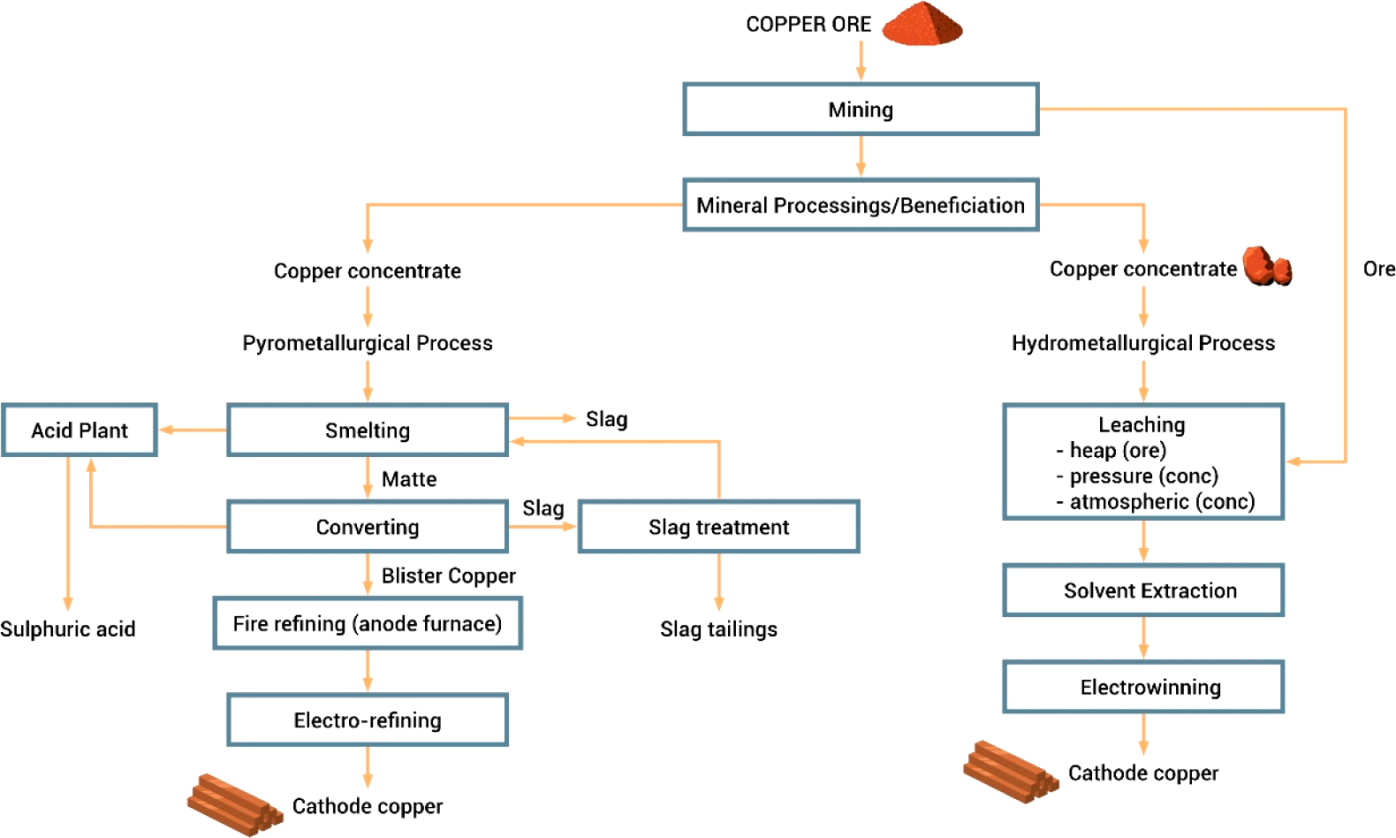
Detailed overview of specific primary copper production process steps.

Primary synthesis and the greenhouse gas emissions and the energy consumption associated with it depend on the type and quality of the ore and the required pyro- or hydrometallurgical processes, Figure 135. Concentrates produced from copper sulfide ores are usually treated by pyrometallurgical processes and account for approximately 80% of total primary production, which means that leverage for improved sustainability is particularly high in this field. Pyrometallurgical extraction from sulfides proceeds along the steps froth flotation, thickening, smelting and electrolysis.^200,356^

In froth flotation the crushed ore is processed into a fine sand-like compound. Liquid is then added to transform it into a slurry consisting of the fine copper ore mixed with the gangue mineral debris. Chemical reagents are next added to selectively bind the copper-containing minerals and protect it from water adhesion. Air is blown into that slurry. The bubbles that form during this process step carry the bound copper to the top where it can be skimmed off while the gangue particles sink to the bottom. Thickening is achieved by pouring the copper foam into a tank where the foam breaks and copper-bearing solids settle to the bottom. The thickened copper concentrate contains metals, impurities and up to 30 wt % copper. The concentrate is then dissolved in a mixed melt and poured into a slag settling furnace. Here, a siliceous aggregate converts the iron oxides into slag. Coke is used to remove sulfur. The iron silicate slag can be drained off as it floats on top of the copper matte. Copper matte, a mixture of copper, sulfur, iron and up to 70% copper, is thus produced in this step. The copper matte is further processed into crude copper. This product, referred to as black copper, has about 98% content. It is put in molten form into a converter and air is injected. In the slag blowing stage, the retained iron sulfide is roasted, a step that transforms the sulfide to iron oxide, and this is bound by quartz to be rendered into slag. This by-product can be drained. In a subsequent step two-thirds of the remaining Cu_2_S are oxidized into Cu_2_O. The oxide reacts with the remaining sulfide to form crude copper. The raw copper, which is also referred to as cement copper, is cleaned by cathodic deposition in an electrolysis step into electrolytic copper with a content of 99.99%. The base metals of these admixtures remain dissolved in the electrolyte; the nobler metals (including silver and gold) form the electrolyte sludge (also referred to as anodic mud) and are extracted separately.

The energy consumption for copper extraction depends on the ore, as discussed above for the case of nickel and cobalt extraction, Figure 128–Figure 132.

**Figure 136:**
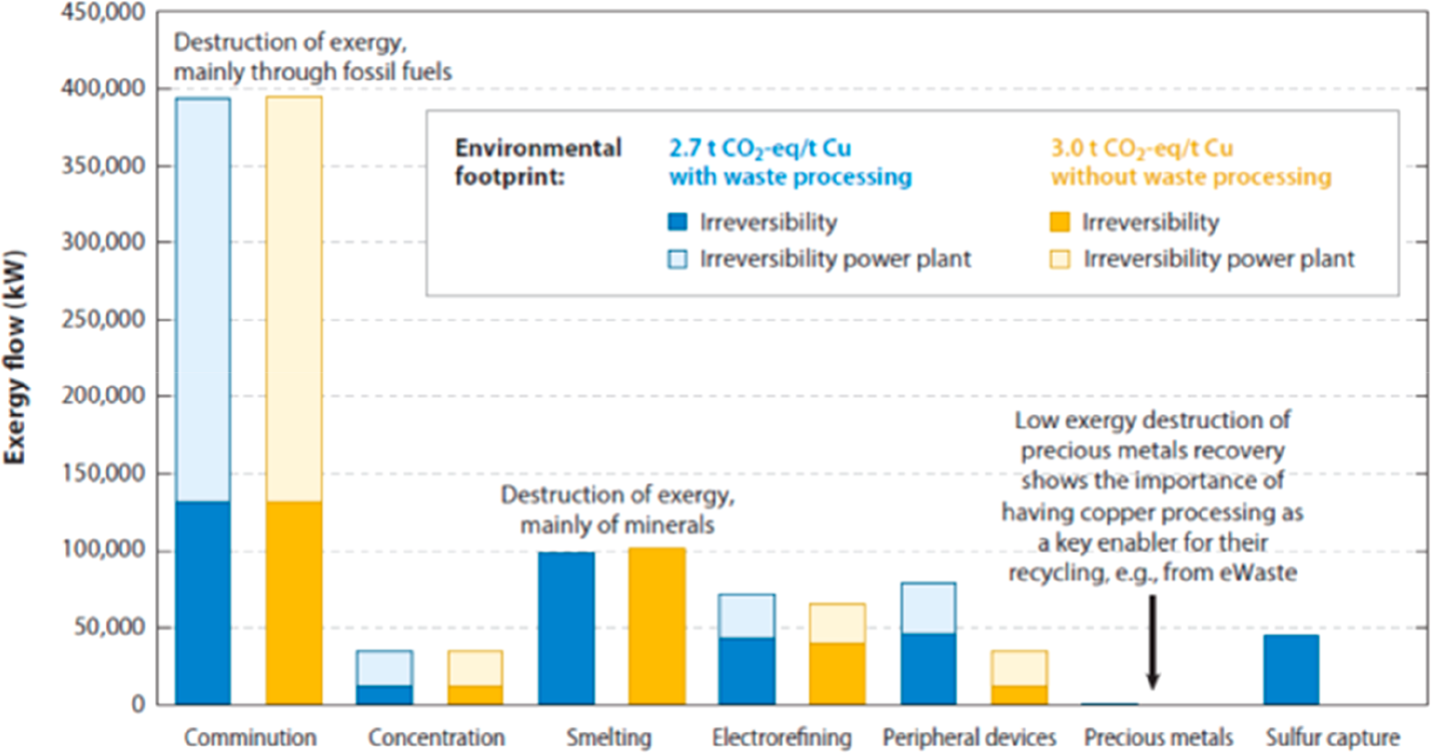
A model-based analysis of the energy intensity in copper production along the production chain.^21^ The figure is reproduced with permission from ref (21). Copyright 2019, Annual Reviews, Inc.

The grade of copper’s mineral feedstock has been declining over time. Since the copper content in mineral feedstock has decreased from between 1.5 and 4% at the turn of the century to an average of 0.62% in 2010, mining and extraction have become significantly more energy-intensive.^198^ The energy intensity of copper production has been estimated to amount to about 1/3^rd^ each for the steps mining; smelting plus refining; and processing including tailing waste treatment. These numbers are expected to change, toward a much higher energy- and cost-intensity of the mining portion, due to expected further falling ore grades and the high dispersion of mineral deposits,^439^Figure 136.

Efforts to reduce greenhouse gas emissions in this field should focus on copper processing steps associated with mining and processing. These can include energy-efficient crushing methods, use of more environmentally friendly melting technology, and the use of a higher percentage of scrap. Currently in the EU about 50% of the copper is produced from scrap and 50% from primary synthesis and production.^11,135,385^

Compared to the roughly 3.8 MWh per tonne copper produced, consumed by copper smelters in global average, the best available technology can produce copper with just 1.75 MWh per tonne copper, but the lowest energy intensity shown by international benchmarking studies is approximately 2.05 MWh per tonne copper.^71^ This implies that 46% of copper smelting energy could be saved using currently available technologies. From a life cycle perspective, the introduction of new technologies can also contribute to reducing greenhouse gas emissions and, possibly, other environmental impacts.

An interesting side aspect associated with the production of copper consists in the coextraction of other metals during production that have high market demand. Important candidates are silver, gold, palladium, platinum and selenium. Their recovery could contribute to additional environmental and economic benefits (production of coproducts can reach up to 20% of the value of primary copper production). Regarding technological improvements, the major trends in copper smelting, refining, and electrowinning involve hydrometallurgical methods and further improvements in pyrometallurgy.

New process variants are currently under development which can extract copper from low-grade oxide ores and even from re-mined copper production waste. An example is the modified one-stage flash smelting technology where the specific improvements come from a lower total energy intensity and better waste management in terms of using aqueous acid.

Again, also in these processes the energy demand for copper production via pyro- and hydrometallurgy depends on ore grade.^204^ Concentrates produced from copper sulfide ores are usually treated by pyrometallurgical processes, which account for approximately 80% of total primary production. Therefore, the results presented in this analysis show the environmental benefits resulting from the use of pyrometallurgical technologies, which are mostly flash furnace-based (a modified one-stage process). The baseline reference for improvement quantification is the production of copper from concentrate based on shaft furnace smelting technology.

The direct treatment of sulfides is another strategy for better sustainability. Chalcopyrite sulfide minerals (CuFeS_2_) constitute 70% of the world’s copper deposits. The mineral also contains several precious and strategically important metals. The oxygen-rich atmosphere governs the distribution of impurities and requires extensive upstream and downstream operations to manage toxic gases and by-products, making smelting the only commercially viable method for processing chalcopyrite. Removing oxygen and directly treating chalcopyrite in the native sulfide regime offers opportunities to make copper production more sustainable. Electrochemical experiments and thermodynamic analyses show that gaseous sulfur and electrochemical reduction in a molten sulfide electrolyte can be efficient levers for selectively extracting the components in chalcopyrite. Electricity and inert anodes have been used to manufacture copper and metallic by-products while separating metal production from fugitive gas emissions and oxidized by-products.

A few aspects and opportunities for sustainable copper metallurgy are summarized in Table 39.

**Table 39:** Opportunities for Basic Research Related to Sustainable Copper Production from Primary Resources via Pyrometallurgical Extraction Methods

Avoidance of the formation of acid mine drainage in copper mining; methods for mining and processing with less water demand
Better separation equipment in copper smelters to minimize the formation of metal-rich dusts; better capturing of sulfur dioxide, which can be used to produce sulfuric acid
Phasing out of less-energy-efficient processing technologies
Multi-metal reduction methods: the coextraction of other metals during copper production that experience high market demand, such as silver, gold, palladium, platinum and selenium (recovery of co-products during copper extraction can reach up to 20% of the value of primary copper production)
Sustainable copper smelting, refining, and electrowinning using both improved hydrometallurgical methods and further improvements in pyrometallurgy
Copper production with less water consumption
Sustainable and less harmful copper production from arsenic-contaminated minerals
Direct chalcopyrite treatment

### 7.5 Hydrogen-Based Plasmametallurgy as a Sustainable Reduction Process

Hydrogen gas (also in concert with other reductant gases) can be converted through electrical excitation into a hydrogen-plasma or hydrogen-containing plasma. This is an ionized gas, in which some of the electrons are detached from the molecules. A hydrogen-based reducing agent in plasma state yields significantly higher reduction reactivity than conventional hydrogen gas, and the reaction becomes exothermic, at least for some of the radicals produced, Figure 68.

For this reason, synthesis methods are of great interest in which a plasma is used as a reducing agent for iron (or other metal) oxides, Figure 37. This can take place for instance in an electric arc furnace, in interaction with an oxide (hematite) melt, i.e. via reduction at a liquid–plasma interface, or with solid oxides, i.e. via reduction at a solid–plasma interface. The latter variant can be also referred to as plasma-enhanced hydrogen-based direct reduction.^237,256,507,508^

The high reductive effect of the plasma in both cases, solid-state and liquid-state reduction, might result not only from the multiple dissociated and exited states into which hydrogen is brought but also from the small size, the high temperature and the entry speed of the exited species.

As in conventional solid-state direct reduction, in the case of the plasma-enhanced solid-state reduction, the reactive plasma species must—after the first very fast surface reduction step—penetrate into the inner oxide material through a layer of already reduced iron. These semi-reduced compounds form a core–shell type structure so that the kinetic bottleneck step is the outbound diffusion of the oxygen through this metal shell layer. In the liquid–plasma interaction this limitation does not exist to the same extent because the reduced material has about a factor of 2 higher mass density than the liquid oxide so that the reduced metal sinks to the bottom of the vessel. This implies that the liquid surface region where the reduction reaction takes place is always replenished with fresh oxide melt.

The reduction of Fe_2_O_3_ by a cold microwave hydrogen-containing plasma takes place already at low temperatures in the solid state, as has been shown by Sabat et al.^253,509^ Rajput et al.^144^ conducted solid-state cold plasma-assisted reduction experiments of hematite at different pressure conditions, both (a) with a plasma-free H_2_ atmosphere as a reference scenario and (b) under hydrogen plasma conditions. In the hydrogen plasma case, the apparent activation energy was 20 kJ mol^–1^, compared to 45 kJ mol^–1^ for the reference reduction case by using a conventional H_2_ (non-plasma) atmosphere. The authors assigned this drop in the apparent activation barrier essentially to the single-exited H_2_* species and other excited species in the plasma, which they estimated to consist of about 2% H, 8% H_2_*, and 90% H_2_.

**Figure 137:**
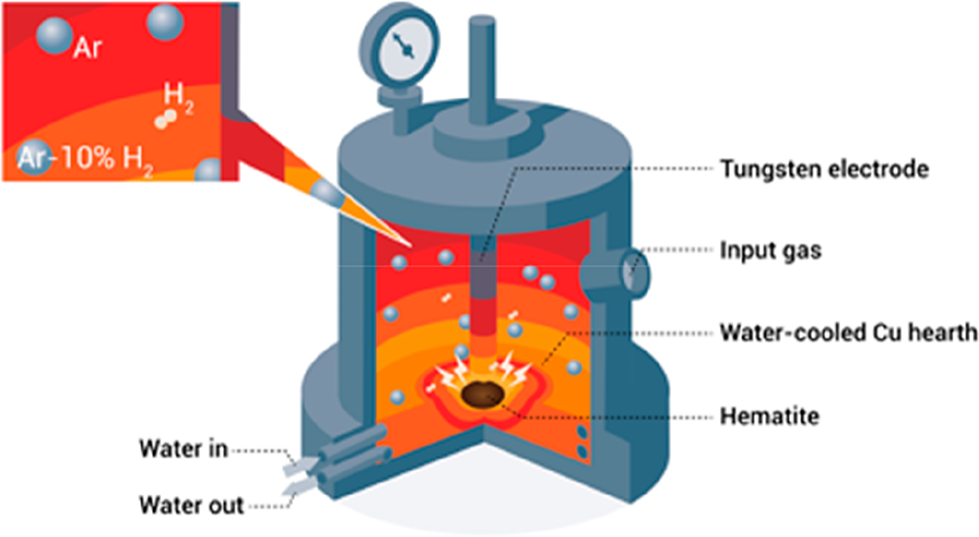
Experimental setup for hydrogen-plasma-based smelting reduction of iron oxides by Souza et al.^143^ with a process using a 10% H_2_–90% Ar gas mixture to produce the plasma.

**Figure 138:**
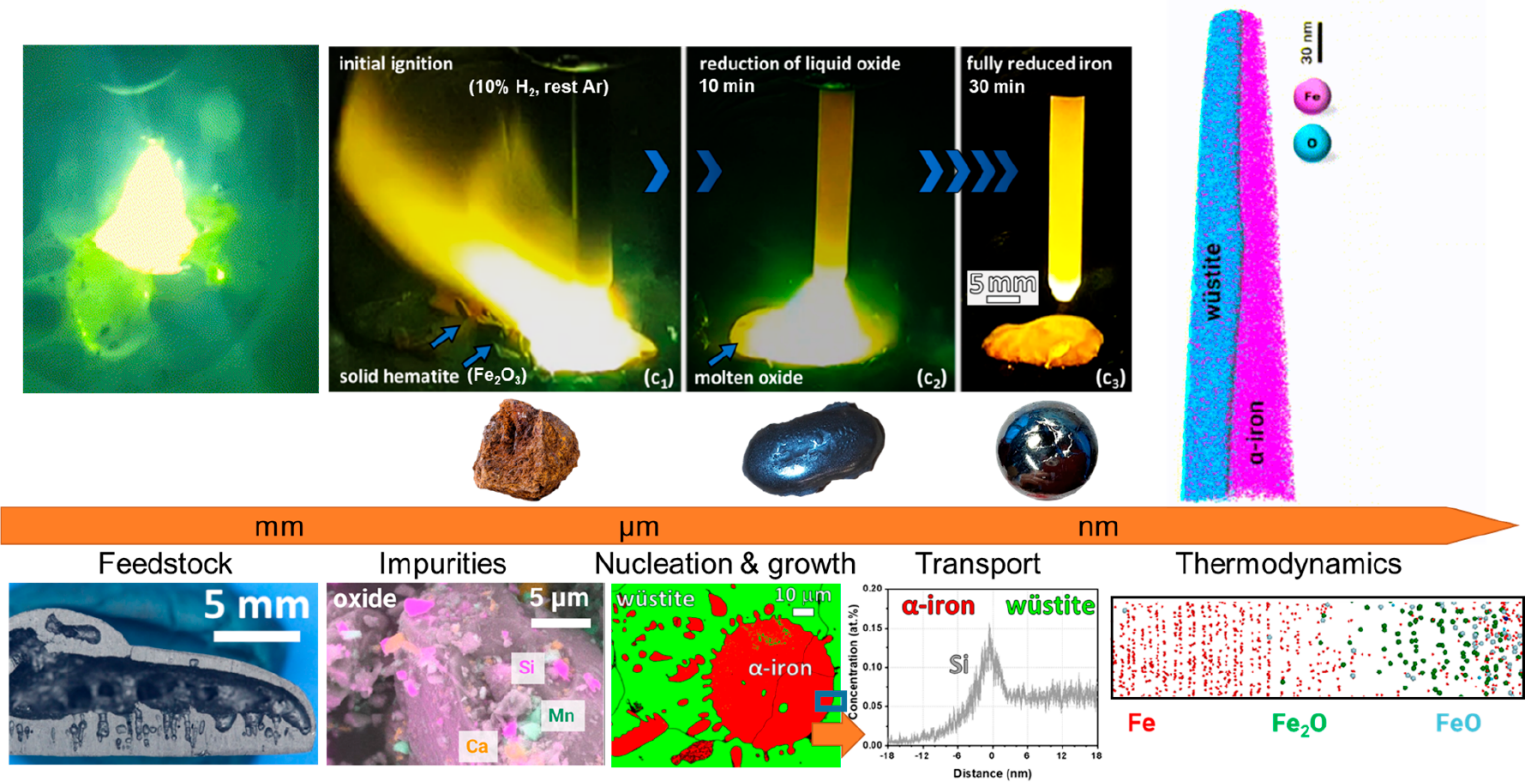
Very fast liquid-state hydrogen-plasma-based synthesis of iron from its hematite oxide. The images document the progress of several intermediate quenched-in partially reduced states from a liquid-state plasma reduction from a 10% H_2_–90% Ar gas mixture that was turned into a plasma.^143^ The oxide feedstock consisted of conventional commercial hematite pellets. The figure is reproduced with permission from ref (143). Copyright 2021, Elsevier.

Liquid-state plasma experiments with very high reduction rate were conducted with a 10% H_2_–90%Ar gas mixture by Souza et al.^143^ using an electric arc furnace, Figure 137 and Figure 138.

**Figure 139:**
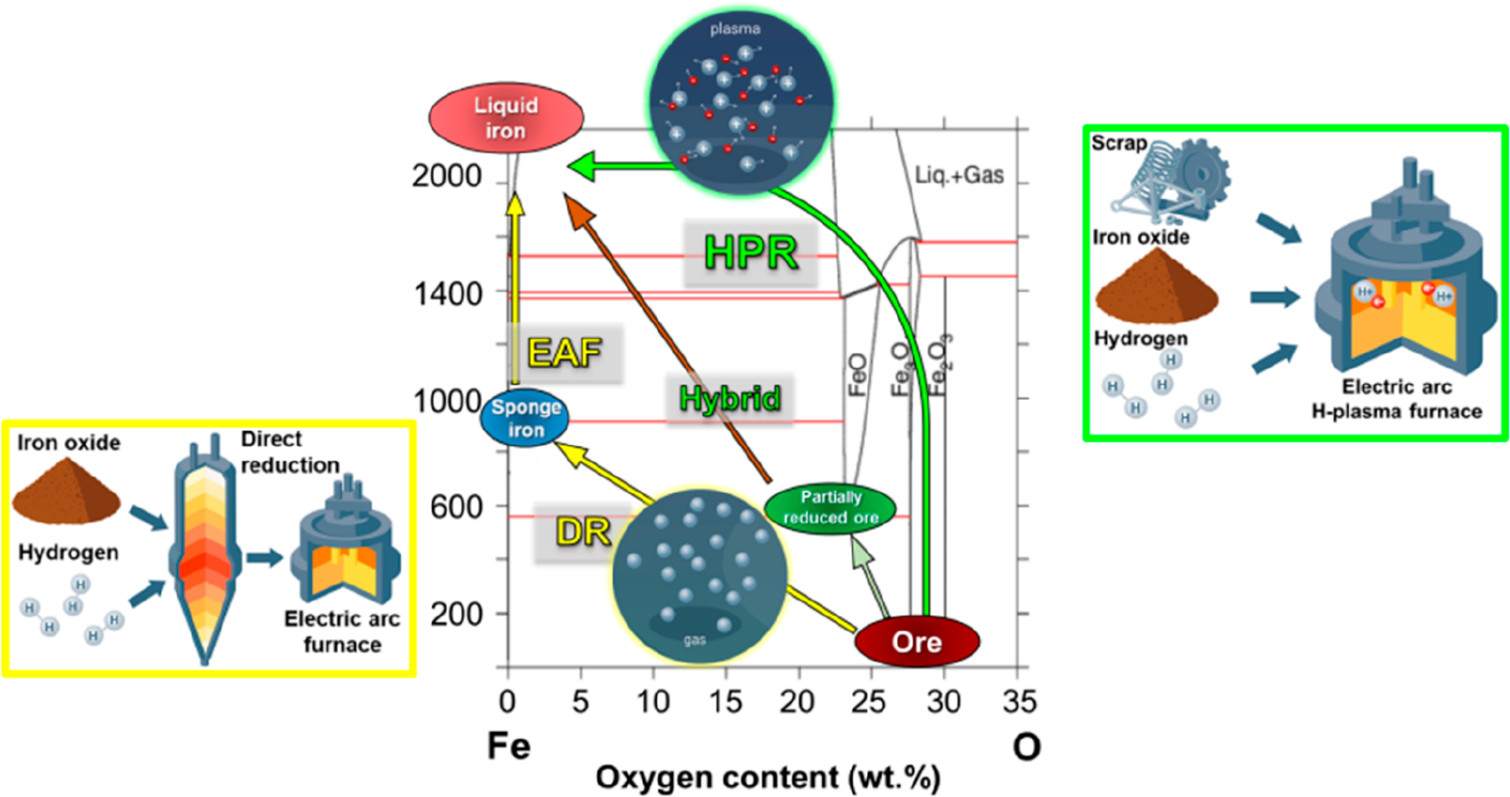
A novel hybrid process for sustainable iron making with hydrogen as a reductant. It consists in a combination of partial hydrogen-based direct reduction (in the solid state of the oxide) and a subsequent hydrogen-plasma-based smelting reduction (i.e., in the liquid state) of such a semireduced metal/metal oxide compound material.^123^ The reasoning behind such a sequential combination of two different reduction processes (solid state direct reduction and liquid state plasma reduction) is that hydrogen from sustainable production will not be available in sufficient quantities to meet the demands of the entire metallurgical market in the coming decades. For this reason, the highest possible stoichiometric efficiency must be ensured in all processes that use hydrogen as a reducing agent, and this can be achieved through this very combination. The figure is reproduced with permission from ref (123). Copyright 2022, Elsevier.

Using a microwave setup, Sabat et al.^253^ also reported the production of Cu from CuO and of Co from a Co_3_O_4_ oxide. Also, they studied the direct plasma-assisted synthesis of alloys like FeCo and CuNi^338^ through the reduction of corresponding metal oxide mixtures, using an oxide particle size up to 15 mm, exposed to a microwave power in the range of 600–1500 W for variable H_2_ flow rates.

The advantage of using plasma-based reduction methods is that for the case of liquid-plasma reduction, existing industry-scale conventional electric arc furnace technology can in principle be adopted and scaled to produce such a reductant-containing plasma atmosphere. Today commercial electric arc furnaces usually operate without using a reducing atmosphere but injection systems for co-fuelling with methane and hydrogen are already commercially available.

Like the reduction of metal oxides via molten salt electrolysis, the electric arc furnace can make direct use of sustainable electrical power, which yields an altogether very high efficiency compared to classical pyrometallurgical methods. This is an important aspect because the iron sponge that is produced by conventional direct reduction must be subsequently anyway charged into an electric arc or induction furnace for melting it into liquid iron. In electric arc furnaces are equipped with a reducing atmosphere, both steps, i.e. the reduction and the melting, can be done in a single furnace operation. Different from conventional solid-state direct reduction, hydrogen-plasma-based reduction in the liquefied oxide state yields full metallization and some of the oxygen is removed from the melt due to vapor pressure. Recently obtained data also seem to indicate that in direct reduction, where solid oxides are reduced, the use of a hydrogen plasma seems to allow substantially higher reduction rates and lower reaction temperatures, likely due to the presence of highly reactive exited hydrogen-radicals.

Several basic mechanisms need to be studied to mature plasma-based reduction and arrive at an optimal process, furnace, plasma and feedstock design. This includes such critical questions as graphite-free electrode materials, mixed plasma species and their reactivity, influence of gangue- and scrap-related contaminant elements on plasma state and reduction kinetics, the interaction between bath motion and reduction kinetics, slag formation and foaming response, the nucleation and growth phenomena of the iron inside of an oxide melt, partitioning in slag metallurgy, use of inexpensive and less-pure lump ores (hematite, magnetite), use of oxide fines, and the possible implementation and inheritance of hydrogen into the as-synthesized steel.

Recently Souza et al.^123^ have also combined the two reduction methods (direct reduction and plasma); i.e., they conducted a hybrid reduction which combined a partial solid-state direct reduction with a subsequent liquid plasma reduction of the partially reduced material, Figure 139. For optimizing the total energy and hydrogen consumption, the first part of the solid-state reduction (Fe_2_O_3_ → Fe_3_O_4_ → FeO) was conducted in a H_2_-based direct reduction furnace. The authors then charged the semireduced material (FeO plus some reduced Fe) into an electric arc furnace (which would have to be ideally operated with green energy) with a 10% H_2_-containing atmosphere (rest Ar) which then accomplishes the second half of the reduction together with melting, establishing a hybrid operation between the two furnaces. In all these hydrogen-based plasma reduction approaches it must be considered that the net energy balance for the reduction of hematite to iron with H_2_ is endothermic, i.e. it requires external energy to proceed,^179^ whereas it is exothermic with CO. When the hydrogen is brought into an ionized state^57^ (such as in an electric arc furnace), the reduction with hydrogen becomes exothermic, at least for some of the exited species in the plasma.^238^

Another very promising observation in the context of hydrogen-based plasma reduction of iron ores and mixtures of iron ores with scrap is also that it can obviously help to remove the undesired copper, Figure 101–Figure 103.

This is an important feature because copper, tin and lead can intrude as undesired scrap-related contaminant elements into such mixed melts, particularly when scraps come increasingly from electrical vehicles, Figure 101–Figure 103. A recent study indeed showed that several impurity elements can be removed from melts by hydrogen-plasma-based smelting reduction,^143^Figure 83.

The reduction with the help of hydrogen-containing plasmas was also investigated for other metals, whereby titanium production in particular would represent an interesting variant for a cheaper and sustainable production of this expensive metal in the future.^506^ More specifically, the feasibility of producing metallic titanium from the usual starting material titanium tetrachloride, which is also used in the Kroll and Hunter processes as starting material for the reduction, was studied using a thermal plasma under equilibrium and adiabatic expansion conditions. It was found that the crucial requirements for the production of metallic titanium powder from TiCl_4_ in a H_2_ originated thermal plasma were a rapid quenching of the plasma gas at high temperature and appropriate reactant concentrations. It was suggested that a fast quenching of the plasma gas and the production of titanium powder could be achieved by adiabatic expansion through a nozzle. Preliminary experimental data indicated that titanium powder of approximately 5 nm in size could be produced.^506^

In summary, hydrogen plasma reduction offers a sustainable approach to extractive metallurgy and it is thus a worthy topic to study the fundamental physical, microstructural, chemical, thermodynamic and kinetic foundations of the hydrogen-plasma-based reduction of iron oxides (lump, fine, pellets, hematite, magnetite, etc.) by H_2_ and its carriers (e.g., NH_3_, LOHC, etc.) including hybrid methods (i.e., hydrogen plasma final reduction after partial direct reduction). Basic research in this field will provide the scientific foundations needed for designing reactors and identify composition-tolerant iron-oxide feedstock and reductant mixtures for the highest metallic yield as well as hydrogen- and energy-efficient processes at fast reduction kinetics.

Table 40 lists some possible topics for basic research on sustainable plasma reduction of iron (and other metal) oxides.

**Table 40:** Opportunities for Basic Research on More Sustainable Plasma Reduction of Transition Metal Oxides

Plasma parameters (for solid- and liquid-state plasma reduction); plasma states and in operando process measurements
Plasma spectroscopy to better understand the presence of reactive species and nonthermal plasma effects as well as contamination
Plasma reduction mechanisms (for plasma-solid and plasma-liquid cases)
Use of mixed plasma feedstock using different reductants
Slag metallurgy and element partitioning and evaporation under plasma conditions
Nonequilibrium plasma conditions (solid and liquid state oxide reduction)
Gangue element removal by plasma reduction
Use of industry and deposited waste material as “urban feedstock” in plasma reduction
Use of mixed mineral and scrap-based feedstock in plasma furnaces
Materials science aspects (transport, phase transformation, etc.) in plasma-based reduction methods for solid and liquid interfaces; evaporation from reaction interfaces
Study of the exited states and the effects in reduction kinetics and metallization
Equilibrium and nonequilibrium plasma states and their translation into reaction rates
Plasma chemistry for complex mineral feedstock and multi-metal extraction
Mixed smelting of scrap and reduction of minerals
Sustainable coextraction of several elements from inferior and low-quality minerals

### 7.6 Hydrometallurgy

#### 7.6.1 Introduction to Hydrometallurgical Extraction Methods

The use of aqueous solutions for the recovery of metals from mineral mixes, solute or colloidal waste, concentrates, ores, and recycled materials is referred to as hydrometallurgy. Hydrometallurgical extraction also includes subtopics such as solvometallurgy or ionometallurgy, and it overlaps with extraction and refinement methods from pyrometallurgy, molten salt electrometallurgy and vapor metallurgy.^142^ Hydrometallurgical procedures have a few subdisciplines and specific process steps such as leaching, solution enrichment, solution purification, metal extraction, and compound extraction.

Ionometallurgy includes hydrometallurgical processes that use nonaqueous ionic solvents such as ionic liquids and deep eutectic solvents. These solvent types enable the development of closed-loop extraction workflows that can make use of leaching and electrowinning to efficiently recover metals. Ionometallurgy enables the processing of metals at moderate temperatures in a nonaqueous environment that controls metal speciation, accepts impurities, and simultaneously displays acceptable solubilities and electrical conductivities. As a result, typical processing routes are made simpler, and the size of a metal processing facility can be significantly reduced. Solvometallurgy is a related hydrometallurgical process variant for the extraction of metals from ores, scrap or waste material by using non- or low-aqueous solutions.

#### 7.6.2 Sustainability-Related Aspects of Hydrometallurgical Leaching and Extraction

Leaching is the key mechanism in most hydrometallurgical techniques. It involves the use of aqueous solutions to extract metal or compounds from metal-containing materials. Solution conditions vary in terms of the pH value, oxidation–reduction potential, presence of chelating agents and temperature, to optimize the rate, extent and selectivity of dissolution of the desired metal component into the aqueous phase. Through the use of chelating agents, one can selectively extract certain metals. Such chelating agents are typically amines of Schiff bases.

Leaching can be done in different reactor types, for instance in the form of in situ, heap, vat, tank and autoclave reactors. In situ leaching (i.e., leaching that takes place on the mineral deposit site) is a fracking variant. This involves drilling holes into the deposit and subjecting it to hydraulic or explosive pressure to penetrate further into the rock. A leachate is then poured onto such deposits to contact the ore. The remedy is then gathered and prepared. In both heap and vat leaching, minerals are exposed to a leachate that penetrates the material to extract the valuable metals. This is often done after crushing, classifying and agglomerating. This leach solution is enriched with dissolved metals and collected in sumps. Tank or agitation leaching, often in the form of staggered sequences of vessels, is a stirring method based on intense mixing dynamics between the refined and size-reduced mineral and the leach solution, where stirring enhances reaction and mass transfer kinetics. Autoclave leaching is related to the other methods described above, yet operated at higher temperatures, to enhance reaction kinetics and/or to allow for the use of gaseous reagents.

Hydrometallurgy is often seen as an environmentally more sustainable extraction method for metals compared to pyrometallurgical reduction, owing to the use of moderate or low temperatures. One must however consider the often massive water use and its contamination as well as the disposal of waste solids from these liquids. Pyrometallurgical methods on the other hand are huge emitters of greenhouse gases and gaseous sulfur; however, some of the slag waste products are rather inert or can serve as feedstock for other processes and materials, particularly in construction or for tertiary metal extraction.

#### 7.6.3 Hydrometallurgical Recycling of Battery Materials

**Figure 140:**
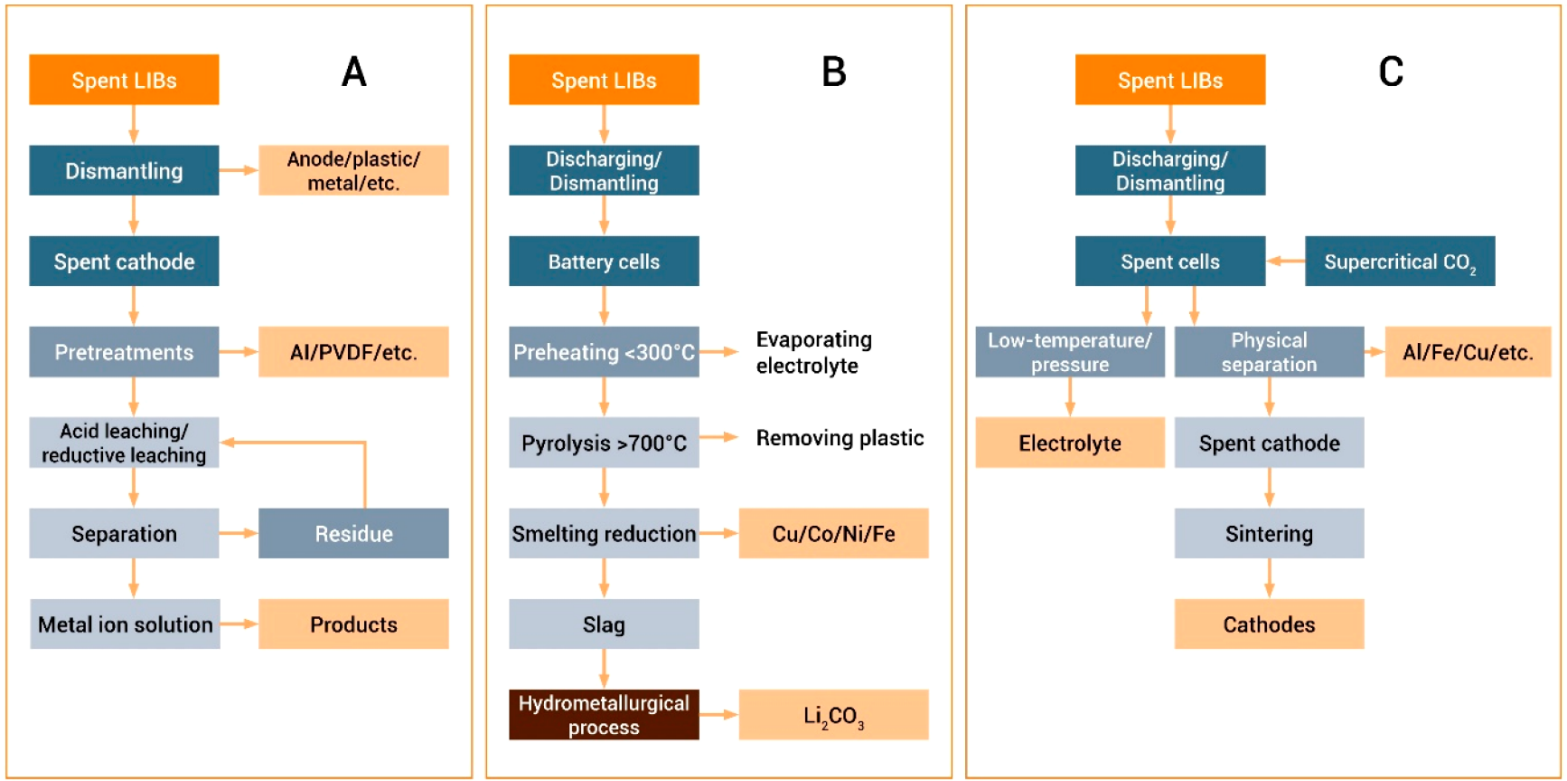
General overview of the different possible processing pathways for the recycling of used batteries. LIB, lithium ion battery.

Hydrometallurgical techniques, which involve leaching and reduction, can be employed for material and compound recovery after disassembling and crushing vehicle batteries, as an alternative to pyrometallurgical methods, such as pyrolysis-based techniques.^224,225^ In most cases of battery material recycling, several of these techniques are used in sequence, targeting the treatment and recovery of the respective subgroups of chemically similar metals, Figure 140.^137,223^

Leaching in hydrometallurgical battery metal recovery is done by acids or via (preceding) biological accumulation and leaching. For cobalt and lithium, recovery rates were reported to be above 99%, and for copper, they were over 98%. In acid leaching of electrode materials, inorganic acids are mostly used in hydrometallurgy, usually including hydrochloric acid,^510−512^ sulfuric acid,^231^ nitric acid, and phosphoric acid.^513^ The use of organic acids was also studied in that context.^136^

Hydrochloric acid leaching is difficult because the gas produced, Cl_2_, is highly corrosive and also very toxic. Strongly acidic HCl solutions should not be utilized to slow the dissolution of Mn from NMC electrode materials (NMC, sometimes also abbreviated NCM, refers here to lithium-nickel-manganese-cobalt-oxide cathode materials). In order to recover nearly 100% lithium and 95% manganese, acid hydrolysis of cathode materials with nitric acid was used as well as H_2_SO_4_ hydrolysis and hydrogen peroxide (H_2_O_2_) to reduce the Co^3+^ to Co^2+^. For inorganic acids, temperature, pH value, reaction time and additives have been shown to have high influence on the leaching performance. Depending on the specific parameter settings, up to 99% cobalt and 99% lithium could be dissolved.

In addition to strong inorganic acids, weak phosphoric acid has also proved to be a good solution for acidolysis. Pinna et al.^513^ revealed the leaching performance at a concentration of 0.7 M H_3_PO_4_ and 4% hydrogen peroxide, achieving a recovery rate of over 99% of lithium and cobalt at 40 °C after 1 h. The effect of ultrasonic waves and microwaves on leaching reactions also showed promising results. In recent years, also some mild organic acids have been widely studied in that context. During oxalic acid leaching, leaching and precipitation usually occur simultaneously, resulting in CoC_2_O_4_ precipitation and the separation directly from the Li^+^ solution without further treatment. In addition, because oxalic acid solutions are reductive, no additional reductants are required.

Table 41 shows some potential advantages and disadvantages of the different processing approaches with regard to sustainability in battery recycling.^136^

**Table 41:** Advantages and Disadvantages of the Different Processing Approaches for Battery Recycling with Regard to Sustainability

Process	Advantages	Disadvantages	Research tasks with regard to enhanced sustainability
Hydrometallurgy	High recovery rate	More wastewater	Wastewater treatment
	High product purity	Long process duration	Process optimization
	Low energy consumption		
	Less waste gas		
	High selectivity		
Pyrometallurgy	Simple operation	Li and Mn are not recovered	Reduction in the total energy consumption
	Less restricted charging size	High energy consumption	Reduction in the emission of waste gas and fumes
	High efficiency	High energy consumption	Combinations between pyro- and hydrometallurgy
		High waste gas output	
		High cost of waste gas	
Direct physical recycling	Low energy consumption	High operating equipment costs	Reduction in equipment costs
	More sustainable approach	Incomplete recovery of metals	Optimization of recovery performance

#### 7.6.4 Hydrometallurgical Recycling of Copper from Electronic Circuit Boards

Urban mining and recovery of metals from electronic circuit boards are essential in metallurgy, from the standpoint of both sustainability and metal scarcity.^500^ The latter point is often underestimated: while the average mining grades of traded copper-containing minerals such as chalcopyrite, chalcocite and malachite contain only very small amounts of about 0.6–0.8% copper, its content in waste circuit boards is usually around 10–20%. Also, it must be considered that copper ore reserves are reaching their limits and the rebound effect from the massive global electrification will accelerate this trend.

Traditionally, pyrometallurgy has been mostly used to recover metals from electronic circuit boards, and these methods often have good metal recovery rates, at least for some metals, but the high energy consumption and hard-to-control toxic dusts make these methods less sustainable. Hydrometallurgical methods are less energy intensive and have no gaseous emissions, so that they offer more sustainable pathways for metal recovery in this field than pyrometallurgy.

Besides the recovery of precious materials such as copper, silver, palladium and gold from printed circuit boards, a main goal of hydrometallurgical processing in this field lies in the control and safe treatment of the toxic and pollutant materials in them and associated with their recycling and deposition. Examples are heavy and potentially harmful metals such as such as arsenic, mercury, zinc, lead, gallium, selenium, cobalt, tin, etc. as well as toxic off-gases, such as chlorofluorocarbons, dioxins, furans, polybrominated organic pollutants, and polycyclic aromatic hydrocarbons.

Common hydrometallurgical methods for copper recovery from electronic circuits are acid leaching, ammonia and ammonium leaching, chloride leaching, and bio-leaching.

Important aspects related to metallurgical sustainability lie in the use of environmentally less harmful solutions as leaching agents, the use of staggered leaching processes prior to the final electrowinning of the copper to sufficiently enrich the solution in copper and deplete it in iron, zinc and nickel, development of sufficiently selective leaching solutions, leaching at reduced temperatures and pressure, and further downstream metal separation for instance of the gold and of toxic and heavy metals.

Table 42 lists some topics for basic research on sustainable hydrometallurgical recovery of copper from circuit boards.

**Table 42:** Opportunities for Basic Research on Sustainable Hydrometallurgical Recovery of Copper from Circuit Boards

Less harmful leaching agents
Low-temperature leaching
Recovery of heavy and toxic metals from dust and waste
Off-gas control

### 7.7 Electrometallurgy

#### 7.7.1 Introduction to Electrometallurgical Extraction Methods

Electrometallurgy can play an essential role in developing a more sustainable metallurgical sector because it can make use of renewable electrical energy to provide the driving forces for the required redox reactions.^514^

Electrometallurgy refers to a group of technologies for the extraction of metals from ionic substances in an electrolytic cell via driving ionic transport in these media and otherwise thermodynamically unfavorable chemical reactions at the electrode surfaces by using electrical energy. This includes molten salt solutions (“electrowinning”) or the purification of metals by electrochemical dissolution into/deposition out of such solutions (“electrorefining”).

The main elements of a metallurgical electrolysis setup are a container with an ionic substance, acting as the electrolyte (which can essentially assume any aggregate state); electrically conductive electrodes (usually solid or liquid); a voltage difference between the electrodes; and an external direct current source. The electrodes, an anode and a cathode, are usually immersed in the ionic substance that contains the metal(s) of interest. Each electrode attracts the ions of opposite charge.

This means that the ions carry the electrical current through the ionic substance, the electrolyte, where the positively charged cations travel to the electron-donating electrode, referred to as the cathode (negative electrode), producing metal extraction through cathodic deposition, and the negatively charged anions travel to the electron-accepting electrode, the anode (positive electrode).

The externally applied voltage difference supplies the potential energy that drives the reaction and that is necessary to discharge the ions when they arrive at the electrodes, where the electric current is then carried further by the electrons into the external circuit. The critical elementary process in any electrolysis is the interchange of atoms and ions by the removal or addition of electrons from the external circuit, i.e. the oxidation and respectively reduction steps.

Electrometallurgy plays a particularly important role for the extraction and purification of less-noble metals of groups 1 and 2 such as sodium and magnesium, along with aluminum, copper and zinc, as most elements that stand above aluminum in the electrochemical series have a high binding strength to oxygen and are thus difficult to reduce by other means.

During electrolysis, electrons are being added directly to the metal ions at the cathode, the negative electrode. The downside particularly in the aluminum preduction case is the high cost of the electricity. An advantage is that it can produce very pure metals and that sustainable electrical energy such as from wind or hydropower sources can be directly used at high Faraday efficiency. This makes many electrochemical extraction methods more attractive than the conventional reduction via fossil reductants and/or through transforming green electricity first into chemical bonds (such as through hydrogen) which are then used as reductant, yet at lower total efficiency.

The development of efficient electrochemical reduction technologies with reduced environmental impact could potentially also replace some of the conventional pyrometallurgical and hydrometallurgical metal extraction technologies. The reasons are that electrochemical reductions work without fossil reductants (yet, they often use graphite electrodes), have high efficiency (they do not have to go through chemical buffer reductants or fuels), and provide metal products of (usually) higher purity. The direct use of electricity is of course only pertinent in this context when using renewable electrical energy; i.e., the fossil footprint of the energy used must be billed in when conducting corresponding life cycle assessments.

Disadvantages of electrochemical reduction technologies are the usually high operating temperatures (which are, however, much smaller when using ionic liquids instead of molten salt mixtures); the highly corrosive electrolytes; fume production because of the high vapor pressure of most molten salts; dendritic cathodic deposition when the melting points of the extracted metals exceed the boiling points of the electrolyte; and limited production rates.

Two main directions prevail in electrometallurgy, namely, aqueous electrolysis, where an aqueous feed serves as electrolyte, and molten salts electrolysis, where the electrolyte is a molten salt (mixture) that contains the metallic ions of interest.

#### 7.7.2 Metal Extraction by Molten Salt Electrolysis

Molten salt electrolysis is widely used in electrometallurgy, such as electrolytic reduction of metallic compounds into metals, referred to as electrowinning, and purification of impure metal mixtures into purer metals, referred to as molten salt electrorefining.^515^

Molten salt electrolysis is specifically attractive in this field due to the high solubility of the salts for many metallic ions and good separability at high electrochemical decomposition potential windows, at often moderate overpotentials due to cathodic polarization in cathodic deposition, and their high ionic conductivities.

The high solubility for metals can also be problematic, for example regarding the corrosive attack and dissolution of the electrodes and electrolyte containers. Promising research topics in this field are thus the identification of inert electrodes, linings, refractories and container materials. Another problematic aspect lies in the usually quite high melting points of suited molten salt mixtures. This is not only expensive but also a serious problem when it comes to sustainability. More specific, a very attractive advantage of electrometallurgy lies in the use of sustainable electrical energy sources, and with this electrolytic cells can be seen essentially as batteries. It would be thus very desirable when they can operate at variable voltage and current density. This means that the molten salt should have a wide operation window before it freezes, to cope with variable sustainable power supply. Many molten salt mixtures are also quite hygroscopic so that the salt must be regularly refined.

Particularly chloride-based mixtures are usually rather hygroscopic, and some can decompose by hydrolysis. Fluoride salts have the advantage of being less reactive with moisture and, additionally, can dissolve oxides directly, avoiding fluorination, which requires reaction with a fluorine-containing compound. However, fluoride salts have usually higher melting points and cause more severe corrosion attack.

#### 7.7.3 Metal Extraction by Aqueous Electrolysis

Water participates directly and indirectly in many electrometallurgical reactions. Water molecule bonds can be broken as in the process of electrolysis to produce oxygen and hydrogen (2H_2_O = 2H_2_ + O_2_). However, in aqueous media this reaction takes place as two separate electrochemical reactions that each consist of half of the overall reaction.

Electrochemical reactions involving half of an overall reaction are known as half-cell reactions because they form half of a complete electrochemical cell. The half-cell reactions for water electrolysis are generally written as 2H_2_O = 4H^+^ + O_2_ + 4e^–^ and 4H^+^ + 4e^–^ = 2H_2_. If the solution is neutral or basic, the availability of hydrogen ions is insufficient to drive these reactions at reasonable rates. However, water molecules, which have a natural equilibrium relationship with hydrogen and hydroxide ions (H^+^ + OH^–^ = H_2_O), can be used in a way that does not directly require hydrogen ions in either the electron donating, anodic oxidation reaction (4OH^–^ = O_2_ + 2H_2_O + 4e^–^) or the corresponding electron accepting, cathodic reduction reaction (4e^–^ + 4H_2_O = 2H_2_ + 4OH^–^).

The polar and open structure of water molecules accommodates ion dissolution and mobility, making the combination of water and dissolved metal ions a very useful electrolyte. An electrolyte is a medium that conducts current through ion movement. Hydrogen ions and hydroxide ions are highly mobile and always available to conduct current through water in proportion to their concentration. Ions dissolved in water can thus conduct current through water without participating in electrochemical reactions, which makes such systems attractive for metal recovery. Metal extraction via aqueous electrolysis requires the minerals that carry the metal ions of interest to be soluble in water. It also requires as a precondition that the metal to be recovered does not react with the water. When such water-soluble metal-containing minerals are dissolved in water, the electrolysis process produces an aqueous ionic liquid by decomposing the compounds in solution into an ionic state. In this process the non-metallic anions then move to the anode where they lose their extra electrons and the metal cations that are less reactive than the hydrogen move to the cathode where they gain electrons and undergo cathodic deposition. Research topics of interest in this context are inert electrodes, the design of suited ionic liquids as electrolytes as well as the interface reactions and interface layers that form at the electrodes.

#### 7.7.4 Sustainable Aluminum Production via Molten Salt Electrolysis

The classical electrowinning of aluminum through the Bayer and Hall–Heroult processes described above creates severe environmental problems. On the one hand aluminum production is a very energy-intensive process: it consumes about 60 GJ per tonne of metal produced and generates huge amounts of red mud and gaseous emissions.^516−519^

The feedstock mineral for aluminum production is bauxite. It is purified into aluminum oxide via the Bayer process, producing an iron- and titanium-rich compound, referred to as red mud as waste. The red mud is also gaining momentum as a re-mined raw material that might serve as possible feedstock for future iron and rare earth production. Aluminum oxide has with over 2000 °C a very high melting point, which would make it very costly to win the metal by pyrometallurgical reduction methods. Therefore, the aluminum is extracted from this refined oxide by electrolysis via the Hall–Héroult process. However, aluminum oxide does not dissolve in aqueous solutions, but it does dissolve in molten cryolite. This is an aluminum-containing sodium hexafluoroaluminate salt (Na_3_[AIF_6_]). While the melting temperature of pure alumina exceeds 2,000 °C, that of the Al_2_O_3_-Na_3_[AIF_6_] compound is reduced to only 950–970 °C.

Graphite acts as negative cathode in this electrolysis operation. The positive anodes are immersed in the molten cryolite and are also made of graphite. Under an electric current the aluminum forms at the negative cathode from the aluminum oxide in the cryolite and sinks to the bottom of the cell, owing to its higher mass density. The oxygen from the aluminum oxide in the cryolite forms at the positive anodes. The oxygen reacts with the carbon of which the graphite consists to form carbon dioxide. The positive anode therefore burns up and needs to be replaced regularly. This is another reason why the extraction of the aluminum is so expensive, and it also explains the high CO_2_ emissions that result from the electrode burnup.

During the reaction at the negative cathode, where the aluminum is deposited, the aluminum ions are reduced from the molten alumina solution. This means they gain electrons at the positive anode where the oxygen reacts with carbon to form carbon dioxide.

Regarding approaches for improved sustainability of the electrochemical synthesis of aluminum, different starting points are conceivable. These are particularly longer-lasting and carbon-free electrodes, use of sustainable electrical energy for electrolysis, high cell efficiency and avoidance of cell freezing also for seasonally variable power availability, and further reduction of the cryolite melting point, Table 43.

**Table 43:** Approaches toward More Sustainable Electrolysis-Based Aluminium Production

Longer-lasting, inert and carbon-free cathodes
Replacement of anodes consisting of petroleum coke and tar pitch, consumed in the electrolysis in an anode reaction
Use of renewable electrical energy sources for electrolysis
Higher cell efficiency and avoidance of cell freezing also for seasonally variable power availability from renewable electrical energy sources
Electrolysis cell operation under variable electrical power supply
Low-temperature electrolytes
Reduction of the cryolite melting point
Use of red mud as a feedstock resource instead of dumping
Less electrode reactions
Replace techniques of leaching in an autoclave, calcination in a rotary kiln, and electrolysis in cells with Söderberg anodes with the altogether up to 20% lower-energy solutions of leaching in a tubular reactor, calcination in a fluidized bed reactor, and electrolysis in cells with prebaked anodes

The latter point has a particularly high leverage for enhanced sustainability because aluminum—owing to its high embodied energy—can serve as an ideal chemical energy buffer for balancing the huge fluctuations from wind and solar energy sources. In other words, in times of high renewable energy supply electrical aluminum winning should be intensified while in times of low renewable energy supply it should be reduced. This creates an interesting nexus between aluminum production and sustainable electrical energy production, through the adaptation of the electricity demand from aluminum winning to the actual current supply of sustainable energy. This approach gains momentum with the globally rapidly increasing contribution of renewable energy supply.

**Figure 141:**
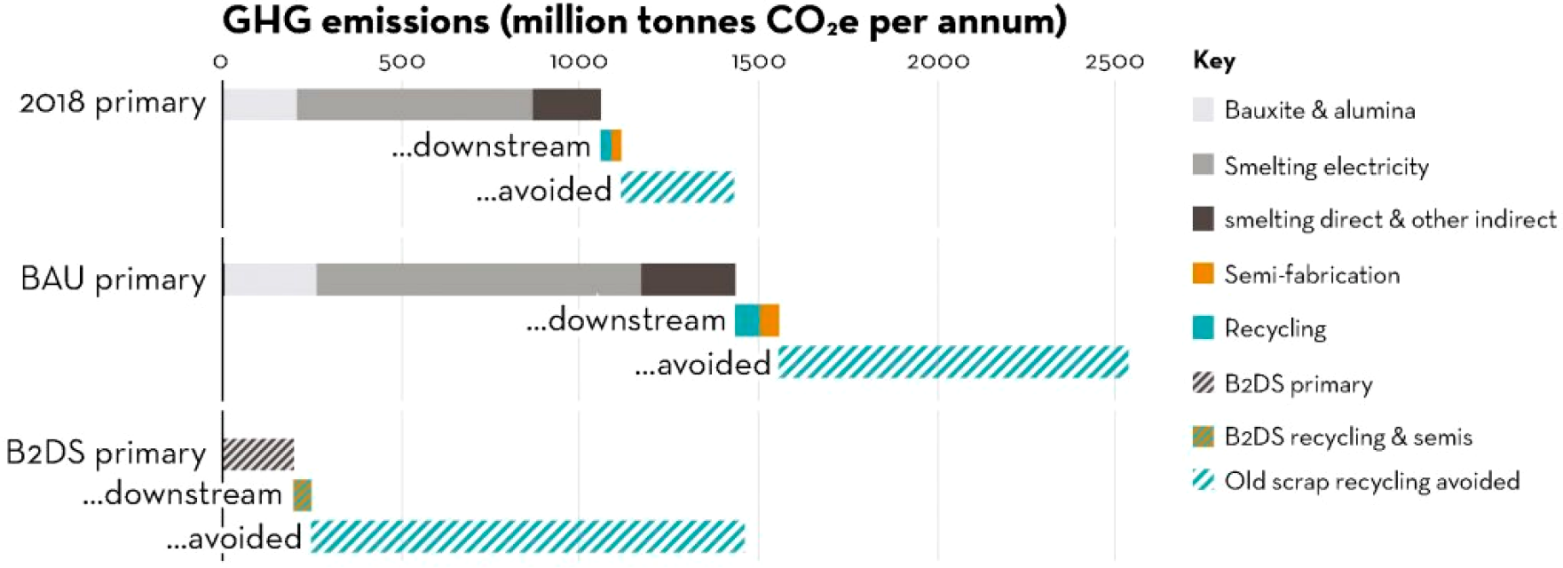
Greenhouse gas emissions per year, broken down by different sectors of the aluminum production chain.^187^ BAU primary, business as usual (i.e., conventional processing) scenario for the primary synthesis of aluminum; B2DS, beyond the 2 °C scenario. The figure is reproduced with permission from ref (187). Copyright 2021, International Aluminum Institute.

With about 60% of CO_2_ emissions, electricity consumption is one of the biggest emission drivers in aluminum production. A large part of this electricity consumption is generated in the electrolysis and smelting process of aluminum and aluminum scrap. Thus, most emissions could already be avoided with carbon-free electricity, Figure 141.

Another access point to improved sustainability are the electrode materials. The anodes consist of petroleum coke and tar pitch and are consumed in the electrolysis in an anode reaction, whereby the aluminum oxide is released in the molten bath reaction with the carbon of the anode to form carbon monoxide (CO) and carbon dioxide (CO_2_). An important research direction for enhancing efficiency and sustainability therefore is the introduction of iron-copper-nickel (or related) alloys as inert anodes, which are not consumed like the graphite anodes. Instead these transition metal electrodes release oxygen instead of CO_2_.^520^ Another approach is to produce aluminum using several vertical inert (nonconsumable) anodes and cathodes in a low-temperature electrolyte (800 °C).^245,248,521^

Research is also conducted regarding the use of new types of solvents referred to as ionic liquids in the primary aluminum production. Here, directly dissolving metallurgical alumina, trihydrated alumina and bauxite in novel ionic liquids is for instance investigated. The first results suggest that alumina can be dissolved in certain ionic liquids at much lower temperatures than required for the classical cryolite mixture, forming a solution that contains up to 10% alumina, a value that even exceeds the alumina content in the conventional Hall–Heroult cryolite mixture. It was found that bauxite can also be directly dissolved in such an ionic liquid. This opens up interesting options to win also other elements contained in the bauxite such as iron or titanium.

Another interesting electrochemical approach was recently suggested for the upcycling of aluminum scrap through a solid-state electrolysis process using molten salts.^68^ The approach produces aluminum with a purity comparable to that of primary aluminum from scrapped cast alloys. The energy consumption has in the paper been estimated to be less than half that of the primary aluminum production process.

Further options are to replace the outdated techniques of leaching in an autoclave, calcination in a rotary kiln, and electrolysis in cells with Söderberg anodes with the altogether up to 20% lower-energy solutions of leaching in a tubular reactor, calcination in a fluidized bed reactor, and electrolysis in cells with prebaked anodes and implementation of the latest cell technology.

Another important emission problem in aluminum production is the evading fluorides that stem from the molten cryolite and other aluminum fluorides required for electrolysis. Depending on the anode technology used (e.g., Söderberg, Prebake, Prebake plus Söderberg designs), these emissions reach values of about 0.6- 1.7 kg fluoride per tonne of aluminum produced. They can be reduced through improved processes for charging the electrolysis cells and improvements in anode technology.

**Figure 142:**
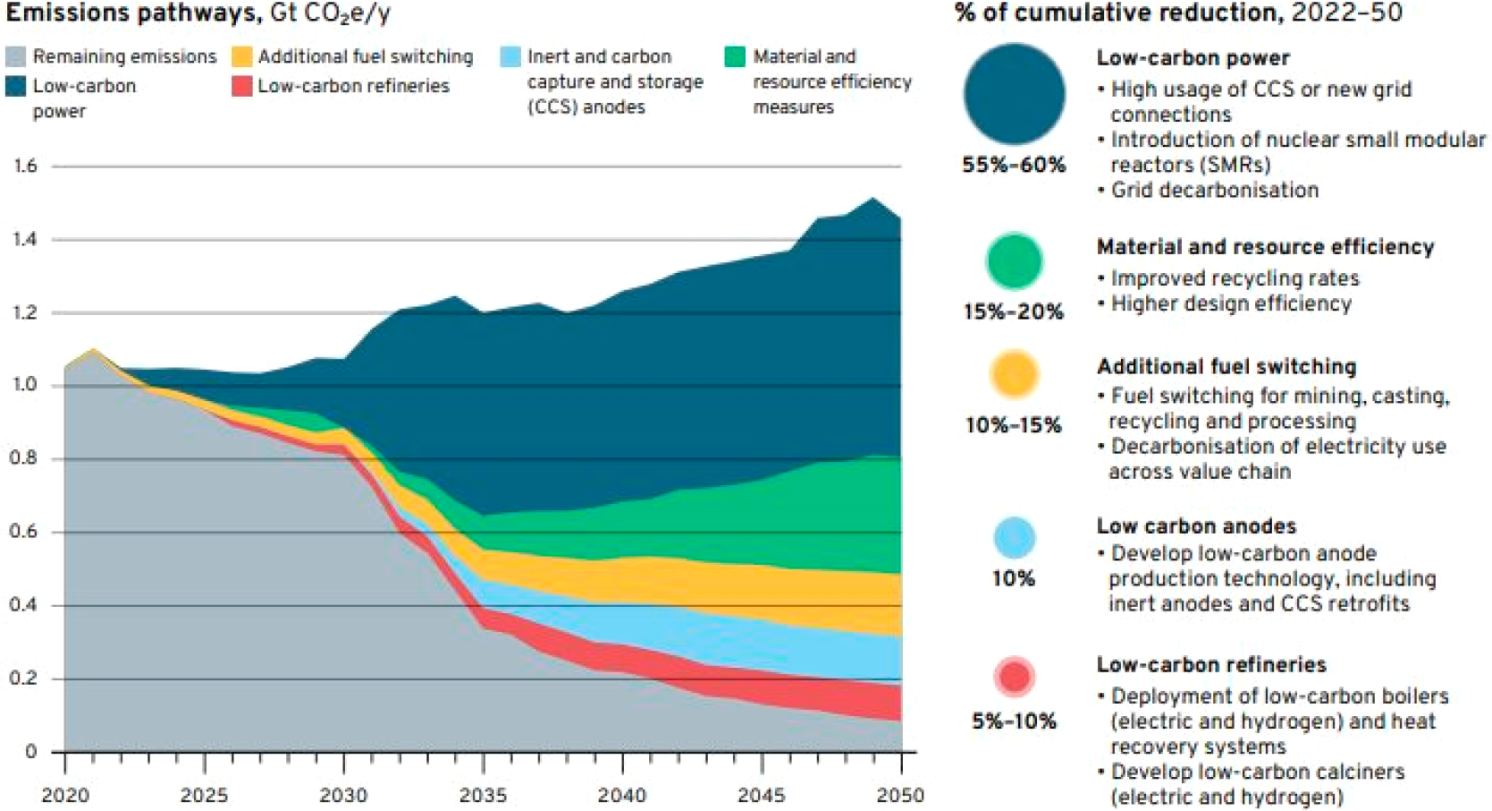
Different possible measures and leverage effects over time for reducing the CO_2_ emissions in the aluminum industry. The data are shown in terms of tonnes of CO_2_ equivalent emissions per year. It can be seen that by far the biggest contribution would come from replacing fossil electrical energy by renewable electrical energy.^187^ The figure is reproduced with permission from ref (187). Copyright 2021, International Aluminum Institute.

Figure 142 gives an overview of different pathways and their effects over time until 2050 for reducing CO_2_ emissions in the aluminum industry. The figure points at those measures with the probably highest leverage for improving sustainability. The data are shown in terms of tonnes of equivalent CO_2_ emissions per year. It can be seen that by far the biggest contribution will have to come from replacing fossil electrical energy that is currently predominantly used in electrolysis by renewable electrical energy. It must be emphasized though that different companies use already today different electrical energy sources so that consequently also large differences apply among the producers. The second biggest effect would come from increasing the fraction of recycled material that is used for making new alloys, i.e. switching from primary to secondary synthesis.^7,51,263^ The third most influential effect could come from replacing any type of fossil fuel currently used along the manufacturing chain by sustainable electricity (such as for instance the fuel used for mining, benefication, heat treatment, etc.). The fourth target of high relevance is the introduction of inert anodes or of low-carbon anode materials, respectively.^248,522^

#### 7.7.5 Production of Steel via Electrochemical Processes

70% of the iron globally consumed is made via reduction in blast furnaces using fossil reductants, qualifying this metal and this production route as the largest single CO_2_ emitter on earth. An alternative processing option is electrolysis in molten salt, aqueous, or oxide-based electrolytes.^176,523−525^ Among these approaches molten salt electrolysis has reached the most mature stage. In molten salt electrolysis, the Fe_2_O_3_ iron oxide (hematite) is dissolved in a salt solvent containing SiO_2_, MgO, Al_2_O_3_ and CaO at temperatures between 1400 and 1600 °C, depending on the specific chemical composition of the molten salt solvent. An electric current is then passed through this iron oxide containing molten salt liquid. The negatively charged oxygen ions migrate to the positively charged anode, producing gaseous oxygen that is released into the air or captured as feedstock for the chemical industry. The positively charged iron ions migrate to the negatively charged cathode where they are reduced to elemental iron. If the electricity used for operating the electrolysis is of fossil-free origin and carbon-free electrodes are used (e.g., molybdenum cathodes), the iron can in principle be produced without emissions of CO_2_.

While the pig-iron that is produced by the conventional blast furnace and oxygen converter route is rich in carbon and other elements, such as silicon, sulfur, phosphorus and manganese, molten salt electrolysis provides chemically relatively pure iron, owing to its selective cathodic deposition.

For comparison, a competing alternative sustainable iron making route is solid-state direct reduction, where a metallic iron sponge with up to 85–95% iron content is produced, depending on the type of ore and reductant mixture used. These mostly gangue-related impurities need to be removed in the electric arc furnace and/or in a subsequent secondary ladle metallurgical treatment. However, it must be noted that direct reduction of iron oxides is currently and also in the near future operated with methane or syngas as reductants, owing to insufficient availability of hydrogen. This means that direct reduction still emits CO_2_, although the total emissions are 50–75% below the emission balance of the blast furnace plus converter route. Electrolysis does not need to go through such a chemical buffer substance as hydrogen, but it can make direct use of sustainable electricity. Electrolysis has therefore potentially by far the highest total energy efficiency of all iron oxide reduction methods. The prerequisite for this technology to play out its advantage is that only sustainable electricity is used to operate the electrolysis cells, as otherwise the carbon footprint from the provision of the primary electrical energy source (e.g., from coal-fired power plants) must be priced in so that the greenhouse gas balance becomes very poor.

As with all hydrogen-based reduction methods, it must always be taken into account and billed in that the green hydrogen must be provided via electrolysis, which in turn has a comparatively low efficiency. This means that over the entire reduction chain—if one takes into account the production of hydrogen as an intermediate storage for energy and as a reducing agent—direct electrolysis is a comparatively much more energy-efficient solution. Another advantage is the recent progress in development of carbon-free electrodes.^526−528^ Another interesting option is the use of molten residual materials from the mining industry containing iron oxide as new feedstock in this process. One possibility for this is bauxite residues, which are an iron-rich waste product from alumina production. An interesting alternative to the high-temperature molten salt electrolysis is the recent progress in low-temperature electrolysis with ionic liquids as solvents.^529,530^ Details of these methods with a focus on open research questions will be discussed in a separate section below.

A disadvantage of the molten salt electrolysis iron reduction technology is its comparably low reduction rate. It must be generally considered in this field that any technology to make green iron must be scalable to match the huge quantities required by the markets. Another challenge of this technology is the variability of the sustainable electrical power supply, which is—when coming from solar or wind energy—not constant. This means that the operation of the electrolysis cells must be designed in a way to cope with variable power supply, e.g. when neither enough wind not solar power is available through the grid. This means that the cells must be designed in a manner that the power supply can be adjusted and even be entirely interrupted. This can also be seen—if it works—as a systemic advantage of this approach because it would translate variable sustainable electricity to the high energy that is stored in the form of reduced metal. This means that electrochemical iron production is in itself not just a reduction method for producing metals from oxides, but it can also be regarded as a potentially efficient energy buffer technology (much like aluminium production). The main incentive here is therefore a high flexibility in the operation of the electrolysis in an energy system that uses a high share of renewable energies. Another important aspect of the technology is that the molten salts in the high-temperature version of this technology are very aggressive, which leads to very strong wear and aggressive interactions with the insulation material and with the electrodes. The development of carbon-free electrode materials is also of special importance here, as otherwise the burnoff of the graphite electrodes would result in considerable CO_2_ emissions.

Another electrochemical approach to iron production was developed by Judge et al.,^531^ yet not for the reduction of iron oxides but for direct decarburization of liquid iron–carbon mixtures. This process thus falls—strictly speaking—not into the category of primary synthesis methods, but it could help to solve one essential problem of the current steel making approach, namely, to eliminate the CO_2_ emissions created by the conventional basic oxygen converter, Figure 119 and Figure 122.

The scientific background behind this is that the blast furnace operates in or near the eutectic point of the iron-carbon system during tapping and thus large amounts of carbon are transferred into the liquid iron by partitioning. This carbon must then be removed downstream from the high eutectic content back to the proportion that is actually needed in commodity wrought steels (i.e., 0.01–0.3 wt % compared to 4.3 wt % in the eutectic point of the iron-carbon phase diagram). Thus, a paradoxical situation in classic steel production is that very high amounts of carbon are first added to the material in the blast furnace, which then have to be eliminated again in the converter, which leads to very high CO_2_ emissions. This is the point where electro-decarburization of such molten iron-carbon mixtures could become interesting. In the process suggested by Judge et al.,^531^ the direct decarburization is achieved by introducing an electromotive force between the molten iron-carbon alloy and the slag which acts as an electrolyte. When using anodic polarization, the oxide anions from the slag discharge directly on the carbon that is dissolved in the molten material, producing molecular CO.

Table 44 lists some topics for basic research on electrowinning and electrorefinement of iron.

**Table 44:** Opportunities for Basic Metallurgical Research on Electrowinning of Iron

Finding molten salts for lower operating temperature
Inert and graphite-free electrodes
Mechanisms of low-temperature electrolysis
Robust and abundant ionic liquids for low-temperature electrolysis
Electrolysis cell operation under variable electrical power supply
Electrolytes with better properties in terms of viscosity and conductivity
Improved refractories for the electrochemical cell
Improved electrode kinetics

#### 7.7.6 Titanium Production by Electrolysis

Like most other metals, also titanium can be extracted from its oxidized state through electrolysis. Several electrochemical extraction processes for titanium have been developed during the last few decades. Recent work on titanium production from molten salts or even via ionic liquids by electrochemical reduction has shown promising results.

One promising approach is the use of a self-consuming anode in this context. During the electrolysis, titanium dissolves in ionic form from the anode, and titanium metal deposits at the cathode from the molten salts bath. Like in other electrolysis processes used for metal extraction, the advantage of this method is that in such an electrolysis cell the metallic titanium and the feed material are separated, at the anode and the cathode, respectively. Also, it provides titanium of high cathodic purity. Different types of consumable anodes have been studied in that context, for example composite anode materials which consist of a mixture of titanium oxide and titanium carbide, titanium suboxide and carbon or a bulk titanium oxycarbide anode.

Current studies on alternative anode materials include the quasi-ternary systems titanium nitride, titanium oxide and titanium carbide, including also aspects associated with the electrical conductivity of the resulting composites.

Molten salt electrochemical reduction of K_2_TiF_6_ from LiF-NaF-KF melts has recently shown high electrical current efficiency (80–85%). Also, such processes allow a continuous operation protocol. Yet, insufficient redox cycling and the formation of dendritic titanium coatings are often still severe limits to the application of such electrochemical reduction methods. The dendritic titanium deposition, low efficiency as well as insufficient purity of the reduction product were also problems in other electrochemical reduction process variants.

Another interesting approach is to use ionic liquids instead of molten salt electrolysis. It was for instance shown that the electrolytic reduction of TiCl_4_ from ionic liquids was cost-effective and proceeded at temperatures as low as 75–125 °C, which is far below the temperatures needed in any other titanium extraction methods currently in use.

Table 45 lists some topics for basic research on electrowinning of titanium.

**Table 45:** Opportunities for Basic Metallurgical Research on Electrowinning of Titanium

Development of tailor-made ionic liquids
Improved anode materials
Avoidance of dendrite formation during electrochemical reduction

#### 7.7.7 Precious Metal Extraction from Electronic Scrap via Electrolysis

Due to the special composition of the scrap, discarded electronic equipment places special demands on the recycling process. For example, one tonne of high-value scrap from electronic devices can contain about 1 kg gold, 6 kg silver, 12 kg aluminum, 20 kg tin and up to 200 kg copper. The scrap material which usually contains the highest amounts of these metals includes printed circuit boards, random access memory parts, plug-in cards, connectors, hard disk boards, integrated circuits of any kind, processors as well as mobile phone boards.

Electronic waste principally poses a multi-metal recycling challenge and must be processed in a multistage recycling process chain. A melting furnace or pyrolysis step can be used to separate the circuit boards made of glass fiber and polymer from metals that have higher melting points. This remaining metal mixture primarily contains copper, aluminum, cobalt, nickel, silver, gold, tin and many other materials. A melting unit heats the slag further to remove impurities.

The metals are then processed into powder and chemically separated from each other in leaching tanks. Here, copper, cobalt and nickel can be separated by electrolysis. Silver, gold, platinum and other precious metals remain in the anodic mud of the electrolysis cells and are separated from each other by chemical reactions with chlorine or further electrolysis along their respective positions in the potential series to recover the pure metals.

Although these separation protocols to recover the metals from the polymers and the required subsequent extraction and refinement processes require huge amounts of energy, the approach is still significantly more environmentally friendly than the production of new metals from ores. About one-third of the annual gold production comes from newly melted scrap gold. The other two-thirds go through a complex process: the ore has to be mined and transported and then the rock separated from the pure gold. The separation is done either chemically on the basis of toxic mercury or cyanide lye. Alternatively, the ore is heated with borax, which also requires large amounts of energy. Recycling thus offers a comparatively gentle way to obtain the precious metal.

However, environmentally friendly processes that are economically worthwhile are still rare in this field.^99,234,500,532^ Currently, it is being investigated whether certain sulfur-containing compounds dissolve gold in a targeted and resource-saving way. It could be shown that the contained gold can be recovered selectively and quickly through the so-called thiol-assisted leaching. Normally, gold is recovered from the anodic sludge in the electronic scrap recycling described above by a hydrometallurgical process using cyanide leaching. However, this process is highly polluting, does not dissolve gold very selectively and produces a lot of hazardous waste. Alternative attempts have therefore been made to dissolve gold in organic solutions. Sulfur compounds turned out to be well suited for such processes. In studies on the selective extraction of the gold from electrical scrap with the help of such organic solutions, it was observed that the gold dissolved best when using pyridinethiol compounds and hydrogen peroxide plus the organic solvent dimethylformamide.

Alternatives to the hydrometallurgical and electrometallurgical extraction methods from electronic waste are based on pyrolysis in conjunction with pyrometallurgical methods. For example, pyrolysis plants for the recycling specifically of printed circuit boards are already operated today that produce metal concentrates in a pyrolysis furnace under exclusion of air.

Prior to pyrolysis the first step must always be a carefully removal of the most disturbing contaminants, i.e. iron and aluminum. For this purpose, the printed circuit boards are always first shredded with subsequent separation of ferrous metals, which make up about 20–25% by weight, and aluminum, which amounts to about 10% by weight. All organic substances are fed in gaseous form into a postcombustion chamber and are burnt. The resulting metal concentrate is processed in downstream smelting furnaces.

### 7.8 Bio-hydrometallurgy and Its Role for Sustainable Metallurgy

#### 7.8.1 Introduction to Bio-hydrometallurgy

Bio-hydrometallurgy encompasses a wide range of metallurgical aggregation, leaching and treatment methods that make use of biological agents such as fungi and bacteria.^454,533,534^

A number of bacteria and several types of filamentous fungi are used in special hydrometallurgical process chains for biological metal-winning. These approaches make use of specific properties of these highly specialized and selectively acting microorganisms; particularly, they are used to leach metals from solids and precipitate metals from solutions, bind them to biomass or form biogenic minerals, usually in the form of nanoparticles.

Bio-hydrometallurgy can be successfully employed to extract and recover metals such as copper, zinc, cobalt, nickel, gold and uranium, both from ores, but particularly also in the case of the often complex multi-metal recycling cases encountered in consumer electronics waste streams.^424,535−537^

A specifically promising research branch of bio-hydrometallurgy lies in metal recovery from mixed material waste solutions through biological wet-chemical and electro-wet-chemical processes, where the metabolism of mostly sulfur- and metal-oxidizing bacteria is used.^361,457,533,538,539^ These biological agents are used to convert minerals, mostly sulfides, such as zinc or copper sulfides into water-soluble leachable sulfates through their oxidative energy metabolism processes.

Bacteria used for this purpose are for example the sulfur bacteria *Acidithiobacillus ferrooxidans* and *Acidithiobacillus thiooxidans*, the iron-oxidizing bacterium *Leptospirillum ferrooxidans* and the sulfur- and iron-oxidizing *Archaea acidianus brierleyi*,^270,457,501,540^ where the latter ones are acidophilic, i.e. acid-loving agents. The sulfur oxidizers even produce sulfuric acid themselves through sulfide and sulfur oxidation. *Acidianus brierleyi* is also thermophilic. This means that it can unfold its metabolism processes also at elevated temperatures. In the leaching process, *Acidithiobacillus ferrooxidans*, *Leptospirillum ferrooxidans* and *Acidianus brierleyi* oxidize divalent to trivalent iron, while *Acidithiobacillus ferrooxidans*, *Acidithiobacillus thiooxidans* and *Acidianus brierleyi* oxidize elemental sulfur to sulfuric acid. Water-insoluble sulfides are abiotically oxidized by trivalent iron, forming elemental sulfur. The elemental sulfur is then oxidized into sulfuric acid by sulfur bacteria. During the abiotic sulfide oxidation by trivalent iron, this is then reduced to divalent iron and then biotically reformed again by oxidation. The abiotic and biotic oxidation of the sulfide releases the heavy metals from the sulfide minerals as dissolved ions. Iron- and sulfur-oxidizing bacteria closely cooperate with their respective metabolism processes together in this way.

Methods from bio-hydrometallurgy might gain particularly momentum for the extraction of rare, precious and radioactive metals. While many of the mass-produced metals and alloys such as steels, nickel and chromium (when bound in stainless steels), copper, aluminum and lead are already nowadays recycled in rather high proportions, between 50% and 90%, rare and precious metals often see much lower recycling rates. Elements such as the platinum group metals, gold, silver, neodymium or samarium, to name but a few, are much less frequently recycled from scrap and post-consumer waste such as from scrapped electronic, catalyst and magnetic equipment. Particularly the recycled fraction of many metals that are urgently needed for making products that serve the sustainable energy supply and the electrification of transport, households and industry such as rare earths, germanium, gallium or indium as well as most catalysts is still generally less than 1% globally.^3,11,70^ In this field bio-hydrometallurgical recovery methods could make an important contribution to recycling, especially of critical and rare metals. Bio-hydrometallurgical processes have been so far successfully deployed for bio-leaching from wastes and residues such as mine dumps, ashes from waste and coal incineration, slags, electroplating sludges and to some extent also from electronic scrap.

#### 7.8.2 Bio-leaching, Bio-oxidation and Biomineralization for Metal Recovery

**Figure 143:**
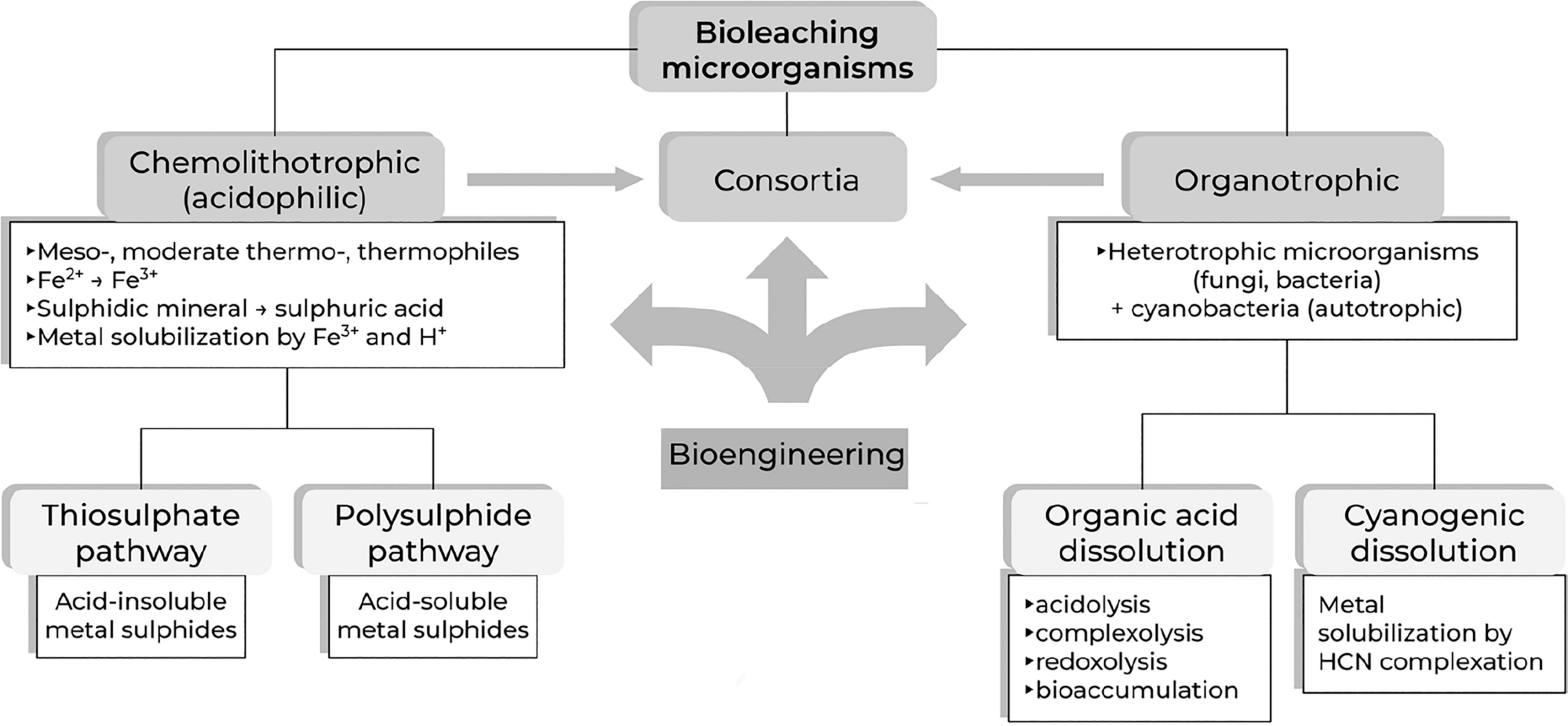
Principles of biomining and bio-leaching microorganisms and mechanisms.^538^ The figure is adapted from the paper of Santomartino et al (ref (541)). Copyright 2022, Springer, Creative Commons Attribution 4.0 International License.

Bio-leaching and bio-oxidation are specific techniques used in the field of bio-hydrometallurgy. In bio-leaching, metal sulfides are dissolved via oxidation reactions in acidic solution by means of azidophilic iron(II) and sulfur-oxidizing bacteria, whereby the metals go into solution and are recovered via a subsequent solvent extraction, Figure 143.

Bio-leaching for copper recovery from sulfide poor ores is today the most significant application of bio-hydrometallurgical methods, and 5–8% of the world’s copper production was recovered with the help of biometallurgy.^454^ Copper is predominantly leached from sulfidic ores containing mostly chalcopyrite, which may also contain pyrite and galenite. This produces sulfuric acid and the soluble, blue-colored copper sulfate. The copper is extracted from the solution by so-called cementation: The divalent copper ions present in the solution are reduced with elemental iron, that can be for instance fed from steel scrap, into elemental copper, which precipitates. The iron goes into solution instead in the form of divalent ions. The increased demand and the simultaneously decreasing stock of copper leads to the circumstance that mining had to be pushed into ever deeper zones in recent years. Energy and development costs increased, so that the more cost-effective bio-leaching is used.

However, bio-leaching is not only suited for the recovery of copper but also for nickel and cobalt.^361,455,539^ Bio-oxidation of refractory gold ores is also conducted in stirred tanks.^454,456^ Unlike bio-leaching, in bio-oxidation the valuable metal gold does not go into solution, but it is released from the sulfidic ores by the oxidizing acidophilic bacteria and subsequently complexed in solution using cyanide. Biomining is thus evolving into an environmentally friendly and economical alternative to conventional processing methods for poor ores and complex ores. The advantages of biomining include lower energy consumption and the avoidance of sulfur and CO_2_ emissions. Most of the scientific works were so far conducted in the fields of bio-leaching of sulfide ores, laterites, and metal extraction from poor minerals, about the cultivation of bacteria and inoculation of technical equipment, and for the biotechnical treatment of waste.

As discussed for the case of copper, bio-leaching and bio-oxidation have so far industrially mainly been used in the processing of sulfide ores, where the microbially catalyzed oxidation of sulfide sulfur is used for energy gain and growth of autotrophic microorganisms and no additional organic carbon source is required. For silicate, carbonate, and oxide ores, there is a need to add reduced organic carbon compounds (e.g., glycerol) for organoheterotrophic bacteria or fungi or reduced sulfur compounds (e.g., elemental sulfur) for lithoautotrophic bacteria as an energy source in the bio-leaching process, which requires additional cost and more complex process control.

Another application example is the biowinning of radioactive metals, specifically of uranium. For recovering this sensitive metal from minerals or from contaminated waste which mainly contains tetravalent uranium, especially uraninite (UO_2_), bacteria can be used.^437,542^

The leaching mechanism of organoheterotrophic bacteria and fungi (heterotrophic leaching) is based on acidolysis by means of the release of organic acids from the cells (oxalic acid, citric acid, gluconic acid, fatty acids), on complexation of metals by means of the release of chelating agents (e.g., citrate, siderophores) and also on the chemical reduction of oxidized iron compounds. In contrast to the acidophilic, lithoautotrophic archaea and bacteria, which leach at pH values below 3, the pH values of heterotrophs are in the weakly acidic, neutral, or alkaline range. Since most of the valuable metals are present as cations in solution only at low pH values, chelation is of great importance in heterotrophic bacteria and fungi. However, too low solution concentrations are currently yielded for economic processing of most ores.^454,534,535^

A promising recent research trend is the use of bioelectrochemistry for bio-leaching.^533,543^ Combining biological metabolism mechanisms with electrochemistry enables us to increase for instance copper recovery in the bio-leaching of primary copper sulfides, such as chalcopyrite. While metals go into solution during bio-leaching, the methods of biosorption, bioaccumulation, biomineralization, and also bioelectrochemistry aim at winning the metals from solutions. In that context the process of biosorption describes the sorption of metals to biomass such as for example to biological cell surfaces or to biomolecules. Biosorption has been studied for many metals such as for platinum group metals, copper, and gold; for actinides such as uranium and thorium; as well as for lanthanides such as cerium, europium and ytterbium and their various isotope forms.^544^ Cases where living cells also take up metals into their respective cell interiors are referred to as bioaccumulation.

The metals contained in leaching solutions can be obtained by various biological processes, e.g. iron by biological oxidation of Fe(II) to Fe(III) and subsequent precipitation as iron oxyhydroxide and transition metals such as copper and zinc as metal sulfides after precipitation with hydrogen sulfide via biological sulfate reduction.

Another field of interest lies in bio-leaching for metal recycling from mine dumps, sediments and soils. Metal ore mining produces residues in the form of overburden dumps and ore processing residues (tailings). Since the residues are usually complex, polymetallic mixtures of substances, established processing methods are often complicated and uneconomical, so that bio-hydrometallurgy, which is less capital-intensive, can be a viable alternative. Processing residues from the mining industry, such as mine dumps, ponds and tailings, often still contain considerable amounts of residual metals which are sometimes even higher in content than in their original ores. Often such mining waste deposits can also contain strategically relevant and rare or even precious metals that were of less economic interest and/or too diluted for extraction at the time of mine operation and the extraction of the primary target metals.

Bio-hydrometallurgical reprocessing of tailings by biomining does not require further comminution. Bio-leaching in stirred tanks or in stockpiles where the fine-grained tailings are deposited as thickened slurry on rock fragments represent reasonable process options. First tests have shown the recovery of gold by bio-oxidation and also the recovery of copper, nickel, cobalt, silver, and uranium via bio-leaching methods.^537,545^ Recovery of cobalt from residues of flotation of copper ores was also demonstrated using bio-leaching. Elimination of heavy metals from sediments and soils can be achieved via phytoremediation or microbial bio-leaching. Heavy metal removal (cadmium, zinc) from an aquatic sediment by bio-leaching with the addition of sulfur using biogenic sulfuric acid formation has been also demonstrated.

Recycling metals from industrial waste using bio-leaching is another very promising area of bio-hydrometallurgy. However, industrial wastes containing metals are usually not in the form of metal sulfides, so that bio-leaching processes developed and optimized for biomining cannot be readily transferred to metal recycling. The reason is that the metals that accumulate in typical industrial waste products are instead often bound as oxides, hydroxides, phosphates, carbonates, and silicates, suggesting the use of alternative methods, i.e. reductive bio-leaching, acidolysis and chelation. In the case of acidolysis, biogenic sulfuric acid formation by means of oxidation of elemental sulfur by *Acidithiobacillus* has been used in several studies. Organic acid-forming heterotrophic bacteria and fungi are also often used. In addition, cyanide- or metal organocomplex-forming microorganisms play a role. Bio-hydrometallurgical metal recovery from ashes, slags, sludges, dusts, spent catalysts, and electronic scrap has also been demonstrated.^270,454,534^

Examples of efficient bio-leaching with sulfur addition in bioreactors with organisms of the sulfur-oxidizing genus *Acidithiobacillus* in the laboratory resulted in high metal yields for various industrial residues.^540,546^ Examples are metal recovery from electroplating sludges by using *Acidithiobacillus thiooxidans* where 100% chromium, 95% copper, and 85% zinc were recovered.

Another example of metal recovery from press filter residues of titanium oxide production with the same bacillus was shown to function for the extraction of nearly all of the copper and zinc and most of the chromium and vanadium. Similar rates were observed for the case of metal recovery from sewage sludges with moderate and strong acidophilic sulfur oxidizers such as *Acidithiobacillu*s with 42–100% copper, 15–57% nickel, 48–100% zinc, 9–100% chromium, 17–78% cadmium, 9–47% lead and most of the manganese. Several interesting results on metal recovery from waste Ni-Cd batteries with sulfur oxidizing microorganisms where most of the cadmium and up to two-thirds of the nickel was extracted have been presented.^547,548^

Only in a few instances did biological leaching with sulfuric acid—which is produced when elemental sulfur is heated—result in greater metal recovery than did pure chemical leaching with sulfuric acid. However, because sulfur is less expensive than sulfuric acid, bio-leaching is frequently less expensive than chemical leaching.

Furthermore, recycling of expensive metals like platinum group elements and gold has been demonstrated at the laboratory scale, using both *Acidithiobacillus* and cyanide- or metal-organocomplexing heterotrophic fungi and bacteria such as the cyanide-forming *Chromobacterium violaceum* or the sulfate reducer *Desulfovibrio desulfuricans*.

Another biometallurgical process is biomineralization.^269^ In the context of metal recovery this process refers to the microbiologically influenced chemical nucleation, growth and precipitation of metals, also referred to as bioprecipitation.^549,550^ Biomineralization encompasses a variety of processes that have been well studied for many species that use for instance calcite crystals for hardening their carapace structures, such as decapoda.^551^ Like for any other precipitation process from oversaturated solutions, it is based on the low-solubility product of metal compounds. One example is the separation of iron from acid mine drainage using microbial Fe(II) oxidation to Fe(III) hydroxides. A geobiotechnical process for selective iron separation in the form of the mineral swordmannite was used on a pilot scale in lignite mining.

Metals such as copper, nickel, zinc, and cobalt can be separated as metal sulfides by chemical precipitation using hydrogen sulfide, and the precipitated pure metal fractions can be obtained by varying the pH value of the polymetallic solutions during the precipitation process.^552^ The hydrogen sulfide can also be produced by microbial sulfur or sulfate reduction using sulfur- or sulfate-reducing bacteria. Recently, also biogenic hydrogen sulfide formation even at low pH values has been made possible in several laboratory studies by cultivating acidophilic and acid-tolerant sulfate-reducing bacteria. These bacteria exhibit high tolerance to acidity and metals, so that complex mining and process waters can be treated in bioreactors with pH values between 1.7 and 5 and, for example, selective recovery of copper, zinc, nickel, and cobalt can be realized.^269^ Another possibility of biological recovery of pure metal or mineral fractions from solutions is biomineralization, targeting the formation of metal-rich nanoparticles by microorganisms (bacteria, fungi, yeasts, and algae). In this process, the nanoparticles can be formed both in the cells (intracellular) and on the cell surfaces (extracellular). To date, successful laboratory-scale attempts have been made to synthesize gold, silver, platinum, palladium, selenium, tellurium, silicon, zirconium and titanium nanoparticles. An example is the formation of nanoparticles of platinum or palladium in the bacterium *Shewanella algae* for recycling catalysts.

Biometallurgical methods were also applied to the recycling of battery materials, a rapidly growing challenge arising from the electrical vehicle market.^93,268,269^ For this purpose, through a hydrometallurgical process, bio-leached metals can be extracted by dissolving spent electrode materials with metabolites excreted by microorganisms (bacteria and fungi). Several groups studied the performance of treating LiCoO_2_ by chemotrophic and acidophilic bacteria.^422^ These studies investigated for example the leaching performance of several types of bacteria for lithium and cobalt extraction in different concentrations.^512,553^ Some research opportunities for biometallurgy are listed in Table 46.

**Table 46:** Opportunities for Basic Research in Sustainable Biometallurgy

Genetic design of bacteria and fungi for efficient bio-leaching
Metal-selective biometallurgy methods
Kinetics of biometallurgy
Metal-selective biomineralization

### 7.9 Phytomining: Metal Accumulation in Plants for Mining and Soil Cleaning

An interesting field for the mining of rare earth and precious metals^554−556^ and for the decontamination of heavy metal-contaminated soils^557^ is phytomining.^141^ This young branch of metallurgy involves cultivating a group of special plants which act as metallic hyper-accumulators. This means that they collect and aggregate metals from the soil and store it in the plant.^558,559^

**Figure 144:**
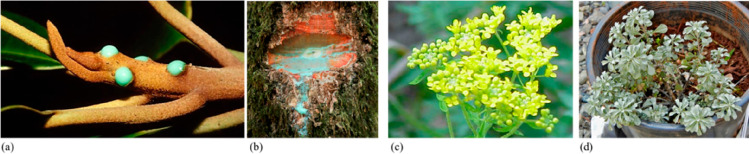
Three examples for plants studied in the field of phytometallurgy: (a,b) *Pycnandra acuminate*, (c) *Alyssum murale* and (d) *Alyssum corsicum*. These plant species all draw nickel from the ground in high quantities. Details about these plants can be found in ref (560).

Examples of such hyper-accumulator plants are mountain brightweed, Haller’s foam cress and stonewort. Extreme examples are *Pycnandra acuminata*, *Alyssum murale* and *Alyssum corsicum*, species which all draw nickel from the ground in high quantities. When for example scratching the bark of *P. acuminata*, a blue-greenish liquid emerges. This plant sap consists of up to 25% of heavy metals, Figure 144. In New Caledonia, where also large nickel deposits are located, these plants help to detoxify nickel-contaminated soil around mines. This approach takes significantly longer than conventional remediation methods; however, it is also less expensive and has advantages from a sustainability perspective. There is also an interesting side effect: the metals extracted from the soil by the plants can be profitably recycled by harvesting, drying and burning the plants. The ashes can then be further processed to extract the nickel (and other metals). Several such studies have shown that hyper-accumulator plants can extract not only nickel but also cobalt, lead and zinc.^557^ After harvesting, the hyper-accumulator plants can be cut, dried, burned and washed with acid to recover the metals from the soil. Hence, they can serve both as metal source and for detoxifying contaminated regions.

Other studies were concerned with the question how phytomining via hyper-accumulator plants can be used to extract not only nickel but also palladium, platinum or rare earth elements such as scandium and neodymium from soils.^141,554^ This might be sensible in regions or on soils in which the metals are not concentrated but are found highly dispersed in the soil.

The original reason for the plant to develop such a mechanism of hyper-accumulation may be a protection against potential enemies for which the high metal concentrations in the plants’ leaves would be toxic.^558^ This assumption is supported by the observation that animals do not eat the plants. It is also possible that this accumulation mechanism of metals serves the plants as a competitive advantage over other plant species. This is because they can thrive in locations where hardly any other plants would otherwise survive. Interestingly, studies have shown that the hyper-accumulators have developed strategies to prevent the toxin from affecting their own metabolism. Many of these plants channel the metals through the plant body and store them far away from the chlorophyll that is important for photosynthesis, for example in special storage vacuoles in the outer leaf layer.

It is also noteworthy that metal hyper-accumulators preferably grow next to former mines where the soil has high concentrations of toxic heavy metals. For most other plants, this would be a toxic environment, but hyper-accumulator plants can grow well in such areas. This is precisely why they are suitable for cleaning and restoring damaged soils. Some of these plants can take up hundreds of times what can normally be found in metals in other plants. So they have a tremendous uptake capacity for metals.

Research opportunities related to phytomining in direct metallurgical sustainability are shown in Table 47.

**Table 47:** Opportunities for Basic Research Related to Phytomining in the Context of Direct Metallurgical Sustainability

Kinetics of phytomining and phytocleaning of contaminated soil
Efficient and sustainable processing of plants used in phytomining
Phytomining of precious and platinum group metals

## 8 Better Sustainability through Improved Large-Scale Processing

### 8.1 Introduction to Metallurgical Sustainability via Lean Downstream Processing

Most of the content of this paper has so far addressed opportunities in research related to the primary synthesis of metals. The reason for this focus is the fact that the majority of the energy consumption and particularly of the CO_2_ emissions come from the primary production of metals, providing therefore also the most efficient leverage for the urgently required improvement of the sustainability of the metallurgical sector, Figure 15, Figure 16, and Figure 29. Particularly regarding the mass-produced structural metallic alloys such as steels and aluminum alloys, there are hence multiple opportunities for sustainability oriented research with high leverage, Table 3.

**Figure 145:**
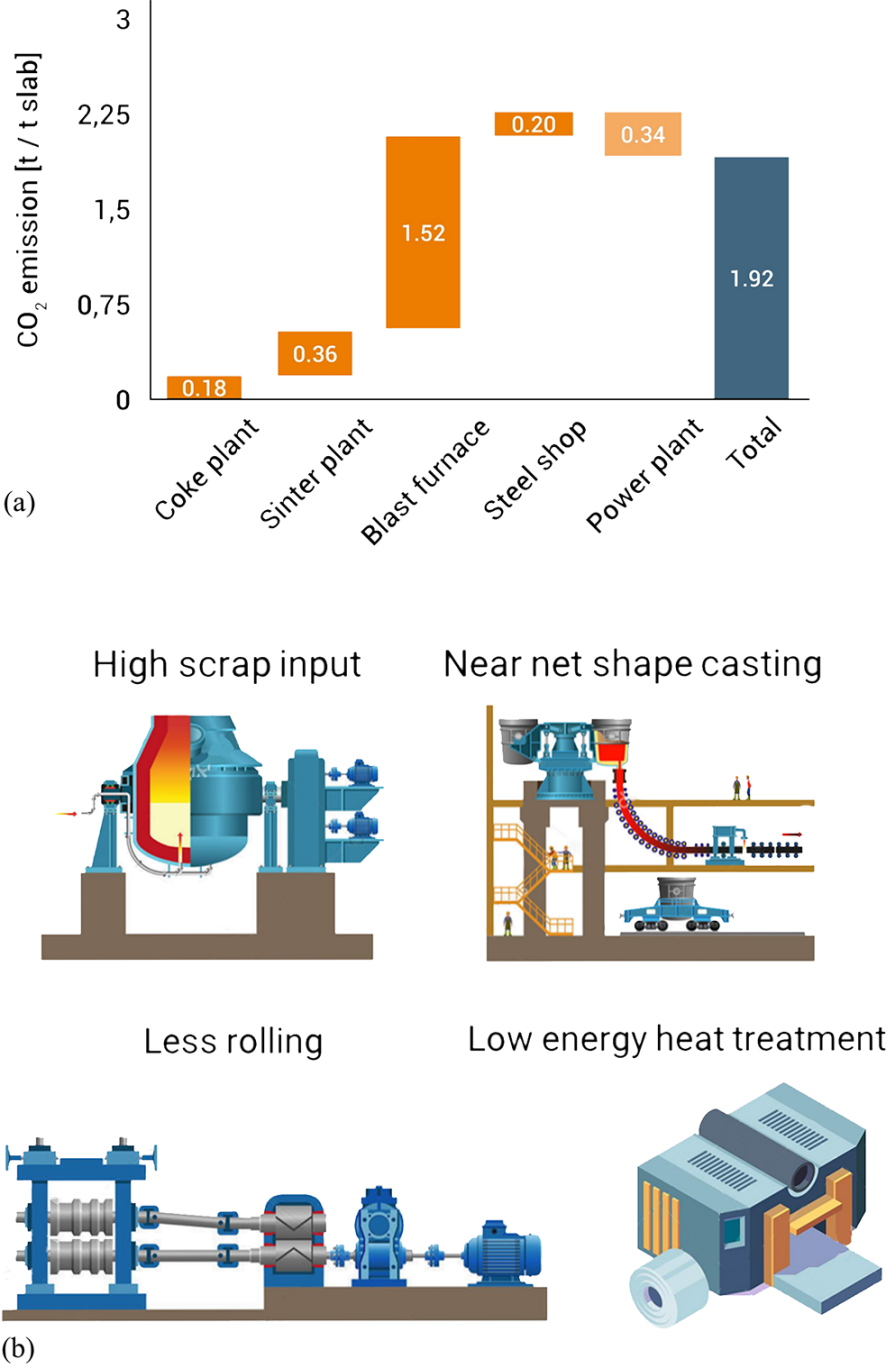
(a) CO_2_ emissions along the process chain for the case of steel. (b) Some measures for reducing energy consumption and CO_2_ emissions along the manufacturing chain of mass-produced materials such as steels and aluminum alloys.^97,108,331^

However, not only the synthesis but also the downstream processing steps of these mass-produced metals are associated with substantial amounts of energy consumption and greenhouse gas emissions,^95^Figure 145. The main reason is that these commodity metals are usually cast in the form of huge slabs with a thickness in excess of 200 mm. Compared to the much thinner typical sheet and extrusion profiles that are finally used in products, this requires thickness reductions in excess of 90%, which explains the high deformation and reheating energy that is used in this field. Therefore, this section places focus on promising energy-reduced downstream processing methods in that context.^561−563^

Sustainable lean metallurgical processing that follows after casting is concerned with all measures that allow production of the same amount of material with identical or improved quality at reduced energy costs, heat treatment times and temperatures, greenhouse gas emissions, and material waste, all at competitive production rates. This is of course not a new approach because all of these measures are typically applied anyway in industry, driven by the permanent quest for efficiency, time savings and cost reduction.

However, the relative weights of the different possible measures and their respective relevance for rendering the production of metallic materials more sustainable as well as the analysis and process control tools as well as the models, digital twins and artificial intelligence behind them have changed, opening new avenues for revisiting opportunities for enhancing the sustainability in metallurgical production through lean and efficient processing.

New ideas from basic materials science can leverage significant and sometimes unexpected progress for higher efficiency in downstream processing, ranging from the “pumping” of vacancies by cycling deformation that can accelerate nanoprecipitation in aluminum alloys and probably replace heat treatments^564^ to the use of machine learning methods for more efficient process design.^48,166,565−567^

Some key measures of sustainable production that is downstream from metallurgical synthesis are summarized in Table 48. Some of these opportunities for improved sustainability and lean production in metallurgy are apparently not topics for basic research but are rather obvious tasks in any production plant, pertaining to management, investment, general quality control and workflow improvement, etc. These topics will thus not be discussed here. Yet a few of these measures could benefit from a more basic research perspective, and these aspects will be discussed below in more detail, Figure 145.

**Table 48:** Opportunities for Basic Research on Measures of Sustainable Production Downstream from Metallurgical Synthesis

Near-net shape and thin strip casting, to reduce the required downstream hot and cold working
In-production material and by-product waste collection and sorting
In-production scrap collection and sorting
Heat treatment improvement, in terms of time–temperature cycles, furnace insulation, fossil footprint of the fuels used, low-temperature heat treatment, development of heat treatment-free alloy types
Inefficient cooling–heating cycles, increase in in-line charging (using residual heat)
Electrification of process steps in conjunction with the use of renewable energy
Better product quality generally leads to less scrap production
Using waste heat and exhaust gases from production for other applications
Create digital twins of manufacturing workflows including life cycle and by-product information
Application of artificial intelligence and simulation methods to existing sensor and probing data etc. for better quality control, process improvement, and downstream processing correction of inherited product and/or composition variations
Life cycle analysis of all manufacturing, logistic, storage, handling and value chain steps
Life cycle assessment of casting vs extrusion vs rolling and forming processes to manufacture certain parts etc. with respect to enhanced sustainability, lower scrap production, high impurity tolerance, etc.
Achieve the same microstructure and product quality with less rolling and metal forming operations, less heat treatment (low heating processing), leaner alloy compositions, higher scrap input, etc.
Less edge cutting, surface trimming, chipping and machining

### 8.2 Near-Net Shape Casting, Thin Slab Casting and Strip Casting

**Figure 146:**
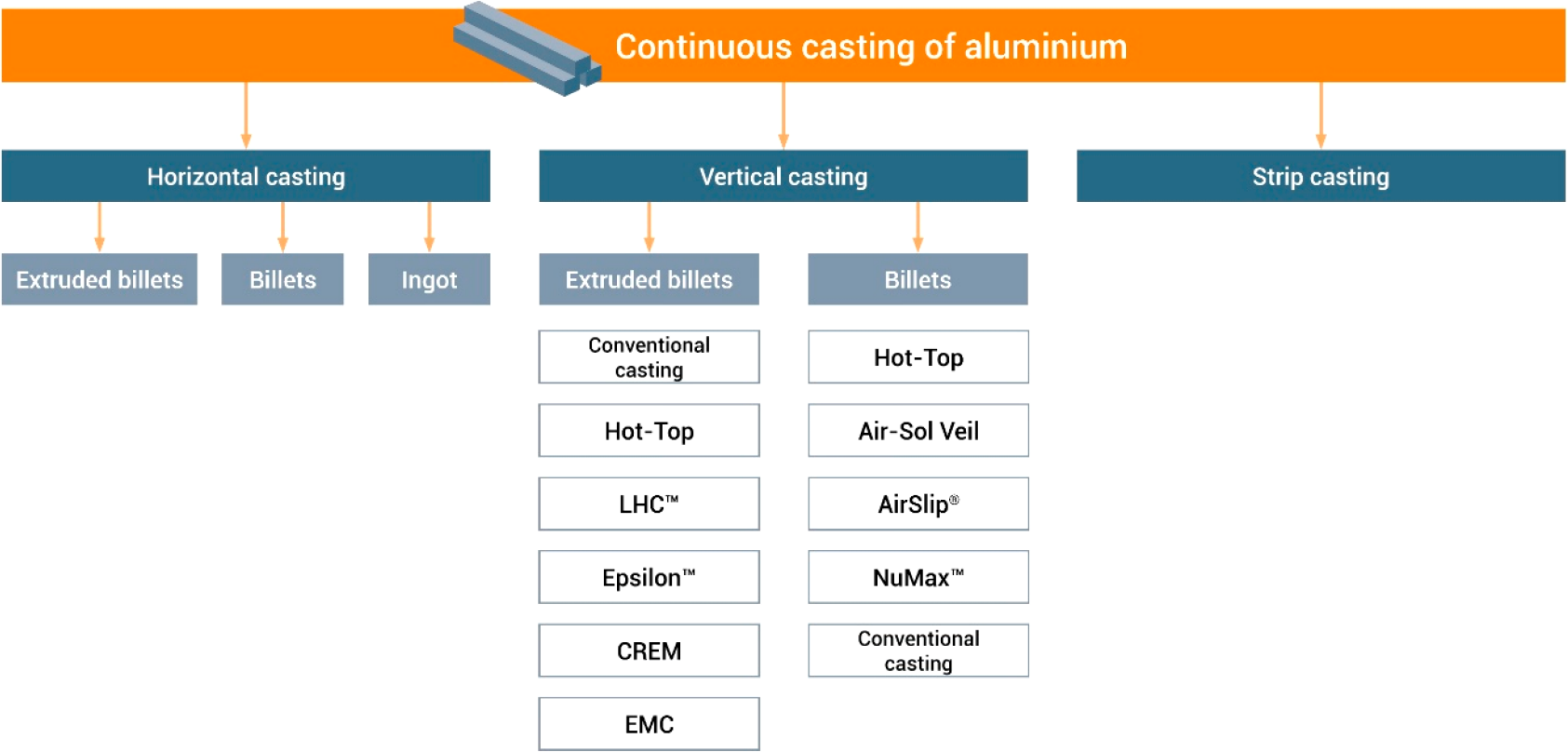
Overview of different types of continuous casting methods for aluminum alloys. LHC, low head composite casting process; CREM, casting, refining, electromagnetic process; EMC, electromagnetic casting; Epsilon, variant of EMC process; AirSlip, process variant supplying oil and mixed gas (N_2_ and O_2_) directly to the billet surface; NuMax, direct chill casting which extracts heat from the molten metal through the mold wall and through direct contact (direct chill); Air-Sol Veil, mold system working with air.

This section briefly reviews near-net shape and thin slab casting techniques as well as strip casting owing to their high leverage on sustainability, Figure 146.^561,568−570^ Near-net shape casting to dimensions that are closer to those required for a final product needs less warm and cold forming, less heat treatment as well as less machining and trimming. Therefore, near-net shape casting is generally a suited method to increase the efficiency and sustainability of metallurgical products, provided the material can be produced with the same of even better microstructures and properties.^571^

The latter point is by no means a trivial aspect because most alloys undergo substantial Scheil segregation and show high gradients in terms of grain size, grain shape, precipitation state and dispersion, and porosity, etc. through the thickness of cast products. Traditional thick slab casting processes which are thereafter subjected to large-strain warm and cold rolling plus subsequent heat treatments have therefore the higher likelihood for achieving a homogeneous through-thickness microstructure compared to materials that are made by thin slab or thin strip casting with less or no subsequent metal working, heat treatment, and homogenization. The reason for this is that the enormous additional forming work as well as the heat treatment applied in between the multiple deformation steps and the associated phase transformations and recrystallization processes usually homogenize the material on the way to the end products with their thinner dimensions in the course of hot forming.

On the other hand, the latter processes, viz. near-net shape casting methods, are associated with much higher solidification rates which in turn reduce the likelihood of chemical and microstructural gradients.^572,573^ This means that the trade-off between these several aspects must be considered when aiming at producing large flat scale materials with near-net shape casting and less forming and heat treatment. Another important aspect of near-net shape manufacturing is the often insufficient surface quality of the sheet materials.

In most conventional thick slab casting processes, steel is cast to a thickness of 180–250 mm. Afterward these slabs are first thickness reduced by reversing roughening mills and subsequently sent to continuous or reversing hot rolling mills, producing a hot strip thickness of around 2–3 mm. This means that mostly more than 98% total thickness reduction must be applied already during hot rolling alone, translating to enormous energy and investment costs. The thinner the cast slabs are, the larger are the energy savings relative to conventional thick slab manufacturing. Only about one tenth of the energy (200 MJ/t steel) is required for sheets produced by the strip casting process than for sheets worked from slabs (2100 MJ/t steel).^108,574,575^ This translates to up to 40 PJ energy savings per year. Near-net shape slab production also replaces some of the otherwise required downstream processing aggregates such as the slab reheating furnaces and rolling mills. When using near-net shape manufacturing, the heat requirement per ton of hot strip can be reduced from 1.2–1.5 GJ per tonne to 0.1–0.5 GJ per tonne. The reduced metal forming work also reduces the electrical energy requirement from 80–100 kWh per tonne of hot strip to 30–60 kWh per tonne. As the hot rolling mills are a major energy consumer in the integrated steel mill, energy consumption could be reduced by 5 to 7% overall through near-net shape casting processes. In most thin slab, thin strip, and related near-net shape manufacturing techniques, the processes of casting and rolling are closely combined. With the help of a casting nozzle, liquid metal is fed into the roll gap. The rolls subsequently have a double function, i.e. heat extraction for solidification and forming of the solidified strip. This eliminates the need to reheat the semifinished product for the forming process. Release agents based on water, graphite or emulsions are used to prevent adhesion to the rolls. The main challenges of this casting technology are the melt distribution system; metal level control; temperature control; release agent optimization; roll cooling and roll gap adjustment; microstructure control; and surface quality. Particularly the latter aspect matters when targeting sheet materials that are used for outer skin applications, e.g. in vehicles. This means that the attractiveness of the comparably low-cost and low-deformation work offered by near-net shape casting is lessened for some alloy variants by lower surface quality and lower microstructure homogeneity compared to material produced via thick slab casting, particularly for the cases of packing and automotive alloy grades.

**Figure 147:**
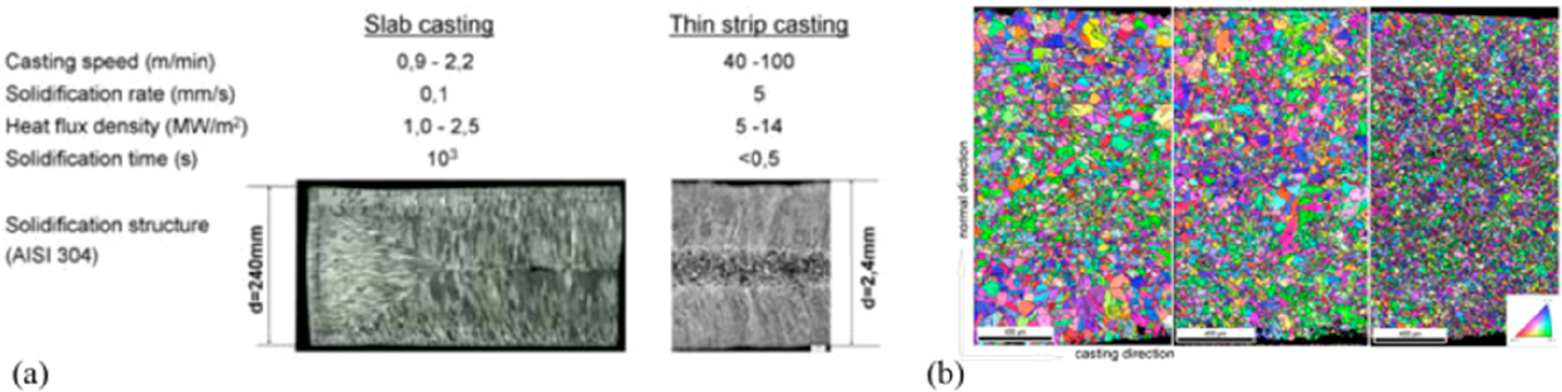
Thin strip casting of austenitic stainless steels. (a) Comparison to a regular slab. (b) Microstructure and microtexture of cast stainless steels under different casting and in-line deformation conditions, as measured by EBSD.^561^ AISI 304 is a standard commercial austenitic stainless steel with about 18 wt % chromium and 10 wt % nickel. The figure is reproduced with permission from ref (561). EBSD, Electron back scatter diffraction. Copyright 2008, Steel Research International, Wiley.

The advantages outlined above become even bigger when going from thin slab casting directly to thin strip casting. Progress in the field of 2 mm thin cast strips was made in the field of stainless steels: In a set of studies, the optimization of the microstructure and properties of thin strip cast austenitic and ferritic stainless steels was shown, Figure 147.^561,570,576^ Concerning the processing steps, the relevance of different thin strip casting parameters, in-line forming operations, and heat treatments for optimizing microstructure and properties has been studied. The microstructures and properties obtained from the different thin strip and subsequent rolling and heat treatment operations were analyzed with respect to phase and grain structures, martensite, delta ferrite, retained austenite, and texture.^573,576^ It had been reported that different process parameters lead to markedly different microstructures and profound differences in strip homogeneity. It was found that the properties of strip cast and in-line hot rolled austenitic stainless steels are competitive to those obtained by conventional continuous casting and hot rolling. This means that the thin strip casting technique not only is competitive to conventional routes with respect to the properties of the material but also represents the most environmentally friendly, flexible, energy-saving, and modern industrial technique to produce stainless steel strips, yet at moderate production rates compared to the conventional thick slab casting and hot rolling approach.^570,577,578^

In aluminum production several techniques for near-net shape casting have been introduced, mostly belt and twin roll casters, which produce thin slabs in a combined solidification and deformation process with much higher solidification rates and less required in-line and/or downstream deformation than in direct-chill (DC) casting, although at smaller overall production rates.^579,580^ Casting the products into thin 1–2 mm dimensions by twin roll casters^580−582^ can be done for both steels and aluminum alloys. In this approach a melt is poured into a gap between two rotating water-cooled cylindrical rolls, where it solidifies rapidly and produces sheets of a few mm thickness.^561^ In twin-roll casters, the metal solidifies almost entirely on the roll surfaces before reaching the roll gap. In the roll gap it undergoes a 5–25% in-line thickness reduction step. The solidification rate achieved in twin-roll strip casting of aluminum alloys depends on the strip thickness and can vary between 10^2^ and 10^4^ K/s.

For aluminum alloys, material of good quality has been produced via the twin roll route for the low-alloyed aluminum grades 1xxx and 8xxx and for 3xxx alloys.^568,583,584^ Twin-belt machines, mainly of the Hazelett design, have been used to make 3xxx and 5xxx series sheet material with moderate strength, surface requirements and good corrosion resistance. The greater solid solution supersaturation during twin-roll casting leads to finer dispersion of primary phases and finer grains elongated in the casting direction. Therefore—in contrast to the processing routes established for DC cast alloys—processing routes for producing twin-roll cast materials require adjustment.

The microstructure, particularly the grain shapes, particle density, grain size, segregation and crystallographic texture, of twin roll cast sheets evolves differently for the near-surface regions and the center regions.^573,585^ The number of dispersoids precipitated in twin roll cast aluminum alloys during the subsequent heat treatment is usually lower in the near-surface region. Grains in the central region are to a greater or lesser extent equiaxed, while those at the surface may be elongated in the casting direction or are very fine due to the recovery and recrystallization caused by the hot in-line deformation step characteristic of the twin-roll casting process.

Several overviews have been published reviewing the methods, alloy variants, microstructures and properties of aluminum and magnesium alloys as well as steels produced by near-net shape manufacturing.

### 8.3 Giga- and Mega-casting of Vehicle Body Structures

Vehicle body-in-white metallic structures usually consist of multiple individual parts that are glued, welded or bolted together. In order to manufacture the individual chassis, engine or body parts, die casting processes have long been established in the automotive industry. In this process, liquid metal is pressed into split metal molds under high pressure. While the cast metal solidifies, the pressure is maintained. With the die casting process, thin-walled workpieces with complicated shapes can be produced with a high quality finish and close tolerances. Up to now these die casting processes were mainly used for parts of moderate size only.

Recently, this technology has been applied also to large vehicle body structures, a technique referred to as giga- and mega-casting.^586^ Mega-casting works in principle in the same way as conventional die casting, only on a much larger scale. In mega-casting, a large number of parts are replaced by a single, large casting and this advantage can be exploited in the case of aluminum casting in particular where there is a need to substitute a construct made of many individual parts. In this context the recent progress in large-scale aluminum die casting processes reveals indeed several potential advantages for large-part body-in-white design compared to conventional sheet production methods. This applies both (a) to the use of cast alloys that could tolerate higher scrap fractions and therefore also a higher impurity content compared to conventional high end sheet alloys and (b) to the fact that casting requires no further downstream forming operations compared to classical sheet manufacturing and is much less energy intense. Potential disadvantages when applied to vehicle technology are reduced repairability.

An open question in that context is if heat-treatable or non-heat-treatable aluminum alloys will prevail in this field. For some parts it has already been shown that heat treatments after casting can be avoided. This is an attractive feature, as it obviously can help save costs for heat treatments, but it particularly also opens this field up for the large-volume use and further development of corresponding sink and acceptor naturally hard aluminum alloys that can tolerate higher scrap fractions than the compositionally often more sensitive heat-treatable aluminum alloys. Precipitation-hardening alloys, on the other hand, offer advantages in terms of further strength and ductility tuning by adequate heat treatments after casting.

In contrast, the conventional sheet metal shell construction method for body-in-white structures of vehicles, together with advanced gluing, soldering and spot welding methods, has developed over many decades as a robust process pathway. While a lower limit of 2–3 mm is assumed for die casting with regard to sheet thicknesses, wall thicknesses down to 0.7 mm are possible with sheet metal shells. Therefore, large-scale casting does not have in all design cases to be necessarily a more efficient solution. However, it is an alternative method that adds an interesting variant to the rather well-worn technological toolbox in car body construction, owing to the usually less demanding and more scrap-friendly alloys that can be used in this field, compared to conventional outer-skin sheet materials.

Mega-casting methods are also suitable for the redesign of specific parts of the car body construction, especially with regard to electromobility. In electrical vehicles, the battery tray is a central and essential component that needs to be integrated into the design. The advances in body construction that have been made in combustion vehicles over the years therefore only apply to a limited extent in electric cars, especially with regard to the rear end and the center of the vehicle.

In addition, the design of electric vehicles must pay particular attention to improved sustainability, since these vehicles can use renewable electrical energy efficiently and directly on the one hand, but on the other hand their entire production and their very high total weight—due to the large batteries—are far more harmful to the environment than the production of conventional vehicles powered by combustion engines. For this reason, the production of such electric vehicles should be thought of more holistically than before, not only in terms of materials, but also in terms of integrative and recyclable design, and therefore, in particular, significantly increased recycling shares should be taken into account during production, which is much easier to realize by using cast alloys than with sheet metal components.

A problematic aspect for cast alloys in that context is that in die casting there is a noticeable limitation in the service life of the die casting molds. Due to the thermal shock effect, the rule of thumb is that a die casting mold lasts only for 100,000 to 150,000 casting shots. A forming tool, on the other hand, can handle five to six million parts. This amounts to a factor of 20 to 30 in difference in service lifetime of the tools.

### 8.4 Efficient and Microstructure-Oriented Heat Treatment Design by Machine Learning

Heat treatments consume the largest part of the energy besides the synthesis of large-scale forming processes and generate substantial high greenhouse gas emissions.

It should be noted that large-scale heat treatments of the important mass-produced structural metals such as steel and aluminum essentially have two quite different tasks, namely on the one hand that the material must be softened between rolling, forging or extrusion passes, due to the often enormous required total forming degrees above 90%, in order to be formable at all and on the other hand that heat treatments are used to set desired microstructures and crystallographic textures, which are essential for the final product properties.

The energy required for this can be calculated from the integral under the flow curve (stress–strain curve) of the material, divided by the corresponding efficiencies of the forming aggregates, plus the energy of the heat treatments required between the individual forming processes. While the section above was concerned with the former question, namely, the reduction in the required energy for the metal forming processes by using near-net shape manufacturing methods, the second aspect, viz., the heat treatments, is addressed here in this section.

In essence this task reduces to finding heat treatments with lower temperatures and shorter durations, fuelled by non-fossil energy, or even to eliminating the heat treatments altogether.

Further opportunities for reduced heating costs combined with well-adjusted microstructure control are in the development of more tailor-made thermal treatments in particular. When many alloys used today were originally designed, including the processing, the thermal heat treatment profiles used for grain size, texture, or precipitation heat treatments were quite simple regarding use of thermal ramps, isothermal holding and cooling.^587,588^ An important consideration in the development of well-tailored future thermal treatments however is the large scope for designing low-energy, composition-specific and nonisothermal time–temperature profiles to control the microstructure and increase the total energetic efficiency.^564,589−591^ Such heat treatments have also to be more robust and composition-tolerant regarding the intrusion of scrap-related tramp elements and their effects on the microstructure. It is in principle conceivable in that context that for every cast slab with its specific and scrap-dependent chemical composition plus its specific charge-dependent and thus variable impurity content, the heat treatment is adjusted automatically every time a new slab arrives at the rolling stand of the heat treatment furnace, based on artificial intelligence or based on corresponding classical models on the effect of certain impurity elements on the microstructure.

In this context specifically heat treatments with nonconstant temperature control as well as specific alloy-specific simulation of heat treatments with regard to minimum energy consumption are promising avenues. Other topics include the dynamical and on-the-fly regulation and adjustment of heat treatments for alloys with high recycled material content, i.e. with a higher impurity content; replacing CH_4_-based combustion technology for furnaces with H_2_-based combustion and/or with electrical heating.

## 9 Sustainable Alloy Concepts

### 9.1 Microstructure-Oriented versus Composition-Oriented Alloy Design

From a sustainability perspective, compositionally “lean” alloys are generally preferable to “overalloyed” materials. The latter term is somewhat ill-defined. The background and motivation behind the approach of developing “lean” alloys is that the use of multiple expensive alloying elements can be avoided and the materials can be better used both as acceptor alloys of scrap and donator alloys of scrap for new alloys, when the materials have in general (a) lean chemical composition and—connected to that—(b) fairly similar composition relative to each other.^7,115^

Basically, metallic alloys can be adjusted with regard to desired properties via their global and local chemical composition or via the microstructure, i.e. the entire cosmos and all the interactions among all the lattice defects. In general, they retrieve their mechanical properties primarily through their microstructure. This means that what matters is not only global composition but also the way it helps to realize specific microstructures.

Of course the two approaches (alloy design by composition vs alloy design by microstructure) are only the extreme ends of an intrinsically much more complex interaction between the microstructure and the chemical composition, as both do not interact linearly with each other, Figure 39. For example, the chemical composition not only changes the phase composition and thus influences the occurrence of certain precipitation phases, but most lattice defects are also decorated by dissolved atoms which can substantially alter their thermodynamic and kinetic properties. This ranges from the change of the stacking fault energy and thus also the dislocation cross-slip and other dislocation interaction processes to Cottrell clouds around the dislocation cores to the chemical decoration of the grain boundaries. Also, obviously certain types and kinetics of precipitate formation and desired metastable states do not occur without specific chemical tuning.

The main line of thought however behind this comparison between microstructure-oriented material design and chemistry-oriented material design is the suggestion that the composition-dependent portion of microstructure tuning could for many alloys be likely achieved by leaner chemical compositions, that means with less use of alloying elements. This would not only substantially enhance the sustainability of metallic alloys, but it would necessarily also bring the different alloy classes chemically closer together, making them therefore mutually more scrap-compatible. This means that modern metallic alloy design should generally aim at achieving the same (or better) microstructures and material properties, but with less alloying elements.

A characteristic example, that was discussed in the section above, refers for the ternary system consisting of iron, manganese and carbon. This chemically simple system enables the design of several 100 types of metallic materials, simply by minute chemical tweaking but massive microstructure optimization.^166,592−595^

The chemical composition of a material is a conserved quantity. This means that, whenever adjusting alloys through modification of their chemical composition, any alteration of an alloy through composition adjustment will come back in the form of a modified mass balance somewhere in a circular material economy. It also means that we must design future alloys in a way that every single atom can re-enter the scrap stream into a fully circular reuse (minus entropy-related losses of course). Microstructure in contrast is not a conserved quantity and can therefore be rejuvenated infinitely, yet at the costs associated with the underlying processing, which—in itself—is also not a cost-neutral process (see section above on lean processing).

**Figure 148:**
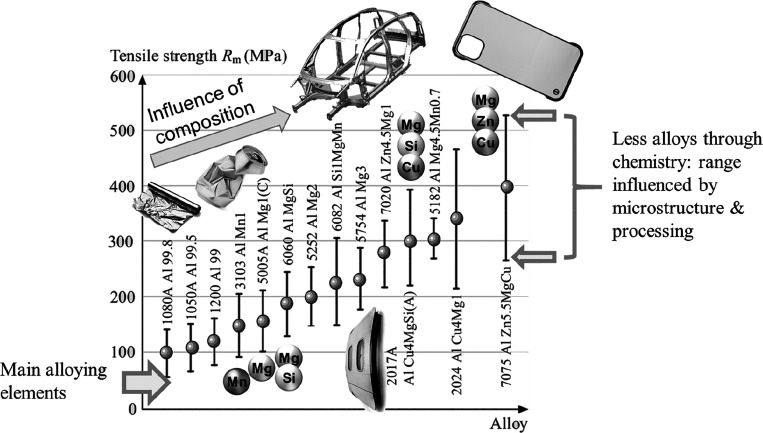
Summary of tensile strength values for various wrought aluminum materials. The ranges of the tensile strength values obtained by varying the composition are trended from left to right. The vertical change bar shows how much the strength of alloys with the same chemical composition can be affected simply by manipulating the microstructure through adequate processing. This trend example could be of particular interest for future designs of scrap-tolerant alloy variants and the underlying process and heat treatment variants.^596^ The wrought aluminum alloy groups indicated above the bars follow the standard description, indicating the main alloying element(s) in aluminum alloys: 1xxx, close to commercially pure aluminum; 2xxx, copper; 3xxx, manganese; 4xxx, silicon; 5xxx, magnesium; 6xxx, magnesium and silicon; 7xxx, zinc, copper and magnesium. The figure has been reproduced with permission from ref (7) under a 4.0 International Creative Commons license (CC BY 4.0). Copyright 2022, Elsevier.

Microstructure has the advantage that it can be altered over multiple orders of magnitude entangled with multiple spatial correlations. Examples are the interface and dislocation densities, grain size, precipitate size distribution and dispersion, etc. An example of both composition- and microstructure-guided alloy design is shown in Figure 148. The figure shows the tensile strength for several commercial wrought aluminum alloys.^596^

The data clearly show a systematic trend that with more complex chemical composition and a larger number of doping elements also the yield strength is increasing. The diagram however also shows a huge scatter in the properties for materials of the same chemical composition/the same chemical alloy variant, and this huge variation in yield strength is possible only due to differences in processing and the associated microstructure variation. The data reveal that a variation in strength of up to 50% is achieved for a maintained chemical composition.

The 1xxx group of alloys, on the left, are relatively pure packaging grades. The 3xxx series alloys, which are primarily manganese-based, are used in cans and buildings. The 5xxx and 6xxx series are medium-strength alloys. Alloys in the 5xxx series are primarily composed of magnesium, whereas those in the 6xxx series are composed of magnesium and silicon. These two alloy classes are widely used in vehicles, mobile phones, and computers.^54,191,597−599^

Copper is the primary alloying element in 2xxx alloys. They are primarily used in highly mechanically loaded parts in the aerospace industry. Aluminum can be doped with copper, zinc, and magnesium to achieve even higher strength levels, resulting in the 7xxx alloys. These are found in electric vehicles as well as aerospace applications. This means that, from left to right, the majority of strength increases are obtained by adjusting chemical compositions, as well as the corresponding heat treatment required to achieve the desired nanoprecipitation state.

In essence, the data show that in many high-strength aluminum alloys, a wide range of tensile strengths can be obtained through proper microstructure adjustment rather than through further chemistry changes.

**Figure 149:**
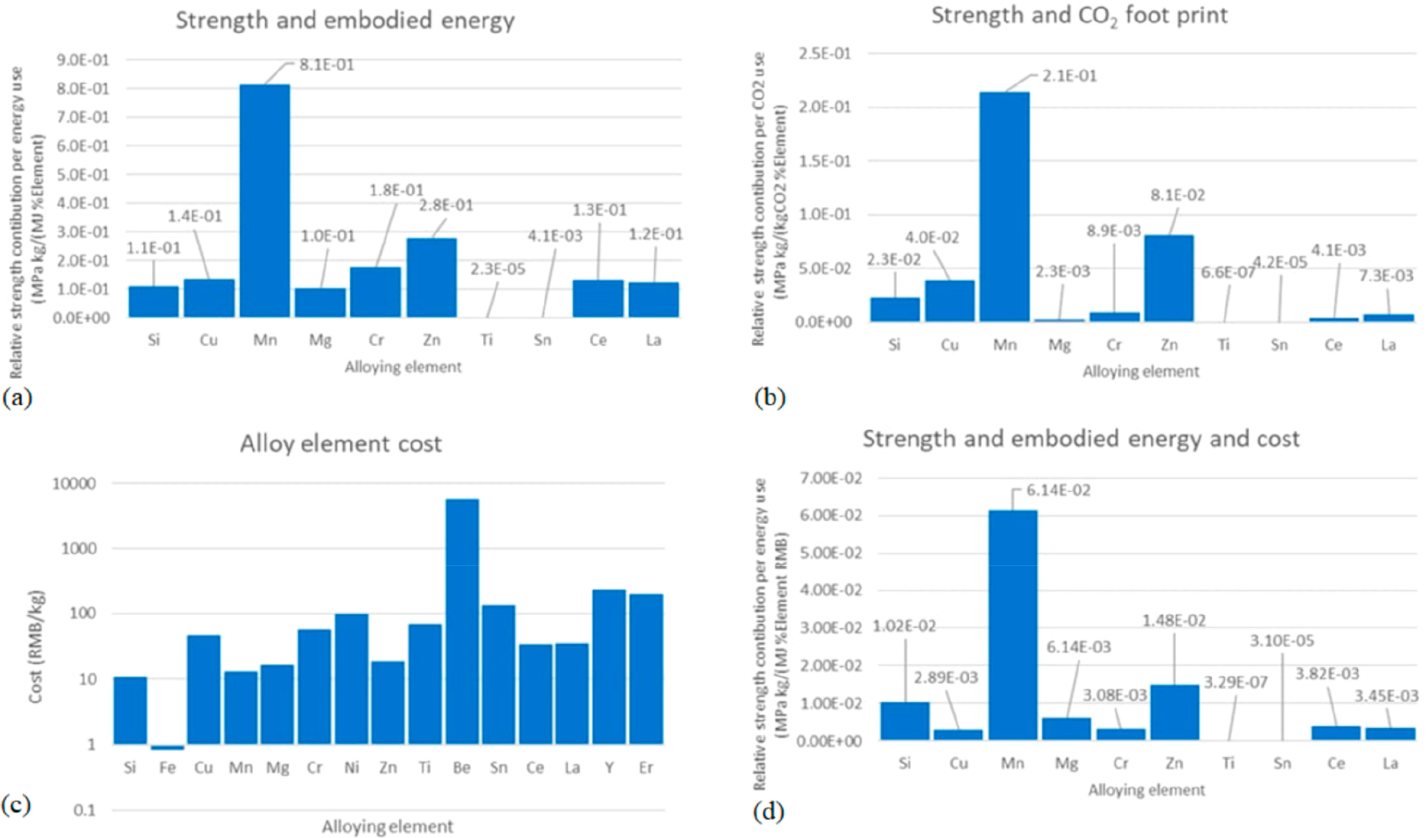
Detailed analysis of different important alloying elements in aluminum alloys by Jarfors et al.^600^ with respect to strength, cost and sustainability. (a) Contribution to alloy strength by alloying per unit of embodied energy. (b) Contribution to alloy strength by alloying per unit of CO_2_ footprint. (c) Alloying element costs using data from September 2019. (d) CO_2_ footprint and strength index with cost weighting. The figure has been reproduced with permission from ref (7) under an open access Creative Commons CC BY license. Copyright 2022, MDPI.

The important (triple) roles of the different main alloying elements in aluminum alloys (viz., mechanical properties, price, carbon footprint) were also discussed in detail by Jarfors et al.,^600^Figure 149. They discussed the effects of these elements on material strength, cost and sustainability, focusing on the sustainability–strength relation of alloying. They came to the conclusion that manganese would be the overall preferred doping element, followed by zinc and silicon. Other groups came to similar conclusions.^7,147,385,601,602^

Several groups conducted similar investigations for the case of alloying elements in steels, specifically regarding the loss of valuable elements due to insufficient scrap sorting and insufficient alloy-specific scrap management between potential scrap donor and scrap acceptor alloys.^165,167,384,431,603^

Specific attention had been in these studies placed on alloying elements such as manganese, chromium, nickel, and molybdenum, as they occur primarily in scrap from vehicles. It was reported that the recycling of end-of-life vehicles significantly affects the cycling of the alloying elements. Currently, the recycling of these steels is performed using electric arc furnace smelting. In this method, substantial losses of alloying elements occur due to vapor pressure and slag formation. Therefore alternative recycling schemes were discussed in the literature which would allow more efficient utilization of alloying elements.^160^

Cann et al.^115^ took a more general view at the problem of overalloying and inefficient material use, analyzing opportunities of alloy design as a sustainability improvement tool for seven different alloy systems. In their detailed work they considered both direct sustainability effects (those effects that help reduce energy consumption and greenhouse gas emissions) and also indirect sustainability effects (those effects that enable enhanced product sustainability through their properties^2^). Their recommendations include reducing quantities of required materials,^18,604^ enhanced longevity, environmentally conscious alloying element selection,^600^ property improvement, extension of lifetimes, materials enabling efficiency improvement, and improved material stability at high temperatures.^122^

### 9.2 The Science of “Dirty Alloys”

The somewhat flashy notion “dirty” alloys was recently coined in an overview paper about the secondary synthesis of aluminum alloys. The reason is that the most effective way to reduce the massive CO_2_ emission footprint of metallic materials is to produce them through secondary synthesis, i.e. from scrap.

This includes maximizing the scrap fraction in recycling as well as guiding the design and production of alloys from the highest possible scrap fractions, low-quality scrap, and scrap types with only a few matching target alloys they can serve in when recycled. A generation of more environmentally friendly metallic materials can be developed by strengthening the scientific foundations of this sector. This has the potential to make a significant impact on the metallurgical sciences as well as on creating a more sustainable society and circular economy. It takes a thorough understanding of the fundamental principles underlying sustainable metallurgy and recycled “green” alloys to translate such ambitions into successful, safe, and high-performance products that can meet the highest quality standards, creating a new field referred to as the “science of dirty alloys”.^7^

To be more specific, future research in advanced alloy design must be directed to better understand the metallurgical mechanisms behind the effects of tramp elements that enter through scrap and their influence on the materials’ properties. This understanding must then be the basis (a) to revisit, interrogate and probably change the impurity specification and bounds for existing alloys, so as to make them compositionally more forgiving and robust as acceptor alloys for scrap and (b) to generally develop new alloys which are upfront more composition tolerant and can cope with certain ranges of composition variation between charges (depending on the specific scrap composition used) without sacrificing performance.

Specific examples are the influence of scrap-related tramp elements on the phase equilibria and kinetics of phase transformation reactions and through that on their mechanical, functional and corrosion properties; formation of unwanted galvanic elements; precipitation-free zones at interfaces; change in recrystallization kinetics; formation of embrittling intermetallic phases; grain boundary decoration and the resulting interfacial embrittling effects; cast microstructures; and the processing parameters required to optimize the microstructure. Some of the most important research questions in that context, i.e. how to cope with alloys that have systematically higher impurity content that has intruded through scrap, were recently discussed in several publications and are summarized in Table 49.

**Table 49:** Research Topics Related to the Science of “Dirty Alloys”

Thermodynamic and kinetic databases for contaminated alloy systems and intermetallic phases with >4 components. When such phases become important in the context of contaminants from scrap, their compositions, kinetics and equilibria must be known, as well as composition adjustment opportunities to modify them.
Adjustment of processes to cope with the effects of contaminant elements: besides improved scrap sorting, techniques for advanced casting techniques including flow optimization and more rapid cooling are gaining momentum. For instance, high solidification rates in near-net-shape casting lead to lower through-thickness composition gradients, higher dispersion of contaminant-affected precipitates, reduced Scheil segregation profiles, and smaller grain and dendrite sizes, and thus generate greater homogeneity in the cast material overall.
Reduce the compositional variety, spread and number of alloy variants on the market, and even develop certain standards to govern which alloys should be used for certain parts so as to avoid the often-observed wide variation in alloy types used in the same product, which makes subsequent scrap separation a challenge.
Microstructure alloy tuning should generally be preferred over composition tuning when adjusting and developing materials for new products.
Microstructure tuning requires state-of-the-art processing equipment and both knowledge-based and machine-learning-based access to the underlying processing-structure–property relationships of alloys which contain impurities.
Developing more multipurpose and cross-over alloys. These could generally help to reduce the number of alloy variants on the market.
Improving scrap separation and establishing closed producer-customer scrap cycles.
Impurity trapping and segregation engineering toward capturing impurities.
Investigation of upper limits for tramp elements in alloys. How do impurities originating from the scrap behave in an alloy when higher impurity contents are permitted?

### 9.3 Alloy Variety and the Influence of Compositional Alloy Sensitivity on Sustainability

**Figure 150:**
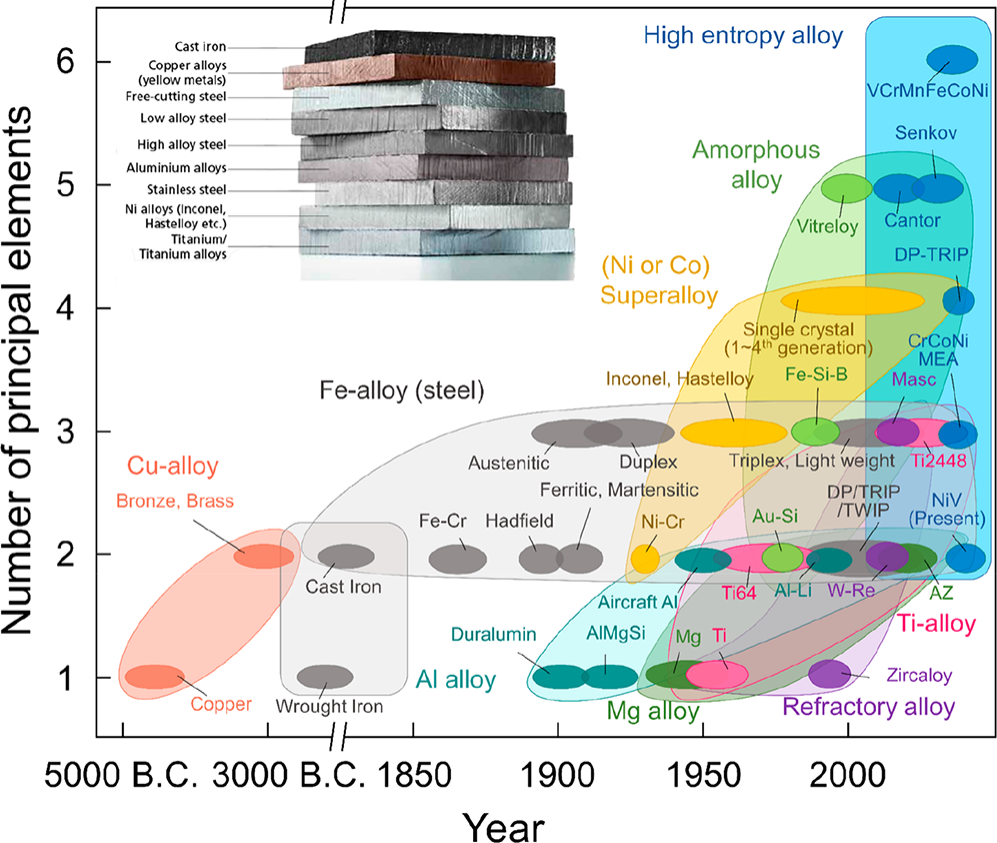
Increasing compositional complexity of alloys.^605^ The figure is reproduced from ref (605) with permission. Copyright 2019, Nature.

**Figure 151:**
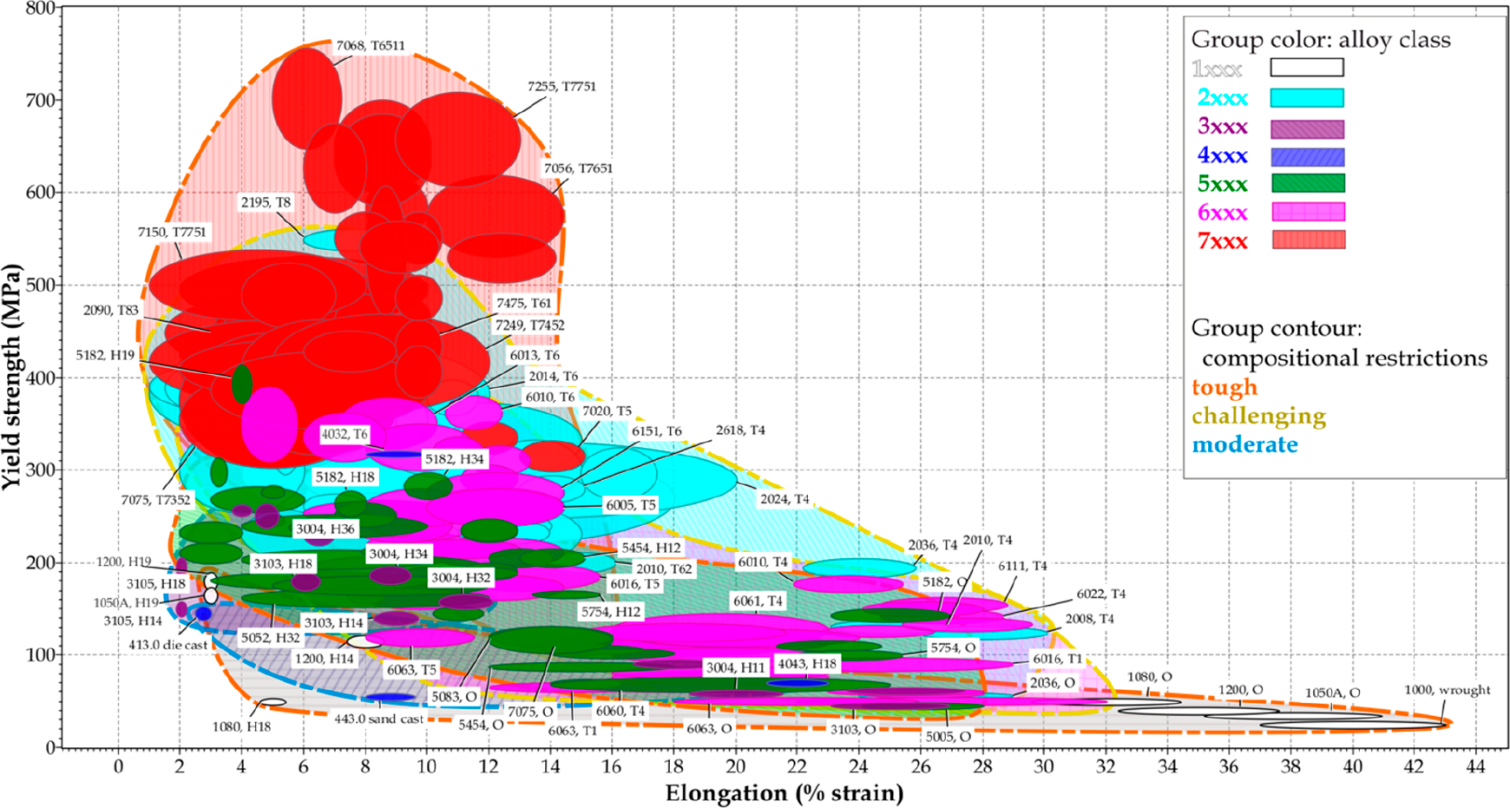
Compositional complexity of alloys shown in terms of an Ashby map of the yield strength values versus elongation of 1xxx–8xxx wrought aluminum alloys.^611^ The color lines indicate if the corresponding alloy families are more or less sensitive to the intrusion of scrap-related impurity elements (details about the most harmful impurity elements have been given by Raabe et al.^7^). The legend refers to the main wrought aluminum alloy groups with the following principal alloying elements: 1xxx, close to commercially pure aluminum; 2xxx, copper; 3xxx, manganese; 4xxx, silicon; 5xxx, magnesium; 6xxx, magnesium and silicon; 7xxx, zinc, copper and magnesium; 8xxx, other elements. The figure has been reproduced using data from Granta Design Limited. CES EduPack software. Granta Des Ltd. 2009 with permission from ref (7) under a 4.0 International Creative Commons license (CC BY 4.0). Copyright 2022, Elsevier.

Figures 150 and 151 illustrate the increasing chemical diversity and thus also the increasing chemical complexity of metallic materials.^605^ While metallic alloys originally consisted essentially of one main element and small amounts of dopants of other alloying elements, in the course of historical developments and the diverse demands on metallic materials, a large number of new alloys have emerged that contain significantly higher proportions of alloying elements.^162^ This circumstance, however, makes the recycling of alloys fundamentally more difficult. In addition, today’s high-performance alloys often have much smaller tolerances with regard to impurities, which also makes the mixing of scrapped alloys and thus recycling more difficult.

However, this circumstance creates interesting new questions for basic research. In addition to the above-mentioned demand to reduce the chemical complexity of alloys at the expense of an increase in microstructure complexity of metallic materials (with the same or a similar chemical composition), it should be investigated more closely in the future how tolerant the respective alloys may be to impurities in order to still be able to reliably fulfill certain requirements. A typical example of this is the copper, tin or zinc content in steels, introduced from scrap, which can lead to accumulation and formation of low-melting compounds which form at interfaces, leading to the phenomenon of liquid metal embrittlement.^10,76^

For aluminum alloys a typical example is the scrap-related inheritance of an enhanced iron content.^194,380,606^ Increased amounts of iron from the admixed scrap can lead to undesirable intermetallic phases and thus to embrittlement and increased corrosion. Aluminum alloys are usually more sensitive to even minor composition variations than many steels because they have usually low solubility so that many impurity elements lead to intermetallic phases, which can influence ductility, fracture toughness, fatigue, forming limits, surface properties, corrosion, age-hardening capability and precipitation-free zones. The associated effects can be positive (e.g., by controlling recrystallization during homogenization or by refining grain size during casting) or negative (e.g., for fracture properties if the particle size, shape and distribution are not well controlled). Well known in this respect is Fe, which can cause morphologically unfavorable intermetallic phases to form in many alloys.

However, the nature and properties of the phases formed are highly dependent on processing and constitution, and there are many possibilities for optimization. In addition to forming intermetallics, impurity elements can also significantly modify existing intermetallics (through changes in intermetallic type or shape). A well-known example is the addition of manganese or chromium to AlFeSi-containing intermetallics.^7,118,429,607−610^ These transition metal dopants can change the shape of the intermetallic phases in aluminum alloys, to render them less harmful or even beneficial.

Another effect of impurities can be segregation at interfaces, weakening mechanical properties or corrosion properties (e.g., copper at grain boundaries). In addition to the formation of intermetallic phases, elements in the solid solution can also have effects, e.g. on the precipitation kinetics. Knowing more about how impurity elements might be used in such areas would be a useful aim in the science of “dirty” alloys. Another important impact of impurity elements is on the precipitation process itself, either directly through effects on phase selection and the trajectory of metastable and stable phases that form or indirectly through interactions with vacancies that subsequently affect precipitation.

It is also important in such investigations that not only the absolute impurity contents and their effect on the material properties are investigated, but also the ability to influence the dispersion of undesirable second phases, for example through faster cooling during solidification or through thermomechanical forming, during which such precipitates could possibly be converted into harmless precipitates.

Furthermore, an important research question could be to classify alloys according to whether they are easy to recycle or not and to evaluate them accordingly in the market. In doing so, two aspects have to be considered: on the one hand, the role of such alloys as acceptors of scrap and, on the other hand, the role as donors for new materials.

In principle all alloys must become more tolerant for high and variable impurity content. The underlying key challenge is the intrusion and accumulation of impurity elements in alloys, in terms of both the number of different elements and their concentrations. Here two main tasks emerge. One is to develop engineering alloys which are more scrap compatible, i.e. more tolerant of tramp elements that intrude from scrap. The other task is to reduce both the number of alloys and their chemical complexity and, wherever possible, to achieve consistent properties by replacing composition tuning by microstructure tuning.

Furthermore, it should be systematically investigated in the future how much certain material properties actually depend on certain impurity levels. In some of the existing material specifications regarding the limitation of maximum impurity contents, the specified limits could possibly be obsolete in the meantime and thus be shiftable to higher values, because possibly newer process conditions, faster cooling conditions and better thermomechanical processes allow higher impurity contents without the material properties suffering. This means that a systematic investigation of the dependence of the material properties on certain impurity levels should be carried out in order to allow higher scrap contents in the synthesis of metallic alloys and in recycling.

### 9.4 Cross-over Alloys and Unialloys: Unified Alloy Concepts for Stainless Steels and Aluminum Alloys

An interesting alternative to the mutual “alloy poisoning” risk when different alloys are mixed together as scrap into new materials, is the introduction of so-called “cross over” or “unialloy” materials.^7,414^ This alloy design principle represents a disruptive approach, where broadband materials which cross established alloy specification boarders are designed to replace alloys that have generally been developed for performance in a rather narrow range of applications for a very specific customer purpose. The key idea behind this novel approach is to replace the currently problematic multimaterial mix in products by a smaller set of alloys, with the aim to improve the recyclability of the product.

The concept has been recently developed further for the case of aluminum alloys, but some of the existing chromium-nickel stainless steel grades had been developed in the past along similar lines, to reduce alloy diversity and thereby improve recyclability through better mutual scrap compatibility.

In the specific context of stainless steels, there is also an interesting conflict of objectives: stainless steels have very high contents of chromium (10–20 wt %), nickel (0–12 wt %) and often molybdenum (0–4 wt %). For this reason, there has long been an economic as well as a sustainability incentive to replace particularly the CO_2_-intensive and expensive elements such as nickel with cheaper and also less CO_2_-polluting elements such as manganese. For example, the nickel content of austenitic stainless steels alone accounts for about 70–80% of the price of the material. If one only considers the primary synthesis of such steels from ores, this would be a real development step. On the other hand, however, such stainless steels are now produced from very high proportions of scrap, with a global average of up to 50%, and regionally even up to 90% scrap input. This scrap cycle, which is often very well developed for established alloy classes, would, however, be interrupted and contaminated by the introduction of new alloying elements. This would reduce the value of these scrap types (at present, between 1000 and 2500 Euros are paid per tonne of stainless steel scrap) and would lead to the accumulation of new and undesirable contamination. This means that all substitution considerations must always take into account whether an alloying element is extracted from primary minerals or from scrap. It could also turn out that alloying elements that have a high CO_2_ footprint when synthesized, once they have reached the material in a reduced state, prove to be more sustainable on the long run, in case the corresponding scrap can then be better recycled.

Another interesting example comes recently from aluminum alloy design. In their papers about cross-over aluminum alloys, the authors have not only tried to combine different alloy grades but also achieved improved properties relative to some of the established commercial aluminum alloys. More specifically they developed cross-over alloys between the established alloy classes AlMg/AlCuMg (5xxx/2xxx) and AlMg/AlZnMg(Cu) (5xxx/7xxx).^193,414^ They showed that the new alloys have excellent formability combined with high age-hardening potential, which is attractive for a wide range of industrial sheet forming applications. Since these new alloys have magnesium as the main constituent but—unlike the commercial AlMg alloys—are age-hardenable, they do not fit into the current alloying scheme and thus represent a novel class of alloys with a high recycling potential. From these initial results the question arises which cross-over alloys are the most promising materials for combining beneficial mechanisms across established alloy families. More specific and detailed aspects of this concept have been discussed in several recent overview papers.^7,115,414^

### 9.5 Medium-Manganese Steels as an Example of a Compositionally Lean and Multifeature Alloy System

Steels with medium-manganese content constitute a broad class of versatile yet compositionally lean alloys. Their manganese content lies between that of the low-manganese first-generation steels with less than 3 wt % manganese and that of the second-generation advanced high-strength steels with more than 13 wt % manganese.^408,612−616^ These steels are attractive due to their excellent mechanical properties paired with moderate alloy content. They could play an important role for the growing demand for sustainable, compositionally lean, strong and ductile steels, especially for body-in-white parts with low weight and high damage tolerance for vehicles.^166,170,404^

Medium-manganese steels differ significantly from other steels with regard to composition, microstructure and their wide spectrum of deformation mechanisms. Four aspects are particularly relevant, namely, their (a) chemical composition and interphase solute partitioning; (b) microstructure variety in terms of phases and microstructure constituents (e.g., austenite, ferrite, martensite, bainite, pearlite and carbides), including their size, dispersion, morphology, distribution and percolation; (c) microscopic solute enrichment/segregation to the multiple defects with several opportunities for segregation functionalization; and (d) austenite mechanical stability/metastability design and the associated dislocation and athermal deformation mechanisms and defect patterning phenomena.

The interplay of these mechanisms and the possibility of compositional fine-tuning of these features, at low alloy costs, allow us to trigger practically almost all known strengthening and strain hardening mechanisms in one single material. These are solid solution strengthening by both interstitials and substitutional elements; interface strengthening; precipitation strengthening; transformation-induced plasticity (TRIP); twinning-induced plasticity (TWIP); dislocation strengthening; dynamic strain aging; and multiphase composite strengthening. This large variety of micromechanical strengthening mechanisms, all assembled within a well adjustable chemical composition and processing range, accessible also to established industry-scale processing routes, opens access to the design of materials with excellent strength-ductility synergy. Here, particularly the product of tensile strength and total elongation up to 80 GPa% at relatively lean alloy content are remarkable features.

**Figure 152:**
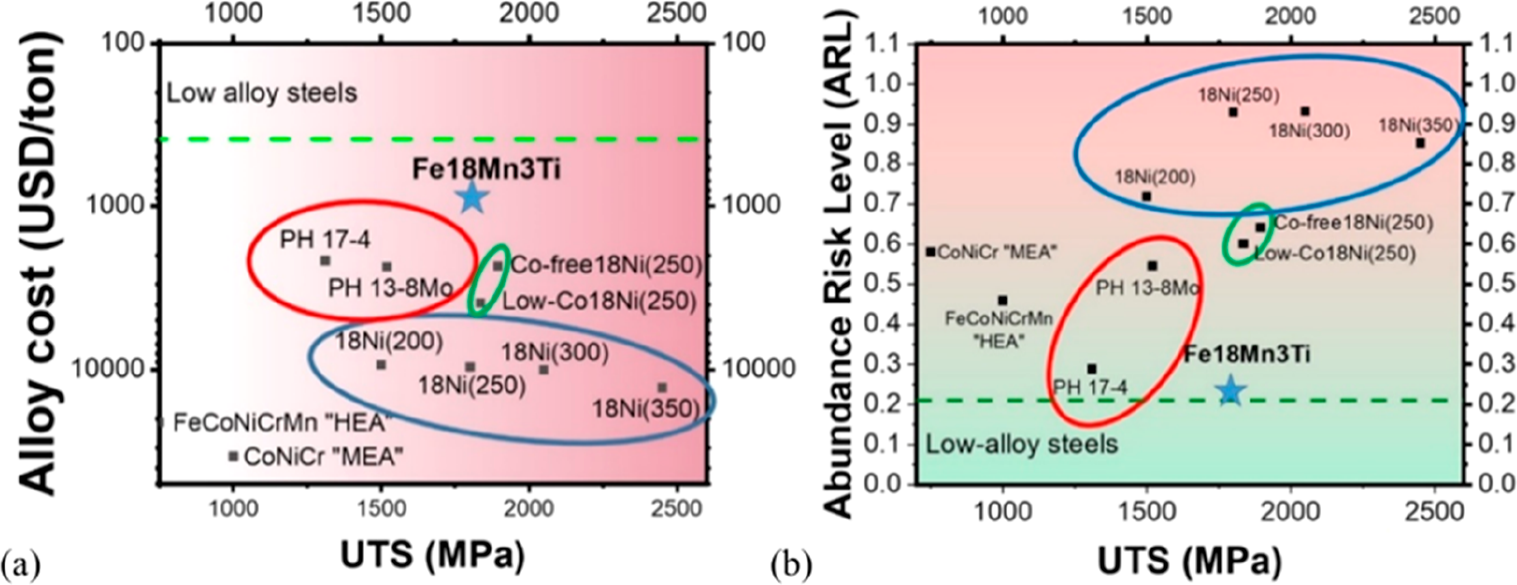
(a) Comparison of alloy costs and (b) the abundance risk level (ARL) of various precipitation hardened ultrahigh strength steels.^617^ MEA, medium entropy alloy; PH, very tough precipitation-hardening stainless steels with varying contents of chromium, nickel, molybdenum, etc. where the specific alloy values have been given in brackets in the figure. UTS, ultimate tensile strength. Figure is reproduced from ref (617) with permission. Copyright 2022, Nature.

A related example of a lean high-performance yet sustainable maraging steel was recently published by Kwiatkowski et al.^617^ They showed how maraging steels can be designed, without using environmentally harmful elements, Figure 152. They developed a Fe18Mn3Ti (wt %) maraging material, susceptible to homogeneous phase decomposition, assisted by segregation and composed of the three abundant transition metals. This alloy composition was designed to be unstable against compositional fluctuations at the intended aging temperature, around 450 °C. Three wt % titanium was added to the alloy, to enable the transformation of austenite to α-martensite during quenching and cold rolling, preventing a high fraction of retained austenite and ϵ martensite with hexagonal lattice structure, and stabilize the second phase α-Mn precipitates.

## 10 Sustainable Combustion of Metals and Alloys: A Nexus for Clean Energy and Sustainable Hydrogen Production

**Figure 153:**
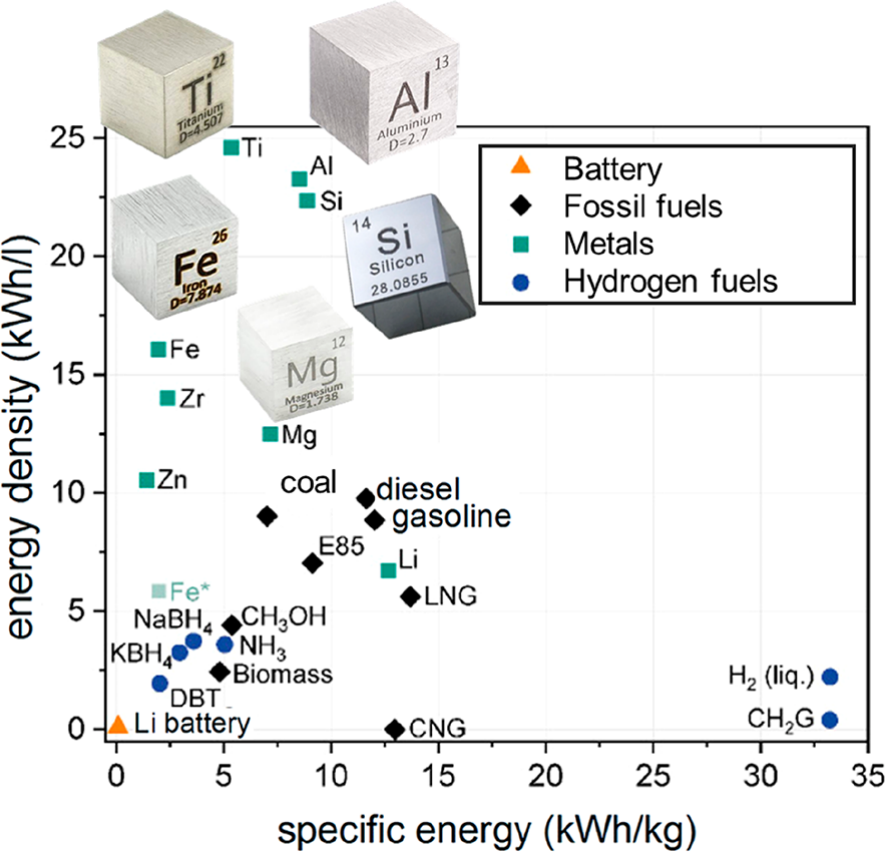
Material-related volumetric and gravimetric energy storage density for some energy carriers:^618^ LNG, liquid natural gas; CNG, compressed natural gas; E85, ethanol fuel; DBT, dibenzyl toluene which serves as a base material serving in liquid organic hydrogen carrier media (LOHC). The figure is in modified form reproduced from ref (618) with permission. Copyright 2017, Royal Society of Chemistry.

Beyond the topics on metallurgical sustainability discussed so far, there is another very exciting development in this context that brings together the topics of sustainable metals, regenerative energy conversion and environmentally friendly hydrogen production. This is the topic of using metals as carbon-free energy carriers, Figure 153.^618,619^

Energy is thereby obtained by burning metal powders or sprays with air or by reacting with a humid atmosphere to form H_2_. With energy from non-fossil sources, the resulting metal oxides are regenerated in an emission-free energy storage cycle by thermo- or electrochemical reduction processes.^112,619,620^ The energy stored in the metal can be made available through oxidation in the course of combustion, for example in power plants or corresponding combustion engines.

So far, this topic has received comparatively little attention in materials science and metallurgy, but it has already been dealt with extensively in the field of combustion technologies.

The thermodynamic principle of this approach is obvious: the thermodynamic energy stored in reduced metals, which can be released during oxidation, is much higher than that of almost all other fuels, an advantage that has already been used in rocket engines, for example. The grand scientific challenge behind this approach is, on the one hand, efficient combustion of the metals, which are usually provided in the form of powders for this purpose, and, on the other hand, reduction of the powder particles, which are oxidized after combustion, back to metals using sustainable reduction pathways in order to feed them back into the combustion process.

The last step in particular presents a major challenge, as the powder distribution of the corresponding oxides cannot usually be converted back into an equivalent powder particle distribution in the case of combustion by means of a reduction treatment, for example in a fluidized-bed reduction process. Another aspect that is currently being considered in research is how completely and how precisely the combustion of the corresponding metals takes place and into exactly which oxidation stages it proceeds. Detailed microstructure- and alloy-oriented research on these topics has also only just begun on the corresponding materials.^113^

Iron, aluminum and silicon in particular are considered to have high potential as metal fuels due to their huge abundance and safe handling. These and other (semi-)metals have a very high energy storage density (iron, 16 kWh per liter; aluminum, 24.5 kWh per liter), exceeding those of most other energy carriers, Figure 153.

The concept is particularly attractive for stationary power generation, as existing infrastructures can be used. Particularly iron is studied intensely as a promising energy carrier, since it has similar combustion properties to coal and few nanoparticles are produced during combustion. According to current estimates, an efficiency of the energy storage cycle of up to 40% can be achieved.

## 11 Conclusions, Opportunities and Future Directions for Basic Research on the Direct Sustainability of Metals

**Figure 154:**
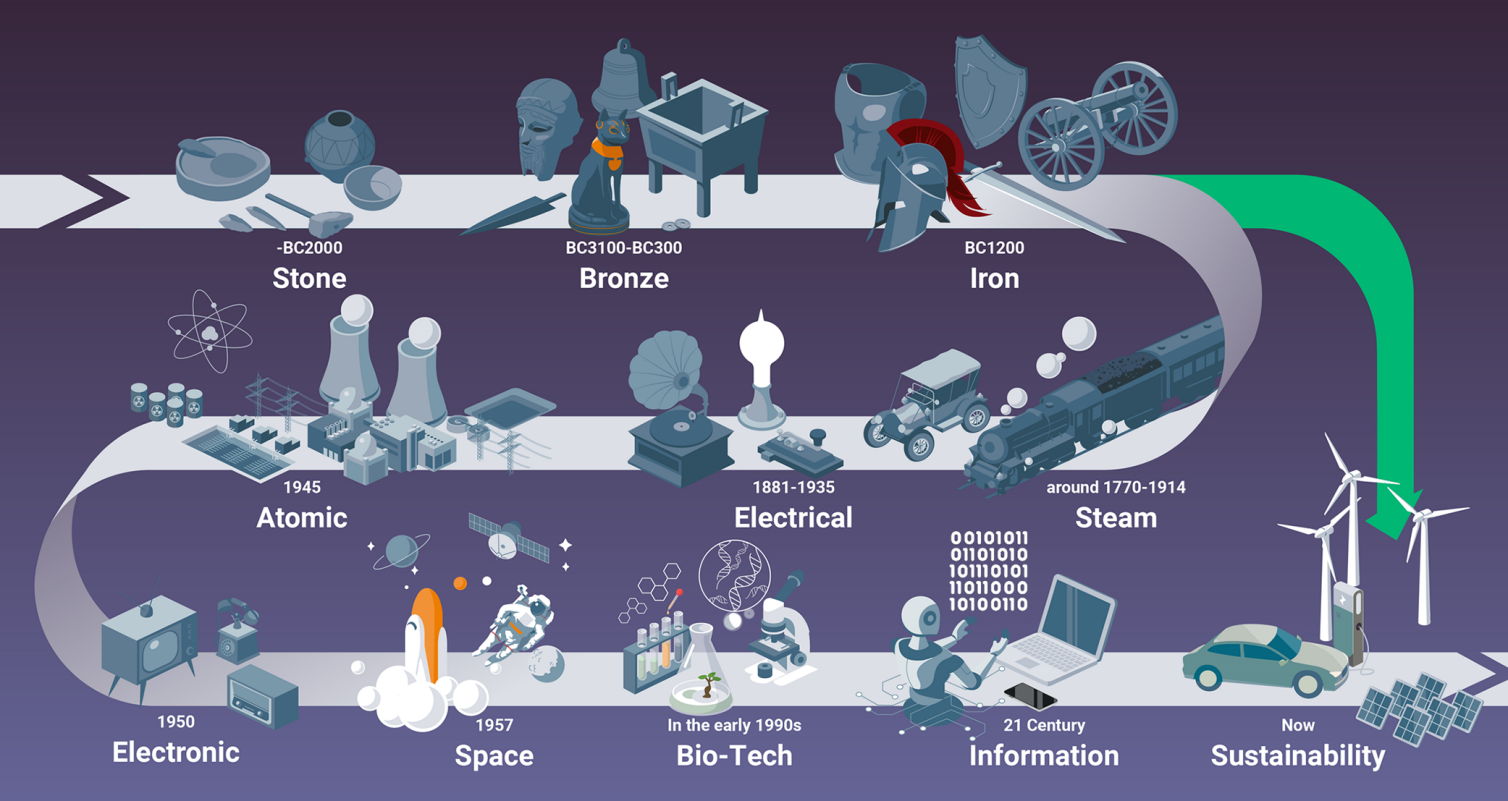
The dawning age of sustainability and a circular economy and the roles metals play in it: After more than 5000 years the metallurgical sector must be decarburized, electrified with renewable energy and turned toward higher scrap use. This is nothing less than a revolution which must be accomplished in less than a few decades.

The synthesis and processing of metallic materials is the largest single source of greenhouse gas emissions in the world. This article has attempted to give an overview of measures in this area from the perspective of basic research, which would allow a fast and massive reduction of these CO_2_ emissions. The paper has collected, presented, filtered and discussed which research efforts would be helpful for achieving and accelerating this once-in-a generation challenge, namely, after more than 5000 years, to decarburize one of the most eminent backbone sectors of our civilization, in less than a few decades, Figure 154. Also, other important sustainability-related aspects of the metallurgical sector have been discussed, such as reduction in energy consumption, water contamination, and waste production, to span the range of sustainability topics also beyond greenhouse gas emissions.

The article is not concerned with the many indirect sustainability measures that metals help realize through their properties, be it through weight reduction, through improved corrosion protection or through functional properties like conductivity, magnetism or thermoelectric features. These aspects are not in the scope of this paper. Likewise, excellent options for improved sustainability in this field are of course the increase of material longevity (that is through better corrosion and fatigue resistance as well as through lower tribological losses) and the reuse of material including also microstructural rejuvenization and self-repair methods. As some of these measures (except corrosion protection) can potentially only cover a moderate fraction of the huge metal market and have been recently reviewed in several excellent works, they have found less room in this paper.

The principle of sustainability of metal production does not only refer to the emission of greenhouse gases and energy consumption, but it also has to take into account numerous other categories, such as the use of land, the contamination of the environment with toxic residues, the consumption and contamination of water, the living and working conditions of the population, etc. However, this article is concerned mainly with the first two aspects, and here in particular with measures and progress that can be leveraged through basic metallurgical research. Likewise, it is clear that besides the many fundamental materials science questions addressed in this paper, multiple boundary conditions to guide improved sustainability of the metal sector are today and in the future also set by costs, engineering, social and political aspects, but these aspects are also beyond the scope of this article.

Considering this focus of the present article, a few main observations need to be generally considered as boundary conditions for basic research on the direct sustainability of metals:

1. Efficiency of metallurgical processes: Further increase in process efficiency alone is insufficient to meet the sustainability challenge. Each and every process step in the metallurgical sector is and must also in the future be permanently revisited and scrutinized with regard to higher efficiency. Yet, the total market growth is so immense that efficiency improvement which is in many parts of metallurgical production already quite good or even high, for obvious cost reasons, does alone not solve the problem of the massive greenhouse gas emissions from this industry. Basic research for further efficiency and sustainability improvement could particularly come from removing fossil fuels from all pyrometallurgical and heat treatment steps, electrification of processes (under consideration of the quite different efficiencies of ladles, converters, melting furnaces and heat treatment ovens, operated with different electrification principles) and the consequent application of artificial intelligence methods such as particularly of reinforced deep learning methods to processes and process chains, a field far underdeveloped and untapped at the moment.
2. Recycling versus primary synthesis: Replacing primary synthesis via mineral reduction by the melting of well sorted scrap is the fastest and most efficient technique to reduce greenhouse gas emissions in this field, and it has also by far the highest technology readiness level compared to many new synthesis methods. Recycling of scrap is already a well-established approach for a number of products, particularly those that use a small number of alloys and have a high collection rate, such as certain packing materials or some automotive and construction parts. However, it must be understood that the enormous market growth in the field of metallurgical products will even in best-case scenarios fall short by about 1/3 of the total market demand, simply due to the lack of sufficient amounts of well sorted scrap. This means that also in the future the economy cannot be 100% circular, neither from a market perspective (insufficient scrap, infrastructure longevity and the associated delay in metal return rates, too much low-quality and contaminated scrap, etc.) nor from an entropy perspective (loss in production, use, corrosion, etc.). This means that in the best case in average (for the large mass-produced metal groups) we can get back in average about 2/3^rd^ of the metal from recycling and the rest must be produced from primary synthesis (using new minerals) and ternary synthesis (re-mining and use of dumped waste) at least until 2060, and this is still mostly done with current primary synthesis technologies which emit too much CO_2_. This simple projection again underlines that basic research activities must be particularly devoted to the improvement of primary synthesis with respect to lowering greenhouse gas emissions.
3. Delay factor in secondary synthesis: An important aspect in assessing the future availability of metallic scrap for the recycling industry is the highly graduated time delay in the return of scrap for secondary synthesis. While, for example, packaging material, e.g. for the production of cans, can already be on the shelf again as a new product a few weeks after scrapping, metal from buildings and comparable infrastructure elements sometimes only returns to the market after 100 years. This is particularly the case with large-volume products and must be considered for the further development of the secondary synthesis of metals.
4. Nanoscrap and element dispersion in products: the recycling of metals from many modern products has become significantly more difficult by the extremely high number of elements used in a single product and their very high system integration and the associated dispersion, down to the nanometer range. This is particularly the case for microelectronics and catalysts. This problem particularly concerns the recovery of very expensive, ecologically questionable, toxic as well as strategically important elements. In this sector, the further development of suitable hydrometallurgical, electrometallurgical and biometallurgical processes is therefore of great importance.
5. Quantification of measures: Measures to remove the fossil element from the metallurgical sector and thereby massively reduce greenhouse gas emissions must not be decided on the basis of “gut feeling” or marketing criteria, but must be subjected to scientifically validated life cycle assessments. Otherwise, some measures might lead to the introduction of processes that may look cleaner in the short term but in macroeconomic terms lead to higher CO_2_ emissions at mid- and long-term. The details of life cycle assessments and similar evaluation criteria must be done by experts and are beyond the scope of this paper.
6. Careful with biomass: any use of biomass in metallurgical processes—especially in those used for the production of the mass-produced metals steel and aluminum—should be avoided as far as possible. The reason for this is simply that, first, CO_2_ will still be emitted in the end and, second, there would be an extremely unfavorable competitive situation between food production and biomass production for industrial processes. Third, biomass use in this sector could lead to catastrophic market effects globally, for example to deforestation to fuel metallurgical processes in industrial regions or to serve as a reducing agent. These three effects should be avoided at all costs, and the use of “new” biomass for the “apparent” improvement of the life cycle assessment of metallurgical processes should therefore be avoided wherever possible. In this context, the huge absolute quantities of metals produced in the world of about two billion tonnes per year must be taken into account, which—if biomass were used for even a fraction of their production—would lead to the destruction of gigantic quantities of plants.
7. Rebound effect associated with sustainable technologies: the introduction of sustainable technologies (such as wind and solar power as well as electrified vehicles) creates a massive rebound effect in the metallurgical sector, that will at the short-term lead to the increase of CO_2_ emissions. The specific consumption of minerals, metals and metallurgical downstream products for realizing the massive change of our energy supply, industry operations, transport, digitalization, etc. to a more sustainable system requires the production of so many new machines which use a much larger spectrum and much more intense system integration than any product generation before them that their production will boost and not reduce the consumption of metals and the resources required for that, at the technology level that we currently globally have in metal production. This is particularly true for many critical and expensive metals such as copper and rare earth elements, but also for some very CO_2_ intense metals such as steel, nickel and cobalt. This means that the emissions of greenhouse gas and the consumption of energy will in the next 2 decades grow and not shrink, if the projected technology shift is about to be realized without disruptive changes in synthesis, production and use of metals.
8. Rebound effect associated with mineral resource consumption: Similar to the rebound effect described above in connection with the introduction of sustainable technologies, there is also a general rebound effect in the global supply of raw materials: an important constraint when considering the sustainability of metals is that many of the minerals required will not be available in the future in the same aggregation state, quantity and quality nor at comparable prices as they are today. This does not generally mean that the metals required for shaping the future will no longer be available in the Earth’s crust, but it does mean that the effort required to make them accessible for industrial use will increase, in some cases drastically, as their accessibility, dispersion and mineral dilution increasingly decrease. This in turn implies that the CO_2_ emissions associated with producing metals will in the future be much higher than they are today when using the same technology, simply due to the insufficient accessibility of the underlying mineral resources. Finally, it also means that we must consider less concentrated and more highly contaminated low-price minerals in the primary synthesis sector and develop processes that can produce high-quality alloys from them.
9. Rebound effect associated with non-fossil fuels and reductants: The drastic reduction of greenhouse gas emissions in primary metallurgical synthesis can only be achieved by a massive increase in the degree of electrification and the use of non-fossil reduction agents such as hydrogen, ammonia and other non-fossil power-to-reductant products made with sustainable energy. The provision of the required sustainable electrical energy by the corresponding aggregates (wind power plants, solar power plants, hydropower, etc.), produced with the currently available conventional and very greenhouse gas-intensive metallurgical production methods, will create a considerable rebound effect which, integrally considered, will lead to an increase in greenhouse gas emissions in the short term and not to their reduction. Furthermore, as already mentioned above, the expansion of renewable energies required for covering, for example, the gigantically growing future demand for hydrogen through electrolysis based on renewable energies alone will take decades and will still only be able to cover part of the actual industrial demand. Both the installation of all these renewable energy sources and the implementation of the corresponding electrolyzers with the necessary catalysts will further fuel this enormous rebound effect and, in the medium term, initially lead to an increase in CO_2_ emissions if only the technologies available today are used for the corresponding metal production.
10. The science of scrap-contaminated “dirty” alloys and “dirty” feedstock: The numerous new production processes for more sustainable metallic products in the future through (sustainable) primary synthesis, recycling and re-miming by use of other variants of minerals, additives and reducing agents used for this purpose will lead overall to metallic products with a different chemical impurity profile compared to the materials currently on the market. This means that in the case of the expected fundamental change in many synthesis and downstream production processes as well as the use of other raw material and reductant compositions, the effect of a wide range of characteristic spectra and impurity elements as well as gas contents in the synthesized materials must be investigated with regard to their properties. This circumstance was also coined with the term “the science of dirty alloys”. It can in part also be considered an advantage, when considering for instance the use of hydrogen or ammonia as non-fossil reductants and furnace fuels in the steel industry, because these processes require not the same purity of the reductants like for instance fuel cells, so that low-price and less-pure synthesis pathways for such raw materials offer attractive market opportunities in this sector. This means that a future sustainable metallurgical sector requires deep understanding of all impurity and contaminant effects in the entire spectrum of alloys, scrap, reductants, by-products, waste products and minerals.
11. Big numbers, small numbers and leverage: in order to reduce emissions in metallurgy fast enough, we need to keep focus on the absolute numbers: we produce every year about 2 billion tonnes of metals, use for that 3.2 billion tonnes of minerals, create about 20 billion tonnes of moved soil, waste, and by-products, create by that nearly 40% of all industrial greenhouse gas emissions, cause about 10% of the global energy consumption and yield recycling rates between 1%–95% (with an average of only 1/3 for the mass products iron and aluminum). Steel alone stands by far for the largest fractions, with about 33% of all industrial greenhouse gas emissions and 3 billion tonnes of iron ores mined every year. This means that quantitatively tangible and rapid progress in reducing greenhouse gas emissions in the metallurgical sector can only be achieved through rapid action in the largest emitters, i.e. primarily in the steel and aluminum industries. This is where the big numbers are, and even very small advances in sustainable synthesis and production have an enormous leverage in the total amounts emitted. To emphasize this, it must therefore be clearly understood that even the smallest possible improvements in sustainability in synthesis should be accelerated through appropriate research, as only then can the really large emission amounts be addressed through the large leverage behind them. Nevertheless, the sustainability of all other metals should also be improved in quantitatively smaller areas, but the main starting point for reducing the increase in global warming lies in the very large numbers and not in the small quantities.
12. Transition technology scenarios: the large metallurgical volumes in the steel and aluminum industry in particular are traditionally linked to very large aggregates and factories, associated with huge investments. These often cannot be replaced in the very short-term without completely jeopardizing the continued existence of the corresponding industries. This means that research must also look specifically at transition technologies that can make maximum use of already existing and available aggregates and processes, especially in the area of primary synthesis and heat treatment. This specifically includes, for example, the introduction of electrification of existing aggregates as well as the use of non-fossil fuels and reducing agents in otherwise existing and operative conventional reduction aggregates. This amounts sometimes only to the refurbishment of existing technologies at moderate costs, compared to the design and installation of entirely new technologies. In the long term, of course, completely new aggregates and more disruptive techniques must be developed, but in a transitional period these aspects must be taken into account in order to be able to filter out technologies that can be implemented from a basic research perspective but which are not commercially viable at short term.
13. Conservative thinking versus disruptive breakthroughs: Especially with regard to the long-term conversion of the entire metallurgical production chains to sustainable technologies, the scenarios currently being discussed are often too conservative and not sufficiently disruptive. The fundamental shift away from a carbon-based industry requires a complete rethinking of chemical processes and metallurgical fundamentals in many areas, which in turn could enable new processes and catalyze the start of new markets, potentially establishing new types of customer demand that have not yet been considered in any textbook or analysis to date. This should come more to the fore in future research, instead of remaining stuck in the above-mentioned transitional technologies. This means that transitional technologies should not be confused with truly new and disruptive methods, but both aspects need to be advanced in basic research, pilots plants and implementation; otherwise, there will be a strong acceleration of global warming. Currently there is too much projection of existing and conservative technologies and insufficient room for disruptive breakthroughs considered in current research and industry projects.
14. Metal- and product-specific solutions: Basic research and appropriate technological solutions to improve the sustainability of the different types of metals and alloys are very different and highly dependent on the metals and the products made from them (when it comes to recycling). For example, while in the case of the steel industry the highest focus is on eliminating carbon from the entire synthesis and production chain, whether through electrolysis, hydrogen-based reduction or plasmametallurgy, in the case of recycling mobile phones and computer chips it is on improved pyrolysis and hydrometallurgical processes that are able to recover a variety of elements in a staggered process with minimal losses and low toxic waste products. Other issues, such as improved scrap sorting or smarter and more efficient heat treatments, require artificial intelligence methods. Yet other topics, such as the limited availability of sustainably recoverable copper minerals, highly CO_2_ polluted metals such as nickel or cobalt, or rare earths, require research into the discovery and development of suitable substitute materials to reduce or even eliminate the use of these elements from current products and replace them with materials of less concern. The take-away here is that serious sustainability research decomposes into a large array of subtopics which have in part even little in common and require highly expert researchers to find viable engineering solutions for the different alloys, processes and product types.
15. Greenhouse gas emissions first: The most important and urgent parameters to address by basic research on sustainable metals should be measures that can help reduce the massive greenhouse gas emissions and the use of energy along the entire process chain. These two challenges must be the major attack points, thus constituting the most urgent research questions in sustainable metallurgy.
16. Use every atom: The entire field of sustainable metallurgy must be thought of in such a way that, in principle, every atom can be fed back into the material cycle, minus the entropy-related losses. This inevitably means that not only the highest efficiency in the extraction of the metals themselves must be the goal, but also the entire refeeding of all auxiliary materials as well as intermediate and residual products that arise during synthesis and production must be rendered back into the material cycle. This means that every “used” atom must be fed back into the recycling stream.
17. Multi-element and high quality recycling: the enormous depth of integration of metals in modern products in terms of size scale and number of chemical elements used is one of the most difficult problems for high-quality recycling. This problem can only be solved by complex process sequences involving pyrometallurgical processes, pyrolysis and hydrometallurgical processes, whereby electrochemical and biological processes will also be used. These process sequences and process chains must be very precisely matched to the corresponding melting points, vapor pressures and oxidation tendencies of all the elements involved in order to remove them step by step from the nanoscale material composites.
18. Material- and recycling-oriented product design: the entire design of complex modern industrial and consumer products must be radically and rapidly changed so that when they are discarded and dismantled, at least a rough presorting can take place, for example, according to electronic components, batteries, magnetic components, etc. This means, for example, quite simply that a battery must always be removable from the product, that components containing magnets must be removed and sorted separately, and that all important electronic elements, for example in modern cars, must be housed in separate removable boxes. Only if there is a closer and above all recycling-oriented connection between product design and material design can the decisive coarse presorting of materials take place, which would improve the subsequent recycling quite considerably in terms of quality at reduced costs and lower environmental impact. This aspect has hardly been given sufficient attention in research and has been neglected in industrial implementation so far.

These basic boundary conditions make clear that metallurgical sustainability does not constitute a homogeneous research field and will not necessarily become one in the future, but rather it breaks down into a large spectrum of quite different disciplines and branches. However, it also becomes clear that this wide theme offers a lot of room for disruptive discoveries especially in the overlap between the established disciplines. This opens up many novel opportunities for fundamental research, to cope with challenges that may only occur once in a generation. This is not blue sky research, but any new finding that helps to make metals more sustainable can have a huge and immediate impact on our lives and the future of the planet. The huge manufacturing quantities involved mean that even small progress can unfold high leverage. This does not mean that the research content should be too closely oriented to the projection of existing technologies, as a merely evolutionary research approach will not solve the big challenges ahead, but instead disruptive technology changes should be pursued. This new exciting field of research always comes with a certain promise of fulfillment, that in case of success, a huge benefit for our society can be achieved, which can hardly be overestimated.

When keeping these considerations and boundary conditions in mind, a few specific directions for basic research, remaining challenges and future directions in direct sustainability, fossil-free synthesis and contaminant-tolerant alloy design can be filtered from this article and from the literature quoted herein. These tasks include the follow possible basic materials science aspects, yet many more interesting and interdisciplinary topics are likely to emerge:

- Replacement of primary synthesis (from minerals) wherever possible by secondary (from scrap) and tertiary (from re-mining of waste) synthesis.
- Reduction of the current huge range of chemically different alloys to improve the mutual recycling of scrap in new alloys, in terms of donor and acceptor quality.
- Alloy design with chemically simple composition based on microstructure optimization rather than chemical optimization.
- Replacing less sustainable materials and alloying elements in metallic alloys with those with better sustainability, i.e. substitution of nonsustainable metallic alloys and alloy ingredients by more sustainable ones.
- Development of smarter, alloy- and alloy-group-specific, machine-learning-informed and automated scrap sorting.
- Increase in the closed-loop recycling fraction, i.e. of in-production and sort-specific scrap collection that is directly returned into production.
- Better coordination of product design and material design for material-specific and alloy-group-specific disassembly of complex products.
- Multi-element recovery of metals from mixed minerals and from intensely mixed (electronic) scrap.
- Investigation of the influence of new types of impurity and tramp element spectra from all types of feedstock, mineral-specific gangue elements, scrap-specific impurities, reductant and fuel contamination with interstitial elements, etc. on alloy properties.
- Differentiation in research efforts regarding large and small numbers. Preference in the study of potentially big leverage effects, after appropriate consideration of life cycle analysis.
- Electrification of as many process steps as possible, including mining, sorting, crunching, benefication, synthesis, casting, forming and heat treatment.
- Study of different types of reductants and their mixtures and of the effects of mixed fossil and non-fossil reductants in primary synthesis.
- Influence of the use of low-quality ores, mixed minerals, mixed post-consumer scrap, as well as dumped and re-mined waste feedstock on synthesis and impurity range and final material properties.
- Sustainable synthesis from re-mined industry and post-consumer waste such as red mud from bauxite refinement and black mass from electronic waste.
- Extraction of metals from low-quality and chemically mixed minerals.
- Recovery of multiple metals in integrated process chains.
- Removal of all fossil fuels from heating treatment and melting processes.
- Development of deep reinforcement learning artificial intelligence methods to optimize reduction, heat treatment and processing schemes and to adapt process steps dynamically to adapt process conditions to alloy variants with varying impurity content.
- Improved recycling and upcycling instead of downcycling.
- Low-temperature and energy efficient heat treatment processes.
- Large-scale near-net shape manufacturing technologies such as thin slab casting and thin strip casting methods.
- Recycling and sustainable utilization of precious and specialty metals.
- Recycling from electronic and other high-tech wastes.
- Plant-based biometallurgy for toxic metal recovery from contaminated soils and products.
- Selective recovery of platinum group metals and rare earth metals.
- Alloy design for endless recycling: the science of “dirty” alloys and the effects of gradual scrap-related impurity accumulation on alloy properties, particularly on mechanical properties and corrosion.
- Use of all by-products, dust and slags and re-mining of valuable metals from them.
- Improving recycling rates of materials with rapidly changing chemistry and design.
- Improving recycling rates from complex consumer products and catalysts.
- Better use and design of monitoring, sensing, and data collections systems for artificial intelligence and better data analysis.
- Investigation of the gradually changing properties of metals after numerous recycling cycles, due to the possible accumulation of unwanted tramp elements.
- Removal of copper, tin and zinc from steels scrap during pyro- and/or electrometallurgical recycling.
- Improvement of thermodynamic and kinetic databases for the design and adjustment of alloy composition with regard to higher tolerance of scrap- and mineral-related impurities. This is particularly important when making alloys more tolerant against formation of undesired intermetallic phases or when aiming at modifying certain intermetallic phases that result from high tramp element concentrations.
- Pre-decoration and passivation of lattice defects to make them impurity tolerant.
- Revisiting and correcting currently permitted impurity ranges in established alloys for higher scrap use.
- Design of cross-over, broad-band, and multipurpose alloys to narrow down the chemical range of materials and by that make them more scrap-compatible.
- Closed collection and recycling loops for chemically complex niche and special alloys.
- Use of methods from artificial intelligence for the automated discovery of scrap-related impurity tolerant composition-microstructure-process-property relations.
- Adjustment of solidification, solutionizing and heat treatment to cope with effects of tramp elements.
- Low-temperature electrolysis of metals.
- Near-zero-waste extraction of metals from minerals without necessity of not dumping large by-product fractions. Use of all ingredients particularly from dilute ores.
- Sustainable polymetallurgical recycling methods, where not only the most precious metals are recovered but where more holistic approaches are developed which allow for the retrieval of all elements.
- Liquid–liquid metal separation methods.
- Opening the range of metal feedstock in terms of the minerals, ores, old scrap, in-line scrap, post-consumer waste, mixed scrap, nanointegrated multi-element scrap, industry waste, and hazardous, nonhazardous, and reductant feedstock used in metallurgy.
- Chemically driven phase transformations under redox conditions.
- Transport mechanisms and phase transformations in complex oxides.
- Diffusion of reductants in (chemically complex) minerals.
- Plasma chemistry for efficient and sustainable reduction in solid and liquid state.
- In-line monitoring of mass, fuel, and reductants in sustainable reduction processes.
- Development of suited donor and acceptor alloys in scrap cycles.
- Element partitioning studies of impurities from reductants and less-pure ores into metals.
- Joint science of “dirty” ores, alloys and reductants.
- Polymetallurgical co-reduction of multiple elements from mixed mineral sources.
- Genetic design and modification of bacteria and fungi for optimized bioaccumulation and bio-hydrometallurgy.
- Basic transport, nucleation and phase transformation mechanisms in direct reduction.
- Size and dispersion effects in solid-state reduction.
- Inert electrodes and refractory materials for electrolysis, including specifically carbon-free electrodes in electrowinning, electrolysis and plasmametallurgy.
- Exited states, nonequilibrium effects, contamination and reductant efficiency in plasma chemistry.
- Thermodynamics and kinetics of solid-state and liquid-state plasma reduction; equilibrium and nonequilibrium hydrogen and mixed-reductant plasma reduction principles.
- Catalysis effects in reduction processes.
- Redox thermodynamics for different metals in conjunction with non-fossil reductants.
- Transport, nucleation and general kinetics in redox reactions during non-fossil reduction and electrolysis.
- Effect of impurities and gangue elements on thermodynamics and kinetics of direct reduction.
- Use of pyrolysis methods in metal recycling.
- Solubility and intermetallic phase formation from scrap- and mineral-related impurity elements.
- Recycling of toxic and harmful metals.
- Thermodynamic competition between primary and secondary synthesis.
